# Contribution to the knowledge of Limoniidae (Diptera: Tipuloidea): first records of 244 species from various European countries

**DOI:** 10.3897/BDJ.9.e67085

**Published:** 2021-07-21

**Authors:** Levente-Péter Kolcsár, Pjotr Oosterbroek, Dmitry I. Gavryushin, Kjell Magne Olsen, Nikolai M. Paramonov, Valentin E. Pilipenko, Jaroslav Starý, Alexei Polevoi, Vladimir I. Lantsov, Eulalia Eiroa, Michael Andersson, Jukka Salmela, Clovis Quindroit, Micha C. d'Oliveira, E. Geoffrey Hancock, Jorge Mederos, Pete Boardman, Esko Viitanen, Kozo Watanabe

**Affiliations:** 1 Center for Marine Environmental Studies, Ehime University, Matsuyama, Japan Center for Marine Environmental Studies, Ehime University Matsuyama Japan; 2 Naturalis Biodiversity Center, Leiden, Netherlands Naturalis Biodiversity Center Leiden Netherlands; 3 Zoological Museum, Moscow Lomonosov State University, Moscow, Russia Zoological Museum, Moscow Lomonosov State University Moscow Russia; 4 BioFokus, Oslo, Norway BioFokus Oslo Norway; 5 Zoological Institute RAS, St. Petersburg, Russia Zoological Institute RAS St. Petersburg Russia; 6 Moscow State University, Moscow, Russia Moscow State University Moscow Russia; 7 Silesian Museum, Opava, Czech Republic Silesian Museum Opava Czech Republic; 8 Forest Research Institute KarRC RAS, Petrozavodsk, Russia Forest Research Institute KarRC RAS Petrozavodsk Russia; 9 Tembotov Institute of Ecology of Mountain Territories of Russian Academy of Sciences, Nalchik, Russia Tembotov Institute of Ecology of Mountain Territories of Russian Academy of Sciences Nalchik Russia; 10 Departamento de Zoología, Genética y Antropología Física, Facultad de Veterinaria, Universidad de Santiago de Compostela, Lugo, Spain Departamento de Zoología, Genética y Antropología Física, Facultad de Veterinaria, Universidad de Santiago de Compostela Lugo Spain; 11 Gripenbergsgatan 64, Huskvarna, Sweden Gripenbergsgatan 64 Huskvarna Sweden; 12 Regional Museum of Lapland, Rovaniemi, Finland Regional Museum of Lapland Rovaniemi Finland; 13 GRETIA, Angers, France GRETIA Angers France; 14 The Hunterian Museum, University of Glasgow, Glasgow, United Kingdom The Hunterian Museum, University of Glasgow Glasgow United Kingdom; 15 Museu de Ciències Naturals de Barcelona, Barcelona, Spain Museu de Ciències Naturals de Barcelona Barcelona Spain; 16 Natural England, Telford, United Kingdom Natural England Telford United Kingdom; 17 Vanhan-Mankkaan tie 29, Espoo, Finland Vanhan-Mankkaan tie 29 Espoo Finland

**Keywords:** biodiversity, Catalogue of the Craneflies of the World (CCW), collection data, database, Global Biodiversity Information Facility (GBIF), fauna, terminalia photos

## Abstract

**Background:**

Limoniidae is one of the most species-rich Dipteran families, with 661 reported species in Europe. Despite the fact that the European limoniid crane fly fauna has been studied ever since Carolus Linnaeus, it is still poorly known.

**New information:**

In this study, we summarise the taxonomic and faunistic studies of European Limoniidae, which described new species and reported first country records, between 2010 and 2020. We also report occurrence data of 244 Limoniidae species which represent the first country records or conformational records from various European countries, as we report ten species from Albania, one from Austria, thirty-seven from Belarus, five from Belgium, two from Bulgaria, two from Estonia, six from Finland, seven from France, fourteen from Greece, sixteen from Hungary, two from Iceland, six from Italy, ten from Latvia, one from Malta, nine from Montenegro, two from The Netherlands, ten from North Macedonia, forty-two from Norway, one from Poland, five from Portugal, twenty from Romania, thirty-eight from Serbia, six from Slovenia, five from Spain and seven species from Sweden for the first time. From the European territory of Russia, we report twenty-eight species from Central European Russia, seventy-two from East European Russia, fifteen from North European Russia, one from Northwest European Russia and seven from North Caucasus for the first time. Confirmatory records and corrigenda are also included.

## Introduction

Crane flies (superfamily Tipuloidea) are one of the most species-rich dipteran groups, with more than 15600 recognised taxa (Oosterbroek 2021). Using currently accepted systematics, Tipuloidea is divided into four families: Cylindrotomidae, Limoniidae, Pediciidae and Tipulidae (Starý 1992, Starý 2021). The family Limoniidae, the so-called short-palped crane flies, is the most speciose dipteran family with more than 10700 valid species, which represents approximately 0.55% of the catalogued species of the world (Roskov et al. 2019, Oosterbroek 2021).

**Figure 1.**
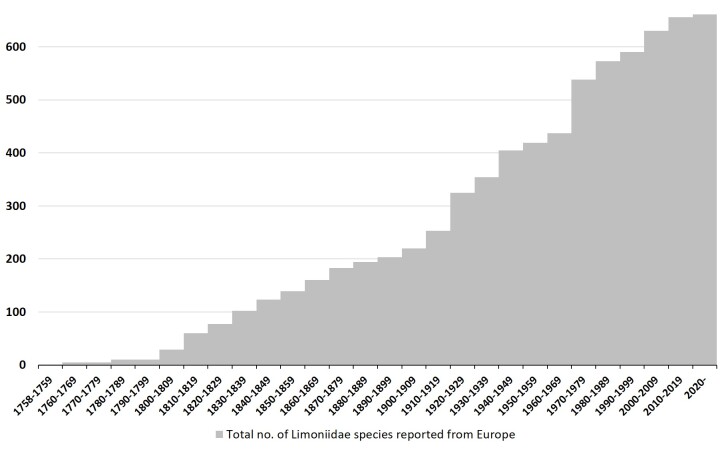
Cumulative number of European Limoniidae species per decade.

Since the description of the first limoniid crane fly species by C. Linnaeus in 1758, the number of described/reported species from Europe has continuously increased (Fig. 1). So far, 661 valid species and subspecies have been reported, according to Catalogue of the Craneflies of the World (Oosterbroek 2021). A total number of 31 new limoniid species are described here from Europe between 2010 and 2020 (Table 1). Most of the new species are found in the Meditterranean area and belong to the species-rich genus *Molophilus* Curtis, 1833.

In the last decade, many new faunistic studies have been published on European Limoniidae, of which we list the ones that report at least one species for the first time from a country (Table 2). Thanks to the increasing accessibility to publications, databases and community forums, more and more people have become interested in crane flies and provide useful information and specimens for faunistic and taxonomic studies. The Catalogue of the Craneflies of the World (CCW, available in https://ccw.naturalis.nl/index.php) is the indispensable online database for all kinds of research connected to crane flies. The CCW includes information about the taxonomy, distribution, ecology and biology of species, based both on scientific publications and on personal information sent/forwarded to Pjotr Oosterbroek. Often these personal observations represent the first record(s) of a species from a country or territory, but they are never published in scientific publications.

The aim of this paper is to publish the first records of limonid crane flies from various European countries. A collection of previously unpublished scientific records from CCW uploaded between 2010 and 2020 is also included, which represent the first country record(s) of the species in question. Confirmatory records and some corrigenda are also included. In total, we present 859 records belonging to 244 species.

## Materials and methods

### Geographic coverage

**Figure 2.**
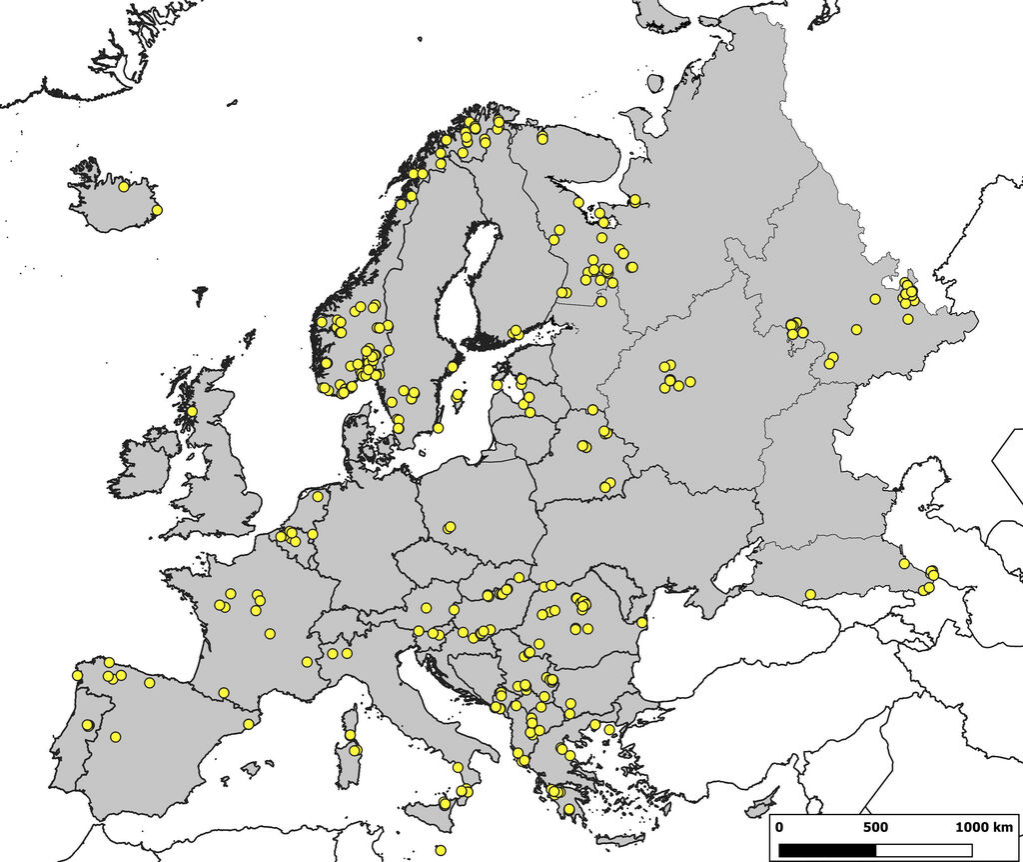
Geopolitical units of Europe according to the CCW, with sampling localities presented in this study.

In this study, we focus on the territory of Europe (Fig. 2), following CCW geopolitical unit concepts, which are based upon the Fauna Europaea, but the North Caucasus is also included (de Jong et al. 2014, Oosterbroek 2021). The European territory of Russia is divided into North, Northwest, Central, East and South European Russia and North Caucasus. The lists of the territories (respublika, oblast, kray), included in these six subdivisions, are presented in Suppl. material 1, including the Russian names of subdivisions and territories.

Abbreviations used for European territories of Russia:

**RUN** – North European Russia

**RUW** – Northwest European Russia

**RUC** – Central European Russia

**RUE** – East European Russia

**RUS** – South European Russia

**NC** – North Caucasus

### Sampling method and identification

Studied material has been collected by recognised manual field methods, such as sweep or hand-netting or trapped by the use of Malaise traps, SLAM-traps, light traps, trunk emergence traps, Lindgren funnel traps, pitfall traps, pan traps and window traps. Some records are based on photos or field observations. The preserved material is pinned or stored in ethanol and deposited in various public or private collections.

About 99% of the material is identified by the authors (excluding K. Watanabe) and the identifier(s) are responsible for the corresponding records. Collection data also avaible on the GBIF.org database (GBIF.org 2021).

Photographs of wing, body and terminalia of some rare species were taken by the first author, by using a Zeiss Stemi 508 stereomicroscope, equipped with a Canon Kiss M digital camera or with Optik microscope, equipped with a LM Digital SLR Adapter and Canon 650D camera.

### Deposits

**BioFokus** – BioFokus' office (https://biofokus.no), Oslo, Norway.

**CKLP** – Private Collection of L.-P. Kolcsár.

**FRIP** – Forest Research Institute, Petrozavodsk, Russia.

**HMUG** – Hunterian Museum, University of Glasgow, Glasgow, United Kindom.

**HNHM** – Hungarian National History Museum, Budapest, Hungary.

**IEMT** – Tembotov Institute of Ecology of Mountain Territories of Russian Academy of Sciences, Nalchik, Russia.

**LMM** – Regional Museum of Lapland, Rovaniemi, Finland.

**MCNB** – Natural Sciences Museum of Barcelona, Barcelona, Spain.

**MNHN** – National Museum of Natural History, Paris, France.

**MZLU** – Museum of Biology of Lund, Lund, Sweden.

**NBCN** – Naturalis Biodiversity Center in Leiden, The Netherlands.

**NHMO** – Natural History Museum at the University of Oslo, Oslo, Norway.

**NHRS** – Entomological Collections, Swedish Museum of Natural History, Stockholm, Sweden.

**OCIC** – Observatoire Conservatoire des Insectes de Corse, Corte, France.

**PCCQ** – Private Collection of C. Quindroit, Angers, France.

**PCJS** – Private Collection of J. Starý, Olomouc, Czechia.

**PCML** – Private Collection of M. Lindström, Sweden.

**PCKMO** – Private Collection of K. M. Olsen, Norway.

**PCMCO** – Private Collection of M. C. d'Oliveira, Haarlem, The Netherlands.

**USC** – Entomological Collection, Zoology Department, University of Santiago de Compostela, Spain.

**VPMC** – Private Collection of V. E. Pilipenko, Moscow, Russia.

**ZIN** – Zoological Institute, Russian Academy of Sciences, St Petersburg, Russia.

**ZMMU** – Zoological Museum of Moscow State University, Moscow, Russia.

**ZMUB** – Zoological Museum, University of Bergen, Bergen, Norway.

**ZMUT** – Zoological Museum of the University of Turku, Turku, Finland.

### First country record

Several species listed below have already been reported from different countries (geopolitical units) in the CCW, based on unpublished information and referred to as *in litt* in the CCW. Here, we publish the collection data for these records as well. If a record was credited to another person other than one of the authors of this paper, we highlight this, otherwise we simply refer to it as the first country record(s). General information and distribution of each species is available on the CCW and can be accessed by the links provided below.

## Taxon treatments

### Achyrolimonia decemmaculata

(Loew, 1873)

E5451CC5-020B-5B23-96DA-13C44E3807CC

https://ccw.naturalis.nl/detail.php?id=7640

#### Materials

- **Type status:** Other material. **Occurrence:** catalogNumber: 656442; occurrenceRemarks: 1 female; recordedBy: L. Breistøl; individualCount: 1; sex: female; preparations: Ethanol; occurrenceID: EU_LIM_001; **Taxon:** scientificName: Achyrolimoniadecemmaculata (Loew, 1873); family: Limoniidae; genus: Achyrolimonia; specificEpithet: decemmaculata; scientificNameAuthorship: (Loew, 1873); **Location:** country: Norway; stateProvince: Aust-Agder; municipality: Evje og Hornnes; locality: Kjetså; verbatimElevation: 180 m; minimumElevationInMeters: 180; decimalLatitude: 58.54398; decimalLongitude: 7.75592; **Identification:** identifiedBy: K.M. Olsen; **Event:** samplingProtocol: Malaise trap; eventDate: 2019-06-29/2019-09-16; verbatimEventDate: 29/Jul-16/Sep/2019; **Record Level:** institutionCode: NHMO; basisOfRecord: PreservedSpecimen
- **Type status:** Other material. **Occurrence:** catalogNumber: 611591; occurrenceRemarks: 1 female; recordedBy: S. Svendsen; individualCount: 1; sex: female; preparations: Ethanol; occurrenceID: EU_LIM_002; **Taxon:** scientificName: Achyrolimoniadecemmaculata (Loew, 1873); family: Limoniidae; genus: Achyrolimonia; specificEpithet: decemmaculata; scientificNameAuthorship: (Loew, 1873); **Location:** country: Norway; stateProvince: Aust-Agder; municipality: Birkenes; locality: Nordåsen; verbatimElevation: 85 m; minimumElevationInMeters: 85; decimalLatitude: 58.33342; decimalLongitude: 8.24004; **Identification:** identifiedBy: K.M. Olsen; **Event:** samplingProtocol: Light trap; eventDate: 2016-06/2016-08; verbatimEventDate: Jul-Aug/2016; **Record Level:** institutionCode: NHMO; basisOfRecord: PreservedSpecimen
- **Type status:** Other material. **Occurrence:** catalogNumber: 520545; occurrenceRemarks: 1 male; recordedBy: S. Svendsen | K. Berggren; individualCount: 1; sex: male; preparations: Ethanol; occurrenceID: EU_LIM_003; **Taxon:** scientificName: Achyrolimoniadecemmaculata (Loew, 1873); family: Limoniidae; genus: Achyrolimonia; specificEpithet: decemmaculata; scientificNameAuthorship: (Loew, 1873); **Location:** country: Norway; stateProvince: Aust-Agder; municipality: Birkenes; locality: Nordåsen; verbatimElevation: 85 m; minimumElevationInMeters: 85; decimalLatitude: 58.33342; decimalLongitude: 8.24004; **Identification:** identifiedBy: K.M. Olsen; **Event:** samplingProtocol: Light trap; eventDate: 2015-08; verbatimEventDate: Aug/2015; **Record Level:** institutionCode: PCKMO; basisOfRecord: PreservedSpecimen
- **Type status:** Other material. **Occurrence:** catalogNumber: 529283; occurrenceRemarks: 1 female; recordedBy: S. Svendsen | K. Berggren; individualCount: 1; sex: female; preparations: Ethanol; occurrenceID: EU_LIM_004; **Taxon:** scientificName: Achyrolimoniadecemmaculata (Loew, 1873); family: Limoniidae; genus: Achyrolimonia; specificEpithet: decemmaculata; scientificNameAuthorship: (Loew, 1873); **Location:** country: Norway; stateProvince: Aust-Agder; municipality: Birkenes; locality: Nordåsen; verbatimElevation: 85 m; minimumElevationInMeters: 85; decimalLatitude: 58.33342; decimalLongitude: 8.24004; **Identification:** identifiedBy: K.M. Olsen; **Event:** samplingProtocol: Light trap; eventDate: 2016-09; verbatimEventDate: Sep/2016; **Record Level:** institutionCode: PCKMO; basisOfRecord: PreservedSpecimen
- **Type status:** Other material. **Occurrence:** catalogNumber: 563683; occurrenceRemarks: 1 male; recordedBy: K. Berggren; individualCount: 1; sex: male; preparations: Ethanol; occurrenceID: EU_LIM_005; **Taxon:** scientificName: Achyrolimoniadecemmaculata (Loew, 1873); family: Limoniidae; genus: Achyrolimonia; specificEpithet: decemmaculata; scientificNameAuthorship: (Loew, 1873); **Location:** country: Norway; stateProvince: Vest-Agder; municipality: Kristiansand; locality: Nedre Timenes; verbatimElevation: 10 m; minimumElevationInMeters: 10; decimalLatitude: 58.16155; decimalLongitude: 8.10013; **Identification:** identifiedBy: K.M. Olsen; **Event:** samplingProtocol: Light trap; eventDate: 2017-06/2017-09; verbatimEventDate: Jul-Sep/2017; **Record Level:** institutionCode: NHMO; basisOfRecord: PreservedSpecimen
- **Type status:** Other material. **Occurrence:** occurrenceRemarks: 1 female; recordedBy: K.P. Tomkovich; individualCount: 1; sex: female; occurrenceID: EU_LIM_006; **Taxon:** scientificName: Achyrolimoniadecemmaculata (Loew, 1873); family: Limoniidae; genus: Achyrolimonia; specificEpithet: decemmaculata; scientificNameAuthorship: (Loew, 1873); **Location:** country: Russia; stateProvince: East European Russia; county: Bashkortostan Respublika; municipality: Beloretsk district; locality: S. Ural, Abzakovo-Murakaevo, Kryktytau Mnt.; verbatimElevation: 700 m; minimumElevationInMeters: 700; decimalLatitude: 53.54548; decimalLongitude: 58.48029; **Identification:** identifiedBy: V.E. Pilipenko; **Event:** samplingProtocol: Sweep net; eventDate: 2008-08-02/2008-08-08; verbatimEventDate: 2-8/Aug/2008; **Record Level:** institutionCode: VPMC; basisOfRecord: PreservedSpecimen
- **Type status:** Other material. **Occurrence:** occurrenceRemarks: 2 males; recordedBy: N.M. Paramonov; individualCount: 2; sex: male; occurrenceID: EU_LIM_007; **Taxon:** scientificName: Achyrolimoniadecemmaculata (Loew, 1873); family: Limoniidae; genus: Achyrolimonia; specificEpithet: decemmaculata; scientificNameAuthorship: (Loew, 1873); **Location:** country: Russia; stateProvince: East European Russia; county: Tatarstan Respublika; municipality: Verhneuslonsk district; locality: base “Zoostation”, 3,5 km NW Pustye Morkvashi env.; verbatimElevation: 80 m; minimumElevationInMeters: 80; decimalLatitude: 55.47005; decimalLongitude: 48.44092; **Identification:** identifiedBy: N.M. Paramonov; **Event:** samplingProtocol: Sweep net; eventDate: 2013-08-22/2013-08-26; verbatimEventDate: 22-26/Aug/2013; habitat: ravine, wetland; **Record Level:** institutionCode: ZIN; basisOfRecord: PreservedSpecimen

#### Distribution

First records from Norway and Russia: RUE.

### Antocha (Orimargula) alpigena

(Mik, 1883)

56AED1E1-4BF9-522B-A890-99B0704ED238

https://ccw.naturalis.nl/detail.php?id=7794

#### Materials

- **Type status:** Other material. **Occurrence:** occurrenceRemarks: 2 males; recordedBy: E. Eiroa; individualCount: 2; sex: male; preparations: Pinned; occurrenceID: EU_LIM_010; **Taxon:** scientificName: Antocha (Orimargula) alpigena (Mik, 1883); family: Limoniidae; genus: Antocha; subgenus: Orimargula; specificEpithet: alpigena; scientificNameAuthorship: (Mik, 1883); **Location:** country: Portugal; stateProvince: Guarda; municipality: Manteigas; locality: Vale do Zézere, serra da Estrela; verbatimElevation: 1240 m; minimumElevationInMeters: 1240; decimalLatitude: 40.32683; decimalLongitude: -7.57641; **Identification:** identifiedBy: E. Eiroa; **Event:** samplingProtocol: Sweep net; eventDate: 1992-05-30; verbatimEventDate: 30/May/1992; **Record Level:** institutionCode: USC; basisOfRecord: PreservedSpecimen

#### Distribution

First record from Portugal.

### Antocha (Antocha) vitripennis

(Meigen, 1830)

1EF02D40-5E7A-5415-87F9-F86C1BCA0B01

https://ccw.naturalis.nl/detail.php?id=7790

#### Materials

- **Type status:** Other material. **Occurrence:** occurrenceRemarks: 1 male; recordedBy: D.I. Gavryushin; individualCount: 1; sex: male; occurrenceID: EU_LIM_008; **Taxon:** scientificName: Antocha (Antocha) vitripennis (Meigen, 1830); family: Limoniidae; genus: Antocha; subgenus: Antocha; specificEpithet: vitripennis; scientificNameAuthorship: (Meigen, 1830); **Location:** country: Russia; stateProvince: East European Russia; county: Bashkortostan Respublika; municipality: Beloretsk district; locality: Nura River (ca. 4km W of Otnurok village), at the foot of Zolotyie Shishki (Golden Cones) Mts.; verbatimElevation: 607 m; minimumElevationInMeters: 607; decimalLatitude: 54.05155; decimalLongitude: 58.26887; **Identification:** identifiedBy: D.I. Gavryushin; **Event:** samplingProtocol: Sweep net; eventDate: 2013-08-22/2013-08-26; verbatimEventDate: 22-26/Aug/2013; **Record Level:** institutionCode: ZMMU; basisOfRecord: PreservedSpecimen
- **Type status:** Other material. **Occurrence:** occurrenceRemarks: 1 male; recordedBy: D.I. Gavryushin; individualCount: 1; sex: male; occurrenceID: EU_LIM_009; **Taxon:** scientificName: Antocha (Antocha) vitripennis (Meigen, 1830); family: Limoniidae; genus: Antocha; subgenus: Antocha; specificEpithet: vitripennis; scientificNameAuthorship: (Meigen, 1830); **Location:** country: Russia; stateProvince: East European Russia; county: Bashkortostan Respublika; municipality: Beloretsk district; locality: Makhmutovo env., Belaya River; verbatimElevation: 550 m; minimumElevationInMeters: 550; decimalLatitude: 54.33012; decimalLongitude: 58.80735; **Identification:** identifiedBy: D.I. Gavryushin; **Event:** samplingProtocol: Sweep net; eventDate: 2012-08-11; verbatimEventDate: 11/Aug/2012; **Record Level:** institutionCode: ZMMU; basisOfRecord: PreservedSpecimen

#### Distribution

First records from Russia: RUE.

### Arctoconopa forcipata forcipata

(Lundstrom, 1915)

18804118-4FC4-52D6-BF6E-33B722DEAA2A

https://ccw.naturalis.nl/detail.php?id=17608

#### Materials

- **Type status:** Other material. **Occurrence:** catalogNumber: 666172; occurrenceRemarks: 3 male+female; recordedBy: K.M. Olsen; individualCount: 3; sex: male, female; preparations: Ethanol; occurrenceID: EU_LIM_011; **Taxon:** scientificName: Arctoconopa forcipata forcipata (Lundstrom, 1915); family: Limoniidae; genus: Arctoconopa; specificEpithet: forcipata; infraspecificEpithet: forcipata; scientificNameAuthorship: (Lundstrom, 1915); **Location:** country: Norway; stateProvince: Finnmark; municipality: Tana; locality: Bodnesáttu – (N Seida); verbatimElevation: 5 m; minimumElevationInMeters: 5; decimalLatitude: 70.24973; decimalLongitude: 28.1806; **Identification:** identifiedBy: K.M. Olsen; **Event:** samplingProtocol: Sweep net; eventDate: 2020-06-07; verbatimEventDate: 07/Jul/2020; **Record Level:** institutionCode: PCKMO; basisOfRecord: PreservedSpecimen
- **Type status:** Other material. **Occurrence:** catalogNumber: 666255; occurrenceRemarks: 3 male+female; recordedBy: K.M. Olsen; individualCount: 3; sex: male, female; preparations: Ethanol; occurrenceID: EU_LIM_012; **Taxon:** scientificName: Arctoconopa forcipata forcipata (Lundstrom, 1915); family: Limoniidae; genus: Arctoconopa; specificEpithet: forcipata; infraspecificEpithet: forcipata; scientificNameAuthorship: (Lundstrom, 1915); **Location:** country: Norway; stateProvince: Finnmark; municipality: Tana; locality: Lismajoki–Nuorinjálbmi; verbatimElevation: 10 m; minimumElevationInMeters: 10; decimalLatitude: 70.14961; decimalLongitude: 28.18817; **Identification:** identifiedBy: K.M. Olsen; **Event:** samplingProtocol: Sweep net; eventDate: 2020-06-08; verbatimEventDate: 08/Jul/2020; **Record Level:** institutionCode: ZMUB; basisOfRecord: PreservedSpecimen
- **Type status:** Other material. **Occurrence:** catalogNumber: 666382; occurrenceRemarks: 9 male+female; recordedBy: K.M. Olsen; individualCount: 9; sex: male, female; occurrenceID: EU_LIM_013; **Taxon:** scientificName: Arctoconopa forcipata forcipata (Lundstrom, 1915); family: Limoniidae; genus: Arctoconopa; specificEpithet: forcipata; infraspecificEpithet: forcipata; scientificNameAuthorship: (Lundstrom, 1915); **Location:** country: Norway; stateProvince: Finnmark; municipality: Karasjok; locality: Sávkadasnjálbmi; verbatimElevation: 120 m; minimumElevationInMeters: 120; decimalLatitude: 69.54093; decimalLongitude: 25.83541; **Identification:** identifiedBy: K.M. Olsen; **Event:** samplingProtocol: Sweep net; eventDate: 2020-06-09; verbatimEventDate: 09/Jul/2020; **Record Level:** institutionCode: BioFokus; basisOfRecord: HumanObservation

#### Distribution

First records from Norway.

### Arctoconopa melampodia

(Loew, 1873)

95180CB7-49E7-5FD0-9BCE-C739414394BD

https://ccw.naturalis.nl/detail.php?id=216

#### Materials

- **Type status:** Other material. **Occurrence:** occurrenceRemarks: 2 females; recordedBy: D.I. Gavryushin; individualCount: 2; sex: female; preparations: Pinned; occurrenceID: EU_LIM_014; **Taxon:** scientificName: Arctoconopamelampodia (Loew, 1873); family: Limoniidae; genus: Arctoconopa; specificEpithet: melampodia; scientificNameAuthorship: (Loew, 1873); **Location:** country: Belarus; stateProvince: Gomel; county: Mazyr; locality: Mazyr; decimalLatitude: 52.02; decimalLongitude: 29.3; **Identification:** identifiedBy: D.I. Gavryushin; **Event:** samplingProtocol: Sweep net; eventDate: 2019-06-29/2019-07-31; verbatimEventDate: 29-31/Jul/2019; **Record Level:** institutionCode: ZMMU; basisOfRecord: PreservedSpecimen
- **Type status:** Other material. **Occurrence:** occurrenceRemarks: 1 male, 2 females; recordedBy: D.I. Gavryushin; individualCount: 3; sex: male, female; preparations: Pinned; occurrenceID: EU_LIM_015; **Taxon:** scientificName: Arctoconopamelampodia (Loew, 1873); family: Limoniidae; genus: Arctoconopa; specificEpithet: melampodia; scientificNameAuthorship: (Loew, 1873); **Location:** country: Belarus; stateProvince: Gomel Region; county: Mazyr District; locality: Mazyr; decimalLatitude: 52.05; decimalLongitude: 29.31; **Identification:** identifiedBy: D.I. Gavryushin; **Event:** samplingProtocol: Sweep net; eventDate: 2019-06-11/2019-06-14; verbatimEventDate: 11-14/Jun/2019; **Record Level:** institutionCode: ZMMU; basisOfRecord: PreservedSpecimen
- **Type status:** Other material. **Occurrence:** occurrenceRemarks: 1 male; recordedBy: J. Roháček; individualCount: 1; sex: male; preparations: Pinned; occurrenceID: EU_LIM_016; **Taxon:** scientificName: Arctoconopamelampodia (Loew, 1873); family: Limoniidae; genus: Arctoconopa; specificEpithet: melampodia; scientificNameAuthorship: (Loew, 1873); **Location:** country: Greece; stateProvince: Peloponnese; municipality: Kalivakia; locality: 1.1 km E, Pinos River; verbatimElevation: 97 m; minimumElevationInMeters: 97; decimalLatitude: 37.9; decimalLongitude: 21.55; **Identification:** identifiedBy: J. Starý; **Event:** eventDate: 2015-05-26; verbatimEventDate: 26/May/2015; **Record Level:** institutionCode: PCJS; basisOfRecord: PreservedSpecimen

#### Distribution

First records from Belarus and Greece (from mainland).

### Arctoconopa quadrivittata

(Siebke, 1872)

C7CECAF0-06AC-51D2-8950-A702565DC8A8

https://ccw.naturalis.nl/detail.php?id=220

#### Materials

- **Type status:** Other material. **Occurrence:** catalogNumber: NVO.TIPU0066; occurrenceRemarks: 10 males; recordedBy: J. Salmela; individualCount: 10; sex: male; preparations: Ethanol; occurrenceID: EU_LIM_017; **Taxon:** scientificName: Arctoconopaquadrivittata (Siebke, 1872); family: Limoniidae; genus: Arctoconopa; specificEpithet: quadrivittata; scientificNameAuthorship: (Siebke, 1872); **Location:** country: Finland; stateProvince: Lapponia inariensis; municipality: Utsjoki; locality: Pulmankijoki; verbatimElevation: 13 m; minimumElevationInMeters: 13; decimalLatitude: 69.9379; decimalLongitude: 28.0362; **Identification:** identifiedBy: J. Salmela; **Event:** samplingProtocol: Pit fall trap; eventDate: 2016-06-13; verbatimEventDate: 13/Jul/2016; **Record Level:** institutionCode: LMM; basisOfRecord: PreservedSpecimen

#### Distribution

The species was first recorded from Finland in Kahanpää (2017), but without collection data. Here, we publish the collection data of that record.

### Arctoconopa zonata

(Zetterstedt, 1851)

2F075043-7142-5C16-BA0D-FC0EE279F3DF

https://ccw.naturalis.nl/detail.php?id=224

#### Materials

- **Type status:** Other material. **Occurrence:** catalogNumber: GZ 119 (NHMO); occurrenceRemarks: 1 male; recordedBy: H. Siebke; individualCount: 1; sex: male; preparations: Pinned; occurrenceID: EU_LIM_018; **Taxon:** scientificName: Arctoconopazonata (Zetterstedt, 1851); family: Limoniidae; genus: Arctoconopa; specificEpithet: zonata; scientificNameAuthorship: (Zetterstedt, 1851); **Location:** country: Norway; stateProvince: Oslo; municipality: Oslo; locality: Youngs Løkke; verbatimElevation: 5 m; minimumElevationInMeters: 5; decimalLatitude: 59.91; decimalLongitude: 10.75; **Identification:** identifiedBy: J. Salmela | K.M. Olsen | Ø. Gammelmo; **Event:** eventDate: 1846-05-29; verbatimEventDate: 29/May/1846; **Record Level:** institutionCode: NHMO; basisOfRecord: PreservedSpecimen
- **Type status:** Other material. **Occurrence:** catalogNumber: 556284; occurrenceRemarks: 1 male; recordedBy: O.J. Lønnve; individualCount: 1; sex: male; preparations: Ethanol; occurrenceID: EU_LIM_019; **Taxon:** scientificName: Arctoconopazonata (Zetterstedt, 1851); family: Limoniidae; genus: Arctoconopa; specificEpithet: zonata; scientificNameAuthorship: (Zetterstedt, 1851); **Location:** country: Norway; stateProvince: Oppland; municipality: Lesja; locality: Kvernåi – (Kvennåe) Langs Lågen; verbatimElevation: 515 m; minimumElevationInMeters: 515; decimalLatitude: 62.08236; decimalLongitude: 9.03524; **Identification:** identifiedBy: J. Salmela; **Event:** samplingProtocol: Sweep net; eventDate: 2017-06-20/2017-06-22; verbatimEventDate: 20-22/Jun/2017; **Record Level:** institutionCode: PCKMO; basisOfRecord: PreservedSpecimen
- **Type status:** Other material. **Occurrence:** occurrenceRemarks: 1 male, 1 female; recordedBy: K.P. Tomkovich; individualCount: 2; sex: male, female; occurrenceID: EU_LIM_020; **Taxon:** scientificName: Arctoconopazonata (Zetterstedt, 1851); family: Limoniidae; genus: Arctoconopa; specificEpithet: zonata; scientificNameAuthorship: (Zetterstedt, 1851); **Location:** country: Russia; stateProvince: Central European Russia; county: Moskovskaya Oblast; municipality: Moscow; locality: Tsaritsino; verbatimElevation: 150 m; minimumElevationInMeters: 150; decimalLatitude: 55.60696; decimalLongitude: 37.68537; **Identification:** identifiedBy: V.E. Pilipenko; **Event:** samplingProtocol: Sweep net; eventDate: 1998-05-26; verbatimEventDate: 26/May/1998; **Record Level:** institutionCode: VPMC; basisOfRecord: PreservedSpecimen
- **Type status:** Other material. **Occurrence:** occurrenceRemarks: 2 males; recordedBy: V.E. Pilipenko; individualCount: 2; sex: male; occurrenceID: EU_LIM_021; **Taxon:** scientificName: Arctoconopazonata (Zetterstedt, 1851); family: Limoniidae; genus: Arctoconopa; specificEpithet: zonata; scientificNameAuthorship: (Zetterstedt, 1851); **Location:** country: Russia; stateProvince: Central European Russia; county: Moskovskaya Oblast; municipality: Solnechnogorsk district; locality: Chashnikovo; verbatimElevation: 220 m; minimumElevationInMeters: 220; decimalLatitude: 56.0375; decimalLongitude: 37.1874; **Identification:** identifiedBy: V.E. Pilipenko; **Event:** samplingProtocol: Sweep net; eventDate: 1995-05-27; verbatimEventDate: 27/May/1995; **Record Level:** institutionCode: VPMC; basisOfRecord: PreservedSpecimen

#### Distribution

First records from Russia: RUC. The species was considered doubtful for Norway, see discussion in Olsen et al. (2018), but has subsequently been verified. Here, we publish the collection data of those records. The older specimen was published by Siebke (1877) as *Eriopteraobscuripes* (see Lackschewitz 1933), under which name it was stored in NHMO until November 2017. However, Lackschewitz (1936) seems to have sorted it out, but this has not been followed up in Norwegian literature and the museum.

### Atypophthalmus (Atypophthalmus) inustus

(Meigen, 1818)

F5FED319-64AB-5217-B711-A09D45E2701B

https://ccw.naturalis.nl/detail.php?id=7868

#### Materials

- **Type status:** Other material. **Occurrence:** occurrenceRemarks: 1 male; recordedBy: D.I. Gavryushin; individualCount: 1; sex: male; preparations: Pinned; occurrenceID: EU_LIM_022; **Taxon:** scientificName: Atypophthalmus (Atypophthalmus) inustus (Meigen, 1818); family: Limoniidae; genus: Atypophthalmus; subgenus: Atypophthalmus; specificEpithet: inustus; scientificNameAuthorship: (Meigen, 1818); **Location:** country: Belarus; stateProvince: Minsk; county: Barysaw; locality: Vialikaje Stachava; verbatimElevation: 156 m; minimumElevationInMeters: 156; decimalLatitude: 54.26555; decimalLongitude: 28.38332; **Identification:** identifiedBy: D.I. Gavryushin; **Event:** samplingProtocol: Sweep net; eventDate: 2013-06-07; verbatimEventDate: 7/Jul/2013; **Record Level:** institutionCode: ZMMU; basisOfRecord: PreservedSpecimen
- **Type status:** Other material. **Occurrence:** occurrenceRemarks: 1 female; recordedBy: L.-P. Kolcsár; individualCount: 1; sex: female; preparations: Ethanol; occurrenceID: EU_LIM_023; **Taxon:** scientificName: Atypophthalmus (Atypophthalmus) inustus (Meigen, 1818); family: Limoniidae; genus: Atypophthalmus; subgenus: Atypophthalmus; specificEpithet: inustus; scientificNameAuthorship: (Meigen, 1818); **Location:** country: Latvia; municipality: Skaistkalne; verbatimElevation: 12 m; minimumElevationInMeters: 12; decimalLatitude: 56.411; decimalLongitude: 24.637; **Identification:** identifiedBy: L.-P. Kolcsár; **Event:** samplingProtocol: Sweep net; eventDate: 2018-06-19; verbatimEventDate: 19/Jul/2018; habitat: birch-spruce forest, small stream; **Record Level:** institutionCode: CKLP; basisOfRecord: PreservedSpecimen
- **Type status:** Other material. **Occurrence:** occurrenceRemarks: 2 males, 1 female; recordedBy: L.-P. Kolcsár; individualCount: 3; sex: male, female; preparations: Ethanol; occurrenceID: EU_LIM_024; **Taxon:** scientificName: Atypophthalmus (Atypophthalmus) inustus (Meigen, 1818); family: Limoniidae; genus: Atypophthalmus; subgenus: Atypophthalmus; specificEpithet: inustus; scientificNameAuthorship: (Meigen, 1818); **Location:** country: Latvia; municipality: Sigulda; locality: Gauja River; verbatimElevation: 13 m; minimumElevationInMeters: 13; decimalLatitude: 57.1505; decimalLongitude: 24.8168; **Identification:** identifiedBy: L.-P. Kolcsár; **Event:** samplingProtocol: Sweep net; eventDate: 2018-06-24; verbatimEventDate: 24/Jul/2018; **Record Level:** institutionCode: CKLP; basisOfRecord: PreservedSpecimen
- **Type status:** Other material. **Occurrence:** occurrenceRemarks: 1 female; recordedBy: D.I. Gavryushin; individualCount: 1; sex: female; occurrenceID: EU_LIM_025; **Taxon:** scientificName: Atypophthalmus (Atypophthalmus) inustus (Meigen, 1818); family: Limoniidae; genus: Atypophthalmus; subgenus: Atypophthalmus; specificEpithet: inustus; scientificNameAuthorship: (Meigen, 1818); **Location:** country: Russia; stateProvince: East European Russia; county: Bashkortostan Respublika; municipality: Beloretsk district; locality: Nura River (ca. 4km W of Otnurok village), at the foot of Zolotyie Shishki (Golden Cones) Mts.; verbatimElevation: 607 m; minimumElevationInMeters: 607; decimalLatitude: 54.05155; decimalLongitude: 58.26887; **Identification:** identifiedBy: D.I. Gavryushin; **Event:** samplingProtocol: Sweep net; eventDate: 2015-06-10; verbatimEventDate: 10/Jul/2015; **Record Level:** institutionCode: ZMMU; basisOfRecord: PreservedSpecimen
- **Type status:** Other material. **Occurrence:** occurrenceRemarks: 1 female; recordedBy: V.E. Pilipenko; individualCount: 1; sex: female; occurrenceID: EU_LIM_026; **Taxon:** scientificName: Atypophthalmus (Atypophthalmus) inustus (Meigen, 1818); family: Limoniidae; genus: Atypophthalmus; subgenus: Atypophthalmus; specificEpithet: inustus; scientificNameAuthorship: (Meigen, 1818); **Location:** country: Russia; stateProvince: Central European Russia; county: Moskovskaya Oblast; municipality: Solnechnogorsk district; locality: Chashnikovo; verbatimElevation: 220 m; minimumElevationInMeters: 220; decimalLatitude: 56.0375; decimalLongitude: 37.1874; **Identification:** identifiedBy: V.E. Pilipenko; **Event:** samplingProtocol: Sweep net; eventDate: 1994-06-22; verbatimEventDate: 22/Jul/1994; **Record Level:** institutionCode: VPMC; basisOfRecord: PreservedSpecimen
- **Type status:** Other material. **Occurrence:** occurrenceRemarks: 1 male; recordedBy: N.M. Paramonov; individualCount: 1; sex: male; occurrenceID: EU_LIM_027; **Taxon:** scientificName: Atypophthalmus (Atypophthalmus) inustus (Meigen, 1818); family: Limoniidae; genus: Atypophthalmus; subgenus: Atypophthalmus; specificEpithet: inustus; scientificNameAuthorship: (Meigen, 1818); **Location:** country: Russia; stateProvince: East European Russia; county: Tatarstan Respublika; municipality: Laishevo district; locality: Volga-Kama State Nature Biosphere Reserve, «Saraly»; verbatimElevation: 71 m; minimumElevationInMeters: 71; decimalLatitude: 55.29303; decimalLongitude: 49.29976; **Identification:** identifiedBy: N.M. Paramonov; **Event:** samplingProtocol: Sweep net; eventDate: 2009-06-18; verbatimEventDate: 18/Jun/2009; habitat: wetland; **Record Level:** institutionCode: ZIN; basisOfRecord: PreservedSpecimen
- **Type status:** Other material. **Occurrence:** occurrenceRemarks: 1 male, 1 female; recordedBy: L.-P. Kolcsár | E. Török; individualCount: 2; sex: male, female; preparations: Ethanol; occurrenceID: EU_LIM_028; **Taxon:** scientificName: Atypophthalmus (Atypophthalmus) inustus (Meigen, 1818); family: Limoniidae; genus: Atypophthalmus; subgenus: Atypophthalmus; specificEpithet: inustus; scientificNameAuthorship: (Meigen, 1818); **Location:** country: Serbia; municipality: Banja Jošanica; locality: Kopaonik Mts., Paljestica River; verbatimElevation: 871 m; minimumElevationInMeters: 871; decimalLatitude: 43.36343; decimalLongitude: 20.7477; **Identification:** identifiedBy: L.-P. Kolcsár; **Event:** samplingProtocol: Sweep net; eventDate: 2017-06-22; verbatimEventDate: 22/Jun/2017; **Record Level:** institutionCode: CKLP; basisOfRecord: PreservedSpecimen

#### Distribution

First records from Belarus, Latvia, Russia: RUC, RUE and Serbia.

### Atypophthalmus (Microlimonia) machidai

(Alexander, 1921)

88344B78-DCE1-5B93-A930-7C10B8C7B601

https://ccw.naturalis.nl/detail.php?id=7907

#### Materials

- **Type status:** Other material. **Occurrence:** occurrenceRemarks: 1 male; recordedBy: M.C. de Haas; individualCount: 1; sex: male; preparations: Ethanol; occurrenceID: EU_LIM_862; **Taxon:** scientificName: Atypophthalmus (Microlimonia) machidai (Alexander, 1921); family: Limoniidae; genus: Atypophthalmus; subgenus: (Microlimonia); specificEpithet: machidai; scientificNameAuthorship: (Alexander, 1921); **Location:** country: Slovenia; municipality: Ljubno; locality: In forest near Savina; verbatimElevation: 490 m; minimumElevationInMeters: 490; decimalLatitude: 46.332; decimalLongitude: 14.839; **Identification:** identifiedBy: M.C. d'Oliveira; **Event:** samplingProtocol: Light trap; eventDate: 2020-12-8; verbatimEventDate: 12/august/2020; habitat: Edge of forest with small stream; **Record Level:** institutionCode: PCMCO; basisOfRecord: PreservedSpecimen

#### Distribution

First record from Slovenia.

### Atypophthalmus (Atypophthalmus) umbratus

(de Meijere, 1911)

B846A55A-F29E-5548-91D9-C5EF61A79DA6

https://ccw.naturalis.nl/detail.php?id=7901

#### Materials

- **Type status:** Other material. **Occurrence:** occurrenceRemarks: 1 male; recordedBy: J. Soors; individualCount: 1; sex: male; occurrenceID: EU_LIM_029; **Taxon:** scientificName: Atypophthalmus (Atypophthalmus) umbratus (de Meijere, 1911); family: Limoniidae; genus: Atypophthalmus; subgenus: Atypophthalmus; specificEpithet: umbratus; scientificNameAuthorship: (de Meijere, 1911); **Location:** country: Belgium; stateProvince: Vlaams-Brabant; municipality: Meise; decimalLatitude: 50.926; decimalLongitude: 4.328; **Identification:** identifiedBy: P. Oosterbroek; **Event:** eventDate: 2019-11-15; verbatimEventDate: 15/Nov/2019; habitat: in glasshouse of local hortus; **Record Level:** basisOfRecord: HumanObservation

#### Distribution

The species was reported from Belgium by J. Soors (in litt. 2020) in the CCW; here, we publish the collection data of that record.

### Austrolimnophila (Austrolimnophila) latistyla

Starý, 1977

0AA93ABF-92B9-552B-B2A1-DFBB632F8C61

https://ccw.naturalis.nl/detail.php?id=4993

#### Materials

- **Type status:** Other material. **Occurrence:** occurrenceRemarks: 1 male; recordedBy: L.-P. Kolcsár; individualCount: 1; sex: male; preparations: Ethanol; occurrenceID: EU_LIM_032; **Taxon:** scientificName: Austrolimnophila (Austrolimnophila) latistyla Starý, 1977; family: Limoniidae; genus: Austrolimnophila; subgenus: Austrolimnophila; specificEpithet: latistyla; scientificNameAuthorship: Starý, 1977; **Location:** country: Albania; municipality: Iljas; locality: Gjipe Canyon; verbatimElevation: 307 m; minimumElevationInMeters: 307; decimalLatitude: 40.1428; decimalLongitude: 19.6781; **Identification:** identifiedBy: L.-P. Kolcsár; **Event:** samplingProtocol: Sweep net; eventDate: 2016-05-05; verbatimEventDate: 05/May/2016; **Record Level:** institutionCode: CKLP; basisOfRecord: PreservedSpecimen
- **Type status:** Other material. **Occurrence:** occurrenceRemarks: 2 males; recordedBy: J. Starý; individualCount: 2; sex: male; preparations: Pinned; occurrenceID: EU_LIM_033; **Taxon:** scientificName: Austrolimnophila (Austrolimnophila) latistyla Starý, 1977; family: Limoniidae; genus: Austrolimnophila; subgenus: Austrolimnophila; specificEpithet: latistyla; scientificNameAuthorship: Starý, 1977; **Location:** island: Sardinia; country: Italy; stateProvince: Sardinia; municipality: Padru; locality: 1.6 km E, Rio su Lernu; verbatimElevation: 125 m; minimumElevationInMeters: 125; decimalLatitude: 40.766; decimalLongitude: 9.533; **Identification:** identifiedBy: J. Starý; **Event:** eventDate: 2014-05-11; verbatimEventDate: 11/May/2014; **Record Level:** institutionCode: PCJS; basisOfRecord: PreservedSpecimen
- **Type status:** Other material. **Occurrence:** occurrenceRemarks: 1 male; recordedBy: J. Starý; individualCount: 1; sex: male; preparations: Pinned; occurrenceID: EU_LIM_034; **Taxon:** scientificName: Austrolimnophila (Austrolimnophila) latistyla Starý, 1977; family: Limoniidae; genus: Austrolimnophila; subgenus: Austrolimnophila; specificEpithet: latistyla; scientificNameAuthorship: Starý, 1977; **Location:** island: Sicily; country: Italy; stateProvince: Sicily; municipality: Ucria; locality: 0.9 km S, torrente Praculla; verbatimElevation: 620 m; minimumElevationInMeters: 620; decimalLatitude: 38.0383; decimalLongitude: 14.8833; **Identification:** identifiedBy: J. Starý; **Event:** eventDate: 2016-04-27; verbatimEventDate: 27/Apr/2016; **Record Level:** institutionCode: PCJS; basisOfRecord: PreservedSpecimen
- **Type status:** Other material. **Occurrence:** occurrenceRemarks: 2 males; recordedBy: J. Starý; individualCount: 2; sex: male; preparations: Pinned; occurrenceID: EU_LIM_035; **Taxon:** scientificName: Austrolimnophila (Austrolimnophila) latistyla Starý, 1977; family: Limoniidae; genus: Austrolimnophila; subgenus: Austrolimnophila; specificEpithet: latistyla; scientificNameAuthorship: Starý, 1977; **Location:** island: Sicily; country: Italy; stateProvince: Sicily; municipality: Raccuja; locality: 0.9 km W, Fiumara di Sinagra; verbatimElevation: 450 m; minimumElevationInMeters: 450; decimalLatitude: 38.05389; decimalLongitude: 14.90083; **Identification:** identifiedBy: J. Starý; **Event:** eventDate: 2016-04-27; verbatimEventDate: 27/Apr/2016; **Record Level:** institutionCode: PCJS; basisOfRecord: PreservedSpecimen
- **Type status:** Other material. **Occurrence:** occurrenceRemarks: 3 males; recordedBy: J. Starý; individualCount: 3; sex: male; preparations: Pinned; occurrenceID: EU_LIM_036; **Taxon:** scientificName: Austrolimnophila (Austrolimnophila) latistyla Starý, 1977; family: Limoniidae; genus: Austrolimnophila; subgenus: Austrolimnophila; specificEpithet: latistyla; scientificNameAuthorship: Starý, 1977; **Location:** island: Sicily; country: Italy; stateProvince: Sicily; municipality: Raccuja; locality: 0.9 km W, Fiumara di Sinagra; verbatimElevation: 450 m; minimumElevationInMeters: 450; decimalLatitude: 38.05389; decimalLongitude: 14.90083; **Identification:** identifiedBy: J. Starý; **Event:** eventDate: 2016-04-22; verbatimEventDate: 22/Apr/2016; **Record Level:** institutionCode: PCJS; basisOfRecord: PreservedSpecimen
- **Type status:** Other material. **Occurrence:** occurrenceRemarks: 1 male; recordedBy: J. Starý; individualCount: 1; sex: male; preparations: Pinned; occurrenceID: EU_LIM_037; **Taxon:** scientificName: Austrolimnophila (Austrolimnophila) latistyla Starý, 1977; family: Limoniidae; genus: Austrolimnophila; subgenus: Austrolimnophila; specificEpithet: latistyla; scientificNameAuthorship: Starý, 1977; **Location:** island: Sicily; country: Italy; stateProvince: Sicily; municipality: Raccuja; locality: 0.9 km W, Fiumara di Sinagra; verbatimElevation: 450 m; minimumElevationInMeters: 450; decimalLatitude: 38.05389; decimalLongitude: 14.90083; **Identification:** identifiedBy: J. Starý; **Event:** eventDate: 2016-04-23; verbatimEventDate: 23/Apr/2016; **Record Level:** institutionCode: PCJS; basisOfRecord: PreservedSpecimen
- **Type status:** Other material. **Occurrence:** occurrenceRemarks: 3 males; recordedBy: J. Starý; individualCount: 3; sex: male; preparations: Pinned; occurrenceID: EU_LIM_038; **Taxon:** scientificName: Austrolimnophila (Austrolimnophila) latistyla Starý, 1977; family: Limoniidae; genus: Austrolimnophila; subgenus: Austrolimnophila; specificEpithet: latistyla; scientificNameAuthorship: Starý, 1977; **Location:** island: Sicily; country: Italy; stateProvince: Sicily; municipality: Raccuja; locality: 0.9 km W, Fiumara di Sinagra; verbatimElevation: 450 m; minimumElevationInMeters: 450; decimalLatitude: 38.05389; decimalLongitude: 14.90083; **Identification:** identifiedBy: J. Starý; **Event:** eventDate: 2016-04-20; verbatimEventDate: 20/Apr/2016; **Record Level:** institutionCode: PCJS; basisOfRecord: PreservedSpecimen
- **Type status:** Other material. **Occurrence:** occurrenceRemarks: 2 males; recordedBy: L.-P. Kolcsár; individualCount: 2; sex: male; preparations: Ethanol; occurrenceID: EU_LIM_039; **Taxon:** scientificName: Austrolimnophila (Austrolimnophila) latistyla Starý, 1977; family: Limoniidae; genus: Austrolimnophila; subgenus: Austrolimnophila; specificEpithet: latistyla; scientificNameAuthorship: Starý, 1977; **Location:** country: Montenegro; municipality: Morinj; locality: Morinj brook; verbatimElevation: 67 m; minimumElevationInMeters: 67; decimalLatitude: 42.48935; decimalLongitude: 18.62896; **Identification:** identifiedBy: L.-P. Kolcsár; **Event:** samplingProtocol: Sweep net; eventDate: 2010-05-13; verbatimEventDate: 13/May/2010; habitat: dry stream bed; **Record Level:** institutionCode: CKLP; basisOfRecord: PreservedSpecimen

#### Distribution

First records from Albania and Montenegro. The species reported before from mainland Italy and here, we publish the first records from Sardinia and Sicily.

### Austrolimnophila (Austrolimnophila) ochracea

(Meigen, 1804)

784A38DA-9A8E-5552-8FD1-C5F6E4A282AF

https://ccw.naturalis.nl/detail.php?id=5027

#### Materials

- **Type status:** Other material. **Occurrence:** occurrenceRemarks: 1 male; recordedBy: D.I. Gavryushin; individualCount: 1; sex: male; preparations: Pinned; occurrenceID: EU_LIM_040; **Taxon:** scientificName: Austrolimnophila (Austrolimnophila) ochracea (Meigen, 1804); family: Limoniidae; genus: Austrolimnophila; subgenus: Austrolimnophila; specificEpithet: ochracea; scientificNameAuthorship: (Meigen, 1804); **Location:** country: Belarus; stateProvince: Minsk; county: Barysaw; locality: Vialikaje Stachava; verbatimElevation: 156 m; minimumElevationInMeters: 156; decimalLatitude: 54.26555; decimalLongitude: 28.38332; **Identification:** identifiedBy: D.I. Gavryushin; **Event:** samplingProtocol: Sweep net; eventDate: 2013-06-07; verbatimEventDate: 7/Jul/2013; **Record Level:** institutionCode: ZMMU; basisOfRecord: PreservedSpecimen

#### Distribution

First record from Belarus.

### Austrolimnophila (Austrolimnophila) unica

(Osten Sacken, 1869)

324A2603-6B53-5862-9A85-59D6E37AF896

https://ccw.naturalis.nl/detail.php?id=4912

#### Materials

- **Type status:** Other material. **Occurrence:** occurrenceRemarks: 1 male; recordedBy: D.I. Gavryushin; individualCount: 1; sex: male; occurrenceID: EU_LIM_030; **Taxon:** scientificName: Austrolimnophila (Archilimnophila) unica (Osten Sacken, 1869); family: Limoniidae; genus: Austrolimnophila; subgenus: Archilimnophila; specificEpithet: unica; scientificNameAuthorship: (Osten Sacken, 1869); **Location:** country: Russia; stateProvince: East European Russia; county: Bashkortostan Respublika; municipality: Beloretsk district; locality: Abzakovo env., Kulsugady River; verbatimElevation: 531 m; minimumElevationInMeters: 531; decimalLatitude: 53.83795; decimalLongitude: 58.5823; **Identification:** identifiedBy: D.I. Gavryushin; **Event:** samplingProtocol: Sweep net; eventDate: 2015-06-17; verbatimEventDate: 17/Jul/2015; **Record Level:** institutionCode: ZMMU; basisOfRecord: PreservedSpecimen
- **Type status:** Other material. **Occurrence:** occurrenceRemarks: 1 male; recordedBy: L.-P. Kolcsár | E. Török; individualCount: 1; sex: male; preparations: Ethanol; occurrenceID: EU_LIM_031; **Taxon:** scientificName: Austrolimnophila (Archilimnophila) unica (Osten Sacken, 1869); family: Limoniidae; genus: Austrolimnophila; subgenus: Archilimnophila; specificEpithet: unica; scientificNameAuthorship: (Osten Sacken, 1869); **Location:** country: Serbia; municipality: Kopaonik; locality: Kopaonik Mts.; verbatimElevation: 1600 m; minimumElevationInMeters: 1600; decimalLatitude: 43.2981; decimalLongitude: 20.78706; **Identification:** identifiedBy: L.-P. Kolcsár; **Event:** samplingProtocol: Sweep net; eventDate: 2017-06-22; verbatimEventDate: 22/Jun/2017; **Record Level:** institutionCode: CKLP; basisOfRecord: PreservedSpecimen

#### Distribution

First records from Russia: RUE and Serbia.

### Baeoura malickyi

Mendl and Tjeder, 1976

0A3418C2-7314-5CB0-87B4-5E01451DCE65

https://ccw.naturalis.nl/detail.php?id=427

#### Materials

- **Type status:** Other material. **Occurrence:** occurrenceRemarks: 1 female; recordedBy: M.C. d'Oliveira; individualCount: 1; sex: female; preparations: Ethanol; occurrenceID: EU_LIM_859; **Taxon:** scientificName: Baeouramalickyi Mendl and Tjeder, 1976; family: Limoniidae; genus: Baeoura; specificEpithet: malickyi; scientificNameAuthorship: Mendl and Tjeder, 1976; **Location:** country: Slovenia; municipality: Kranjska Gora; locality: Gozd Martuljek, 10 meters from the Sava river; verbatimElevation: 745 m; minimumElevationInMeters: 745; decimalLatitude: 46.483; decimalLongitude: 13.837539; **Identification:** identifiedBy: M.C. d'Oliveira; **Event:** samplingProtocol: Light trap; eventDate: 2019-20-8; verbatimEventDate: 20/August/2019; habitat: Small woodland next to river; **Record Level:** institutionCode: PCMCO; basisOfRecord: PreservedSpecimen

#### Distribution

First record from Slovenia.

### Cheilotrichia (Empeda) affinis

(Lackschewitz, 1927)

8897208B-8A70-58F9-AFEC-54633AF5AD64

https://ccw.naturalis.nl/detail.php?id=494

#### Materials

- **Type status:** Other material. **Occurrence:** occurrenceRemarks: 1 male; recordedBy: G. Zilahi-Sebess; individualCount: 1; sex: male; preparations: Pinned; occurrenceID: EU_LIM_044; **Taxon:** scientificName: Cheilotrichia (Empeda) affinis (Lackschewitz, 1927); family: Limoniidae; genus: Cheilotrichia; subgenus: Empeda; specificEpithet: affinis; scientificNameAuthorship: (Lackschewitz, 1927); **Location:** country: Hungary; stateProvince: Heves; municipality: Nagyvisnyó; locality: Bánkút, Bügg Mts.; decimalLatitude: 48.10051; decimalLongitude: 20.47942; **Identification:** identifiedBy: L.-P. Kolcsár; **Event:** eventDate: 1955-10-12; verbatimEventDate: 12/Oct/1955; habitat: in beech forest; **Record Level:** institutionCode: HNHM; basisOfRecord: PreservedSpecimen

#### Distribution

First record from Hungary.

### Cheilotrichia (Empeda) alpina

(Strobl, 1895)

A6FDF95E-4BD2-5ADE-A906-DD85F34B3F12

https://ccw.naturalis.nl/detail.php?id=497

#### Materials

- **Type status:** Other material. **Occurrence:** catalogNumber: 605503, 605504, 605505, 605537; occurrenceRemarks: 4 males; recordedBy: K.M. Olsen; individualCount: 4; sex: male; preparations: Ethanol; occurrenceID: EU_LIM_045; **Taxon:** scientificName: Cheilotrichia (Empeda) alpina (Strobl, 1895); family: Limoniidae; genus: Cheilotrichia; subgenus: Empeda; specificEpithet: alpina; scientificNameAuthorship: (Strobl, 1895); **Location:** country: Norway; stateProvince: Hedmark; municipality: Åmot; locality: Deifjell-lia; verbatimElevation: 515 m; minimumElevationInMeters: 515; decimalLatitude: 61.28427; decimalLongitude: 11.50497; **Identification:** identifiedBy: K.M. Olsen; **Event:** samplingProtocol: Malaise trap; eventDate: 2018-05-05/2018-07-06; verbatimEventDate: 05/May-06/Jul/2018; **Record Level:** institutionCode: ZMUB | PCKMO; basisOfRecord: PreservedSpecimen
- **Type status:** Other material. **Occurrence:** catalogNumber: 606525; occurrenceRemarks: 1 male; recordedBy: K.M. Olsen; individualCount: 1; sex: male; preparations: Ethanol; occurrenceID: EU_LIM_046; **Taxon:** scientificName: Cheilotrichia (Empeda) alpina (Strobl, 1895); family: Limoniidae; genus: Cheilotrichia; subgenus: Empeda; specificEpithet: alpina; scientificNameAuthorship: (Strobl, 1895); **Location:** country: Norway; stateProvince: Hedmark; municipality: Åmot; locality: Deifjell-lia; verbatimElevation: 515 m; minimumElevationInMeters: 515; decimalLatitude: 61.28427; decimalLongitude: 11.50497; **Identification:** identifiedBy: K.M. Olsen; **Event:** samplingProtocol: Malaise trap; eventDate: 2018-06-06/2018-09-22; verbatimEventDate: 06/Jul-22/Sep/2018; **Record Level:** institutionCode: NHMO; basisOfRecord: PreservedSpecimen
- **Type status:** Other material. **Occurrence:** catalogNumber: 521313; occurrenceRemarks: 1 male; recordedBy: K. Berggren | L. Byrkjeland; individualCount: 1; sex: male; preparations: Ethanol; occurrenceID: EU_LIM_047; **Taxon:** scientificName: Cheilotrichia (Empeda) alpina (Strobl, 1895); family: Limoniidae; genus: Cheilotrichia; subgenus: Empeda; specificEpithet: alpina; scientificNameAuthorship: (Strobl, 1895); **Location:** country: Norway; stateProvince: Sogn og Fjordane; municipality: Luster; locality: Solvorn – Ved Løteigane; verbatimElevation: 70 m; minimumElevationInMeters: 70; decimalLatitude: 61.30313; decimalLongitude: 7.24108; **Identification:** identifiedBy: K.M. Olsen; **Event:** samplingProtocol: Light trap; eventDate: 2016-09-02/2016-09-15; verbatimEventDate: 02-15/Sep/2016; **Record Level:** institutionCode: PCKMO; basisOfRecord: PreservedSpecimen
- **Type status:** Other material. **Occurrence:** catalogNumber: 553835; occurrenceRemarks: 1 male; recordedBy: K. Berggren | R.-A. Golf; individualCount: 1; sex: male; preparations: Ethanol; occurrenceID: EU_LIM_048; **Taxon:** scientificName: Cheilotrichia (Empeda) alpina (Strobl, 1895); family: Limoniidae; genus: Cheilotrichia; subgenus: Empeda; specificEpithet: alpina; scientificNameAuthorship: (Strobl, 1895); **Location:** country: Norway; stateProvince: Sogn og Fjordane; municipality: Lærdal; locality: Sløgrandane – NE Nygard; verbatimElevation: 70 m; minimumElevationInMeters: 70; decimalLatitude: 61.04407; decimalLongitude: 7.62354; **Identification:** identifiedBy: K.M. Olsen; **Event:** samplingProtocol: Light trap; eventDate: 2017-06; verbatimEventDate: Jul/2017; **Record Level:** institutionCode: PCKMO; basisOfRecord: PreservedSpecimen
- **Type status:** Other material. **Occurrence:** catalogNumber: 577270; occurrenceRemarks: 1 male; recordedBy: K. Berggren | R.-A. Golf; individualCount: 1; sex: male; preparations: Ethanol; occurrenceID: EU_LIM_049; **Taxon:** scientificName: Cheilotrichia (Empeda) alpina (Strobl, 1895); family: Limoniidae; genus: Cheilotrichia; subgenus: Empeda; specificEpithet: alpina; scientificNameAuthorship: (Strobl, 1895); **Location:** country: Norway; stateProvince: Sogn og Fjordane; municipality: Lærdal; locality: Sløgrandane – NE Nygard; verbatimElevation: 70 m; minimumElevationInMeters: 70; decimalLatitude: 61.04407; decimalLongitude: 7.62354; **Identification:** identifiedBy: K.M. Olsen; **Event:** samplingProtocol: Light trap; eventDate: 2017-05-11/2017-06-24; verbatimEventDate: 11/Sly-24/Jun/2017; **Record Level:** institutionCode: NHMO; basisOfRecord: PreservedSpecimen

#### Distribution

First records from Norway.

### Cheilotrichia (Empeda) cinerascens

(Meigen, 1804)

FC86DD89-D2AF-59FF-85A0-B7CDE9931420

https://ccw.naturalis.nl/detail.php?id=516

#### Materials

- **Type status:** Other material. **Occurrence:** occurrenceRemarks: 2 females; recordedBy: D.I. Gavryushin; individualCount: 2; sex: female; occurrenceID: EU_LIM_050; **Taxon:** scientificName: Cheilotrichia (Empeda) cinerascens (Meigen, 1804); family: Limoniidae; genus: Cheilotrichia; subgenus: Empeda; specificEpithet: cinerascens; scientificNameAuthorship: (Meigen, 1804); **Location:** country: Russia; stateProvince: East European Russia; county: Bashkortostan Respublika; municipality: Beloretsk district; locality: Nura River (ca. 4km W of Otnurok village), at the foot of Zolotyie Shishki (Golden Cones) Mts.; verbatimElevation: 607 m; minimumElevationInMeters: 607; decimalLatitude: 54.05155; decimalLongitude: 58.26887; **Identification:** identifiedBy: D.I. Gavryushin; **Event:** samplingProtocol: Sweep net; eventDate: 2012-08-09; verbatimEventDate: Aug-09-2012; **Record Level:** institutionCode: ZMMU; basisOfRecord: PreservedSpecimen

#### Distribution

First record from Russia: RUE.

### Cheilotrichia (Cheilotrichia) imbuta

(Meigen, 1818)

33EE5E17-C901-5F27-B0B4-83C22A0E7DDE

https://ccw.naturalis.nl/detail.php?id=482

#### Materials

- **Type status:** Other material. **Occurrence:** occurrenceRemarks: 1 male; recordedBy: D.I. Gavryushin; individualCount: 1; sex: male; preparations: Pinned; occurrenceID: EU_LIM_041; **Taxon:** scientificName: Cheilotrichia (Cheilotrichia) imbuta (Meigen, 1818); family: Limoniidae; genus: Cheilotrichia; subgenus: Cheilotrichia; specificEpithet: imbuta; scientificNameAuthorship: (Meigen, 1818); **Location:** country: Belarus; stateProvince: Minsk; county: Barysaw; locality: Glivin; verbatimElevation: 161 m; minimumElevationInMeters: 161; decimalLatitude: 54.14902; decimalLongitude: 28.63648; **Identification:** identifiedBy: D.I. Gavryushin; **Event:** samplingProtocol: Sweep net; eventDate: 2013-06-06; verbatimEventDate: 6/Jul/2013; **Record Level:** institutionCode: ZMMU; basisOfRecord: PreservedSpecimen
- **Type status:** Other material. **Occurrence:** occurrenceRemarks: 2 males; recordedBy: N.M. Paramonov; individualCount: 2; sex: male; occurrenceID: EU_LIM_042; **Taxon:** scientificName: Cheilotrichia (Cheilotrichia) imbuta (Meigen, 1818); family: Limoniidae; genus: Cheilotrichia; subgenus: Cheilotrichia; specificEpithet: imbuta; scientificNameAuthorship: (Meigen, 1818); **Location:** country: Russia; stateProvince: East European Russia; county: Tatarstan Respublika; municipality: Zelenodol’sk district; locality: Volga-Kama State Nature Biosphere Reserve, «Raifa», Lake Lenevo; verbatimElevation: 80 m; minimumElevationInMeters: 80; decimalLatitude: 55.90433; decimalLongitude: 48.79115; **Identification:** identifiedBy: N.M. Paramonov; **Event:** samplingProtocol: Sweep net; eventDate: 2012-06-26; verbatimEventDate: 26/Jun/2012; **Record Level:** institutionCode: ZIN; basisOfRecord: PreservedSpecimen

#### Distribution

First records from Belarus and Russia: RUE.

### Cheilotrichia (Empeda) neglecta

(Lackschewitz, 1927)

64F275F9-98EA-532F-A532-27F7AFC61B3B

https://ccw.naturalis.nl/detail.php?id=556

#### Materials

- **Type status:** Other material. **Occurrence:** occurrenceRemarks: 1 male; recordedBy: M. Andersson; individualCount: 1; sex: male; preparations: Pinned; occurrenceID: EU_LIM_051; **Taxon:** scientificName: Cheilotrichia (Empeda) neglecta (Lackschewitz, 1927); family: Limoniidae; genus: Cheilotrichia; subgenus: Empeda; specificEpithet: neglecta; scientificNameAuthorship: (Lackschewitz, 1927); **Location:** country: Sweden; stateProvince: Småland; municipality: Jönköping; locality: Älgagölen, Smedstorp, Hakarp; verbatimElevation: 100 m; minimumElevationInMeters: 100; decimalLatitude: 57.80818; decimalLongitude: 14.28735; **Identification:** identifiedBy: M. Andersson; **Event:** samplingProtocol: Sweep net; eventDate: 2020-09-22; verbatimEventDate: 22/Sep/2020; **Record Level:** institutionCode: NHRS; basisOfRecord: PreservedSpecimen
- **Type status:** Other material. **Occurrence:** occurrenceRemarks: 1 male; recordedBy: M. Andersson | M. Oomen; individualCount: 1; sex: male; preparations: Pinned; occurrenceID: EU_LIM_052; **Taxon:** scientificName: Cheilotrichia (Empeda) neglecta (Lackschewitz, 1927); family: Limoniidae; genus: Cheilotrichia; subgenus: Empeda; specificEpithet: neglecta; scientificNameAuthorship: (Lackschewitz, 1927); **Location:** country: Sweden; stateProvince: Småland; municipality: Jönköping; locality: Älgagölen, Smedstorp, Hakarp; verbatimElevation: 100 m; minimumElevationInMeters: 100; decimalLatitude: 57.80818; decimalLongitude: 14.28735; **Identification:** identifiedBy: M. Andersson; **Event:** samplingProtocol: Sweep net; eventDate: 2020-09-27; verbatimEventDate: 27/Sep/2020; **Record Level:** institutionCode: NHRS; basisOfRecord: PreservedSpecimen
- **Type status:** Other material. **Occurrence:** occurrenceRemarks: 3 males; recordedBy: The Swedish Malaise Trap Project; individualCount: 3; sex: male; preparations: Ethanol; occurrenceID: EU_LIM_053; **Taxon:** scientificName: Cheilotrichia (Empeda) neglecta (Lackschewitz, 1927); family: Limoniidae; genus: Cheilotrichia; subgenus: Empeda; specificEpithet: neglecta; scientificNameAuthorship: (Lackschewitz, 1927); **Location:** country: Sweden; stateProvince: Småland; municipality: Jönköping; locality: Lönnemålen, Gränna; verbatimElevation: 10 m; minimumElevationInMeters: 10; decimalLatitude: 58.04892; decimalLongitude: 14.57303; **Identification:** identifiedBy: M. Andersson; **Event:** samplingProtocol: Malaise trap; eventDate: 2005-08-31/2005-09-13; verbatimEventDate: 31/Aug-13/Sep/2005; **Record Level:** institutionCode: NHRS; basisOfRecord: PreservedSpecimen
- **Type status:** Other material. **Occurrence:** occurrenceRemarks: 2 males; recordedBy: The Swedish Malaise Trap Project; individualCount: 2; sex: male; preparations: Ethanol; occurrenceID: EU_LIM_054; **Taxon:** scientificName: Cheilotrichia (Empeda) neglecta (Lackschewitz, 1927); family: Limoniidae; genus: Cheilotrichia; subgenus: Empeda; specificEpithet: neglecta; scientificNameAuthorship: (Lackschewitz, 1927); **Location:** country: Sweden; stateProvince: Småland; municipality: Jönköping; locality: Lönnemålen, Gränna; verbatimElevation: 10 m; minimumElevationInMeters: 10; decimalLatitude: 58.04892; decimalLongitude: 14.57303; **Identification:** identifiedBy: M. Andersson; **Event:** samplingProtocol: Malaise trap; eventDate: 2005-09-13/2005-10-04; verbatimEventDate: 13/Sep-4/Oct/2005; **Record Level:** institutionCode: NHRS; basisOfRecord: PreservedSpecimen

#### Distribution

First record from Sweden.

### Cheilotrichia (Cheilotrichia) valai

Starý, 1992

247AFC2E-908F-5C4E-9585-BA53B68CC118

https://ccw.naturalis.nl/detail.php?id=490

#### Materials

- **Type status:** Other material. **Occurrence:** occurrenceRemarks: 5 males, 1 female; recordedBy: L.-P. Kolcsár; individualCount: 6; sex: male, female; preparations: Ethanol; occurrenceID: EU_LIM_043; **Taxon:** scientificName: Cheilotrichia (Cheilotrichia) valai Starý, 1992; family: Limoniidae; genus: Cheilotrichia; subgenus: Cheilotrichia; specificEpithet: valai; scientificNameAuthorship: Starý, 1992; **Location:** country: Romania; stateProvince: Satu Mare; municipality: Turulung; locality: Râul Tur Protected Area, Turulung-Vii; verbatimElevation: 132 m; minimumElevationInMeters: 132; decimalLatitude: 47.9194; decimalLongitude: 23.14722; **Identification:** identifiedBy: L.-P. Kolcsár; **Event:** samplingProtocol: Sweep net; eventDate: 2011-05-20; verbatimEventDate: 20/May/2011; **Record Level:** institutionCode: CKLP; basisOfRecord: PreservedSpecimen

#### Distribution

First record from Romania.

### Chionea araneoides

Dalman, 1816

EB45E8B3-4330-5ABB-81B7-CDCCFBC4F67C

https://ccw.naturalis.nl/detail.php?id=610

#### Materials

- **Type status:** Other material. **Occurrence:** occurrenceRemarks: 1 male, 3 females; recordedBy: Sh.A. Murtazin; individualCount: 4; sex: male, female; occurrenceID: EU_LIM_055; **Taxon:** scientificName: Chioneaaraneoides Dalman, 1816; family: Limoniidae; genus: Chionea; specificEpithet: araneoides; scientificNameAuthorship: Dalman, 1816; **Location:** country: Russia; stateProvince: East European Russia; county: Bashkortostan Respublika; locality: Bolshoy Iremel Mts.; verbatimElevation: 800 m; minimumElevationInMeters: 800; decimalLatitude: 54.51997; decimalLongitude: 58.794; **Identification:** identifiedBy: N.M. Paramonov; **Event:** samplingProtocol: hand picking; eventDate: 2017-09-30; verbatimEventDate: 30/Sep/2017; **Record Level:** institutionCode: ZIN; basisOfRecord: PreservedSpecimen

#### Distribution

First record from Russia: RUE.

### Crypteria (Crypteria) limnophiloides

Bergroth, 1913

803D2DFE-BEAA-5658-9313-626A6C3285B9

https://ccw.naturalis.nl/detail.php?id=703

#### Materials

- **Type status:** Other material. **Occurrence:** occurrenceRemarks: 1 male; recordedBy: K.P. Tomkovich; individualCount: 1; sex: male; occurrenceID: EU_LIM_056; **Taxon:** scientificName: Crypteria (Crypteria) limnophiloides Bergroth, 1913; family: Limoniidae; genus: Crypteria; subgenus: Crypteria; specificEpithet: limnophiloides; scientificNameAuthorship: Bergroth, 1913; **Location:** country: Russia; stateProvince: Central European Russia; county: Moskovskaya Oblast; municipality: Taldom district; locality: vill Pripuschaevo; verbatimElevation: 120 m; minimumElevationInMeters: 120; decimalLatitude: 56.70168; decimalLongitude: 37.68251; **Identification:** identifiedBy: V.E. Pilipenko; **Event:** samplingProtocol: Sweep net; eventDate: 2008-09-08/2008-09-10; verbatimEventDate: 8-10/Sep/2008; **Record Level:** institutionCode: VPMC; basisOfRecord: PreservedSpecimen
- **Type status:** Other material. **Occurrence:** occurrenceRemarks: 1 female; recordedBy: D.I. Gavryushin; individualCount: 1; sex: female; preparations: Pinned; occurrenceID: EU_LIM_057; **Taxon:** scientificName: Crypteria (Crypteria) limnophiloides Bergroth, 1913; family: Limoniidae; genus: Crypteria; subgenus: Crypteria; specificEpithet: limnophiloides; scientificNameAuthorship: Bergroth, 1913; **Location:** country: Serbia; stateProvince: Zaječar; municipality: Knjaževac; locality: Crni Vrh; verbatimElevation: 800 m; minimumElevationInMeters: 800; decimalLatitude: 43.407; decimalLongitude: 22.587; **Identification:** identifiedBy: D.I. Gavryushin; **Event:** samplingProtocol: Sweep net; eventDate: 2014-09-16/2014-09-18; verbatimEventDate: 16-22/Sep/2014; **Record Level:** institutionCode: ZMMU; basisOfRecord: PreservedSpecimen
- **Type status:** Other material. **Occurrence:** occurrenceRemarks: 1 female; recordedBy: D.I. Gavryushin; individualCount: 1; sex: female; preparations: Pinned; occurrenceID: EU_LIM_058; **Taxon:** scientificName: Crypteria (Crypteria) limnophiloides Bergroth, 1913; family: Limoniidae; genus: Crypteria; subgenus: Crypteria; specificEpithet: limnophiloides; scientificNameAuthorship: Bergroth, 1913; **Location:** country: Serbia; locality: Stara Planina Mts.; verbatimElevation: 1500 m; minimumElevationInMeters: 1500; decimalLatitude: 43.37; decimalLongitude: 22.6; **Identification:** identifiedBy: D.I. Gavryushin; **Event:** samplingProtocol: Sweep net; eventDate: 2014-09-16/2014-09-18; verbatimEventDate: 16-18/Sep/2014; **Record Level:** institutionCode: ZMMU; basisOfRecord: PreservedSpecimen

#### Distribution

First records from Russia: RUC and Serbia.

### Dactylolabis (Coenolabis) posthabita

(Bergroth, 1888)

E97AD282-3115-5C4D-954A-FA4B80FF4100

https://ccw.naturalis.nl/detail.php?id=4768

#### Materials

- **Type status:** Other material. **Occurrence:** occurrenceRemarks: 5 males; recordedBy: S. Tóth; individualCount: 5; sex: male; occurrenceID: EU_LIM_059; **Taxon:** scientificName: Dactylolabis (Coenolabis) posthabita (Bergroth, 1888); family: Limoniidae; genus: Dactylolabis; subgenus: Coenolabis; specificEpithet: posthabita; scientificNameAuthorship: (Bergroth, 1888); **Location:** country: Hungary; stateProvince: Somogy; municipality: Potony; decimalLatitude: 45.93; decimalLongitude: 17.64; **Identification:** identifiedBy: L.-P. Kolcsár; **Event:** eventDate: 1977-05-03; verbatimEventDate: 03/May/1977; **Record Level:** institutionCode: HNHM; basisOfRecord: PreservedSpecimen

#### Description

Fig. 3

#### Distribution

First records from Hungary.

### Dactylolabis (Dactylolabis) sexmaculata

(Macquart, 1826)

8354026C-B132-59C6-AC2E-9CFAE52986C4

https://ccw.naturalis.nl/detail.php?id=4817

#### Materials

- **Type status:** Other material. **Occurrence:** occurrenceRemarks: 1 male; recordedBy: D.I. Gavryushin; individualCount: 1; sex: male; preparations: Pinned; occurrenceID: EU_LIM_060; **Taxon:** scientificName: Dactylolabis (Dactylolabis) sexmaculata (Macquart, 1826); family: Limoniidae; genus: Dactylolabis; subgenus: Dactylolabis; specificEpithet: sexmaculata; scientificNameAuthorship: (Macquart, 1826); **Location:** country: Serbia; locality: Stara Planina Mts., Babin Zub Mountain; verbatimElevation: 1547 m; minimumElevationInMeters: 1547; decimalLatitude: 43.374; decimalLongitude: 22.621; **Identification:** identifiedBy: D.I. Gavryushin; **Event:** samplingProtocol: Sweep net; eventDate: 2015-06-06; verbatimEventDate: 6/Jul/2015; **Record Level:** institutionCode: ZMMU; basisOfRecord: PreservedSpecimen

#### Distribution

First record from Serbia.

Records of *Dactylolabissexmaculata* from the European part of Turkey reported by Koç et al. (2016) are misidentifications; they refer to *Dactylolabistransversa* (Meigen, 1804), based on the published wing and genital photos.

### Dactylolabis (Dactylolabis) symplectoidea

Egger, 1863

8E395515-51B2-5C5B-BFA9-76AFEBA42B47

https://ccw.naturalis.nl/detail.php?id=4822

#### Materials

- **Type status:** Other material. **Occurrence:** occurrenceRemarks: 1 male; recordedBy: J. Martinovský; individualCount: 1; sex: male; preparations: Pinned; occurrenceID: EU_LIM_061; **Taxon:** scientificName: Dactylolabis (Dactylolabis) symplectoidea Egger, 1863; family: Limoniidae; genus: Dactylolabis; subgenus: Dactylolabis; specificEpithet: symplectoidea; scientificNameAuthorship: Egger, 1863; **Location:** island: Corfu; country: Greece; stateProvince: Ionian Islands; municipality: Mparmpati; locality: Barbati env.; decimalLatitude: 39.713; decimalLongitude: 19.857; **Identification:** identifiedBy: J. Starý; **Event:** eventDate: 1993-05-02; verbatimEventDate: May-02-1993; **Record Level:** institutionCode: PCJS; basisOfRecord: PreservedSpecimen
- **Type status:** Other material. **Occurrence:** occurrenceRemarks: 3 males, 1 female; recordedBy: J. Starý; individualCount: 4; sex: male, female; preparations: Pinned; occurrenceID: EU_LIM_062; **Taxon:** scientificName: Dactylolabis (Dactylolabis) symplectoidea Egger, 1863; family: Limoniidae; genus: Dactylolabis; subgenus: Dactylolabis; specificEpithet: symplectoidea; scientificNameAuthorship: Egger, 1863; **Location:** country: Greece; stateProvince: Pieria; municipality: Prionia; locality: Olympos Mts.; verbatimElevation: 1000-1200 m; minimumElevationInMeters: 1000; maximumElevationInMeters: 1200; decimalLatitude: 40.079; decimalLongitude: 22.393; **Identification:** identifiedBy: J. Starý; **Event:** eventDate: 2007-06-06; verbatimEventDate: 6/Jun/2007; **Record Level:** institutionCode: PCJS; basisOfRecord: PreservedSpecimen

#### Distribution

First records from Greece (from Corfu and mainland).

### Dicranomyia (Dicranomyia) affinis

(Schummel, 1829)

DB5825A5-FDA9-5C7C-BEA9-8CA6E8436E8D

https://ccw.naturalis.nl/detail.php?id=8018

#### Materials

- **Type status:** Other material. **Occurrence:** occurrenceRemarks: 2 males, 1 female; recordedBy: Canopy project; individualCount: 3; sex: male, female; preparations: Ethanol; occurrenceID: EU_LIM_063; **Taxon:** scientificName: Dicranomyia (Dicranomyia) affinis (Schummel, 1829); family: Limoniidae; genus: Dicranomyia; subgenus: Dicranomyia; specificEpithet: affinis; scientificNameAuthorship: (Schummel, 1829); **Location:** country: France; municipality: Saint Rémy en Rollat; locality: Forêt de Marcenat; verbatimElevation: 318 m; minimumElevationInMeters: 318; decimalLatitude: 46.2064; decimalLongitude: 3.35888; **Identification:** identifiedBy: C. Quindroit; **Event:** samplingProtocol: Lindgren trap; eventDate: 2019-06-06; verbatimEventDate: 06/Jun/2019; **Record Level:** institutionCode: PCCQ; basisOfRecord: PreservedSpecimen
- **Type status:** Other material. **Occurrence:** occurrenceRemarks: 2 males; recordedBy: Canopy project; individualCount: 2; sex: male; preparations: Ethanol; occurrenceID: EU_LIM_064; **Taxon:** scientificName: Dicranomyia (Dicranomyia) affinis (Schummel, 1829); family: Limoniidae; genus: Dicranomyia; subgenus: Dicranomyia; specificEpithet: affinis; scientificNameAuthorship: (Schummel, 1829); **Location:** country: France; municipality: Vouzeron; locality: Forêt de Vouzeron; verbatimElevation: 170 m; minimumElevationInMeters: 170; decimalLatitude: 47.26713; decimalLongitude: 2.19792; **Identification:** identifiedBy: C. Quindroit; **Event:** samplingProtocol: Lindgren trap; eventDate: 2019-05-07; verbatimEventDate: 07/May/2019; **Record Level:** institutionCode: PCCQ; basisOfRecord: PreservedSpecimen
- **Type status:** Other material. **Occurrence:** occurrenceRemarks: 27 males, 16 females; recordedBy: Canopy project; individualCount: 43; sex: male; preparations: Ethanol; occurrenceID: EU_LIM_065; **Taxon:** scientificName: Dicranomyia (Dicranomyia) affinis (Schummel, 1829); family: Limoniidae; genus: Dicranomyia; subgenus: Dicranomyia; specificEpithet: affinis; scientificNameAuthorship: (Schummel, 1829); **Location:** country: France; municipality: Loury; locality: Forêt d'Orléans; verbatimElevation: 138 m; minimumElevationInMeters: 138; decimalLatitude: 48.039; decimalLongitude: 2.18181; **Identification:** identifiedBy: C. Quindroit; **Event:** samplingProtocol: Lindgren trap; eventDate: 2019-05-07; verbatimEventDate: 07/May/2019; **Record Level:** institutionCode: PCCQ; basisOfRecord: PreservedSpecimen
- **Type status:** Other material. **Occurrence:** catalogNumber: 535222, 644160; occurrenceRemarks: 3 male+female; recordedBy: K. Berggren; individualCount: 3; sex: male, female; preparations: Ethanol; occurrenceID: EU_LIM_066; **Taxon:** scientificName: Dicranomyia (Dicranomyia) affinis (Schummel, 1829); family: Limoniidae; genus: Dicranomyia; subgenus: Dicranomyia; specificEpithet: affinis; scientificNameAuthorship: (Schummel, 1829); **Location:** country: Norway; stateProvince: Aust-Agder; municipality: Grimstad; locality: Søm (Skogstuen); verbatimElevation: 10 m; minimumElevationInMeters: 10; decimalLatitude: 58.3896; decimalLongitude: 8.71272; **Identification:** identifiedBy: K.M. Olsen; **Event:** samplingProtocol: Light trap; eventDate: 2017-06; verbatimEventDate: Jun/2017; habitat: I bøkeskogen; **Record Level:** institutionCode: PCKMO; basisOfRecord: PreservedSpecimen
- **Type status:** Other material. **Occurrence:** catalogNumber: 531880; occurrenceRemarks: 1 male; recordedBy: K. Berggren; individualCount: 1; sex: male; preparations: Ethanol; occurrenceID: EU_LIM_067; **Taxon:** scientificName: Dicranomyia (Dicranomyia) affinis (Schummel, 1829); family: Limoniidae; genus: Dicranomyia; subgenus: Dicranomyia; specificEpithet: affinis; scientificNameAuthorship: (Schummel, 1829); **Location:** country: Norway; stateProvince: Vest-Agder; municipality: Kristiansand; locality: Nedre Timenes; verbatimElevation: 10 m; minimumElevationInMeters: 10; decimalLatitude: 58.16155; decimalLongitude: 8.10013; **Identification:** identifiedBy: J. Salmela; **Event:** samplingProtocol: Light trap; eventDate: 2017-06-01/2017-06-19; verbatimEventDate: 01-19/Jun/2017; **Record Level:** institutionCode: PCKMO; basisOfRecord: PreservedSpecimen
- **Type status:** Other material. **Occurrence:** occurrenceRemarks: 1 male; recordedBy: E. Eiroa; individualCount: 1; sex: male; preparations: Pinned; occurrenceID: EU_LIM_068; **Taxon:** scientificName: Dicranomyia (Dicranomyia) affinis (Schummel, 1829); family: Limoniidae; genus: Dicranomyia; subgenus: Dicranomyia; specificEpithet: affinis; scientificNameAuthorship: (Schummel, 1829); **Location:** country: Spain; stateProvince: Castilla y León, León; municipality: Candín; locality: Candín; verbatimElevation: 885 m; minimumElevationInMeters: 885; decimalLatitude: 42.81674; decimalLongitude: -6.72645; **Identification:** identifiedBy: E. Eiroa; **Event:** samplingProtocol: Sweep net; eventDate: 1984-05-09; verbatimEventDate: 9/May/1984; habitat: meadow; **Record Level:** institutionCode: USC; basisOfRecord: PreservedSpecimen
- **Type status:** Other material. **Occurrence:** occurrenceRemarks: 1 male + 1 female; recordedBy: D. Nyström; individualCount: 2; sex: male, female; preparations: Pinned; occurrenceID: EU_LIM_069; **Taxon:** scientificName: Dicranomyia (Dicranomyia) affinis (Schummel, 1829); family: Limoniidae; genus: Dicranomyia; subgenus: Dicranomyia; specificEpithet: affinis; scientificNameAuthorship: (Schummel, 1829); **Location:** country: Sweden; stateProvince: Gotland; municipality: Gotland; locality: Själsöån Nature Reserve, Väskinde; verbatimElevation: 25 m; minimumElevationInMeters: 25; decimalLatitude: 57.69364; decimalLongitude: 18.35533; **Identification:** identifiedBy: M. Andersson; **Event:** samplingProtocol: Sweep net; eventDate: 2020-05-16; verbatimEventDate: 16/May/2020; **Record Level:** institutionCode: NHRS; basisOfRecord: PreservedSpecimen
- **Type status:** Other material. **Occurrence:** occurrenceRemarks: 11 males + 12 females; recordedBy: M. Andersson | R. Isaksson | M. Karlsson; individualCount: 23; sex: male; preparations: Pinned; occurrenceID: EU_LIM_070; **Taxon:** scientificName: Dicranomyia (Dicranomyia) affinis (Schummel, 1829); family: Limoniidae; genus: Dicranomyia; subgenus: Dicranomyia; specificEpithet: affinis; scientificNameAuthorship: (Schummel, 1829); **Location:** country: Sweden; stateProvince: Öland; municipality: Mörbylånga; locality: Albrunna; verbatimElevation: 500 m; minimumElevationInMeters: 500; decimalLatitude: 56.33362; decimalLongitude: 16.42606; **Identification:** identifiedBy: M. Andersson; **Event:** samplingProtocol: Sweep net; eventDate: 2019-05-31; verbatimEventDate: 31/May/2019; **Record Level:** institutionCode: NHRS | MZLU; basisOfRecord: PreservedSpecimen
- **Type status:** Other material. **Occurrence:** occurrenceRemarks: 4 males; recordedBy: R. Isaksson; individualCount: 4; sex: male; preparations: Pinned; occurrenceID: EU_LIM_071; **Taxon:** scientificName: Dicranomyia (Dicranomyia) affinis (Schummel, 1829); family: Limoniidae; genus: Dicranomyia; subgenus: Dicranomyia; specificEpithet: affinis; scientificNameAuthorship: (Schummel, 1829); **Location:** country: Sweden; stateProvince: Öland; municipality: Mörbylånga; locality: Eketorp; verbatimElevation: 25 m; minimumElevationInMeters: 25; decimalLatitude: 56.29902; decimalLongitude: 16.47221; **Identification:** identifiedBy: M. Andersson; **Event:** samplingProtocol: Sweep net; eventDate: 2019-05-31; verbatimEventDate: 31/May/2019; **Record Level:** institutionCode: NHRS; basisOfRecord: PreservedSpecimen

#### Distribution

First records from France (from mainland), Norway, Spain (from mainland) and Sweden.

### Dicranomyia (Idiopyga) alpina

Bangerter, 1948

3708C328-1E09-52A8-9789-F496D9C87659

https://ccw.naturalis.nl/detail.php?id=8937

#### Materials

- **Type status:** Other material. **Occurrence:** occurrenceRemarks: 2 males, 1 female; recordedBy: L.-P. Kolcsár | E. Török; individualCount: 3; sex: male, female; preparations: Ethanol; occurrenceID: EU_LIM_189; **Taxon:** scientificName: Dicranomyia (Idiopyga) alpina Bangerter, 1948; family: Limoniidae; genus: Dicranomyia; subgenus: Idiopyga; specificEpithet: alpina; scientificNameAuthorship: Bangerter, 1948; **Location:** country: Romania; stateProvince: Harghita; municipality: Toplița; locality: Călimani Mts., Lomaș Valley; verbatimElevation: 1210 m; minimumElevationInMeters: 1210; decimalLatitude: 47.06016; decimalLongitude: 25.29841; **Identification:** identifiedBy: L.-P. Kolcsár; **Event:** samplingProtocol: Sweep net; eventDate: 2011-09-05; verbatimEventDate: 5/Sep/2011; **Record Level:** institutionCode: CKLP; basisOfRecord: PreservedSpecimen
- **Type status:** Other material. **Occurrence:** occurrenceRemarks: 1 male, 1 female; recordedBy: L.-P. Kolcsár | E. Török; individualCount: 2; sex: male, female; preparations: Ethanol; occurrenceID: EU_LIM_190; **Taxon:** scientificName: Dicranomyia (Idiopyga) alpina Bangerter, 1948; family: Limoniidae; genus: Dicranomyia; subgenus: Idiopyga; specificEpithet: alpina; scientificNameAuthorship: Bangerter, 1948; **Location:** country: Romania; stateProvince: Harghita; municipality: Toplița; locality: Călimani Mts., Iezer Lake; verbatimElevation: 1740 m; minimumElevationInMeters: 1740; decimalLatitude: 47.09359; decimalLongitude: 25.26134; **Identification:** identifiedBy: L.-P. Kolcsár; **Event:** samplingProtocol: Sweep net; eventDate: 2011-09-05; verbatimEventDate: 5/Sep/2011; **Record Level:** institutionCode: CKLP; basisOfRecord: PreservedSpecimen

#### Distribution

First records from Romania.

### Dicranomyia (Dicranomyia) aperta

Wahlgren, 1904

854526CE-4EC0-53E4-8BA2-A45CC62704DC

https://ccw.naturalis.nl/detail.php?id=8041

#### Materials

- **Type status:** Other material. **Occurrence:** catalogNumber: 534926, 644161; occurrenceRemarks: 8 male+female; recordedBy: K.M. Olsen; individualCount: 8; sex: male, female; preparations: Ethanol; occurrenceID: EU_LIM_072; **Taxon:** scientificName: Dicranomyia (Dicranomyia) aperta Wahlgren, 1904; family: Limoniidae; genus: Dicranomyia; subgenus: Dicranomyia; specificEpithet: aperta; scientificNameAuthorship: Wahlgren, 1904; **Location:** country: Norway; stateProvince: Aust-Agder; municipality: Arendal; locality: Flangeborgkilen; verbatimElevation: 0.5 m; minimumElevationInMeters: 0.5; decimalLatitude: 58.44768; decimalLongitude: 8.82698; **Identification:** identifiedBy: K.M. Olsen; **Event:** samplingProtocol: Sweep net; eventDate: 2017-08-02; verbatimEventDate: 02/Aug/2017; habitat: Strandengene; **Record Level:** institutionCode: PCKMO; basisOfRecord: PreservedSpecimen

#### Distribution

First record from Norway.

### Dicranomyia (Sivalimnobia) aquosa

Verrall, 1886

4C0BA7C0-D4CD-5E4C-A6C4-9EF6A4E747B7

https://ccw.naturalis.nl/detail.php?id=9171

#### Materials

- **Type status:** Other material. **Occurrence:** occurrenceRemarks: 4 males; recordedBy: D. Murányi; individualCount: 4; sex: male; preparations: Pinned; occurrenceID: EU_LIM_251; **Taxon:** scientificName: Dicranomyia (Sivalimnobia) aquosa Verrall, 1886; family: Limoniidae; genus: Dicranomyia; subgenus: Sivalimnobia; specificEpithet: aquosa; scientificNameAuthorship: Verrall, 1886; **Location:** country: Romania; stateProvince: Maramureș; municipality: Săpânţa; locality: Igniş Mts.; verbatimElevation: 408; minimumElevationInMeters: 408; decimalLatitude: 47.93486; decimalLongitude: 23.67811; **Identification:** identifiedBy: L.-P. Kolcsár; **Event:** samplingProtocol: Sweep net; eventDate: 2005-06-30; verbatimEventDate: 30/Jun/2005; habitat: mineral water springs and their outflows in a beech forest; **Record Level:** institutionCode: HNHM; basisOfRecord: PreservedSpecimen

#### Distribution

First record from Romania.

### Dicranomyia (Dicranomyia) autumnalis

(Staeger, 1840)

4C655356-9D44-50F8-871F-16AF9C115136

https://ccw.naturalis.nl/detail.php?id=8062

#### Materials

- **Type status:** Other material. **Occurrence:** occurrenceRemarks: 1 male; recordedBy: D.I. Gavryushin; individualCount: 1; sex: male; preparations: Pinned; occurrenceID: EU_LIM_073; **Taxon:** scientificName: Dicranomyia (Dicranomyia) autumnalis (Staeger, 1840); family: Limoniidae; genus: Dicranomyia; subgenus: Dicranomyia; specificEpithet: autumnalis; scientificNameAuthorship: (Staeger, 1840); **Location:** country: Serbia; locality: Stara Planina Mts.; verbatimElevation: 1500 m; minimumElevationInMeters: 1500; decimalLatitude: 43.37; decimalLongitude: 22.6; **Identification:** identifiedBy: D.I. Gavryushin; **Event:** samplingProtocol: Sweep net; eventDate: 2014-09-16/2014-09-18; verbatimEventDate: 16-18/Sep/2014; **Record Level:** institutionCode: ZMMU; basisOfRecord: PreservedSpecimen

#### Distribution

First record from Serbia.

### Dicranomyia (Melanolimonia) caledonica

Edwards, 1926

46C13691-AD6F-5052-AA50-5E0C537AF646

https://ccw.naturalis.nl/detail.php?id=8978

#### Materials

- **Type status:** Other material. **Occurrence:** occurrenceRemarks: 2 males; recordedBy: L.-P. Kolcsár | E. Török; individualCount: 2; sex: male; preparations: Ethanol; occurrenceID: EU_LIM_223; **Taxon:** scientificName: Dicranomyia (Melanolimonia) caledonica Edwards, 1926; family: Limoniidae; genus: Dicranomyia; subgenus: Melanolimonia; specificEpithet: caledonica; scientificNameAuthorship: Edwards, 1926; **Location:** country: Serbia; municipality: Kopaonik; locality: Kopaonik Mts.; verbatimElevation: 1556 m; minimumElevationInMeters: 1556; decimalLatitude: 43.0997; decimalLongitude: 20.76563; **Identification:** identifiedBy: L.-P. Kolcsár; **Event:** samplingProtocol: Sweep net; eventDate: 2017-06-22; verbatimEventDate: 22/Jun/2017; **Record Level:** institutionCode: CKLP; basisOfRecord: PreservedSpecimen

#### Distribution

First record from Serbia.

### Dicranomyia (Dicranomyia) chorea

(Meigen, 1818)

4CC089C9-8CEA-5B78-98E7-E799D446B1A3

https://ccw.naturalis.nl/detail.php?id=8127

#### Materials

- **Type status:** Other material. **Occurrence:** occurrenceRemarks: 1 male; recordedBy: D.I. Gavryushin; individualCount: 1; sex: male; occurrenceID: EU_LIM_074; **Taxon:** scientificName: Dicranomyia (Dicranomyia) chorea (Meigen, 1818); family: Limoniidae; genus: Dicranomyia; subgenus: Dicranomyia; specificEpithet: chorea; scientificNameAuthorship: (Meigen, 1818); **Location:** country: Russia; stateProvince: East European Russia; county: Bashkortostan Respublika; municipality: Beloretsk district; locality: Mata River; verbatimElevation: 552; minimumElevationInMeters: 552; decimalLatitude: 54.00438; decimalLongitude: 58.46398; **Identification:** identifiedBy: D.I. Gavryushin; **Event:** samplingProtocol: Sweep net; eventDate: 2012-08-07; verbatimEventDate: Aug-07-2012; **Record Level:** institutionCode: ZMMU; basisOfRecord: PreservedSpecimen

#### Distribution

First record from Russia: RUE.

### Dicranomyia (Dicranomyia) conchifera

(Strobl, 1900)

F526C1D9-F35F-582C-8D0E-731E2E146D6B

https://ccw.naturalis.nl/detail.php?id=8152

#### Materials

- **Type status:** Other material. **Occurrence:** occurrenceRemarks: 1 male; recordedBy: D.I. Gavryushin; individualCount: 1; sex: male; preparations: Pinned; occurrenceID: EU_LIM_075; **Taxon:** scientificName: Dicranomyia (Dicranomyia) conchifera (Strobl, 1900); family: Limoniidae; genus: Dicranomyia; subgenus: Dicranomyia; specificEpithet: conchifera; scientificNameAuthorship: (Strobl, 1900); **Location:** country: Serbia; stateProvince: Zaječar; municipality: Knjaževac; locality: Crni Vrh; verbatimElevation: 800 m; minimumElevationInMeters: 800; decimalLatitude: 43.407; decimalLongitude: 22.587; **Identification:** identifiedBy: D.I. Gavryushin; **Event:** samplingProtocol: Sweep net; eventDate: 2015-06-01/2015-07-07; verbatimEventDate: 01-07/Jul/2015; **Record Level:** institutionCode: ZMMU; basisOfRecord: PreservedSpecimen

#### Distribution

First record from Serbia.

### Dicranomyia (Dicranomyia) consimilis

(Zetterstedt, 1838)

A2245042-EA2F-5D86-A1F8-D53C2B11A0FC

https://ccw.naturalis.nl/detail.php?id=8156

#### Materials

- **Type status:** Other material. **Occurrence:** occurrenceRemarks: 1 male, 1 female; recordedBy: G.B. Delmastro; individualCount: 2; sex: male, female; preparations: Pinned; occurrenceID: EU_LIM_076; **Taxon:** scientificName: Dicranomyia (Dicranomyia) consimilis (Zetterstedt, 1838); family: Limoniidae; genus: Dicranomyia; subgenus: Dicranomyia; specificEpithet: consimilis; scientificNameAuthorship: (Zetterstedt, 1838); **Location:** country: Italy; stateProvince: Piedmont; municipality: Trecate; locality: Fascia fluviale dei Ticino, loc. Ristol, La Chioccida; verbatimElevation: 110 m; minimumElevationInMeters: 110; decimalLatitude: 45.453; decimalLongitude: 8.797; **Identification:** identifiedBy: J. Starý; **Event:** eventDate: 2004-09-07; verbatimEventDate: Sep-07-2004; **Record Level:** institutionCode: PCJS; basisOfRecord: PreservedSpecimen
- **Type status:** Other material. **Occurrence:** occurrenceRemarks: 1 male; recordedBy: D.I. Gavryushin; individualCount: 1; sex: male; occurrenceID: EU_LIM_077; **Taxon:** scientificName: Dicranomyia (Dicranomyia) consimilis (Zetterstedt, 1838); family: Limoniidae; genus: Dicranomyia; subgenus: Dicranomyia; specificEpithet: consimilis; scientificNameAuthorship: (Zetterstedt, 1838); **Location:** country: Russia; stateProvince: East European Russia; county: Bashkortostan Respublika; municipality: Uchaly district; locality: Ural-Tau St. env., upper reaches of Mindyak River; verbatimElevation: 765; minimumElevationInMeters: 765; decimalLatitude: 53.96525; decimalLongitude: 58.57888; **Identification:** identifiedBy: D.I. Gavryushin; **Event:** samplingProtocol: Sweep net; eventDate: 2015-06-09; verbatimEventDate: 09/Jul/2015; **Record Level:** institutionCode: ZMMU; basisOfRecord: PreservedSpecimen
- **Type status:** Other material. **Occurrence:** occurrenceRemarks: 1 male; recordedBy: D.I. Gavryushin; individualCount: 1; sex: male; occurrenceID: EU_LIM_078; **Taxon:** scientificName: Dicranomyia (Dicranomyia) consimilis (Zetterstedt, 1838); family: Limoniidae; genus: Dicranomyia; subgenus: Dicranomyia; specificEpithet: consimilis; scientificNameAuthorship: (Zetterstedt, 1838); **Location:** country: Russia; stateProvince: East European Russia; county: Bashkortostan Respublika; municipality: Beloretsk district; locality: Nura River (ca. 4km W of Otnurok village), at the foot of Zolotyie Shishki (Golden Cones) Mts.; verbatimElevation: 607; minimumElevationInMeters: 607; decimalLatitude: 54.05155; decimalLongitude: 58.26887; **Identification:** identifiedBy: D.I. Gavryushin; **Event:** samplingProtocol: Sweep net; eventDate: 2012-08-11; verbatimEventDate: 11/Aug/2012; **Record Level:** institutionCode: ZMMU; basisOfRecord: PreservedSpecimen
- **Type status:** Other material. **Occurrence:** occurrenceRemarks: 2 males; recordedBy: N.M. Paramonov; individualCount: 2; sex: male; occurrenceID: EU_LIM_079; **Taxon:** scientificName: Dicranomyia (Dicranomyia) consimilis (Zetterstedt, 1838); family: Limoniidae; genus: Dicranomyia; subgenus: Dicranomyia; specificEpithet: consimilis; scientificNameAuthorship: (Zetterstedt, 1838); **Location:** country: Russia; stateProvince: East European Russia; county: Tatarstan Respublika; municipality: Zelenodol’sk district; locality: Volga-Kama State Nature Biosphere Reserve, «Raifa», Sumka River, Belo-Bezvodnoe village env.; verbatimElevation: 75 m; minimumElevationInMeters: 75; decimalLatitude: 55.92183; decimalLongitude: 48.75462; **Identification:** identifiedBy: N.M. Paramonov; **Event:** samplingProtocol: Sweep net; eventDate: 2012-06-28; verbatimEventDate: 28/Jun/2012; **Record Level:** institutionCode: ZIN; basisOfRecord: PreservedSpecimen

#### Distribution

First records from Italy (from mainland) and Russia: RUE.

### Dicranomyia (Idiopyga) ctenopyga

(Alexander, 1943)

1CD28057-C970-5077-B652-40B5EBD53D10

https://ccw.naturalis.nl/detail.php?id=8939

#### Materials

- **Type status:** Other material. **Occurrence:** occurrenceRemarks: 1 male; recordedBy: N.M. Paramonov; individualCount: 1; sex: male; occurrenceID: EU_LIM_191; **Taxon:** scientificName: Dicranomyia (Idiopyga) ctenopyga (Alexander, 1943); family: Limoniidae; genus: Dicranomyia; subgenus: Idiopyga; specificEpithet: ctenopyga; scientificNameAuthorship: (Alexander, 1943); **Location:** country: Russia; stateProvince: East European Russia; county: Tatarstan Respublika; municipality: Kazan; locality: district Derbyshki, Noksa River; verbatimElevation: 60 m; minimumElevationInMeters: 60; decimalLatitude: 55.8655; decimalLongitude: 49.22461; **Identification:** identifiedBy: N.M. Paramonov; **Event:** samplingProtocol: Sweep net; eventDate: 2010-06-17; verbatimEventDate: Jun-17-2010; **Record Level:** institutionCode: ZIN; basisOfRecord: PreservedSpecimen

#### Distribution

First records from Russia: RUE.

### Dicranomyia (Idiopyga) danica

Kuntze, 1919

C19BB125-8101-5EBA-8C94-54A3835613EE

https://ccw.naturalis.nl/detail.php?id=8940

#### Materials

- **Type status:** Other material. **Occurrence:** occurrenceRemarks: 1 male; recordedBy: D.I. Gavryushin; individualCount: 1; sex: male; preparations: Pinned; occurrenceID: EU_LIM_192; **Taxon:** scientificName: Dicranomyia (Idiopyga) danica Kuntze, 1919; family: Limoniidae; genus: Dicranomyia; subgenus: Idiopyga; specificEpithet: danica; scientificNameAuthorship: Kuntze, 1919; **Location:** country: Belarus; stateProvince: Gomel; county: Mazyr; locality: Mazyr; decimalLatitude: 52.02; decimalLongitude: 29.32; **Identification:** identifiedBy: D.I. Gavryushin; **Event:** samplingProtocol: Sweep net; eventDate: 2019-05-19/2019-05-21; verbatimEventDate: 19-21/May/2019; **Record Level:** institutionCode: ZMMU; basisOfRecord: PreservedSpecimen

#### Distribution

First record from Belarus.

### Dicranomyia (Dicranomyia) didyma

(Meigen, 1804)

1B707462-A380-5402-80E3-ED3BE75297C8

https://ccw.naturalis.nl/detail.php?id=8181

#### Materials

- **Type status:** Other material. **Occurrence:** occurrenceRemarks: 1 male; recordedBy: I.V. Lyubvina; individualCount: 1; sex: male; occurrenceID: EU_LIM_080; **Taxon:** scientificName: Dicranomyia (Dicranomyia) didyma (Meigen, 1804); family: Limoniidae; genus: Dicranomyia; subgenus: Dicranomyia; specificEpithet: didyma; scientificNameAuthorship: (Meigen, 1804); **Location:** country: Russia; stateProvince: East European Russia; county: Samarskaya Oblast; municipality: Kamyshlinskiy district; locality: Krasnyy Yar village env., Karmalaka River; verbatimElevation: 30 m; minimumElevationInMeters: 30; decimalLatitude: 53.50088; decimalLongitude: 50.37471; **Identification:** identifiedBy: N.M. Paramonov; **Event:** samplingProtocol: Sweep net; eventDate: 1996-06-15/1996-07-17; verbatimEventDate: 15-17/Jul/1996; **Record Level:** institutionCode: ZIN; basisOfRecord: PreservedSpecimen

#### Distribution

First record from Russia: RUE.

### Dicranomyia (Dicranomyia) distendens distendens

Lundstrom, 1912

A65729C2-D697-5A46-A6A9-4CFFFC5F9C9F

https://ccw.naturalis.nl/detail.php?id=8188

#### Materials

- **Type status:** Other material. **Occurrence:** occurrenceRemarks: 1 male; recordedBy: K.P. Tomkovich; individualCount: 1; sex: male; occurrenceID: EU_LIM_081; **Taxon:** scientificName: Dicranomyia (Dicranomyia) distendens distendens Lundstrom, 1912; family: Limoniidae; genus: Dicranomyia; subgenus: Dicranomyia; specificEpithet: distendens; infraspecificEpithet: distendens; scientificNameAuthorship: Lundstrom, 1912; **Location:** country: Russia; stateProvince: Central European Russia; county: Moskovskaya Oblast; municipality: Orechovo-Zuevo district; locality: Antsiferovo; verbatimElevation: 145 m; minimumElevationInMeters: 145; decimalLatitude: 55.58219; decimalLongitude: 38.74287; **Identification:** identifiedBy: V.E. Pilipenko; **Event:** samplingProtocol: Sweep net; eventDate: 2000-10-04; verbatimEventDate: 04/Oct/2000; **Record Level:** institutionCode: VPMC; basisOfRecord: PreservedSpecimen
- **Type status:** Other material. **Occurrence:** occurrenceRemarks: 1 female; recordedBy: V.E. Pilipenko; individualCount: 1; sex: female; occurrenceID: EU_LIM_082; **Taxon:** scientificName: Dicranomyia (Dicranomyia) distendens distendens Lundstrom, 1912; family: Limoniidae; genus: Dicranomyia; subgenus: Dicranomyia; specificEpithet: distendens; infraspecificEpithet: distendens; scientificNameAuthorship: Lundstrom, 1912; **Location:** country: Russia; stateProvince: Central European Russia; county: Moskovskaya Oblast; municipality: Solnechnogorsk district; locality: Alabushevo; verbatimElevation: 220 m; minimumElevationInMeters: 220; decimalLatitude: 55.99676; decimalLongitude: 37.14919; **Identification:** identifiedBy: V.E. Pilipenko; **Event:** samplingProtocol: Sweep net; eventDate: 1992-06-26; verbatimEventDate: 26/Jun/1992; **Record Level:** institutionCode: VPMC; basisOfRecord: PreservedSpecimen
- **Type status:** Other material. **Occurrence:** occurrenceRemarks: 1 male; recordedBy: V.E. Pilipenko; individualCount: 1; sex: male; occurrenceID: EU_LIM_083; **Taxon:** scientificName: Dicranomyia (Dicranomyia) distendens distendens Lundstrom, 1912; family: Limoniidae; genus: Dicranomyia; subgenus: Dicranomyia; specificEpithet: distendens; infraspecificEpithet: distendens; scientificNameAuthorship: Lundstrom, 1912; **Location:** country: Russia; stateProvince: Central European Russia; county: Moskovskaya Oblast; municipality: Solnechnogorsk district; locality: Chashnikovo; verbatimElevation: 220 m; minimumElevationInMeters: 220; decimalLatitude: 56.0375; decimalLongitude: 37.1874; **Identification:** identifiedBy: V.E. Pilipenko; **Event:** samplingProtocol: Sweep net; eventDate: 1993-06-23; verbatimEventDate: 23/Jun/1993; **Record Level:** institutionCode: VPMC; basisOfRecord: PreservedSpecimen
- **Type status:** Other material. **Occurrence:** occurrenceRemarks: 1 male; recordedBy: V.E. Pilipenko; individualCount: 1; sex: male; occurrenceID: EU_LIM_084; **Taxon:** scientificName: Dicranomyia (Dicranomyia) distendens distendens Lundstrom, 1912; family: Limoniidae; genus: Dicranomyia; subgenus: Dicranomyia; specificEpithet: distendens; infraspecificEpithet: distendens; scientificNameAuthorship: Lundstrom, 1912; **Location:** country: Russia; stateProvince: Central European Russia; county: Moskovskaya Oblast; municipality: Solnechnogorsk district; locality: Chashnikovo; verbatimElevation: 220 m; minimumElevationInMeters: 220; decimalLatitude: 56.0375; decimalLongitude: 37.1874; **Identification:** identifiedBy: V.E. Pilipenko; **Event:** samplingProtocol: Sweep net; eventDate: 1993-06-27; verbatimEventDate: 27/Jul/1993; **Record Level:** institutionCode: VPMC; basisOfRecord: PreservedSpecimen
- **Type status:** Other material. **Occurrence:** occurrenceRemarks: 1 male; recordedBy: V.E. Pilipenko; individualCount: 1; sex: male; occurrenceID: EU_LIM_085; **Taxon:** scientificName: Dicranomyia (Dicranomyia) distendens distendens Lundstrom, 1912; family: Limoniidae; genus: Dicranomyia; subgenus: Dicranomyia; specificEpithet: distendens; infraspecificEpithet: distendens; scientificNameAuthorship: Lundstrom, 1912; **Location:** country: Russia; stateProvince: Central European Russia; county: Moskovskaya Oblast; municipality: Solnechnogorsk district; locality: Chashnikovo; verbatimElevation: 220 m; minimumElevationInMeters: 220; decimalLatitude: 56.0375; decimalLongitude: 37.1874; **Identification:** identifiedBy: V.E. Pilipenko; **Event:** samplingProtocol: Sweep net; eventDate: 1995-06-27; verbatimEventDate: 27/Jun/1995; **Record Level:** institutionCode: VPMC; basisOfRecord: PreservedSpecimen
- **Type status:** Other material. **Occurrence:** occurrenceRemarks: 1 female; recordedBy: V.E. Pilipenko; individualCount: 1; sex: female; occurrenceID: EU_LIM_086; **Taxon:** scientificName: Dicranomyia (Dicranomyia) distendens distendens Lundstrom, 1912; family: Limoniidae; genus: Dicranomyia; subgenus: Dicranomyia; specificEpithet: distendens; infraspecificEpithet: distendens; scientificNameAuthorship: Lundstrom, 1912; **Location:** country: Russia; stateProvince: Central European Russia; county: Moskovskaya Oblast; municipality: Solnechnogorsk district; locality: Chashnikovo; verbatimElevation: 220 m; minimumElevationInMeters: 220; decimalLatitude: 56.0375; decimalLongitude: 37.1874; **Identification:** identifiedBy: V.E. Pilipenko; **Event:** samplingProtocol: Sweep net; eventDate: 1993-10-13; verbatimEventDate: 13/Oct/1993; **Record Level:** institutionCode: VPMC; basisOfRecord: PreservedSpecimen

#### Distribution

First records from Russia: RUC.

### Dicranomyia (Dicranomyia) frontalis

(Staeger, 1840)

83A400EF-E333-5935-82C9-B75748F1BC9E

https://ccw.naturalis.nl/detail.php?id=8248

#### Materials

- **Type status:** Other material. **Occurrence:** occurrenceRemarks: 1 male; recordedBy: D.I. Gavryushin; individualCount: 1; sex: male; preparations: Pinned; occurrenceID: EU_LIM_087; **Taxon:** scientificName: Dicranomyia (Dicranomyia) frontalis (Staeger, 1840); family: Limoniidae; genus: Dicranomyia; subgenus: Dicranomyia; specificEpithet: frontalis; scientificNameAuthorship: (Staeger, 1840); **Location:** country: Belarus; stateProvince: Minsk; county: Barysaw; locality: Glivin; verbatimElevation: 161 m; minimumElevationInMeters: 161; decimalLatitude: 54.14902; decimalLongitude: 28.63648; **Identification:** identifiedBy: D.I. Gavryushin; **Event:** samplingProtocol: Sweep net; eventDate: 2013-06-06; verbatimEventDate: Jul-06-2013; **Record Level:** institutionCode: ZMMU; basisOfRecord: PreservedSpecimen
- **Type status:** Other material. **Occurrence:** occurrenceRemarks: 2 males; recordedBy: D.I. Gavryushin; individualCount: 2; sex: male; preparations: Pinned; occurrenceID: EU_LIM_088; **Taxon:** scientificName: Dicranomyia (Dicranomyia) frontalis (Staeger, 1840); family: Limoniidae; genus: Dicranomyia; subgenus: Dicranomyia; specificEpithet: frontalis; scientificNameAuthorship: (Staeger, 1840); **Location:** country: Belarus; stateProvince: Gomel; county: Mazyr; locality: Mazyr; decimalLatitude: 52.02; decimalLongitude: 29.32; **Identification:** identifiedBy: D.I. Gavryushin; **Event:** samplingProtocol: Sweep net; eventDate: 2019-05-19/2019-05-21; verbatimEventDate: 19-21/May/2019; **Record Level:** institutionCode: ZMMU; basisOfRecord: PreservedSpecimen
- **Type status:** Other material. **Occurrence:** occurrenceRemarks: 1 female; recordedBy: D.I. Gavryushin; individualCount: 1; sex: female; preparations: Pinned; occurrenceID: EU_LIM_089; **Taxon:** scientificName: Dicranomyia (Dicranomyia) frontalis (Staeger, 1840); family: Limoniidae; genus: Dicranomyia; subgenus: Dicranomyia; specificEpithet: frontalis; scientificNameAuthorship: (Staeger, 1840); **Location:** country: Belarus; stateProvince: Gomel; county: Mazyr; locality: Mazyr; decimalLatitude: 52.02; decimalLongitude: 29.3; **Identification:** identifiedBy: D.I. Gavryushin; **Event:** samplingProtocol: Sweep net; eventDate: 2019-06-29/2019-07-31; verbatimEventDate: 29-31/Jul/2019; **Record Level:** institutionCode: ZMMU; basisOfRecord: PreservedSpecimen
- **Type status:** Other material. **Occurrence:** occurrenceRemarks: 1 male; recordedBy: D.I. Gavryushin; individualCount: 1; sex: male; occurrenceID: EU_LIM_090; **Taxon:** scientificName: Dicranomyia (Dicranomyia) frontalis (Staeger, 1840); family: Limoniidae; genus: Dicranomyia; subgenus: Dicranomyia; specificEpithet: frontalis; scientificNameAuthorship: (Staeger, 1840); **Location:** country: Russia; stateProvince: East European Russia; county: Bashkortostan Respublika; municipality: Uchaly district; locality: Ural-Tau St. env.; verbatimElevation: 777 m; minimumElevationInMeters: 777; decimalLatitude: 53.96805; decimalLongitude: 58.57613; **Identification:** identifiedBy: D.I. Gavryushin; **Event:** samplingProtocol: Sweep net; eventDate: 2015-06-09; verbatimEventDate: 09/Jul/2015; **Record Level:** institutionCode: ZMMU; basisOfRecord: PreservedSpecimen

#### Distribution

First records from Belarus and Russia: RUE.

### Dicranomyia (Numantia) fusca

(Meigen, 1804)

EC7FF2E4-D351-5A01-B3C5-86D9B07DCA11

https://ccw.naturalis.nl/detail.php?id=9088

#### Materials

- **Type status:** Other material. **Occurrence:** occurrenceRemarks: 1 male; recordedBy: L.-P. Kolcsár; individualCount: 1; sex: male; preparations: Ethanol; occurrenceID: EU_LIM_235; **Taxon:** scientificName: Dicranomyia (Numantia) fusca (Meigen, 1804); family: Limoniidae; genus: Dicranomyia; subgenus: Numantia; specificEpithet: fusca; scientificNameAuthorship: (Meigen, 1804); **Location:** country: Albania; municipality: Iljas; locality: Gjipe Canyon; verbatimElevation: 307 m; minimumElevationInMeters: 307; decimalLatitude: 40.1428; decimalLongitude: 19.6781; **Identification:** identifiedBy: L.-P. Kolcsár; **Event:** samplingProtocol: Sweep net; eventDate: 2016-05-05; verbatimEventDate: May-05-2016; **Record Level:** institutionCode: CKLP; basisOfRecord: PreservedSpecimen
- **Type status:** Other material. **Occurrence:** occurrenceRemarks: 1 male; recordedBy: L.-P. Kolcsár; individualCount: 1; sex: male; preparations: Ethanol; occurrenceID: EU_LIM_236; **Taxon:** scientificName: Dicranomyia (Numantia) fusca (Meigen, 1804); family: Limoniidae; genus: Dicranomyia; subgenus: Numantia; specificEpithet: fusca; scientificNameAuthorship: (Meigen, 1804); **Location:** country: Montenegro; municipality: Zabljak; locality: Durmitor Mts., Crno Jezero; verbatimElevation: 1440 m; minimumElevationInMeters: 1440; decimalLatitude: 43.14861; decimalLongitude: 19.0882; **Identification:** identifiedBy: L.-P. Kolcsár; **Event:** samplingProtocol: Sweep net; eventDate: 2010-05-11; verbatimEventDate: 11/May/2010; habitat: lakeshore; **Record Level:** institutionCode: CKLP; basisOfRecord: PreservedSpecimen
- **Type status:** Other material. **Occurrence:** occurrenceRemarks: 8 males, 1 female; recordedBy: L.-P. Kolcsár | E. Török; individualCount: 9; sex: male, female; preparations: Ethanol; occurrenceID: EU_LIM_237; **Taxon:** scientificName: Dicranomyia (Numantia) fusca (Meigen, 1804); family: Limoniidae; genus: Dicranomyia; subgenus: Numantia; specificEpithet: fusca; scientificNameAuthorship: (Meigen, 1804); **Location:** country: North Macedonia; municipality: Novo Selo; locality: Bistra Mts., Marlovo NP.; verbatimElevation: 990 m; minimumElevationInMeters: 990; decimalLatitude: 41.71944; decimalLongitude: 20.82889; **Identification:** identifiedBy: L.-P. Kolcsár; **Event:** samplingProtocol: Sweep net; eventDate: 2017-06-29; verbatimEventDate: 29/Jun/2017; **Record Level:** institutionCode: CKLP; basisOfRecord: PreservedSpecimen
- **Type status:** Other material. **Occurrence:** occurrenceRemarks: 1 male; recordedBy: E. Eiroa; individualCount: 1; sex: male; preparations: Pinned; occurrenceID: EU_LIM_238; **Taxon:** scientificName: Dicranomyia (Numantia) fusca (Meigen, 1804); family: Limoniidae; genus: Dicranomyia; subgenus: Numantia; specificEpithet: fusca; scientificNameAuthorship: (Meigen, 1804); **Location:** country: Portugal; stateProvince: Guarda; municipality: Manteigas; locality: road to Poço do Inferno, serra da Estrela; verbatimElevation: 1088 m; minimumElevationInMeters: 1088; decimalLatitude: 40.38384; decimalLongitude: -7.52205; **Identification:** identifiedBy: E. Eiroa; **Event:** samplingProtocol: Sweep net; eventDate: 1992-05-30; verbatimEventDate: 30/May/1992; habitat: riverside vegetation; **Record Level:** institutionCode: USC; basisOfRecord: PreservedSpecimen
- **Type status:** Other material. **Occurrence:** occurrenceRemarks: 1 male; recordedBy: V.E. Pilipenko; individualCount: 1; sex: male; occurrenceID: EU_LIM_239; **Taxon:** scientificName: Dicranomyia (Numantia) fusca (Meigen, 1804); family: Limoniidae; genus: Dicranomyia; subgenus: Numantia; specificEpithet: fusca; scientificNameAuthorship: (Meigen, 1804); **Location:** country: Russia; stateProvince: Central European Russia; county: Moskovskaya Oblast; municipality: Solnechnogorsk district; locality: Chashnikovo; verbatimElevation: 220 m; minimumElevationInMeters: 220; decimalLatitude: 56.0375; decimalLongitude: 37.1874; **Identification:** identifiedBy: V.E. Pilipenko; **Event:** samplingProtocol: Sweep net; eventDate: 1992-06-03; verbatimEventDate: 03/Jun/1992; **Record Level:** institutionCode: VPMC; basisOfRecord: PreservedSpecimen
- **Type status:** Other material. **Occurrence:** occurrenceRemarks: 1 male; recordedBy: V.E. Pilipenko; individualCount: 1; sex: male; occurrenceID: EU_LIM_240; **Taxon:** scientificName: Dicranomyia (Numantia) fusca (Meigen, 1804); family: Limoniidae; genus: Dicranomyia; subgenus: Numantia; specificEpithet: fusca; scientificNameAuthorship: (Meigen, 1804); **Location:** country: Russia; stateProvince: Central European Russia; county: Moskovskaya Oblast; municipality: Solnechnogorsk district; locality: Chashnikovo; verbatimElevation: 220 m; minimumElevationInMeters: 220; decimalLatitude: 56.0375; decimalLongitude: 37.1874; **Identification:** identifiedBy: V.E. Pilipenko; **Event:** samplingProtocol: Sweep net; eventDate: 1992-06-04; verbatimEventDate: 04/Jun/1992; **Record Level:** institutionCode: VPMC; basisOfRecord: PreservedSpecimen
- **Type status:** Other material. **Occurrence:** occurrenceRemarks: 1 male; recordedBy: V.E. Pilipenko; individualCount: 1; sex: male; occurrenceID: EU_LIM_241; **Taxon:** scientificName: Dicranomyia (Numantia) fusca (Meigen, 1804); family: Limoniidae; genus: Dicranomyia; subgenus: Numantia; specificEpithet: fusca; scientificNameAuthorship: (Meigen, 1804); **Location:** country: Russia; stateProvince: Central European Russia; county: Moskovskaya Oblast; municipality: Solnechnogorsk district; locality: Chashnikovo; verbatimElevation: 220 m; minimumElevationInMeters: 220; decimalLatitude: 56.0375; decimalLongitude: 37.1874; **Identification:** identifiedBy: V.E. Pilipenko; **Event:** samplingProtocol: Sweep net; eventDate: 1993-06-26; verbatimEventDate: 26/Jun/1993; **Record Level:** institutionCode: VPMC; basisOfRecord: PreservedSpecimen
- **Type status:** Other material. **Occurrence:** occurrenceRemarks: 1 male, 1 female; recordedBy: V.E. Pilipenko; individualCount: 2; sex: male, female; occurrenceID: EU_LIM_242; **Taxon:** scientificName: Dicranomyia (Numantia) fusca (Meigen, 1804); family: Limoniidae; genus: Dicranomyia; subgenus: Numantia; specificEpithet: fusca; scientificNameAuthorship: (Meigen, 1804); **Location:** country: Russia; stateProvince: Central European Russia; county: Moskovskaya Oblast; municipality: Solnechnogorsk district; locality: Chashnikovo; verbatimElevation: 220 m; minimumElevationInMeters: 220; decimalLatitude: 56.0375; decimalLongitude: 37.1874; **Identification:** identifiedBy: V.E. Pilipenko; **Event:** samplingProtocol: Sweep net; eventDate: 1996-06-15; verbatimEventDate: 15/Jun/1996; **Record Level:** institutionCode: VPMC; basisOfRecord: PreservedSpecimen
- **Type status:** Other material. **Occurrence:** occurrenceRemarks: 1 male; recordedBy: V.E. Pilipenko; individualCount: 1; sex: male; occurrenceID: EU_LIM_243; **Taxon:** scientificName: Dicranomyia (Numantia) fusca (Meigen, 1804); family: Limoniidae; genus: Dicranomyia; subgenus: Numantia; specificEpithet: fusca; scientificNameAuthorship: (Meigen, 1804); **Location:** country: Russia; stateProvince: Central European Russia; county: Moskovskaya Oblast; municipality: Solnechnogorsk district; locality: Chashnikovo; verbatimElevation: 220 m; minimumElevationInMeters: 220; decimalLatitude: 56.0375; decimalLongitude: 37.1874; **Identification:** identifiedBy: V.E. Pilipenko; **Event:** samplingProtocol: Sweep net; eventDate: 1997-08-21; verbatimEventDate: 21/Aug/1997; **Record Level:** institutionCode: VPMC; basisOfRecord: PreservedSpecimen
- **Type status:** Other material. **Occurrence:** occurrenceRemarks: 1 male; recordedBy: A. Polevoi; individualCount: 1; sex: male; preparations: Pinned; occurrenceID: EU_LIM_244; **Taxon:** scientificName: Dicranomyia (Numantia) fusca (Meigen, 1804); family: Limoniidae; genus: Dicranomyia; subgenus: Numantia; specificEpithet: fusca; scientificNameAuthorship: (Meigen, 1804); **Location:** country: Russia; stateProvince: North European Russia; county: Republic Karelia; municipality: Kondopoga district; locality: Shaidoma, Vidruchei; verbatimElevation: 80 m; minimumElevationInMeters: 80; decimalLatitude: 62.73391; decimalLongitude: 34.20126; **Identification:** identifiedBy: A. Polevoi; **Event:** samplingProtocol: Sweep net; eventDate: 2018-08-10; verbatimEventDate: 10/Aug/2018; **Record Level:** institutionCode: FRIP; basisOfRecord: PreservedSpecimen
- **Type status:** Other material. **Occurrence:** occurrenceRemarks: 3 males; recordedBy: N.M. Paramonov; individualCount: 3; sex: male; occurrenceID: EU_LIM_245; **Taxon:** scientificName: Dicranomyia (Numantia) fusca (Meigen, 1804); family: Limoniidae; genus: Dicranomyia; subgenus: Numantia; specificEpithet: fusca; scientificNameAuthorship: (Meigen, 1804); **Location:** country: Russia; stateProvince: East European Russia; county: Tatarstan Respublika; municipality: Verhneuslonsk district; locality: base “Zoostation”, 3,5 km NW Pustye Morkvashi env.; verbatimElevation: 80 m; minimumElevationInMeters: 80; decimalLatitude: 55.47005; decimalLongitude: 48.44092; **Identification:** identifiedBy: N.M. Paramonov; **Event:** samplingProtocol: Sweep net; eventDate: 2013-08-22/2013-08-26; verbatimEventDate: 22-26/Aug/2013; habitat: ravine, wetland; **Record Level:** institutionCode: ZIN; basisOfRecord: PreservedSpecimen
- **Type status:** Other material. **Occurrence:** occurrenceRemarks: 1 male, 1 female; recordedBy: D.I. Gavryushin; individualCount: 2; sex: male, female; preparations: Ethanol; occurrenceID: EU_LIM_246; **Taxon:** scientificName: Dicranomyia (Numantia) fusca (Meigen, 1804); family: Limoniidae; genus: Dicranomyia; subgenus: Numantia; specificEpithet: fusca; scientificNameAuthorship: (Meigen, 1804); **Location:** country: Serbia; stateProvince: Zaječar; municipality: Knjaževac; locality: Crni Vrh; verbatimElevation: 800 m; minimumElevationInMeters: 800; decimalLatitude: 43.407; decimalLongitude: 22.587; **Identification:** identifiedBy: D.I. Gavryushin; **Event:** samplingProtocol: Sweep net; eventDate: 2015-06-01/2015-07-07; verbatimEventDate: 01-07/Jul/2015; **Record Level:** institutionCode: ZMMU; basisOfRecord: PreservedSpecimen
- **Type status:** Other material. **Occurrence:** occurrenceRemarks: 1 male; recordedBy: D.I. Gavryushin; individualCount: 1; sex: male; preparations: Pinned; occurrenceID: EU_LIM_247; **Taxon:** scientificName: Dicranomyia (Numantia) fusca (Meigen, 1804); family: Limoniidae; genus: Dicranomyia; subgenus: Numantia; specificEpithet: fusca; scientificNameAuthorship: (Meigen, 1804); **Location:** country: Serbia; stateProvince: Zaječar; municipality: Knjaževac; locality: Crni Vrh; verbatimElevation: 800 m; minimumElevationInMeters: 800; decimalLatitude: 43.407; decimalLongitude: 22.587; **Identification:** identifiedBy: D.I. Gavryushin; **Event:** samplingProtocol: Sweep net; eventDate: 2014-09-16/2014-09-18; verbatimEventDate: 16-22/Sep/2014; **Record Level:** institutionCode: ZMMU; basisOfRecord: PreservedSpecimen
- **Type status:** Other material. **Occurrence:** occurrenceRemarks: 1 male; recordedBy: D.I. Gavryushin; individualCount: 1; sex: male; preparations: Pinned; occurrenceID: EU_LIM_248; **Taxon:** scientificName: Dicranomyia (Numantia) fusca (Meigen, 1804); family: Limoniidae; genus: Dicranomyia; subgenus: Numantia; specificEpithet: fusca; scientificNameAuthorship: (Meigen, 1804); **Location:** country: Serbia; stateProvince: Zaječar; municipality: Knjaževac; locality: Crni Vrh; verbatimElevation: 800 m; minimumElevationInMeters: 800; decimalLatitude: 43.407; decimalLongitude: 22.587; **Identification:** identifiedBy: D.I. Gavryushin; **Event:** samplingProtocol: Sweep net; eventDate: 2015-06-01/2015-07-07; verbatimEventDate: 01-07/Jul/2015; **Record Level:** institutionCode: ZMMU; basisOfRecord: PreservedSpecimen
- **Type status:** Other material. **Occurrence:** occurrenceRemarks: 2 females; recordedBy: D.I. Gavryushin; individualCount: 2; sex: female; preparations: Ethanol; occurrenceID: EU_LIM_249; **Taxon:** scientificName: Dicranomyia (Numantia) fusca (Meigen, 1804); family: Limoniidae; genus: Dicranomyia; subgenus: Numantia; specificEpithet: fusca; scientificNameAuthorship: (Meigen, 1804); **Location:** country: Serbia; locality: Stara Planina Mts.; verbatimElevation: 1030 m; minimumElevationInMeters: 1030; decimalLatitude: 43.396; decimalLongitude: 22.607; **Identification:** identifiedBy: D.I. Gavryushin; **Event:** samplingProtocol: Sweep net; eventDate: 2015-05-01/2015-05-08; verbatimEventDate: 01-08/May/2015; **Record Level:** institutionCode: ZMMU; basisOfRecord: PreservedSpecimen
- **Type status:** Other material. **Occurrence:** occurrenceRemarks: 2 males; recordedBy: D.I. Gavryushin; individualCount: 2; sex: male; preparations: Pinned; occurrenceID: EU_LIM_250; **Taxon:** scientificName: Dicranomyia (Numantia) fusca (Meigen, 1804); family: Limoniidae; genus: Dicranomyia; subgenus: Numantia; specificEpithet: fusca; scientificNameAuthorship: (Meigen, 1804); **Location:** country: Serbia; locality: Stara Planina Mts.; verbatimElevation: 1030 m; minimumElevationInMeters: 1030; decimalLatitude: 43.396; decimalLongitude: 22.607; **Identification:** identifiedBy: D.I. Gavryushin; **Event:** samplingProtocol: Sweep net; eventDate: 2015-05-01/2015-05-08; verbatimEventDate: 01-08/May/2015; **Record Level:** institutionCode: ZMMU; basisOfRecord: PreservedSpecimen

#### Distribution

First records from Albania, Montenegro, North Macedonia, Portugal, Russia: RUC, RUE, RUN and Serbia.

### Dicranomyia (Idiopyga) halterella

Edwards, 1921

992F453E-F3FF-5E1C-9854-8BA5DA60A7FF

https://ccw.naturalis.nl/detail.php?id=8944

#### Materials

- **Type status:** Other material. **Occurrence:** occurrenceRemarks: 3 males; recordedBy: J. Slípka; individualCount: 3; sex: male; preparations: Pinned; occurrenceID: EU_LIM_193; **Taxon:** scientificName: Dicranomyia (Idiopyga) halterella Edwards, 1921; family: Limoniidae; genus: Dicranomyia; subgenus: Idiopyga; specificEpithet: halterella; scientificNameAuthorship: Edwards, 1921; **Location:** country: Bulgaria; locality: Rila Mts.; verbatimElevation: 2600 m; minimumElevationInMeters: 2600; decimalLatitude: 42.1; decimalLongitude: 23.5; **Identification:** identifiedBy: J. Starý; **Event:** eventDate: 1949-10-02; verbatimEventDate: Oct-02-1949; **Record Level:** institutionCode: PCJS; basisOfRecord: PreservedSpecimen
- **Type status:** Other material. **Occurrence:** occurrenceRemarks: 1 male; recordedBy: L.-P. Kolcsár | E. Török; individualCount: 1; sex: male; preparations: Ethanol; occurrenceID: EU_LIM_194; **Taxon:** scientificName: Dicranomyia (Idiopyga) halterella Edwards, 1921; family: Limoniidae; genus: Dicranomyia; subgenus: Idiopyga; specificEpithet: halterella; scientificNameAuthorship: Edwards, 1921; **Location:** country: Romania; stateProvince: Harghita; municipality: Hagota; locality: Giurgeu Mts., Tisașul Valley; verbatimElevation: 860 m; minimumElevationInMeters: 860; decimalLatitude: 46.86179; decimalLongitude: 25.67723; **Identification:** identifiedBy: L.-P. Kolcsár; **Event:** samplingProtocol: Sweep net; eventDate: 2011-09-07; verbatimEventDate: 7/Sep/2011; **Record Level:** institutionCode: CKLP; basisOfRecord: PreservedSpecimen
- **Type status:** Other material. **Occurrence:** occurrenceRemarks: 1 male; recordedBy: L.-P. Kolcsár | E. Török; individualCount: 1; sex: male; preparations: Ethanol; occurrenceID: EU_LIM_195; **Taxon:** scientificName: Dicranomyia (Idiopyga) halterella Edwards, 1921; family: Limoniidae; genus: Dicranomyia; subgenus: Idiopyga; specificEpithet: halterella; scientificNameAuthorship: Edwards, 1921; **Location:** country: Romania; stateProvince: Harghita; municipality: Toplița; locality: Călimani Mts., Lomaș Valley; verbatimElevation: 1210 m; minimumElevationInMeters: 1210; decimalLatitude: 47.06016; decimalLongitude: 25.29841; **Identification:** identifiedBy: L.-P. Kolcsár; **Event:** samplingProtocol: Sweep net; eventDate: 2011-09-05; verbatimEventDate: 5/Sep/2011; **Record Level:** institutionCode: CKLP; basisOfRecord: PreservedSpecimen
- **Type status:** Other material. **Occurrence:** occurrenceRemarks: 1 male; recordedBy: V.E. Pilipenko; individualCount: 1; sex: male; occurrenceID: EU_LIM_196; **Taxon:** scientificName: Dicranomyia (Idiopyga) halterella Edwards, 1921; family: Limoniidae; genus: Dicranomyia; subgenus: Idiopyga; specificEpithet: halterella; scientificNameAuthorship: Edwards, 1921; **Location:** country: Russia; stateProvince: Central European Russia; county: Moskovskaya Oblast; municipality: Solnechnogorsk district; locality: Chashnikovo; verbatimElevation: 220 m; minimumElevationInMeters: 220; decimalLatitude: 56.0375; decimalLongitude: 37.1874; **Identification:** identifiedBy: V.E. Pilipenko; **Event:** samplingProtocol: Sweep net; eventDate: 1994-09-20; verbatimEventDate: 20/Sep/1994; **Record Level:** institutionCode: VPMC; basisOfRecord: PreservedSpecimen
- **Type status:** Other material. **Occurrence:** occurrenceRemarks: 2 males, 2 females; recordedBy: V.E. Pilipenko; individualCount: 4; sex: male, female; occurrenceID: EU_LIM_197; **Taxon:** scientificName: Dicranomyia (Idiopyga) halterella Edwards, 1921; family: Limoniidae; genus: Dicranomyia; subgenus: Idiopyga; specificEpithet: halterella; scientificNameAuthorship: Edwards, 1921; **Location:** country: Russia; stateProvince: Central European Russia; county: Moskovskaya Oblast; municipality: Solnechnogorsk district; locality: Chashnikovo; verbatimElevation: 220 m; minimumElevationInMeters: 220; decimalLatitude: 56.0375; decimalLongitude: 37.1874; **Identification:** identifiedBy: V.E. Pilipenko; **Event:** samplingProtocol: Sweep net; eventDate: 1995-10-03; verbatimEventDate: 03/Oct/1995; **Record Level:** institutionCode: VPMC; basisOfRecord: PreservedSpecimen

#### Distribution

First records from Bulgaria, Romania and Russia: RUC.

### Dicranomyia (Melanolimonia) hamata

Becker, 1908

A62A1722-9AFD-5C3C-BB69-D2926A364E15

https://ccw.naturalis.nl/detail.php?id=18502

#### Materials

- **Type status:** Other material. **Occurrence:** occurrenceRemarks: 1 male; recordedBy: L.-P. Kolcsár; individualCount: 1; sex: male; preparations: Ethanol; occurrenceID: EU_LIM_224; **Taxon:** scientificName: Dicranomyia (Melanolimonia) hamata Becker, 1908; family: Limoniidae; genus: Dicranomyia; subgenus: Melanolimonia; specificEpithet: hamata; scientificNameAuthorship: Becker, 1908; **Location:** country: Albania; municipality: Iljas; locality: Gjipe Canyon; verbatimElevation: 307 m; minimumElevationInMeters: 307; decimalLatitude: 40.1428; decimalLongitude: 19.6781; **Identification:** identifiedBy: L.-P. Kolcsár; **Event:** samplingProtocol: Sweep net; eventDate: 2016-05-05; verbatimEventDate: 05/May/2016; **Record Level:** institutionCode: CKLP; basisOfRecord: PreservedSpecimen

#### Description

Fig. 4

#### Distribution

First record from Albania.

### Dicranomyia (Dicranomyia) imbecilla

Lackschewitz, 1941

00D03069-A4F7-565B-AADF-33F84A38604D

https://ccw.naturalis.nl/detail.php?id=8309

#### Materials

- **Type status:** Other material. **Occurrence:** catalogNumber: JES-20120305; occurrenceRemarks: 1 male; recordedBy: J. Salmela; individualCount: 1; sex: male; preparations: Ethanol; occurrenceID: EU_LIM_091; **Taxon:** scientificName: Dicranomyia (Dicranomyia) imbecilla Lackschewitz, 1941; family: Limoniidae; genus: Dicranomyia; subgenus: Dicranomyia; specificEpithet: imbecilla; scientificNameAuthorship: Lackschewitz, 1941; **Location:** country: Finland; stateProvince: Lapponia inariensis; municipality: Utsjoki; locality: Galddasduolbbas; verbatimElevation: 295 m; minimumElevationInMeters: 295; decimalLatitude: 69.86; decimalLongitude: 27.77; **Identification:** identifiedBy: J. Salmela; **Event:** samplingProtocol: Malaise trap; eventDate: 2007-08-27; verbatimEventDate: Aug-27-2007; **Record Level:** institutionCode: LMM; basisOfRecord: PreservedSpecimen
- **Type status:** Other material. **Occurrence:** occurrenceRemarks: 1 male; recordedBy: D.I. Gavryushin; individualCount: 1; sex: male; occurrenceID: EU_LIM_092; **Taxon:** scientificName: Dicranomyia (Dicranomyia) imbecilla Lackschewitz, 1941; family: Limoniidae; genus: Dicranomyia; subgenus: Dicranomyia; specificEpithet: imbecilla; scientificNameAuthorship: Lackschewitz, 1941; **Location:** country: Russia; stateProvince: East European Russia; county: Bashkortostan Respublika; municipality: Beloretsk district; locality: Nura River (ca. 4km W of Otnurok village), at the foot of Zolotyie Shishki (Golden Cones) Mts.; verbatimElevation: 607 m; minimumElevationInMeters: 607; decimalLatitude: 54.05155; decimalLongitude: 58.26887; **Identification:** identifiedBy: D.I. Gavryushin; **Event:** samplingProtocol: Sweep net; eventDate: 2015-06-13; verbatimEventDate: 13/Jul/2015; **Record Level:** institutionCode: ZMMU; basisOfRecord: PreservedSpecimen

#### Distribution

First records from Finland and Russia: RUE.

### Dicranomyia (Glochina) liberta

Osten Sacken, 1860

0E47B171-59A0-563B-9B73-CAD1C38458BD

https://ccw.naturalis.nl/detail.php?id=8880

#### Materials

- **Type status:** Other material. **Occurrence:** occurrenceRemarks: 1 male; recordedBy: D.I. Gavryushin; individualCount: 1; sex: male; preparations: Pinned; occurrenceID: EU_LIM_180; **Taxon:** scientificName: Dicranomyia (Glochina) liberta Osten Sacken, 1860; family: Limoniidae; genus: Dicranomyia; subgenus: Glochina; specificEpithet: liberta; scientificNameAuthorship: Osten Sacken, 1860; **Location:** country: Belarus; stateProvince: Vitebsk; county: Orsha; locality: Orsha; decimalLatitude: 54.555; decimalLongitude: 30.63; **Identification:** identifiedBy: D.I. Gavryushin; **Event:** samplingProtocol: Sweep net; eventDate: 2019-06-28; verbatimEventDate: 28/Jul/2019; **Record Level:** institutionCode: ZMMU; basisOfRecord: PreservedSpecimen

#### Distribution

First record from Belarus.

### Dicranomyia (Dicranomyia) longipennis

(Schummel, 1829)

E71E4151-E241-5F1E-988E-D0AC7791812B

https://ccw.naturalis.nl/detail.php?id=8402

#### Materials

- **Type status:** Other material. **Occurrence:** occurrenceRemarks: 1 male; recordedBy: D.I. Gavryushin; individualCount: 1; sex: male; preparations: Pinned; occurrenceID: EU_LIM_093; **Taxon:** scientificName: Dicranomyia (Dicranomyia) longipennis (Schummel, 1829); family: Limoniidae; genus: Dicranomyia; subgenus: Dicranomyia; specificEpithet: longipennis; scientificNameAuthorship: (Schummel, 1829); **Location:** country: Serbia; stateProvince: Zaječar; municipality: Knjaževac; locality: Crni Vrh; verbatimElevation: 800 m; minimumElevationInMeters: 800; decimalLatitude: 43.407; decimalLongitude: 22.587; **Identification:** identifiedBy: D.I. Gavryushin; **Event:** samplingProtocol: Sweep net; eventDate: 2015-06-01/2015-07-07; verbatimEventDate: 01-07/Jul/2015; **Record Level:** institutionCode: ZMMU; basisOfRecord: PreservedSpecimen

#### Distribution

First record from Serbia.

### Dicranomyia (Idiopyga) lulensis

(Tjeder, 1969)

D4448E94-4D2A-5C47-ABDD-CBE8C1003574

https://ccw.naturalis.nl/detail.php?id=8949

#### Materials

- **Type status:** Other material. **Occurrence:** catalogNumber: 585268, 644165; occurrenceRemarks: 2 male+female; recordedBy: K.M. Olsen | Ø. Gammelmo | J. Salmela; individualCount: 2; sex: male, female; preparations: Ethanol; occurrenceID: EU_LIM_198; **Taxon:** scientificName: Dicranomyia (Idiopyga) lulensis (Tjeder, 1969); family: Limoniidae; genus: Dicranomyia; subgenus: Idiopyga; specificEpithet: lulensis; scientificNameAuthorship: (Tjeder, 1969); **Location:** country: Norway; stateProvince: Finnmark; municipality: Kautokeino; locality: Dierbajohka – S veien; verbatimElevation: 400 m; minimumElevationInMeters: 400; decimalLatitude: 69.12007; decimalLongitude: 22.71557; **Identification:** identifiedBy: K.M. Olsen; **Event:** samplingProtocol: Sweep net; eventDate: 2018-08-20; verbatimEventDate: 20/Aug/2018; **Record Level:** institutionCode: PCKMO; basisOfRecord: PreservedSpecimen
- **Type status:** Other material. **Occurrence:** catalogNumber: 585320; occurrenceRemarks: 1 male; recordedBy: K.M. Olsen | Ø. Gammelmo | J. Salmela; individualCount: 1; sex: male; preparations: Ethanol; occurrenceID: EU_LIM_199; **Taxon:** scientificName: Dicranomyia (Idiopyga) lulensis (Tjeder, 1969); family: Limoniidae; genus: Dicranomyia; subgenus: Idiopyga; specificEpithet: lulensis; scientificNameAuthorship: (Tjeder, 1969); **Location:** country: Norway; stateProvince: Finnmark; municipality: Kautokeino; locality: Borrejávri – (Søndre); verbatimElevation: 420 m; minimumElevationInMeters: 420; decimalLatitude: 69.55512; decimalLongitude: 23.51802; **Identification:** identifiedBy: K.M. Olsen; **Event:** samplingProtocol: Sweep net; eventDate: 2018-08-21; verbatimEventDate: 21/Aug/2018; **Record Level:** institutionCode: NHMO; basisOfRecord: PreservedSpecimen
- **Type status:** Other material. **Occurrence:** catalogNumber: 585298; occurrenceRemarks: 4 males; recordedBy: K.M. Olsen | Ø. Gammelmo | J. Salmela; individualCount: 4; sex: male; preparations: Ethanol; occurrenceID: EU_LIM_200; **Taxon:** scientificName: Dicranomyia (Idiopyga) lulensis (Tjeder, 1969); family: Limoniidae; genus: Dicranomyia; subgenus: Idiopyga; specificEpithet: lulensis; scientificNameAuthorship: (Tjeder, 1969); **Location:** country: Norway; stateProvince: Finnmark; municipality: Kautokeino; locality: Suolovuopmi – N Unna Suolojávrrás; verbatimElevation: 380 m; minimumElevationInMeters: 380; decimalLatitude: 69.579; decimalLongitude: 23.54476; **Identification:** identifiedBy: K.M. Olsen; **Event:** samplingProtocol: Sweep net; eventDate: 2018-08-21; verbatimEventDate: 21/Aug/2018; **Record Level:** institutionCode: NHMO; basisOfRecord: PreservedSpecimen
- **Type status:** Other material. **Occurrence:** catalogNumber: NVO.ins2018-102; occurrenceRemarks: 4 male+female; recordedBy: J. Salmela | Ø. Gammelmo | K.M. Olsen; individualCount: 4; sex: male, female; preparations: Ethanol; occurrenceID: EU_LIM_201; **Taxon:** scientificName: Dicranomyia (Idiopyga) lulensis (Tjeder, 1969); family: Limoniidae; genus: Dicranomyia; subgenus: Idiopyga; specificEpithet: lulensis; scientificNameAuthorship: (Tjeder, 1969); **Location:** country: Norway; stateProvince: Finnmark; municipality: Kautokeino; locality: Suolovuopmi – N Unna Suolojávrrás; verbatimElevation: 380 m; minimumElevationInMeters: 380; decimalLatitude: 69.579; decimalLongitude: 23.54476; **Identification:** identifiedBy: J. Salmela; **Event:** samplingProtocol: Sweep net; eventDate: 2018-08-21; verbatimEventDate: 21/Aug/2018; **Record Level:** institutionCode: LMM; basisOfRecord: PreservedSpecimen

#### Distribution

First records from Norway.

### Dicranomyia (Dicranomyia) lutea

(Meigen, 1804)

D6DB5E69-7CF8-5B5C-91EA-A34901BFCB35

https://ccw.naturalis.nl/detail.php?id=8411

#### Materials

- **Type status:** Other material. **Occurrence:** catalogNumber: 574356, 574357; occurrenceRemarks: 4 males; recordedBy: K.M. Olsen; individualCount: 4; sex: male; preparations: Ethanol; occurrenceID: EU_LIM_094; **Taxon:** scientificName: Dicranomyia (Dicranomyia) lutea (Meigen, 1804); family: Limoniidae; genus: Dicranomyia; subgenus: Dicranomyia; specificEpithet: lutea; scientificNameAuthorship: (Meigen, 1804); **Location:** country: Norway; stateProvince: Akershus; municipality: Bærum; locality: N Oksenøyveien – W nr. 71; verbatimElevation: 6 m; minimumElevationInMeters: 6; decimalLatitude: 59.89985; decimalLongitude: 10.60751; **Identification:** identifiedBy: K.M. Olsen; **Event:** samplingProtocol: Malaise trap; eventDate: 2017-06-05/2017-08-01; verbatimEventDate: 05/Jun-01/Aug/2017; **Record Level:** institutionCode: PCKMO; basisOfRecord: PreservedSpecimen
- **Type status:** Other material. **Occurrence:** catalogNumber: 524329; occurrenceRemarks: 1 male; recordedBy: K.M. Olsen; individualCount: 1; sex: male; preparations: Ethanol; occurrenceID: EU_LIM_095; **Taxon:** scientificName: Dicranomyia (Dicranomyia) lutea (Meigen, 1804); family: Limoniidae; genus: Dicranomyia; subgenus: Dicranomyia; specificEpithet: lutea; scientificNameAuthorship: (Meigen, 1804); **Location:** country: Norway; stateProvince: Østfold; municipality: Halden; locality: E SkRiverøya; verbatimElevation: 0-1 m; minimumElevationInMeters: 0; maximumElevationInMeters: 1; decimalLatitude: 59.0591; decimalLongitude: 11.41393; **Identification:** identifiedBy: K.M. Olsen; **Event:** samplingProtocol: Sweep net; eventDate: 2017-05-23; verbatimEventDate: 23/May/2017; habitat: Bĺde nordre og sřndre strandeng; **Record Level:** institutionCode: PCKMO; basisOfRecord: PreservedSpecimen
- **Type status:** Other material. **Occurrence:** catalogNumber: 528591; occurrenceRemarks: 1 male; recordedBy: K. Berggren; individualCount: 1; sex: male; preparations: Ethanol; occurrenceID: EU_LIM_096; **Taxon:** scientificName: Dicranomyia (Dicranomyia) lutea (Meigen, 1804); family: Limoniidae; genus: Dicranomyia; subgenus: Dicranomyia; specificEpithet: lutea; scientificNameAuthorship: (Meigen, 1804); **Location:** country: Norway; stateProvince: Vest-Agder; municipality: Kristiansand; locality: Bråvann terrasse – I hagen til nr. 21; verbatimElevation: 75 m; minimumElevationInMeters: 75; decimalLatitude: 58.11029; decimalLongitude: 7.93429; **Identification:** identifiedBy: K.M. Olsen; **Event:** samplingProtocol: Light trap; eventDate: 2017-06/2017-07; verbatimEventDate: Jun-Jul/2017; **Record Level:** institutionCode: PCKMO; basisOfRecord: PreservedSpecimen
- **Type status:** Other material. **Occurrence:** catalogNumber: 528448; occurrenceRemarks: 1 male; recordedBy: K.M. Olsen; individualCount: 1; sex: male; preparations: Ethanol; occurrenceID: EU_LIM_097; **Taxon:** scientificName: Dicranomyia (Dicranomyia) lutea (Meigen, 1804); family: Limoniidae; genus: Dicranomyia; subgenus: Dicranomyia; specificEpithet: lutea; scientificNameAuthorship: (Meigen, 1804); **Location:** country: Norway; stateProvince: Vestfold; municipality: Tjøme; locality: Kolabekkilen – Svartorstrandskog i NE; verbatimElevation: 0-1 m; minimumElevationInMeters: 0; maximumElevationInMeters: 1; decimalLatitude: 59.09218; decimalLongitude: 10.40413; **Identification:** identifiedBy: K.M. Olsen; **Event:** samplingProtocol: Sweep net; eventDate: 2017-05-30; verbatimEventDate: 30/May/2017; **Record Level:** institutionCode: PCKMO; basisOfRecord: PreservedSpecimen
- **Type status:** Other material. **Occurrence:** catalogNumber: 536559; occurrenceRemarks: 1 male; recordedBy: L.O. Hansen; individualCount: 1; sex: male; preparations: Ethanol; occurrenceID: EU_LIM_098; **Taxon:** scientificName: Dicranomyia (Dicranomyia) lutea (Meigen, 1804); family: Limoniidae; genus: Dicranomyia; subgenus: Dicranomyia; specificEpithet: lutea; scientificNameAuthorship: (Meigen, 1804); **Location:** country: Norway; stateProvince: Vestfold; municipality: Re; locality: Langøya; verbatimElevation: 5 m; minimumElevationInMeters: 5; decimalLatitude: 59.49494; decimalLongitude: 10.37418; **Identification:** identifiedBy: K.M. Olsen; **Event:** samplingProtocol: Light trap; eventDate: 1987-06; verbatimEventDate: Jul/1987; **Record Level:** institutionCode: PCKMO; basisOfRecord: PreservedSpecimen

#### Distribution

First records from Norway.

### Dicranomyia (Dicranomyia) luteipennis

Goetghebuer, 1920

ABC5E1EB-D893-5CEC-B2CB-8A5B9049FDC8

https://ccw.naturalis.nl/detail.php?id=8414

#### Materials

- **Type status:** Other material. **Occurrence:** occurrenceRemarks: 1 male; recordedBy: E. Eiroa; individualCount: 1; sex: male; preparations: Pinned; occurrenceID: EU_LIM_099; **Taxon:** scientificName: Dicranomyia (Dicranomyia) luteipennis Goetghebuer, 1920; family: Limoniidae; genus: Dicranomyia; subgenus: Dicranomyia; specificEpithet: luteipennis; scientificNameAuthorship: Goetghebuer, 1920; **Location:** country: Spain; stateProvince: Extremadura, Cáceres; municipality: Cuacos de Yuste; locality: Yuste, near monastery; verbatimElevation: 683 m; minimumElevationInMeters: 683; decimalLatitude: 40.11339; decimalLongitude: -5.74043; **Identification:** identifiedBy: E. Eiroa; **Event:** samplingProtocol: Sweep net; eventDate: 1995-04-23; verbatimEventDate: 23/April/1995; **Record Level:** institutionCode: USC; basisOfRecord: PreservedSpecimen

#### Distribution

First record from Spain (from mainland).

### Dicranomyia (Idiopyga) magnicauda

Lundström, 1912

FCFB6A19-09D6-5BA7-9CA6-FBCAB292179E

https://ccw.naturalis.nl/detail.php?id=8950

#### Materials

- **Type status:** Other material. **Occurrence:** catalogNumber: 609910, 644166; occurrenceRemarks: 2 males; recordedBy: K.M. Olsen | S. Reiso; individualCount: 2; sex: male; preparations: Ethanol; occurrenceID: EU_LIM_202; **Taxon:** scientificName: Dicranomyia (Idiopyga) magnicauda Lundstrom, 1912; family: Limoniidae; genus: Dicranomyia; subgenus: Idiopyga; specificEpithet: magnicauda; scientificNameAuthorship: Lundstrom, 1912; **Location:** country: Norway; stateProvince: Telemark; municipality: Skien; locality: Børsesjø – Ved kanalen, ca. 70 m N broen, samt ved fugletårnet; verbatimElevation: 15 m; minimumElevationInMeters: 15; decimalLatitude: 59.22137; decimalLongitude: 9.62615; **Identification:** identifiedBy: K.M. Olsen; **Event:** samplingProtocol: Malaise trap; eventDate: 2004-08-19/2004-09-10; verbatimEventDate: 19/Aug-10/Sep/2004; **Record Level:** institutionCode: PCKMO; basisOfRecord: PreservedSpecimen

#### Distribution

First record from Norway.

### Dicranomyia (Idiopyga) megacauda

Alexander, 1924

2B2FED3E-963A-528A-B4EB-916B792EADFB

https://ccw.naturalis.nl/detail.php?id=8951

#### Materials

- **Type status:** Other material. **Occurrence:** occurrenceRemarks: 4 males; recordedBy: J. Starý; individualCount: 4; sex: male; preparations: Pinned; occurrenceID: EU_LIM_203; **Taxon:** scientificName: Dicranomyia (Idiopyga) megacauda Alexander, 1924; family: Limoniidae; genus: Dicranomyia; subgenus: Idiopyga; specificEpithet: megacauda; scientificNameAuthorship: Alexander, 1924; **Location:** country: Austria; stateProvince: Styria; municipality: Admont; locality: Hall-Grieshof; decimalLatitude: 47.584; decimalLongitude: 14.471; **Identification:** identifiedBy: J. Starý; **Event:** samplingProtocol: Sweep net; eventDate: 2006-06-15; verbatimEventDate: 15/Jun/2006; habitat: brook and boggy meadows; **Record Level:** institutionCode: PCJS; basisOfRecord: PreservedSpecimen

#### Distribution

First record from Austria.

### Dicranomyia (Dicranomyia) melanantha

Savchenko, 1984

348511F0-8C78-539E-9B7C-CA34140FD052

https://ccw.naturalis.nl/detail.php?id=8434

#### Materials

- **Type status:** Other material. **Occurrence:** occurrenceRemarks: 5 males, 1 female; recordedBy: J. Roháček; individualCount: 6; sex: male, female; preparations: Pinned; occurrenceID: EU_LIM_100; **Taxon:** scientificName: Dicranomyia (Dicranomyia) melanantha Savchenko, 1984; family: Limoniidae; genus: Dicranomyia; subgenus: Dicranomyia; specificEpithet: melanantha; scientificNameAuthorship: Savchenko, 1984; **Location:** country: Greece; stateProvince: Peloponnese; municipality: Alagonia; locality: 2.4 km NW, Taygetos Mts.; verbatimElevation: 1335 m; minimumElevationInMeters: 1335; decimalLatitude: 37.11528; decimalLongitude: 22.26861; **Identification:** identifiedBy: J. Starý; **Event:** eventDate: 2017-10-07; verbatimEventDate: 7/Oct/2017; habitat: brook, spring; **Record Level:** institutionCode: PCJS; basisOfRecord: PreservedSpecimen
- **Type status:** Other material. **Occurrence:** occurrenceRemarks: 13 males, 2 female; recordedBy: J. Starý; individualCount: 15; sex: male, female; preparations: Pinned; occurrenceID: EU_LIM_102; **Taxon:** scientificName: Dicranomyia (Dicranomyia) melanantha Savchenko, 1984; family: Limoniidae; genus: Dicranomyia; subgenus: Dicranomyia; specificEpithet: melanantha; scientificNameAuthorship: Savchenko, 1984; **Location:** country: Greece; stateProvince: Peloponnese; municipality: Alagonia; locality: 2.4 km NW, Taygetos Mts.; verbatimElevation: 1336 m; minimumElevationInMeters: 1336; decimalLatitude: 37.11528; decimalLongitude: 22.26861; **Identification:** identifiedBy: J. Starý; **Event:** eventDate: 2017-10-09; verbatimEventDate: 9/Oct/2017; habitat: brook, spring; **Record Level:** institutionCode: PCJS; basisOfRecord: PreservedSpecimen

#### Distribution

First records from Greece (from mainland).

### Dicranomyia (Idiopyga) melleicauda stenoptera

Savchenko, 1970

3663C734-832C-51DC-B376-FA47C35000F0

https://ccw.naturalis.nl/detail.php?id=8955

#### Materials

- **Type status:** Other material. **Occurrence:** occurrenceRemarks: 4 males, 4 females; recordedBy: L.-P. Kolcsár; individualCount: 8; sex: male, female; preparations: Ethanol; occurrenceID: EU_LIM_204; **Taxon:** scientificName: Dicranomyia (Idiopyga) melleicauda stenoptera Savchenko, 1970; family: Limoniidae; genus: Dicranomyia; subgenus: Idiopyga; specificEpithet: melleicauda; infraspecificEpithet: stenoptera; scientificNameAuthorship: Savchenko, 1970; **Location:** country: Norway; stateProvince: Finnmark; municipality: Porsanger; locality: Lakselv; verbatimElevation: 0.5 m; minimumElevationInMeters: 0.5; decimalLatitude: 70.0757; decimalLongitude: 24.9241; **Identification:** identifiedBy: L.-P. Kolcsár; **Event:** samplingProtocol: Sweep net; eventDate: 2018-08-22; verbatimEventDate: Aug-22-2018; habitat: salt marsh; **Record Level:** institutionCode: CKLP; basisOfRecord: PreservedSpecimen
- **Type status:** Other material. **Occurrence:** catalogNumber: 585626; occurrenceRemarks: 24 male+female; recordedBy: K.M. Olsen | Ø. Gammelmo; individualCount: 24; sex: male, female; preparations: Ethanol; occurrenceID: EU_LIM_205; **Taxon:** scientificName: Dicranomyia (Idiopyga) melleicauda stenoptera Savchenko, 1970; family: Limoniidae; genus: Dicranomyia; subgenus: Idiopyga; specificEpithet: melleicauda; infraspecificEpithet: stenoptera; scientificNameAuthorship: Savchenko, 1970; **Location:** country: Norway; stateProvince: Finnmark; municipality: Alta; locality: Bunes; verbatimElevation: 0.5 m; minimumElevationInMeters: 0.5; decimalLatitude: 70.01476; decimalLongitude: 23.52966; **Identification:** identifiedBy: K.M. Olsen; **Event:** samplingProtocol: Sweep net; eventDate: 2018-08-23; verbatimEventDate: 23/Aug/2018; **Record Level:** institutionCode: PCKMO; basisOfRecord: PreservedSpecimen
- **Type status:** Other material. **Occurrence:** catalogNumber: 585006; occurrenceRemarks: 10 male+female; recordedBy: K.M. Olsen | Ø. Gammelmo; individualCount: 10; sex: male, female; preparations: Ethanol; occurrenceID: EU_LIM_206; **Taxon:** scientificName: Dicranomyia (Idiopyga) melleicauda stenoptera Savchenko, 1970; family: Limoniidae; genus: Dicranomyia; subgenus: Idiopyga; specificEpithet: melleicauda; infraspecificEpithet: stenoptera; scientificNameAuthorship: Savchenko, 1970; **Location:** country: Norway; stateProvince: Finnmark; municipality: Kvalsund; locality: Oldernes – SSE skrotemarken; verbatimElevation: 0.5 m; minimumElevationInMeters: 0.5; decimalLatitude: 70.44766; decimalLongitude: 24.31427; **Identification:** identifiedBy: K.M. Olsen; **Event:** samplingProtocol: Sweep net; eventDate: 2018-08-23; verbatimEventDate: 23/Aug/2018; **Record Level:** institutionCode: NHMO; basisOfRecord: PreservedSpecimen
- **Type status:** Other material. **Occurrence:** catalogNumber: 585491; occurrenceRemarks: 10 male+female; recordedBy: K.M. Olsen | Ø. Gammelmo | J. Salmela; individualCount: 10; sex: male, female; preparations: Ethanol; occurrenceID: EU_LIM_207; **Taxon:** scientificName: Dicranomyia (Idiopyga) melleicauda stenoptera Savchenko, 1970; family: Limoniidae; genus: Dicranomyia; subgenus: Idiopyga; specificEpithet: melleicauda; infraspecificEpithet: stenoptera; scientificNameAuthorship: Savchenko, 1970; **Location:** country: Norway; stateProvince: Finnmark; municipality: Porsanger; locality: Lakselvmunningen – På vestsiden; verbatimElevation: 0.5 m; minimumElevationInMeters: 0.5; decimalLatitude: 70.08148; decimalLongitude: 24.91744; **Identification:** identifiedBy: K.M. Olsen; **Event:** samplingProtocol: Sweep net; eventDate: 2018-08-22; verbatimEventDate: 22/Aug/2018; **Record Level:** institutionCode: NHMO; basisOfRecord: PreservedSpecimen
- **Type status:** Other material. **Occurrence:** catalogNumber: 585443, 644167; occurrenceRemarks: 21 male+female; recordedBy: K.M. Olsen | Ø. Gammelmo | J. Salmela; individualCount: 21; sex: male, female; preparations: Ethanol; occurrenceID: EU_LIM_208; **Taxon:** scientificName: Dicranomyia (Idiopyga) melleicauda stenoptera Savchenko, 1970; family: Limoniidae; genus: Dicranomyia; subgenus: Idiopyga; specificEpithet: melleicauda; infraspecificEpithet: stenoptera; scientificNameAuthorship: Savchenko, 1970; **Location:** country: Norway; stateProvince: Finnmark; municipality: Porsanger; locality: S Stornes; verbatimElevation: 0.5 m; minimumElevationInMeters: 0.5; decimalLatitude: 70.19462; decimalLongitude: 24.91884; **Identification:** identifiedBy: K.M. Olsen; **Event:** samplingProtocol: Sweep net; eventDate: 2018-08-22; verbatimEventDate: 22/Aug/2018; **Record Level:** institutionCode: PCKMO; basisOfRecord: PreservedSpecimen
- **Type status:** Other material. **Occurrence:** catalogNumber: 584996; occurrenceRemarks: 14 male+female; recordedBy: K.M. Olsen | Ø. Gammelmo | J. Salmela; individualCount: 14; sex: male, female; preparations: Ethanol; occurrenceID: EU_LIM_209; **Taxon:** scientificName: Dicranomyia (Idiopyga) melleicauda stenoptera Savchenko, 1970; family: Limoniidae; genus: Dicranomyia; subgenus: Idiopyga; specificEpithet: melleicauda; infraspecificEpithet: stenoptera; scientificNameAuthorship: Savchenko, 1970; **Location:** country: Norway; stateProvince: Finnmark; municipality: Porsanger; locality: Uhca Gáhtir; verbatimElevation: 0.5 m; minimumElevationInMeters: 0.5; decimalLatitude: 70.11393; decimalLongitude: 24.92069; **Identification:** identifiedBy: K.M. Olsen; **Event:** samplingProtocol: Sweep net; eventDate: 2018-08-22; verbatimEventDate: 22/Aug/2018; **Record Level:** institutionCode: NHMO; basisOfRecord: PreservedSpecimen
- **Type status:** Other material. **Occurrence:** catalogNumber: 585554; occurrenceRemarks: 14 male+female; recordedBy: J. Salmela | Ø. Gammelmo | K.M. Olsen; individualCount: 14; sex: male, female; preparations: Ethanol; occurrenceID: EU_LIM_210; **Taxon:** scientificName: Dicranomyia (Idiopyga) melleicauda stenoptera Savchenko, 1970; family: Limoniidae; genus: Dicranomyia; subgenus: Idiopyga; specificEpithet: melleicauda; infraspecificEpithet: stenoptera; scientificNameAuthorship: Savchenko, 1970; **Location:** country: Norway; stateProvince: Finnmark; municipality: Porsanger; locality: Seines; verbatimElevation: 0.5 m; minimumElevationInMeters: 0.5; decimalLatitude: 70.08047; decimalLongitude: 24.94146; **Identification:** identifiedBy: K.M. Olsen; **Event:** samplingProtocol: Sweep net; eventDate: 2018-08-23; verbatimEventDate: 23/Aug/2018; **Record Level:** institutionCode: NHMO; basisOfRecord: PreservedSpecimen
- **Type status:** Other material. **Occurrence:** catalogNumber: 588048, 588049; occurrenceRemarks: 17 male+female; recordedBy: J. Salmela | Ø. Gammelmo | K.M. Olsen; individualCount: 17; sex: male, female; preparations: Ethanol; occurrenceID: EU_LIM_211; **Taxon:** scientificName: Dicranomyia (Idiopyga) melleicauda stenoptera Savchenko, 1970; family: Limoniidae; genus: Dicranomyia; subgenus: Idiopyga; specificEpithet: melleicauda; infraspecificEpithet: stenoptera; scientificNameAuthorship: Savchenko, 1970; **Location:** country: Norway; stateProvince: Finnmark; municipality: Porsanger; locality: S Stornes; verbatimElevation: 0.5 m; minimumElevationInMeters: 0.5; decimalLatitude: 70.19462; decimalLongitude: 24.91884; **Identification:** identifiedBy: Ø. Gammelmo; **Event:** samplingProtocol: Sweep net; eventDate: 2018-08-22; verbatimEventDate: 22/Aug/2018; **Record Level:** institutionCode: BioFokus; basisOfRecord: PreservedSpecimen
- **Type status:** Other material. **Occurrence:** catalogNumber: 588038, 588039; occurrenceRemarks: 17 male+female; recordedBy: Ø. Gammelmo | J. Salmela | K.M. Olsen; individualCount: 17; sex: male, female; preparations: Ethanol; occurrenceID: EU_LIM_212; **Taxon:** scientificName: Dicranomyia (Idiopyga) melleicauda stenoptera Savchenko, 1970; family: Limoniidae; genus: Dicranomyia; subgenus: Idiopyga; specificEpithet: melleicauda; infraspecificEpithet: stenoptera; scientificNameAuthorship: Savchenko, 1970; **Location:** country: Norway; stateProvince: Finnmark; municipality: Porsanger; locality: Seines; verbatimElevation: 0.5 m; minimumElevationInMeters: 0.5; decimalLatitude: 70.08047; decimalLongitude: 24.94146; **Identification:** identifiedBy: Ø. Gammelmo; **Event:** samplingProtocol: Sweep net; eventDate: 2018-08-23; verbatimEventDate: 23/Aug/2018; **Record Level:** institutionCode: BioFokus; basisOfRecord: PreservedSpecimen
- **Type status:** Other material. **Occurrence:** catalogNumber: 588046, 588047; occurrenceRemarks: 3 male+female; recordedBy: Ø. Gammelmo | K.M. Olsen; individualCount: 3; sex: male, female; preparations: Ethanol; occurrenceID: EU_LIM_213; **Taxon:** scientificName: Dicranomyia (Idiopyga) melleicauda stenoptera Savchenko, 1970; family: Limoniidae; genus: Dicranomyia; subgenus: Idiopyga; specificEpithet: melleicauda; infraspecificEpithet: stenoptera; scientificNameAuthorship: Savchenko, 1970; **Location:** country: Norway; stateProvince: Finnmark; municipality: Kvalsund; locality: Oldernes – SSE skrotemarken; verbatimElevation: 0.5 m; minimumElevationInMeters: 0.5; decimalLatitude: 70.44766; decimalLongitude: 24.31427; **Identification:** identifiedBy: Ø. Gammelmo; **Event:** samplingProtocol: Sweep net; eventDate: 2018-08-23; verbatimEventDate: 23/Aug/2018; **Record Level:** institutionCode: BioFokus; basisOfRecord: PreservedSpecimen
- **Type status:** Other material. **Occurrence:** catalogNumber: NVO.ins2018-153; occurrenceRemarks: 7 male+female; recordedBy: J. Salmela | Ø. Gammelmo | K.M. Olsen; individualCount: 7; sex: male, female; preparations: Ethanol; occurrenceID: EU_LIM_214; **Taxon:** scientificName: Dicranomyia (Idiopyga) melleicauda stenoptera Savchenko, 1970; family: Limoniidae; genus: Dicranomyia; subgenus: Idiopyga; specificEpithet: melleicauda; infraspecificEpithet: stenoptera; scientificNameAuthorship: Savchenko, 1970; **Location:** country: Norway; stateProvince: Finnmark; municipality: Porsanger; locality: Lakselvmunningen – På vestsiden; verbatimElevation: 0.5 m; minimumElevationInMeters: 0.5; decimalLatitude: 70.08148; decimalLongitude: 24.91744; **Identification:** identifiedBy: J. Salmela; **Event:** samplingProtocol: Sweep net; eventDate: 2018-08-22; verbatimEventDate: 22/Aug/2018; **Record Level:** institutionCode: LMM; basisOfRecord: PreservedSpecimen
- **Type status:** Other material. **Occurrence:** catalogNumber: NVO.ins2018-146; occurrenceRemarks: 2 male+female; recordedBy: J. Salmela | Ø. Gammelmo | K.M. Olsen; individualCount: 2; sex: male, female; preparations: Ethanol; occurrenceID: EU_LIM_215; **Taxon:** scientificName: Dicranomyia (Idiopyga) melleicauda stenoptera Savchenko, 1970; family: Limoniidae; genus: Dicranomyia; subgenus: Idiopyga; specificEpithet: melleicauda; infraspecificEpithet: stenoptera; scientificNameAuthorship: Savchenko, 1970; **Location:** country: Norway; stateProvince: Finnmark; municipality: Porsanger; locality: S Stornes; verbatimElevation: 0.5 m; minimumElevationInMeters: 0.5; decimalLatitude: 70.19462; decimalLongitude: 24.91884; **Identification:** identifiedBy: J. Salmela; **Event:** samplingProtocol: Sweep net; eventDate: 2018-08-22; verbatimEventDate: 22/Aug/2018; **Record Level:** institutionCode: LMM; basisOfRecord: PreservedSpecimen
- **Type status:** Other material. **Occurrence:** catalogNumber: NVO.ins2018-147; occurrenceRemarks: 14 male+female; recordedBy: J. Salmela | Ø. Gammelmo | K.M. Olsen; individualCount: 14; sex: male, female; preparations: Ethanol; occurrenceID: EU_LIM_216; **Taxon:** scientificName: Dicranomyia (Idiopyga) melleicauda stenoptera Savchenko, 1970; family: Limoniidae; genus: Dicranomyia; subgenus: Idiopyga; specificEpithet: melleicauda; infraspecificEpithet: stenoptera; scientificNameAuthorship: Savchenko, 1970; **Location:** country: Norway; stateProvince: Finnmark; municipality: Porsanger; locality: S Stornes; verbatimElevation: 0.5 m; minimumElevationInMeters: 0.5; decimalLatitude: 70.19462; decimalLongitude: 24.91884; **Identification:** identifiedBy: J. Salmela; **Event:** samplingProtocol: Sweep net; eventDate: 2018-08-22; verbatimEventDate: 22/Aug/2018; **Record Level:** institutionCode: LMM; basisOfRecord: PreservedSpecimen
- **Type status:** Other material. **Occurrence:** catalogNumber: NVO.ins2018-97; occurrenceRemarks: 9 male+female; recordedBy: J. Salmela | Ø. Gammelmo | K.M. Olsen; individualCount: 9; sex: male, female; preparations: Ethanol; occurrenceID: EU_LIM_217; **Taxon:** scientificName: Dicranomyia (Idiopyga) melleicauda stenoptera Savchenko, 1970; family: Limoniidae; genus: Dicranomyia; subgenus: Idiopyga; specificEpithet: melleicauda; infraspecificEpithet: stenoptera; scientificNameAuthorship: Savchenko, 1970; **Location:** country: Norway; stateProvince: Finnmark; municipality: Porsanger; locality: Seines; verbatimElevation: 0.5 m; minimumElevationInMeters: 0.5; decimalLatitude: 70.08047; decimalLongitude: 24.94146; **Identification:** identifiedBy: J. Salmela; **Event:** samplingProtocol: Sweep net; eventDate: 2018-08-23; verbatimEventDate: 23/Aug/2018; **Record Level:** institutionCode: LMM; basisOfRecord: PreservedSpecimen
- **Type status:** Other material. **Occurrence:** catalogNumber: NVO.ins2018-98; occurrenceRemarks: 1 male; recordedBy: J. Salmela | Ø. Gammelmo | K.M. Olsen; individualCount: 1; sex: male; preparations: Ethanol; occurrenceID: EU_LIM_218; **Taxon:** scientificName: Dicranomyia (Idiopyga) melleicauda stenoptera Savchenko, 1970; family: Limoniidae; genus: Dicranomyia; subgenus: Idiopyga; specificEpithet: melleicauda; infraspecificEpithet: stenoptera; scientificNameAuthorship: Savchenko, 1970; **Location:** country: Norway; stateProvince: Finnmark; municipality: Porsanger; locality: Seines; verbatimElevation: 0.5 m; minimumElevationInMeters: 0.5; decimalLatitude: 70.08047; decimalLongitude: 24.94146; **Identification:** identifiedBy: J. Salmela; **Event:** samplingProtocol: Sweep net; eventDate: 2018-08-23; verbatimEventDate: 23/Aug/2018; **Record Level:** institutionCode: LMM; basisOfRecord: PreservedSpecimen
- **Type status:** Other material. **Occurrence:** catalogNumber: NVO.ins2018-163; occurrenceRemarks: 6 male+female; recordedBy: J. Salmela | Ø. Gammelmo | K.M. Olsen; individualCount: 6; sex: male, female; preparations: Ethanol; occurrenceID: EU_LIM_219; **Taxon:** scientificName: Dicranomyia (Idiopyga) melleicauda stenoptera Savchenko, 1970; family: Limoniidae; genus: Dicranomyia; subgenus: Idiopyga; specificEpithet: melleicauda; infraspecificEpithet: stenoptera; scientificNameAuthorship: Savchenko, 1970; **Location:** country: Norway; stateProvince: Finnmark; municipality: Porsanger; locality: Uhca Gáhtir; verbatimElevation: 0.5 m; minimumElevationInMeters: 0.5; decimalLatitude: 70.11393; decimalLongitude: 24.92069; **Identification:** identifiedBy: J. Salmela; **Event:** samplingProtocol: Sweep net; eventDate: 2018-08-22; verbatimEventDate: 22/Aug/2018; **Record Level:** institutionCode: LMM; basisOfRecord: PreservedSpecimen

#### Distribution

Presence of the species in Norway mentioned in Viitanen (2018) without further details; here, we publish the collection data of that record.

### Dicranomyia (Dicranomyia) mitis

(Meigen, 1830)

BAB5E4A5-C9E9-57DD-9F5E-8262845AC652

https://ccw.naturalis.nl/detail.php?id=8457

#### Materials

- **Type status:** Other material. **Occurrence:** occurrenceRemarks: 1 female; recordedBy: D.I. Gavryushin; individualCount: 1; sex: female; preparations: Ethanol; occurrenceID: EU_LIM_103; **Taxon:** scientificName: Dicranomyia (Dicranomyia) mitis (Meigen, 1830); family: Limoniidae; genus: Dicranomyia; subgenus: Dicranomyia; specificEpithet: mitis; scientificNameAuthorship: (Meigen, 1830); **Location:** country: Belarus; stateProvince: Vitebsk; county: Haradok; locality: Ezerische; decimalLatitude: 55.83; decimalLongitude: 30; **Identification:** identifiedBy: D.I. Gavryushin; **Event:** samplingProtocol: Sweep net; eventDate: 2019-05-16/2019-05-17; verbatimEventDate: 16-17/May/2019; **Record Level:** institutionCode: ZMMU; basisOfRecord: PreservedSpecimen
- **Type status:** Other material. **Occurrence:** occurrenceRemarks: 5 males, 6 females; recordedBy: D.I. Gavryushin; individualCount: 11; sex: male, female; occurrenceID: EU_LIM_104; **Taxon:** scientificName: Dicranomyia (Dicranomyia) mitis (Meigen, 1830); family: Limoniidae; genus: Dicranomyia; subgenus: Dicranomyia; specificEpithet: mitis; scientificNameAuthorship: (Meigen, 1830); **Location:** country: Russia; stateProvince: East European Russia; county: Bashkortostan Respublika; municipality: Beloretsk district; locality: Nura River (ca. 4km W of Otnurok village), at the foot of Zolotyie Shishki (Golden Cones) Mts.; verbatimElevation: 607 m; minimumElevationInMeters: 607; decimalLatitude: 54.05155; decimalLongitude: 58.26887; **Identification:** identifiedBy: D.I. Gavryushin; **Event:** samplingProtocol: Sweep net; eventDate: 2012-08-11; verbatimEventDate: 11/Aug/2012; **Record Level:** institutionCode: ZMMU; basisOfRecord: PreservedSpecimen
- **Type status:** Other material. **Occurrence:** occurrenceRemarks: 2 males; recordedBy: D.I. Gavryushin; individualCount: 2; sex: male; occurrenceID: EU_LIM_105; **Taxon:** scientificName: Dicranomyia (Dicranomyia) mitis (Meigen, 1830); family: Limoniidae; genus: Dicranomyia; subgenus: Dicranomyia; specificEpithet: mitis; scientificNameAuthorship: (Meigen, 1830); **Location:** country: Russia; stateProvince: East European Russia; county: Bashkortostan Respublika; municipality: Beloretsk district; locality: Nura River (ca. 4km W of Otnurok village), at the foot of Zolotyie Shishki (Golden Cones) Mts.; verbatimElevation: 607 m; minimumElevationInMeters: 607; decimalLatitude: 54.05155; decimalLongitude: 58.26887; **Identification:** identifiedBy: D.I. Gavryushin; **Event:** samplingProtocol: Sweep net; eventDate: 2012-08-09; verbatimEventDate: 09/Aug/2012; **Record Level:** institutionCode: ZMMU; basisOfRecord: PreservedSpecimen
- **Type status:** Other material. **Occurrence:** occurrenceRemarks: 1 female; recordedBy: D.I. Gavryushin; individualCount: 1; sex: female; occurrenceID: EU_LIM_106; **Taxon:** scientificName: Dicranomyia (Dicranomyia) mitis (Meigen, 1830); family: Limoniidae; genus: Dicranomyia; subgenus: Dicranomyia; specificEpithet: mitis; scientificNameAuthorship: (Meigen, 1830); **Location:** country: Russia; stateProvince: East European Russia; county: Bashkortostan Respublika; municipality: Beloretsk district; locality: Nura River (ca. 4km W of Otnurok village), at the foot of Zolotyie Shishki (Golden Cones) Mts.; verbatimElevation: 607 m; minimumElevationInMeters: 607; decimalLatitude: 54.05155; decimalLongitude: 58.26887; **Identification:** identifiedBy: D.I. Gavryushin; **Event:** samplingProtocol: Sweep net; eventDate: 2012-08-08; verbatimEventDate: 08/Aug/2012; **Record Level:** institutionCode: ZMMU; basisOfRecord: PreservedSpecimen
- **Type status:** Other material. **Occurrence:** occurrenceRemarks: 1 male; recordedBy: A. Polevoi; individualCount: 1; sex: male; preparations: Pinned; occurrenceID: EU_LIM_107; **Taxon:** scientificName: Dicranomyia (Dicranomyia) mitis (Meigen, 1830); family: Limoniidae; genus: Dicranomyia; subgenus: Dicranomyia; specificEpithet: mitis; scientificNameAuthorship: (Meigen, 1830); **Location:** country: Russia; stateProvince: North European Russia; county: Republic Karelia; municipality: Sortavala district; locality: Meijeri, 1 km S; verbatimElevation: 5 m; minimumElevationInMeters: 5; decimalLatitude: 61.61652; decimalLongitude: 30.59417; **Identification:** identifiedBy: A. Polevoi; **Event:** samplingProtocol: Sweep net; eventDate: 2015-06-12; verbatimEventDate: 12/Jun/2015; **Record Level:** institutionCode: FRIP; basisOfRecord: PreservedSpecimen

#### Distribution

First records from Belarus and Russia: RUE, RUN.

### Dicranomyia (Dicranomyia) modesta

(Meigen, 1818)

260ACE2F-8F6F-592E-98D8-84A16A7EE249

https://ccw.naturalis.nl/detail.php?id=8458

#### Materials

- **Type status:** Other material. **Occurrence:** occurrenceRemarks: 2 males, 1 female; recordedBy: L.-P. Kolcsár | E. Török; individualCount: 3; sex: male, female; preparations: Ethanol; occurrenceID: EU_LIM_108; **Taxon:** scientificName: Dicranomyia (Dicranomyia) modesta (Meigen, 1818); family: Limoniidae; genus: Dicranomyia; subgenus: Dicranomyia; specificEpithet: modesta; scientificNameAuthorship: (Meigen, 1818); **Location:** country: Albania; stateProvince: Korçë; municipality: Buqezë; locality: Ohrid Lake; verbatimElevation: 696 m; minimumElevationInMeters: 696; decimalLatitude: 41.04104; decimalLongitude: 20.63448; **Identification:** identifiedBy: L.-P. Kolcsár; **Event:** samplingProtocol: Sweep net; eventDate: 2017-06-01; verbatimEventDate: Jul-01-2017; **Record Level:** institutionCode: CKLP; basisOfRecord: PreservedSpecimen
- **Type status:** Other material. **Occurrence:** occurrenceRemarks: 2 females; recordedBy: D.I. Gavryushin; individualCount: 2; sex: female; preparations: Pinned; occurrenceID: EU_LIM_109; **Taxon:** scientificName: Dicranomyia (Dicranomyia) modesta (Meigen, 1818); family: Limoniidae; genus: Dicranomyia; subgenus: Dicranomyia; specificEpithet: modesta; scientificNameAuthorship: (Meigen, 1818); **Location:** country: Belarus; stateProvince: Minsk; county: Barysaw; locality: Vialikaje Stachava; verbatimElevation: 156 m; minimumElevationInMeters: 156; decimalLatitude: 54.26555; decimalLongitude: 28.38332; **Identification:** identifiedBy: D.I. Gavryushin; **Event:** samplingProtocol: Sweep net; eventDate: 2013-06-07; verbatimEventDate: 7/Jul/2013; **Record Level:** institutionCode: ZMMU; basisOfRecord: PreservedSpecimen
- **Type status:** Other material. **Occurrence:** occurrenceRemarks: 1 female; recordedBy: D.I. Gavryushin; individualCount: 1; sex: female; preparations: Ethanol; occurrenceID: EU_LIM_110; **Taxon:** scientificName: Dicranomyia (Dicranomyia) modesta (Meigen, 1818); family: Limoniidae; genus: Dicranomyia; subgenus: Dicranomyia; specificEpithet: modesta; scientificNameAuthorship: (Meigen, 1818); **Location:** country: Belarus; stateProvince: Gomel; county: Mazyr; locality: Mazyr; decimalLatitude: 52.02; decimalLongitude: 29.32; **Identification:** identifiedBy: D.I. Gavryushin; **Event:** samplingProtocol: Sweep net; eventDate: 2019-05-19/2019-05-21; verbatimEventDate: 19-21/May/2019; **Record Level:** institutionCode: ZMMU; basisOfRecord: PreservedSpecimen
- **Type status:** Other material. **Occurrence:** occurrenceRemarks: 2 males; recordedBy: D.I. Gavryushin; individualCount: 2; sex: male; preparations: Pinned; occurrenceID: EU_LIM_111; **Taxon:** scientificName: Dicranomyia (Dicranomyia) modesta (Meigen, 1818); family: Limoniidae; genus: Dicranomyia; subgenus: Dicranomyia; specificEpithet: modesta; scientificNameAuthorship: (Meigen, 1818); **Location:** country: Belarus; stateProvince: Gomel; county: Mazyr; locality: Mazyr; decimalLatitude: 52.02; decimalLongitude: 29.32; **Identification:** identifiedBy: D.I. Gavryushin; **Event:** samplingProtocol: Sweep net; eventDate: 2019-05-19/2019-05-21; verbatimEventDate: 19-21/May/2019; **Record Level:** institutionCode: ZMMU; basisOfRecord: PreservedSpecimen
- **Type status:** Other material. **Occurrence:** occurrenceRemarks: 1 male; recordedBy: D.I. Gavryushin; individualCount: 1; sex: male; preparations: Pinned; occurrenceID: EU_LIM_112; **Taxon:** scientificName: Dicranomyia (Dicranomyia) modesta (Meigen, 1818); family: Limoniidae; genus: Dicranomyia; subgenus: Dicranomyia; specificEpithet: modesta; scientificNameAuthorship: (Meigen, 1818); **Location:** country: Belarus; stateProvince: Gomel Region; county: Mazyr District; locality: Mazyr; decimalLatitude: 52.05; decimalLongitude: 29.31; **Identification:** identifiedBy: D.I. Gavryushin; **Event:** samplingProtocol: Sweep net; eventDate: 2019-06-11/2019-06-14; verbatimEventDate: 11-14/Jun/2019; **Record Level:** institutionCode: ZMMU; basisOfRecord: PreservedSpecimen
- **Type status:** Other material. **Occurrence:** occurrenceRemarks: 1 male; recordedBy: D.I. Gavryushin; individualCount: 1; sex: male; preparations: Pinned; occurrenceID: EU_LIM_113; **Taxon:** scientificName: Dicranomyia (Dicranomyia) modesta (Meigen, 1818); family: Limoniidae; genus: Dicranomyia; subgenus: Dicranomyia; specificEpithet: modesta; scientificNameAuthorship: (Meigen, 1818); **Location:** country: Belarus; stateProvince: Vitebsk; county: Orsha; locality: Dnieper River; decimalLatitude: 54.543; decimalLongitude: 30.463; **Identification:** identifiedBy: D.I. Gavryushin; **Event:** samplingProtocol: Sweep net; eventDate: 2017-06-11; verbatimEventDate: 11/Jun/2017; **Record Level:** institutionCode: ZMMU; basisOfRecord: PreservedSpecimen
- **Type status:** Other material. **Occurrence:** occurrenceRemarks: 4 males; recordedBy: A. McCluskey; individualCount: 4; sex: male; preparations: Pinned; occurrenceID: EU_LIM_114; **Taxon:** scientificName: Dicranomyia (Dicranomyia) modesta (Meigen, 1818); family: Limoniidae; genus: Dicranomyia; subgenus: Dicranomyia; specificEpithet: modesta; scientificNameAuthorship: (Meigen, 1818); **Location:** country: Iceland; locality: Skalanes, near Seydisfjordur; verbatimElevation: 15 m; minimumElevationInMeters: 15; decimalLatitude: 65.2937; decimalLongitude: -13.7034; **Identification:** identifiedBy: E. G. Hancock; **Event:** samplingProtocol: Malaise trap; eventDate: 2014-06-28; verbatimEventDate: 28/Jun/2014; **Record Level:** institutionCode: HMUG; basisOfRecord: PreservedSpecimen
- **Type status:** Other material. **Occurrence:** occurrenceRemarks: 2 males, 2 females; recordedBy: E.G. Hancock; individualCount: 4; sex: male, female; preparations: Pinned; occurrenceID: EU_LIM_115; **Taxon:** scientificName: Dicranomyia (Dicranomyia) modesta (Meigen, 1818); family: Limoniidae; genus: Dicranomyia; subgenus: Dicranomyia; specificEpithet: modesta; scientificNameAuthorship: (Meigen, 1818); **Location:** country: Iceland; locality: Skalanes, near Seydisfjordur; verbatimElevation: 15 m; minimumElevationInMeters: 15; decimalLatitude: 65.2937; decimalLongitude: -13.7034; **Identification:** identifiedBy: E. G. Hancock; **Event:** samplingProtocol: Malaise trap; eventDate: 2009-06-25; verbatimEventDate: 25/Jun/2009; **Record Level:** institutionCode: HMUG; basisOfRecord: PreservedSpecimen
- **Type status:** Other material. **Occurrence:** occurrenceRemarks: 3 males; recordedBy: D.I. Gavryushin; individualCount: 3; sex: male; occurrenceID: EU_LIM_116; **Taxon:** scientificName: Dicranomyia (Dicranomyia) modesta (Meigen, 1818); family: Limoniidae; genus: Dicranomyia; subgenus: Dicranomyia; specificEpithet: modesta; scientificNameAuthorship: (Meigen, 1818); **Location:** country: Russia; stateProvince: East European Russia; county: Bashkortostan Respublika; municipality: Beloretsk district; locality: Abzakovo env., Malyi Kizil River; verbatimElevation: 510 m; minimumElevationInMeters: 510; decimalLatitude: 53.81428; decimalLongitude: 58.5942; **Identification:** identifiedBy: D.I. Gavryushin; **Event:** samplingProtocol: Sweep net; eventDate: 2015-06-12; verbatimEventDate: 12/Jul/2015; **Record Level:** institutionCode: ZMMU; basisOfRecord: PreservedSpecimen
- **Type status:** Other material. **Occurrence:** occurrenceRemarks: 1 male; recordedBy: D.I. Gavryushin; individualCount: 1; sex: male; occurrenceID: EU_LIM_117; **Taxon:** scientificName: Dicranomyia (Dicranomyia) modesta (Meigen, 1818); family: Limoniidae; genus: Dicranomyia; subgenus: Dicranomyia; specificEpithet: modesta; scientificNameAuthorship: (Meigen, 1818); **Location:** country: Russia; stateProvince: East European Russia; county: Bashkortostan Respublika; municipality: Beloretsk district; locality: Abzakovo env., Karan River; verbatimElevation: 533 m; minimumElevationInMeters: 533; decimalLatitude: 53.83717; decimalLongitude: 58.57878; **Identification:** identifiedBy: D.I. Gavryushin; **Event:** samplingProtocol: Sweep net; eventDate: 2015-06-17; verbatimEventDate: 17/Jul/2015; **Record Level:** institutionCode: ZMMU; basisOfRecord: PreservedSpecimen
- **Type status:** Other material. **Occurrence:** occurrenceRemarks: 1 male; recordedBy: D.I. Gavryushin; individualCount: 1; sex: male; occurrenceID: EU_LIM_118; **Taxon:** scientificName: Dicranomyia (Dicranomyia) modesta (Meigen, 1818); family: Limoniidae; genus: Dicranomyia; subgenus: Dicranomyia; specificEpithet: modesta; scientificNameAuthorship: (Meigen, 1818); **Location:** country: Russia; stateProvince: East European Russia; county: Bashkortostan Respublika; municipality: Beloretsk district; locality: Abzakovo env., Karan River; verbatimElevation: 533 m; minimumElevationInMeters: 533; decimalLatitude: 53.83717; decimalLongitude: 58.57878; **Identification:** identifiedBy: D.I. Gavryushin; **Event:** samplingProtocol: Sweep net; eventDate: 2015-06-19; verbatimEventDate: 19/Jul/2015; **Record Level:** institutionCode: ZMMU; basisOfRecord: PreservedSpecimen
- **Type status:** Other material. **Occurrence:** occurrenceRemarks: 1 male; recordedBy: D.I. Gavryushin; individualCount: 1; sex: male; occurrenceID: EU_LIM_119; **Taxon:** scientificName: Dicranomyia (Dicranomyia) modesta (Meigen, 1818); family: Limoniidae; genus: Dicranomyia; subgenus: Dicranomyia; specificEpithet: modesta; scientificNameAuthorship: (Meigen, 1818); **Location:** country: Russia; stateProvince: East European Russia; county: Bashkortostan Respublika; municipality: Beloretsk district; locality: Abzakovo env., Kulsugady River; verbatimElevation: 531 m; minimumElevationInMeters: 531; decimalLatitude: 53.83795; decimalLongitude: 58.5823; **Identification:** identifiedBy: D.I. Gavryushin; **Event:** samplingProtocol: Sweep net; eventDate: 2012-08-15; verbatimEventDate: 15/Aug/2012; **Record Level:** institutionCode: ZMMU; basisOfRecord: PreservedSpecimen
- **Type status:** Other material. **Occurrence:** occurrenceRemarks: 2 males; recordedBy: D.I. Gavryushin; individualCount: 2; sex: male; occurrenceID: EU_LIM_120; **Taxon:** scientificName: Dicranomyia (Dicranomyia) modesta (Meigen, 1818); family: Limoniidae; genus: Dicranomyia; subgenus: Dicranomyia; specificEpithet: modesta; scientificNameAuthorship: (Meigen, 1818); **Location:** country: Russia; stateProvince: East European Russia; county: Bashkortostan Respublika; municipality: Uchaly district; locality: Ural-Tau St. env., upper reaches of Mindyak River; verbatimElevation: 765 m; minimumElevationInMeters: 765; decimalLatitude: 53.96525; decimalLongitude: 58.57888; **Identification:** identifiedBy: D.I. Gavryushin; **Event:** samplingProtocol: Sweep net; eventDate: 2015-06-09; verbatimEventDate: 09/Jul/2015; **Record Level:** institutionCode: ZMMU; basisOfRecord: PreservedSpecimen
- **Type status:** Other material. **Occurrence:** occurrenceRemarks: 1 female; recordedBy: D.I. Gavryushin; individualCount: 1; sex: female; occurrenceID: EU_LIM_121; **Taxon:** scientificName: Dicranomyia (Dicranomyia) modesta (Meigen, 1818); family: Limoniidae; genus: Dicranomyia; subgenus: Dicranomyia; specificEpithet: modesta; scientificNameAuthorship: (Meigen, 1818); **Location:** country: Russia; stateProvince: East European Russia; county: Bashkortostan Respublika; municipality: Uchaly district; locality: Ural-Tau St. env.; verbatimElevation: 777 m; minimumElevationInMeters: 777; decimalLatitude: 53.96805; decimalLongitude: 58.57613; **Identification:** identifiedBy: D.I. Gavryushin; **Event:** samplingProtocol: Sweep net; eventDate: 2015-06-09; verbatimEventDate: 09/Jul/2015; **Record Level:** institutionCode: ZMMU; basisOfRecord: PreservedSpecimen
- **Type status:** Other material. **Occurrence:** occurrenceRemarks: 2 males; recordedBy: D.I. Gavryushin; individualCount: 2; sex: male; occurrenceID: EU_LIM_122; **Taxon:** scientificName: Dicranomyia (Dicranomyia) modesta (Meigen, 1818); family: Limoniidae; genus: Dicranomyia; subgenus: Dicranomyia; specificEpithet: modesta; scientificNameAuthorship: (Meigen, 1818); **Location:** country: Russia; stateProvince: East European Russia; county: Bashkortostan Respublika; municipality: Uchaly district; locality: Kazakkulovo village env., Mindyak River; verbatimElevation: 505 m; minimumElevationInMeters: 505; decimalLatitude: 53.96983; decimalLongitude: 58.76983; **Identification:** identifiedBy: D.I. Gavryushin; **Event:** samplingProtocol: Sweep net; eventDate: 2012-08-12; verbatimEventDate: 12/Aug/2012; **Record Level:** institutionCode: ZMMU; basisOfRecord: PreservedSpecimen
- **Type status:** Other material. **Occurrence:** occurrenceRemarks: 1 female; recordedBy: D.I. Gavryushin; individualCount: 1; sex: female; occurrenceID: EU_LIM_123; **Taxon:** scientificName: Dicranomyia (Dicranomyia) modesta (Meigen, 1818); family: Limoniidae; genus: Dicranomyia; subgenus: Dicranomyia; specificEpithet: modesta; scientificNameAuthorship: (Meigen, 1818); **Location:** country: Russia; stateProvince: East European Russia; county: Bashkortostan Respublika; municipality: Beloretsk district; locality: Beloretsk env., Nura River; verbatimElevation: 494 m; minimumElevationInMeters: 494; decimalLatitude: 53.97365; decimalLongitude: 58.34415; **Identification:** identifiedBy: D.I. Gavryushin; **Event:** samplingProtocol: Sweep net; eventDate: 2012-08-10; verbatimEventDate: 10/Aug/2012; **Record Level:** institutionCode: ZMMU; basisOfRecord: PreservedSpecimen
- **Type status:** Other material. **Occurrence:** occurrenceRemarks: 3 males; recordedBy: D.I. Gavryushin; individualCount: 3; sex: male; occurrenceID: EU_LIM_124; **Taxon:** scientificName: Dicranomyia (Dicranomyia) modesta (Meigen, 1818); family: Limoniidae; genus: Dicranomyia; subgenus: Dicranomyia; specificEpithet: modesta; scientificNameAuthorship: (Meigen, 1818); **Location:** country: Russia; stateProvince: East European Russia; county: Bashkortostan Respublika; municipality: Beloretsk district; locality: Bolshoy Inzer River (ca. 6km NW of Ulu-Elga village); verbatimElevation: 505 m; minimumElevationInMeters: 505; decimalLatitude: 53.97375; decimalLongitude: 57.87472; **Identification:** identifiedBy: D.I. Gavryushin; **Event:** samplingProtocol: Sweep net; eventDate: 2012-08-14; verbatimEventDate: 14/Aug/2012; **Record Level:** institutionCode: ZMMU; basisOfRecord: PreservedSpecimen
- **Type status:** Other material. **Occurrence:** occurrenceRemarks: 2 males, 1 female; recordedBy: D.I. Gavryushin; individualCount: 3; sex: male, female; occurrenceID: EU_LIM_125; **Taxon:** scientificName: Dicranomyia (Dicranomyia) modesta (Meigen, 1818); family: Limoniidae; genus: Dicranomyia; subgenus: Dicranomyia; specificEpithet: modesta; scientificNameAuthorship: (Meigen, 1818); **Location:** country: Russia; stateProvince: East European Russia; county: Bashkortostan Respublika; municipality: Uchaly district; locality: Mindyak River (ca. 2km NW of [5]); verbatimElevation: 492 m; minimumElevationInMeters: 492; decimalLatitude: 53.9884; decimalLongitude: 58.81745; **Identification:** identifiedBy: D.I. Gavryushin; **Event:** samplingProtocol: Sweep net; eventDate: 2012-08-12; verbatimEventDate: 12/Aug/2012; **Record Level:** institutionCode: ZMMU; basisOfRecord: PreservedSpecimen
- **Type status:** Other material. **Occurrence:** occurrenceRemarks: 1 female; recordedBy: D.I. Gavryushin; individualCount: 1; sex: female; occurrenceID: EU_LIM_126; **Taxon:** scientificName: Dicranomyia (Dicranomyia) modesta (Meigen, 1818); family: Limoniidae; genus: Dicranomyia; subgenus: Dicranomyia; specificEpithet: modesta; scientificNameAuthorship: (Meigen, 1818); **Location:** country: Russia; stateProvince: East European Russia; county: Bashkortostan Respublika; municipality: Beloretsk district; locality: Nura River (ca. 4km W of Otnurok village), at the foot of Zolotyie Shishki (Golden Cones) Mts.; verbatimElevation: 607 m; minimumElevationInMeters: 607; decimalLatitude: 54.05155; decimalLongitude: 58.26887; **Identification:** identifiedBy: D.I. Gavryushin; **Event:** samplingProtocol: Sweep net; eventDate: 2012-08-08; verbatimEventDate: 08/Aug/2012; **Record Level:** institutionCode: ZMMU; basisOfRecord: PreservedSpecimen
- **Type status:** Other material. **Occurrence:** occurrenceRemarks: 1 female; recordedBy: D.I. Gavryushin; individualCount: 1; sex: female; occurrenceID: EU_LIM_127; **Taxon:** scientificName: Dicranomyia (Dicranomyia) modesta (Meigen, 1818); family: Limoniidae; genus: Dicranomyia; subgenus: Dicranomyia; specificEpithet: modesta; scientificNameAuthorship: (Meigen, 1818); **Location:** country: Russia; stateProvince: East European Russia; county: Bashkortostan Respublika; municipality: Beloretsk district; locality: Nura River (ca. 4km W of Otnurok village), at the foot of Zolotyie Shishki (Golden Cones) Mts.; verbatimElevation: 607 m; minimumElevationInMeters: 607; decimalLatitude: 54.05155; decimalLongitude: 58.26887; **Identification:** identifiedBy: D.I. Gavryushin; **Event:** samplingProtocol: Sweep net; eventDate: 2012-08-09; verbatimEventDate: 09/Aug/2012; **Record Level:** institutionCode: ZMMU; basisOfRecord: PreservedSpecimen
- **Type status:** Other material. **Occurrence:** occurrenceRemarks: 3 males; recordedBy: D.I. Gavryushin; individualCount: 3; sex: male; occurrenceID: EU_LIM_128; **Taxon:** scientificName: Dicranomyia (Dicranomyia) modesta (Meigen, 1818); family: Limoniidae; genus: Dicranomyia; subgenus: Dicranomyia; specificEpithet: modesta; scientificNameAuthorship: (Meigen, 1818); **Location:** country: Russia; stateProvince: East European Russia; county: Bashkortostan Respublika; municipality: Beloretsk district; locality: Nura River (ca. 4km W of Otnurok village), at the foot of Zolotyie Shishki (Golden Cones) Mts.; verbatimElevation: 607 m; minimumElevationInMeters: 607; decimalLatitude: 54.05155; decimalLongitude: 58.26887; **Identification:** identifiedBy: D.I. Gavryushin; **Event:** samplingProtocol: Sweep net; eventDate: 2012-08-11; verbatimEventDate: 11/Aug/2012; **Record Level:** institutionCode: ZMMU; basisOfRecord: PreservedSpecimen
- **Type status:** Other material. **Occurrence:** occurrenceRemarks: 2 males; recordedBy: D.I. Gavryushin; individualCount: 2; sex: male; occurrenceID: EU_LIM_129; **Taxon:** scientificName: Dicranomyia (Dicranomyia) modesta (Meigen, 1818); family: Limoniidae; genus: Dicranomyia; subgenus: Dicranomyia; specificEpithet: modesta; scientificNameAuthorship: (Meigen, 1818); **Location:** country: Russia; stateProvince: East European Russia; county: Bashkortostan Respublika; municipality: Beloretsk district; locality: Makhmutovo env., Belaya River; verbatimElevation: 550 m; minimumElevationInMeters: 550; decimalLatitude: 54.33012; decimalLongitude: 58.80735; **Identification:** identifiedBy: D.I. Gavryushin; **Event:** samplingProtocol: Sweep net; eventDate: 2015-06-15; verbatimEventDate: 15/Jul/2015; **Record Level:** institutionCode: ZMMU; basisOfRecord: PreservedSpecimen
- **Type status:** Other material. **Occurrence:** occurrenceRemarks: 1 male; recordedBy: N.G. Petrov; individualCount: 1; sex: male; occurrenceID: EU_LIM_130; **Taxon:** scientificName: Dicranomyia (Dicranomyia) modesta (Meigen, 1818); family: Limoniidae; genus: Dicranomyia; subgenus: Dicranomyia; specificEpithet: modesta; scientificNameAuthorship: (Meigen, 1818); **Location:** country: Russia; stateProvince: East European Russia; county: Tatarstan Respublika; municipality: Kazan; locality: district Derbyshki; verbatimElevation: 60 m; minimumElevationInMeters: 60; decimalLatitude: 55.8655; decimalLongitude: 49.22461; **Identification:** identifiedBy: N.M. Paramonov; **Event:** samplingProtocol: To light; eventDate: 2009-06-25; verbatimEventDate: 25/Jun/2009; **Record Level:** institutionCode: ZIN; basisOfRecord: PreservedSpecimen
- **Type status:** Other material. **Occurrence:** occurrenceRemarks: 2 males; recordedBy: N.M. Paramonov; individualCount: 2; sex: male; occurrenceID: EU_LIM_131; **Taxon:** scientificName: Dicranomyia (Dicranomyia) modesta (Meigen, 1818); family: Limoniidae; genus: Dicranomyia; subgenus: Dicranomyia; specificEpithet: modesta; scientificNameAuthorship: (Meigen, 1818); **Location:** country: Russia; stateProvince: East European Russia; county: Tatarstan Respublika; municipality: Laishevo district; locality: Volga-Kama State Nature Biosphere Reserve, «Saraly», Island Ornitologicheskiy; verbatimElevation: 50 m; minimumElevationInMeters: 50; decimalLatitude: 55.28392; decimalLongitude: 49.26081; **Identification:** identifiedBy: N.M. Paramonov; **Event:** samplingProtocol: Sweep net; eventDate: 2009-06-22/2009-06-23; verbatimEventDate: 22-23/Jun/2009; **Record Level:** institutionCode: ZIN; basisOfRecord: PreservedSpecimen
- **Type status:** Other material. **Occurrence:** occurrenceRemarks: 5 males; recordedBy: N.M. Paramonov; individualCount: 5; sex: male; occurrenceID: EU_LIM_132; **Taxon:** scientificName: Dicranomyia (Dicranomyia) modesta (Meigen, 1818); family: Limoniidae; genus: Dicranomyia; subgenus: Dicranomyia; specificEpithet: modesta; scientificNameAuthorship: (Meigen, 1818); **Location:** country: Russia; stateProvince: East European Russia; county: Tatarstan Respublika; municipality: Laishevo district; locality: Volga-Kama State Nature Biosphere Reserve, «Saraly»; verbatimElevation: 71 m; minimumElevationInMeters: 71; decimalLatitude: 55.29303; decimalLongitude: 49.29976; **Identification:** identifiedBy: N.M. Paramonov; **Event:** samplingProtocol: Sweep net; eventDate: 2009-06-19; verbatimEventDate: 19/Jun/2009; habitat: wetland; **Record Level:** institutionCode: ZIN; basisOfRecord: PreservedSpecimen
- **Type status:** Other material. **Occurrence:** occurrenceRemarks: 1 male; recordedBy: N.M. Paramonov; individualCount: 1; sex: male; occurrenceID: EU_LIM_133; **Taxon:** scientificName: Dicranomyia (Dicranomyia) modesta (Meigen, 1818); family: Limoniidae; genus: Dicranomyia; subgenus: Dicranomyia; specificEpithet: modesta; scientificNameAuthorship: (Meigen, 1818); **Location:** country: Russia; stateProvince: East European Russia; county: Tatarstan Respublika; municipality: Laishevo district; locality: Volga-Kama State Nature Biosphere Reserve, «Saraly»; verbatimElevation: 71 m; minimumElevationInMeters: 71; decimalLatitude: 55.29303; decimalLongitude: 49.29976; **Identification:** identifiedBy: N.M. Paramonov; **Event:** samplingProtocol: Sweep net; eventDate: 2009-06-24; verbatimEventDate: 24/Jun/2009; habitat: wetland; **Record Level:** institutionCode: ZIN; basisOfRecord: PreservedSpecimen
- **Type status:** Other material. **Occurrence:** occurrenceRemarks: 5 males; recordedBy: N.M. Paramonov; individualCount: 5; sex: male; occurrenceID: EU_LIM_134; **Taxon:** scientificName: Dicranomyia (Dicranomyia) modesta (Meigen, 1818); family: Limoniidae; genus: Dicranomyia; subgenus: Dicranomyia; specificEpithet: modesta; scientificNameAuthorship: (Meigen, 1818); **Location:** country: Russia; stateProvince: East European Russia; county: Tatarstan Respublika; municipality: Verhneuslonsk district; locality: base “Zoostation”, 3,5 km NW Pustye Morkvashi env.; verbatimElevation: 80 m; minimumElevationInMeters: 80; decimalLatitude: 55.47005; decimalLongitude: 48.44092; **Identification:** identifiedBy: N.M. Paramonov; **Event:** samplingProtocol: Sweep net; eventDate: 2013-08-22/2013-08-26; verbatimEventDate: 22-26/Aug/2013; habitat: ravine, wetland; **Record Level:** institutionCode: ZIN; basisOfRecord: PreservedSpecimen
- **Type status:** Other material. **Occurrence:** occurrenceRemarks: 1 male; recordedBy: N.M. Paramonov; individualCount: 1; sex: male; occurrenceID: EU_LIM_135; **Taxon:** scientificName: Dicranomyia (Dicranomyia) modesta (Meigen, 1818); family: Limoniidae; genus: Dicranomyia; subgenus: Dicranomyia; specificEpithet: modesta; scientificNameAuthorship: (Meigen, 1818); **Location:** country: Russia; stateProvince: East European Russia; county: Tatarstan Respublika; municipality: Zelenodol’sk district; locality: Zaymishche env., Geomagnetic station; verbatimElevation: 87 m; minimumElevationInMeters: 87; decimalLatitude: 55.82684; decimalLongitude: 48.84395; **Identification:** identifiedBy: N.M. Paramonov; **Event:** samplingProtocol: Sweep net; eventDate: 2011-08-19/2011-08-21; verbatimEventDate: 19-21/Aug/2011; **Record Level:** institutionCode: ZIN; basisOfRecord: PreservedSpecimen
- **Type status:** Other material. **Occurrence:** occurrenceRemarks: 1 male; recordedBy: N.M. Paramonov; individualCount: 1; sex: male; occurrenceID: EU_LIM_136; **Taxon:** scientificName: Dicranomyia (Dicranomyia) modesta (Meigen, 1818); family: Limoniidae; genus: Dicranomyia; subgenus: Dicranomyia; specificEpithet: modesta; scientificNameAuthorship: (Meigen, 1818); **Location:** country: Russia; stateProvince: East European Russia; county: Tatarstan Respublika; municipality: Zelenodol’sk district; locality: Zaymishche env., Geomagnetic station; verbatimElevation: 87 m; minimumElevationInMeters: 87; decimalLatitude: 55.82684; decimalLongitude: 48.84395; **Identification:** identifiedBy: N.M. Paramonov; **Event:** samplingProtocol: Sweep net; eventDate: 2012-06-30; verbatimEventDate: 30/Jun/2012; **Record Level:** institutionCode: ZIN; basisOfRecord: PreservedSpecimen
- **Type status:** Other material. **Occurrence:** occurrenceRemarks: 1 male; recordedBy: N.M. Paramonov; individualCount: 1; sex: male; occurrenceID: EU_LIM_137; **Taxon:** scientificName: Dicranomyia (Dicranomyia) modesta (Meigen, 1818); family: Limoniidae; genus: Dicranomyia; subgenus: Dicranomyia; specificEpithet: modesta; scientificNameAuthorship: (Meigen, 1818); **Location:** country: Russia; stateProvince: East European Russia; county: Tatarstan Respublika; municipality: Zelenodol’sk district; locality: Ilinskoe; verbatimElevation: 90 m; minimumElevationInMeters: 90; decimalLatitude: 55.87455; decimalLongitude: 48.68579; **Identification:** identifiedBy: N.M. Paramonov; **Event:** samplingProtocol: Sweep net; eventDate: 2012-06-29; verbatimEventDate: 29/Jun/2012; habitat: village env.; **Record Level:** institutionCode: ZIN; basisOfRecord: PreservedSpecimen
- **Type status:** Other material. **Occurrence:** occurrenceRemarks: 1 male; recordedBy: N.M. Paramonov; individualCount: 1; sex: male; occurrenceID: EU_LIM_138; **Taxon:** scientificName: Dicranomyia (Dicranomyia) modesta (Meigen, 1818); family: Limoniidae; genus: Dicranomyia; subgenus: Dicranomyia; specificEpithet: modesta; scientificNameAuthorship: (Meigen, 1818); **Location:** country: Russia; stateProvince: East European Russia; county: Tatarstan Respublika; municipality: Zelenodol’sk district; locality: Volga-Kama State Nature Biosphere Reserve, «Raifa», Serbulak River; verbatimElevation: 100 m; minimumElevationInMeters: 100; decimalLatitude: 55.88868; decimalLongitude: 48.71434; **Identification:** identifiedBy: N.M. Paramonov; **Event:** samplingProtocol: Sweep net; eventDate: 2009-06-14; verbatimEventDate: 14/Jun/2009; **Record Level:** institutionCode: ZIN; basisOfRecord: PreservedSpecimen
- **Type status:** Other material. **Occurrence:** occurrenceRemarks: 1 male; recordedBy: N.M. Paramonov; individualCount: 1; sex: male; occurrenceID: EU_LIM_139; **Taxon:** scientificName: Dicranomyia (Dicranomyia) modesta (Meigen, 1818); family: Limoniidae; genus: Dicranomyia; subgenus: Dicranomyia; specificEpithet: modesta; scientificNameAuthorship: (Meigen, 1818); **Location:** country: Russia; stateProvince: East European Russia; county: Tatarstan Respublika; municipality: Zelenodol’sk district; locality: Volga-Kama State Nature Biosphere Reserve, «Raifa», Lake Lenevo; verbatimElevation: 80 m; minimumElevationInMeters: 80; decimalLatitude: 55.90433; decimalLongitude: 48.79115; **Identification:** identifiedBy: N.M. Paramonov; **Event:** samplingProtocol: Sweep net; eventDate: 2012-06-26; verbatimEventDate: 26/Jun/2012; **Record Level:** institutionCode: ZIN; basisOfRecord: PreservedSpecimen
- **Type status:** Other material. **Occurrence:** occurrenceRemarks: 2 males; recordedBy: N.M. Paramonov; individualCount: 2; sex: male; occurrenceID: EU_LIM_140; **Taxon:** scientificName: Dicranomyia (Dicranomyia) modesta (Meigen, 1818); family: Limoniidae; genus: Dicranomyia; subgenus: Dicranomyia; specificEpithet: modesta; scientificNameAuthorship: (Meigen, 1818); **Location:** country: Russia; stateProvince: East European Russia; county: Tatarstan Respublika; municipality: Zelenodol’sk district; locality: Sumka River, Lake Raifskoe; verbatimElevation: 75 m; minimumElevationInMeters: 75; decimalLatitude: 55.91237; decimalLongitude: 48.73159; **Identification:** identifiedBy: N.M. Paramonov; **Event:** samplingProtocol: Sweep net; eventDate: 2012-06-27; verbatimEventDate: 27/Jun/2012; **Record Level:** institutionCode: ZIN; basisOfRecord: PreservedSpecimen
- **Type status:** Other material. **Occurrence:** occurrenceRemarks: 3 males, 1 female; recordedBy: N.M. Paramonov; individualCount: 4; sex: male, female; occurrenceID: EU_LIM_141; **Taxon:** scientificName: Dicranomyia (Dicranomyia) modesta (Meigen, 1818); family: Limoniidae; genus: Dicranomyia; subgenus: Dicranomyia; specificEpithet: modesta; scientificNameAuthorship: (Meigen, 1818); **Location:** country: Russia; stateProvince: East European Russia; county: Tatarstan Respublika; municipality: Zelenodol’sk district; locality: Volga-Kama State Nature Biosphere Reserve, «Raifa», Sumka River, Belo-Bezvodnoe village env.; verbatimElevation: 75 m; minimumElevationInMeters: 75; decimalLatitude: 55.92183; decimalLongitude: 48.75462; **Identification:** identifiedBy: N.M. Paramonov; **Event:** samplingProtocol: Sweep net; eventDate: 2012-06-28; verbatimEventDate: 28/Jun/2012; **Record Level:** institutionCode: ZIN; basisOfRecord: PreservedSpecimen

#### Distribution

First records from Albania, Belarus, Iceland and Russia: RUE.

### Dicranomyia (Melanolimonia) morio

(Fabricius, 1787)

0344253C-FC3D-51CC-9462-05894CCD56CE

https://ccw.naturalis.nl/detail.php?id=8990

#### Materials

- **Type status:** Other material. **Occurrence:** occurrenceRemarks: 1 male; recordedBy: L.-P. Kolcsár | E. Török; individualCount: 1; sex: male; preparations: Ethanol; occurrenceID: EU_LIM_225; **Taxon:** scientificName: Dicranomyia (Melanolimonia) morio (Fabricius, 1787); family: Limoniidae; genus: Dicranomyia; subgenus: Melanolimonia; specificEpithet: morio; scientificNameAuthorship: (Fabricius, 1787); **Location:** country: Albania; stateProvince: Korçë; municipality: Buqezë; locality: Ohrid Lake; verbatimElevation: 696 m; minimumElevationInMeters: 696; decimalLatitude: 41.04104; decimalLongitude: 20.63448; **Identification:** identifiedBy: L.-P. Kolcsár; **Event:** samplingProtocol: Sweep net; eventDate: 2017-06-01; verbatimEventDate: Jul-01-2017; **Record Level:** institutionCode: CKLP; basisOfRecord: PreservedSpecimen
- **Type status:** Other material. **Occurrence:** occurrenceRemarks: 1 male; recordedBy: L.-P. Kolcsár | E. Török; individualCount: 1; sex: male; preparations: Ethanol; occurrenceID: EU_LIM_226; **Taxon:** scientificName: Dicranomyia (Melanolimonia) morio (Fabricius, 1787); family: Limoniidae; genus: Dicranomyia; subgenus: Melanolimonia; specificEpithet: morio; scientificNameAuthorship: (Fabricius, 1787); **Location:** country: Albania; stateProvince: Vlorë; municipality: Ilias; locality: Gjipe Canyon; verbatimElevation: 10 m; minimumElevationInMeters: 10; decimalLatitude: 40.12771; decimalLongitude: 19.67168; **Identification:** identifiedBy: L.-P. Kolcsár; **Event:** samplingProtocol: Sweep net; eventDate: 2017-06-29; verbatimEventDate: 29/Jun/2017; **Record Level:** institutionCode: CKLP; basisOfRecord: PreservedSpecimen
- **Type status:** Other material. **Occurrence:** occurrenceRemarks: 1 male; recordedBy: L.-P. Kolcsár | E. Török; individualCount: 1; sex: male; preparations: Ethanol; occurrenceID: EU_LIM_227; **Taxon:** scientificName: Dicranomyia (Melanolimonia) morio (Fabricius, 1787); family: Limoniidae; genus: Dicranomyia; subgenus: Melanolimonia; specificEpithet: morio; scientificNameAuthorship: (Fabricius, 1787); **Location:** country: North Macedonia; municipality: Kolari; locality: Bistra Mts., Straza pass; verbatimElevation: 1219 m; minimumElevationInMeters: 1219; decimalLatitude: 41.66974; decimalLongitude: 20.85049; **Identification:** identifiedBy: L.-P. Kolcsár; **Event:** samplingProtocol: Sweep net; eventDate: 2017-06-01; verbatimEventDate: 1/Jul/2017; **Record Level:** institutionCode: CKLP; basisOfRecord: PreservedSpecimen
- **Type status:** Other material. **Occurrence:** occurrenceRemarks: 1 male; recordedBy: D.I. Gavryushin; individualCount: 1; sex: male; occurrenceID: EU_LIM_228; **Taxon:** scientificName: Dicranomyia (Melanolimonia) morio (Fabricius, 1787); family: Limoniidae; genus: Dicranomyia; subgenus: Melanolimonia; specificEpithet: morio; scientificNameAuthorship: (Fabricius, 1787); **Location:** country: Russia; stateProvince: East European Russia; county: Bashkortostan Respublika; municipality: Beloretsk district; locality: Abzakovo env., Kulsugady River; verbatimElevation: 531 m; minimumElevationInMeters: 531; decimalLatitude: 53.83795; decimalLongitude: 58.5823; **Identification:** identifiedBy: D.I. Gavryushin; **Event:** samplingProtocol: Sweep net; eventDate: 2012-08-15; verbatimEventDate: 15/Aug/2012; **Record Level:** institutionCode: ZMMU; basisOfRecord: PreservedSpecimen
- **Type status:** Other material. **Occurrence:** occurrenceRemarks: 2 males; recordedBy: N.M. Paramonov; individualCount: 2; sex: male; occurrenceID: EU_LIM_229; **Taxon:** scientificName: Dicranomyia (Melanolimonia) morio (Fabricius, 1787); family: Limoniidae; genus: Dicranomyia; subgenus: Melanolimonia; specificEpithet: morio; scientificNameAuthorship: (Fabricius, 1787); **Location:** country: Russia; stateProvince: East European Russia; county: Tatarstan Respublika; municipality: Bavlinsk district; locality: Hansverkino village env., Verhnii Kandiz River; verbatimElevation: 142 m; minimumElevationInMeters: 142; decimalLatitude: 54.02989; decimalLongitude: 53.22563; **Identification:** identifiedBy: N.M. Paramonov; **Event:** samplingProtocol: Sweep net; eventDate: 2013-05-09/2013-05-10; verbatimEventDate: 9-10/May/2013; habitat: spring; **Record Level:** institutionCode: ZIN; basisOfRecord: PreservedSpecimen
- **Type status:** Other material. **Occurrence:** occurrenceRemarks: 1 male; recordedBy: D.I. Gavryushin; individualCount: 1; sex: male; preparations: Pinned; occurrenceID: EU_LIM_230; **Taxon:** scientificName: Dicranomyia (Melanolimonia) morio (Fabricius, 1787); family: Limoniidae; genus: Dicranomyia; subgenus: Melanolimonia; specificEpithet: morio; scientificNameAuthorship: (Fabricius, 1787); **Location:** country: Serbia; stateProvince: Zaječar; municipality: Knjaževac; locality: Crni Vrh; verbatimElevation: 800 m; minimumElevationInMeters: 800; decimalLatitude: 43.407; decimalLongitude: 22.587; **Identification:** identifiedBy: D.I. Gavryushin; **Event:** samplingProtocol: Sweep net; eventDate: 2014-09-16/2014-09-18; verbatimEventDate: 16-22/Sep/2014; **Record Level:** institutionCode: ZMMU; basisOfRecord: PreservedSpecimen
- **Type status:** Other material. **Occurrence:** occurrenceRemarks: 7 males; recordedBy: D.I. Gavryushin; individualCount: 7; sex: male; preparations: Pinned; occurrenceID: EU_LIM_231; **Taxon:** scientificName: Dicranomyia (Melanolimonia) morio (Fabricius, 1787); family: Limoniidae; genus: Dicranomyia; subgenus: Melanolimonia; specificEpithet: morio; scientificNameAuthorship: (Fabricius, 1787); **Location:** country: Serbia; stateProvince: Zaječar; municipality: Knjaževac; locality: Crni Vrh; verbatimElevation: 800 m; minimumElevationInMeters: 800; decimalLatitude: 43.407; decimalLongitude: 22.587; **Identification:** identifiedBy: D.I. Gavryushin; **Event:** samplingProtocol: Sweep net; eventDate: 2015-05-01/2015-05-08; verbatimEventDate: 01-08/May/2015; **Record Level:** institutionCode: ZMMU; basisOfRecord: PreservedSpecimen
- **Type status:** Other material. **Occurrence:** occurrenceRemarks: 1 male, 2 females; recordedBy: D.I. Gavryushin; individualCount: 3; sex: male, female; preparations: Pinned; occurrenceID: EU_LIM_232; **Taxon:** scientificName: Dicranomyia (Melanolimonia) morio (Fabricius, 1787); family: Limoniidae; genus: Dicranomyia; subgenus: Melanolimonia; specificEpithet: morio; scientificNameAuthorship: (Fabricius, 1787); **Location:** country: Serbia; stateProvince: Zaječar; municipality: Knjaževac; locality: Kalna, Timok River; decimalLatitude: 43.42; decimalLongitude: 22.42; **Identification:** identifiedBy: D.I. Gavryushin; **Event:** samplingProtocol: Sweep net; eventDate: 2015-06-01/2015-07-07; verbatimEventDate: 01-07/Jul/2015; **Record Level:** institutionCode: ZMMU; basisOfRecord: PreservedSpecimen
- **Type status:** Other material. **Occurrence:** occurrenceRemarks: 1 female; recordedBy: D.I. Gavryushin; individualCount: 1; sex: female; preparations: Pinned; occurrenceID: EU_LIM_233; **Taxon:** scientificName: Dicranomyia (Melanolimonia) morio (Fabricius, 1787); family: Limoniidae; genus: Dicranomyia; subgenus: Melanolimonia; specificEpithet: morio; scientificNameAuthorship: (Fabricius, 1787); **Location:** country: Serbia; locality: Stara Planina Mts., Babin Zub Mountain; verbatimElevation: 1550 m; minimumElevationInMeters: 1550; decimalLatitude: 43.375; decimalLongitude: 22.625; **Identification:** identifiedBy: D.I. Gavryushin; **Event:** samplingProtocol: Sweep net; eventDate: 2015-06-01/2015-07-07; verbatimEventDate: 01-07/Jul/2015; **Record Level:** institutionCode: ZMMU; basisOfRecord: PreservedSpecimen

#### Distribution

First records from Albania, North Macedonia, Russia: RUE and Serbia.

### Dicranomyia (Idiopyga) nigristigma

Nielsen, 1919

DBFC9434-B3D2-5CC0-A7E9-A4796BB55013

https://ccw.naturalis.nl/detail.php?id=8958

#### Materials

- **Type status:** Other material. **Occurrence:** catalogNumber: 530925, 644168; occurrenceRemarks: 2 male+female; recordedBy: K.M. Olsen; individualCount: 2; sex: male, female; preparations: Ethanol; occurrenceID: EU_LIM_220; **Taxon:** scientificName: Dicranomyia (Idiopyga) nigristigma Nielsen, 1919; family: Limoniidae; genus: Dicranomyia; subgenus: Idiopyga; specificEpithet: nigristigma; scientificNameAuthorship: Nielsen, 1919; **Location:** country: Norway; stateProvince: Akershus; municipality: Skedsmo; locality: SSE Buhaugen; verbatimElevation: 110 m; minimumElevationInMeters: 110; decimalLatitude: 59.97227; decimalLongitude: 10.98641; **Identification:** identifiedBy: K.M. Olsen; **Event:** samplingProtocol: Sweep net; eventDate: 2017-09-07; verbatimEventDate: 07/Sep/2017; **Record Level:** institutionCode: PCKMO; basisOfRecord: PreservedSpecimen

#### Distribution

First record from Norway.

### Dicranomyia (Dicranomyia) omissinervis

de Meijere, 1918

46951FE5-E4BF-5181-8CD3-4ABD70C8F80E

https://ccw.naturalis.nl/detail.php?id=8516

#### Materials

- **Type status:** Other material. **Occurrence:** occurrenceRemarks: 1 male; recordedBy: D.I. Gavryushin; individualCount: 1; sex: male; occurrenceID: EU_LIM_142; **Taxon:** scientificName: Dicranomyia (Dicranomyia) omissinervis de Meijere, 1918; family: Limoniidae; genus: Dicranomyia; subgenus: Dicranomyia; specificEpithet: omissinervis; scientificNameAuthorship: de Meijere, 1918; **Location:** country: Russia; stateProvince: East European Russia; county: Bashkortostan Respublika; municipality: Beloretsk district; locality: Makhmutovo env., Belaya River; verbatimElevation: 550 m; minimumElevationInMeters: 550; decimalLatitude: 54.33012; decimalLongitude: 58.80735; **Identification:** identifiedBy: D.I. Gavryushin; **Event:** samplingProtocol: Sweep net; eventDate: 2015-06-15; verbatimEventDate: Jul-15-2015; **Record Level:** institutionCode: ZMMU; basisOfRecord: PreservedSpecimen

#### Distribution

First record from Russia: RUE.

### Dicranomyia (Dicranomyia) patricia

Starý, 1982

F718CDFE-249C-5D56-B19D-B434066ECB50

https://ccw.naturalis.nl/detail.php?id=8541

#### Materials

- **Type status:** Other material. **Occurrence:** occurrenceRemarks: 2 males; recordedBy: J. Starý; individualCount: 2; sex: male; preparations: Pinned; occurrenceID: EU_LIM_143; **Taxon:** scientificName: Dicranomyia (Dicranomyia) patricia Starý, 1982; family: Limoniidae; genus: Dicranomyia; subgenus: Dicranomyia; specificEpithet: patricia; scientificNameAuthorship: Starý, 1982; **Location:** island: Sicily; country: Italy; stateProvince: Sicily; municipality: Ucria; locality: 0.9 km S, torrente Praculla; verbatimElevation: 620 m; minimumElevationInMeters: 620; decimalLatitude: 38.03833; decimalLongitude: 14.88333; **Identification:** identifiedBy: J. Starý; **Event:** eventDate: 2016-04-27; verbatimEventDate: 27/Apr/2016; **Record Level:** institutionCode: PCJS; basisOfRecord: PreservedSpecimen
- **Type status:** Other material. **Occurrence:** occurrenceRemarks: 1 male; recordedBy: J. Starý; individualCount: 1; sex: male; preparations: Pinned; occurrenceID: EU_LIM_144; **Taxon:** scientificName: Dicranomyia (Dicranomyia) patricia Starý, 1982; family: Limoniidae; genus: Dicranomyia; subgenus: Dicranomyia; specificEpithet: patricia; scientificNameAuthorship: Starý, 1982; **Location:** island: Sicily; country: Italy; stateProvince: Sicily; municipality: Raccuja; locality: 0.9 km W, Fiumara di Sinagra; verbatimElevation: 450 m; minimumElevationInMeters: 450; decimalLatitude: 38.05389; decimalLongitude: 14.90083; **Identification:** identifiedBy: J. Starý; **Event:** eventDate: 2016-04-19; verbatimEventDate: 19/Apr/2016; **Record Level:** institutionCode: PCJS; basisOfRecord: PreservedSpecimen

#### Distribution

First records from Italy (from Sicily).

### Dicranomyia (Dicranomyia) quadra

(Meigen, 1838)

773C5791-FD3C-57CA-BF89-1E7426039F97

https://ccw.naturalis.nl/detail.php?id=8610

#### Materials

- **Type status:** Other material. **Occurrence:** occurrenceRemarks: 2 males; recordedBy: L.-P. Kolcsár | E. Török; individualCount: 2; sex: male; preparations: Ethanol; occurrenceID: EU_LIM_145; **Taxon:** scientificName: Dicranomyia (Dicranomyia) quadra (Meigen, 1838); family: Limoniidae; genus: Dicranomyia; subgenus: Dicranomyia; specificEpithet: quadra; scientificNameAuthorship: (Meigen, 1838); **Location:** country: North Macedonia; municipality: Ljubanishta; locality: monastery of St Naum; verbatimElevation: 698 m; minimumElevationInMeters: 698; decimalLatitude: 40.91242; decimalLongitude: 20.74367; **Identification:** identifiedBy: J. Starý; **Event:** samplingProtocol: Sweep net; eventDate: 2012-05-04; verbatimEventDate: May-04-2012; **Record Level:** institutionCode: PCJS; basisOfRecord: PreservedSpecimen
- **Type status:** Other material. **Occurrence:** catalogNumber: 581914; occurrenceRemarks: 1 male; recordedBy: K.M. Olsen; individualCount: 1; sex: male; preparations: Ethanol; occurrenceID: EU_LIM_146; **Taxon:** scientificName: Dicranomyia (Dicranomyia) quadra (Meigen, 1838); family: Limoniidae; genus: Dicranomyia; subgenus: Dicranomyia; specificEpithet: quadra; scientificNameAuthorship: (Meigen, 1838); **Location:** country: Norway; stateProvince: Akershus; municipality: Ski; locality: Kapelldammen; verbatimElevation: 125 m; minimumElevationInMeters: 125; decimalLatitude: 59.72433; decimalLongitude: 10.8386; **Identification:** identifiedBy: K.M. Olsen; **Event:** samplingProtocol: Sweep net; eventDate: 2018-06-12; verbatimEventDate: 12/Jun/2018; habitat: Rundt hele dammen; **Record Level:** institutionCode: PCKMO; basisOfRecord: PreservedSpecimen
- **Type status:** Other material. **Occurrence:** catalogNumber: 611595; occurrenceRemarks: 1 male; recordedBy: S. Svendsen; individualCount: 1; sex: male; preparations: Ethanol; occurrenceID: EU_LIM_147; **Taxon:** scientificName: Dicranomyia (Dicranomyia) quadra (Meigen, 1838); family: Limoniidae; genus: Dicranomyia; subgenus: Dicranomyia; specificEpithet: quadra; scientificNameAuthorship: (Meigen, 1838); **Location:** country: Norway; stateProvince: Aust-Agder; municipality: Birkenes; locality: Nordåsen; verbatimElevation: 85 m; minimumElevationInMeters: 85; decimalLatitude: 58.33342; decimalLongitude: 8.24004; **Identification:** identifiedBy: K.M. Olsen; **Event:** samplingProtocol: Light trap; eventDate: 2016-06/2016-08; verbatimEventDate: Jul-Aug/2016; **Record Level:** institutionCode: PCKMO; basisOfRecord: PreservedSpecimen
- **Type status:** Other material. **Occurrence:** catalogNumber: 641502; occurrenceRemarks: 1 male; recordedBy: E.S. Paulsen | S. Apeland | L.T. Bjørnø; individualCount: 1; sex: male; preparations: Ethanol; occurrenceID: EU_LIM_148; **Taxon:** scientificName: Dicranomyia (Dicranomyia) quadra (Meigen, 1838); family: Limoniidae; genus: Dicranomyia; subgenus: Dicranomyia; specificEpithet: quadra; scientificNameAuthorship: (Meigen, 1838); **Location:** country: Norway; stateProvince: Rogaland; municipality: Suldal; locality: Tengesdal – Slettå; verbatimElevation: 65 m; minimumElevationInMeters: 65; decimalLatitude: 59.55973; decimalLongitude: 6.47109; **Identification:** identifiedBy: K.M. Olsen; **Event:** samplingProtocol: Malaise trap; eventDate: 2019-06-09/2019-07-02; verbatimEventDate: 09/Jun-02/Jul/2019; **Record Level:** institutionCode: NHMO; basisOfRecord: PreservedSpecimen
- **Type status:** Other material. **Occurrence:** catalogNumber: 649186; occurrenceRemarks: 1 male; recordedBy: E.S. Paulsen | S. Apeland | L.T. Bjørnø; individualCount: 1; sex: male; preparations: Ethanol; occurrenceID: EU_LIM_149; **Taxon:** scientificName: Dicranomyia (Dicranomyia) quadra (Meigen, 1838); family: Limoniidae; genus: Dicranomyia; subgenus: Dicranomyia; specificEpithet: quadra; scientificNameAuthorship: (Meigen, 1838); **Location:** country: Norway; stateProvince: Rogaland; municipality: Suldal; locality: Tengesdal – Slettå; verbatimElevation: 65 m; minimumElevationInMeters: 65; decimalLatitude: 59.55973; decimalLongitude: 6.47109; **Identification:** identifiedBy: K.M. Olsen; **Event:** samplingProtocol: Malaise trap; eventDate: 2019-06-02/2019-07-21; verbatimEventDate: 02-21/Jul/2019; **Record Level:** institutionCode: NHMO; basisOfRecord: PreservedSpecimen
- **Type status:** Other material. **Occurrence:** catalogNumber: 553766; occurrenceRemarks: 1 male; recordedBy: K. Berggren | R.-A. Golf; individualCount: 1; sex: male; preparations: Ethanol; occurrenceID: EU_LIM_150; **Taxon:** scientificName: Dicranomyia (Dicranomyia) quadra (Meigen, 1838); family: Limoniidae; genus: Dicranomyia; subgenus: Dicranomyia; specificEpithet: quadra; scientificNameAuthorship: (Meigen, 1838); **Location:** country: Norway; stateProvince: Sogn og Fjordane; municipality: Lærdal; locality: Sløgrandane – NE Nygard; verbatimElevation: 70 m; minimumElevationInMeters: 70; decimalLatitude: 61.04407; decimalLongitude: 7.62354; **Identification:** identifiedBy: K.M. Olsen; **Event:** samplingProtocol: Light trap; eventDate: 2017-06; verbatimEventDate: Jul/2017; **Record Level:** institutionCode: PCKMO; basisOfRecord: PreservedSpecimen
- **Type status:** Other material. **Occurrence:** catalogNumber: 549429; occurrenceRemarks: 1 male; recordedBy: K.M. Olsen; individualCount: 1; sex: male; preparations: Ethanol; occurrenceID: EU_LIM_151; **Taxon:** scientificName: Dicranomyia (Dicranomyia) quadra (Meigen, 1838); family: Limoniidae; genus: Dicranomyia; subgenus: Dicranomyia; specificEpithet: quadra; scientificNameAuthorship: (Meigen, 1838); **Location:** country: Norway; stateProvince: Sogn og Fjordane; municipality: Lærdal; locality: Storøyni; verbatimElevation: 185 m; minimumElevationInMeters: 185; decimalLatitude: 61.05269; decimalLongitude: 7.71152; **Identification:** identifiedBy: K.M. Olsen; **Event:** samplingProtocol: Sweep net; eventDate: 2017-06-23; verbatimEventDate: 23/Jun/2017; **Record Level:** institutionCode: PCKMO; basisOfRecord: PreservedSpecimen
- **Type status:** Other material. **Occurrence:** occurrenceRemarks: 1 female; recordedBy: D.I. Gavryushin; individualCount: 1; sex: female; occurrenceID: EU_LIM_152; **Taxon:** scientificName: Dicranomyia (Dicranomyia) quadra (Meigen, 1838); family: Limoniidae; genus: Dicranomyia; subgenus: Dicranomyia; specificEpithet: quadra; scientificNameAuthorship: (Meigen, 1838); **Location:** country: Russia; stateProvince: East European Russia; county: Bashkortostan Respublika; municipality: Beloretsk district; locality: Makhmutovo env., Belaya River; verbatimElevation: 550 m; minimumElevationInMeters: 550; decimalLatitude: 54.33012; decimalLongitude: 58.80735; **Identification:** identifiedBy: D.I. Gavryushin; **Event:** samplingProtocol: Sweep net; eventDate: 2015-06-15; verbatimEventDate: 15/Jul/2015; **Record Level:** institutionCode: ZMMU; basisOfRecord: PreservedSpecimen
- **Type status:** Other material. **Occurrence:** occurrenceRemarks: 1 female; recordedBy: D.I. Gavryushin; individualCount: 1; sex: female; preparations: Ethanol; occurrenceID: EU_LIM_153; **Taxon:** scientificName: Dicranomyia (Dicranomyia) quadra (Meigen, 1838); family: Limoniidae; genus: Dicranomyia; subgenus: Dicranomyia; specificEpithet: quadra; scientificNameAuthorship: (Meigen, 1838); **Location:** country: Serbia; stateProvince: Zaječar; municipality: Knjaževac; locality: Crni Vrh; verbatimElevation: 800 m; minimumElevationInMeters: 800; decimalLatitude: 43.407; decimalLongitude: 22.587; **Identification:** identifiedBy: D.I. Gavryushin; **Event:** samplingProtocol: Sweep net; eventDate: 2015-06-01/2015-07-07; verbatimEventDate: 01-07/Jul/2015; **Record Level:** institutionCode: ZMMU; basisOfRecord: PreservedSpecimen
- **Type status:** Other material. **Occurrence:** occurrenceRemarks: 1 male; recordedBy: D.I. Gavryushin; individualCount: 1; sex: male; preparations: Pinned; occurrenceID: EU_LIM_154; **Taxon:** scientificName: Dicranomyia (Dicranomyia) quadra (Meigen, 1838); family: Limoniidae; genus: Dicranomyia; subgenus: Dicranomyia; specificEpithet: quadra; scientificNameAuthorship: (Meigen, 1838); **Location:** country: Serbia; stateProvince: Zaječar; municipality: Knjaževac; locality: Crni Vrh; verbatimElevation: 800 m; minimumElevationInMeters: 800; decimalLatitude: 43.407; decimalLongitude: 22.587; **Identification:** identifiedBy: D.I. Gavryushin; **Event:** samplingProtocol: Sweep net; eventDate: 2015-06-01/2015-07-07; verbatimEventDate: 01-07/Jul/2015; **Record Level:** institutionCode: ZMMU; basisOfRecord: PreservedSpecimen
- **Type status:** Other material. **Occurrence:** occurrenceRemarks: 1 female; recordedBy: D.I. Gavryushin; individualCount: 1; sex: female; preparations: Ethanol; occurrenceID: EU_LIM_155; **Taxon:** scientificName: Dicranomyia (Dicranomyia) quadra (Meigen, 1838); family: Limoniidae; genus: Dicranomyia; subgenus: Dicranomyia; specificEpithet: quadra; scientificNameAuthorship: (Meigen, 1838); **Location:** country: Serbia; locality: Stara Planina Mts.; verbatimElevation: 1030 m; minimumElevationInMeters: 1030; decimalLatitude: 43.396; decimalLongitude: 22.607; **Identification:** identifiedBy: D.I. Gavryushin; **Event:** samplingProtocol: Sweep net; eventDate: 2015-05-01/2015-05-08; verbatimEventDate: 01-08/May/2015; **Record Level:** institutionCode: ZMMU; basisOfRecord: PreservedSpecimen
- **Type status:** Other material. **Occurrence:** occurrenceRemarks: 1 male; recordedBy: M. Andersson; individualCount: 1; sex: male; preparations: Pinned; occurrenceID: EU_LIM_156; **Taxon:** scientificName: Dicranomyia (Dicranomyia) quadra (Meigen, 1838); family: Limoniidae; genus: Dicranomyia; subgenus: Dicranomyia; specificEpithet: quadra; scientificNameAuthorship: (Meigen, 1838); **Location:** country: Sweden; stateProvince: Småland; municipality: Jönköping; locality: Norrängen, Huskvarna; verbatimElevation: 25 m; minimumElevationInMeters: 25; decimalLatitude: 57.81127; decimalLongitude: 14.27511; **Identification:** identifiedBy: M. Andersson; **Event:** samplingProtocol: Observation; eventDate: 2019-05-21; verbatimEventDate: 21/May/2019; **Record Level:** institutionCode: NHRS; basisOfRecord: PreservedSpecimen
- **Type status:** Other material. **Occurrence:** occurrenceRemarks: 1 male; recordedBy: M. Andersson; individualCount: 1; sex: male; preparations: Pinned; occurrenceID: EU_LIM_157; **Taxon:** scientificName: Dicranomyia (Dicranomyia) quadra (Meigen, 1838); family: Limoniidae; genus: Dicranomyia; subgenus: Dicranomyia; specificEpithet: quadra; scientificNameAuthorship: (Meigen, 1838); **Location:** country: Sweden; stateProvince: Småland; municipality: Jönköping; locality: Klevenbrantens Nature Reserve, Gränna; verbatimElevation: 169 m; minimumElevationInMeters: 169; decimalLatitude: 58.10962; decimalLongitude: 14.54469; **Identification:** identifiedBy: M. Andersson; **Event:** samplingProtocol: Sweep net; eventDate: 2020-06-18; verbatimEventDate: 18/Jun/2020; **Record Level:** institutionCode: NHRS; basisOfRecord: PreservedSpecimen
- **Type status:** Other material. **Occurrence:** occurrenceRemarks: 1 male; recordedBy: M. Andersson | R. Isaksson; individualCount: 1; sex: male; preparations: Pinned; occurrenceID: EU_LIM_158; **Taxon:** scientificName: Dicranomyia (Dicranomyia) quadra (Meigen, 1838); family: Limoniidae; genus: Dicranomyia; subgenus: Dicranomyia; specificEpithet: quadra; scientificNameAuthorship: (Meigen, 1838); **Location:** country: Sweden; stateProvince: Västergötland; municipality: Falköping; locality: Tomten, Torbjörntorp; verbatimElevation: 100 m; minimumElevationInMeters: 100; decimalLatitude: 58.22315; decimalLongitude: 13.61435; **Identification:** identifiedBy: M. Andersson; **Event:** samplingProtocol: Sweep net; eventDate: 2020-06-07; verbatimEventDate: 7/Jun/2020; **Record Level:** institutionCode: NHRS; basisOfRecord: PreservedSpecimen

#### Distribution

First records from North Macedonia, Norway, Russia: RUE, Serbia and Sweden.

### Dicranomyia (Dicranomyia) radegasti

Starý, 1993

F88162A4-FE78-56A7-A8EA-B6F677A1601C

https://ccw.naturalis.nl/detail.php?id=8614

#### Materials

- **Type status:** Other material. **Occurrence:** catalogNumber: 611590; occurrenceRemarks: 2 males; recordedBy: S. Svendsen; individualCount: 2; sex: male; preparations: Ethanol; occurrenceID: EU_LIM_159; **Taxon:** scientificName: Dicranomyia (Dicranomyia) radegasti Starý, 1993; family: Limoniidae; genus: Dicranomyia; subgenus: Dicranomyia; specificEpithet: radegasti; scientificNameAuthorship: Starý, 1993; **Location:** country: Norway; stateProvince: Aust-Agder; municipality: Birkenes; locality: Nordåsen; verbatimElevation: 85 m; minimumElevationInMeters: 85; decimalLatitude: 58.33342; decimalLongitude: 8.24004; **Identification:** identifiedBy: K.M. Olsen; **Event:** samplingProtocol: Light trap; eventDate: 2016-06/2016-08; verbatimEventDate: Jul-Aug/2016; **Record Level:** institutionCode: NHMO; basisOfRecord: PreservedSpecimen
- **Type status:** Other material. **Occurrence:** catalogNumber: 612205; occurrenceRemarks: 4 males; recordedBy: S. Svendsen | K. Berggren; individualCount: 4; sex: male; preparations: Ethanol; occurrenceID: EU_LIM_160; **Taxon:** scientificName: Dicranomyia (Dicranomyia) radegasti Starý, 1993; family: Limoniidae; genus: Dicranomyia; subgenus: Dicranomyia; specificEpithet: radegasti; scientificNameAuthorship: Starý, 1993; **Location:** country: Norway; stateProvince: Aust-Agder; municipality: Birkenes; locality: Nordåsen; verbatimElevation: 85 m; minimumElevationInMeters: 85; decimalLatitude: 58.33342; decimalLongitude: 8.24004; **Identification:** identifiedBy: K.M. Olsen; **Event:** samplingProtocol: Light trap; eventDate: 2017-05/2017-07; verbatimEventDate: May-Jul/2017; **Record Level:** institutionCode: NHMO; basisOfRecord: PreservedSpecimen
- **Type status:** Other material. **Occurrence:** catalogNumber: 630637; occurrenceRemarks: 1 male; recordedBy: S. Svendsen | K. Berggren; individualCount: 1; sex: male; preparations: Ethanol; occurrenceID: EU_LIM_161; **Taxon:** scientificName: Dicranomyia (Dicranomyia) radegasti Starý, 1993; family: Limoniidae; genus: Dicranomyia; subgenus: Dicranomyia; specificEpithet: radegasti; scientificNameAuthorship: Starý, 1993; **Location:** country: Norway; stateProvince: Aust-Agder; municipality: Birkenes; locality: Nordåsen; verbatimElevation: 85 m; minimumElevationInMeters: 85; decimalLatitude: 58.33342; decimalLongitude: 8.24004; **Identification:** identifiedBy: K.M. Olsen; **Event:** samplingProtocol: Light trap; eventDate: 2018-06/2018-08; verbatimEventDate: Jul-Aug/2018; **Record Level:** institutionCode: NHMO; basisOfRecord: PreservedSpecimen
- **Type status:** Other material. **Occurrence:** catalogNumber: 647991, 647992, 647993; occurrenceRemarks: 4 male+female; recordedBy: Rikmyrsprosjektet (L.K. Hagenlund); individualCount: 4; sex: male, female; preparations: Ethanol; occurrenceID: EU_LIM_162; **Taxon:** scientificName: Dicranomyia (Dicranomyia) radegasti Starý, 1993; family: Limoniidae; genus: Dicranomyia; subgenus: Dicranomyia; specificEpithet: radegasti; scientificNameAuthorship: Starý, 1993; **Location:** country: Norway; stateProvince: Hedmark; municipality: Stor-Elvdal; locality: Ottestad; verbatimElevation: 240 m; minimumElevationInMeters: 240; decimalLatitude: 61.29711; decimalLongitude: 11.27715; **Identification:** identifiedBy: K.M. Olsen; **Event:** samplingProtocol: Light trap; eventDate: 2017-06-21/2017-07-20; verbatimEventDate: 21/Jun-20/Jul/2017; **Record Level:** institutionCode: PCKMO; basisOfRecord: PreservedSpecimen
- **Type status:** Other material. **Occurrence:** catalogNumber: 606848; occurrenceRemarks: 1 male; recordedBy: F. Midtgaard; individualCount: 1; sex: male; preparations: Ethanol; occurrenceID: EU_LIM_163; **Taxon:** scientificName: Dicranomyia (Dicranomyia) radegasti Starý, 1993; family: Limoniidae; genus: Dicranomyia; subgenus: Dicranomyia; specificEpithet: radegasti; scientificNameAuthorship: Starý, 1993; **Location:** country: Norway; stateProvince: Sogn og Fjordane; municipality: Naustdal; locality: Naustdal; verbatimElevation: 10 m; minimumElevationInMeters: 10; decimalLatitude: 61.51197; decimalLongitude: 5.72036; **Identification:** identifiedBy: K.M. Olsen; **Event:** samplingProtocol: Malaise trap; eventDate: 1986-05-28/1986-07-03; verbatimEventDate: 28/May-03/Jul/1986; **Record Level:** institutionCode: NHMO; basisOfRecord: PreservedSpecimen
- **Type status:** Other material. **Occurrence:** catalogNumber: 548952; occurrenceRemarks: 1 male; recordedBy: K. Berggren | R.-A. Golf; individualCount: 1; sex: male; preparations: Ethanol; occurrenceID: EU_LIM_164; **Taxon:** scientificName: Dicranomyia (Dicranomyia) radegasti Starý, 1993; family: Limoniidae; genus: Dicranomyia; subgenus: Dicranomyia; specificEpithet: radegasti; scientificNameAuthorship: Starý, 1993; **Location:** country: Norway; stateProvince: Sogn og Fjordane; municipality: Luster; locality: SE Buhaug – Ved Breheimsenteret; verbatimElevation: 260 m; minimumElevationInMeters: 260; decimalLatitude: 61.65147; decimalLongitude: 7.27477; **Identification:** identifiedBy: K.M. Olsen; **Event:** samplingProtocol: Light trap; eventDate: 2017-06/2017-07; verbatimEventDate: Jun-Jul/2017; **Record Level:** institutionCode: PCKMO; basisOfRecord: PreservedSpecimen
- **Type status:** Other material. **Occurrence:** catalogNumber: 600548; occurrenceRemarks: 3 males; recordedBy: K. Berggren; individualCount: 3; sex: male; preparations: Ethanol; occurrenceID: EU_LIM_165; **Taxon:** scientificName: Dicranomyia (Dicranomyia) radegasti Starý, 1993; family: Limoniidae; genus: Dicranomyia; subgenus: Dicranomyia; specificEpithet: radegasti; scientificNameAuthorship: Starý, 1993; **Location:** country: Norway; stateProvince: Vest-Agder; municipality: Flekkefjord; locality: Helle; verbatimElevation: 25 m; minimumElevationInMeters: 25; decimalLatitude: 58.23954; decimalLongitude: 6.69968; **Identification:** identifiedBy: K.M. Olsen; **Event:** samplingProtocol: Light trap; eventDate: 2017-06; verbatimEventDate: Jul/2017; **Record Level:** institutionCode: NHMO; basisOfRecord: PreservedSpecimen
- **Type status:** Other material. **Occurrence:** catalogNumber: 528590; occurrenceRemarks: 1 male; recordedBy: K. Berggren; individualCount: 1; sex: male; preparations: Ethanol; occurrenceID: EU_LIM_166; **Taxon:** scientificName: Dicranomyia (Dicranomyia) radegasti Starý, 1993; family: Limoniidae; genus: Dicranomyia; subgenus: Dicranomyia; specificEpithet: radegasti; scientificNameAuthorship: Starý, 1993; **Location:** country: Norway; stateProvince: Vest-Agder; municipality: Kristiansand; locality: Bråvann terrasse – I hagen til nr. 21; verbatimElevation: 75 m; minimumElevationInMeters: 75; decimalLatitude: 58.11029; decimalLongitude: 7.93429; **Identification:** identifiedBy: K.M. Olsen; **Event:** samplingProtocol: Light trap; eventDate: 2017-06/2017-07; verbatimEventDate: Jun-Jul/2017; **Record Level:** institutionCode: PCKMO; basisOfRecord: PreservedSpecimen
- **Type status:** Other material. **Occurrence:** catalogNumber: 600603; occurrenceRemarks: 1 male; recordedBy: K. Berggren; individualCount: 1; sex: male; preparations: Ethanol; occurrenceID: EU_LIM_167; **Taxon:** scientificName: Dicranomyia (Dicranomyia) radegasti Starý, 1993; family: Limoniidae; genus: Dicranomyia; subgenus: Dicranomyia; specificEpithet: radegasti; scientificNameAuthorship: Starý, 1993; **Location:** country: Norway; stateProvince: Vest-Agder; municipality: Kristiansand; locality: Bråvann terrasse – I hagen til nr. 21; verbatimElevation: 75 m; minimumElevationInMeters: 75; decimalLatitude: 58.11029; decimalLongitude: 7.93429; **Identification:** identifiedBy: K.M. Olsen; **Event:** samplingProtocol: Light trap; eventDate: 2017-08; verbatimEventDate: Aug/2017; **Record Level:** institutionCode: NHMO; basisOfRecord: PreservedSpecimen
- **Type status:** Other material. **Occurrence:** catalogNumber: 594986; occurrenceRemarks: 4 males; recordedBy: K. Berggren; individualCount: 4; sex: male; preparations: Ethanol; occurrenceID: EU_LIM_168; **Taxon:** scientificName: Dicranomyia (Dicranomyia) radegasti Starý, 1993; family: Limoniidae; genus: Dicranomyia; subgenus: Dicranomyia; specificEpithet: radegasti; scientificNameAuthorship: Starý, 1993; **Location:** country: Norway; stateProvince: Vest-Agder; municipality: Kristiansand; locality: Nedre Timenes; verbatimElevation: 10 m; minimumElevationInMeters: 10; decimalLatitude: 58.16164; decimalLongitude: 8.09907; **Identification:** identifiedBy: K.M. Olsen; **Event:** samplingProtocol: Light trap; eventDate: 2017-10-28; verbatimEventDate: 28/Oct/2017; **Record Level:** institutionCode: PCKMO; basisOfRecord: PreservedSpecimen
- **Type status:** Other material. **Occurrence:** catalogNumber: 599024; occurrenceRemarks: 1 male; recordedBy: K. Berggren; individualCount: 1; sex: male; preparations: Ethanol; occurrenceID: EU_LIM_169; **Taxon:** scientificName: Dicranomyia (Dicranomyia) radegasti Starý, 1993; family: Limoniidae; genus: Dicranomyia; subgenus: Dicranomyia; specificEpithet: radegasti; scientificNameAuthorship: Starý, 1993; **Location:** country: Norway; stateProvince: Vest-Agder; municipality: Kristiansand; locality: Nedre Timenes; verbatimElevation: 10 m; minimumElevationInMeters: 10; decimalLatitude: 58.16155; decimalLongitude: 8.10013; **Identification:** identifiedBy: K.M. Olsen; **Event:** samplingProtocol: Light trap; eventDate: 2017-06; verbatimEventDate: Jul/2017; **Record Level:** institutionCode: NHMO; basisOfRecord: PreservedSpecimen
- **Type status:** Other material. **Occurrence:** catalogNumber: 612379; occurrenceRemarks: 1 male; recordedBy: K. Berggren; individualCount: 1; sex: male; preparations: Ethanol; occurrenceID: EU_LIM_170; **Taxon:** scientificName: Dicranomyia (Dicranomyia) radegasti Starý, 1993; family: Limoniidae; genus: Dicranomyia; subgenus: Dicranomyia; specificEpithet: radegasti; scientificNameAuthorship: Starý, 1993; **Location:** country: Norway; stateProvince: Vest-Agder; municipality: Kristiansand; locality: Nedre Timenes; verbatimElevation: 10 m; minimumElevationInMeters: 10; decimalLatitude: 58.16155; decimalLongitude: 8.10013; **Identification:** identifiedBy: K.M. Olsen; **Event:** samplingProtocol: Light trap; eventDate: 2018-06; verbatimEventDate: Jun/2018; **Record Level:** institutionCode: NHMO; basisOfRecord: PreservedSpecimen
- **Type status:** Other material. **Occurrence:** catalogNumber: 614224; occurrenceRemarks: 3 males; recordedBy: K. Berggren; individualCount: 3; sex: male; preparations: Ethanol; occurrenceID: EU_LIM_171; **Taxon:** scientificName: Dicranomyia (Dicranomyia) radegasti Starý, 1993; family: Limoniidae; genus: Dicranomyia; subgenus: Dicranomyia; specificEpithet: radegasti; scientificNameAuthorship: Starý, 1993; **Location:** country: Norway; stateProvince: Vest-Agder; municipality: Kristiansand; locality: Nedre Timenes; verbatimElevation: 10 m; minimumElevationInMeters: 10; decimalLatitude: 58.16155; decimalLongitude: 8.10013; **Identification:** identifiedBy: K.M. Olsen; **Event:** samplingProtocol: Light trap; eventDate: 2018-06; verbatimEventDate: Jul/2018; **Record Level:** institutionCode: NHMO; basisOfRecord: PreservedSpecimen
- **Type status:** Other material. **Occurrence:** catalogNumber: 629216; occurrenceRemarks: 2 males; recordedBy: K. Berggren; individualCount: 2; sex: male; preparations: Ethanol; occurrenceID: EU_LIM_172; **Taxon:** scientificName: Dicranomyia (Dicranomyia) radegasti Starý, 1993; family: Limoniidae; genus: Dicranomyia; subgenus: Dicranomyia; specificEpithet: radegasti; scientificNameAuthorship: Starý, 1993; **Location:** country: Norway; stateProvince: Vest-Agder; municipality: Kristiansand; locality: Nedre Timenes; verbatimElevation: 10 m; minimumElevationInMeters: 10; decimalLatitude: 58.16155; decimalLongitude: 8.10013; **Identification:** identifiedBy: K.M. Olsen; **Event:** samplingProtocol: Light trap; eventDate: 2018-06-01/2018-07-11; verbatimEventDate: 01-11/Jul/2018; **Record Level:** institutionCode: NHMO; basisOfRecord: PreservedSpecimen
- **Type status:** Other material. **Occurrence:** catalogNumber: 604526; occurrenceRemarks: 1 male; recordedBy: K. Berggren; individualCount: 1; sex: male; preparations: Ethanol; occurrenceID: EU_LIM_173; **Taxon:** scientificName: Dicranomyia (Dicranomyia) radegasti Starý, 1993; family: Limoniidae; genus: Dicranomyia; subgenus: Dicranomyia; specificEpithet: radegasti; scientificNameAuthorship: Starý, 1993; **Location:** country: Norway; stateProvince: Vest-Agder; municipality: Kristiansand; locality: Nedre Timenes; verbatimElevation: 10 m; minimumElevationInMeters: 10; decimalLatitude: 58.16155; decimalLongitude: 8.10013; **Identification:** identifiedBy: K.M. Olsen; **Event:** samplingProtocol: Light trap; eventDate: 2018-08; verbatimEventDate: Aug/2018; **Record Level:** institutionCode: PCKMO; basisOfRecord: PreservedSpecimen
- **Type status:** Other material. **Occurrence:** catalogNumber: 614292; occurrenceRemarks: 1 male; recordedBy: K. Berggren; individualCount: 1; sex: male; occurrenceID: EU_LIM_174; **Taxon:** scientificName: Dicranomyia (Dicranomyia) radegasti Starý, 1993; family: Limoniidae; genus: Dicranomyia; subgenus: Dicranomyia; specificEpithet: radegasti; scientificNameAuthorship: Starý, 1993; **Location:** country: Norway; stateProvince: Vest-Agder; municipality: Kristiansand; locality: Nedre Timenes; verbatimElevation: 10 m; minimumElevationInMeters: 10; decimalLatitude: 58.16155; decimalLongitude: 8.10013; **Identification:** identifiedBy: K.M. Olsen; **Event:** samplingProtocol: Light trap; eventDate: 2018-08/2018-09; verbatimEventDate: Aug-Sep/2018; **Record Level:** institutionCode: BioFokus; basisOfRecord: HumanObservation
- **Type status:** Other material. **Occurrence:** catalogNumber: 582417; occurrenceRemarks: 1 male; recordedBy: K.M. Olsen; individualCount: 1; sex: male; preparations: Ethanol; occurrenceID: EU_LIM_175; **Taxon:** scientificName: Dicranomyia (Dicranomyia) radegasti Starý, 1993; family: Limoniidae; genus: Dicranomyia; subgenus: Dicranomyia; specificEpithet: radegasti; scientificNameAuthorship: Starý, 1993; **Location:** country: United Kingdom; stateProvince: Scotland; county: Argyll and Bute; municipality: Taynuilt; locality: Glen Nant National Nature Reserve; verbatimElevation: 85 m; minimumElevationInMeters: 85; decimalLatitude: 56.39899; decimalLongitude: -5.2136; **Identification:** identifiedBy: K.M. Olsen; **Event:** samplingProtocol: Sweep net; eventDate: 2018-05-30; verbatimEventDate: 30/May/2018; **Record Level:** institutionCode: NHMO; basisOfRecord: PreservedSpecimen

#### Distribution

Presence of the species in Norway mentioned in Viitanen (2018) without further details; here, we publish the collection data of that record. Uncertain presence of the species in the United Kingdom (Scotland) mentioned in Kramer 2018; here, we confirm the identification of that specimen and the presence of the species in the United Kingdom.

### Dicranomyia (Melanolimonia) rufiventris

(Strobl, 1900)

0C1CB87A-A982-5D8A-A4F3-E6ABD284DA1B

https://ccw.naturalis.nl/detail.php?id=9012

#### Materials

- **Type status:** Other material. **Occurrence:** catalogNumber: 530925, 644168; occurrenceRemarks: 2 male+female; recordedBy: K.M. Olsen; individualCount: 3; sex: male, female; preparations: Ethanol; occurrenceID: EU_LIM_220; **Taxon:** scientificName: Dicranomyia (Idiopyga) nigristigma Nielsen, 1919; family: Limoniidae; genus: Dicranomyia; subgenus: Idiopyga; specificEpithet: nigristigma; scientificNameAuthorship: Nielsen, 1919; **Location:** country: Norway; stateProvince: Akershus; municipality: Skedsmo; locality: SSE Buhaugen; verbatimElevation: 110 m; minimumElevationInMeters: 110; decimalLatitude: 59.97227; decimalLongitude: 10.98641; **Identification:** identifiedBy: K.M. Olsen; **Event:** samplingProtocol: Sweep net; eventDate: 2017-09-07; verbatimEventDate: 07/Sep/2017; **Record Level:** institutionCode: PCKMO; basisOfRecord: PreservedSpecimen

#### Distribution

First record from Russia: RUE.

### Dicranomyia (Dicranomyia) sera

(Walker, 1848)

F25AAB72-A927-57ED-8A26-A1539840A0E4

https://ccw.naturalis.nl/detail.php?id=8655

#### Materials

- **Type status:** Other material. **Occurrence:** occurrenceRemarks: 6 males; recordedBy: E. Eiroa; individualCount: 6; sex: male; preparations: Pinned; occurrenceID: EU_LIM_176; **Taxon:** scientificName: Dicranomyia (Dicranomyia) sera (Walker, 1848); family: Limoniidae; genus: Dicranomyia; subgenus: Dicranomyia; specificEpithet: sera; scientificNameAuthorship: (Walker, 1848); **Location:** country: Spain; stateProvince: Galicia, La Coruńa; municipality: Riveira; locality: Corrubedo, laguna Carregal; verbatimElevation: 0 m; decimalLatitude: 42.57518; decimalLongitude: -9.03748; **Identification:** identifiedBy: E. Eiroa; **Event:** samplingProtocol: Sweep net; eventDate: 1995-04-10; verbatimEventDate: 10/April/1995; habitat: saltmarsh; **Record Level:** institutionCode: USC; basisOfRecord: PreservedSpecimen

#### Distribution

First record from the mainland Spain, previously reported from Canary Is.

### Dicranomyia (Glochina) sericata

(Meigen, 1830)

8C30DF19-F0CB-5C80-8625-0035F5223AC0

https://ccw.naturalis.nl/detail.php?id=8890

#### Materials

- **Type status:** Other material. **Occurrence:** occurrenceRemarks: 2 males; recordedBy: D. Nyström; individualCount: 2; sex: male; preparations: Pinned; occurrenceID: EU_LIM_181; **Taxon:** scientificName: Dicranomyia (Glochina) sericata (Meigen, 1830); family: Limoniidae; genus: Dicranomyia; subgenus: Glochina; specificEpithet: sericata; scientificNameAuthorship: (Meigen, 1830); **Location:** country: Sweden; stateProvince: Gotland; municipality: Gotland; locality: Själsöån Nature Reserve, Väskinde; verbatimElevation: 25 m; minimumElevationInMeters: 25; decimalLatitude: 57.69364; decimalLongitude: 18.35533; **Identification:** identifiedBy: M. Andersson; **Event:** samplingProtocol: Sweep net; eventDate: 2020-05-16; verbatimEventDate: 16/May/2020; **Record Level:** institutionCode: NHRS; basisOfRecord: PreservedSpecimen
- **Type status:** Other material. **Occurrence:** occurrenceRemarks: 1 male; recordedBy: D. Nyström; individualCount: 1; sex: male; preparations: Pinned; occurrenceID: EU_LIM_182; **Taxon:** scientificName: Dicranomyia (Glochina) sericata (Meigen, 1830); family: Limoniidae; genus: Dicranomyia; subgenus: Glochina; specificEpithet: sericata; scientificNameAuthorship: (Meigen, 1830); **Location:** country: Sweden; stateProvince: Gotland; municipality: Gotland; locality: Jungfrun Nature Reserve, Stenkyrka; verbatimElevation: 5 m; minimumElevationInMeters: 5; decimalLatitude: 57.83043; decimalLongitude: 18.50187; **Identification:** identifiedBy: M. Andersson; **Event:** samplingProtocol: Observation; eventDate: 2020-05-06; verbatimEventDate: 6/May/2020; **Record Level:** institutionCode: NHRS; basisOfRecord: PreservedSpecimen

#### Distribution

First records from Sweden.

### Dicranomyia (Dicranomyia) signatella

Starý and Freidberg, 2007

06769363-4566-5090-A9C8-16ED97177614

https://ccw.naturalis.nl/detail.php?id=17501

#### Materials

- **Type status:** Other material. **Occurrence:** occurrenceRemarks: 1 male; recordedBy: L.-P. Kolcsár; individualCount: 1; sex: male; preparations: Ethanol; occurrenceID: EU_LIM_177; **Taxon:** scientificName: Dicranomyia (Dicranomyia) signatella Starý and Freidberg, 2007; family: Limoniidae; genus: Dicranomyia; subgenus: Dicranomyia; specificEpithet: signatella; scientificNameAuthorship: Starý and Freidberg, 2007; **Location:** country: Montenegro; municipality: Bjelos; locality: Lovćen Mts.; verbatimElevation: 950 m; minimumElevationInMeters: 950; decimalLatitude: 42.36701; decimalLongitude: 18.89107; **Identification:** identifiedBy: J. Starý; **Event:** samplingProtocol: Sweep net; eventDate: 2014-05-03; verbatimEventDate: 3/May/2014; **Record Level:** institutionCode: PCJS; basisOfRecord: PreservedSpecimen

#### Distribution

First records from Montenegro.

### Dicranomyia (Idiopyga) stigmatica

(Meigen, 1830)

C6681337-DDA9-5288-BC5F-FE10FE955B04

https://ccw.naturalis.nl/detail.php?id=8966

#### Materials

- **Type status:** Other material. **Occurrence:** occurrenceRemarks: 1 male; recordedBy: D.I. Gavryushin; individualCount: 1; sex: male; occurrenceID: EU_LIM_221; **Taxon:** scientificName: Dicranomyia (Idiopyga) stigmatica (Meigen, 1830); family: Limoniidae; genus: Dicranomyia; subgenus: Idiopyga; specificEpithet: stigmatica; scientificNameAuthorship: (Meigen, 1830); **Location:** country: Russia; stateProvince: East European Russia; county: Bashkortostan Respublika; municipality: Beloretsk district; locality: Abzakovo env., Kulsugady River; verbatimElevation: 531 m; minimumElevationInMeters: 531; decimalLatitude: 53.83795; decimalLongitude: 58.5823; **Identification:** identifiedBy: D.I. Gavryushin; **Event:** samplingProtocol: Sweep net; eventDate: 2012-08-15; verbatimEventDate: Aug-15-2012; **Record Level:** institutionCode: ZMMU; basisOfRecord: PreservedSpecimen
- **Type status:** Other material. **Occurrence:** occurrenceRemarks: 1 male, 1 female; recordedBy: V.E. Pilipenko; individualCount: 2; sex: male, female; occurrenceID: EU_LIM_222; **Taxon:** scientificName: Dicranomyia (Idiopyga) stigmatica (Meigen, 1830); family: Limoniidae; genus: Dicranomyia; subgenus: Idiopyga; specificEpithet: stigmatica; scientificNameAuthorship: (Meigen, 1830); **Location:** country: Russia; stateProvince: Central European Russia; county: Moskovskaya Oblast; municipality: Solnechnogorsk district; locality: Chashnikovo; verbatimElevation: 220 m; minimumElevationInMeters: 220; decimalLatitude: 56.0375; decimalLongitude: 37.1874; **Identification:** identifiedBy: V.E. Pilipenko; **Event:** samplingProtocol: Sweep net; eventDate: 1995-10-03; verbatimEventDate: 03/Oct/1995; **Record Level:** institutionCode: VPMC; basisOfRecord: PreservedSpecimen

#### Distribution

First records from Russia: RUC, RUE.

### Dicranomyia (Glochina) tristis

(Schummel, 1829)

9D70606E-F24B-5069-B9D0-7112F43F428C

https://ccw.naturalis.nl/detail.php?id=8898

#### Materials

- **Type status:** Other material. **Occurrence:** occurrenceRemarks: 1 male; recordedBy: L.-P. Kolcsár; individualCount: 1; sex: male; preparations: Ethanol; occurrenceID: EU_LIM_183; **Taxon:** scientificName: Dicranomyia (Glochina) tristis (Schummel, 1829); family: Limoniidae; genus: Dicranomyia; subgenus: Glochina; specificEpithet: tristis; scientificNameAuthorship: (Schummel, 1829); **Location:** country: Latvia; municipality: Skaistkalne; verbatimElevation: 12 m; minimumElevationInMeters: 12; decimalLatitude: 56.411; decimalLongitude: 24.637; **Identification:** identifiedBy: L.-P. Kolcsár; **Event:** samplingProtocol: Sweep net; eventDate: 2018-06-19; verbatimEventDate: Jul-19-2018; habitat: birch-spruce forest, small stream; **Record Level:** institutionCode: CKLP; basisOfRecord: PreservedSpecimen
- **Type status:** Other material. **Occurrence:** occurrenceRemarks: 1 male; recordedBy: Sh.A. Murtazin; individualCount: 1; sex: male; occurrenceID: EU_LIM_184; **Taxon:** scientificName: Dicranomyia (Glochina) tristis (Schummel, 1829); family: Limoniidae; genus: Dicranomyia; subgenus: Glochina; specificEpithet: tristis; scientificNameAuthorship: (Schummel, 1829); **Location:** country: Russia; stateProvince: East European Russia; county: Bashkortostan Respublika; municipality: Ufa; verbatimElevation: 100 m; minimumElevationInMeters: 100; decimalLatitude: 54.73515; decimalLongitude: 55.95873; **Identification:** identifiedBy: N.M. Paramonov; **Event:** samplingProtocol: Sweep net; eventDate: 2017-06-11; verbatimEventDate: 11/Jun/2017; **Record Level:** institutionCode: ZIN; basisOfRecord: PreservedSpecimen
- **Type status:** Other material. **Occurrence:** occurrenceRemarks: 1 male; recordedBy: A. Polevoi; individualCount: 1; sex: male; preparations: Pinned; occurrenceID: EU_LIM_185; **Taxon:** scientificName: Dicranomyia (Glochina) tristis (Schummel, 1829); family: Limoniidae; genus: Dicranomyia; subgenus: Glochina; specificEpithet: tristis; scientificNameAuthorship: (Schummel, 1829); **Location:** country: Russia; stateProvince: North European Russia; county: Republic Karelia; municipality: Lahdenpohja district; locality: Sikopohja, 9 km NE; verbatimElevation: 80 m; minimumElevationInMeters: 80; decimalLatitude: 61.68749; decimalLongitude: 30.16007; **Identification:** identifiedBy: A. Polevoi; **Event:** samplingProtocol: Sweep net; eventDate: 2005-06-07; verbatimEventDate: 07/Jul/2005; **Record Level:** institutionCode: FRIP; basisOfRecord: PreservedSpecimen
- **Type status:** Other material. **Occurrence:** occurrenceRemarks: 1 male; recordedBy: N.M. Paramonov; individualCount: 1; sex: male; occurrenceID: EU_LIM_186; **Taxon:** scientificName: Dicranomyia (Glochina) tristis (Schummel, 1829); family: Limoniidae; genus: Dicranomyia; subgenus: Glochina; specificEpithet: tristis; scientificNameAuthorship: (Schummel, 1829); **Location:** country: Russia; stateProvince: East European Russia; county: Tatarstan Respublika; municipality: Kazan; locality: district Derbyshki, Noksa River; verbatimElevation: 60 m; minimumElevationInMeters: 60; decimalLatitude: 55.8655; decimalLongitude: 49.22461; **Identification:** identifiedBy: N.M. Paramonov; **Event:** samplingProtocol: Sweep net; eventDate: 2009-06-26; verbatimEventDate: 26/Jun/2009; **Record Level:** institutionCode: ZIN; basisOfRecord: PreservedSpecimen
- **Type status:** Other material. **Occurrence:** occurrenceRemarks: 1 male; recordedBy: N.M. Paramonov; individualCount: 1; sex: male; occurrenceID: EU_LIM_187; **Taxon:** scientificName: Dicranomyia (Glochina) tristis (Schummel, 1829); family: Limoniidae; genus: Dicranomyia; subgenus: Glochina; specificEpithet: tristis; scientificNameAuthorship: (Schummel, 1829); **Location:** country: Russia; stateProvince: East European Russia; county: Tatarstan Respublika; municipality: Zelenodol’sk district; locality: Ilinskoe; verbatimElevation: 90 m; minimumElevationInMeters: 90; decimalLatitude: 55.87455; decimalLongitude: 48.68579; **Identification:** identifiedBy: N.M. Paramonov; **Event:** samplingProtocol: Sweep net; eventDate: 2009-06-12; verbatimEventDate: 12/Jun/2009; habitat: village env.; **Record Level:** institutionCode: ZIN; basisOfRecord: PreservedSpecimen
- **Type status:** Other material. **Occurrence:** occurrenceRemarks: 2 males; recordedBy: N.M. Paramonov; individualCount: 2; sex: male; occurrenceID: EU_LIM_188; **Taxon:** scientificName: Dicranomyia (Glochina) tristis (Schummel, 1829); family: Limoniidae; genus: Dicranomyia; subgenus: Glochina; specificEpithet: tristis; scientificNameAuthorship: (Schummel, 1829); **Location:** country: Russia; stateProvince: East European Russia; county: Tatarstan Respublika; municipality: Zelenodol’sk district; locality: Volga-Kama State Nature Biosphere Reserve, «Raifa»; verbatimElevation: 100 m; minimumElevationInMeters: 100; decimalLatitude: 55.88868; decimalLongitude: 48.71434; **Identification:** identifiedBy: N.M. Paramonov; **Event:** samplingProtocol: Sweep net; eventDate: 2009-06-14/2009-06-15; verbatimEventDate: 14-15/Jun/2009; **Record Level:** institutionCode: ZIN; basisOfRecord: PreservedSpecimen

#### Distribution

First records from Latvia and Russia: RUE, RUN.

### Dicranomyia (Dicranomyia) ventralis

(Schummel, 1829)

C12CFAF8-9256-5DB1-913A-829E126F65B7

https://ccw.naturalis.nl/detail.php?id=8795

#### Materials

- **Type status:** Other material. **Occurrence:** occurrenceRemarks: 1 male; recordedBy: D.I. Gavryushin; individualCount: 1; sex: male; preparations: Pinned; occurrenceID: EU_LIM_178; **Taxon:** scientificName: Dicranomyia (Dicranomyia) ventralis (Schummel, 1829); family: Limoniidae; genus: Dicranomyia; subgenus: Dicranomyia; specificEpithet: ventralis; scientificNameAuthorship: (Schummel, 1829); **Location:** country: Belarus; stateProvince: Minsk; county: Barysaw; locality: Byarezina River; verbatimElevation: 151 m; minimumElevationInMeters: 151; decimalLatitude: 54.23935; decimalLongitude: 28.49438; **Identification:** identifiedBy: D.I. Gavryushin; **Event:** samplingProtocol: Sweep net; eventDate: 2013-06-05; verbatimEventDate: Jul-05-2013; **Record Level:** institutionCode: ZMMU; basisOfRecord: PreservedSpecimen
- **Type status:** Other material. **Occurrence:** occurrenceRemarks: 1 male; recordedBy: D.I. Gavryushin; individualCount: 1; sex: male; occurrenceID: EU_LIM_179; **Taxon:** scientificName: Dicranomyia (Dicranomyia) ventralis (Schummel, 1829); family: Limoniidae; genus: Dicranomyia; subgenus: Dicranomyia; specificEpithet: ventralis; scientificNameAuthorship: (Schummel, 1829); **Location:** country: Russia; stateProvince: East European Russia; county: Bashkortostan Respublika; municipality: Beloretsk district; locality: Bolshoy Inzer River (ca. 6km NW of Ulu-Elga village); verbatimElevation: 505 m; minimumElevationInMeters: 505; decimalLatitude: 53.97375; decimalLongitude: 57.87472; **Identification:** identifiedBy: D.I. Gavryushin; **Event:** samplingProtocol: Sweep net; eventDate: 2012-08-14; verbatimEventDate: 14/Aug/2012; **Record Level:** institutionCode: ZMMU; basisOfRecord: PreservedSpecimen

#### Distribution

First records from Belarus and Russia: RUE.

### Dicranophragma (Brachylimnophila) separatum

(Walker, 1848)

894295A6-3FFE-5723-8234-81A48172A82D

https://ccw.naturalis.nl/detail.php?id=6897

#### Materials

- **Type status:** Other material. **Occurrence:** occurrenceRemarks: 1 female; recordedBy: D.I. Gavryushin; individualCount: 1; sex: female; preparations: Pinned; occurrenceID: EU_LIM_252; **Taxon:** scientificName: Dicranophragma (Brachylimnophila) separatum (Walker, 1848); family: Limoniidae; genus: Dicranophragma; subgenus: Brachylimnophila; specificEpithet: separatum; scientificNameAuthorship: (Walker, 1848); **Location:** country: Belarus; stateProvince: Minsk; county: Barysaw; locality: Glivin; verbatimElevation: 161 m; minimumElevationInMeters: 161; decimalLatitude: 54.14902; decimalLongitude: 28.63648; **Identification:** identifiedBy: D.I. Gavryushin; **Event:** samplingProtocol: Sweep net; eventDate: 2013-06-06; verbatimEventDate: Jul-06-2013; **Record Level:** institutionCode: ZMMU; basisOfRecord: PreservedSpecimen
- **Type status:** Other material. **Occurrence:** occurrenceRemarks: 2 females; recordedBy: D.I. Gavryushin; individualCount: 2; sex: female; preparations: Pinned; occurrenceID: EU_LIM_253; **Taxon:** scientificName: Dicranophragma (Brachylimnophila) separatum (Walker, 1848); family: Limoniidae; genus: Dicranophragma; subgenus: Brachylimnophila; specificEpithet: separatum; scientificNameAuthorship: (Walker, 1848); **Location:** country: Belarus; stateProvince: Minsk; county: Barysaw; locality: Barysaw; verbatimElevation: 155 m; minimumElevationInMeters: 155; decimalLatitude: 54.25542; decimalLongitude: 28.48092; **Identification:** identifiedBy: D.I. Gavryushin; **Event:** samplingProtocol: Sweep net; eventDate: 2013-06-05; verbatimEventDate: 5/Jul/2013; **Record Level:** institutionCode: ZMMU; basisOfRecord: PreservedSpecimen
- **Type status:** Other material. **Occurrence:** occurrenceRemarks: 2 males, 3 females; recordedBy: D.I. Gavryushin; individualCount: 5; sex: male, female; preparations: Pinned; occurrenceID: EU_LIM_254; **Taxon:** scientificName: Dicranophragma (Brachylimnophila) separatum (Walker, 1848); family: Limoniidae; genus: Dicranophragma; subgenus: Brachylimnophila; specificEpithet: separatum; scientificNameAuthorship: (Walker, 1848); **Location:** country: Belarus; stateProvince: Minsk; county: Barysaw; locality: Vialikaje Stachava; verbatimElevation: 156 m; minimumElevationInMeters: 156; decimalLatitude: 54.26555; decimalLongitude: 28.38332; **Identification:** identifiedBy: D.I. Gavryushin; **Event:** samplingProtocol: Sweep net; eventDate: 2013-06-07; verbatimEventDate: 7/Jul/2013; **Record Level:** institutionCode: ZMMU; basisOfRecord: PreservedSpecimen
- **Type status:** Other material. **Occurrence:** occurrenceRemarks: 1 male; recordedBy: D.I. Gavryushin; individualCount: 1; sex: male; preparations: Pinned; occurrenceID: EU_LIM_255; **Taxon:** scientificName: Dicranophragma (Brachylimnophila) separatum (Walker, 1848); family: Limoniidae; genus: Dicranophragma; subgenus: Brachylimnophila; specificEpithet: separatum; scientificNameAuthorship: (Walker, 1848); **Location:** country: Belarus; stateProvince: Gomel; county: Mazyr; locality: Mazyr; decimalLatitude: 52.05; decimalLongitude: 29.31; **Identification:** identifiedBy: D.I. Gavryushin; **Event:** samplingProtocol: Sweep net; eventDate: 2019-06-11/2019-06-14; verbatimEventDate: 11-14/Jun/2019; **Record Level:** institutionCode: ZMMU; basisOfRecord: PreservedSpecimen
- **Type status:** Other material. **Occurrence:** occurrenceRemarks: 1 male; recordedBy: L. Papp; individualCount: 1; sex: male; preparations: Pinned; occurrenceID: EU_LIM_256; **Taxon:** scientificName: Dicranophragma (Brachylimnophila) separatum (Walker, 1848); family: Limoniidae; genus: Dicranophragma; subgenus: Brachylimnophila; specificEpithet: separatum; scientificNameAuthorship: (Walker, 1848); **Location:** country: Hungary; stateProvince: Baranya; municipality: Orfű; locality: Mecsek Mts.; decimalLatitude: 46.1276; decimalLongitude: 18.1537; **Identification:** identifiedBy: L.-P. Kolcsár; **Event:** eventDate: 1978-06-06; verbatimEventDate: 06/Jul/1978; habitat: hornbeam-oak forest; **Record Level:** institutionCode: HNHM; basisOfRecord: PreservedSpecimen
- **Type status:** Other material. **Occurrence:** occurrenceRemarks: 3 males; recordedBy: F. Mihányi; individualCount: 3; sex: male; preparations: Pinned; occurrenceID: EU_LIM_257; **Taxon:** scientificName: Dicranophragma (Brachylimnophila) separatum (Walker, 1848); family: Limoniidae; genus: Dicranophragma; subgenus: Brachylimnophila; specificEpithet: separatum; scientificNameAuthorship: (Walker, 1848); **Location:** country: Hungary; stateProvince: Heves; municipality: Mátraszentimre; decimalLatitude: 47.91221; decimalLongitude: 19.87901; **Identification:** identifiedBy: L.-P. Kolcsár; **Event:** eventDate: 1982-06-03/1982-06-14; verbatimEventDate: 3-14/Jun/1982; **Record Level:** institutionCode: HNHM; basisOfRecord: PreservedSpecimen
- **Type status:** Other material. **Occurrence:** occurrenceRemarks: 2 males; recordedBy: L.-P. Kolcsár; individualCount: 2; sex: male; preparations: Ethanol; occurrenceID: EU_LIM_258; **Taxon:** scientificName: Dicranophragma (Brachylimnophila) separatum (Walker, 1848); family: Limoniidae; genus: Dicranophragma; subgenus: Brachylimnophila; specificEpithet: separatum; scientificNameAuthorship: (Walker, 1848); **Location:** country: Latvia; municipality: Skaistkalne; verbatimElevation: 12 m; minimumElevationInMeters: 12; decimalLatitude: 56.411; decimalLongitude: 24.637; **Identification:** identifiedBy: L.-P. Kolcsár; **Event:** samplingProtocol: Sweep net; eventDate: 2018-06-19; verbatimEventDate: 19/Jul/2018; habitat: birch-spruce forest, small stream; **Record Level:** institutionCode: CKLP; basisOfRecord: PreservedSpecimen
- **Type status:** Other material. **Occurrence:** occurrenceRemarks: 1 male; recordedBy: L.-P. Kolcsár; individualCount: 1; sex: male; preparations: Ethanol; occurrenceID: EU_LIM_259; **Taxon:** scientificName: Dicranophragma (Brachylimnophila) separatum (Walker, 1848); family: Limoniidae; genus: Dicranophragma; subgenus: Brachylimnophila; specificEpithet: separatum; scientificNameAuthorship: (Walker, 1848); **Location:** country: Latvia; municipality: Ainaži; locality: Randu pļavas; verbatimElevation: 3 m; minimumElevationInMeters: 3; decimalLatitude: 57.8335; decimalLongitude: 24.3441; **Identification:** identifiedBy: L.-P. Kolcsár; **Event:** samplingProtocol: Sweep net; eventDate: 2018-06-25; verbatimEventDate: 25/Jul/2018; **Record Level:** institutionCode: CKLP; basisOfRecord: PreservedSpecimen
- **Type status:** Other material. **Occurrence:** occurrenceRemarks: 4 males; recordedBy: E.G. Hancock; individualCount: 4; sex: male; preparations: Pinned; occurrenceID: EU_LIM_260; **Taxon:** scientificName: Dicranophragma (Brachylimnophila) separatum (Walker, 1848); family: Limoniidae; genus: Dicranophragma; subgenus: Brachylimnophila; specificEpithet: separatum; scientificNameAuthorship: (Walker, 1848); **Location:** country: Poland; stateProvince: Lower Silesia; locality: rez. Uroczysko Wrzosy; verbatimElevation: 105m; minimumElevationInMeters: 105; decimalLatitude: 51.368; decimalLongitude: 16.549; **Identification:** identifiedBy: E. G. Hancock; **Event:** samplingProtocol: Sweep net; eventDate: 2016-05-23; verbatimEventDate: 23/May/2016; **Record Level:** institutionCode: HMUG; basisOfRecord: PreservedSpecimen
- **Type status:** Other material. **Occurrence:** occurrenceRemarks: 1 male; recordedBy: E.G. Hancock; individualCount: 1; sex: male; preparations: Pinned; occurrenceID: EU_LIM_261; **Taxon:** scientificName: Dicranophragma (Brachylimnophila) separatum (Walker, 1848); family: Limoniidae; genus: Dicranophragma; subgenus: Brachylimnophila; specificEpithet: separatum; scientificNameAuthorship: (Walker, 1848); **Location:** country: Poland; stateProvince: Lower Silesia; locality: Glebowice; verbatimElevation: 103m; minimumElevationInMeters: 103; decimalLatitude: 51.4508; decimalLongitude: 16.7386; **Identification:** identifiedBy: E. G. Hancock; **Event:** samplingProtocol: Sweep net; eventDate: 2016-05-23; verbatimEventDate: 23/May/2016; **Record Level:** institutionCode: HMUG; basisOfRecord: PreservedSpecimen
- **Type status:** Other material. **Occurrence:** occurrenceRemarks: 1 male, 1 female; recordedBy: D.I. Gavryushin; individualCount: 2; sex: male, female; occurrenceID: EU_LIM_262; **Taxon:** scientificName: Dicranophragma (Brachylimnophila) separatum (Walker, 1848); family: Limoniidae; genus: Dicranophragma; subgenus: Brachylimnophila; specificEpithet: separatum; scientificNameAuthorship: (Walker, 1848); **Location:** country: Russia; stateProvince: North European Russia; county: Arkhangelsk region; locality: Arkhangelsk; verbatimElevation: 25 m; minimumElevationInMeters: 25; decimalLatitude: 64.55542; decimalLongitude: 40.61068; **Identification:** identifiedBy: D.I. Gavryushin; **Event:** samplingProtocol: Sweep net; eventDate: 2011-08-06; verbatimEventDate: 06/Aug/2011; **Record Level:** institutionCode: ZMMU; basisOfRecord: PreservedSpecimen
- **Type status:** Other material. **Occurrence:** occurrenceRemarks: 1 male; recordedBy: D.I. Gavryushin; individualCount: 1; sex: male; occurrenceID: EU_LIM_263; **Taxon:** scientificName: Dicranophragma (Brachylimnophila) separatum (Walker, 1848); family: Limoniidae; genus: Dicranophragma; subgenus: Brachylimnophila; specificEpithet: separatum; scientificNameAuthorship: (Walker, 1848); **Location:** country: Russia; stateProvince: East European Russia; county: Bashkortostan Respublika; municipality: Beloretsk district; locality: Abzakovo env., Karan River; verbatimElevation: 533 m; minimumElevationInMeters: 533; decimalLatitude: 53.83717; decimalLongitude: 58.57878; **Identification:** identifiedBy: D.I. Gavryushin; **Event:** samplingProtocol: Sweep net; eventDate: 2015-06-17; verbatimEventDate: 17/Jul/2015; **Record Level:** institutionCode: ZMMU; basisOfRecord: PreservedSpecimen
- **Type status:** Other material. **Occurrence:** occurrenceRemarks: 3 males; recordedBy: D.I. Gavryushin; individualCount: 3; sex: male; occurrenceID: EU_LIM_264; **Taxon:** scientificName: Dicranophragma (Brachylimnophila) separatum (Walker, 1848); family: Limoniidae; genus: Dicranophragma; subgenus: Brachylimnophila; specificEpithet: separatum; scientificNameAuthorship: (Walker, 1848); **Location:** country: Russia; stateProvince: East European Russia; county: Bashkortostan Respublika; municipality: Uchaly district; locality: Uzunkul Lake, 1km S of Ozernyi; verbatimElevation: 504 m; minimumElevationInMeters: 504; decimalLatitude: 53.96357; decimalLongitude: 58.85165; **Identification:** identifiedBy: D.I. Gavryushin; **Event:** samplingProtocol: Sweep net; eventDate: 2015-06-14; verbatimEventDate: 14/Jul/2015; **Record Level:** institutionCode: ZMMU; basisOfRecord: PreservedSpecimen
- **Type status:** Other material. **Occurrence:** occurrenceRemarks: 2 males; recordedBy: D.I. Gavryushin; individualCount: 2; sex: male; occurrenceID: EU_LIM_265; **Taxon:** scientificName: Dicranophragma (Brachylimnophila) separatum (Walker, 1848); family: Limoniidae; genus: Dicranophragma; subgenus: Brachylimnophila; specificEpithet: separatum; scientificNameAuthorship: (Walker, 1848); **Location:** country: Russia; stateProvince: East European Russia; county: Bashkortostan Respublika; municipality: Uchaly district; locality: Ural-Tau St. env., upper reaches of Mindyak River; verbatimElevation: 765 m; minimumElevationInMeters: 765; decimalLatitude: 53.96525; decimalLongitude: 58.57888; **Identification:** identifiedBy: D.I. Gavryushin; **Event:** samplingProtocol: Sweep net; eventDate: 2015-06-09; verbatimEventDate: 09/Jul/2015; **Record Level:** institutionCode: ZMMU; basisOfRecord: PreservedSpecimen
- **Type status:** Other material. **Occurrence:** occurrenceRemarks: 3 males, 1 female; recordedBy: D.I. Gavryushin; individualCount: 4; sex: male, female; occurrenceID: EU_LIM_266; **Taxon:** scientificName: Dicranophragma (Brachylimnophila) separatum (Walker, 1848); family: Limoniidae; genus: Dicranophragma; subgenus: Brachylimnophila; specificEpithet: separatum; scientificNameAuthorship: (Walker, 1848); **Location:** country: Russia; stateProvince: East European Russia; county: Bashkortostan Respublika; municipality: Uchaly district; locality: Ural-Tau St. env.; verbatimElevation: 777 m; minimumElevationInMeters: 777; decimalLatitude: 53.96805; decimalLongitude: 58.57613; **Identification:** identifiedBy: D.I. Gavryushin; **Event:** samplingProtocol: Sweep net; eventDate: 2015-06-09; verbatimEventDate: 09/Jul/2015; **Record Level:** institutionCode: ZMMU; basisOfRecord: PreservedSpecimen
- **Type status:** Other material. **Occurrence:** occurrenceRemarks: 1 male, 1 female; recordedBy: D.I. Gavryushin; individualCount: 2; sex: male, female; occurrenceID: EU_LIM_267; **Taxon:** scientificName: Dicranophragma (Brachylimnophila) separatum (Walker, 1848); family: Limoniidae; genus: Dicranophragma; subgenus: Brachylimnophila; specificEpithet: separatum; scientificNameAuthorship: (Walker, 1848); **Location:** country: Russia; stateProvince: East European Russia; county: Bashkortostan Respublika; municipality: Beloretsk district; locality: Nura River (ca. 4km W of Otnurok village), at the foot of Zolotyie Shishki (Golden Cones) Mts.; verbatimElevation: 607 m; minimumElevationInMeters: 607; decimalLatitude: 54.05155; decimalLongitude: 58.26887; **Identification:** identifiedBy: D.I. Gavryushin; **Event:** samplingProtocol: Sweep net; eventDate: 2015-06-10; verbatimEventDate: 10/Jul/2015; **Record Level:** institutionCode: ZMMU; basisOfRecord: PreservedSpecimen
- **Type status:** Other material. **Occurrence:** occurrenceRemarks: 1 female; recordedBy: D.I. Gavryushin; individualCount: 1; sex: female; occurrenceID: EU_LIM_268; **Taxon:** scientificName: Dicranophragma (Brachylimnophila) separatum (Walker, 1848); family: Limoniidae; genus: Dicranophragma; subgenus: Brachylimnophila; specificEpithet: separatum; scientificNameAuthorship: (Walker, 1848); **Location:** country: Russia; stateProvince: East European Russia; county: Bashkortostan Respublika; municipality: Beloretsk district; locality: Makhmutovo env., Belaya River; verbatimElevation: 550 m; minimumElevationInMeters: 550; decimalLatitude: 54.33012; decimalLongitude: 58.80735; **Identification:** identifiedBy: D.I. Gavryushin; **Event:** samplingProtocol: Sweep net; eventDate: 2015-06-15; verbatimEventDate: 15/Jul/2015; **Record Level:** institutionCode: ZMMU; basisOfRecord: PreservedSpecimen
- **Type status:** Other material. **Occurrence:** occurrenceRemarks: 1 male, 2 females; recordedBy: D.I. Gavryushin; individualCount: 3; sex: male, female; occurrenceID: EU_LIM_269; **Taxon:** scientificName: Dicranophragma (Brachylimnophila) separatum (Walker, 1848); family: Limoniidae; genus: Dicranophragma; subgenus: Brachylimnophila; specificEpithet: separatum; scientificNameAuthorship: (Walker, 1848); **Location:** country: Russia; stateProvince: North European Russia; county: Murmansk region; municipality: Kolsky district; locality: Kola; verbatimElevation: 44 m; minimumElevationInMeters: 44; decimalLatitude: 68.8747; decimalLongitude: 33.03777; **Identification:** identifiedBy: D.I. Gavryushin; **Event:** samplingProtocol: Sweep net; eventDate: 2011-06-19; verbatimEventDate: 19/Jul/2011; **Record Level:** institutionCode: ZMMU; basisOfRecord: PreservedSpecimen
- **Type status:** Other material. **Occurrence:** occurrenceRemarks: 1 male; recordedBy: A. Polevoi; individualCount: 1; sex: male; preparations: Pinned; occurrenceID: EU_LIM_270; **Taxon:** scientificName: Dicranophragma (Brachylimnophila) separatum (Walker, 1848); family: Limoniidae; genus: Dicranophragma; subgenus: Brachylimnophila; specificEpithet: separatum; scientificNameAuthorship: (Walker, 1848); **Location:** country: Russia; stateProvince: North European Russia; county: Republic Karelia; municipality: Prionezhskiy district; locality: Sheltozero, 3 km NW; verbatimElevation: 50 m; minimumElevationInMeters: 50; decimalLatitude: 61.39267; decimalLongitude: 35.30831; **Identification:** identifiedBy: A. Polevoi; **Event:** samplingProtocol: Sweep net; eventDate: 2004-06-13; verbatimEventDate: 13/Jul/2004; **Record Level:** institutionCode: FRIP; basisOfRecord: PreservedSpecimen
- **Type status:** Other material. **Occurrence:** occurrenceRemarks: 1 male; recordedBy: A. Polevoi; individualCount: 1; sex: male; preparations: Pinned; occurrenceID: EU_LIM_271; **Taxon:** scientificName: Dicranophragma (Brachylimnophila) separatum (Walker, 1848); family: Limoniidae; genus: Dicranophragma; subgenus: Brachylimnophila; specificEpithet: separatum; scientificNameAuthorship: (Walker, 1848); **Location:** country: Russia; stateProvince: North European Russia; county: Republic Karelia; municipality: Lahdenpohja district; locality: Sikopohja, 9 km NE; verbatimElevation: 80 m; minimumElevationInMeters: 80; decimalLatitude: 61.68749; decimalLongitude: 30.16007; **Identification:** identifiedBy: A. Polevoi; **Event:** samplingProtocol: Sweep net; eventDate: 2005-06-07; verbatimEventDate: 07/Jul/2005; **Record Level:** institutionCode: FRIP; basisOfRecord: PreservedSpecimen
- **Type status:** Other material. **Occurrence:** occurrenceRemarks: 1 male; recordedBy: A. Polevoi; individualCount: 1; sex: male; preparations: Pinned; occurrenceID: EU_LIM_272; **Taxon:** scientificName: Dicranophragma (Brachylimnophila) separatum (Walker, 1848); family: Limoniidae; genus: Dicranophragma; subgenus: Brachylimnophila; specificEpithet: separatum; scientificNameAuthorship: (Walker, 1848); **Location:** country: Russia; stateProvince: North European Russia; county: Republic Karelia; municipality: Pudozh district; locality: Prirechnyi, 3 km SW; verbatimElevation: 140 m; minimumElevationInMeters: 140; decimalLatitude: 61.77809; decimalLongitude: 37.58233; **Identification:** identifiedBy: A. Polevoi; **Event:** samplingProtocol: Malaise trap; eventDate: 2009-06-24/2009-08-13; verbatimEventDate: 24/Jun-13/Aug/2009; **Record Level:** institutionCode: FRIP; basisOfRecord: PreservedSpecimen
- **Type status:** Other material. **Occurrence:** occurrenceRemarks: 1 male; recordedBy: A. Polevoi; individualCount: 1; sex: male; preparations: Pinned; occurrenceID: EU_LIM_273; **Taxon:** scientificName: Dicranophragma (Brachylimnophila) separatum (Walker, 1848); family: Limoniidae; genus: Dicranophragma; subgenus: Brachylimnophila; specificEpithet: separatum; scientificNameAuthorship: (Walker, 1848); **Location:** country: Russia; stateProvince: North European Russia; county: Republic Karelia; municipality: Muezerskiy district; locality: Murdoyoki River; verbatimElevation: 215 m; minimumElevationInMeters: 215; decimalLatitude: 64.20192; decimalLongitude: 30.86608; **Identification:** identifiedBy: A. Polevoi; **Event:** samplingProtocol: Sweep net; eventDate: 2009-06-03; verbatimEventDate: 03/Jul/2009; **Record Level:** institutionCode: FRIP; basisOfRecord: PreservedSpecimen
- **Type status:** Other material. **Occurrence:** occurrenceRemarks: 1 male; recordedBy: A. Polevoi; individualCount: 1; sex: male; preparations: Pinned; occurrenceID: EU_LIM_274; **Taxon:** scientificName: Dicranophragma (Brachylimnophila) separatum (Walker, 1848); family: Limoniidae; genus: Dicranophragma; subgenus: Brachylimnophila; specificEpithet: separatum; scientificNameAuthorship: (Walker, 1848); **Location:** country: Russia; stateProvince: North European Russia; county: Republic Karelia; municipality: Kem' district; locality: White Sea shore, 2 km NW of Island Syrovatka; verbatimElevation: 25 m; minimumElevationInMeters: 25; decimalLatitude: 65.52825; decimalLongitude: 34.72966; **Identification:** identifiedBy: A. Polevoi; **Event:** samplingProtocol: Sweep net; eventDate: 2003-06-16; verbatimEventDate: 16/Jul/2003; **Record Level:** institutionCode: FRIP; basisOfRecord: PreservedSpecimen
- **Type status:** Other material. **Occurrence:** occurrenceRemarks: 1 male; recordedBy: N.M. Paramonov; individualCount: 1; sex: male; occurrenceID: EU_LIM_275; **Taxon:** scientificName: Dicranophragma (Brachylimnophila) separatum (Walker, 1848); family: Limoniidae; genus: Dicranophragma; subgenus: Brachylimnophila; specificEpithet: separatum; scientificNameAuthorship: (Walker, 1848); **Location:** country: Russia; stateProvince: East European Russia; county: Tatarstan Respublika; municipality: Zelenodol’sk district; locality: Ilinskoe; verbatimElevation: 90 m; minimumElevationInMeters: 90; decimalLatitude: 55.87455; decimalLongitude: 48.68579; **Identification:** identifiedBy: N.M. Paramonov; **Event:** samplingProtocol: Sweep net; eventDate: 2012-06-29; verbatimEventDate: 29/Jun/2012; habitat: village env.; **Record Level:** institutionCode: ZIN; basisOfRecord: PreservedSpecimen
- **Type status:** Other material. **Occurrence:** occurrenceRemarks: 1 male; recordedBy: L.-P. Kolcsár | E. Török; individualCount: 1; sex: male; preparations: Ethanol; occurrenceID: EU_LIM_276; **Taxon:** scientificName: Dicranophragma (Brachylimnophila) separatum (Walker, 1848); family: Limoniidae; genus: Dicranophragma; subgenus: Brachylimnophila; specificEpithet: separatum; scientificNameAuthorship: (Walker, 1848); **Location:** country: Serbia; municipality: Kopaonik; locality: Kopaonik Mts.; verbatimElevation: 1556 m; minimumElevationInMeters: 1556; decimalLatitude: 43.30997; decimalLongitude: 20.76563; **Identification:** identifiedBy: L.-P. Kolcsár; **Event:** samplingProtocol: Sweep net; eventDate: 2017-06-22; verbatimEventDate: 22/Jun/2017; **Record Level:** institutionCode: CKLP; basisOfRecord: PreservedSpecimen

#### Distribution

First records from Belarus, Hungary, Latvia, Poland, Russia: RUE and Serbia. We confirm the presence of the species in Russia: RUN.

### Dicranoptycha livescens

Loew, 1871

8AE56D05-D96D-595C-9790-542C9EC81EC2

https://ccw.naturalis.nl/detail.php?id=9274

#### Materials

- **Type status:** Other material. **Occurrence:** occurrenceRemarks: 2 males, 21 females; recordedBy: N.M. Paramonov; individualCount: 23; sex: male, female; occurrenceID: EU_LIM_277; **Taxon:** scientificName: Dicranoptychalivescens Loew, 1871; family: Limoniidae; genus: Dicranoptycha; specificEpithet: livescens; scientificNameAuthorship: Loew, 1871; **Location:** country: Russia; stateProvince: East European Russia; county: Tatarstan Respublika; municipality: Zelenodol’sk district; locality: Zaymishche env., Geomagnetic station; verbatimElevation: 87 m; minimumElevationInMeters: 87; decimalLatitude: 55.82684; decimalLongitude: 48.84395; **Identification:** identifiedBy: N.M. Paramonov; **Event:** samplingProtocol: Sweep net; eventDate: 2011-08-19/2011-08-21; verbatimEventDate: 19-21/Aug/2011; **Record Level:** institutionCode: ZIN; basisOfRecord: PreservedSpecimen
- **Type status:** Other material. **Occurrence:** occurrenceRemarks: 1 male; recordedBy: Fürjes; individualCount: 1; sex: male; preparations: Pinned; occurrenceID: EU_LIM_278; **Taxon:** scientificName: Dicranoptychaparalivescens Starý, 1972; family: Limoniidae; genus: Dicranoptycha; specificEpithet: paralivescens; scientificNameAuthorship: Starý, 1972; **Location:** country: Hungary; stateProvince: Tolna; municipality: Őcsény; locality: Gemenc forest, Rezéti-Holt-Duna; decimalLatitude: 46.24454; decimalLongitude: 18.87574; **Identification:** identifiedBy: L.-P. Kolcsár; **Event:** eventDate: 1982-06-07; verbatimEventDate: 07/Jul/1982; **Record Level:** institutionCode: HNHM; basisOfRecord: PreservedSpecimen
- **Type status:** Other material. **Occurrence:** occurrenceRemarks: 1 male; recordedBy: V.E. Pilipenko; individualCount: 1; sex: male; occurrenceID: EU_LIM_279; **Taxon:** scientificName: Dicranoptychaparalivescens Starý, 1972; family: Limoniidae; genus: Dicranoptycha; specificEpithet: paralivescens; scientificNameAuthorship: Starý, 1972; **Location:** country: Russia; stateProvince: East European Russia; county: Bashkortostan Respublika; locality: S. Ural, Belaya River (White River), 5 km E Muratovo; verbatimElevation: 290 m; minimumElevationInMeters: 290; decimalLatitude: 53.0038; decimalLongitude: 57.17075; **Identification:** identifiedBy: V.E. Pilipenko; **Event:** samplingProtocol: Sweep net; eventDate: 2001-06-09; verbatimEventDate: 09/Jul/2001; **Record Level:** basisOfRecord: PreservedSpecimen

#### Distribution

First record from Russia: RUE.

### Dicranoptycha paralivescens

Starý, 1972

92A17698-3C6C-5A95-89EB-D3E5CC3DAB96

https://ccw.naturalis.nl/detail.php?id=9294

#### Materials

- **Type status:** Other material. **Occurrence:** occurrenceRemarks: 1 male; recordedBy: Fürjes; individualCount: 1; sex: male; preparations: Pinned; occurrenceID: EU_LIM_278; **Taxon:** scientificName: Dicranoptychaparalivescens Starý, 1972; family: Limoniidae; genus: Dicranoptycha; specificEpithet: paralivescens; scientificNameAuthorship: Starý, 1972; **Location:** country: Hungary; stateProvince: Tolna; municipality: Őcsény; locality: Gemenc forest, Rezéti-Holt-Duna; decimalLatitude: 46.24454; decimalLongitude: 18.87574; **Identification:** identifiedBy: L.-P. Kolcsár; **Event:** eventDate: 1982-06-07; verbatimEventDate: 07/Jul/1982; **Record Level:** institutionCode: HNHM; basisOfRecord: PreservedSpecimen
- **Type status:** Other material. **Occurrence:** occurrenceRemarks: 1 male; recordedBy: V.E. Pilipenko; individualCount: 1; sex: male; occurrenceID: EU_LIM_279; **Taxon:** scientificName: Dicranoptychaparalivescens Starý, 1972; family: Limoniidae; genus: Dicranoptycha; specificEpithet: paralivescens; scientificNameAuthorship: Starý, 1972; **Location:** country: Russia; stateProvince: East European Russia; county: Bashkortostan Respublika; locality: S. Ural, Belaya River (White River), 5 km E Muratovo; verbatimElevation: 290 m; minimumElevationInMeters: 290; decimalLatitude: 53.0038; decimalLongitude: 57.17075; **Identification:** identifiedBy: V.E. Pilipenko; **Event:** samplingProtocol: Sweep net; eventDate: 2001-06-09; verbatimEventDate: 09/Jul/2001; **Record Level:** basisOfRecord: PreservedSpecimen

#### Distribution

First records from Hungary and Russia: RUE.

Records of *Dicranoptychaparalivescens* from Europan part of Turkey reported by Koç et al. (2016) are misidentifications and refer to *Dicranoptychalivescens*, based on the published wing and genital photos.

### Discobola annulata

(Linnaeus, 1758)

F3D96CA0-5E62-5540-B4AF-88F1858426AA

https://ccw.naturalis.nl/detail.php?id=9330

#### Materials

- **Type status:** Other material. **Occurrence:** occurrenceRemarks: 1 female; recordedBy: L.-P. Kolcsár; individualCount: 1; sex: female; preparations: Ethanol; occurrenceID: EU_LIM_280; **Taxon:** scientificName: Discobolaannulata (Linnaeus, 1758); family: Limoniidae; genus: Discobola; specificEpithet: annulata; scientificNameAuthorship: (Linnaeus, 1758); **Location:** country: Latvia; municipality: Sigulda; locality: Gauja River; verbatimElevation: 13 m; minimumElevationInMeters: 13; decimalLatitude: 57.1505; decimalLongitude: 24.8168; **Identification:** identifiedBy: L.-P. Kolcsár; **Event:** samplingProtocol: Sweep net; eventDate: 2018-06-24; verbatimEventDate: 24/Jul/2018; **Record Level:** institutionCode: CKLP; basisOfRecord: PreservedSpecimen
- **Type status:** Other material. **Occurrence:** occurrenceRemarks: 1 female; recordedBy: D.I. Gavryushin; individualCount: 1; sex: female; occurrenceID: EU_LIM_281; **Taxon:** scientificName: Discobolaannulata (Linnaeus, 1758); family: Limoniidae; genus: Discobola; specificEpithet: annulata; scientificNameAuthorship: (Linnaeus, 1758); **Location:** country: Russia; stateProvince: East European Russia; county: Bashkortostan Respublika; municipality: Beloretsk district; locality: Abzakovo env., Malyi Kizil River; verbatimElevation: 510 m; minimumElevationInMeters: 510; decimalLatitude: 53.81428; decimalLongitude: 58.5942; **Identification:** identifiedBy: D.I. Gavryushin; **Event:** samplingProtocol: Sweep net; eventDate: 2015-06-12; verbatimEventDate: 12/Jul/2015; **Record Level:** institutionCode: ZMMU; basisOfRecord: PreservedSpecimen
- **Type status:** Other material. **Occurrence:** occurrenceRemarks: 1 male; recordedBy: D.I. Gavryushin; individualCount: 1; sex: male; occurrenceID: EU_LIM_282; **Taxon:** scientificName: Discobolaannulata (Linnaeus, 1758); family: Limoniidae; genus: Discobola; specificEpithet: annulata; scientificNameAuthorship: (Linnaeus, 1758); **Location:** country: Russia; stateProvince: East European Russia; county: Bashkortostan Respublika; municipality: Beloretsk district; locality: Abzakovo env., Karan River; verbatimElevation: 533 m; minimumElevationInMeters: 533; decimalLatitude: 53.83717; decimalLongitude: 58.57878; **Identification:** identifiedBy: D.I. Gavryushin; **Event:** samplingProtocol: Sweep net; eventDate: 2015-06-19; verbatimEventDate: 19/Jul/2015; **Record Level:** institutionCode: ZMMU; basisOfRecord: PreservedSpecimen
- **Type status:** Other material. **Occurrence:** occurrenceRemarks: 2 males; recordedBy: D.I. Gavryushin; individualCount: 2; sex: male; occurrenceID: EU_LIM_283; **Taxon:** scientificName: Discobolaannulata (Linnaeus, 1758); family: Limoniidae; genus: Discobola; specificEpithet: annulata; scientificNameAuthorship: (Linnaeus, 1758); **Location:** country: Russia; stateProvince: East European Russia; county: Bashkortostan Respublika; municipality: Beloretsk district; locality: Nura River (ca. 4km W of Otnurok village), at the foot of Zolotyie Shishki (Golden Cones) Mts.; verbatimElevation: 607 m; minimumElevationInMeters: 607; decimalLatitude: 54.05155; decimalLongitude: 58.26887; **Identification:** identifiedBy: D.I. Gavryushin; **Event:** samplingProtocol: Sweep net; eventDate: 2012-08-09; verbatimEventDate: 09/Aug/2012; **Record Level:** institutionCode: ZMMU; basisOfRecord: PreservedSpecimen
- **Type status:** Other material. **Occurrence:** occurrenceRemarks: 3 males; recordedBy: D.I. Gavryushin; individualCount: 3; sex: male; occurrenceID: EU_LIM_284; **Taxon:** scientificName: Discobolaannulata (Linnaeus, 1758); family: Limoniidae; genus: Discobola; specificEpithet: annulata; scientificNameAuthorship: (Linnaeus, 1758); **Location:** country: Russia; stateProvince: East European Russia; county: Bashkortostan Respublika; municipality: Beloretsk district; locality: Nura River (ca. 4km W of Otnurok village), at the foot of Zolotyie Shishki (Golden Cones) Mts.; verbatimElevation: 607 m; minimumElevationInMeters: 607; decimalLatitude: 54.05155; decimalLongitude: 58.26887; **Identification:** identifiedBy: D.I. Gavryushin; **Event:** samplingProtocol: Sweep net; eventDate: 2012-08-08; verbatimEventDate: 08/Aug/2012; **Record Level:** institutionCode: ZMMU; basisOfRecord: PreservedSpecimen
- **Type status:** Other material. **Occurrence:** occurrenceRemarks: 1 female; recordedBy: D.I. Gavryushin; individualCount: 1; sex: female; occurrenceID: EU_LIM_285; **Taxon:** scientificName: Discobolaannulata (Linnaeus, 1758); family: Limoniidae; genus: Discobola; specificEpithet: annulata; scientificNameAuthorship: (Linnaeus, 1758); **Location:** country: Russia; stateProvince: East European Russia; county: Bashkortostan Respublika; municipality: Beloretsk district; locality: Nura River (ca. 4km W of Otnurok village), at the foot of Zolotyie Shishki (Golden Cones) Mts.; verbatimElevation: 607 m; minimumElevationInMeters: 607; decimalLatitude: 54.05155; decimalLongitude: 58.26887; **Identification:** identifiedBy: D.I. Gavryushin; **Event:** samplingProtocol: Sweep net; eventDate: 2012-08-11; verbatimEventDate: 11/Aug/2012; **Record Level:** institutionCode: ZMMU; basisOfRecord: PreservedSpecimen
- **Type status:** Other material. **Occurrence:** occurrenceRemarks: 1 female; recordedBy: D.I. Gavryushin; individualCount: 1; sex: female; occurrenceID: EU_LIM_286; **Taxon:** scientificName: Discobolaannulata (Linnaeus, 1758); family: Limoniidae; genus: Discobola; specificEpithet: annulata; scientificNameAuthorship: (Linnaeus, 1758); **Location:** country: Russia; stateProvince: East European Russia; county: Bashkortostan Respublika; municipality: Beloretsk district; locality: Nura River (ca. 2km NW of Nura village); verbatimElevation: 650 m; minimumElevationInMeters: 650; decimalLatitude: 54.07352; decimalLongitude: 58.29457; **Identification:** identifiedBy: D.I. Gavryushin; **Event:** samplingProtocol: Sweep net; eventDate: 2012-08-13; verbatimEventDate: 13/Aug/2012; **Record Level:** institutionCode: ZMMU; basisOfRecord: PreservedSpecimen
- **Type status:** Other material. **Occurrence:** occurrenceRemarks: 1 male; recordedBy: D.I. Gavryushin; individualCount: 1; sex: male; occurrenceID: EU_LIM_287; **Taxon:** scientificName: Discobolaannulata (Linnaeus, 1758); family: Limoniidae; genus: Discobola; specificEpithet: annulata; scientificNameAuthorship: (Linnaeus, 1758); **Location:** country: Russia; stateProvince: East European Russia; county: Bashkortostan Respublika; municipality: Beloretsk district; locality: Makhmutovo env., Belaya River; verbatimElevation: 550 m; minimumElevationInMeters: 550; decimalLatitude: 54.33012; decimalLongitude: 58.80735; **Identification:** identifiedBy: D.I. Gavryushin; **Event:** samplingProtocol: Sweep net; eventDate: 2015-06-15; verbatimEventDate: 15/Jul/2015; **Record Level:** institutionCode: ZMMU; basisOfRecord: PreservedSpecimen

#### Distribution

First records from Latvia and Russia: RUE.

### Discobola caesarea

(Osten Sacken, 1854)

1E16B412-F459-5EF6-94AB-D9F0EE58ECD6

https://ccw.naturalis.nl/detail.php?id=9335

#### Materials

- **Type status:** Other material. **Occurrence:** occurrenceRemarks: 1 female; individualCount: 1; sex: female; preparations: Pinned; occurrenceID: EU_LIM_288; **Taxon:** scientificName: Discobolacaesarea (Osten Sacken, 1854); family: Limoniidae; genus: Discobola; specificEpithet: caesarea; scientificNameAuthorship: (Osten Sacken, 1854); **Location:** country: Hungary; stateProvince: Somogy; municipality: Zákány; decimalLatitude: 46.26151; decimalLongitude: 16.95126; **Identification:** identifiedBy: L.-P. Kolcsár; **Event:** eventDate: 1982-06-24; verbatimEventDate: 24/Jun/1982; **Record Level:** institutionCode: HNHM; basisOfRecord: PreservedSpecimen
- **Type status:** Other material. **Occurrence:** catalogNumber: 552054; occurrenceRemarks: 1 male; recordedBy: K.M. Olsen; individualCount: 1; sex: male; preparations: Ethanol; occurrenceID: EU_LIM_289; **Taxon:** scientificName: Discobolacaesarea (Osten Sacken, 1854); family: Limoniidae; genus: Discobola; specificEpithet: caesarea; scientificNameAuthorship: (Osten Sacken, 1854); **Location:** country: Norway; stateProvince: Buskerud; municipality: Lier; locality: Linnesstranda E; verbatimElevation: 1 m; minimumElevationInMeters: 1; decimalLatitude: 59.74775; decimalLongitude: 10.28775; **Identification:** identifiedBy: K.M. Olsen; **Event:** samplingProtocol: Sweep net; eventDate: 2017-08-11; verbatimEventDate: 11/Aug/2017; **Record Level:** institutionCode: NHMO; basisOfRecord: PreservedSpecimen
- **Type status:** Other material. **Occurrence:** catalogNumber: 641155; occurrenceRemarks: 1 male; recordedBy: K. Sund; individualCount: 1; sex: male; preparations: Ethanol; occurrenceID: EU_LIM_290; **Taxon:** scientificName: Discobolacaesarea (Osten Sacken, 1854); family: Limoniidae; genus: Discobola; specificEpithet: caesarea; scientificNameAuthorship: (Osten Sacken, 1854); **Location:** country: Norway; stateProvince: Hedmark; municipality: Kongsvinger; locality: Dragonmoen; verbatimElevation: 196 m; minimumElevationInMeters: 196; decimalLatitude: 60.19641; decimalLongitude: 12.36511; **Identification:** identifiedBy: K.M. Olsen; **Event:** samplingProtocol: Malaise trap; eventDate: 2005-08-14/2005-09-06; verbatimEventDate: 14/Aug-06/Sep/2005; **Record Level:** institutionCode: NHMO; basisOfRecord: PreservedSpecimen
- **Type status:** Other material. **Occurrence:** catalogNumber: 514559; occurrenceRemarks: 1 male; recordedBy: O.J. Lønnve; individualCount: 1; sex: male; preparations: Ethanol; occurrenceID: EU_LIM_291; **Taxon:** scientificName: Discobolacaesarea (Osten Sacken, 1854); family: Limoniidae; genus: Discobola; specificEpithet: caesarea; scientificNameAuthorship: (Osten Sacken, 1854); **Location:** country: Norway; stateProvince: Oppland; municipality: Lunner; locality: Grindvoll, Vestern – Vestby; verbatimElevation: 300 m; minimumElevationInMeters: 300; decimalLatitude: 60.30357; decimalLongitude: 10.46949; **Identification:** identifiedBy: K.M. Olsen; **Event:** samplingProtocol: Malaise trap; eventDate: 1994-09-18/1994-10-09; verbatimEventDate: 18/Sep-09/Oct/1994; **Record Level:** institutionCode: PCKMO; basisOfRecord: PreservedSpecimen
- **Type status:** Other material. **Occurrence:** catalogNumber: 476508; occurrenceRemarks: 1 male; recordedBy: K.M. Olsen; individualCount: 1; sex: male; preparations: Ethanol; occurrenceID: EU_LIM_292; **Taxon:** scientificName: Discobolacaesarea (Osten Sacken, 1854); family: Limoniidae; genus: Discobola; specificEpithet: caesarea; scientificNameAuthorship: (Osten Sacken, 1854); **Location:** country: Norway; stateProvince: Telemark; municipality: Seljord; locality: Lindviki; verbatimElevation: 120-201 m; minimumElevationInMeters: 120; maximumElevationInMeters: 201; decimalLatitude: 59.45099; decimalLongitude: 8.69994; **Identification:** identifiedBy: J. Salmela; **Event:** samplingProtocol: Sweep net; eventDate: 2016-08-28; verbatimEventDate: 28/Aug/2016; habitat: Opp langs vei og bekk; **Record Level:** institutionCode: PCKMO; basisOfRecord: PreservedSpecimen

#### Distribution

First records from Norway. We confirm the presence of the species in Hungary.

### Discobola parvispinula

(Alexander, 1947)

CF578998-06B0-5738-80BF-5758BAD78624

https://ccw.naturalis.nl/detail.php?id=9357

#### Materials

- **Type status:** Other material. **Occurrence:** occurrenceRemarks: 1 male; recordedBy: L.-P. Kolcsár; individualCount: 1; sex: male; preparations: Ethanol; occurrenceID: EU_LIM_293; **Taxon:** scientificName: Discobolaparvispinula (Alexander, 1947); family: Limoniidae; genus: Discobola; specificEpithet: parvispinula; scientificNameAuthorship: (Alexander, 1947); **Location:** country: Romania; stateProvince: Harghita; municipality: Ditrău; locality: Depresiunea Giurgeului, village; verbatimElevation: 757 m; minimumElevationInMeters: 757; decimalLatitude: 46.80781; decimalLongitude: 25.50682; **Identification:** identifiedBy: L.-P. Kolcsár; **Event:** samplingProtocol: Sweep net; eventDate: 2010-06-17; verbatimEventDate: 17/Jun/2010; **Record Level:** institutionCode: CKLP; basisOfRecord: PreservedSpecimen
- **Type status:** Other material. **Occurrence:** occurrenceRemarks: 1 male; recordedBy: L.-P. Kolcsár; individualCount: 1; sex: male; preparations: Ethanol; occurrenceID: EU_LIM_294; **Taxon:** scientificName: Discobolaparvispinula (Alexander, 1947); family: Limoniidae; genus: Discobola; specificEpithet: parvispinula; scientificNameAuthorship: (Alexander, 1947); **Location:** country: Romania; stateProvince: Harghita; municipality: Ditrău; locality: Depresiunea Giurgeului, village; verbatimElevation: 757 m; minimumElevationInMeters: 757; decimalLatitude: 46.80781; decimalLongitude: 25.50682; **Identification:** identifiedBy: L.-P. Kolcsár; **Event:** samplingProtocol: Sweep net; eventDate: 2011-08-10; verbatimEventDate: 10/Aug/2011; **Record Level:** institutionCode: CKLP; basisOfRecord: PreservedSpecimen
- **Type status:** Other material. **Occurrence:** occurrenceRemarks: 1 female; recordedBy: D.I. Gavryushin; individualCount: 1; sex: female; occurrenceID: EU_LIM_295; **Taxon:** scientificName: Discobolaparvispinula (Alexander, 1947); family: Limoniidae; genus: Discobola; specificEpithet: parvispinula; scientificNameAuthorship: (Alexander, 1947); **Location:** country: Russia; stateProvince: East European Russia; county: Bashkortostan Respublika; municipality: Beloretsk district; locality: Nura River; verbatimElevation: 494 m; minimumElevationInMeters: 494; decimalLatitude: 53.97365; decimalLongitude: 58.34415; **Identification:** identifiedBy: D.I. Gavryushin; **Event:** samplingProtocol: Sweep net; eventDate: 2012-08-10; verbatimEventDate: 10/Aug/2012; **Record Level:** institutionCode: ZMMU; basisOfRecord: PreservedSpecimen
- **Type status:** Other material. **Occurrence:** occurrenceRemarks: 1 male; recordedBy: D.I. Gavryushin; individualCount: 1; sex: male; occurrenceID: EU_LIM_296; **Taxon:** scientificName: Discobolaparvispinula (Alexander, 1947); family: Limoniidae; genus: Discobola; specificEpithet: parvispinula; scientificNameAuthorship: (Alexander, 1947); **Location:** country: Russia; stateProvince: East European Russia; county: Bashkortostan Respublika; municipality: Beloretsk district; locality: Nura River (ca. 4km W of Otnurok village), at the foot of Zolotyie Shishki (Golden Cones) Mts.; verbatimElevation: 607 m; minimumElevationInMeters: 607; decimalLatitude: 54.05155; decimalLongitude: 58.26887; **Identification:** identifiedBy: D.I. Gavryushin; **Event:** samplingProtocol: Sweep net; eventDate: 2012-08-13; verbatimEventDate: 13/Aug/2012; **Record Level:** institutionCode: ZMMU; basisOfRecord: PreservedSpecimen
- **Type status:** Other material. **Occurrence:** occurrenceRemarks: 3 males; recordedBy: D.I. Gavryushin; individualCount: 3; sex: male; occurrenceID: EU_LIM_297; **Taxon:** scientificName: Discobolaparvispinula (Alexander, 1947); family: Limoniidae; genus: Discobola; specificEpithet: parvispinula; scientificNameAuthorship: (Alexander, 1947); **Location:** country: Russia; stateProvince: East European Russia; county: Bashkortostan Respublika; municipality: Beloretsk district; locality: Makhmutovo env., Belaya River; verbatimElevation: 550 m; minimumElevationInMeters: 550; decimalLatitude: 54.33012; decimalLongitude: 58.80735; **Identification:** identifiedBy: D.I. Gavryushin; **Event:** samplingProtocol: Sweep net; eventDate: 2015-06-19; verbatimEventDate: 19/Jul/2015; **Record Level:** institutionCode: ZMMU; basisOfRecord: PreservedSpecimen

#### Distribution

First records from Romania and Russia: RUE.

### Elephantomyia (Elephantomyia) edwardsi

Lackschewitz, 1932

29D2368A-EF07-5FCE-A585-1C5DF6E9EDAB

https://ccw.naturalis.nl/detail.php?id=9393

#### Materials

- **Type status:** Other material. **Occurrence:** catalogNumber: 527948; occurrenceRemarks: 2 females; recordedBy: K.M. Olsen; individualCount: 2; sex: female; preparations: Ethanol; occurrenceID: EU_LIM_298; **Taxon:** scientificName: Elephantomyia (Elephantomyia) edwardsi Lackschewitz, 1932; family: Limoniidae; genus: Elephantomyia; subgenus: Elephantomyia; specificEpithet: edwardsi; scientificNameAuthorship: Lackschewitz, 1932; **Location:** country: Norway; stateProvince: Akershus; municipality: Skedsmo; locality: N Asak Mellom; verbatimElevation: 150 m; minimumElevationInMeters: 150; decimalLatitude: 59.98733; decimalLongitude: 11.10011; **Identification:** identifiedBy: K.M. Olsen; **Event:** samplingProtocol: Sweep net; eventDate: 2017-06-20; verbatimEventDate: 20/Jun/2017; **Record Level:** institutionCode: NHMO; basisOfRecord: PreservedSpecimen
- **Type status:** Other material. **Occurrence:** catalogNumber: 526231, 644172; occurrenceRemarks: 2 males; recordedBy: K.M. Olsen mfl.; individualCount: 2; sex: male; preparations: Ethanol; occurrenceID: EU_LIM_299; **Taxon:** scientificName: Elephantomyia (Elephantomyia) edwardsi Lackschewitz, 1932; family: Limoniidae; genus: Elephantomyia; subgenus: Elephantomyia; specificEpithet: edwardsi; scientificNameAuthorship: Lackschewitz, 1932; **Location:** country: Norway; stateProvince: Vestfold; municipality: Larvik; locality: NE Eineren; verbatimElevation: 8 m; minimumElevationInMeters: 8; decimalLatitude: 58.97812; decimalLongitude: 9.88454; **Identification:** identifiedBy: K.M. Olsen; **Event:** samplingProtocol: Sweep net; eventDate: 2017-06-08; verbatimEventDate: 08/Jun/2017; **Record Level:** institutionCode: PCKMO; basisOfRecord: PreservedSpecimen
- **Type status:** Other material. **Occurrence:** catalogNumber: 515744; occurrenceRemarks: 2 females; recordedBy: S. Olberg; individualCount: 2; sex: female; preparations: Ethanol; occurrenceID: EU_LIM_300; **Taxon:** scientificName: Elephantomyia (Elephantomyia) edwardsi Lackschewitz, 1932; family: Limoniidae; genus: Elephantomyia; subgenus: Elephantomyia; specificEpithet: edwardsi; scientificNameAuthorship: Lackschewitz, 1932; **Location:** country: Norway; stateProvince: Vestfold; municipality: Larvik; locality: Småås; verbatimElevation: 75 m; minimumElevationInMeters: 75; decimalLatitude: 59.21133; decimalLongitude: 10.01027; **Identification:** identifiedBy: K.M. Olsen; **Event:** samplingProtocol: Malaise trap; eventDate: 2014-06-18/2014-07-10; verbatimEventDate: 18/Jun-10/Jul/2014; **Record Level:** institutionCode: PCKMO; basisOfRecord: PreservedSpecimen
- **Type status:** Other material. **Occurrence:** occurrenceRemarks: 1 male; recordedBy: M. Lindström; individualCount: 1; sex: male; preparations: Ethanol; occurrenceID: EU_LIM_301; **Taxon:** scientificName: Elephantomyia (Elephantomyia) edwardsi Lackschewitz, 1932; family: Limoniidae; genus: Elephantomyia; subgenus: Elephantomyia; specificEpithet: edwardsi; scientificNameAuthorship: Lackschewitz, 1932; **Location:** country: Sweden; stateProvince: Halland; municipality: Halmstad; locality: Almeberget, Slättåkra; verbatimElevation: 100 m; minimumElevationInMeters: 100; decimalLatitude: 56.85344; decimalLongitude: 12.89848; **Identification:** identifiedBy: M. Lindström; **Event:** samplingProtocol: Malaise trap; eventDate: 2011-05-05/2011-07-19; verbatimEventDate: 5/May-19/Jul/2011; **Record Level:** basisOfRecord: PreservedSpecimen
- **Type status:** Other material. **Occurrence:** occurrenceRemarks: 1 female; recordedBy: B. Viklund | L-O. Wikars | H. Ahnlund; individualCount: 1; sex: female; preparations: Pinned; occurrenceID: EU_LIM_302; **Taxon:** scientificName: Elephantomyia (Elephantomyia) edwardsi Lackschewitz, 1932; family: Limoniidae; genus: Elephantomyia; subgenus: Elephantomyia; specificEpithet: edwardsi; scientificNameAuthorship: Lackschewitz, 1932; **Location:** country: Sweden; stateProvince: Södermanland; municipality: Tyresö; locality: Tyresta National Park, omr. 3; decimalLatitude: 59.17844; decimalLongitude: 18.31086; **Identification:** identifiedBy: J. Kramer; **Event:** samplingProtocol: Malaise trap; eventDate: 2000-06-19/2000-07-28; verbatimEventDate: 19/Jun-28/Jul/2000; **Record Level:** institutionCode: NHRS; basisOfRecord: PreservedSpecimen
- **Type status:** Other material. **Occurrence:** occurrenceRemarks: 1 male; recordedBy: B. Viklund | L-O. Wikars | H. Ahnlund; individualCount: 1; sex: male; preparations: Pinned; occurrenceID: EU_LIM_303; **Taxon:** scientificName: Elephantomyia (Elephantomyia) edwardsi Lackschewitz, 1932; family: Limoniidae; genus: Elephantomyia; subgenus: Elephantomyia; specificEpithet: edwardsi; scientificNameAuthorship: Lackschewitz, 1932; **Location:** country: Sweden; stateProvince: Södermanland; municipality: Tyresö; locality: Tyresta National Park, omr. 5; decimalLatitude: 59.17844; decimalLongitude: 18.31086; **Identification:** identifiedBy: J. Kramer; **Event:** samplingProtocol: Malaise trap; eventDate: 2000-06-19/2000-07-28; verbatimEventDate: 19/Jun-28/Jul/2000; **Record Level:** institutionCode: NHRS; basisOfRecord: PreservedSpecimen
- **Type status:** Other material. **Occurrence:** occurrenceRemarks: 1 male; recordedBy: M. Oomen | S. Lemurell | U. Unger | M. Unger | O. Bäckman; individualCount: 1; sex: male; occurrenceID: EU_LIM_304; **Taxon:** scientificName: Elephantomyia (Elephantomyia) edwardsi Lackschewitz, 1932; family: Limoniidae; genus: Elephantomyia; subgenus: Elephantomyia; specificEpithet: edwardsi; scientificNameAuthorship: Lackschewitz, 1932; **Location:** country: Sweden; stateProvince: Västergötland; municipality: Härryda; locality: Klippan Nature Reserve, Hindås; verbatimElevation: 50 m; minimumElevationInMeters: 50; decimalLatitude: 57.68917; decimalLongitude: 12.48149; **Identification:** identifiedBy: M. Andersson; **Event:** samplingProtocol: To light; eventDate: 2017-06-05/2017-07-06; verbatimEventDate: 5-6/Jul/2017; **Record Level:** basisOfRecord: HumanObservation
- **Type status:** Other material. **Occurrence:** occurrenceRemarks: 1 female; recordedBy: M. Oomen | S. Lemurell | U. Unger | M. Unger | O. Bäckman; individualCount: 1; sex: female; preparations: Pinned; occurrenceID: EU_LIM_305; **Taxon:** scientificName: Elephantomyia (Elephantomyia) edwardsi Lackschewitz, 1932; family: Limoniidae; genus: Elephantomyia; subgenus: Elephantomyia; specificEpithet: edwardsi; scientificNameAuthorship: Lackschewitz, 1932; **Location:** country: Sweden; stateProvince: Västergötland; municipality: Härryda; locality: Klippan Nature Reserve, Hindås; verbatimElevation: 50 m; minimumElevationInMeters: 50; decimalLatitude: 57.68917; decimalLongitude: 12.48149; **Identification:** identifiedBy: M. Andersson; **Event:** samplingProtocol: To light; eventDate: 2018-06-02/2018-07-03; verbatimEventDate: 2-3/Jul/2018; **Record Level:** institutionCode: NHRS; basisOfRecord: PreservedSpecimen

#### Distribution

The species removed from the Swedish checklist by Salmela (2011a), but presence recently confirmed, based on records published on Artportalen.se (Artportalen 2017); here, we republish these confirmatory records.

### Elephantomyia (Elephantomyia) krivosheinae

Savchenko, 1976

C2A65E22-0215-5086-BCDB-1F4B87CCCB9C

https://ccw.naturalis.nl/detail.php?id=9412

#### Materials

- **Type status:** Other material. **Occurrence:** catalogNumber: 525894; occurrenceRemarks: 1 male; recordedBy: K.M. Olsen | L.E. Høitomt; individualCount: 1; sex: male; preparations: Ethanol; occurrenceID: EU_LIM_306; **Taxon:** scientificName: Elephantomyia (Elephantomyia) krivosheinae Savchenko, 1976; family: Limoniidae; genus: Elephantomyia; subgenus: Elephantomyia; specificEpithet: krivosheinae; scientificNameAuthorship: Savchenko, 1976; **Location:** country: Norway; stateProvince: Akershus; municipality: Asker; locality: Poverudelva – Kantsone langs nordsiden; verbatimElevation: 184 m; minimumElevationInMeters: 184; decimalLatitude: 59.80926; decimalLongitude: 10.3632; **Identification:** identifiedBy: K.M. Olsen; **Event:** samplingProtocol: Sweep net; eventDate: 2017-06-15; verbatimEventDate: Jun-15-2017; **Record Level:** institutionCode: NHMO; basisOfRecord: PreservedSpecimen
- **Type status:** Other material. **Occurrence:** catalogNumber: 605516; occurrenceRemarks: 3 males; recordedBy: K.M. Olsen; individualCount: 3; sex: male; preparations: Ethanol; occurrenceID: EU_LIM_307; **Taxon:** scientificName: Elephantomyia (Elephantomyia) krivosheinae Savchenko, 1976; family: Limoniidae; genus: Elephantomyia; subgenus: Elephantomyia; specificEpithet: krivosheinae; scientificNameAuthorship: Savchenko, 1976; **Location:** country: Norway; stateProvince: Hedmark; municipality: Åmot; locality: Deifjell-lia; verbatimElevation: 515 m; minimumElevationInMeters: 515; decimalLatitude: 61.28427; decimalLongitude: 11.50497; **Identification:** identifiedBy: K.M. Olsen; **Event:** samplingProtocol: Malaise trap; eventDate: 2018-05-05/2018-07-06; verbatimEventDate: 05/May-06/Jul/2018; **Record Level:** institutionCode: NHMO; basisOfRecord: PreservedSpecimen
- **Type status:** Other material. **Occurrence:** catalogNumber: 549211; occurrenceRemarks: 1 male; recordedBy: K.M. Olsen; individualCount: 1; sex: male; preparations: Ethanol; occurrenceID: EU_LIM_308; **Taxon:** scientificName: Elephantomyia (Elephantomyia) krivosheinae Savchenko, 1976; family: Limoniidae; genus: Elephantomyia; subgenus: Elephantomyia; specificEpithet: krivosheinae; scientificNameAuthorship: Savchenko, 1976; **Location:** country: Norway; stateProvince: Sogn og Fjordane; municipality: Luster; locality: Mørkrisdalen – Hyrnavollen/Hødnevollen; verbatimElevation: 130 m; minimumElevationInMeters: 130; decimalLatitude: 61.55032; decimalLongitude: 7.62649; **Identification:** identifiedBy: K.M. Olsen; **Event:** samplingProtocol: Sweep net; eventDate: 2017-06-25; verbatimEventDate: 25/Jun/2017; **Record Level:** institutionCode: PCKMO; basisOfRecord: PreservedSpecimen
- **Type status:** Other material. **Occurrence:** catalogNumber: 524825; occurrenceRemarks: 1 female; recordedBy: K.M. Olsen | O.M. Wergeland Krog; individualCount: 1; sex: female; preparations: Ethanol; occurrenceID: EU_LIM_309; **Taxon:** scientificName: Elephantomyia (Elephantomyia) krivosheinae Savchenko, 1976; family: Limoniidae; genus: Elephantomyia; subgenus: Elephantomyia; specificEpithet: krivosheinae; scientificNameAuthorship: Savchenko, 1976; **Location:** country: Norway; stateProvince: Telemark; municipality: Porsgrunn; locality: Solbakkmoen; verbatimElevation: 40 m; minimumElevationInMeters: 40; decimalLatitude: 59.09547; decimalLongitude: 9.72142; **Identification:** identifiedBy: K.M. Olsen; **Event:** samplingProtocol: Sweep net; eventDate: 2017-05-29; verbatimEventDate: 29/May/2017; **Record Level:** institutionCode: NHMO; basisOfRecord: PreservedSpecimen
- **Type status:** Other material. **Occurrence:** catalogNumber: 533501; occurrenceRemarks: 1 female; recordedBy: K.M. Olsen; individualCount: 1; sex: female; preparations: Ethanol; occurrenceID: EU_LIM_310; **Taxon:** scientificName: Elephantomyia (Elephantomyia) krivosheinae Savchenko, 1976; family: Limoniidae; genus: Elephantomyia; subgenus: Elephantomyia; specificEpithet: krivosheinae; scientificNameAuthorship: Savchenko, 1976; **Location:** country: Norway; stateProvince: Troms; municipality: Målselv; locality: Frihetsli–Anaskåla; verbatimElevation: 215-585 m; minimumElevationInMeters: 215; maximumElevationInMeters: 585; decimalLatitude: 68.7739; decimalLongitude: 19.72745; **Identification:** identifiedBy: K.M. Olsen; **Event:** samplingProtocol: Sweep net; eventDate: 2017-06-04; verbatimEventDate: 04/Jul/2017; **Record Level:** institutionCode: NHMO; basisOfRecord: PreservedSpecimen
- **Type status:** Other material. **Occurrence:** catalogNumber: 524773; occurrenceRemarks: 3 male+female; recordedBy: K.M. Olsen | S. Olberg; individualCount: 3; sex: male, female; preparations: Ethanol; occurrenceID: EU_LIM_311; **Taxon:** scientificName: Elephantomyia (Elephantomyia) krivosheinae Savchenko, 1976; family: Limoniidae; genus: Elephantomyia; subgenus: Elephantomyia; specificEpithet: krivosheinae; scientificNameAuthorship: Savchenko, 1976; **Location:** country: Norway; stateProvince: Vestfold; municipality: Horten; locality: WSW Solberg; verbatimElevation: 70 m; minimumElevationInMeters: 70; decimalLatitude: 59.40173; decimalLongitude: 10.39881; **Identification:** identifiedBy: K.M. Olsen; **Event:** samplingProtocol: Hand picking; eventDate: 2017-06-01; verbatimEventDate: 01/Jun/2017; **Record Level:** institutionCode: PCKMO; basisOfRecord: PreservedSpecimen

#### Distribution

First records from Norway.

### Elliptera hungarica

Madarassy, 1881

0215B74C-ADC5-510B-8DE3-47CBC5C2BFBE

https://ccw.naturalis.nl/detail.php?id=9509

#### Materials

- **Type status:** Other material. **Occurrence:** occurrenceRemarks: 1 male; recordedBy: J. Starý; individualCount: 1; sex: male; preparations: Pinned; occurrenceID: EU_LIM_312; **Taxon:** scientificName: Ellipterahungarica Madarassy, 1881; family: Limoniidae; genus: Elliptera; specificEpithet: hungarica; scientificNameAuthorship: Madarassy, 1881; **Location:** country: Greece; stateProvince: Pieria; municipality: Prionia; locality: Olympos Mts.; verbatimElevation: 1000-1200 m; minimumElevationInMeters: 1000; maximumElevationInMeters: 1200; decimalLatitude: 40.079; decimalLongitude: 22.393; **Identification:** identifiedBy: J. Starý; **Event:** eventDate: 2007-06-01; verbatimEventDate: 1/Jun/2007; **Record Level:** institutionCode: PCJS; basisOfRecord: PreservedSpecimen
- **Type status:** Other material. **Occurrence:** occurrenceRemarks: 7 males, 3 females; recordedBy: J. Starý; individualCount: 10; sex: male, female; preparations: Pinned; occurrenceID: EU_LIM_313; **Taxon:** scientificName: Ellipterahungarica Madarassy, 1881; family: Limoniidae; genus: Elliptera; specificEpithet: hungarica; scientificNameAuthorship: Madarassy, 1881; **Location:** country: Greece; stateProvince: Pieria; municipality: Prionia; locality: Olympos Mts.; verbatimElevation: 1000-1200 m; minimumElevationInMeters: 1000; maximumElevationInMeters: 1200; decimalLatitude: 40.079; decimalLongitude: 22.393; **Identification:** identifiedBy: J. Starý; **Event:** eventDate: 2007-06-06; verbatimEventDate: 6/Jun/2007; **Record Level:** institutionCode: PCJS; basisOfRecord: PreservedSpecimen
- **Type status:** Other material. **Occurrence:** occurrenceRemarks: 2 males; recordedBy: L.-P. Kolcsár; individualCount: 2; sex: male; preparations: Ethanol; occurrenceID: EU_LIM_314; **Taxon:** scientificName: Ellipterahungarica Madarassy, 1881; family: Limoniidae; genus: Elliptera; specificEpithet: hungarica; scientificNameAuthorship: Madarassy, 1881; **Location:** country: Montenegro; municipality: Cetinje; verbatimElevation: 670 m; minimumElevationInMeters: 670; decimalLatitude: 42.38725; decimalLongitude: 18.93299; **Identification:** identifiedBy: L.-P. Kolcsár; **Event:** samplingProtocol: Sweep net; eventDate: 2014-04-30; verbatimEventDate: 30/Apr/2014; **Record Level:** institutionCode: CKLP; basisOfRecord: PreservedSpecimen

#### Distribution

First records from Greece (from mainland) and Montenegro.

### Ellipteroides (Protogonomyia) alboscutellatus

(von Roser, 1840)

BD684809-87C0-5E85-B730-48B67E5E2913

https://ccw.naturalis.nl/detail.php?id=864

#### Materials

- **Type status:** Other material. **Occurrence:** catalogNumber: JES-20120225; occurrenceRemarks: 1 male; recordedBy: J. Ilmonen; individualCount: 1; sex: male; preparations: Ethanol; occurrenceID: EU_LIM_315; **Taxon:** scientificName: Ellipteroides (Protogonomyia) alboscutellatus (von Roser, 1840); family: Limoniidae; genus: Ellipteroides; subgenus: Protogonomyia; specificEpithet: alboscutellatus; scientificNameAuthorship: (von Roser, 1840); **Location:** island: Saaremaa; country: Estonia; municipality: Viieristi; verbatimElevation: 30 m; minimumElevationInMeters: 30; decimalLatitude: 58.017; decimalLongitude: 22.184; **Identification:** identifiedBy: J. Salmela; **Event:** samplingProtocol: Sweep net; eventDate: 2010-06-09; verbatimEventDate: 09/Jun/2010; **Record Level:** institutionCode: LMM; basisOfRecord: PreservedSpecimen

#### Distribution

First record from Estonia.

### Ellipteroides (Protogonomyia) limbatus

(von Roser, 1840)

C714BED4-3C8B-50C0-8592-F2BFB38D05C9

https://ccw.naturalis.nl/detail.php?id=886

#### Materials

- **Type status:** Other material. **Occurrence:** occurrenceRemarks: 1 female; recordedBy: M.C. de Haas; individualCount: 1; sex: female; preparations: Ethanol; occurrenceID: EU_LIM_861; **Taxon:** scientificName: Ellipteroides (Protogonomyia) limbatus (von Roser, 1840); family: Limoniidae; genus: Ellipteroides; subgenus: Protogonomyia; specificEpithet: limbatus; scientificNameAuthorship: (von Roser, 1840); **Location:** country: Slovenia; municipality: Ljubno; locality: In forest near Savina; verbatimElevation: 490 m; minimumElevationInMeters: 490; decimalLatitude: 46.332; decimalLongitude: 14.839; **Identification:** identifiedBy: M.C. d'Oliveira; **Event:** samplingProtocol: Light trap; eventDate: 2020-9-8; verbatimEventDate: 09/august/2020; habitat: Edge of forest with small stream; **Record Level:** institutionCode: PCMCO; basisOfRecord: PreservedSpecimen

#### Distribution

First record from Slovenia.

### Eloeophila apicata

(Loew, 1871)

166ABA61-4F01-59AF-B901-A885D9E4E3C7

https://ccw.naturalis.nl/detail.php?id=5201

#### Materials

- **Type status:** Other material. **Occurrence:** catalogNumber: vial No8, vial No14; occurrenceRemarks: 4 males, 8 females; recordedBy: V. Tichonov; individualCount: 12; sex: male, female; preparations: Ethanol; occurrenceID: EU_LIM_316; **Taxon:** scientificName: Eloeophilaapicata (Loew, 1871); family: Limoniidae; genus: Eloeophila; specificEpithet: apicata; scientificNameAuthorship: (Loew, 1871); **Location:** country: Russia; stateProvince: North Caucasus; county: Republic of Dagestan; municipality: Magaramkent; locality: between villages Magaramkent and Levashi; verbatimElevation: 1000 m; minimumElevationInMeters: 1000; decimalLatitude: 41.46; decimalLongitude: 47.55; **Identification:** identifiedBy: V.I. Lantsov; **Event:** samplingProtocol: Sweep net; eventDate: 2004-05-03; verbatimEventDate: 03/May/2004; habitat: mountain steppe; **Record Level:** institutionCode: ZIN; basisOfRecord: PreservedSpecimen

#### Distribution

Presence of the species in Russia: NC mentioned in Lantsov (2020) without further details. Here, we publish the collection data for that record.

### Eloeophila maculata

(Meigen, 1804)

204E206C-1BF6-50B8-98E5-A5D1BDDDE413

https://ccw.naturalis.nl/detail.php?id=5232

#### Materials

- **Type status:** Other material. **Occurrence:** occurrenceRemarks: 2 males; recordedBy: L.-P. Kolcsár; individualCount: 2; sex: male; preparations: Ethanol; occurrenceID: EU_LIM_317; **Taxon:** scientificName: Eloeophilamaculata (Meigen, 1804); family: Limoniidae; genus: Eloeophila; specificEpithet: maculata; scientificNameAuthorship: (Meigen, 1804); **Location:** country: North Macedonia; municipality: Bratin Dol; locality: Pelister Mts.; verbatimElevation: 875 m; minimumElevationInMeters: 875; decimalLatitude: 41.06944; decimalLongitude: 21.23435; **Identification:** identifiedBy: L.-P. Kolcsár; **Event:** samplingProtocol: Sweep net; eventDate: 2012-05-04; verbatimEventDate: 4/May/2012; habitat: brook; **Record Level:** institutionCode: CKLP; basisOfRecord: PreservedSpecimen
- **Type status:** Other material. **Occurrence:** occurrenceRemarks: 1 male; recordedBy: L.-P. Kolcsár | E. Török; individualCount: 1; sex: male; preparations: Ethanol; occurrenceID: EU_LIM_318; **Taxon:** scientificName: Eloeophilamaculata (Meigen, 1804); family: Limoniidae; genus: Eloeophila; specificEpithet: maculata; scientificNameAuthorship: (Meigen, 1804); **Location:** country: North Macedonia; municipality: Izvor; locality: Treska River; verbatimElevation: 755 m; minimumElevationInMeters: 755; decimalLatitude: 41.48021; decimalLongitude: 20.83466; **Identification:** identifiedBy: L.-P. Kolcsár; **Event:** samplingProtocol: Sweep net; eventDate: 2017-06-30; verbatimEventDate: 30/Jun/2017; **Record Level:** institutionCode: CKLP; basisOfRecord: PreservedSpecimen

#### Distribution

First records from North Macedonia.

### Eloeophila minor

Starý, 2009

1FB68DA3-9B76-523C-AE6C-49C63C238AAD

https://ccw.naturalis.nl/detail.php?id=17523

#### Materials

- **Type status:** Other material. **Occurrence:** occurrenceRemarks: 1 female; recordedBy: J. Starý; individualCount: 1; sex: female; preparations: Pinned; occurrenceID: EU_LIM_319; **Taxon:** scientificName: Eloeophilaminor Starý, 2009; family: Limoniidae; genus: Eloeophila; specificEpithet: minor; scientificNameAuthorship: Starý, 2009; **Location:** country: Bulgaria; stateProvince: Blagoevgrad; municipality: Lilianovo nr. Sandanski; decimalLatitude: 41.617; decimalLongitude: 23.321; **Identification:** identifiedBy: J. Starý; **Event:** eventDate: 1989-05-09; verbatimEventDate: 9/May/1989; **Record Level:** institutionCode: PCJS; basisOfRecord: PreservedSpecimen
- **Type status:** Other material. **Occurrence:** occurrenceRemarks: 2 males; recordedBy: J. Starý; individualCount: 2; sex: male; preparations: Pinned; occurrenceID: EU_LIM_320; **Taxon:** scientificName: Eloeophilaminor Starý, 2009; family: Limoniidae; genus: Eloeophila; specificEpithet: minor; scientificNameAuthorship: Starý, 2009; **Location:** country: Greece; stateProvince: Larisa; municipality: Karia; locality: 7 km E; verbatimElevation: 830 m; minimumElevationInMeters: 830; decimalLatitude: 39.998; decimalLongitude: 22.451; **Identification:** identifiedBy: J. Starý; **Event:** eventDate: 2007-06-03; verbatimEventDate: 3/Jun/2007; **Record Level:** institutionCode: PCJS; basisOfRecord: PreservedSpecimen
- **Type status:** Other material. **Occurrence:** occurrenceRemarks: 3 males, 2 females; recordedBy: J. Starý; individualCount: 5; sex: male, female; preparations: Pinned; occurrenceID: EU_LIM_321; **Taxon:** scientificName: Eloeophilaminor Starý, 2009; family: Limoniidae; genus: Eloeophila; specificEpithet: minor; scientificNameAuthorship: Starý, 2009; **Location:** country: Greece; stateProvince: Peloponnese; municipality: Ano Vlasia; locality: 2 km S; verbatimElevation: 1020 m; minimumElevationInMeters: 1020; decimalLatitude: 37.983; decimalLongitude: 21.9; **Identification:** identifiedBy: J. Starý; **Event:** eventDate: 2015-05-29; verbatimEventDate: 29/May/2015; habitat: brooks and springs; **Record Level:** institutionCode: PCJS; basisOfRecord: PreservedSpecimen
- **Type status:** Other material. **Occurrence:** occurrenceRemarks: 2 males; recordedBy: L. Papp; individualCount: 2; sex: male; preparations: Pinned; occurrenceID: EU_LIM_322; **Taxon:** scientificName: Eloeophilaminor Starý, 2009; family: Limoniidae; genus: Eloeophila; specificEpithet: minor; scientificNameAuthorship: Starý, 2009; **Location:** country: Hungary; stateProvince: Nógrád; municipality: Diósjenő; locality: Kemence Stream; decimalLatitude: 47.95245; decimalLongitude: 19.0019; **Identification:** identifiedBy: L.-P. Kolcsár; **Event:** eventDate: 2001-06-09; verbatimEventDate: 09/Jun/2001; **Record Level:** institutionCode: HNHM; basisOfRecord: PreservedSpecimen

#### Distribution

First records from Bulgaria, Greece (from mainland) and Hungary.

### Eloeophila mundata

(Loew, 1871)

015C6FA3-DB3C-55E5-8819-19DDAAE1B742

https://ccw.naturalis.nl/detail.php?id=5242

#### Materials

- **Type status:** Other material. **Occurrence:** occurrenceRemarks: 1 male; recordedBy: P. Juhász | T. Kovács | D. Murányi; individualCount: 1; sex: male; preparations: Ethanol; occurrenceID: EU_LIM_323; **Taxon:** scientificName: Eloeophilamundata (Loew, 1871); family: Limoniidae; genus: Eloeophila; specificEpithet: mundata; scientificNameAuthorship: (Loew, 1871); **Location:** country: Serbia; stateProvince: Moravica; municipality: Ivanjica; locality: Golija Mts.,; verbatimElevation: 1500 m; minimumElevationInMeters: 1500; decimalLatitude: 43.3381; decimalLongitude: 20.2509; **Identification:** identifiedBy: L.-P. Kolcsár; **Event:** samplingProtocol: Sweep net; eventDate: 2018-06-26; verbatimEventDate: 26/Jun/2018; habitat: forest stream and its sidebrook along road No. 197; **Record Level:** institutionCode: CKLP; basisOfRecord: PreservedSpecimen
- **Type status:** Other material. **Occurrence:** occurrenceRemarks: 3 males; recordedBy: L.-P. Kolcsár | E. Török; individualCount: 3; sex: male; preparations: Ethanol; occurrenceID: EU_LIM_324; **Taxon:** scientificName: Eloeophilamundata (Loew, 1871); family: Limoniidae; genus: Eloeophila; specificEpithet: mundata; scientificNameAuthorship: (Loew, 1871); **Location:** country: Serbia; municipality: Kopaonik; locality: Kopaonik Mts.; verbatimElevation: 1556 m; minimumElevationInMeters: 1556; decimalLatitude: 43.3099; decimalLongitude: 20.7656; **Identification:** identifiedBy: L.-P. Kolcsár; **Event:** samplingProtocol: Sweep net; eventDate: 2017-06-22; verbatimEventDate: 22/Jun/2017; **Record Level:** institutionCode: CKLP; basisOfRecord: PreservedSpecimen

#### Distribution

First record from Serbia.

### Eloeophila pectinistylus

Starý, 2009

9389DEBE-F8CB-594F-B204-5B6DDFD17982

https://ccw.naturalis.nl/detail.php?id=17524

#### Materials

- **Type status:** Other material. **Occurrence:** occurrenceRemarks: 5 males; recordedBy: E. Eiroa; individualCount: 5; sex: male; preparations: Pinned; occurrenceID: EU_LIM_325; **Taxon:** scientificName: Eloeophilapectinistylus Starý, 2009; family: Limoniidae; genus: Eloeophila; specificEpithet: pectinistylus; scientificNameAuthorship: Starý, 2009; **Location:** country: Portugal; stateProvince: Guarda; municipality: Manteigas; locality: Caldas de Manteigas, serra da Estrela; verbatimElevation: 775 m; minimumElevationInMeters: 775; decimalLatitude: 40.37863; decimalLongitude: -7.5488; **Identification:** identifiedBy: E. Eiroa; **Event:** samplingProtocol: Sweep net; eventDate: 1992-05-30; verbatimEventDate: 30/May/1992; **Record Level:** institutionCode: USC; basisOfRecord: PreservedSpecimen

#### Distribution

First record from Portugal.

### Eloeophila sparsipunctum

Starý, 2009

9E426A15-9B25-50E5-9035-BC15E0DCDFCA

https://ccw.naturalis.nl/detail.php?id=17526

#### Materials

- **Type status:** Other material. **Occurrence:** occurrenceRemarks: 1 male; recordedBy: L.-P. Kolcsár; individualCount: 1; sex: male; preparations: Ethanol; occurrenceID: EU_LIM_326; **Taxon:** scientificName: Eloeophilasparsipunctum Starý, 2009; family: Limoniidae; genus: Eloeophila; specificEpithet: sparsipunctum; scientificNameAuthorship: Starý, 2009; **Location:** country: Romania; stateProvince: Cluj; municipality: Măguri-Răcătău; locality: Gilău Mts., Someșul Rece River; verbatimElevation: 582 m; minimumElevationInMeters: 582; decimalLatitude: 46.66561; decimalLongitude: 23.24234; **Identification:** identifiedBy: L.-P. Kolcsár; **Event:** samplingProtocol: Sweep net; eventDate: 2017-08-06; verbatimEventDate: 06/Aug/2017; **Record Level:** institutionCode: CKLP; basisOfRecord: PreservedSpecimen

#### Description

Figs 5, 6

#### Distribution

First record from Romania.

### Eloeophila submarmorata

(Verrall, 1887)

32DA1F5A-5BD0-50DC-BEDD-6094A095DEFB

https://ccw.naturalis.nl/detail.php?id=5268

#### Materials

- **Type status:** Other material. **Occurrence:** occurrenceRemarks: 1 male; recordedBy: L. Papp; individualCount: 1; sex: male; preparations: Pinned; occurrenceID: EU_LIM_327; **Taxon:** scientificName: Eloeophilasubmarmorata (Verrall, 1887); family: Limoniidae; genus: Eloeophila; specificEpithet: submarmorata; scientificNameAuthorship: (Verrall, 1887); **Location:** country: Hungary; stateProvince: Baranya; municipality: Pécs; locality: Égervölgyi Protected Area; decimalLatitude: 46.0889; decimalLongitude: 18.18; **Identification:** identifiedBy: L.-P. Kolcsár; **Event:** eventDate: 2006-05-22; verbatimEventDate: 22/May/2006; **Record Level:** institutionCode: HNHM; basisOfRecord: PreservedSpecimen
- **Type status:** Other material. **Occurrence:** occurrenceRemarks: 1 male; recordedBy: L. Papp; individualCount: 1; sex: male; preparations: Pinned; occurrenceID: EU_LIM_328; **Taxon:** scientificName: Eloeophilasubmarmorata (Verrall, 1887); family: Limoniidae; genus: Eloeophila; specificEpithet: submarmorata; scientificNameAuthorship: (Verrall, 1887); **Location:** country: Hungary; stateProvince: Pest; municipality: Szokolya; locality: upper reach of Szén Stream; decimalLatitude: 47.9048; decimalLongitude: 18.9808; **Identification:** identifiedBy: L.-P. Kolcsár; **Event:** eventDate: 2001-05-05; verbatimEventDate: 05/May/2001; **Record Level:** institutionCode: HNHM; basisOfRecord: PreservedSpecimen
- **Type status:** Other material. **Occurrence:** occurrenceRemarks: 4 males; recordedBy: V.E. Pilipenko; individualCount: 4; sex: male; occurrenceID: EU_LIM_329; **Taxon:** scientificName: Eloeophilasubmarmorata (Verrall, 1887); family: Limoniidae; genus: Eloeophila; specificEpithet: submarmorata; scientificNameAuthorship: (Verrall, 1887); **Location:** country: Russia; stateProvince: Central European Russia; county: Moskovskaya Oblast; municipality: Solnechnogorsk district; locality: Chashnikovo; verbatimElevation: 220 m; minimumElevationInMeters: 220; decimalLatitude: 56.0375; decimalLongitude: 37.1874; **Identification:** identifiedBy: V.E. Pilipenko; **Event:** samplingProtocol: Sweep net; eventDate: 1992-06-14; verbatimEventDate: 14/Jun/1992; **Record Level:** institutionCode: VPMC; basisOfRecord: PreservedSpecimen
- **Type status:** Other material. **Occurrence:** occurrenceRemarks: 1 male, 2 females; recordedBy: V.E. Pilipenko; individualCount: 3; sex: male, female; occurrenceID: EU_LIM_330; **Taxon:** scientificName: Eloeophilasubmarmorata (Verrall, 1887); family: Limoniidae; genus: Eloeophila; specificEpithet: submarmorata; scientificNameAuthorship: (Verrall, 1887); **Location:** country: Russia; stateProvince: Central European Russia; county: Moskovskaya Oblast; municipality: Solnechnogorsk district; locality: Chashnikovo; verbatimElevation: 220 m; minimumElevationInMeters: 220; decimalLatitude: 56.0375; decimalLongitude: 37.1874; **Identification:** identifiedBy: V.E. Pilipenko; **Event:** samplingProtocol: Sweep net; eventDate: 1997-06-03; verbatimEventDate: 03/Jun/1997; **Record Level:** institutionCode: VPMC; basisOfRecord: PreservedSpecimen
- **Type status:** Other material. **Occurrence:** occurrenceRemarks: 1 male, 1 female; recordedBy: V.E. Pilipenko; individualCount: 2; sex: male, female; occurrenceID: EU_LIM_331; **Taxon:** scientificName: Eloeophilasubmarmorata (Verrall, 1887); family: Limoniidae; genus: Eloeophila; specificEpithet: submarmorata; scientificNameAuthorship: (Verrall, 1887); **Location:** country: Russia; stateProvince: Central European Russia; county: Moskovskaya Oblast; municipality: Solnechnogorsk district; locality: Chashnikovo; verbatimElevation: 220 m; minimumElevationInMeters: 220; decimalLatitude: 56.0375; decimalLongitude: 37.1874; **Identification:** identifiedBy: V.E. Pilipenko; **Event:** samplingProtocol: Sweep net; eventDate: 1997-06-03; verbatimEventDate: 03/Jul/1997; **Record Level:** institutionCode: VPMC; basisOfRecord: PreservedSpecimen
- **Type status:** Other material. **Occurrence:** occurrenceRemarks: 1 female; recordedBy: V.E. Pilipenko; individualCount: 1; sex: female; occurrenceID: EU_LIM_332; **Taxon:** scientificName: Eloeophilasubmarmorata (Verrall, 1887); family: Limoniidae; genus: Eloeophila; specificEpithet: submarmorata; scientificNameAuthorship: (Verrall, 1887); **Location:** country: Russia; stateProvince: Central European Russia; county: Moskovskaya Oblast; municipality: Solnechnogorsk district; locality: Chashnikovo; verbatimElevation: 220 m; minimumElevationInMeters: 220; decimalLatitude: 56.0375; decimalLongitude: 37.1874; **Identification:** identifiedBy: V.E. Pilipenko; **Event:** samplingProtocol: Sweep net; eventDate: 1996-05-14; verbatimEventDate: 14/May/1996; **Record Level:** institutionCode: VPMC; basisOfRecord: PreservedSpecimen
- **Type status:** Other material. **Occurrence:** occurrenceRemarks: 4 males, 2 females; recordedBy: L.-P. Kolcsár | E. Török; individualCount: 6; sex: male, female; preparations: Ethanol; occurrenceID: EU_LIM_333; **Taxon:** scientificName: Eloeophilasubmarmorata (Verrall, 1887); family: Limoniidae; genus: Eloeophila; specificEpithet: submarmorata; scientificNameAuthorship: (Verrall, 1887); **Location:** country: Serbia; municipality: Kopaonik; locality: Kopaonik Mts.; verbatimElevation: 1600 m; minimumElevationInMeters: 1600; decimalLatitude: 43.2981; decimalLongitude: 20.78706; **Identification:** identifiedBy: L.-P. Kolcsár; **Event:** samplingProtocol: Sweep net; eventDate: 2017-06-22; verbatimEventDate: 22/Jun/2017; **Record Level:** institutionCode: CKLP; basisOfRecord: PreservedSpecimen

#### Distribution

First records from Hungary, Russia: RUC and Serbia.

### Eloeophila trimaculata

(Zetterstedt, 1838)

ACC49D8C-0FFA-5FEB-99A9-3B691892823C

https://ccw.naturalis.nl/detail.php?id=5272

#### Materials

- **Type status:** Other material. **Occurrence:** occurrenceRemarks: 1 male; recordedBy: L. Papp; individualCount: 1; sex: male; preparations: Pinned; occurrenceID: EU_LIM_334; **Taxon:** scientificName: Eloeophilatrimaculata (Zetterstedt, 1838); family: Limoniidae; genus: Eloeophila; specificEpithet: trimaculata; scientificNameAuthorship: (Zetterstedt, 1838); **Location:** country: Hungary; stateProvince: Nógrád; municipality: Diósjenő; locality: Kemence Stream; decimalLatitude: 47.95245; decimalLongitude: 19.0019; **Identification:** identifiedBy: L.-P. Kolcsár; **Event:** eventDate: 2006-05-03; verbatimEventDate: 03/May/2006; **Record Level:** institutionCode: HNHM; basisOfRecord: PreservedSpecimen

#### Distribution

First record from Hungary.

### Eloeophila verralli

(Bergroth, 1912)

3075AD20-5577-5EC8-AE98-4808B3A9FBF1

https://ccw.naturalis.nl/detail.php?id=5278

#### Materials

- **Type status:** Other material. **Occurrence:** occurrenceRemarks: 1 male; recordedBy: L. Papp; individualCount: 1; sex: male; preparations: Pinned; occurrenceID: EU_LIM_335; **Taxon:** scientificName: Eloeophilaverralli (Bergroth, 1912); family: Limoniidae; genus: Eloeophila; specificEpithet: verralli; scientificNameAuthorship: (Bergroth, 1912); **Location:** country: Hungary; stateProvince: Baranya; municipality: Pécs; locality: Melegmány Protected Area, Melegmány Valley; decimalLatitude: 46.11808; decimalLongitude: 18.21515; **Identification:** identifiedBy: L.-P. Kolcsár; **Event:** eventDate: 2000-06-15; verbatimEventDate: 15/Jun/2000; **Record Level:** institutionCode: HNHM; basisOfRecord: PreservedSpecimen
- **Type status:** Other material. **Occurrence:** occurrenceRemarks: 2 males; recordedBy: L.-P. Kolcsár; individualCount: 2; sex: male; preparations: Ethanol; occurrenceID: EU_LIM_336; **Taxon:** scientificName: Eloeophilaverralli (Bergroth, 1912); family: Limoniidae; genus: Eloeophila; specificEpithet: verralli; scientificNameAuthorship: (Bergroth, 1912); **Location:** country: Serbia; county: Pirot; municipality: Temska; locality: Cerovo; verbatimElevation: 450 m; minimumElevationInMeters: 450; decimalLatitude: 43.2886; decimalLongitude: 22.5168; **Identification:** identifiedBy: L.-P. Kolcsár; **Event:** samplingProtocol: Sweep net; eventDate: 2012-05-07; verbatimEventDate: 07/May/2012; **Record Level:** institutionCode: CKLP; basisOfRecord: PreservedSpecimen

#### Distribution

First records from Hungary and Serbia.

### Epiphragma (Epiphragma) ocellare

(Linnaeus, 1760)

FF2F92CB-B0B8-5178-92D6-DE7FF62985FC

https://ccw.naturalis.nl/detail.php?id=5363

#### Materials

- **Type status:** Other material. **Occurrence:** occurrenceRemarks: 1 male; recordedBy: L.-P. Kolcsár; individualCount: 1; sex: male; preparations: Ethanol; occurrenceID: EU_LIM_337; **Taxon:** scientificName: Epiphragma (Epiphragma) ocellare (Linnaeus, 1760); family: Limoniidae; genus: Epiphragma; subgenus: Epiphragma; specificEpithet: ocellare; scientificNameAuthorship: (Linnaeus, 1760); **Location:** country: Greece; stateProvince: Eastern Macedonia and Thrace; municipality: Chrysoupoli; locality: Nea Karya, Nestos River and Delta; verbatimElevation: 3 m; minimumElevationInMeters: 3; decimalLatitude: 40.87841; decimalLongitude: 24.78541; **Identification:** identifiedBy: L.-P. Kolcsár; **Event:** samplingProtocol: Sweep net; eventDate: 2011-05-26; verbatimEventDate: 26/May/2011; **Record Level:** institutionCode: CKLP; basisOfRecord: PreservedSpecimen

#### Distribution

First record from Greece (from mainland).

### Erioconopa diuturna

(Walker, 1848)

EA615EAD-CF76-5E20-A433-3743C5F79197

https://ccw.naturalis.nl/detail.php?id=925

#### Materials

- **Type status:** Other material. **Occurrence:** occurrenceRemarks: 1 female; recordedBy: J. Roháček; individualCount: 1; sex: female; preparations: Pinned; occurrenceID: EU_LIM_338; **Taxon:** scientificName: Erioconopadiuturna (Walker, 1848); family: Limoniidae; genus: Erioconopa; specificEpithet: diuturna; scientificNameAuthorship: (Walker, 1848); **Location:** island: Sardinia; country: Italy; stateProvince: Sardinia; municipality: Monti; locality: 8.1 km S, Rio de s´Éleme, road bridge; verbatimElevation: 465 m; minimumElevationInMeters: 465; decimalLatitude: 40.7333; decimalLongitude: 9.366; **Identification:** identifiedBy: J. Starý; **Event:** eventDate: 2014-05-07; verbatimEventDate: May-07-2014; habitat: riverside vegetation; **Record Level:** institutionCode: PCJS; basisOfRecord: PreservedSpecimen
- **Type status:** Other material. **Occurrence:** occurrenceRemarks: 6 males, 1 female; recordedBy: J. Starý; individualCount: 7; sex: male, female; preparations: Pinned; occurrenceID: EU_LIM_339; **Taxon:** scientificName: Erioconopadiuturna (Walker, 1848); family: Limoniidae; genus: Erioconopa; specificEpithet: diuturna; scientificNameAuthorship: (Walker, 1848); **Location:** island: Sicily; country: Italy; stateProvince: Sicily; municipality: Tortorici; locality: Parco dei Nebrodi, Lago Pisciotto env.; verbatimElevation: 1235 m; minimumElevationInMeters: 1235; decimalLatitude: 37.97611; decimalLongitude: 14.85167; **Identification:** identifiedBy: J. Starý; **Event:** eventDate: 2016-04-21; verbatimEventDate: 21/Apr/2016; habitat: brook, wetlands; **Record Level:** institutionCode: PCJS; basisOfRecord: PreservedSpecimen
- **Type status:** Other material. **Occurrence:** occurrenceRemarks: 3 males; recordedBy: J. Starý; individualCount: 3; sex: male; preparations: Pinned; occurrenceID: EU_LIM_340; **Taxon:** scientificName: Erioconopadiuturna (Walker, 1848); family: Limoniidae; genus: Erioconopa; specificEpithet: diuturna; scientificNameAuthorship: (Walker, 1848); **Location:** island: Sicily; country: Italy; stateProvince: Sicily; municipality: Floresta; locality: Parco dei Nebrodi, 2 km W; verbatimElevation: 1260 m; minimumElevationInMeters: 1260; decimalLatitude: 37.98778; decimalLongitude: 14.88722; **Identification:** identifiedBy: J. Starý; **Event:** eventDate: 2016-04-21; verbatimEventDate: 21/Apr/2016; habitat: wetlands; **Record Level:** institutionCode: PCJS; basisOfRecord: PreservedSpecimen
- **Type status:** Other material. **Occurrence:** occurrenceRemarks: 2 males; recordedBy: J. Starý; individualCount: 2; sex: male; preparations: Pinned; occurrenceID: EU_LIM_341; **Taxon:** scientificName: Erioconopadiuturna (Walker, 1848); family: Limoniidae; genus: Erioconopa; specificEpithet: diuturna; scientificNameAuthorship: (Walker, 1848); **Location:** island: Sicily; country: Italy; stateProvince: Sicily; municipality: Brolo; locality: 1.5 km S, Fiumara di Brolo; verbatimElevation: 620 m; minimumElevationInMeters: 620; decimalLatitude: 38.14417; decimalLongitude: 14.8225; **Identification:** identifiedBy: J. Starý; **Event:** eventDate: 2016-04-22; verbatimEventDate: 22/Apr/2016; **Record Level:** institutionCode: PCJS; basisOfRecord: PreservedSpecimen
- **Type status:** Other material. **Occurrence:** occurrenceRemarks: 3 males; recordedBy: A. Polevoi; individualCount: 3; sex: male; preparations: Pinned; occurrenceID: EU_LIM_342; **Taxon:** scientificName: Erioconopadiuturna (Walker, 1848); family: Limoniidae; genus: Erioconopa; specificEpithet: diuturna; scientificNameAuthorship: (Walker, 1848); **Location:** island: Kondostrov; country: Russia; stateProvince: North European Russia; county: Republic Karelia; municipality: Belomorsk; locality: Abakumicha; verbatimElevation: 10 m; minimumElevationInMeters: 10; decimalLatitude: 64.23462; decimalLongitude: 36.58452; **Identification:** identifiedBy: A. Polevoi; **Event:** samplingProtocol: Sweep net; eventDate: 2019-09-01; verbatimEventDate: 01/Sep/2019; **Record Level:** institutionCode: FRIP; basisOfRecord: PreservedSpecimen

#### Distribution

First records from Russia: RUN. The species previously reported from mainland Italy and here, we report first time from Sardinia and Sicily.

### Erioconopa symplectoides

(Kuntze, 1914)

851548EA-0480-5D33-A609-D7C7219BB1CB

https://ccw.naturalis.nl/detail.php?id=933

#### Materials

- **Type status:** Other material. **Occurrence:** occurrenceRemarks: 1 male, 1 female; recordedBy: D.I. Gavryushin; individualCount: 2; sex: male, female; preparations: Pinned; occurrenceID: EU_LIM_343; **Taxon:** scientificName: Erioconopasymplectoides (Kuntze, 1914); family: Limoniidae; genus: Erioconopa; specificEpithet: symplectoides; scientificNameAuthorship: (Kuntze, 1914); **Location:** country: Serbia; locality: Stara Planina Mts.; verbatimElevation: 1500 m; minimumElevationInMeters: 1500; decimalLatitude: 43.37; decimalLongitude: 22.6; **Identification:** identifiedBy: D.I. Gavryushin; **Event:** samplingProtocol: Sweep net; eventDate: 2015-05-01/2015-05-08; verbatimEventDate: 01-08/May/2015; **Record Level:** institutionCode: ZMMU; basisOfRecord: PreservedSpecimen

#### Distribution

First record from Serbia.

### Erioconopa trivialis

(Meigen, 1818)

0B8F4E2F-FD14-5039-9EE7-C97C4B33C5E2

https://ccw.naturalis.nl/detail.php?id=935

#### Materials

- **Type status:** Other material. **Occurrence:** occurrenceRemarks: 1 male; recordedBy: D.I. Gavryushin; individualCount: 1; sex: male; preparations: Ethanol; occurrenceID: EU_LIM_344; **Taxon:** scientificName: Erioconopatrivialis (Meigen, 1818); family: Limoniidae; genus: Erioconopa; specificEpithet: trivialis; scientificNameAuthorship: (Meigen, 1818); **Location:** country: Belarus; stateProvince: Vitebsk; county: Haradok; locality: Ezerische; decimalLatitude: 55.83; decimalLongitude: 30; **Identification:** identifiedBy: D.I. Gavryushin; **Event:** samplingProtocol: Sweep net; eventDate: 2019-05-16/2019-05-17; verbatimEventDate: 16-17/May/2019; **Record Level:** institutionCode: ZMMU; basisOfRecord: PreservedSpecimen
- **Type status:** Other material. **Occurrence:** occurrenceRemarks: 2 males, 1 female; recordedBy: D.I. Gavryushin; individualCount: 3; sex: male, female; preparations: Pinned; occurrenceID: EU_LIM_345; **Taxon:** scientificName: Erioconopatrivialis (Meigen, 1818); family: Limoniidae; genus: Erioconopa; specificEpithet: trivialis; scientificNameAuthorship: (Meigen, 1818); **Location:** country: Belarus; stateProvince: Vitebsk; county: Haradok; locality: Ezerische; decimalLatitude: 55.83; decimalLongitude: 30; **Identification:** identifiedBy: D.I. Gavryushin; **Event:** samplingProtocol: Sweep net; eventDate: 2019-05-16/2019-05-17; verbatimEventDate: 16-17/May/2019; **Record Level:** institutionCode: ZMMU; basisOfRecord: PreservedSpecimen
- **Type status:** Other material. **Occurrence:** occurrenceRemarks: 6 males; recordedBy: D.I. Gavryushin; individualCount: 6; sex: male; occurrenceID: EU_LIM_346; **Taxon:** scientificName: Erioconopatrivialis (Meigen, 1818); family: Limoniidae; genus: Erioconopa; specificEpithet: trivialis; scientificNameAuthorship: (Meigen, 1818); **Location:** country: Russia; stateProvince: East European Russia; county: Bashkortostan Respublika; municipality: Beloretsk district; locality: Abzakovo env., Kulsugady River; verbatimElevation: 531 m; minimumElevationInMeters: 531; decimalLatitude: 53.83795; decimalLongitude: 58.5823; **Identification:** identifiedBy: D.I. Gavryushin; **Event:** samplingProtocol: Sweep net; eventDate: 2012-08-15; verbatimEventDate: 15/Aug/2012; **Record Level:** institutionCode: ZMMU; basisOfRecord: PreservedSpecimen
- **Type status:** Other material. **Occurrence:** occurrenceRemarks: 1 female; recordedBy: D.I. Gavryushin; individualCount: 1; sex: female; occurrenceID: EU_LIM_347; **Taxon:** scientificName: Erioconopatrivialis (Meigen, 1818); family: Limoniidae; genus: Erioconopa; specificEpithet: trivialis; scientificNameAuthorship: (Meigen, 1818); **Location:** country: Russia; stateProvince: East European Russia; county: Bashkortostan Respublika; municipality: Beloretsk district; locality: Beloretsk env., Nura River; verbatimElevation: 494 m; minimumElevationInMeters: 494; decimalLatitude: 53.97365; decimalLongitude: 58.34415; **Identification:** identifiedBy: D.I. Gavryushin; **Event:** samplingProtocol: Sweep net; eventDate: 2012-08-10; verbatimEventDate: 10/Aug/2012; **Record Level:** institutionCode: ZMMU; basisOfRecord: PreservedSpecimen

#### Distribution

First records from Belarus and Russia: RUE.

### Erioptera (Erioptera) beckeri

Kuntze, 1914

87E220CC-26A2-56A5-A896-32C693A448FA

https://ccw.naturalis.nl/detail.php?id=964

#### Materials

- **Type status:** Other material. **Occurrence:** catalogNumber: 536180, 644173; occurrenceRemarks: 9 male+female; recordedBy: F. Midtgaard; individualCount: 9; sex: male, female; preparations: Ethanol; occurrenceID: EU_LIM_348; **Taxon:** scientificName: Erioptera (Erioptera) beckeri Kuntze, 1914; family: Limoniidae; genus: Erioptera; subgenus: Erioptera; specificEpithet: beckeri; scientificNameAuthorship: Kuntze, 1914; **Location:** country: Norway; stateProvince: Akershus; municipality: Bærum; locality: Ostøya; verbatimElevation: 20 m; minimumElevationInMeters: 20; decimalLatitude: 59.86854; decimalLongitude: 10.57335; **Identification:** identifiedBy: K.M. Olsen; **Event:** samplingProtocol: Malaise trap; eventDate: 1984-06-10/1984-07-01; verbatimEventDate: 10/Jun-01/Jul/1984; **Record Level:** institutionCode: PCKMO; basisOfRecord: PreservedSpecimen
- **Type status:** Other material. **Occurrence:** catalogNumber: 655922; occurrenceRemarks: 2 male+female; recordedBy: E. Rindal; individualCount: 2; sex: male, female; preparations: Ethanol; occurrenceID: EU_LIM_349; **Taxon:** scientificName: Erioptera (Erioptera) beckeri Kuntze, 1914; family: Limoniidae; genus: Erioptera; subgenus: Erioptera; specificEpithet: beckeri; scientificNameAuthorship: Kuntze, 1914; **Location:** country: Norway; stateProvince: Vestfold; municipality: Horten; locality: Adalstjernet SE; verbatimElevation: 71 m; minimumElevationInMeters: 71; decimalLatitude: 59.36956; decimalLongitude: 10.43836; **Identification:** identifiedBy: K.M. Olsen; **Event:** samplingProtocol: Malaise trap; eventDate: 2003-05-14/2003-07-08; verbatimEventDate: 14/May-08/Jul/2003; **Record Level:** institutionCode: PCKMO; basisOfRecord: PreservedSpecimen

#### Distribution

First records from Norway.

### Erioptera (Erioptera) divisa

(Walker, 1848)

B6A14361-89B8-5246-8D67-4DC8981CB582

https://ccw.naturalis.nl/detail.php?id=995

#### Materials

- **Type status:** Other material. **Occurrence:** occurrenceRemarks: 1 female; recordedBy: V.E. Pilipenko; individualCount: 1; sex: female; occurrenceID: EU_LIM_350; **Taxon:** scientificName: Erioptera (Erioptera) divisa (Walker, 1848); family: Limoniidae; genus: Erioptera; subgenus: Erioptera; specificEpithet: divisa; scientificNameAuthorship: (Walker, 1848); **Location:** country: Russia; stateProvince: Central European Russia; county: Moskovskaya Oblast; municipality: Solnechnogorsk district; locality: Chashnikovo; verbatimElevation: 220 m; minimumElevationInMeters: 220; decimalLatitude: 56.0375; decimalLongitude: 37.1874; **Identification:** identifiedBy: V.E. Pilipenko; **Event:** samplingProtocol: Sweep net; eventDate: 1996-06-16; verbatimEventDate: Jun-16-1996; **Record Level:** institutionCode: VPMC; basisOfRecord: PreservedSpecimen

#### Distribution

First records from Russia: RUC.

### Erioptera (Erioptera) flavata

(Westhoff, 1882)

86C29F64-659B-5652-9E9E-76A7EF27572C

https://ccw.naturalis.nl/detail.php?id=999

#### Materials

- **Type status:** Other material. **Occurrence:** occurrenceRemarks: 1 female; recordedBy: D.I. Gavryushin; individualCount: 1; sex: female; occurrenceID: EU_LIM_351; **Taxon:** scientificName: Erioptera (Erioptera) flavata (Westhoff, 1882); family: Limoniidae; genus: Erioptera; subgenus: Erioptera; specificEpithet: flavata; scientificNameAuthorship: (Westhoff, 1882); **Location:** country: Russia; stateProvince: East European Russia; county: Bashkortostan Respublika; municipality: Beloretsk district; locality: Nura River (ca. 4km W of Otnurok village), at the foot of Zolotyie Shishki (Golden Cones) Mts.; verbatimElevation: 607 m; minimumElevationInMeters: 607; decimalLatitude: 54.05155; decimalLongitude: 58.26887; **Identification:** identifiedBy: D.I. Gavryushin; **Event:** samplingProtocol: Sweep net; eventDate: 2015-06-10; verbatimEventDate: Jul-10-2015; **Record Level:** institutionCode: ZMMU; basisOfRecord: PreservedSpecimen
- **Type status:** Other material. **Occurrence:** occurrenceRemarks: 3 males, 1 female; recordedBy: D.I. Gavryushin; individualCount: 4; sex: male, female; occurrenceID: EU_LIM_352; **Taxon:** scientificName: Erioptera (Erioptera) flavata (Westhoff, 1882); family: Limoniidae; genus: Erioptera; subgenus: Erioptera; specificEpithet: flavata; scientificNameAuthorship: (Westhoff, 1882); **Location:** country: Russia; stateProvince: East European Russia; county: Bashkortostan Respublika; municipality: Beloretsk district; locality: Makhmutovo env., Belaya River; verbatimElevation: 550 m; minimumElevationInMeters: 550; decimalLatitude: 54.33012; decimalLongitude: 58.80735; **Identification:** identifiedBy: D.I. Gavryushin; **Event:** samplingProtocol: Sweep net; eventDate: 2015-06-15; verbatimEventDate: 15/Jul/2015; **Record Level:** institutionCode: ZMMU; basisOfRecord: PreservedSpecimen

#### Distribution

First records from Russia: RUE.

### Erioptera (Erioptera) fusculenta

Edwards, 1938

DB588ABF-53B5-5226-899A-D248406C3F07

https://ccw.naturalis.nl/detail.php?id=1007

#### Materials

- **Type status:** Other material. **Occurrence:** occurrenceRemarks: 2 males; recordedBy: D.I. Gavryushin; individualCount: 2; sex: male; occurrenceID: EU_LIM_353; **Taxon:** scientificName: Erioptera (Erioptera) fusculenta Edwards, 1938; family: Limoniidae; genus: Erioptera; subgenus: Erioptera; specificEpithet: fusculenta; scientificNameAuthorship: Edwards, 1938; **Location:** country: Russia; stateProvince: East European Russia; county: Bashkortostan Respublika; municipality: Beloretsk district; locality: Abzakovo env., Karan River; verbatimElevation: 533 m; minimumElevationInMeters: 533; decimalLatitude: 53.83717; decimalLongitude: 58.57878; **Identification:** identifiedBy: D.I. Gavryushin; **Event:** samplingProtocol: Sweep net; eventDate: 2015-06-17; verbatimEventDate: Jul-17-2015; **Record Level:** institutionCode: ZMMU; basisOfRecord: PreservedSpecimen
- **Type status:** Other material. **Occurrence:** occurrenceRemarks: 1 female; recordedBy: V.E. Pilipenko; individualCount: 1; sex: female; occurrenceID: EU_LIM_354; **Taxon:** scientificName: Erioptera (Erioptera) fusculenta Edwards, 1938; family: Limoniidae; genus: Erioptera; subgenus: Erioptera; specificEpithet: fusculenta; scientificNameAuthorship: Edwards, 1938; **Location:** country: Russia; stateProvince: Central European Russia; county: Moskovskaya Oblast; municipality: Solnechnogorsk district; locality: Chashnikovo; verbatimElevation: 220 m; minimumElevationInMeters: 220; decimalLatitude: 56.0375; decimalLongitude: 37.1874; **Identification:** identifiedBy: V.E. Pilipenko; **Event:** samplingProtocol: Sweep net; eventDate: 1993-08-30; verbatimEventDate: 30/Aug/1993; **Record Level:** institutionCode: VPMC; basisOfRecord: PreservedSpecimen

#### Distribution

First records from Russia: RUC, RUE.

### Erioptera (Erioptera) griseipennis

Meigen, 1838

98FA6C60-974A-501B-B1FB-E9EA61C3B2EC

https://ccw.naturalis.nl/detail.php?id=1016

#### Materials

- **Type status:** Other material. **Occurrence:** catalogNumber: 524929, 644174; occurrenceRemarks: 2 males; recordedBy: K.M. Olsen; individualCount: 2; sex: male; preparations: Ethanol; occurrenceID: EU_LIM_355; **Taxon:** scientificName: Erioptera (Erioptera) griseipennis Meigen, 1838; family: Limoniidae; genus: Erioptera; subgenus: Erioptera; specificEpithet: griseipennis; scientificNameAuthorship: Meigen, 1838; **Location:** country: Norway; stateProvince: Akershus; municipality: Asker; locality: Brønnøya – ESE Kuhavna; verbatimElevation: 0.5 m; minimumElevationInMeters: 0.5; decimalLatitude: 59.85636; decimalLongitude: 10.54636; **Identification:** identifiedBy: K.M. Olsen; **Event:** samplingProtocol: Sweep net; eventDate: 2017-06-05; verbatimEventDate: 05/Jun/2017; **Record Level:** institutionCode: PCKMO; basisOfRecord: PreservedSpecimen
- **Type status:** Other material. **Occurrence:** catalogNumber: 581560; occurrenceRemarks: 1 male; recordedBy: K.M. Olsen; individualCount: 1; sex: male; preparations: Ethanol; occurrenceID: EU_LIM_356; **Taxon:** scientificName: Erioptera (Erioptera) griseipennis Meigen, 1838; family: Limoniidae; genus: Erioptera; subgenus: Erioptera; specificEpithet: griseipennis; scientificNameAuthorship: Meigen, 1838; **Location:** country: Norway; stateProvince: Akershus; municipality: Asker; locality: Orestien – Ved nr. 7; verbatimElevation: 40 m; minimumElevationInMeters: 40; decimalLatitude: 59.84243; decimalLongitude: 10.46826; **Identification:** identifiedBy: K.M. Olsen; **Event:** samplingProtocol: Sweep net; eventDate: 2018-06-06; verbatimEventDate: 06/Jun/2018; **Record Level:** institutionCode: NHMO; basisOfRecord: PreservedSpecimen
- **Type status:** Other material. **Occurrence:** catalogNumber: 559130; occurrenceRemarks: 1 male; recordedBy: K.M. Olsen | T. Blindheim; individualCount: 1; sex: male; preparations: Ethanol; occurrenceID: EU_LIM_357; **Taxon:** scientificName: Erioptera (Erioptera) griseipennis Meigen, 1838; family: Limoniidae; genus: Erioptera; subgenus: Erioptera; specificEpithet: griseipennis; scientificNameAuthorship: Meigen, 1838; **Location:** country: Norway; stateProvince: Akershus; municipality: Skedsmo; locality: SSE Øvre Tærud; verbatimElevation: 155 m; minimumElevationInMeters: 155; decimalLatitude: 60.00445; decimalLongitude: 11.01449; **Identification:** identifiedBy: K.M. Olsen; **Event:** samplingProtocol: Malaise trap; eventDate: 2017-06-20/2017-07-31; verbatimEventDate: 20/Jun-31/Jul/2017; **Record Level:** institutionCode: NHMO; basisOfRecord: PreservedSpecimen
- **Type status:** Other material. **Occurrence:** catalogNumber: 641069; occurrenceRemarks: 26 males; recordedBy: H. Elven; individualCount: 26; sex: male; preparations: Ethanol; occurrenceID: EU_LIM_358; **Taxon:** scientificName: Erioptera (Erioptera) griseipennis Meigen, 1838; family: Limoniidae; genus: Erioptera; subgenus: Erioptera; specificEpithet: griseipennis; scientificNameAuthorship: Meigen, 1838; **Location:** country: Norway; stateProvince: Oslo; municipality: Oslo; locality: Finnerud; verbatimElevation: 347 m; minimumElevationInMeters: 347; decimalLatitude: 60.02948; decimalLongitude: 10.63866; **Identification:** identifiedBy: K.M. Olsen; **Event:** samplingProtocol: Malaise trap; eventDate: 2018-06-18/2018-07-05; verbatimEventDate: 18/Jun-05/Jul/2018; **Record Level:** institutionCode: NHMO; basisOfRecord: PreservedSpecimen

#### Distribution

First records from Norway.

### Erioptera (Erioptera) limbata

Loew, 1873

1F590178-AB22-56CF-AD8B-07F2F42FAEA3

https://ccw.naturalis.nl/detail.php?id=1032

#### Materials

- **Type status:** Other material. **Occurrence:** occurrenceRemarks: 1 male; recordedBy: D.I. Gavryushin; individualCount: 1; sex: male; occurrenceID: EU_LIM_359; **Taxon:** scientificName: Erioptera (Erioptera) limbata Loew, 1873; family: Limoniidae; genus: Erioptera; subgenus: Erioptera; specificEpithet: limbata; scientificNameAuthorship: Loew, 1873; **Location:** country: Russia; stateProvince: East European Russia; county: Bashkortostan Respublika; municipality: Beloretsk district; locality: Abzakovo env., Malyi Kizil River; verbatimElevation: 510 m; minimumElevationInMeters: 510; decimalLatitude: 53.81428; decimalLongitude: 58.5942; **Identification:** identifiedBy: D.I. Gavryushin; **Event:** samplingProtocol: Sweep net; eventDate: 2015-06-12; verbatimEventDate: Jul-12-2015; **Record Level:** institutionCode: ZMMU; basisOfRecord: PreservedSpecimen

#### Distribution

First record from Russia: RUE.

### Erioptera (Erioptera) longicauda

Loew, 1871

8E157993-8FD6-5952-BD5A-B3859C412D74

https://ccw.naturalis.nl/detail.php?id=1034

#### Materials

- **Type status:** Other material. **Occurrence:** occurrenceRemarks: 1 male, 1 female; recordedBy: J. Starý; individualCount: 2; sex: male, female; preparations: Pinned; occurrenceID: EU_LIM_360; **Taxon:** scientificName: Erioptera (Erioptera) longicauda Loew, 1871; family: Limoniidae; genus: Erioptera; subgenus: Erioptera; specificEpithet: longicauda; scientificNameAuthorship: Loew, 1871; **Location:** country: Italy; stateProvince: Calabria; municipality: Filogaso; locality: 2.9 km NE; verbatimElevation: 100 m; minimumElevationInMeters: 100; decimalLatitude: 38.7; decimalLongitude: 16.25028; **Identification:** identifiedBy: J. Starý; **Event:** eventDate: 2018-05-26; verbatimEventDate: 26/May/2018; habitat: spring areas, brook; **Record Level:** institutionCode: PCJS; basisOfRecord: PreservedSpecimen

#### Distribution

First record from Italy (from mainland).

### Erioptera (Erioptera) lutea lutea

Meigen, 1804

E91EA940-4C4D-5930-9275-1BE7F06787BA

https://ccw.naturalis.nl/detail.php?id=1039

#### Materials

- **Type status:** Other material. **Occurrence:** occurrenceRemarks: 1 male; recordedBy: D.I. Gavryushin; individualCount: 1; sex: male; preparations: Pinned; occurrenceID: EU_LIM_361; **Taxon:** scientificName: Erioptera (Erioptera) lutea >lutea Meigen, 1804; family: Limoniidae; genus: Erioptera; subgenus: Erioptera; specificEpithet: lutea; infraspecificEpithet: lutea; scientificNameAuthorship: Meigen, 1804; **Location:** country: Belarus; stateProvince: Minsk; county: Barysaw; locality: Vialikaje Stachava; verbatimElevation: 156 m; minimumElevationInMeters: 156; decimalLatitude: 54.26555; decimalLongitude: 28.38332; **Identification:** identifiedBy: D.I. Gavryushin; **Event:** samplingProtocol: Sweep net; eventDate: 2013-06-07; verbatimEventDate: 7/Jul/2013; **Record Level:** institutionCode: ZMMU; basisOfRecord: PreservedSpecimen
- **Type status:** Other material. **Occurrence:** occurrenceRemarks: 1 female; recordedBy: D.I. Gavryushin; individualCount: 1; sex: female; occurrenceID: EU_LIM_362; **Taxon:** scientificName: Erioptera (Erioptera) lutea >lutea Meigen, 1804; family: Limoniidae; genus: Erioptera; subgenus: Erioptera; specificEpithet: lutea; infraspecificEpithet: lutea; scientificNameAuthorship: Meigen, 1804; **Location:** country: Russia; stateProvince: East European Russia; county: Bashkortostan Respublika; municipality: Beloretsk district; locality: Abzakovo env., Malyi Kizil River; verbatimElevation: 510 m; minimumElevationInMeters: 510; decimalLatitude: 53.81428; decimalLongitude: 58.5942; **Identification:** identifiedBy: D.I. Gavryushin; **Event:** samplingProtocol: Sweep net; eventDate: 2015-06-12; verbatimEventDate: 12/Jul/2015; **Record Level:** institutionCode: ZMMU; basisOfRecord: PreservedSpecimen
- **Type status:** Other material. **Occurrence:** occurrenceRemarks: 6 males; recordedBy: D.I. Gavryushin; individualCount: 6; sex: male; occurrenceID: EU_LIM_363; **Taxon:** scientificName: Erioptera (Erioptera) lutea >lutea Meigen, 1804; family: Limoniidae; genus: Erioptera; subgenus: Erioptera; specificEpithet: lutea; infraspecificEpithet: lutea; scientificNameAuthorship: Meigen, 1804; **Location:** country: Russia; stateProvince: East European Russia; county: Bashkortostan Respublika; municipality: Beloretsk district; locality: Abzakovo env., Karan River; verbatimElevation: 533 m; minimumElevationInMeters: 533; decimalLatitude: 53.83717; decimalLongitude: 58.57878; **Identification:** identifiedBy: D.I. Gavryushin; **Event:** samplingProtocol: Sweep net; eventDate: 2015-06-17; verbatimEventDate: 17/Jul/2015; **Record Level:** institutionCode: ZMMU; basisOfRecord: PreservedSpecimen
- **Type status:** Other material. **Occurrence:** occurrenceRemarks: 2 males; recordedBy: D.I. Gavryushin; individualCount: 2; sex: male; occurrenceID: EU_LIM_364; **Taxon:** scientificName: Erioptera (Erioptera) lutea >lutea Meigen, 1804; family: Limoniidae; genus: Erioptera; subgenus: Erioptera; specificEpithet: lutea; infraspecificEpithet: lutea; scientificNameAuthorship: Meigen, 1804; **Location:** country: Russia; stateProvince: East European Russia; county: Bashkortostan Respublika; municipality: Beloretsk district; locality: Abzakovo env., Karan River; verbatimElevation: 533 m; minimumElevationInMeters: 533; decimalLatitude: 53.83717; decimalLongitude: 58.57878; **Identification:** identifiedBy: D.I. Gavryushin; **Event:** samplingProtocol: Sweep net; eventDate: 2015-06-19; verbatimEventDate: 19/Jul/2015; **Record Level:** institutionCode: ZMMU; basisOfRecord: PreservedSpecimen
- **Type status:** Other material. **Occurrence:** occurrenceRemarks: 1 male; recordedBy: D.I. Gavryushin; individualCount: 1; sex: male; occurrenceID: EU_LIM_365; **Taxon:** scientificName: Erioptera (Erioptera) lutea >lutea Meigen, 1804; family: Limoniidae; genus: Erioptera; subgenus: Erioptera; specificEpithet: lutea; infraspecificEpithet: lutea; scientificNameAuthorship: Meigen, 1804; **Location:** country: Russia; stateProvince: East European Russia; county: Bashkortostan Respublika; municipality: Beloretsk district; locality: Abzakovo env., Kulsugady River; verbatimElevation: 531 m; minimumElevationInMeters: 531; decimalLatitude: 53.83795; decimalLongitude: 58.5823; **Identification:** identifiedBy: D.I. Gavryushin; **Event:** samplingProtocol: Sweep net; eventDate: 2015-06-17; verbatimEventDate: 17/Jul/2015; **Record Level:** institutionCode: ZMMU; basisOfRecord: PreservedSpecimen
- **Type status:** Other material. **Occurrence:** occurrenceRemarks: 1 female; recordedBy: D.I. Gavryushin; individualCount: 1; sex: female; occurrenceID: EU_LIM_366; **Taxon:** scientificName: Erioptera (Erioptera) lutea >lutea Meigen, 1804; family: Limoniidae; genus: Erioptera; subgenus: Erioptera; specificEpithet: lutea; infraspecificEpithet: lutea; scientificNameAuthorship: Meigen, 1804; **Location:** country: Russia; stateProvince: East European Russia; county: Bashkortostan Respublika; municipality: Beloretsk district; locality: Abzakovo env., Kulsugady River; verbatimElevation: 531 m; minimumElevationInMeters: 531; decimalLatitude: 53.83795; decimalLongitude: 58.5823; **Identification:** identifiedBy: D.I. Gavryushin; **Event:** samplingProtocol: Sweep net; eventDate: 2012-08-15; verbatimEventDate: 15/Aug/2012; **Record Level:** institutionCode: ZMMU; basisOfRecord: PreservedSpecimen
- **Type status:** Other material. **Occurrence:** occurrenceRemarks: 2 males; recordedBy: D.I. Gavryushin; individualCount: 2; sex: male; occurrenceID: EU_LIM_367; **Taxon:** scientificName: Erioptera (Erioptera) lutea >lutea Meigen, 1804; family: Limoniidae; genus: Erioptera; subgenus: Erioptera; specificEpithet: lutea; infraspecificEpithet: lutea; scientificNameAuthorship: Meigen, 1804; **Location:** country: Russia; stateProvince: East European Russia; county: Bashkortostan Respublika; municipality: Uchaly district; locality: Ural-Tau St. env.; verbatimElevation: 777 m; minimumElevationInMeters: 777; decimalLatitude: 53.96805; decimalLongitude: 58.57613; **Identification:** identifiedBy: D.I. Gavryushin; **Event:** samplingProtocol: Sweep net; eventDate: 2015-06-09; verbatimEventDate: 09/Jul/2015; **Record Level:** institutionCode: ZMMU; basisOfRecord: PreservedSpecimen
- **Type status:** Other material. **Occurrence:** occurrenceRemarks: 1 male; recordedBy: D.I. Gavryushin; individualCount: 1; sex: male; occurrenceID: EU_LIM_368; **Taxon:** scientificName: Erioptera (Erioptera) lutea >lutea Meigen, 1804; family: Limoniidae; genus: Erioptera; subgenus: Erioptera; specificEpithet: lutea; infraspecificEpithet: lutea; scientificNameAuthorship: Meigen, 1804; **Location:** country: Russia; stateProvince: East European Russia; county: Bashkortostan Respublika; municipality: Beloretsk district; locality: Nura River (ca. 4km W of Otnurok village), at the foot of Zolotyie Shishki (Golden Cones) Mts.; verbatimElevation: 607 m; minimumElevationInMeters: 607; decimalLatitude: 54.05155; decimalLongitude: 58.26887; **Identification:** identifiedBy: D.I. Gavryushin; **Event:** samplingProtocol: Sweep net; eventDate: 2015-06-13; verbatimEventDate: 13/Jul/2015; **Record Level:** institutionCode: ZMMU; basisOfRecord: PreservedSpecimen
- **Type status:** Other material. **Occurrence:** occurrenceRemarks: 1 male; recordedBy: D.I. Gavryushin; individualCount: 1; sex: male; occurrenceID: EU_LIM_369; **Taxon:** scientificName: Erioptera (Erioptera) lutea >lutea Meigen, 1804; family: Limoniidae; genus: Erioptera; subgenus: Erioptera; specificEpithet: lutea; infraspecificEpithet: lutea; scientificNameAuthorship: Meigen, 1804; **Location:** country: Russia; stateProvince: East European Russia; county: Bashkortostan Respublika; municipality: Beloretsk district; locality: Makhmutovo env., Belaya River; verbatimElevation: 550 m; minimumElevationInMeters: 550; decimalLatitude: 54.33012; decimalLongitude: 58.80735; **Identification:** identifiedBy: D.I. Gavryushin; **Event:** samplingProtocol: Sweep net; eventDate: 2015-06-15; verbatimEventDate: 15/Jul/2015; **Record Level:** institutionCode: ZMMU; basisOfRecord: PreservedSpecimen
- **Type status:** Other material. **Occurrence:** occurrenceRemarks: 1 male; recordedBy: Sh.A. Murtazin; individualCount: 1; sex: male; occurrenceID: EU_LIM_370; **Taxon:** scientificName: Erioptera (Erioptera) lutea >lutea Meigen, 1804; family: Limoniidae; genus: Erioptera; subgenus: Erioptera; specificEpithet: lutea; infraspecificEpithet: lutea; scientificNameAuthorship: Meigen, 1804; **Location:** country: Russia; stateProvince: East European Russia; county: Bashkortostan Respublika; municipality: Beloretsk district; locality: Otnurok village; verbatimElevation: 615 m; minimumElevationInMeters: 615; decimalLatitude: 54.06062; decimalLongitude: 58.25379; **Identification:** identifiedBy: N.M. Paramonov; **Event:** samplingProtocol: Sweep net; eventDate: 2017-09-09; verbatimEventDate: 09/Sep/2017; **Record Level:** institutionCode: ZIN; basisOfRecord: PreservedSpecimen

#### Distribution

First records from Belarus and Russia: RUE.

### Erioptera (Erioptera) meijerei

Edwards, 1921

615C94C6-6D8C-504E-BA12-1D7BAD5254AF

https://ccw.naturalis.nl/detail.php?id=1046

#### Materials

- **Type status:** Other material. **Occurrence:** occurrenceRemarks: 3 males, 4 females; recordedBy: J. Starý; individualCount: 7; sex: male, female; preparations: Pinned; occurrenceID: EU_LIM_371; **Taxon:** scientificName: Erioptera (Erioptera) meijerei Edwards, 1921; family: Limoniidae; genus: Erioptera; subgenus: Erioptera; specificEpithet: meijerei; scientificNameAuthorship: Edwards, 1921; **Location:** country: Greece; stateProvince: Peloponnese; municipality: Voupraisio; locality: 0.6 km W, Limni marshland; verbatimElevation: -3 m; minimumElevationInMeters: -3; decimalLatitude: 38.066; decimalLongitude: 21.55; **Identification:** identifiedBy: J. Starý; **Event:** eventDate: 2015-05-26; verbatimEventDate: 26/May/2015; **Record Level:** institutionCode: PCJS; basisOfRecord: PreservedSpecimen
- **Type status:** Other material. **Occurrence:** occurrenceRemarks: 5 males, 3 females; recordedBy: J. Starý; individualCount: 8; sex: male, female; preparations: Pinned; occurrenceID: EU_LIM_372; **Taxon:** scientificName: Erioptera (Erioptera) meijerei Edwards, 1921; family: Limoniidae; genus: Erioptera; subgenus: Erioptera; specificEpithet: meijerei; scientificNameAuthorship: Edwards, 1921; **Location:** country: Greece; stateProvince: Peloponnese; municipality: Voupraisio; locality: 0.6 km W, Limni marshland; verbatimElevation: -3 m; minimumElevationInMeters: -3; decimalLatitude: 38.066; decimalLongitude: 21.55; **Identification:** identifiedBy: J. Starý; **Event:** eventDate: 2015-05-28; verbatimEventDate: 28/May/2015; **Record Level:** institutionCode: PCJS; basisOfRecord: PreservedSpecimen

#### Distribution

First records from Greece (from mainland).

### Erioptera (Erioptera) nielseni

de Meijere, 1921

EA9C0E14-6576-5981-93CA-35F3038F53A1

https://ccw.naturalis.nl/detail.php?id=1051

#### Materials

- **Type status:** Other material. **Occurrence:** catalogNumber: 648531; occurrenceRemarks: 14 male+female; recordedBy: K.M. Olsen; individualCount: 14; sex: male, female; preparations: Ethanol; occurrenceID: EU_LIM_373; **Taxon:** scientificName: Erioptera (Erioptera) nielseni de Meijere, 1921; family: Limoniidae; genus: Erioptera; subgenus: Erioptera; specificEpithet: nielseni; scientificNameAuthorship: de Meijere, 1921; **Location:** country: Norway; stateProvince: Hedmark; municipality: Trysil; locality: Møkkelbrynnbrennene; verbatimElevation: 640 m; minimumElevationInMeters: 640; decimalLatitude: 61.40042; decimalLongitude: 12.34231; **Identification:** identifiedBy: K.M. Olsen; **Event:** samplingProtocol: Sweep net; eventDate: 2019-06-12; verbatimEventDate: Jul-12-2019; **Record Level:** institutionCode: PCKMO; basisOfRecord: PreservedSpecimen
- **Type status:** Other material. **Occurrence:** occurrenceRemarks: 1 male; recordedBy: A. Polevoi; individualCount: 1; sex: male; preparations: Pinned; occurrenceID: EU_LIM_374; **Taxon:** scientificName: Erioptera (Erioptera) nielseni de Meijere, 1921; family: Limoniidae; genus: Erioptera; subgenus: Erioptera; specificEpithet: nielseni; scientificNameAuthorship: de Meijere, 1921; **Location:** country: Russia; stateProvince: North European Russia; county: Republic Karelia; municipality: Medvezhegorsk district; locality: Uzkaya Salma, Lake Rugozero; verbatimElevation: 50 m; minimumElevationInMeters: 50; decimalLatitude: 62.15173; decimalLongitude: 34.94471; **Identification:** identifiedBy: A. Polevoi; **Event:** samplingProtocol: Malaise trap; eventDate: 2013-06-22/2013-08-22; verbatimEventDate: 22/Jun-22/Aug/2013; **Record Level:** institutionCode: FRIP; basisOfRecord: PreservedSpecimen

#### Distribution

First records from Norway and Russia: RUN.

### Erioptera (Erioptera) sordida

Zetterstedt, 1838

FA2E31E5-4CD5-58AE-BFD4-0D820633BFE9

https://ccw.naturalis.nl/detail.php?id=1084

#### Materials

- **Type status:** Other material. **Occurrence:** occurrenceRemarks: 1 female; recordedBy: V.E. Pilipenko; individualCount: 1; sex: female; occurrenceID: EU_LIM_375; **Taxon:** scientificName: Erioptera (Erioptera) sordida Zetterstedt, 1838; family: Limoniidae; genus: Erioptera; subgenus: Erioptera; specificEpithet: sordida; scientificNameAuthorship: Zetterstedt, 1838; **Location:** country: Russia; stateProvince: Central European Russia; county: Moskovskaya Oblast; municipality: Ruza district; locality: Glubokoe Lake; verbatimElevation: 200 m; minimumElevationInMeters: 200; decimalLatitude: 55.7539; decimalLongitude: 36.50491; **Identification:** identifiedBy: V.E. Pilipenko; **Event:** samplingProtocol: Sweep net; eventDate: 1997-06-07; verbatimEventDate: Jun-07-1997; **Record Level:** institutionCode: VPMC; basisOfRecord: PreservedSpecimen
- **Type status:** Other material. **Occurrence:** occurrenceRemarks: 1 male; recordedBy: A. Polevoi; individualCount: 1; sex: male; preparations: Pinned; occurrenceID: EU_LIM_376; **Taxon:** scientificName: Erioptera (Erioptera) sordida Zetterstedt, 1838; family: Limoniidae; genus: Erioptera; subgenus: Erioptera; specificEpithet: sordida; scientificNameAuthorship: Zetterstedt, 1838; **Location:** country: Russia; stateProvince: North European Russia; county: Republic Karelia; municipality: Suojarvi district; locality: Ar'koila, 1.5 km NE; verbatimElevation: 163 m; minimumElevationInMeters: 163; decimalLatitude: 61.93518; decimalLongitude: 32.84214; **Identification:** identifiedBy: A. Polevoi; **Event:** samplingProtocol: Sweep net; eventDate: 2018-06-19; verbatimEventDate: 19/Jun/2018; **Record Level:** institutionCode: FRIP; basisOfRecord: PreservedSpecimen

#### Distribution

First records from Russia: RUC, RUN.

### Erioptera (Erioptera) squalida

Loew, 1871

884E696D-7AEC-5C67-B931-E935AC3BEF08

https://ccw.naturalis.nl/detail.php?id=1085

#### Materials

- **Type status:** Other material. **Occurrence:** catalogNumber: 669727; occurrenceRemarks: 1 female; recordedBy: K.M. Olsen; individualCount: 1; sex: female; preparations: Ethanol; occurrenceID: EU_LIM_377; **Taxon:** scientificName: Erioptera (Erioptera) squalida Loew, 1871; family: Limoniidae; genus: Erioptera; subgenus: Erioptera; specificEpithet: squalida; scientificNameAuthorship: Loew, 1871; **Location:** country: Norway; stateProvince: Buskerud; municipality: Hurum; locality: Verket; verbatimElevation: 50 m; minimumElevationInMeters: 50; decimalLatitude: 59.61885; decimalLongitude: 10.44178; **Identification:** identifiedBy: K.M. Olsen; **Event:** samplingProtocol: Malaise trap; eventDate: 2020-06-11/2020-07-31; verbatimEventDate: 11/Jun-31/Jul/2020; habitat: Østre deler av sandtaket; **Record Level:** institutionCode: PCKMO; basisOfRecord: PreservedSpecimen
- **Type status:** Other material. **Occurrence:** occurrenceRemarks: 1 male; recordedBy: V.E. Pilipenko; individualCount: 1; sex: male; occurrenceID: EU_LIM_378; **Taxon:** scientificName: Erioptera (Erioptera) squalida Loew, 1871; family: Limoniidae; genus: Erioptera; subgenus: Erioptera; specificEpithet: squalida; scientificNameAuthorship: Loew, 1871; **Location:** country: Russia; stateProvince: Central European Russia; county: Moskovskaya Oblast; municipality: Taldom district; locality: city Dubna; verbatimElevation: 125 m; minimumElevationInMeters: 125; decimalLatitude: 56.71431; decimalLongitude: 37.13492; **Identification:** identifiedBy: V.E. Pilipenko; **Event:** samplingProtocol: Sweep net; eventDate: 1997-06-16; verbatimEventDate: 16/Jul/1997; **Record Level:** institutionCode: VPMC; basisOfRecord: PreservedSpecimen

#### Distribution

First records from Norway and Russia: RUC.

### Euphylidorea (Euphylidorea) aperta

(Verrall, 1887)

0C53AA21-BFFB-5D77-8DCE-972408EE3489

https://ccw.naturalis.nl/detail.php?id=5445

#### Materials

- **Type status:** Other material. **Occurrence:** catalogNumber: 611980; occurrenceRemarks: 1 male; recordedBy: K. Berggren; individualCount: 1; sex: male; preparations: Ethanol; occurrenceID: EU_LIM_379; **Taxon:** scientificName: Euphylidorea (Euphylidorea) aperta (Verrall, 1887); family: Limoniidae; genus: Euphylidorea; subgenus: Euphylidorea; specificEpithet: aperta; scientificNameAuthorship: (Verrall, 1887); **Location:** country: Norway; stateProvince: Aust-Agder; municipality: Grimstad; locality: Søm (Skogstuen); verbatimElevation: 10 m; minimumElevationInMeters: 10; decimalLatitude: 58.3896; decimalLongitude: 8.71272; **Identification:** identifiedBy: K.M. Olsen; **Event:** samplingProtocol: Light trap; eventDate: 2017-08; verbatimEventDate: Aug/2017; **Record Level:** institutionCode: PCKMO; basisOfRecord: PreservedSpecimen
- **Type status:** Other material. **Occurrence:** occurrenceRemarks: 1 male; recordedBy: H. Andersson; individualCount: 1; sex: male; preparations: Pinned; occurrenceID: EU_LIM_380; **Taxon:** scientificName: Euphylidorea (Euphylidorea) aperta (Verrall, 1887); family: Limoniidae; genus: Euphylidorea; subgenus: Euphylidorea; specificEpithet: aperta; scientificNameAuthorship: (Verrall, 1887); **Location:** country: Sweden; stateProvince: Halland; municipality: Halmstad; locality: Årnilt, Enslöv; verbatimElevation: 1000 m; minimumElevationInMeters: 1000; decimalLatitude: 56.81; decimalLongitude: 13.05; **Identification:** identifiedBy: B. Tjeder; **Event:** eventDate: 1974-08-08; verbatimEventDate: 8/Aug/1974; **Record Level:** institutionCode: MZLU; basisOfRecord: PreservedSpecimen

#### Distribution

First records from Norway and Sweden.

### Euphylidorea (Euphylidorea) dispar

(Meigen, 1818)

5A7E7E1E-28C1-57F5-BFE7-13407D40F0A6

https://ccw.naturalis.nl/detail.php?id=5458

#### Materials

- **Type status:** Other material. **Occurrence:** occurrenceRemarks: 1 male; recordedBy: J. Starý; individualCount: 1; sex: male; preparations: Pinned; occurrenceID: EU_LIM_381; **Taxon:** scientificName: Euphylidorea (Euphylidorea) dispar (Meigen, 1818); family: Limoniidae; genus: Euphylidorea; subgenus: Euphylidorea; specificEpithet: dispar; scientificNameAuthorship: (Meigen, 1818); **Location:** island: Sicily; country: Italy; stateProvince: Sicily; municipality: Raccuja; locality: 0.9 km W; verbatimElevation: 450 m; minimumElevationInMeters: 450; decimalLatitude: 38.05389; decimalLongitude: 14.90083; **Identification:** identifiedBy: J. Starý; **Event:** eventDate: Apr-25-2016; verbatimEventDate: Apr-25-2016; **Record Level:** institutionCode: PCJS; basisOfRecord: PreservedSpecimen
- **Type status:** Other material. **Occurrence:** catalogNumber: 614300; occurrenceRemarks: 2 males; recordedBy: F. Midtgaard; individualCount: 2; sex: male; preparations: Ethanol; occurrenceID: EU_LIM_382; **Taxon:** scientificName: Euphylidorea (Euphylidorea) dispar (Meigen, 1818); family: Limoniidae; genus: Euphylidorea; subgenus: Euphylidorea; specificEpithet: dispar; scientificNameAuthorship: (Meigen, 1818); **Location:** country: Norway; stateProvince: Akershus; municipality: Frogn; locality: Håøya – “A”; verbatimElevation: 75 m; minimumElevationInMeters: 75; decimalLatitude: 59.69417; decimalLongitude: 10.5728; **Identification:** identifiedBy: K.M. Olsen; **Event:** samplingProtocol: Malaise trap; eventDate: 1984-06-16/1984-06-27; verbatimEventDate: 16-27/Jun/1984; **Record Level:** institutionCode: NHMO; basisOfRecord: PreservedSpecimen
- **Type status:** Other material. **Occurrence:** catalogNumber: 531164; occurrenceRemarks: 2 male+female; recordedBy: S. Svendsen | K. Berggren; individualCount: 2; sex: male, female; preparations: Ethanol; occurrenceID: EU_LIM_383; **Taxon:** scientificName: Euphylidorea (Euphylidorea) dispar (Meigen, 1818); family: Limoniidae; genus: Euphylidorea; subgenus: Euphylidorea; specificEpithet: dispar; scientificNameAuthorship: (Meigen, 1818); **Location:** country: Norway; stateProvince: Aust-Agder; municipality: Birkenes; locality: Nordåsen; verbatimElevation: 85 m; minimumElevationInMeters: 85; decimalLatitude: 58.33342; decimalLongitude: 8.24004; **Identification:** identifiedBy: J. Starý; **Event:** samplingProtocol: Light trap; eventDate: 2016-09; verbatimEventDate: Sep-2016; **Record Level:** institutionCode: NHMO; basisOfRecord: PreservedSpecimen
- **Type status:** Other material. **Occurrence:** catalogNumber: 586665; occurrenceRemarks: 1 female; recordedBy: S. Olberg | Ø. Gammelmo; individualCount: 1; sex: female; preparations: Ethanol; occurrenceID: EU_LIM_384; **Taxon:** scientificName: Euphylidorea (Euphylidorea) dispar (Meigen, 1818); family: Limoniidae; genus: Euphylidorea; subgenus: Euphylidorea; specificEpithet: dispar; scientificNameAuthorship: (Meigen, 1818); **Location:** country: Norway; stateProvince: Østfold; municipality: Hvaler; locality: Makø; verbatimElevation: 5 m; minimumElevationInMeters: 5; decimalLatitude: 59.04704; decimalLongitude: 11.11083; **Identification:** identifiedBy: K.M. Olsen; **Event:** samplingProtocol: Window trap; eventDate: 2018-05-09/2018-06-12; verbatimEventDate: 09/May-12/Jun/2018; **Record Level:** institutionCode: PCKMO; basisOfRecord: PreservedSpecimen
- **Type status:** Other material. **Occurrence:** catalogNumber: 528840; occurrenceRemarks: 2 males; recordedBy: K. Berggren; individualCount: 2; sex: male; preparations: Ethanol; occurrenceID: EU_LIM_385; **Taxon:** scientificName: Euphylidorea (Euphylidorea) dispar (Meigen, 1818); family: Limoniidae; genus: Euphylidorea; subgenus: Euphylidorea; specificEpithet: dispar; scientificNameAuthorship: (Meigen, 1818); **Location:** country: Norway; stateProvince: Vest-Agder; municipality: Kristiansand; locality: Nedre Timenes; verbatimElevation: 10 m; minimumElevationInMeters: 10; decimalLatitude: 58.16155; decimalLongitude: 8.10013; **Identification:** identifiedBy: K.M. Olsen; **Event:** samplingProtocol: Light trap; eventDate: 2017-06; verbatimEventDate: Jun/2017; **Record Level:** institutionCode: PCKMO; basisOfRecord: PreservedSpecimen
- **Type status:** Other material. **Occurrence:** catalogNumber: 531872; occurrenceRemarks: 1 male; recordedBy: K. Berggren; individualCount: 1; sex: male; preparations: Ethanol; occurrenceID: EU_LIM_386; **Taxon:** scientificName: Euphylidorea (Euphylidorea) dispar (Meigen, 1818); family: Limoniidae; genus: Euphylidorea; subgenus: Euphylidorea; specificEpithet: dispar; scientificNameAuthorship: (Meigen, 1818); **Location:** country: Norway; stateProvince: Vest-Agder; municipality: Kristiansand; locality: Nedre Timenes; verbatimElevation: 10 m; minimumElevationInMeters: 10; decimalLatitude: 58.16155; decimalLongitude: 8.10013; **Identification:** identifiedBy: K.M. Olsen; **Event:** samplingProtocol: Light trap; eventDate: 2017-06-01/2017-06-19; verbatimEventDate: 01-19/Jun/2017; **Record Level:** institutionCode: PCKMO; basisOfRecord: PreservedSpecimen
- **Type status:** Other material. **Occurrence:** catalogNumber: 556761; occurrenceRemarks: 3 males; recordedBy: K. Berggren; individualCount: 3; sex: male; preparations: Ethanol; occurrenceID: EU_LIM_387; **Taxon:** scientificName: Euphylidorea (Euphylidorea) dispar (Meigen, 1818); family: Limoniidae; genus: Euphylidorea; subgenus: Euphylidorea; specificEpithet: dispar; scientificNameAuthorship: (Meigen, 1818); **Location:** country: Norway; stateProvince: Vest-Agder; municipality: Kristiansand; locality: Nedre Timenes; verbatimElevation: 10 m; minimumElevationInMeters: 10; decimalLatitude: 58.16155; decimalLongitude: 8.10013; **Identification:** identifiedBy: K.M. Olsen; **Event:** samplingProtocol: Light trap; eventDate: 2017-06-01/2017-06-19; verbatimEventDate: 01-19/Jun/2017; **Record Level:** institutionCode: NHMO; basisOfRecord: PreservedSpecimen
- **Type status:** Other material. **Occurrence:** catalogNumber: 612362; occurrenceRemarks: 5 males; recordedBy: K. Berggren; individualCount: 5; sex: male; preparations: Ethanol; occurrenceID: EU_LIM_388; **Taxon:** scientificName: Euphylidorea (Euphylidorea) dispar (Meigen, 1818); family: Limoniidae; genus: Euphylidorea; subgenus: Euphylidorea; specificEpithet: dispar; scientificNameAuthorship: (Meigen, 1818); **Location:** country: Norway; stateProvince: Vest-Agder; municipality: Kristiansand; locality: Nedre Timenes; verbatimElevation: 10 m; minimumElevationInMeters: 10; decimalLatitude: 58.16155; decimalLongitude: 8.10013; **Identification:** identifiedBy: K.M. Olsen; **Event:** samplingProtocol: Light trap; eventDate: 2018-06; verbatimEventDate: Jun/2018; **Record Level:** institutionCode: NHMO; basisOfRecord: PreservedSpecimen
- **Type status:** Other material. **Occurrence:** catalogNumber: 599025, 529602; occurrenceRemarks: 2 males; recordedBy: K. Berggren; individualCount: 2; sex: male; preparations: Ethanol; occurrenceID: EU_LIM_389; **Taxon:** scientificName: Euphylidorea (Euphylidorea) dispar (Meigen, 1818); family: Limoniidae; genus: Euphylidorea; subgenus: Euphylidorea; specificEpithet: dispar; scientificNameAuthorship: (Meigen, 1818); **Location:** country: Norway; stateProvince: Vest-Agder; municipality: Kristiansand; locality: Nedre Timenes; verbatimElevation: 10 m; minimumElevationInMeters: 10; decimalLatitude: 58.16155; decimalLongitude: 8.10013; **Identification:** identifiedBy: K.M. Olsen | J. Starý; **Event:** samplingProtocol: Light trap; eventDate: 2017-06; verbatimEventDate: Jul/2017; **Record Level:** institutionCode: NHMO | PCKMO; basisOfRecord: PreservedSpecimen
- **Type status:** Other material. **Occurrence:** catalogNumber: 658776; occurrenceRemarks: 2 male+female; recordedBy: K.M. Olsen; individualCount: 2; sex: male, female; preparations: Ethanol; occurrenceID: EU_LIM_390; **Taxon:** scientificName: Euphylidorea (Euphylidorea) dispar (Meigen, 1818); family: Limoniidae; genus: Euphylidorea; subgenus: Euphylidorea; specificEpithet: dispar; scientificNameAuthorship: (Meigen, 1818); **Location:** country: Norway; stateProvince: Vestfold; municipality: Tjøme; locality: Kolabekkilen – Svartorstrandskog i NNE; verbatimElevation: 1 m; minimumElevationInMeters: 1; decimalLatitude: 59.09041; decimalLongitude: 10.40467; **Identification:** identifiedBy: K.M. Olsen; **Event:** samplingProtocol: Malaise trap; eventDate: 2019-05-12/2019-07-16; verbatimEventDate: 12/May-16/Jul/2019; **Record Level:** institutionCode: NHMO; basisOfRecord: PreservedSpecimen
- **Type status:** Other material. **Occurrence:** catalogNumber: 536156; occurrenceRemarks: 1 male; recordedBy: K.M. Olsen; individualCount: 1; sex: male; preparations: Ethanol; occurrenceID: EU_LIM_391; **Taxon:** scientificName: Euphylidorea (Euphylidorea) dispar (Meigen, 1818); family: Limoniidae; genus: Euphylidorea; subgenus: Euphylidorea; specificEpithet: dispar; scientificNameAuthorship: (Meigen, 1818); **Location:** country: Norway; stateProvince: Vestfold; municipality: Horten; locality: Mellomøya – Midtre deler; verbatimElevation: 10 m; minimumElevationInMeters: 10; decimalLatitude: 59.44413; decimalLongitude: 10.46238; **Identification:** identifiedBy: K.M. Olsen; **Event:** samplingProtocol: Sweep net; eventDate: 2017-06-17; verbatimEventDate: 17/Jun/2017; **Record Level:** institutionCode: NHMO; basisOfRecord: PreservedSpecimen
- **Type status:** Other material. **Occurrence:** catalogNumber: 557409; occurrenceRemarks: 2 males; recordedBy: K.M. Olsen mfl.; individualCount: 2; sex: male; preparations: Ethanol; occurrenceID: EU_LIM_392; **Taxon:** scientificName: Euphylidorea (Euphylidorea) dispar (Meigen, 1818); family: Limoniidae; genus: Euphylidorea; subgenus: Euphylidorea; specificEpithet: dispar; scientificNameAuthorship: (Meigen, 1818); **Location:** country: Norway; stateProvince: Vestfold; municipality: Larvik; locality: NW Nevlungstranda; verbatimElevation: 5 m; minimumElevationInMeters: 5; decimalLatitude: 58.96931; decimalLongitude: 9.84438; **Identification:** identifiedBy: K.M. Olsen; **Event:** samplingProtocol: Sweep net; eventDate: 2017-06-08; verbatimEventDate: 08/Jun/2017; **Record Level:** institutionCode: NHMO; basisOfRecord: PreservedSpecimen
- **Type status:** Other material. **Occurrence:** catalogNumber: 548922; occurrenceRemarks: 1 male; recordedBy: K.M. Olsen | S. Olberg; individualCount: 1; sex: male; preparations: Ethanol; occurrenceID: EU_LIM_393; **Taxon:** scientificName: Euphylidorea (Euphylidorea) dispar (Meigen, 1818); family: Limoniidae; genus: Euphylidorea; subgenus: Euphylidorea; specificEpithet: dispar; scientificNameAuthorship: (Meigen, 1818); **Location:** country: Norway; stateProvince: Vestfold; municipality: Larvik; locality: Sandvikbukta PFO; verbatimElevation: 2 m; minimumElevationInMeters: 2; decimalLatitude: 59.01169; decimalLongitude: 10.14128; **Identification:** identifiedBy: K.M. Olsen; **Event:** samplingProtocol: Sweep net; eventDate: 2017-06-06; verbatimEventDate: 06/Jun/2017; **Record Level:** institutionCode: NHMO; basisOfRecord: PreservedSpecimen
- **Type status:** Other material. **Occurrence:** catalogNumber: 536561; occurrenceRemarks: 1 male; recordedBy: L.O. Hansen; individualCount: 1; sex: male; preparations: Ethanol; occurrenceID: EU_LIM_394; **Taxon:** scientificName: Euphylidorea (Euphylidorea) dispar (Meigen, 1818); family: Limoniidae; genus: Euphylidorea; subgenus: Euphylidorea; specificEpithet: dispar; scientificNameAuthorship: (Meigen, 1818); **Location:** country: Norway; stateProvince: Vestfold; municipality: Re; locality: Langøya; verbatimElevation: 5 m; minimumElevationInMeters: 5; decimalLatitude: 59.49494; decimalLongitude: 10.37418; **Identification:** identifiedBy: K.M. Olsen; **Event:** samplingProtocol: Light trap; eventDate: 1987-06; verbatimEventDate: Jul/1987; **Record Level:** institutionCode: NHMO; basisOfRecord: PreservedSpecimen
- **Type status:** Other material. **Occurrence:** occurrenceRemarks: 1 male; recordedBy: V.E. Pilipenko; individualCount: 1; sex: male; occurrenceID: EU_LIM_395; **Taxon:** scientificName: Euphylidorea (Euphylidorea) dispar (Meigen, 1818); family: Limoniidae; genus: Euphylidorea; subgenus: Euphylidorea; specificEpithet: dispar; scientificNameAuthorship: (Meigen, 1818); **Location:** country: Russia; stateProvince: Central European Russia; county: Moskovskaya Oblast; municipality: Solnechnogorsk district; locality: Chashnikovo; verbatimElevation: 220 m; minimumElevationInMeters: 220; decimalLatitude: 56.0375; decimalLongitude: 37.1874; **Identification:** identifiedBy: V.E. Pilipenko; **Event:** samplingProtocol: Sweep net; eventDate: 1993-06-01; verbatimEventDate: 01/Jul/1993; **Record Level:** institutionCode: VPMC; basisOfRecord: PreservedSpecimen

#### Distribution

First records from Norway and Russia: RUC. The species was previously reported from mainland Italy and here, we report for the first time from Sicily.

### Euphylidorea (Euphylidorea) lineola

(Meigen, 1804)

FAB99A7D-6648-56C3-90EE-EB263A268E35

https://ccw.naturalis.nl/detail.php?id=5468

#### Materials

- **Type status:** Other material. **Occurrence:** occurrenceRemarks: 1 female; recordedBy: V.E. Pilipenko; individualCount: 1; sex: female; occurrenceID: EU_LIM_396; **Taxon:** scientificName: Euphylidorea (Euphylidorea) lineola (Meigen, 1804); family: Limoniidae; genus: Euphylidorea; subgenus: Euphylidorea; specificEpithet: lineola; scientificNameAuthorship: (Meigen, 1804); **Location:** country: Russia; stateProvince: Central European Russia; county: Moskovskaya Oblast; municipality: Solnechnogorsk district; locality: city Zelenograd; verbatimElevation: 200 m; minimumElevationInMeters: 200; decimalLatitude: 55.98722; decimalLongitude: 37.20443; **Identification:** identifiedBy: V.E. Pilipenko; **Event:** samplingProtocol: Sweep net; eventDate: 1989-06-04; verbatimEventDate: Jun-04-1989; **Record Level:** institutionCode: VPMC; basisOfRecord: PreservedSpecimen
- **Type status:** Other material. **Occurrence:** occurrenceRemarks: 1 female; recordedBy: V.E. Pilipenko; individualCount: 1; sex: female; occurrenceID: EU_LIM_397; **Taxon:** scientificName: Euphylidorea (Euphylidorea) lineola (Meigen, 1804); family: Limoniidae; genus: Euphylidorea; subgenus: Euphylidorea; specificEpithet: lineola; scientificNameAuthorship: (Meigen, 1804); **Location:** country: Russia; stateProvince: Central European Russia; county: Moskovskaya Oblast; municipality: Solnechnogorsk district; locality: Chashnikovo; verbatimElevation: 220 m; minimumElevationInMeters: 220; decimalLatitude: 56.0375; decimalLongitude: 37.1874; **Identification:** identifiedBy: V.E. Pilipenko; **Event:** samplingProtocol: Sweep net; eventDate: 1992-06-03; verbatimEventDate: 03/Jun/1992; **Record Level:** institutionCode: VPMC; basisOfRecord: PreservedSpecimen

#### Distribution

First records from Russia: RUC.

### Euphylidorea (Euphylidorea) meigenii

(Verrall, 1886)

0BC3C3CB-1B3F-58AB-B04A-AFC3A099CE53

https://ccw.naturalis.nl/detail.php?id=5472

#### Materials

- **Type status:** Other material. **Occurrence:** occurrenceRemarks: 2 males, 2 females; recordedBy: D.I. Gavryushin; individualCount: 4; sex: male, female; occurrenceID: EU_LIM_398; **Taxon:** scientificName: Euphylidorea (Euphylidorea) meigenii (Verrall, 1886); family: Limoniidae; genus: Euphylidorea; subgenus: Euphylidorea; specificEpithet: meigenii; scientificNameAuthorship: (Verrall, 1886); **Location:** country: Russia; stateProvince: North European Russia; county: Murmansk region; locality: Murmansk; verbatimElevation: 113 m; minimumElevationInMeters: 113; decimalLatitude: 68.97425; decimalLongitude: 33.13702; **Identification:** identifiedBy: D.I. Gavryushin; **Event:** samplingProtocol: Sweep net; eventDate: 2011-06-21; verbatimEventDate: Jul-21-2011; **Record Level:** institutionCode: ZMMU; basisOfRecord: PreservedSpecimen
- **Type status:** Other material. **Occurrence:** occurrenceRemarks: 1 female; recordedBy: D.I. Gavryushin; individualCount: 1; sex: female; occurrenceID: EU_LIM_399; **Taxon:** scientificName: Euphylidorea (Euphylidorea) meigenii (Verrall, 1886); family: Limoniidae; genus: Euphylidorea; subgenus: Euphylidorea; specificEpithet: meigenii; scientificNameAuthorship: (Verrall, 1886); **Location:** country: Russia; stateProvince: North European Russia; county: Murmansk region; locality: Murmansk, Sredneye Lake; verbatimElevation: 112 m; minimumElevationInMeters: 112; decimalLatitude: 68.97887; decimalLongitude: 33.11513; **Identification:** identifiedBy: D.I. Gavryushin; **Event:** samplingProtocol: Sweep net; eventDate: 2011-06-18; verbatimEventDate: 18./Jul/2011; **Record Level:** institutionCode: ZMMU; basisOfRecord: PreservedSpecimen
- **Type status:** Other material. **Occurrence:** occurrenceRemarks: 1 male, 1 female; recordedBy: D.I. Gavryushin; individualCount: 2; sex: male, female; occurrenceID: EU_LIM_400; **Taxon:** scientificName: Euphylidorea (Euphylidorea) meigenii (Verrall, 1886); family: Limoniidae; genus: Euphylidorea; subgenus: Euphylidorea; specificEpithet: meigenii; scientificNameAuthorship: (Verrall, 1886); **Location:** country: Russia; stateProvince: North European Russia; county: Murmansk region; locality: Murmansk, Bolshoye Lake; verbatimElevation: 64 m; minimumElevationInMeters: 64; decimalLatitude: 68.98117; decimalLongitude: 33.15159; **Identification:** identifiedBy: D.I. Gavryushin; **Event:** samplingProtocol: Sweep net; eventDate: 2011-06-21; verbatimEventDate: 21/Jul/2011; **Record Level:** institutionCode: ZMMU; basisOfRecord: PreservedSpecimen
- **Type status:** Other material. **Occurrence:** occurrenceRemarks: 1 male; recordedBy: A. Polevoi; individualCount: 1; sex: male; preparations: Pinned; occurrenceID: EU_LIM_401; **Taxon:** scientificName: Euphylidorea (Euphylidorea) meigenii (Verrall, 1886); family: Limoniidae; genus: Euphylidorea; subgenus: Euphylidorea; specificEpithet: meigenii; scientificNameAuthorship: (Verrall, 1886); **Location:** country: Russia; stateProvince: North European Russia; county: Republic Karelia; municipality: Prionezhskiy district; locality: Lososinnoe, 5 km NE; verbatimElevation: 200 m; minimumElevationInMeters: 200; decimalLatitude: 61.70767; decimalLongitude: 34.24139; **Identification:** identifiedBy: A. Polevoi; **Event:** samplingProtocol: Sweep net; eventDate: 2013-05-28; verbatimEventDate: 28/May/2013; **Record Level:** institutionCode: FRIP; basisOfRecord: PreservedSpecimen
- **Type status:** Other material. **Occurrence:** occurrenceRemarks: 1 male; recordedBy: A. Polevoi; individualCount: 1; sex: male; preparations: Pinned; occurrenceID: EU_LIM_402; **Taxon:** scientificName: Euphylidorea (Euphylidorea) meigenii (Verrall, 1886); family: Limoniidae; genus: Euphylidorea; subgenus: Euphylidorea; specificEpithet: meigenii; scientificNameAuthorship: (Verrall, 1886); **Location:** country: Russia; stateProvince: North European Russia; county: Republic Karelia; municipality: Muezerskiy district; locality: Murdoyoki River; verbatimElevation: 215 m; minimumElevationInMeters: 215; decimalLatitude: 64.20192; decimalLongitude: 30.86608; **Identification:** identifiedBy: A. Polevoi; **Event:** samplingProtocol: Sweep net; eventDate: 2009-06-07; verbatimEventDate: 07/Jul/2009; **Record Level:** institutionCode: FRIP; basisOfRecord: PreservedSpecimen

#### Distribution

First records from Russia: RUN.

### Eutonia barbipes

(Meigen, 1804)

7FD5A45A-F834-5379-9789-4746CE9AA466

https://ccw.naturalis.nl/detail.php?id=5520

#### Materials

- **Type status:** Other material. **Occurrence:** occurrenceRemarks: 24 males; recordedBy: N.M. Paramonov; individualCount: 24; sex: male; occurrenceID: EU_LIM_403; **Taxon:** scientificName: Eutoniabarbipes (Meigen, 1804); family: Limoniidae; genus: Eutonia; specificEpithet: barbipes; scientificNameAuthorship: (Meigen, 1804); **Location:** country: Russia; stateProvince: East European Russia; county: Tatarstan Respublika; municipality: Laishevo district; locality: Volga-Kama State Nature Biosphere Reserve, «Saraly», Island Ornitologicheskiy; verbatimElevation: 50 m; minimumElevationInMeters: 50; decimalLatitude: 55.28392; decimalLongitude: 49.26081; **Identification:** identifiedBy: N.M. Paramonov; **Event:** samplingProtocol: Sweep net; eventDate: 2009-06-22; verbatimEventDate: 22/Jun/2009; **Record Level:** institutionCode: ZIN; basisOfRecord: PreservedSpecimen
- **Type status:** Other material. **Occurrence:** occurrenceRemarks: 2 males, 1 female; recordedBy: N.M. Paramonov; individualCount: 3; sex: male, female; occurrenceID: EU_LIM_404; **Taxon:** scientificName: Eutoniabarbipes (Meigen, 1804); family: Limoniidae; genus: Eutonia; specificEpithet: barbipes; scientificNameAuthorship: (Meigen, 1804); **Location:** country: Russia; stateProvince: East European Russia; county: Tatarstan Respublika; municipality: Laishevo district; locality: Volga-Kama State Nature Biosphere Reserve, «Saraly»; verbatimElevation: 71 m; minimumElevationInMeters: 71; decimalLatitude: 55.29303; decimalLongitude: 49.29976; **Identification:** identifiedBy: N.M. Paramonov; **Event:** samplingProtocol: Sweep net; eventDate: 2009-06-19; verbatimEventDate: 19/Jun/2009; habitat: wetland; **Record Level:** institutionCode: ZIN; basisOfRecord: PreservedSpecimen
- **Type status:** Other material. **Occurrence:** occurrenceRemarks: 4 males, 1 female; recordedBy: N.M. Paramonov; individualCount: 5; sex: male, female; occurrenceID: EU_LIM_405; **Taxon:** scientificName: Eutoniabarbipes (Meigen, 1804); family: Limoniidae; genus: Eutonia; specificEpithet: barbipes; scientificNameAuthorship: (Meigen, 1804); **Location:** country: Russia; stateProvince: East European Russia; county: Tatarstan Respublika; municipality: Laishevo district; locality: Volga-Kama State Nature Biosphere Reserve, «Saraly»; verbatimElevation: 71 m; minimumElevationInMeters: 71; decimalLatitude: 55.29303; decimalLongitude: 49.29976; **Identification:** identifiedBy: N.M. Paramonov; **Event:** samplingProtocol: Sweep net; eventDate: 2009-06-24; verbatimEventDate: 24/Jun/2009; habitat: wetland; **Record Level:** institutionCode: ZIN; basisOfRecord: PreservedSpecimen
- **Type status:** Other material. **Occurrence:** occurrenceRemarks: 1 female; recordedBy: N.M. Paramonov; individualCount: 1; sex: female; occurrenceID: EU_LIM_406; **Taxon:** scientificName: Eutoniabarbipes (Meigen, 1804); family: Limoniidae; genus: Eutonia; specificEpithet: barbipes; scientificNameAuthorship: (Meigen, 1804); **Location:** country: Russia; stateProvince: East European Russia; county: Tatarstan Respublika; municipality: Zelenodol’sk district; locality: Zaymishche env., Geomagnetic station; verbatimElevation: 87 m; minimumElevationInMeters: 87; decimalLatitude: 55.82684; decimalLongitude: 48.84395; **Identification:** identifiedBy: N.M. Paramonov; **Event:** samplingProtocol: Sweep net; eventDate: 2012-06-30; verbatimEventDate: 30/Jun/2012; **Record Level:** institutionCode: ZIN; basisOfRecord: PreservedSpecimen
- **Type status:** Other material. **Occurrence:** occurrenceRemarks: 1 male; recordedBy: N.M. Paramonov; individualCount: 1; sex: male; occurrenceID: EU_LIM_407; **Taxon:** scientificName: Eutoniabarbipes (Meigen, 1804); family: Limoniidae; genus: Eutonia; specificEpithet: barbipes; scientificNameAuthorship: (Meigen, 1804); **Location:** country: Russia; stateProvince: East European Russia; county: Tatarstan Respublika; municipality: Zelenodol’sk district; locality: Ilinskoe; verbatimElevation: 90 m; minimumElevationInMeters: 90; decimalLatitude: 55.87455; decimalLongitude: 48.68579; **Identification:** identifiedBy: N.M. Paramonov; **Event:** samplingProtocol: Sweep net; eventDate: 2012-06-29; verbatimEventDate: 29/Jun/2012; habitat: village env.; **Record Level:** institutionCode: ZIN; basisOfRecord: PreservedSpecimen
- **Type status:** Other material. **Occurrence:** occurrenceRemarks: 1 male; recordedBy: N.M. Paramonov; individualCount: 1; sex: male; occurrenceID: EU_LIM_408; **Taxon:** scientificName: Eutoniabarbipes (Meigen, 1804); family: Limoniidae; genus: Eutonia; specificEpithet: barbipes; scientificNameAuthorship: (Meigen, 1804); **Location:** country: Russia; stateProvince: East European Russia; county: Tatarstan Respublika; municipality: Zelenodol’sk district; locality: Volga-Kama State Nature Biosphere Reserve, «Raifa», Lake Lenevo; verbatimElevation: 80 m; minimumElevationInMeters: 80; decimalLatitude: 55.90433; decimalLongitude: 48.79115; **Identification:** identifiedBy: N.M. Paramonov; **Event:** samplingProtocol: Sweep net; eventDate: 2012-06-26; verbatimEventDate: 26/Jun/2012; **Record Level:** institutionCode: ZIN; basisOfRecord: PreservedSpecimen

#### Distribution

First records from Russia: RUE.

### Geranomyia inornata

Lackschewitz, 1928

BAF56428-4C75-5B4D-88E2-EE2851A2B4B7

https://ccw.naturalis.nl/detail.php?id=9676

#### Materials

- **Type status:** Other material. **Occurrence:** occurrenceRemarks: 2 males; recordedBy: J. Starý; individualCount: 2; sex: male; preparations: Pinned; occurrenceID: EU_LIM_409; **Taxon:** scientificName: Geranomyiainornata Lackschewitz, 1928; family: Limoniidae; genus: Geranomyia; specificEpithet: inornata; scientificNameAuthorship: Lackschewitz, 1928; **Location:** country: Greece; stateProvince: Peloponnese; municipality: Araxos; locality: 1.1 km N, Araxos lagoon; verbatimElevation: 1 m; minimumElevationInMeters: 1; decimalLatitude: 38.183; decimalLongitude: 21.4; **Identification:** identifiedBy: J. Starý; **Event:** eventDate: 2015-05-28; verbatimEventDate: 28/May/2015; habitat: saltmarsh; **Record Level:** institutionCode: PCJS; basisOfRecord: PreservedSpecimen
- **Type status:** Other material. **Occurrence:** occurrenceRemarks: 6 males; recordedBy: J. Starý; individualCount: 6; sex: male; preparations: Pinned; occurrenceID: EU_LIM_410; **Taxon:** scientificName: Geranomyiainornata Lackschewitz, 1928; family: Limoniidae; genus: Geranomyia; specificEpithet: inornata; scientificNameAuthorship: Lackschewitz, 1928; **Location:** country: Greece; stateProvince: Peloponnese; municipality: Araxos; locality: 1.1 km N, Araxos lagoon; verbatimElevation: 1 m; minimumElevationInMeters: 1; decimalLatitude: 38.183; decimalLongitude: 21.4; **Identification:** identifiedBy: J. Starý; **Event:** eventDate: 2015-05-30; verbatimEventDate: 30/May/2015; habitat: saltmarsh; **Record Level:** institutionCode: PCJS; basisOfRecord: PreservedSpecimen

#### Distribution

First records from Greece (from mainland).

### Geranomyia unicolor

Haliday, 1833

FB59A285-62FF-5085-AD4C-ED33D3FFC29A

https://ccw.naturalis.nl/detail.php?id=9867

#### Materials

- **Type status:** Other material. **Occurrence:** occurrenceRemarks: 2 males, 1 female; recordedBy: E. Eiroa; individualCount: 3; sex: male, female; preparations: Pinned; occurrenceID: EU_LIM_411; **Taxon:** scientificName: Geranomyiaunicolor Haliday, 1833; family: Limoniidae; genus: Geranomyia; specificEpithet: unicolor; scientificNameAuthorship: Haliday, 1833; **Location:** country: Spain; stateProvince: Galicia, Lugo; municipality: Barreiros; locality: Playa de Barreiros beach; verbatimElevation: 7 m; minimumElevationInMeters: 7; decimalLatitude: 43.56272; decimalLongitude: -7.20722; **Identification:** identifiedBy: E. Eiroa; **Event:** samplingProtocol: Sweep net; eventDate: 1992-08-13; verbatimEventDate: 13/August/1992; habitat: rocky seashore; **Record Level:** institutionCode: USC; basisOfRecord: PreservedSpecimen

#### Distribution

First record from mainland Spain, previously reported from Canary Islands.

### Gnophomyia viridipennis

(Gimmerthal, 1847)

02CB345F-06D3-5BB2-B609-02C6587FFD5A

https://ccw.naturalis.nl/detail.php?id=1434

#### Materials

- **Type status:** Other material. **Occurrence:** occurrenceRemarks: 1 male; recordedBy: D.I. Gavryushin; individualCount: 1; sex: male; preparations: Pinned; occurrenceID: EU_LIM_412; **Taxon:** scientificName: Gnophomyiaviridipennis (Gimmerthal, 1847); family: Limoniidae; genus: Gnophomyia; specificEpithet: viridipennis; scientificNameAuthorship: (Gimmerthal, 1847); **Location:** country: Serbia; stateProvince: Zaječar; municipality: Knjaževac; locality: Crni Vrh; verbatimElevation: 800 m; minimumElevationInMeters: 800; decimalLatitude: 43.407; decimalLongitude: 22.587; **Identification:** identifiedBy: D.I. Gavryushin; **Event:** samplingProtocol: Sweep net; eventDate: 2015-06-01/2015-07-07; verbatimEventDate: 01-07/Jul/2015; **Record Level:** institutionCode: ZMMU; basisOfRecord: PreservedSpecimen

#### Distribution

First record from Serbia.

### Gonempeda flava

(Schummel, 1829)

1902F74B-B89C-558D-997C-896D758233D0

https://ccw.naturalis.nl/detail.php?id=1439

#### Materials

- **Type status:** Other material. **Occurrence:** occurrenceRemarks: 1 female; recordedBy: D.I. Gavryushin; individualCount: 1; sex: female; preparations: Pinned; occurrenceID: EU_LIM_413; **Taxon:** scientificName: Gonempedaflava (Schummel, 1829); family: Limoniidae; genus: Gonempeda; specificEpithet: flava; scientificNameAuthorship: (Schummel, 1829); **Location:** country: Belarus; stateProvince: Vitebsk; county: Orsha; locality: Orsha; decimalLatitude: 54.555; decimalLongitude: 30.63; **Identification:** identifiedBy: D.I. Gavryushin; **Event:** samplingProtocol: Sweep net; eventDate: 2019-06-09/2019-06-10; verbatimEventDate: 9-10/Jun/2019; **Record Level:** institutionCode: ZMMU; basisOfRecord: PreservedSpecimen
- **Type status:** Other material. **Occurrence:** occurrenceRemarks: 1 male; recordedBy: L.-P. Kolcsár; individualCount: 1; sex: male; preparations: Ethanol; occurrenceID: EU_LIM_414; **Taxon:** scientificName: Gonempedaflava (Schummel, 1829); family: Limoniidae; genus: Gonempeda; specificEpithet: flava; scientificNameAuthorship: (Schummel, 1829); **Location:** country: Montenegro; municipality: Zelenika; locality: Kotor Bay; verbatimElevation: 22 m; minimumElevationInMeters: 22; decimalLatitude: 42.45388; decimalLongitude: 18.56961; **Identification:** identifiedBy: L.-P. Kolcsár; **Event:** samplingProtocol: Sweep net; eventDate: 2010-05-14; verbatimEventDate: 14/May/2010; habitat: oak forest; **Record Level:** institutionCode: CKLP; basisOfRecord: PreservedSpecimen
- **Type status:** Other material. **Occurrence:** occurrenceRemarks: 1 male; recordedBy: L.-P. Kolcsár | E. Török; individualCount: 1; sex: male; preparations: Ethanol; occurrenceID: EU_LIM_415; **Taxon:** scientificName: Gonempedaflava (Schummel, 1829); family: Limoniidae; genus: Gonempeda; specificEpithet: flava; scientificNameAuthorship: (Schummel, 1829); **Location:** country: North Macedonia; municipality: Izvor; locality: Treska River; verbatimElevation: 755 m; minimumElevationInMeters: 755; decimalLatitude: 41.48021; decimalLongitude: 20.83466; **Identification:** identifiedBy: L.-P. Kolcsár; **Event:** samplingProtocol: Sweep net; eventDate: 2017-06-29; verbatimEventDate: 29/Jun/2017; **Record Level:** institutionCode: CKLP; basisOfRecord: PreservedSpecimen
- **Type status:** Other material. **Occurrence:** occurrenceRemarks: 1 male; recordedBy: L.-P. Kolcsár; individualCount: 1; sex: male; preparations: Ethanol; occurrenceID: EU_LIM_416; **Taxon:** scientificName: Gonomyia (Gonomyia) abscondita Lackschewitz, 1935; family: Limoniidae; genus: Gonomyia; subgenus: Gonomyia; specificEpithet: abscondita; scientificNameAuthorship: Lackschewitz, 1935; **Location:** country: Latvia; municipality: Sigulda; locality: Gauja River; verbatimElevation: 13 m; minimumElevationInMeters: 13; decimalLatitude: 57.1505; decimalLongitude: 24.8168; **Identification:** identifiedBy: L.-P. Kolcsár; **Event:** samplingProtocol: Sweep net; eventDate: 2018-06-24; verbatimEventDate: Jul-24-2018; **Record Level:** institutionCode: CKLP; basisOfRecord: PreservedSpecimen
- **Type status:** Other material. **Occurrence:** occurrenceRemarks: 1 male; recordedBy: D.I. Gavryushin; individualCount: 1; sex: male; occurrenceID: EU_LIM_417; **Taxon:** scientificName: Gonomyia (Gonomyia) abscondita Lackschewitz, 1935; family: Limoniidae; genus: Gonomyia; subgenus: Gonomyia; specificEpithet: abscondita; scientificNameAuthorship: Lackschewitz, 1935; **Location:** country: Russia; stateProvince: East European Russia; county: Bashkortostan Respublika; municipality: Uchaly district; locality: Ural-Tau St. env.; verbatimElevation: 777 m; minimumElevationInMeters: 777; decimalLatitude: 53.96805; decimalLongitude: 58.57613; **Identification:** identifiedBy: D.I. Gavryushin; **Event:** samplingProtocol: Sweep net; eventDate: 2015-06-09; verbatimEventDate: 09/Jul/2015; **Record Level:** institutionCode: ZMMU; basisOfRecord: PreservedSpecimen
- **Type status:** Other material. **Occurrence:** occurrenceRemarks: 2 male, 1 female; recordedBy: D.I. Gavryushin; individualCount: 3; sex: male, female; occurrenceID: EU_LIM_418; **Taxon:** scientificName: Gonomyia (Gonomyia) abscondita Lackschewitz, 1935; family: Limoniidae; genus: Gonomyia; subgenus: Gonomyia; specificEpithet: abscondita; scientificNameAuthorship: Lackschewitz, 1935; **Location:** country: Russia; stateProvince: East European Russia; county: Bashkortostan Respublika; municipality: Beloretsk district; locality: Nura River (ca. 4km W of Otnurok village), at the foot of Zolotyie Shishki (Golden Cones) Mts.; verbatimElevation: 607 m; minimumElevationInMeters: 607; decimalLatitude: 54.05155; decimalLongitude: 58.26887; **Identification:** identifiedBy: D.I. Gavryushin; **Event:** samplingProtocol: Sweep net; eventDate: 2015-06-13; verbatimEventDate: 13/Jul/2015; **Record Level:** institutionCode: ZMMU; basisOfRecord: PreservedSpecimen
- **Type status:** Other material. **Occurrence:** occurrenceRemarks: 1 male; recordedBy: A. Polevoi; individualCount: 1; sex: male; preparations: Pinned; occurrenceID: EU_LIM_419; **Taxon:** scientificName: Gonomyia (Gonomyia) abscondita Lackschewitz, 1935; family: Limoniidae; genus: Gonomyia; subgenus: Gonomyia; specificEpithet: abscondita; scientificNameAuthorship: Lackschewitz, 1935; **Location:** country: Russia; stateProvince: North European Russia; county: Republic Karelia; municipality: Suojarvi district; locality: Ar'koila, 1.5 km NE; verbatimElevation: 163 m; minimumElevationInMeters: 163; decimalLatitude: 61.93518; decimalLongitude: 32.84214; **Identification:** identifiedBy: A. Polevoi; **Event:** samplingProtocol: Sweep net; eventDate: 2018-06-20; verbatimEventDate: 20/Jun/2018; **Record Level:** institutionCode: FRIP; basisOfRecord: PreservedSpecimen

#### Distribution

First records from Belarus, Montenegro and North Macedonia.

### Gonomyia (Gonomyia) abscondita

Lackschewitz, 1935

0BCAB52B-E095-5386-BDED-A4C83821CB31

https://ccw.naturalis.nl/detail.php?id=1447

#### Materials

- **Type status:** Other material. **Occurrence:** occurrenceRemarks: 1 male; recordedBy: L.-P. Kolcsár; individualCount: 1; sex: male; preparations: Ethanol; occurrenceID: EU_LIM_416; **Taxon:** scientificName: Gonomyia (Gonomyia) abscondita Lackschewitz, 1935; family: Limoniidae; genus: Gonomyia; subgenus: Gonomyia; specificEpithet: abscondita; scientificNameAuthorship: Lackschewitz, 1935; **Location:** country: Latvia; municipality: Sigulda; locality: Gauja River; verbatimElevation: 13 m; minimumElevationInMeters: 13; decimalLatitude: 57.1505; decimalLongitude: 24.8168; **Identification:** identifiedBy: L.-P. Kolcsár; **Event:** samplingProtocol: Sweep net; eventDate: 2018-06-24; verbatimEventDate: Jul-24-2018; **Record Level:** institutionCode: CKLP; basisOfRecord: PreservedSpecimen
- **Type status:** Other material. **Occurrence:** occurrenceRemarks: 1 male; recordedBy: D.I. Gavryushin; individualCount: 1; sex: male; occurrenceID: EU_LIM_417; **Taxon:** scientificName: Gonomyia (Gonomyia) abscondita Lackschewitz, 1935; family: Limoniidae; genus: Gonomyia; subgenus: Gonomyia; specificEpithet: abscondita; scientificNameAuthorship: Lackschewitz, 1935; **Location:** country: Russia; stateProvince: East European Russia; county: Bashkortostan Respublika; municipality: Uchaly district; locality: Ural-Tau St. env.; verbatimElevation: 777 m; minimumElevationInMeters: 777; decimalLatitude: 53.96805; decimalLongitude: 58.57613; **Identification:** identifiedBy: D.I. Gavryushin; **Event:** samplingProtocol: Sweep net; eventDate: 2015-06-09; verbatimEventDate: 09/Jul/2015; **Record Level:** institutionCode: ZMMU; basisOfRecord: PreservedSpecimen
- **Type status:** Other material. **Occurrence:** occurrenceRemarks: 2 male, 1 female; recordedBy: D.I. Gavryushin; individualCount: 3; sex: male, female; occurrenceID: EU_LIM_418; **Taxon:** scientificName: Gonomyia (Gonomyia) abscondita Lackschewitz, 1935; family: Limoniidae; genus: Gonomyia; subgenus: Gonomyia; specificEpithet: abscondita; scientificNameAuthorship: Lackschewitz, 1935; **Location:** country: Russia; stateProvince: East European Russia; county: Bashkortostan Respublika; municipality: Beloretsk district; locality: Nura River (ca. 4km W of Otnurok village), at the foot of Zolotyie Shishki (Golden Cones) Mts.; verbatimElevation: 607 m; minimumElevationInMeters: 607; decimalLatitude: 54.05155; decimalLongitude: 58.26887; **Identification:** identifiedBy: D.I. Gavryushin; **Event:** samplingProtocol: Sweep net; eventDate: 2015-06-13; verbatimEventDate: 13/Jul/2015; **Record Level:** institutionCode: ZMMU; basisOfRecord: PreservedSpecimen
- **Type status:** Other material. **Occurrence:** occurrenceRemarks: 1 male; recordedBy: A. Polevoi; individualCount: 1; sex: male; preparations: Pinned; occurrenceID: EU_LIM_419; **Taxon:** scientificName: Gonomyia (Gonomyia) abscondita Lackschewitz, 1935; family: Limoniidae; genus: Gonomyia; subgenus: Gonomyia; specificEpithet: abscondita; scientificNameAuthorship: Lackschewitz, 1935; **Location:** country: Russia; stateProvince: North European Russia; county: Republic Karelia; municipality: Suojarvi district; locality: Ar'koila, 1.5 km NE; verbatimElevation: 163 m; minimumElevationInMeters: 163; decimalLatitude: 61.93518; decimalLongitude: 32.84214; **Identification:** identifiedBy: A. Polevoi; **Event:** samplingProtocol: Sweep net; eventDate: 2018-06-20; verbatimEventDate: 20/Jun/2018; **Record Level:** institutionCode: FRIP; basisOfRecord: PreservedSpecimen

#### Distribution

First records from Latvia and Russia: RUE, RUN.

### Gonomyia (Gonomyia) bifida

Tonnoir, 1920

C66202CC-A2FE-5742-91A1-D13D805881A9

https://ccw.naturalis.nl/detail.php?id=1465

#### Materials

- **Type status:** Other material. **Occurrence:** occurrenceRemarks: 1 male; recordedBy: D.I. Gavryushin; individualCount: 1; sex: male; preparations: Pinned; occurrenceID: EU_LIM_420; **Taxon:** scientificName: Gonomyia (Gonomyia) bifida Tonnoir, 1920; family: Limoniidae; genus: Gonomyia; subgenus: Gonomyia; specificEpithet: bifida; scientificNameAuthorship: Tonnoir, 1920; **Location:** country: Belarus; stateProvince: Minsk; county: Barysaw; locality: Barysaw; verbatimElevation: 155 m; minimumElevationInMeters: 155; decimalLatitude: 54.25542; decimalLongitude: 28.48092; **Identification:** identifiedBy: D.I. Gavryushin; **Event:** samplingProtocol: Sweep net; eventDate: 2013-06-05; verbatimEventDate: Jul-05-2013; **Record Level:** institutionCode: ZMMU; basisOfRecord: PreservedSpecimen
- **Type status:** Other material. **Occurrence:** occurrenceRemarks: 1 male; recordedBy: L.-P. Kolcsár; individualCount: 1; sex: male; preparations: Ethanol; occurrenceID: EU_LIM_421; **Taxon:** scientificName: Gonomyia (Gonomyia) bifida Tonnoir, 1920; family: Limoniidae; genus: Gonomyia; subgenus: Gonomyia; specificEpithet: bifida; scientificNameAuthorship: Tonnoir, 1920; **Location:** country: Romania; stateProvince: Harghita; municipality: Voșlăbeni; locality: Senetea; verbatimElevation: 764 m; minimumElevationInMeters: 764; decimalLatitude: 46.62588; decimalLongitude: 25.59745; **Identification:** identifiedBy: L.-P. Kolcsár; **Event:** samplingProtocol: Sweep net; eventDate: 2016-10-01; verbatimEventDate: 01/Oct/2016; habitat: marshy meadow; **Record Level:** institutionCode: CKLP; basisOfRecord: PreservedSpecimen

#### Distribution

First records from Belarus and Romania.

### Gonomyia (Gonomyia) conoviensis

Barnes, 1924

AB524EF4-CD45-5755-B44D-AB7DBC209221

https://ccw.naturalis.nl/detail.php?id=18545

#### Materials

- **Type status:** Other material. **Occurrence:** occurrenceRemarks: 1 male; recordedBy: L.-P. Kolcsár; individualCount: 1; sex: male; preparations: Ethanol; occurrenceID: EU_LIM_422; **Taxon:** scientificName: Gonomyia (Gonomyia) conoviensis Barnes, 1924; family: Limoniidae; genus: Gonomyia; subgenus: Gonomyia; specificEpithet: conoviensis; scientificNameAuthorship: Barnes, 1924; **Location:** country: Montenegro; municipality: Bjelos; locality: Lovćen Mts.; verbatimElevation: 950 m; minimumElevationInMeters: 950; decimalLatitude: 42.36701; decimalLongitude: 18.89107; **Identification:** identifiedBy: L.-P. Kolcsár; **Event:** samplingProtocol: Sweep net; eventDate: 2014-05-03; verbatimEventDate: May-03-2014; **Record Level:** institutionCode: CKLP; basisOfRecord: PreservedSpecimen
- **Type status:** Other material. **Occurrence:** occurrenceRemarks: 1 male; recordedBy: V.I. Lantsov; individualCount: 1; sex: male; preparations: Pinned; occurrenceID: EU_LIM_423; **Taxon:** scientificName: Gonomyia (Gonomyia) conoviensis Barnes, 1924; family: Limoniidae; genus: Gonomyia; subgenus: Gonomyia; specificEpithet: conoviensis; scientificNameAuthorship: Barnes, 1924; **Location:** country: Russia; stateProvince: North Caucasus; county: Karachay-Cherkess Republic; municipality: Kurdzhinovo; locality: Caucasian State Natural Biosphere Reserve. In 45-50- km SW from Kurdzhinovo. at the foot of Mount Zakan (ridge Magisho), River Valley Zakan, basin of Bolshaya Laba; verbatimElevation: 1342 m; minimumElevationInMeters: 1342; decimalLatitude: 43.73278; decimalLongitude: 40.79444; **Identification:** identifiedBy: V.I. Lantsov; **Event:** samplingProtocol: Light trap; eventDate: 2014-06-21; verbatimEventDate: 21/Jun/2014; habitat: Terrace above the floodplain, in the bend of the Zakan and Imeritinka rivers; **Record Level:** institutionCode: ZIN; basisOfRecord: PreservedSpecimen

#### Distribution

First records from Montenegro and Russia: NC.

### Gonomyia (Gonomyia) dentata

de Meijere, 1920

01BA4CC1-D254-55A5-91E7-82F8FBE75872

https://ccw.naturalis.nl/detail.php?id=1491

#### Materials

- **Type status:** Other material. **Occurrence:** occurrenceRemarks: 1 male; recordedBy: E. Eiroa; individualCount: 1; sex: male; preparations: Pinned; occurrenceID: EU_LIM_424; **Taxon:** scientificName: Gonomyia (Gonomyia) dentata de Meijere, 1920; family: Limoniidae; genus: Gonomyia; subgenus: Gonomyia; specificEpithet: dentata; scientificNameAuthorship: de Meijere, 1920; **Location:** country: Portugal; stateProvince: Guarda; municipality: Manteigas; locality: Fonte da Jonja, serra da Estrela; verbatimElevation: 1360 m; minimumElevationInMeters: 1360; decimalLatitude: 40.32452; decimalLongitude: -7.57769; **Identification:** identifiedBy: E. Eiroa; **Event:** samplingProtocol: Sweep net; eventDate: 1992-05-30; verbatimEventDate: 30/May/1992; **Record Level:** institutionCode: USC; basisOfRecord: PreservedSpecimen
- **Type status:** Other material. **Occurrence:** occurrenceRemarks: 1 female; recordedBy: E. Eiroa; individualCount: 1; sex: female; preparations: Pinned; occurrenceID: EU_LIM_425; **Taxon:** scientificName: Gonomyia (Gonomyia) dentata de Meijere, 1920; family: Limoniidae; genus: Gonomyia; subgenus: Gonomyia; specificEpithet: dentata; scientificNameAuthorship: de Meijere, 1920; **Location:** country: Portugal; stateProvince: Guarda; municipality: Manteigas; locality: Vale do Zézere, serra da Estrela; verbatimElevation: 1240 m; minimumElevationInMeters: 1240; decimalLatitude: 40.32683; decimalLongitude: -7.57641; **Identification:** identifiedBy: E. Eiroa; **Event:** samplingProtocol: Sweep net; eventDate: 1992-05-30; verbatimEventDate: 30/May/1992; habitat: riverside vegetation; **Record Level:** institutionCode: USC; basisOfRecord: PreservedSpecimen

#### Distribution

First records from Portugal.

### Gonomyia (Gonomyia) recta

Tonnoir, 1920

4818E24F-CA8D-5688-BC0E-33C09818E6F6

https://ccw.naturalis.nl/detail.php?id=1597

#### Materials

- **Type status:** Other material. **Occurrence:** occurrenceRemarks: 1 male; recordedBy: D.I. Gavryushin; individualCount: 1; sex: male; preparations: Pinned; occurrenceID: EU_LIM_426; **Taxon:** scientificName: Gonomyia (Gonomyia) recta Tonnoir, 1920; family: Limoniidae; genus: Gonomyia; subgenus: Gonomyia; specificEpithet: recta; scientificNameAuthorship: Tonnoir, 1920; **Location:** country: Serbia; stateProvince: Zaječar; municipality: Knjaževac; locality: Crni Vrh; verbatimElevation: 800 m; minimumElevationInMeters: 800; decimalLatitude: 43.407; decimalLongitude: 22.587; **Identification:** identifiedBy: D.I. Gavryushin; **Event:** samplingProtocol: Sweep net; eventDate: 2015-06-01/2015-07-07; verbatimEventDate: 01-07/Jul/2015; **Record Level:** institutionCode: ZMMU; basisOfRecord: PreservedSpecimen

#### Distribution

First record from Serbia.

### Gonomyia (Gonomyia) subtenella

Savchenko, 1972

A943ECE6-EABC-51B3-8E1B-0750B51B7667

https://ccw.naturalis.nl/detail.php?id=1630

#### Materials

- **Type status:** Other material. **Occurrence:** occurrenceRemarks: 1 male; recordedBy: D.I. Gavryushin; individualCount: 1; sex: male; preparations: Pinned; occurrenceID: EU_LIM_427; **Taxon:** scientificName: Gonomyia (Gonomyia) subtenella Savchenko, 1972; family: Limoniidae; genus: Gonomyia; subgenus: Gonomyia; specificEpithet: subtenella; scientificNameAuthorship: Savchenko, 1972; **Location:** country: Serbia; stateProvince: Zaječar; municipality: Knjaževac; locality: Knjaževac; decimalLatitude: 43.55; decimalLongitude: 22.24; **Identification:** identifiedBy: D.I. Gavryushin; **Event:** samplingProtocol: Sweep net; eventDate: 2015-04-27/2015-04-30; verbatimEventDate: 27-30/Apr/2015; **Record Level:** institutionCode: ZMMU; basisOfRecord: PreservedSpecimen

#### Distribution

First record from Serbia.

### Gonomyia (Gonomyia) tenella

(Meigen, 1818)

76CDBE3E-4430-519C-B0BC-7EE4CB5FF031

https://ccw.naturalis.nl/detail.php?id=1639

#### Materials

- **Type status:** Other material. **Occurrence:** occurrenceRemarks: 1 male; recordedBy: L.-P. Kolcsár | E. Török; individualCount: 1; sex: male; preparations: Ethanol; occurrenceID: EU_LIM_428; **Taxon:** scientificName: Gonomyia (Gonomyia) tenella (Meigen, 1818); family: Limoniidae; genus: Gonomyia; subgenus: Gonomyia; specificEpithet: tenella; scientificNameAuthorship: (Meigen, 1818); **Location:** country: Albania; stateProvince: Korçë; municipality: Buqezë; locality: Ohrid Lake; verbatimElevation: 696 m; minimumElevationInMeters: 696; decimalLatitude: 41.04104; decimalLongitude: 20.63448; **Identification:** identifiedBy: L.-P. Kolcsár; **Event:** samplingProtocol: Sweep net; eventDate: 2017-06-01; verbatimEventDate: 1/Jul/2017; **Record Level:** institutionCode: CKLP; basisOfRecord: PreservedSpecimen
- **Type status:** Other material. **Occurrence:** occurrenceRemarks: 1 male; recordedBy: L.-P. Kolcsár | E. Török; individualCount: 1; sex: male; preparations: Ethanol; occurrenceID: EU_LIM_429; **Taxon:** scientificName: Gonomyia (Gonomyia) tenella (Meigen, 1818); family: Limoniidae; genus: Gonomyia; subgenus: Gonomyia; specificEpithet: tenella; scientificNameAuthorship: (Meigen, 1818); **Location:** country: Serbia; municipality: Banatska Palanka; locality: Kanal DTD; verbatimElevation: 67 m; minimumElevationInMeters: 67; decimalLatitude: 44.85833; decimalLongitude: 21.30525; **Identification:** identifiedBy: L.-P. Kolcsár; **Event:** samplingProtocol: Sweep net; eventDate: 2017-04-30; verbatimEventDate: 30/Apr/2017; **Record Level:** institutionCode: CKLP; basisOfRecord: PreservedSpecimen

#### Distribution

First records from Albania and Serbia.

### Helius (Helius) calviensis

Edwards, 1928

80A0954E-B5F7-5D86-9B57-98C3A76F06E4

https://ccw.naturalis.nl/detail.php?id=9950

#### Materials

- **Type status:** Other material. **Occurrence:** occurrenceRemarks: 2 males; recordedBy: L.-P. Kolcsár; individualCount: 2; sex: male; preparations: Ethanol; occurrenceID: EU_LIM_430; **Taxon:** scientificName: Helius (Helius) calviensis Edwards, 1928; family: Limoniidae; genus: Helius; subgenus: Helius; specificEpithet: calviensis; scientificNameAuthorship: Edwards, 1928; **Location:** country: Albania; stateProvince: Vlorë; municipality: Butrint; locality: Butrint Lake; verbatimElevation: 12 m; minimumElevationInMeters: 12; decimalLatitude: 39.74623; decimalLongitude: 20.01736; **Identification:** identifiedBy: L.-P. Kolcsár; **Event:** samplingProtocol: Sweep net; eventDate: 2016-05-03; verbatimEventDate: 3/May/2016; **Record Level:** institutionCode: CKLP; basisOfRecord: PreservedSpecimen
- **Type status:** Other material. **Occurrence:** occurrenceRemarks: 1 male, 1 female; recordedBy: L.-P. Kolcsár; individualCount: 2; sex: male, female; preparations: Ethanol; occurrenceID: EU_LIM_431; **Taxon:** scientificName: Helius (Helius) calviensis Edwards, 1928; family: Limoniidae; genus: Helius; subgenus: Helius; specificEpithet: calviensis; scientificNameAuthorship: Edwards, 1928; **Location:** country: Greece; stateProvince: Eastern Macedonia and Thrace; municipality: Chrysoupoli; locality: Nea Karya, Nestos River and Delta; verbatimElevation: 10 m; minimumElevationInMeters: 10; decimalLatitude: 40.87841; decimalLongitude: 24.78541; **Identification:** identifiedBy: L.-P. Kolcsár; **Event:** samplingProtocol: Sweep net; eventDate: 2011-05-06; verbatimEventDate: 6/May/2011; **Record Level:** institutionCode: CKLP; basisOfRecord: PreservedSpecimen
- **Type status:** Other material. **Occurrence:** occurrenceRemarks: 1 male, 1 female; recordedBy: L.-P. Kolcsár; individualCount: 2; sex: male, female; preparations: Ethanol; occurrenceID: EU_LIM_432; **Taxon:** scientificName: Helius (Helius) calviensis Edwards, 1928; family: Limoniidae; genus: Helius; subgenus: Helius; specificEpithet: calviensis; scientificNameAuthorship: Edwards, 1928; **Location:** country: Montenegro; municipality: Zelenika; locality: Kotor Bay; verbatimElevation: 20 m; minimumElevationInMeters: 20; decimalLatitude: 42.45388; decimalLongitude: 18.56961; **Identification:** identifiedBy: L.-P. Kolcsár; **Event:** samplingProtocol: Sweep net; eventDate: 2010-05-14; verbatimEventDate: 14/May/2010; **Record Level:** institutionCode: CKLP; basisOfRecord: PreservedSpecimen
- **Type status:** Other material. **Occurrence:** occurrenceRemarks: 1 males; recordedBy: L.-P. Kolcsár; individualCount: 1; sex: male; preparations: Ethanol; occurrenceID: EU_LIM_433; **Taxon:** scientificName: Helius (Helius) calviensis Edwards, 1928; family: Limoniidae; genus: Helius; subgenus: Helius; specificEpithet: calviensis; scientificNameAuthorship: Edwards, 1928; **Location:** country: Romania; stateProvince: Tulcea; municipality: Crișan; locality: Danube Delta, Sulina Branch of Dunabe; verbatimElevation: 3 m; minimumElevationInMeters: 3; decimalLatitude: 45.17491; decimalLongitude: 29.35658; **Identification:** identifiedBy: L.-P. Kolcsár; **Event:** samplingProtocol: Sweep net; eventDate: 2010-06-18; verbatimEventDate: 18/Jul/2010; **Record Level:** institutionCode: CKLP; basisOfRecord: PreservedSpecimen

#### Distribution

First records from Albania, Greece (from mainland), Montenegro and Romania.

### Helius (Helius) flavus

(Walker, 1856)

00911F66-1AA3-5B91-9DB2-F2A3ABEFBFF1

https://ccw.naturalis.nl/detail.php?id=9981

#### Materials

- **Type status:** Other material. **Occurrence:** occurrenceRemarks: 1 male; recordedBy: D.I. Gavryushin; individualCount: 1; sex: male; preparations: Pinned; occurrenceID: EU_LIM_434; **Taxon:** scientificName: Helius (Helius) flavus (Walker, 1856); family: Limoniidae; genus: Helius; subgenus: Helius; specificEpithet: flavus; scientificNameAuthorship: (Walker, 1856); **Location:** country: Belarus; stateProvince: Minsk; county: Barysaw; locality: Glivin; verbatimElevation: 161 m; minimumElevationInMeters: 161; decimalLatitude: 54.14902; decimalLongitude: 28.63648; **Identification:** identifiedBy: D.I. Gavryushin; **Event:** samplingProtocol: Sweep net; eventDate: 2013-06-06; verbatimEventDate: Jul-06-2013; **Record Level:** institutionCode: ZMMU; basisOfRecord: PreservedSpecimen
- **Type status:** Other material. **Occurrence:** occurrenceRemarks: 3 males, 1 female; recordedBy: D.I. Gavryushin; individualCount: 4; sex: male, female; preparations: Pinned; occurrenceID: EU_LIM_435; **Taxon:** scientificName: Helius (Helius) flavus (Walker, 1856); family: Limoniidae; genus: Helius; subgenus: Helius; specificEpithet: flavus; scientificNameAuthorship: (Walker, 1856); **Location:** country: Belarus; stateProvince: Minsk; county: Barysaw; locality: Vialikaje Stachava; verbatimElevation: 156 m; minimumElevationInMeters: 156; decimalLatitude: 54.26555; decimalLongitude: 28.38332; **Identification:** identifiedBy: D.I. Gavryushin; **Event:** samplingProtocol: Sweep net; eventDate: 2013-06-07; verbatimEventDate: 7/Jul/2013; **Record Level:** institutionCode: ZMMU; basisOfRecord: PreservedSpecimen
- **Type status:** Other material. **Occurrence:** occurrenceRemarks: 1 male, 1 female; recordedBy: K. Peeters; individualCount: 2; sex: male, female; occurrenceID: EU_LIM_436; **Taxon:** scientificName: Helius (Helius) flavus (Walker, 1856); family: Limoniidae; genus: Helius; subgenus: Helius; specificEpithet: flavus; scientificNameAuthorship: (Walker, 1856); **Location:** country: Belgium; stateProvince: Antwerpen; municipality: Zwijndrecht; locality: Vlietbos; decimalLatitude: 51.22; decimalLongitude: 4.34; **Identification:** identifiedBy: P. Oosterbroek; **Event:** eventDate: 2013-06-04; verbatimEventDate: 04/Jul/2013; **Record Level:** basisOfRecord: HumanObservation
- **Type status:** Other material. **Occurrence:** occurrenceRemarks: 1 female; recordedBy: W. Vercruysse; individualCount: 1; sex: female; occurrenceID: EU_LIM_437; **Taxon:** scientificName: Helius (Helius) flavus (Walker, 1856); family: Limoniidae; genus: Helius; subgenus: Helius; specificEpithet: flavus; scientificNameAuthorship: (Walker, 1856); **Location:** country: Belgium; stateProvince: Oost-Vlaanderen; municipality: Destelbergen; decimalLatitude: 51.05; decimalLongitude: 3.79; **Identification:** identifiedBy: P. Oosterbroek; **Event:** eventDate: 2013-06-05; verbatimEventDate: 05/Jul/2013; **Record Level:** basisOfRecord: HumanObservation
- **Type status:** Other material. **Occurrence:** occurrenceRemarks: 1 female; recordedBy: J. Menten; individualCount: 1; sex: female; occurrenceID: EU_LIM_438; **Taxon:** scientificName: Helius (Helius) flavus (Walker, 1856); family: Limoniidae; genus: Helius; subgenus: Helius; specificEpithet: flavus; scientificNameAuthorship: (Walker, 1856); **Location:** country: Belgium; stateProvince: Vlaams-Brabant; locality: Meerdaalwoud; decimalLatitude: 50.8; decimalLongitude: 4.67; **Identification:** identifiedBy: P. Oosterbroek; **Event:** eventDate: 2012-06-30; verbatimEventDate: 30/Jun/2012; **Record Level:** basisOfRecord: HumanObservation
- **Type status:** Other material. **Occurrence:** occurrenceRemarks: 1 male; recordedBy: N.M. Paramonov; individualCount: 1; sex: male; occurrenceID: EU_LIM_439; **Taxon:** scientificName: Helius (Helius) flavus (Walker, 1856); family: Limoniidae; genus: Helius; subgenus: Helius; specificEpithet: flavus; scientificNameAuthorship: (Walker, 1856); **Location:** country: Russia; stateProvince: East European Russia; county: Tatarstan Respublika; municipality: Zelenodol’sk district; locality: Zaymishche env., Geomagnetic station; verbatimElevation: 87 m; minimumElevationInMeters: 87; decimalLatitude: 55.82684; decimalLongitude: 48.84395; **Identification:** identifiedBy: N.M. Paramonov; **Event:** samplingProtocol: Sweep net; eventDate: 2010-06-17; verbatimEventDate: 17/Jun/2010; **Record Level:** institutionCode: ZIN; basisOfRecord: PreservedSpecimen
- **Type status:** Other material. **Occurrence:** occurrenceRemarks: 1 male; recordedBy: N.M. Paramonov; individualCount: 1; sex: male; occurrenceID: EU_LIM_440; **Taxon:** scientificName: Helius (Helius) flavus (Walker, 1856); family: Limoniidae; genus: Helius; subgenus: Helius; specificEpithet: flavus; scientificNameAuthorship: (Walker, 1856); **Location:** country: Russia; stateProvince: East European Russia; county: Tatarstan Respublika; municipality: Zelenodol’sk district; locality: Zaymishche env., Geomagnetic station; verbatimElevation: 87 m; minimumElevationInMeters: 87; decimalLatitude: 55.82684; decimalLongitude: 48.84395; **Identification:** identifiedBy: N.M. Paramonov; **Event:** samplingProtocol: Sweep net; eventDate: 2012-06-30; verbatimEventDate: 30/Jun/2012; **Record Level:** institutionCode: ZIN; basisOfRecord: PreservedSpecimen
- **Type status:** Other material. **Occurrence:** occurrenceRemarks: 1 male; recordedBy: N.M. Paramonov; individualCount: 1; sex: male; occurrenceID: EU_LIM_441; **Taxon:** scientificName: Helius (Helius) flavus (Walker, 1856); family: Limoniidae; genus: Helius; subgenus: Helius; specificEpithet: flavus; scientificNameAuthorship: (Walker, 1856); **Location:** country: Russia; stateProvince: East European Russia; county: Tatarstan Respublika; municipality: Zelenodol’sk district; locality: Sumka River, Lake Raifskoe; verbatimElevation: 75 m; minimumElevationInMeters: 75; decimalLatitude: 55.91237; decimalLongitude: 48.73159; **Identification:** identifiedBy: N.M. Paramonov; **Event:** samplingProtocol: Sweep net; eventDate: 2009-06-11; verbatimEventDate: 11/Jun/2009; **Record Level:** institutionCode: ZIN; basisOfRecord: PreservedSpecimen

#### Distribution

First records from Belarus and Russia: RUE. The species is recorded from Belgium in the CCW by J. Menten and K. Peeters (in litt. 2012); here we publish the collection data for those records.

### Helius (Helius) hispanicus

Lackschewitz, 1928

164EDC6A-B364-5281-A697-641DF0AB009B

https://ccw.naturalis.nl/detail.php?id=9996

#### Materials

- **Type status:** Other material. **Occurrence:** occurrenceRemarks: 1 male; recordedBy: V.I. Lantsov; individualCount: 1; sex: male; preparations: Pinned; occurrenceID: EU_LIM_442; **Taxon:** scientificName: Helius (Helius) hispanicus Lackschewitz, 1928; family: Limoniidae; genus: Helius; subgenus: Helius; specificEpithet: hispanicus; scientificNameAuthorship: Lackschewitz, 1928; **Location:** country: Russia; stateProvince: North Caucasus; county: Republic of Dagestan; municipality: Magaramkent, Samur; locality: Samur liana forest, in vicinity of village Samur; verbatimElevation: 20 m; minimumElevationInMeters: 20; decimalLatitude: 41.99611; decimalLongitude: 48.485; **Identification:** identifiedBy: V.I. Lantsov; **Event:** samplingProtocol: Sweep net; eventDate: 2016-05-24; verbatimEventDate: May-24-2016; habitat: biotype 2, over near aquatic and aquatic plants along the stream; **Record Level:** institutionCode: ZIN; basisOfRecord: PreservedSpecimen

#### Distribution

Presence of the species in Russia: NC mentioned in Lantsov (2020) without further details. Here, we publish the collection data for that record.

### Helius (Helius) longirostris longirostris

(Meigen, 1818)

91AEC020-2400-5E25-B333-C18E3068CE21

https://ccw.naturalis.nl/detail.php?id=10016

#### Materials

- **Type status:** Other material. **Occurrence:** occurrenceRemarks: 1 male; recordedBy: D.I. Gavryushin; occurrenceID: EU_LIM_443; **Taxon:** scientificName: Helius (Helius) longirostris longirostris (Meigen, 1818); family: Limoniidae; genus: Helius; subgenus: Helius; specificEpithet: longirostris; infraspecificEpithet: longirostris; scientificNameAuthorship: (Meigen, 1818); **Location:** country: Russia; stateProvince: East European Russia; county: Bashkortostan Respublika; municipality: Beloretsk district; locality: Nura River; verbatimElevation: 494 m; minimumElevationInMeters: 494; decimalLatitude: 53.97365; decimalLongitude: 58.34415; **Identification:** identifiedBy: D.I. Gavryushin; **Event:** samplingProtocol: Sweep net; eventDate: 2012-08-10; verbatimEventDate: 10/Aug/2012; **Record Level:** institutionCode: ZMMU; basisOfRecord: PreservedSpecimen
- **Type status:** Other material. **Occurrence:** occurrenceRemarks: 1 female; recordedBy: I.V. Lyubvina; occurrenceID: EU_LIM_444; **Taxon:** scientificName: Helius (Helius) longirostris longirostris (Meigen, 1818); family: Limoniidae; genus: Helius; subgenus: Helius; specificEpithet: longirostris; infraspecificEpithet: longirostris; scientificNameAuthorship: (Meigen, 1818); **Location:** country: Russia; stateProvince: East European Russia; county: Samarskaya Oblast; locality: Zhiguli Nature Reserve, natural boundary Churokayka; verbatimElevation: 155 m; minimumElevationInMeters: 155; decimalLatitude: 53.32359; decimalLongitude: 49.83607; **Identification:** identifiedBy: N.M. Paramonov; **Event:** samplingProtocol: Sweep net; eventDate: 1996-06-13; verbatimEventDate: 13/Jun/1996; habitat: dry meadow; **Record Level:** institutionCode: ZIN; basisOfRecord: PreservedSpecimen
- **Type status:** Other material. **Occurrence:** occurrenceRemarks: 1 male; recordedBy: N.M. Paramonov; occurrenceID: EU_LIM_445; **Taxon:** scientificName: Helius (Helius) longirostris longirostris (Meigen, 1818); family: Limoniidae; genus: Helius; subgenus: Helius; specificEpithet: longirostris; infraspecificEpithet: longirostris; scientificNameAuthorship: (Meigen, 1818); **Location:** country: Russia; stateProvince: East European Russia; county: Tatarstan Respublika; municipality: Laishevo district; locality: Volga-Kama State Nature Biosphere Reserve, «Saraly»; verbatimElevation: 71 m; minimumElevationInMeters: 71; decimalLatitude: 55.29303; decimalLongitude: 49.29976; **Identification:** identifiedBy: N.M. Paramonov; **Event:** samplingProtocol: Sweep net; eventDate: 2009-06-18; verbatimEventDate: 18/Jun/2009; habitat: wetland; **Record Level:** institutionCode: ZIN; basisOfRecord: PreservedSpecimen
- **Type status:** Other material. **Occurrence:** occurrenceRemarks: 2 males, 1 male; recordedBy: N.M. Paramonov; occurrenceID: EU_LIM_446; **Taxon:** scientificName: Helius (Helius) longirostris longirostris (Meigen, 1818); family: Limoniidae; genus: Helius; subgenus: Helius; specificEpithet: longirostris; infraspecificEpithet: longirostris; scientificNameAuthorship: (Meigen, 1818); **Location:** country: Russia; stateProvince: East European Russia; county: Tatarstan Respublika; locality: city Kazan, district Derbyshki; verbatimElevation: 55 m; minimumElevationInMeters: 55; decimalLatitude: 55.87669; decimalLongitude: 49.19071; **Identification:** identifiedBy: N.M. Paramonov; **Event:** samplingProtocol: Sweep net; eventDate: 2010-06-18; verbatimEventDate: 18/Jun/2010; habitat: wetland; **Record Level:** institutionCode: ZIN; basisOfRecord: PreservedSpecimen
- **Type status:** Other material. **Occurrence:** occurrenceRemarks: 1 male, 1 female; recordedBy: N.M. Paramonov; occurrenceID: EU_LIM_447; **Taxon:** scientificName: Helius (Helius) longirostris longirostris (Meigen, 1818); family: Limoniidae; genus: Helius; subgenus: Helius; specificEpithet: longirostris; infraspecificEpithet: longirostris; scientificNameAuthorship: (Meigen, 1818); **Location:** country: Russia; stateProvince: East European Russia; county: Tatarstan Respublika; municipality: Zelenodol’sk district; locality: Volga-Kama State Nature Biosphere Reserve, «Raifa»; verbatimElevation: 100 m; minimumElevationInMeters: 100; decimalLatitude: 55.88868; decimalLongitude: 48.71434; **Identification:** identifiedBy: N.M. Paramonov; **Event:** samplingProtocol: Sweep net; eventDate: 2012-06-26; verbatimEventDate: 26/Jun/2012; **Record Level:** institutionCode: ZIN; basisOfRecord: PreservedSpecimen
- **Type status:** Other material. **Occurrence:** occurrenceRemarks: 1 male; recordedBy: V.M. Basov; occurrenceID: EU_LIM_448; **Taxon:** scientificName: Helius (Helius) longirostris longirostris (Meigen, 1818); family: Limoniidae; genus: Helius; subgenus: Helius; specificEpithet: longirostris; infraspecificEpithet: longirostris; scientificNameAuthorship: (Meigen, 1818); **Location:** country: Russia; stateProvince: East European Russia; county: Tatarstan Respublika; municipality: Laishevo district; locality: Volga-Kama State Nature Biosphere Reserve, «Saraly», Island Ornitologicheskiy; verbatimElevation: 50 m; minimumElevationInMeters: 50; decimalLatitude: 55.28392; decimalLongitude: 49.26081; **Identification:** identifiedBy: N.M. Paramonov; **Event:** samplingProtocol: Sweep net; eventDate: 2007-06-27; verbatimEventDate: 27/Jul/2007; **Record Level:** institutionCode: ZIN; basisOfRecord: PreservedSpecimen
- **Type status:** Other material. **Occurrence:** occurrenceRemarks: 1 male; recordedBy: L.-P. Kolcsár; preparations: Ethanol; occurrenceID: EU_LIM_449; **Taxon:** scientificName: Helius (Helius) longirostris longirostris (Meigen, 1818); family: Limoniidae; genus: Helius; subgenus: Helius; specificEpithet: longirostris; infraspecificEpithet: longirostris; scientificNameAuthorship: (Meigen, 1818); **Location:** country: Serbia; municipality: Kovin; verbatimElevation: 66 m; minimumElevationInMeters: 66; decimalLatitude: 44.73265; decimalLongitude: 20.96622; **Identification:** identifiedBy: L.-P. Kolcsár; **Event:** samplingProtocol: Sweep net; eventDate: 2017-05-07; verbatimEventDate: 7/May/2017; **Record Level:** institutionCode: CKLP; basisOfRecord: PreservedSpecimen
- **Type status:** Other material. **Occurrence:** occurrenceRemarks: 4 males, 3 females; recordedBy: L.-P. Kolcsár | E. Török; preparations: Ethanol; occurrenceID: EU_LIM_450; **Taxon:** scientificName: Helius (Helius) longirostris longirostris (Meigen, 1818); family: Limoniidae; genus: Helius; subgenus: Helius; specificEpithet: longirostris; infraspecificEpithet: longirostris; scientificNameAuthorship: (Meigen, 1818); **Location:** country: Serbia; municipality: Banatska Palanka; locality: Kanal DTD; verbatimElevation: 67 m; minimumElevationInMeters: 67; decimalLatitude: 44.85833; decimalLongitude: 21.30525; **Identification:** identifiedBy: L.-P. Kolcsár; **Event:** samplingProtocol: Sweep net; eventDate: 2017-04-30; verbatimEventDate: 30/Apr/2017; **Record Level:** institutionCode: CKLP; basisOfRecord: PreservedSpecimen

#### Distribution

First records from Russia: RUE and Serbia.

### Helius (Helius) pallirostris

Edwards, 1921

64D70693-D721-5AAC-87C7-DD2A3CF2E36C

https://ccw.naturalis.nl/detail.php?id=10044

#### Materials

- **Type status:** Other material. **Occurrence:** occurrenceRemarks: 1 female; recordedBy: K. Peeters; individualCount: 1; sex: female; occurrenceID: EU_LIM_451; **Taxon:** scientificName: Helius (Helius) pallirostris Edwards, 1921; family: Limoniidae; genus: Helius; subgenus: Helius; specificEpithet: pallirostris; scientificNameAuthorship: Edwards, 1921; **Location:** country: Belgium; stateProvince: Oost-Vlaanderen; municipality: Kieldrecht; locality: De Putten; decimalLatitude: 51.28; decimalLongitude: 4.17; **Identification:** identifiedBy: P. Oosterbroek; **Event:** eventDate: 2012-08-22; verbatimEventDate: 22/Aug/2012; **Record Level:** basisOfRecord: HumanObservation

#### Distribution

The species is mentioned from Belgium in the CCW by K. Peeters (in litt. 2012); here, we publish the collection data for that record.

### Hexatoma (Coreozelia) cimicoides

(Scopoli, 1763)

7D20F4D8-3C8D-5141-9C63-06573E1B9D86

https://ccw.naturalis.nl/detail.php?id=5889

#### Materials

- **Type status:** Other material. **Occurrence:** occurrenceRemarks: 1 male, 1 female; recordedBy: D.I. Gavryushin; individualCount: 2; sex: male, female; preparations: Pinned; occurrenceID: EU_LIM_452; **Taxon:** scientificName: Hexatoma (Coreozelia) cimicoides (Scopoli, 1763); family: Limoniidae; genus: Hexatoma; subgenus: Coreozelia; specificEpithet: cimicoides; scientificNameAuthorship: (Scopoli, 1763); **Location:** country: Serbia; stateProvince: Zaječar; municipality: Knjaževac; locality: Knjaževac; decimalLatitude: 43.55; decimalLongitude: 22.24; **Identification:** identifiedBy: D.I. Gavryushin; **Event:** samplingProtocol: Sweep net; eventDate: 2015-04-27/2015-04-30; verbatimEventDate: 27-30/Apr/2015; **Record Level:** institutionCode: ZMMU; basisOfRecord: PreservedSpecimen

#### Distribution

First record from Serbia.

### Hexatoma (Hexatoma) fuscipennis

(Curtis, 1836)

BE6CE7CB-9C5E-5706-ABC9-7A5BF77FC8F6

https://ccw.naturalis.nl/detail.php?id=6483

#### Materials

- **Type status:** Other material. **Occurrence:** catalogNumber: 633997; occurrenceRemarks: 1 male; recordedBy: S. Olberg; individualCount: 1; sex: male; preparations: Ethanol; occurrenceID: EU_LIM_453; **Taxon:** scientificName: Hexatoma (Hexatoma) fuscipennis (Curtis, 1836); family: Limoniidae; genus: Hexatoma; subgenus: Hexatoma; specificEpithet: fuscipennis; scientificNameAuthorship: (Curtis, 1836); **Location:** country: Norway; stateProvince: Finnmark; municipality: Karasjok; locality: Dorvonjarga; verbatimElevation: 125 m; minimumElevationInMeters: 125; decimalLatitude: 69.40415; decimalLongitude: 25.82222; **Identification:** identifiedBy: K.M. Olsen; **Event:** samplingProtocol: Sweep net; eventDate: 2019-06-22; verbatimEventDate: Jul-22-2019; **Record Level:** institutionCode: PCKMO; basisOfRecord: PreservedSpecimen
- **Type status:** Other material. **Occurrence:** catalogNumber: 666173; occurrenceRemarks: 4 male+female; recordedBy: K.M. Olsen; individualCount: 4; sex: male, female; preparations: Ethanol; occurrenceID: EU_LIM_454; **Taxon:** scientificName: Hexatoma (Hexatoma) fuscipennis (Curtis, 1836); family: Limoniidae; genus: Hexatoma; subgenus: Hexatoma; specificEpithet: fuscipennis; scientificNameAuthorship: (Curtis, 1836); **Location:** country: Norway; stateProvince: Finnmark; municipality: Tana; locality: Bodnesáttu – (N Seida); verbatimElevation: 5 m; minimumElevationInMeters: 5; decimalLatitude: 70.24973; decimalLongitude: 28.1806; **Identification:** identifiedBy: K.M. Olsen; **Event:** samplingProtocol: Sweep net; eventDate: 2020-06-07; verbatimEventDate: 07/Jul/2020; **Record Level:** institutionCode: PCKMO; basisOfRecord: PreservedSpecimen
- **Type status:** Other material. **Occurrence:** catalogNumber: 666236; occurrenceRemarks: 8 male+female; recordedBy: K.M. Olsen; individualCount: 8; sex: male, female; preparations: Ethanol; occurrenceID: EU_LIM_455; **Taxon:** scientificName: Hexatoma (Hexatoma) fuscipennis (Curtis, 1836); family: Limoniidae; genus: Hexatoma; subgenus: Hexatoma; specificEpithet: fuscipennis; scientificNameAuthorship: (Curtis, 1836); **Location:** country: Norway; stateProvince: Finnmark; municipality: Tana; locality: Lismajoki–Nuorinjálbmi; verbatimElevation: 10 m; minimumElevationInMeters: 10; decimalLatitude: 70.14961; decimalLongitude: 28.18817; **Identification:** identifiedBy: K.M. Olsen; **Event:** samplingProtocol: Sweep net; eventDate: 2020-06-08; verbatimEventDate: 08/Jul/2020; **Record Level:** institutionCode: ZMUB; basisOfRecord: PreservedSpecimen
- **Type status:** Other material. **Occurrence:** catalogNumber: 666369; occurrenceRemarks: 3 male+female; recordedBy: K.M. Olsen; individualCount: 3; sex: male, female; occurrenceID: EU_LIM_456; **Taxon:** scientificName: Hexatoma (Hexatoma) fuscipennis (Curtis, 1836); family: Limoniidae; genus: Hexatoma; subgenus: Hexatoma; specificEpithet: fuscipennis; scientificNameAuthorship: (Curtis, 1836); **Location:** country: Norway; stateProvince: Finnmark; municipality: Karasjok; locality: Gámehisjoganjálbmi; verbatimElevation: 125 m; minimumElevationInMeters: 125; decimalLatitude: 69.41823; decimalLongitude: 25.80078; **Identification:** identifiedBy: K.M. Olsen; **Event:** samplingProtocol: Sweep net; eventDate: 2020-06-09; verbatimEventDate: 09/Jul/2020; **Record Level:** institutionCode: BioFokus; basisOfRecord: HumanObservation
- **Type status:** Other material. **Occurrence:** catalogNumber: 666400; occurrenceRemarks: 4 male+female; recordedBy: K.M. Olsen; individualCount: 4; sex: male, female; occurrenceID: EU_LIM_457; **Taxon:** scientificName: Hexatoma (Hexatoma) fuscipennis (Curtis, 1836); family: Limoniidae; genus: Hexatoma; subgenus: Hexatoma; specificEpithet: fuscipennis; scientificNameAuthorship: (Curtis, 1836); **Location:** country: Norway; stateProvince: Finnmark; municipality: Karasjok; locality: Sávkadasnjálbmi; verbatimElevation: 120 m; minimumElevationInMeters: 120; decimalLatitude: 69.54093; decimalLongitude: 25.83541; **Identification:** identifiedBy: K.M. Olsen; **Event:** samplingProtocol: Sweep net; eventDate: 2020-06-09; verbatimEventDate: 09/Jul/2020; **Record Level:** institutionCode: BioFokus; basisOfRecord: HumanObservation
- **Type status:** Other material. **Occurrence:** occurrenceRemarks: 5 males, 2 females; recordedBy: D.I. Gavryushin; individualCount: 7; sex: male, female; occurrenceID: EU_LIM_458; **Taxon:** scientificName: Hexatoma (Hexatoma) fuscipennis (Curtis, 1836); family: Limoniidae; genus: Hexatoma; subgenus: Hexatoma; specificEpithet: fuscipennis; scientificNameAuthorship: (Curtis, 1836); **Location:** country: Russia; stateProvince: North European Russia; county: Murmansk region; municipality: Kolsky district; locality: Molochny env., Kola River; verbatimElevation: 20 m; minimumElevationInMeters: 20; decimalLatitude: 68.84773; decimalLongitude: 33.04877; **Identification:** identifiedBy: D.I. Gavryushin; **Event:** samplingProtocol: Sweep net; eventDate: 2011-06-20; verbatimEventDate: 20/Jul/2011; **Record Level:** institutionCode: ZMMU; basisOfRecord: PreservedSpecimen
- **Type status:** Other material. **Occurrence:** occurrenceRemarks: 1 male; recordedBy: A. Polevoi; individualCount: 1; sex: male; preparations: Pinned; occurrenceID: EU_LIM_459; **Taxon:** scientificName: Hexatoma (Hexatoma) fuscipennis (Curtis, 1836); family: Limoniidae; genus: Hexatoma; subgenus: Hexatoma; specificEpithet: fuscipennis; scientificNameAuthorship: (Curtis, 1836); **Location:** country: Russia; stateProvince: North European Russia; county: Republic Karelia; municipality: Pudozh district; locality: Schanikovskaya; verbatimElevation: 120 m; minimumElevationInMeters: 120; decimalLatitude: 61.76084; decimalLongitude: 37.73409; **Identification:** identifiedBy: A. Polevoi; **Event:** samplingProtocol: Sweep net; eventDate: 2009-06-22; verbatimEventDate: 22/Jun/2009; **Record Level:** institutionCode: FRIP; basisOfRecord: PreservedSpecimen
- **Type status:** Other material. **Occurrence:** occurrenceRemarks: 1 male; recordedBy: D. Janevic; individualCount: 1; sex: male; occurrenceID: EU_LIM_460; **Taxon:** scientificName: Hexatoma (Hexatoma) fuscipennis (Curtis, 1836); family: Limoniidae; genus: Hexatoma; subgenus: Hexatoma; specificEpithet: fuscipennis; scientificNameAuthorship: (Curtis, 1836); **Location:** country: Slovenia; municipality: Celje; locality: in the forest near Celje; decimalLatitude: 46.22; decimalLongitude: 15.26; **Identification:** identifiedBy: P. Oosterbroek; **Event:** eventDate: 2016-05-06; verbatimEventDate: 06/May/2016; **Record Level:** basisOfRecord: HumanObservation

#### Distribution

First records from Norway and Russia: RUN. The species is mentioned from Slovenia in the CCW by D. Janevic (in litt. 2017); here, we publish the collection data for that record.

### Hexatoma (Hexatoma) vittata

(Meigen, 1830)

054F7EBE-5CE2-57F9-8AE8-A27FD3133F65

https://ccw.naturalis.nl/detail.php?id=6509

#### Materials

- **Type status:** Other material. **Occurrence:** occurrenceRemarks: 2 males; recordedBy: C. Quindroit; individualCount: 2; sex: male; preparations: Ethanol; occurrenceID: EU_LIM_461; **Taxon:** scientificName: Hexatoma (Hexatoma) vittata (Meigen, 1830); family: Limoniidae; genus: Hexatoma; subgenus: Hexatoma; specificEpithet: vittata; scientificNameAuthorship: (Meigen, 1830); **Location:** country: France; municipality: Villeneuve-de-Rivière; locality: Moulin saint Jean; verbatimElevation: 350 m; minimumElevationInMeters: 350; decimalLatitude: 43.10793; decimalLongitude: 0.66069; **Identification:** identifiedBy: C. Quindroit; **Event:** samplingProtocol: Sweep net; eventDate: 2016-04-28; verbatimEventDate: 28/Apr/2016; **Record Level:** institutionCode: PCCQ; basisOfRecord: PreservedSpecimen

#### Distribution

First record from France (from mainland).

### Hoplolabis (Parilisia) subareolata

(Alexander, 1932)

5E458765-29C9-587B-BB22-7E2616E923F6

https://ccw.naturalis.nl/detail.php?id=2172

#### Materials

- **Type status:** Other material. **Occurrence:** occurrenceRemarks: 2 males, 2 females; recordedBy: D.I. Gavryushin; individualCount: 4; sex: male, female; preparations: Pinned; occurrenceID: EU_LIM_462; **Taxon:** scientificName: Hoplolabis (Parilisia) subareolata (Alexander, 1932); family: Limoniidae; genus: Hoplolabis; subgenus: Parilisia; specificEpithet: subareolata; scientificNameAuthorship: (Alexander, 1932); **Location:** country: Serbia; stateProvince: Zaječar; municipality: Knjaževac; locality: Kalna, Timok River; decimalLatitude: 43.417; decimalLongitude: 22.424; **Identification:** identifiedBy: D.I. Gavryushin; **Event:** samplingProtocol: Sweep net; eventDate: 2014-09-19/2014-09-21; verbatimEventDate: 19-21/Sep/2014; **Record Level:** institutionCode: ZMMU; basisOfRecord: PreservedSpecimen

#### Distribution

First record from Serbia.

### Idiocera (Idiocera) pallens

(Alexander, 1928)

2293D337-D6E5-5887-8E90-2D80749CC886

https://ccw.naturalis.nl/detail.php?id=2293

#### Materials

- **Type status:** Other material. **Occurrence:** catalogNumber: THS-20160006; occurrenceRemarks: 1 male; recordedBy: J. Salmela; individualCount: 1; sex: male; preparations: Ethanol; occurrenceID: EU_LIM_463; **Taxon:** scientificName: Idiocera (Idiocera) pallens (Alexander, 1928); family: Limoniidae; genus: Idiocera; subgenus: Idiocera; specificEpithet: pallens; scientificNameAuthorship: (Alexander, 1928); **Location:** country: Finland; stateProvince: Lapponia inariensis; municipality: Utsjoki; locality: Karigasniemi; verbatimElevation: 120 m; minimumElevationInMeters: 120; decimalLatitude: 69.369; decimalLongitude: 25.824; **Identification:** identifiedBy: J. Salmela; **Event:** samplingProtocol: Sweep net; eventDate: 2016-06-11; verbatimEventDate: 11/Jul/2016; **Record Level:** institutionCode: LMM; basisOfRecord: PreservedSpecimen

#### Distribution

First record from Finland.

### Idiocera (Idiocera) sziladyi

(Lackschewitz, 1940)

1724F1E9-12EF-52F9-96FC-D9F56A1B3DCC

https://ccw.naturalis.nl/detail.php?id=2332

#### Materials

- **Type status:** Other material. **Occurrence:** occurrenceRemarks: 1 male; recordedBy: C. Quindroit; individualCount: 1; sex: male; preparations: Ethanol; occurrenceID: EU_LIM_464; **Taxon:** scientificName: Idiocera (Idiocera) sziladyi (Lackschewitz, 1940); family: Limoniidae; genus: Idiocera; subgenus: Idiocera; specificEpithet: sziladyi; scientificNameAuthorship: (Lackschewitz, 1940); **Location:** country: France; municipality: Saumur; locality: Ile Trotuin; decimalLatitude: 47.24796; decimalLongitude: -0.03472; **Identification:** identifiedBy: C. Quindroit; **Event:** samplingProtocol: Sweep net; eventDate: 2020-08-05; verbatimEventDate: 05/Aug/2020; **Record Level:** institutionCode: PCCQ; basisOfRecord: PreservedSpecimen
- **Type status:** Other material. **Occurrence:** occurrenceRemarks: 1 male; recordedBy: C. Quindroit; individualCount: 1; sex: male; preparations: Ethanol; occurrenceID: EU_LIM_465; **Taxon:** scientificName: Idiocera (Idiocera) sziladyi (Lackschewitz, 1940); family: Limoniidae; genus: Idiocera; subgenus: Idiocera; specificEpithet: sziladyi; scientificNameAuthorship: (Lackschewitz, 1940); **Location:** country: France; municipality: Saint-Aignan-le-Jaillard; locality: L'épinoy; decimalLatitude: 47.76721; decimalLongitude: 2.42763; **Identification:** identifiedBy: C. Quindroit; **Event:** samplingProtocol: Sweep net; eventDate: 2019-06-15; verbatimEventDate: 15/Jun/2019; **Record Level:** institutionCode: PCCQ; basisOfRecord: PreservedSpecimen

#### Distribution

First records from France (from mainland).

### Ilisia maculata

(Meigen, 1804)

5804B435-E449-5571-B0F5-38FA41EC8544

https://ccw.naturalis.nl/detail.php?id=2360

#### Materials

- **Type status:** Other material. **Occurrence:** occurrenceRemarks: 1 male; recordedBy: D.I. Gavryushin; individualCount: 1; sex: male; preparations: Pinned; occurrenceID: EU_LIM_466; **Taxon:** scientificName: Ilisiamaculata (Meigen, 1804); family: Limoniidae; genus: Ilisia; specificEpithet: maculata; scientificNameAuthorship: (Meigen, 1804); **Location:** country: Belarus; stateProvince: Minsk; county: Barysaw; locality: Glivin; verbatimElevation: 161 m; minimumElevationInMeters: 161; decimalLatitude: 54.14902; decimalLongitude: 28.63648; **Identification:** identifiedBy: D.I. Gavryushin; **Event:** samplingProtocol: Sweep net; eventDate: 2013-06-06; verbatimEventDate: 6/Jul/2013; **Record Level:** institutionCode: ZMMU; basisOfRecord: PreservedSpecimen
- **Type status:** Other material. **Occurrence:** occurrenceRemarks: 2 females; recordedBy: D.I. Gavryushin; individualCount: 2; sex: female; preparations: Pinned; occurrenceID: EU_LIM_467; **Taxon:** scientificName: Ilisiamaculata (Meigen, 1804); family: Limoniidae; genus: Ilisia; specificEpithet: maculata; scientificNameAuthorship: (Meigen, 1804); **Location:** country: Belarus; stateProvince: Minsk; county: Barysaw; locality: Vialikaje Stachava; verbatimElevation: 156 m; minimumElevationInMeters: 156; decimalLatitude: 54.26555; decimalLongitude: 28.38332; **Identification:** identifiedBy: D.I. Gavryushin; **Event:** samplingProtocol: Sweep net; eventDate: 2013-06-07; verbatimEventDate: 7/Jul/2013; **Record Level:** institutionCode: ZMMU; basisOfRecord: PreservedSpecimen
- **Type status:** Other material. **Occurrence:** occurrenceRemarks: 1 male; recordedBy: N.G. Petrov; individualCount: 1; sex: male; occurrenceID: EU_LIM_468; **Taxon:** scientificName: Ilisiamaculata (Meigen, 1804); family: Limoniidae; genus: Ilisia; specificEpithet: maculata; scientificNameAuthorship: (Meigen, 1804); **Location:** country: Russia; stateProvince: East European Russia; county: Tatarstan Respublika; municipality: Kazan; locality: district Derbyshki, Noksa River; verbatimElevation: 60 m; minimumElevationInMeters: 60; decimalLatitude: 55.8655; decimalLongitude: 49.22461; **Identification:** identifiedBy: N.M. Paramonov; **Event:** samplingProtocol: Sweep net; eventDate: 2011-08-25; verbatimEventDate: 25/Aug/2011; **Record Level:** institutionCode: ZIN; basisOfRecord: PreservedSpecimen

#### Distribution

First records from Belarus and Russia: RUE.

### Ilisia occoecata

Edwards, 1936

B958040E-875F-5305-8F86-C11FC3B10719

https://ccw.naturalis.nl/detail.php?id=2361

#### Materials

- **Type status:** Other material. **Occurrence:** occurrenceRemarks: 1 female; recordedBy: V. Verhoeyen; individualCount: 1; sex: female; occurrenceID: EU_LIM_469; **Taxon:** scientificName: Ilisiaoccoecata Edwards, 1936; family: Limoniidae; genus: Ilisia; specificEpithet: occoecata; scientificNameAuthorship: Edwards, 1936; **Location:** country: Belgium; stateProvince: Oost-Vlaanderen; municipality: Petegem-Leie; decimalLatitude: 50.98; decimalLongitude: 3.52; **Identification:** identifiedBy: P. Oosterbroek; **Event:** eventDate: 2012-06-06; verbatimEventDate: 06/Jun/2012; **Record Level:** basisOfRecord: HumanObservation
- **Type status:** Other material. **Occurrence:** catalogNumber: 611604; occurrenceRemarks: 1 female; recordedBy: K. Berggren; individualCount: 1; sex: female; preparations: Ethanol; occurrenceID: EU_LIM_470; **Taxon:** scientificName: Ilisiaoccoecata Edwards, 1936; family: Limoniidae; genus: Ilisia; specificEpithet: occoecata; scientificNameAuthorship: Edwards, 1936; **Location:** country: Norway; stateProvince: Aust-Agder; municipality: Grimstad; locality: Søm (Skogstuen); verbatimElevation: 10 m; minimumElevationInMeters: 10; decimalLatitude: 58.3896; decimalLongitude: 8.71272; **Identification:** identifiedBy: K.M. Olsen; **Event:** samplingProtocol: Light trap; eventDate: 2017-06/2017-08; verbatimEventDate: Jul-Aug/2017; **Record Level:** institutionCode: PCKMO; basisOfRecord: PreservedSpecimen
- **Type status:** Other material. **Occurrence:** catalogNumber: 534242; occurrenceRemarks: 1 ad.; recordedBy: K.M. Olsen; individualCount: 1; sex: ad.; preparations: Ethanol; occurrenceID: EU_LIM_471; **Taxon:** scientificName: Ilisiaoccoecata Edwards, 1936; family: Limoniidae; genus: Ilisia; specificEpithet: occoecata; scientificNameAuthorship: Edwards, 1936; **Location:** country: Norway; stateProvince: Aust-Agder; municipality: Arendal; locality: Styrsviga; verbatimElevation: 0-3 m; maximumElevationInMeters: 3; decimalLatitude: 58.45696; decimalLongitude: 8.7934; **Identification:** identifiedBy: K.M. Olsen; **Event:** samplingProtocol: Sweep net; eventDate: 2017-08-05; verbatimEventDate: 05/Aug/2017; **Record Level:** institutionCode: PCKMO; basisOfRecord: PreservedSpecimen
- **Type status:** Other material. **Occurrence:** catalogNumber: 651362; occurrenceRemarks: 1 female; recordedBy: J. Svetlik | I. Børja; individualCount: 1; sex: female; preparations: Ethanol; occurrenceID: EU_LIM_472; **Taxon:** scientificName: Ilisiaoccoecata Edwards, 1936; family: Limoniidae; genus: Ilisia; specificEpithet: occoecata; scientificNameAuthorship: Edwards, 1936; **Location:** country: Norway; stateProvince: Vestfold; municipality: Horten; locality: Vestmannrød – Ash canopy 12A; verbatimElevation: 10 m; minimumElevationInMeters: 10; decimalLatitude: 59.3655; decimalLongitude: 10.4604; **Identification:** identifiedBy: K.M. Olsen; **Event:** samplingProtocol: Canopy fogging; eventDate: 2018-06-18; verbatimEventDate: 18/07/2018; **Record Level:** institutionCode: ZMUB; basisOfRecord: PreservedSpecimen
- **Type status:** Other material. **Occurrence:** occurrenceRemarks: 1 male; recordedBy: L.-P. Kolcsár; individualCount: 1; sex: male; preparations: Ethanol; occurrenceID: EU_LIM_473; **Taxon:** scientificName: Ilisiaoccoecata Edwards, 1936; family: Limoniidae; genus: Ilisia; specificEpithet: occoecata; scientificNameAuthorship: Edwards, 1936; **Location:** country: Romania; stateProvince: Harghita; municipality: Hagota; locality: Giurgeu Mts., Tisașul Valley; verbatimElevation: 860 m; minimumElevationInMeters: 860; decimalLatitude: 46.86179; decimalLongitude: 25.67723; **Identification:** identifiedBy: L.-P. Kolcsár; **Event:** samplingProtocol: Sweep net; eventDate: 2013-06-28; verbatimEventDate: 28/Jun/2013; **Record Level:** institutionCode: CKLP; basisOfRecord: PreservedSpecimen

#### Description

Fig. 7

#### Distribution

First records from Norway and Romania. The species is reported from Belgium in the CCW by K. Verhoeyen (in litt. 2012); we publish the collection data for that record.

### Libnotes (Afrolimonia) ladogensis

(Lackschewitz, 1940)

8156CEBC-B59B-5D39-9078-C8495260F4EE

https://ccw.naturalis.nl/detail.php?id=10183

#### Materials

- **Type status:** Other material. **Occurrence:** occurrenceRemarks: 1 male; recordedBy: D.I. Gavryushin; individualCount: 1; sex: male; occurrenceID: EU_LIM_474; **Taxon:** scientificName: Libnotes (Afrolimonia) ladogensis (Lackschewitz, 1940); family: Limoniidae; genus: Libnotes; subgenus: Afrolimonia; specificEpithet: ladogensis; scientificNameAuthorship: (Lackschewitz, 1940); **Location:** country: Russia; stateProvince: East European Russia; county: Bashkortostan Respublika; municipality: Beloretsk district; locality: Nura River (ca. 4km W of Otnurok village), at the foot of Zolotyie Shishki (Golden Cones) Mts.; verbatimElevation: 607 m; minimumElevationInMeters: 607; decimalLatitude: 54.05155; decimalLongitude: 58.26887; **Identification:** identifiedBy: D.I. Gavryushin; **Event:** samplingProtocol: Sweep net; eventDate: 2015-06-13; verbatimEventDate: Jul-13-2015; **Record Level:** institutionCode: ZMMU; basisOfRecord: PreservedSpecimen
- **Type status:** Other material. **Occurrence:** occurrenceRemarks: 1 male; recordedBy: N.M. Paramonov; individualCount: 1; sex: male; occurrenceID: EU_LIM_475; **Taxon:** scientificName: Libnotes (Afrolimonia) ladogensis (Lackschewitz, 1940); family: Limoniidae; genus: Libnotes; subgenus: Afrolimonia; specificEpithet: ladogensis; scientificNameAuthorship: (Lackschewitz, 1940); **Location:** country: Russia; stateProvince: East European Russia; county: Tatarstan Respublika; municipality: Zelenodol’sk district; locality: Volga-Kama State Nature Biosphere Reserve, «Raifa»; verbatimElevation: 100 m; minimumElevationInMeters: 100; decimalLatitude: 55.88868; decimalLongitude: 48.71434; **Identification:** identifiedBy: N.M. Paramonov; **Event:** samplingProtocol: Sweep net; eventDate: 2009-06-14; verbatimEventDate: 14/Jun/2009; **Record Level:** institutionCode: ZIN; basisOfRecord: PreservedSpecimen

#### Distribution

First records from Russia: RUE.

### Limnophila (Limnophila) pictipennis

(Meigen, 1818)

564B4EA3-464D-55DE-B084-DB366D552658

https://ccw.naturalis.nl/detail.php?id=6787

#### Materials

- **Type status:** Other material. **Occurrence:** occurrenceRemarks: 1 male; recordedBy: D.I. Gavryushin; individualCount: 1; sex: male; preparations: Pinned; occurrenceID: EU_LIM_476; **Taxon:** scientificName: Limnophila (Limnophila) pictipennis (Meigen, 1818); family: Limoniidae; genus: Limnophila; subgenus: Limnophila; specificEpithet: pictipennis; scientificNameAuthorship: (Meigen, 1818); **Location:** country: Belarus; stateProvince: Vitebsk; county: Haradok; locality: Ezerische; decimalLatitude: 55.83; decimalLongitude: 30; **Identification:** identifiedBy: D.I. Gavryushin; **Event:** samplingProtocol: Sweep net; eventDate: 2019-05-16/2019-05-17; verbatimEventDate: 16-17/May/2019; **Record Level:** institutionCode: ZMMU; basisOfRecord: PreservedSpecimen

#### Distribution

First records from Belarus.

### Limonia albifrons

(Meigen, 1818)

00014D14-75A5-5890-B428-4F14452AB498

https://ccw.naturalis.nl/detail.php?id=10495

#### Materials

- **Type status:** Other material. **Occurrence:** catalogNumber: 583266; occurrenceRemarks: 1 male; recordedBy: K.M. Olsen; individualCount: 1; sex: male; preparations: Ethanol; occurrenceID: EU_LIM_477; **Taxon:** scientificName: Limoniaalbifrons (Meigen, 1818); family: Limoniidae; genus: Limonia; specificEpithet: albifrons; scientificNameAuthorship: (Meigen, 1818); **Location:** country: Norway; stateProvince: Hedmark; municipality: Åmot; locality: Deifjell-lia; verbatimElevation: 395-470 m; minimumElevationInMeters: 395; maximumElevationInMeters: 470; decimalLatitude: 61.28375; decimalLongitude: 11.50163; **Identification:** identifiedBy: K.M. Olsen; **Event:** samplingProtocol: Sweep net; eventDate: 2018-06-06; verbatimEventDate: Jun-06-2018; **Record Level:** institutionCode: PCKMO; basisOfRecord: PreservedSpecimen
- **Type status:** Other material. **Occurrence:** catalogNumber: 642060; occurrenceRemarks: 1 male; recordedBy: E.S. Paulsen | S. Apeland | L.T. Bjørnø; individualCount: 1; sex: male; preparations: Ethanol; occurrenceID: EU_LIM_478; **Taxon:** scientificName: Limoniaalbifrons (Meigen, 1818); family: Limoniidae; genus: Limonia; specificEpithet: albifrons; scientificNameAuthorship: (Meigen, 1818); **Location:** country: Norway; stateProvince: Rogaland; municipality: Suldal; locality: Hedlesbø – Ved grensen til Ørland NR; verbatimElevation: 5 m; minimumElevationInMeters: 5; decimalLatitude: 59.54636; decimalLongitude: 6.3741; **Identification:** identifiedBy: K.M. Olsen; **Event:** samplingProtocol: Malaise trap; eventDate: 2019-06-10/2019-07-03; verbatimEventDate: 10/Jun-03/Jul/2019; **Record Level:** institutionCode: PCKMO; basisOfRecord: PreservedSpecimen
- **Type status:** Other material. **Occurrence:** occurrenceRemarks: 1 male; recordedBy: N.M. Paramonov; individualCount: 1; sex: male; occurrenceID: EU_LIM_479; **Taxon:** scientificName: Limoniaalbifrons (Meigen, 1818); family: Limoniidae; genus: Limonia; specificEpithet: albifrons; scientificNameAuthorship: (Meigen, 1818); **Location:** country: Russia; stateProvince: East European Russia; county: Tatarstan Respublika; municipality: Zelenodol’sk district; locality: Sumka River, Lake Raifskoe; verbatimElevation: 75 m; minimumElevationInMeters: 75; decimalLatitude: 55.91237; decimalLongitude: 48.73159; **Identification:** identifiedBy: N.M. Paramonov; **Event:** samplingProtocol: Sweep net; eventDate: 2009-06-11; verbatimEventDate: 11/Jun/2009; **Record Level:** institutionCode: ZIN; basisOfRecord: PreservedSpecimen

#### Distribution

First records from Norway and Russia: RUE.

### Limonia aquilina

Starý, 1984

2EF31687-0804-5A8B-BD0B-4E844A57182F

https://ccw.naturalis.nl/detail.php?id=10510

#### Materials

- **Type status:** Other material. **Occurrence:** catalogNumber: JES-20110511; occurrenceRemarks: 1 female; recordedBy: J. Ilmonen; individualCount: 1; sex: female; preparations: Ethanol; occurrenceID: EU_LIM_480; **Taxon:** scientificName: Limoniaaquilina Starý, 1984; family: Limoniidae; genus: Limonia; specificEpithet: aquilina; scientificNameAuthorship: Starý, 1984; **Location:** country: Finland; stateProvince: Regio aboensis; municipality: Vihti; locality: Myllypuro; verbatimElevation: 40 m; minimumElevationInMeters: 40; decimalLatitude: 60.333; decimalLongitude: 24.501; **Identification:** identifiedBy: J. Salmela; **Event:** samplingProtocol: Malaise trap; eventDate: 2005-08-01; verbatimEventDate: 1/Aug/2005; **Record Level:** institutionCode: ZMUT; basisOfRecord: PreservedSpecimen

#### Distribution

First record from Finland.

### Limonia badia

(Walker, 1848)

81E27418-2C5B-5551-8611-8C6EFAFC3919

https://ccw.naturalis.nl/detail.php?id=10518

#### Materials

- **Type status:** Other material. **Occurrence:** catalogNumber: Aug-10-3560; occurrenceRemarks: 1 female; recordedBy: K.M. Olsen; individualCount: 1; sex: female; preparations: Ethanol; occurrenceID: EU_LIM_481; **Taxon:** scientificName: Limoniabadia (Walker, 1848); family: Limoniidae; genus: Limonia; specificEpithet: badia; scientificNameAuthorship: (Walker, 1848); **Location:** country: Norway; stateProvince: Hedmark; municipality: Åmot; locality: Deifjell-lia; verbatimElevation: 515 m; minimumElevationInMeters: 515; decimalLatitude: 61.28427; decimalLongitude: 11.50497; **Identification:** identifiedBy: K.M. Olsen; **Event:** samplingProtocol: Malaise trap; eventDate: 2018-06-06/2018-09-22; verbatimEventDate: 06/Jul-22/Sep/2018; **Record Level:** institutionCode: PCKMO; basisOfRecord: PreservedSpecimen
- **Type status:** Other material. **Occurrence:** occurrenceRemarks: 1 male; recordedBy: A. Polevoi; individualCount: 1; sex: male; preparations: Pinned; occurrenceID: EU_LIM_482; **Taxon:** scientificName: Limoniabadia (Walker, 1848); family: Limoniidae; genus: Limonia; specificEpithet: badia; scientificNameAuthorship: (Walker, 1848); **Location:** country: Russia; stateProvince: Northwest European Russia; county: Leningrad province; municipality: Lodeinoe Pole District; locality: River Ludanka, 3 km SW of Yanega; verbatimElevation: 25 m; minimumElevationInMeters: 25; decimalLatitude: 60.7203; decimalLongitude: 33.66034; **Identification:** identifiedBy: A. Polevoi; **Event:** samplingProtocol: Sweep net; eventDate: 2019-08-25; verbatimEventDate: 25/Aug/2019; **Record Level:** institutionCode: ZMMU; basisOfRecord: PreservedSpecimen

#### Distribution

First records from Norway and Russia: RUW.

### Limonia dilutior

(Edwards, 1921)

1E49DBD9-24FA-5092-8AA7-62EB429AE496

https://ccw.naturalis.nl/detail.php?id=10538

#### Materials

- **Type status:** Other material. **Occurrence:** catalogNumber: Jul-14-3403; occurrenceRemarks: 2 males; recordedBy: J.S. Birkeland; individualCount: 2; sex: male; preparations: Ethanol; occurrenceID: EU_LIM_483; **Taxon:** scientificName: Limoniadilutior (Edwards, 1921); family: Limoniidae; genus: Limonia; specificEpithet: dilutior; scientificNameAuthorship: (Edwards, 1921); **Location:** country: Norway; stateProvince: Rogaland; municipality: Sokndal; locality: Birkeland; verbatimElevation: 145 m; minimumElevationInMeters: 145; decimalLatitude: 58.35646; decimalLongitude: 6.15446; **Identification:** identifiedBy: K.M. Olsen; **Event:** samplingProtocol: Malaise trap; eventDate: 2017-06-15/2017-10-01; verbatimEventDate: 15/Jul-01/Oct/2017; **Record Level:** institutionCode: PCKMO; basisOfRecord: PreservedSpecimen
- **Type status:** Other material. **Occurrence:** catalogNumber: 593015; occurrenceRemarks: 1 male; recordedBy: J.S. Birkeland; individualCount: 1; sex: male; preparations: Ethanol; occurrenceID: EU_LIM_484; **Taxon:** scientificName: Limoniadilutior (Edwards, 1921); family: Limoniidae; genus: Limonia; specificEpithet: dilutior; scientificNameAuthorship: (Edwards, 1921); **Location:** country: Norway; stateProvince: Rogaland; municipality: Sokndal; locality: Sandbekk – Sandtippen; verbatimElevation: 110 m; minimumElevationInMeters: 110; decimalLatitude: 58.35833; decimalLongitude: 6.3325; **Identification:** identifiedBy: K.M. Olsen; **Event:** samplingProtocol: SLAM-trap; eventDate: 2018-04-29/2018-10-04; verbatimEventDate: 29/Apr-04/Oct/2018; **Record Level:** institutionCode: PCKMO; basisOfRecord: PreservedSpecimen
- **Type status:** Other material. **Occurrence:** occurrenceRemarks: 1 male; recordedBy: E. Eiroa; individualCount: 1; sex: male; preparations: Pinned; occurrenceID: EU_LIM_485; **Taxon:** scientificName: Limoniaflavipes (Fabricius, 1787); family: Limoniidae; genus: Limonia; specificEpithet: flavipes; scientificNameAuthorship: (Fabricius, 1787); **Location:** country: Portugal; stateProvince: Guarda; municipality: Manteigas; locality: Fonte da Jonja, serra da Estrela; verbatimElevation: 1360 m; minimumElevationInMeters: 1360; decimalLatitude: 40.32452; decimalLongitude: -7.57769; **Identification:** identifiedBy: E. Eiroa; **Event:** samplingProtocol: Sweep net; eventDate: 1992-05-30; verbatimEventDate: 30/May/1992; habitat: riverside vegetation; **Record Level:** institutionCode: USC; basisOfRecord: PreservedSpecimen
- **Type status:** Other material. **Occurrence:** occurrenceRemarks: 3 males; recordedBy: E. Eiroa; individualCount: 3; sex: male; preparations: Pinned; occurrenceID: EU_LIM_486; **Taxon:** scientificName: Limoniaflavipes (Fabricius, 1787); family: Limoniidae; genus: Limonia; specificEpithet: flavipes; scientificNameAuthorship: (Fabricius, 1787); **Location:** country: Portugal; stateProvince: Guarda; municipality: Seia; locality: Vasqueanes, serra da Estrela; verbatimElevation: 1384 m; minimumElevationInMeters: 1384; decimalLatitude: 40.38059; decimalLongitude: -7.64948; **Identification:** identifiedBy: E. Eiroa; **Event:** samplingProtocol: Sweep net; eventDate: 1992-05-29; verbatimEventDate: 29/May/1992; **Record Level:** institutionCode: USC; basisOfRecord: PreservedSpecimen
- **Type status:** Other material. **Occurrence:** occurrenceRemarks: 2 males, 1 female; recordedBy: D.I. Gavryushin; individualCount: 3; sex: male, female; occurrenceID: EU_LIM_487; **Taxon:** scientificName: Limoniaflavipes (Fabricius, 1787); family: Limoniidae; genus: Limonia; specificEpithet: flavipes; scientificNameAuthorship: (Fabricius, 1787); **Location:** country: Russia; stateProvince: East European Russia; county: Bashkortostan Respublika; municipality: Uchaly district; locality: Ural-Tau St. env., upper reaches of Mindyak River; verbatimElevation: 765 m; minimumElevationInMeters: 765; decimalLatitude: 53.96525; decimalLongitude: 58.57888; **Identification:** identifiedBy: D.I. Gavryushin; **Event:** samplingProtocol: Sweep net; eventDate: 2015-06-09; verbatimEventDate: 09/Jul/2015; **Record Level:** institutionCode: ZMMU; basisOfRecord: PreservedSpecimen
- **Type status:** Other material. **Occurrence:** occurrenceRemarks: 1 male, 1 female; recordedBy: D.I. Gavryushin; individualCount: 2; sex: male, female; occurrenceID: EU_LIM_488; **Taxon:** scientificName: Limoniaflavipes (Fabricius, 1787); family: Limoniidae; genus: Limonia; specificEpithet: flavipes; scientificNameAuthorship: (Fabricius, 1787); **Location:** country: Russia; stateProvince: East European Russia; county: Bashkortostan Respublika; municipality: Uchaly district; locality: Ural-Tau St. env.; verbatimElevation: 777 m; minimumElevationInMeters: 777; decimalLatitude: 53.96805; decimalLongitude: 58.57613; **Identification:** identifiedBy: D.I. Gavryushin; **Event:** samplingProtocol: Sweep net; eventDate: 2015-06-09; verbatimEventDate: 09/Jul/2015; **Record Level:** institutionCode: ZMMU; basisOfRecord: PreservedSpecimen
- **Type status:** Other material. **Occurrence:** occurrenceRemarks: 4 males, 1 female; recordedBy: D.I. Gavryushin; individualCount: 5; sex: male, female; occurrenceID: EU_LIM_489; **Taxon:** scientificName: Limoniaflavipes (Fabricius, 1787); family: Limoniidae; genus: Limonia; specificEpithet: flavipes; scientificNameAuthorship: (Fabricius, 1787); **Location:** country: Russia; stateProvince: East European Russia; county: Bashkortostan Respublika; municipality: Beloretsk district; locality: Nura River (ca. 4km W of Otnurok village), at the foot of Zolotyie Shishki (Golden Cones) Mts.; verbatimElevation: 607 m; minimumElevationInMeters: 607; decimalLatitude: 54.05155; decimalLongitude: 58.26887; **Identification:** identifiedBy: D.I. Gavryushin; **Event:** samplingProtocol: Sweep net; eventDate: 2015-06-10; verbatimEventDate: 10/Jul/2015; **Record Level:** institutionCode: ZMMU; basisOfRecord: PreservedSpecimen
- **Type status:** Other material. **Occurrence:** occurrenceRemarks: 1 male; recordedBy: V.I. Lantsov; individualCount: 1; sex: male; preparations: Pinned; occurrenceID: EU_LIM_490; **Taxon:** scientificName: Limoniaflavipes (Fabricius, 1787); family: Limoniidae; genus: Limonia; specificEpithet: flavipes; scientificNameAuthorship: (Fabricius, 1787); **Location:** country: Russia; stateProvince: North Caucasus; county: Republic of Dagestan; municipality: Magaramkent, Primorskiy; locality: In 3 km NW of the village Primorskiy, Dagestan Nature Reserve; verbatimElevation: 16 m; minimumElevationInMeters: 16; decimalLatitude: 41.86472; decimalLongitude: 48.61083; **Identification:** identifiedBy: V.I. Lantsov; **Event:** samplingProtocol: Sweep net; eventDate: 2014-05-17; verbatimEventDate: 17/May/2014; habitat: Samur liana forest, hornbeam and spurge liana forest, with an admixture of oak, maple and high ash - elderberry, horsetail, sweeping on plants along the stream; **Record Level:** institutionCode: ZIN; basisOfRecord: PreservedSpecimen

#### Distribution

First records from Norway.

### Limonia flavipes

(Fabricius, 1787)

AAE1614A-B9F3-523D-842A-633BCECF9DB2

https://ccw.naturalis.nl/detail.php?id=10558

#### Materials

- **Type status:** Other material. **Occurrence:** occurrenceRemarks: 1 male; recordedBy: E. Eiroa; individualCount: 1; sex: male; preparations: Pinned; occurrenceID: EU_LIM_485; **Taxon:** scientificName: Limoniaflavipes (Fabricius, 1787); family: Limoniidae; genus: Limonia; specificEpithet: flavipes; scientificNameAuthorship: (Fabricius, 1787); **Location:** country: Portugal; stateProvince: Guarda; municipality: Manteigas; locality: Fonte da Jonja, serra da Estrela; verbatimElevation: 1360 m; minimumElevationInMeters: 1360; decimalLatitude: 40.32452; decimalLongitude: -7.57769; **Identification:** identifiedBy: E. Eiroa; **Event:** samplingProtocol: Sweep net; eventDate: 1992-05-30; verbatimEventDate: 30/May/1992; habitat: riverside vegetation; **Record Level:** institutionCode: USC; basisOfRecord: PreservedSpecimen
- **Type status:** Other material. **Occurrence:** occurrenceRemarks: 3 males; recordedBy: E. Eiroa; individualCount: 3; sex: male; preparations: Pinned; occurrenceID: EU_LIM_486; **Taxon:** scientificName: Limoniaflavipes (Fabricius, 1787); family: Limoniidae; genus: Limonia; specificEpithet: flavipes; scientificNameAuthorship: (Fabricius, 1787); **Location:** country: Portugal; stateProvince: Guarda; municipality: Seia; locality: Vasqueanes, serra da Estrela; verbatimElevation: 1384 m; minimumElevationInMeters: 1384; decimalLatitude: 40.38059; decimalLongitude: -7.64948; **Identification:** identifiedBy: E. Eiroa; **Event:** samplingProtocol: Sweep net; eventDate: 1992-05-29; verbatimEventDate: 29/May/1992; **Record Level:** institutionCode: USC; basisOfRecord: PreservedSpecimen
- **Type status:** Other material. **Occurrence:** occurrenceRemarks: 2 males, 1 female; recordedBy: D.I. Gavryushin; individualCount: 3; sex: male, female; occurrenceID: EU_LIM_487; **Taxon:** scientificName: Limoniaflavipes (Fabricius, 1787); family: Limoniidae; genus: Limonia; specificEpithet: flavipes; scientificNameAuthorship: (Fabricius, 1787); **Location:** country: Russia; stateProvince: East European Russia; county: Bashkortostan Respublika; municipality: Uchaly district; locality: Ural-Tau St. env., upper reaches of Mindyak River; verbatimElevation: 765 m; minimumElevationInMeters: 765; decimalLatitude: 53.96525; decimalLongitude: 58.57888; **Identification:** identifiedBy: D.I. Gavryushin; **Event:** samplingProtocol: Sweep net; eventDate: 2015-06-09; verbatimEventDate: 09/Jul/2015; **Record Level:** institutionCode: ZMMU; basisOfRecord: PreservedSpecimen
- **Type status:** Other material. **Occurrence:** occurrenceRemarks: 1 male, 1 female; recordedBy: D.I. Gavryushin; individualCount: 2; sex: male, female; occurrenceID: EU_LIM_488; **Taxon:** scientificName: Limoniaflavipes (Fabricius, 1787); family: Limoniidae; genus: Limonia; specificEpithet: flavipes; scientificNameAuthorship: (Fabricius, 1787); **Location:** country: Russia; stateProvince: East European Russia; county: Bashkortostan Respublika; municipality: Uchaly district; locality: Ural-Tau St. env.; verbatimElevation: 777 m; minimumElevationInMeters: 777; decimalLatitude: 53.96805; decimalLongitude: 58.57613; **Identification:** identifiedBy: D.I. Gavryushin; **Event:** samplingProtocol: Sweep net; eventDate: 2015-06-09; verbatimEventDate: 09/Jul/2015; **Record Level:** institutionCode: ZMMU; basisOfRecord: PreservedSpecimen
- **Type status:** Other material. **Occurrence:** occurrenceRemarks: 4 males, 1 female; recordedBy: D.I. Gavryushin; individualCount: 5; sex: male, female; occurrenceID: EU_LIM_489; **Taxon:** scientificName: Limoniaflavipes (Fabricius, 1787); family: Limoniidae; genus: Limonia; specificEpithet: flavipes; scientificNameAuthorship: (Fabricius, 1787); **Location:** country: Russia; stateProvince: East European Russia; county: Bashkortostan Respublika; municipality: Beloretsk district; locality: Nura River (ca. 4km W of Otnurok village), at the foot of Zolotyie Shishki (Golden Cones) Mts.; verbatimElevation: 607 m; minimumElevationInMeters: 607; decimalLatitude: 54.05155; decimalLongitude: 58.26887; **Identification:** identifiedBy: D.I. Gavryushin; **Event:** samplingProtocol: Sweep net; eventDate: 2015-06-10; verbatimEventDate: 10/Jul/2015; **Record Level:** institutionCode: ZMMU; basisOfRecord: PreservedSpecimen
- **Type status:** Other material. **Occurrence:** occurrenceRemarks: 1 male; recordedBy: V.I. Lantsov; individualCount: 1; sex: male; preparations: Pinned; occurrenceID: EU_LIM_490; **Taxon:** scientificName: Limoniaflavipes (Fabricius, 1787); family: Limoniidae; genus: Limonia; specificEpithet: flavipes; scientificNameAuthorship: (Fabricius, 1787); **Location:** country: Russia; stateProvince: North Caucasus; county: Republic of Dagestan; municipality: Magaramkent, Primorskiy; locality: In 3 km NW of the village Primorskiy, Dagestan Nature Reserve; verbatimElevation: 16 m; minimumElevationInMeters: 16; decimalLatitude: 41.86472; decimalLongitude: 48.61083; **Identification:** identifiedBy: V.I. Lantsov; **Event:** samplingProtocol: Sweep net; eventDate: 2014-05-17; verbatimEventDate: 17/May/2014; habitat: Samur liana forest, hornbeam and spurge liana forest, with an admixture of oak, maple and high ash - elderberry, horsetail, sweeping on plants along the stream; **Record Level:** institutionCode: ZIN; basisOfRecord: PreservedSpecimen

#### Distribution

First records from Portugal and Russia: RUE, NC.

### Limonia interjecta

Starý, 1974

B82C6B8F-D5A9-589A-B8E8-7AC7AFF5B42D

https://ccw.naturalis.nl/detail.php?id=10581

#### Materials

- **Type status:** Other material. **Occurrence:** catalogNumber: 529133; occurrenceRemarks: 1 male; recordedBy: Ř. Gammelmo; individualCount: 1; sex: male; preparations: Ethanol; occurrenceID: EU_LIM_491; **Taxon:** scientificName: Limoniainterjecta Starý, 1974; family: Limoniidae; genus: Limonia; specificEpithet: interjecta; scientificNameAuthorship: Starý, 1974; **Location:** country: Norway; stateProvince: Troms; municipality: Nordreisa; locality: Veibrink – Both south and north of E6; verbatimElevation: 205-250 m; minimumElevationInMeters: 205; maximumElevationInMeters: 250; decimalLatitude: 69.81725; decimalLongitude: 20.86584; **Identification:** identifiedBy: J. Starý; **Event:** samplingProtocol: Sweep net; eventDate: 2017-06-12; verbatimEventDate: 12/Jul/2017; **Record Level:** institutionCode: PCKMO; basisOfRecord: PreservedSpecimen
- **Type status:** Other material. **Occurrence:** catalogNumber: 564567; occurrenceRemarks: 2 males; recordedBy: K.M. Olsen | S. Olsen; individualCount: 2; sex: male; preparations: Ethanol; occurrenceID: EU_LIM_492; **Taxon:** scientificName: Limoniainterjecta Starý, 1974; family: Limoniidae; genus: Limonia; specificEpithet: interjecta; scientificNameAuthorship: Starý, 1974; **Location:** country: Norway; stateProvince: Finnmark; municipality: Alta; locality: Grønnåsen S; verbatimElevation: 330-482 m; minimumElevationInMeters: 330; maximumElevationInMeters: 482; decimalLatitude: 69.78945; decimalLongitude: 23.5455; **Identification:** identifiedBy: K.M. Olsen; **Event:** samplingProtocol: Sweep net; eventDate: 2017-06-16; verbatimEventDate: 16/Jul/2017; habitat: Mellom veien og søndre topp; **Record Level:** institutionCode: PCKMO; basisOfRecord: PreservedSpecimen
- **Type status:** Other material. **Occurrence:** catalogNumber: 647849, 647850; occurrenceRemarks: 8 male+female; recordedBy: Rikmyrsprosjektet; individualCount: 8; sex: male, female; preparations: Ethanol; occurrenceID: EU_LIM_493; **Taxon:** scientificName: Limoniainterjecta Starý, 1974; family: Limoniidae; genus: Limonia; specificEpithet: interjecta; scientificNameAuthorship: Starý, 1974; **Location:** country: Norway; stateProvince: Hedmark; municipality: Tolga; locality: N Bjørvollen; verbatimElevation: 770 m; minimumElevationInMeters: 770; decimalLatitude: 62.38703; decimalLongitude: 11.11887; **Identification:** identifiedBy: K.M. Olsen; **Event:** samplingProtocol: Window trap; eventDate: 2016-06-01/2016-07-01; verbatimEventDate: 01/Jun-16/Jul/2016; habitat: Near Malaise trap 7; **Record Level:** institutionCode: ZMUB | PCKMO; basisOfRecord: PreservedSpecimen
- **Type status:** Other material. **Occurrence:** catalogNumber: 647854; occurrenceRemarks: 3 male+female; recordedBy: Rikmyrsprosjektet; individualCount: 3; sex: male, female; preparations: Ethanol; occurrenceID: EU_LIM_494; **Taxon:** scientificName: Limoniainterjecta Starý, 1974; family: Limoniidae; genus: Limonia; specificEpithet: interjecta; scientificNameAuthorship: Starý, 1974; **Location:** country: Norway; stateProvince: Hedmark; municipality: Tolga; locality: N Bjørvollen; verbatimElevation: 770 m; minimumElevationInMeters: 770; decimalLatitude: 62.38703; decimalLongitude: 11.11887; **Identification:** identifiedBy: K.M. Olsen; **Event:** samplingProtocol: Window trap; eventDate: 2016-06-16/2016-09-19; verbatimEventDate: 16/Jul-19/Sep/2016; habitat: Near Malaise trap 7; **Record Level:** institutionCode: ZMUB; basisOfRecord: PreservedSpecimen
- **Type status:** Other material. **Occurrence:** catalogNumber: 647922; occurrenceRemarks: 1 male; recordedBy: K.M. Olsen; individualCount: 1; sex: male; preparations: Ethanol; occurrenceID: EU_LIM_495; **Taxon:** scientificName: Limoniainterjecta Starý, 1974; family: Limoniidae; genus: Limonia; specificEpithet: interjecta; scientificNameAuthorship: Starý, 1974; **Location:** country: Norway; stateProvince: Sør-Trøndelag; municipality: Oppdal; locality: Kongsvoll; verbatimElevation: 880 m; minimumElevationInMeters: 880; decimalLatitude: 62.30573; decimalLongitude: 9.60587; **Identification:** identifiedBy: K.M. Olsen; **Event:** samplingProtocol: Sweep net; eventDate: 2019-06-11; verbatimEventDate: 11/Jul/2019; **Record Level:** institutionCode: NHMO; basisOfRecord: PreservedSpecimen
- **Type status:** Other material. **Occurrence:** occurrenceRemarks: 1 male; recordedBy: L.-P. Kolcsár; individualCount: 1; sex: male; preparations: Ethanol; occurrenceID: EU_LIM_496; **Taxon:** scientificName: Limoniainterjecta Starý, 1974; family: Limoniidae; genus: Limonia; specificEpithet: interjecta; scientificNameAuthorship: Starý, 1974; **Location:** country: Romania; stateProvince: Brașov; municipality: Sâmbăta de Sus; locality: Făgăraș Mts., Sâmbăta Valley; verbatimElevation: 1000 m; minimumElevationInMeters: 1000; decimalLatitude: 45.65241; decimalLongitude: 24.78867; **Identification:** identifiedBy: L.-P. Kolcsár; **Event:** samplingProtocol: Sweep net; eventDate: 2017-05-29; verbatimEventDate: 29/May/2017; **Record Level:** institutionCode: CKLP; basisOfRecord: PreservedSpecimen

#### Distribution

First record from Romania. This species was denoted uncertain for Norway in Olsen et al. (2018). Here, we confirm the presence of the species in Norway.

### Limonia macrostigma

(Schummel, 1829)

B0276E97-1144-54F6-BA06-B6AEFC6BBD37

https://ccw.naturalis.nl/detail.php?id=10597

#### Materials

- **Type status:** Other material. **Occurrence:** occurrenceRemarks: 1 female; recordedBy: D.I. Gavryushin; individualCount: 1; sex: female; preparations: Pinned; occurrenceID: EU_LIM_497; **Taxon:** scientificName: Limoniamacrostigma (Schummel, 1829); family: Limoniidae; genus: Limonia; specificEpithet: macrostigma; scientificNameAuthorship: (Schummel, 1829); **Location:** country: Belarus; stateProvince: Minsk; county: Barysaw; locality: Vialikaje Stachava; verbatimElevation: 156 m; minimumElevationInMeters: 156; decimalLatitude: 54.26555; decimalLongitude: 28.38332; **Identification:** identifiedBy: D.I. Gavryushin; **Event:** samplingProtocol: Sweep net; eventDate: 2013-06-07; verbatimEventDate: 7/Jul/2013; **Record Level:** institutionCode: ZMMU; basisOfRecord: PreservedSpecimen
- **Type status:** Other material. **Occurrence:** occurrenceRemarks: 1 male; recordedBy: D.I. Gavryushin; individualCount: 1; sex: male; preparations: Pinned; occurrenceID: EU_LIM_498; **Taxon:** scientificName: Limoniamacrostigma (Schummel, 1829); family: Limoniidae; genus: Limonia; specificEpithet: macrostigma; scientificNameAuthorship: (Schummel, 1829); **Location:** country: Belarus; stateProvince: Vitebsk; county: Orsha; locality: Svyatoe Lake; decimalLatitude: 54.686; decimalLongitude: 30.442; **Identification:** identifiedBy: D.I. Gavryushin; **Event:** samplingProtocol: Sweep net; eventDate: 2017-06-12; verbatimEventDate: 12/Jun/2017; **Record Level:** institutionCode: ZMMU; basisOfRecord: PreservedSpecimen

#### Distribution

First records from Belarus.

### Limonia nigropunctata

(Schummel, 1829)

052914EA-F3F4-53E7-B9C1-5A6684398289

https://ccw.naturalis.nl/detail.php?id=10621

#### Materials

- **Type status:** Other material. **Occurrence:** occurrenceRemarks: 1 male; recordedBy: D.I. Gavryushin; individualCount: 1; sex: male; preparations: Ethanol; occurrenceID: EU_LIM_501; **Taxon:** scientificName: Limonianigropunctata nigropunctata (Schummel, 1829); family: Limoniidae; genus: Limonia; specificEpithet: nigropunctata; infraspecificEpithet: nigropunctata; scientificNameAuthorship: (Schummel, 1829); **Location:** country: Belarus; stateProvince: Gomel; county: Mazyr; locality: Mazyr; decimalLatitude: 52.02; decimalLongitude: 29.32; **Identification:** identifiedBy: D.I. Gavryushin; **Event:** samplingProtocol: Sweep net; eventDate: 2019-05-19/2019-05-21; verbatimEventDate: 19-21/May/2019; **Record Level:** institutionCode: ZMMU; basisOfRecord: PreservedSpecimen
- **Type status:** Other material. **Occurrence:** occurrenceRemarks: 1 male; recordedBy: D.I. Gavryushin; individualCount: 1; sex: male; preparations: Pinned; occurrenceID: EU_LIM_502; **Taxon:** scientificName: Limonianigropunctata nigropunctata (Schummel, 1829); family: Limoniidae; genus: Limonia; specificEpithet: nigropunctata; infraspecificEpithet: nigropunctata; scientificNameAuthorship: (Schummel, 1829); **Location:** country: Belarus; stateProvince: Gomel; county: Mazyr; locality: 35 km E Mazyr; decimalLatitude: 52.173; decimalLongitude: 29.79; **Identification:** identifiedBy: D.I. Gavryushin; **Event:** samplingProtocol: Sweep net; eventDate: 2019-05-18; verbatimEventDate: 18/May/2019; **Record Level:** institutionCode: ZMMU; basisOfRecord: PreservedSpecimen

#### Distribution

First records from Belarus.

### Limonia nubeculosa

Meigen, 1804

BC0C086B-F83F-5B25-8A7C-EEC17C1F6759

https://ccw.naturalis.nl/detail.php?id=10626

#### Materials

- **Type status:** Other material. **Occurrence:** occurrenceRemarks: 1 male; recordedBy: D.I. Gavryushin; individualCount: 1; sex: male; preparations: Pinned; occurrenceID: EU_LIM_503; **Taxon:** scientificName: Limonianubeculosa Meigen, 1804; family: Limoniidae; genus: Limonia; specificEpithet: nubeculosa; scientificNameAuthorship: Meigen, 1804; **Location:** country: Belarus; stateProvince: Gomel; county: Mazyr; locality: Mazyr; decimalLatitude: 52.02; decimalLongitude: 29.3; **Identification:** identifiedBy: D.I. Gavryushin; **Event:** samplingProtocol: Sweep net; eventDate: 2019-06-29/2019-07-31; verbatimEventDate: 29-31/Jul/2019; **Record Level:** institutionCode: ZMMU; basisOfRecord: PreservedSpecimen
- **Type status:** Other material. **Occurrence:** occurrenceRemarks: 1 male; recordedBy: A. Valletta; individualCount: 1; sex: male; preparations: Pinned; occurrenceID: EU_LIM_504; **Taxon:** scientificName: Limonianubeculosa Meigen, 1804; family: Limoniidae; genus: Limonia; specificEpithet: nubeculosa; scientificNameAuthorship: Meigen, 1804; **Location:** country: Malta; decimalLatitude: 35.85; decimalLongitude: 14.46; **Identification:** identifiedBy: P. Oosterbroek; **Event:** eventDate: 1979-02-26; verbatimEventDate: 26/Feb/1979; **Record Level:** institutionCode: NBCN; basisOfRecord: PreservedSpecimen
- **Type status:** Other material. **Occurrence:** occurrenceRemarks: 1 male; recordedBy: A. Valletta; individualCount: 1; sex: male; preparations: Pinned; occurrenceID: EU_LIM_505; **Taxon:** scientificName: Limonianubeculosa Meigen, 1804; family: Limoniidae; genus: Limonia; specificEpithet: nubeculosa; scientificNameAuthorship: Meigen, 1804; **Location:** country: Malta; decimalLatitude: 35.85; decimalLongitude: 14.46; **Identification:** identifiedBy: P. Oosterbroek; **Event:** eventDate: 1979-04-10; verbatimEventDate: 10/Apr/1979; **Record Level:** institutionCode: NBCN; basisOfRecord: PreservedSpecimen

#### Distribution

First records from Belarus and Malta.

### Limonia phragmitidis

(Schrank, 1781)

C0E4E7D2-6445-566A-9BFF-DE8759AF5580

https://ccw.naturalis.nl/detail.php?id=10646

#### Materials

- **Type status:** Other material. **Occurrence:** occurrenceRemarks: 1 male; recordedBy: L.-P. Kolcsár; individualCount: 1; sex: male; preparations: Ethanol; occurrenceID: EU_LIM_506; **Taxon:** scientificName: Limoniaphragmitidis (Schrank, 1781); family: Limoniidae; genus: Limonia; specificEpithet: phragmitidis; scientificNameAuthorship: (Schrank, 1781); **Location:** country: Estonia; stateProvince: Pärnu; municipality: Häädemeeste; verbatimElevation: 5 m; minimumElevationInMeters: 5; decimalLatitude: 58.0735; decimalLongitude: 24.4931; **Identification:** identifiedBy: L.-P. Kolcsár; **Event:** samplingProtocol: Sweep net; eventDate: 2018-06-26; verbatimEventDate: 26/Jul/2018; habitat: small stream near to Baltic sea; **Record Level:** institutionCode: CKLP; basisOfRecord: PreservedSpecimen

#### Distribution

First record from Estonia.

### Limonia stigma

(Meigen, 1818)

61CB3080-BD9E-5E7F-8794-3F3CD1E16D03

https://ccw.naturalis.nl/detail.php?id=10672

#### Materials

- **Type status:** Other material. **Occurrence:** occurrenceRemarks: 2 males, 1 female; recordedBy: D.I. Gavryushin; individualCount: 3; sex: male, female; preparations: Pinned; occurrenceID: EU_LIM_507; **Taxon:** scientificName: Limoniastigma (Meigen, 1818); family: Limoniidae; genus: Limonia; specificEpithet: stigma; scientificNameAuthorship: (Meigen, 1818); **Location:** country: Belarus; stateProvince: Minsk; county: Barysaw; locality: Vialikaje Stachava; verbatimElevation: 156 m; minimumElevationInMeters: 156; decimalLatitude: 54.26555; decimalLongitude: 28.38332; **Identification:** identifiedBy: D.I. Gavryushin; **Event:** samplingProtocol: Sweep net; eventDate: 2013-06-07; verbatimEventDate: 7/Jul/2013; **Record Level:** institutionCode: ZMMU; basisOfRecord: PreservedSpecimen
- **Type status:** Other material. **Occurrence:** occurrenceRemarks: 1 male; recordedBy: L.-P. Kolcsár; individualCount: 1; sex: male; preparations: Ethanol; occurrenceID: EU_LIM_508; **Taxon:** scientificName: Limoniastigma (Meigen, 1818); family: Limoniidae; genus: Limonia; specificEpithet: stigma; scientificNameAuthorship: (Meigen, 1818); **Location:** country: Latvia; municipality: Sigulda; locality: Gauja River; verbatimElevation: 13 m; minimumElevationInMeters: 13; decimalLatitude: 57.1505; decimalLongitude: 24.8168; **Identification:** identifiedBy: L.-P. Kolcsár; **Event:** samplingProtocol: Sweep net; eventDate: 2018-06-24; verbatimEventDate: 24/Jul/2018; **Record Level:** institutionCode: CKLP; basisOfRecord: PreservedSpecimen
- **Type status:** Other material. **Occurrence:** occurrenceRemarks: 1 male; recordedBy: D.I. Gavryushin; individualCount: 1; sex: male; occurrenceID: EU_LIM_509; **Taxon:** scientificName: Limoniastigma (Meigen, 1818); family: Limoniidae; genus: Limonia; specificEpithet: stigma; scientificNameAuthorship: (Meigen, 1818); **Location:** country: Russia; stateProvince: East European Russia; county: Bashkortostan Respublika; municipality: Beloretsk district; locality: Abzakovo env., Malyi Kizil River; verbatimElevation: 510 m; minimumElevationInMeters: 510; decimalLatitude: 53.81428; decimalLongitude: 58.5942; **Identification:** identifiedBy: D.I. Gavryushin; **Event:** samplingProtocol: Sweep net; eventDate: 2015-06-12; verbatimEventDate: 12/Jul/2015; **Record Level:** institutionCode: ZMMU; basisOfRecord: PreservedSpecimen
- **Type status:** Other material. **Occurrence:** occurrenceRemarks: 1 male; recordedBy: D.I. Gavryushin; individualCount: 1; sex: male; occurrenceID: EU_LIM_510; **Taxon:** scientificName: Limoniastigma (Meigen, 1818); family: Limoniidae; genus: Limonia; specificEpithet: stigma; scientificNameAuthorship: (Meigen, 1818); **Location:** country: Russia; stateProvince: East European Russia; county: Bashkortostan Respublika; municipality: Beloretsk district; locality: Abzakovo env., Kulsugady River; verbatimElevation: 531 m; minimumElevationInMeters: 531; decimalLatitude: 53.83795; decimalLongitude: 58.5823; **Identification:** identifiedBy: D.I. Gavryushin; **Event:** samplingProtocol: Sweep net; eventDate: 2015-06-17; verbatimEventDate: 17/Jul/2015; **Record Level:** institutionCode: ZMMU; basisOfRecord: PreservedSpecimen
- **Type status:** Other material. **Occurrence:** occurrenceRemarks: 1 female; recordedBy: D.I. Gavryushin; individualCount: 1; sex: female; occurrenceID: EU_LIM_511; **Taxon:** scientificName: Limoniastigma (Meigen, 1818); family: Limoniidae; genus: Limonia; specificEpithet: stigma; scientificNameAuthorship: (Meigen, 1818); **Location:** country: Russia; stateProvince: East European Russia; county: Bashkortostan Respublika; municipality: Beloretsk district; locality: Nura River (ca. 4km W of Otnurok village), at the foot of Zolotyie Shishki (Golden Cones) Mts.; verbatimElevation: 607 m; minimumElevationInMeters: 607; decimalLatitude: 54.05155; decimalLongitude: 58.26887; **Identification:** identifiedBy: D.I. Gavryushin; **Event:** samplingProtocol: Sweep net; eventDate: 2015-06-10; verbatimEventDate: 10/Jul/2015; **Record Level:** institutionCode: ZMMU; basisOfRecord: PreservedSpecimen
- **Type status:** Other material. **Occurrence:** occurrenceRemarks: 2 males; recordedBy: D.I. Gavryushin; individualCount: 2; sex: male; occurrenceID: EU_LIM_512; **Taxon:** scientificName: Limoniastigma (Meigen, 1818); family: Limoniidae; genus: Limonia; specificEpithet: stigma; scientificNameAuthorship: (Meigen, 1818); **Location:** country: Russia; stateProvince: East European Russia; county: Bashkortostan Respublika; municipality: Beloretsk district; locality: Makhmutovo env., Belaya River; verbatimElevation: 550 m; minimumElevationInMeters: 550; decimalLatitude: 54.33012; decimalLongitude: 58.80735; **Identification:** identifiedBy: D.I. Gavryushin; **Event:** samplingProtocol: Sweep net; eventDate: 2015-06-15; verbatimEventDate: 15/Jul/2015; **Record Level:** institutionCode: ZMMU; basisOfRecord: PreservedSpecimen
- **Type status:** Other material. **Occurrence:** occurrenceRemarks: 1 female; recordedBy: K.P. Tomkovich; individualCount: 1; sex: female; occurrenceID: EU_LIM_513; **Taxon:** scientificName: Limoniastigma (Meigen, 1818); family: Limoniidae; genus: Limonia; specificEpithet: stigma; scientificNameAuthorship: (Meigen, 1818); **Location:** country: Russia; stateProvince: Central European Russia; county: Moskovskaya Oblast; municipality: Moscow; locality: Tsaritsino; verbatimElevation: 150 m; minimumElevationInMeters: 150; decimalLatitude: 55.60696; decimalLongitude: 37.68537; **Identification:** identifiedBy: V.E. Pilipenko; **Event:** samplingProtocol: Sweep net; eventDate: 2008-08-13; verbatimEventDate: 13/Aug/2008; **Record Level:** institutionCode: VPMC; basisOfRecord: PreservedSpecimen

#### Distribution

First records from Belarus, Latvia and Russia: RUC, RUE.

### Limonia sylvicola

(Schummel, 1829)

CBCECE91-BF53-5054-B886-A0C99407EFE2

https://ccw.naturalis.nl/detail.php?id=10682

#### Materials

- **Type status:** Other material. **Occurrence:** occurrenceRemarks: 1 female; recordedBy: D.I. Gavryushin; individualCount: 1; sex: female; occurrenceID: EU_LIM_514; **Taxon:** scientificName: Limoniasylvicola (Schummel, 1829); family: Limoniidae; genus: Limonia; specificEpithet: sylvicola; scientificNameAuthorship: (Schummel, 1829); **Location:** country: Russia; stateProvince: East European Russia; county: Bashkortostan Respublika; municipality: Beloretsk district; locality: Nura River (ca. 4km W of Otnurok village), at the foot of Zolotyie Shishki (Golden Cones) Mts.; verbatimElevation: 607 m; minimumElevationInMeters: 607; decimalLatitude: 54.05155; decimalLongitude: 58.26887; **Identification:** identifiedBy: D.I. Gavryushin; **Event:** samplingProtocol: Sweep net; eventDate: 2012-08-08; verbatimEventDate: 08/Aug/2012; **Record Level:** institutionCode: ZMMU; basisOfRecord: PreservedSpecimen
- **Type status:** Other material. **Occurrence:** occurrenceRemarks: 1 female; recordedBy: K.P. Tomkovich; individualCount: 1; sex: female; occurrenceID: EU_LIM_515; **Taxon:** scientificName: Limoniasylvicola (Schummel, 1829); family: Limoniidae; genus: Limonia; specificEpithet: sylvicola; scientificNameAuthorship: (Schummel, 1829); **Location:** country: Russia; stateProvince: East European Russia; county: Bashkortostan Respublika; municipality: Beloretsk district; locality: S. Ural, Abzakovo-Murakaevo, Kryktytau Mt.; verbatimElevation: 700 m; minimumElevationInMeters: 700; decimalLatitude: 53.54548; decimalLongitude: 58.48029; **Identification:** identifiedBy: V.E. Pilipenko; **Event:** samplingProtocol: Sweep net; eventDate: 2008-08-02/2008-08-08; verbatimEventDate: 2-8/Aug/2008; **Record Level:** basisOfRecord: PreservedSpecimen
- **Type status:** Other material. **Occurrence:** occurrenceRemarks: 1 male; recordedBy: V.E. Pilipenko; individualCount: 1; sex: male; occurrenceID: EU_LIM_516; **Taxon:** scientificName: Limoniasylvicola (Schummel, 1829); family: Limoniidae; genus: Limonia; specificEpithet: sylvicola; scientificNameAuthorship: (Schummel, 1829); **Location:** country: Russia; stateProvince: Central European Russia; county: Moskovskaya Oblast; municipality: Solnechnogorsk district; locality: Chashnikovo; verbatimElevation: 220 m; minimumElevationInMeters: 220; decimalLatitude: 56.0375; decimalLongitude: 37.1874; **Identification:** identifiedBy: V.E. Pilipenko; **Event:** samplingProtocol: Sweep net; eventDate: 1994-06-22; verbatimEventDate: 22/Jul/1994; **Record Level:** institutionCode: VPMC; basisOfRecord: PreservedSpecimen
- **Type status:** Other material. **Occurrence:** occurrenceRemarks: 1 female; recordedBy: V.E. Pilipenko; individualCount: 1; sex: female; occurrenceID: EU_LIM_517; **Taxon:** scientificName: Limoniasylvicola (Schummel, 1829); family: Limoniidae; genus: Limonia; specificEpithet: sylvicola; scientificNameAuthorship: (Schummel, 1829); **Location:** country: Russia; stateProvince: Central European Russia; county: Moskovskaya Oblast; municipality: Solnechnogorsk district; locality: Chashnikovo; verbatimElevation: 220 m; minimumElevationInMeters: 220; decimalLatitude: 56.0375; decimalLongitude: 37.1874; **Identification:** identifiedBy: V.E. Pilipenko; **Event:** samplingProtocol: Sweep net; eventDate: 1994-08-14; verbatimEventDate: 14/Aug/1994; **Record Level:** institutionCode: VPMC; basisOfRecord: PreservedSpecimen
- **Type status:** Other material. **Occurrence:** occurrenceRemarks: 1 female; recordedBy: V.E. Pilipenko; individualCount: 1; sex: female; occurrenceID: EU_LIM_518; **Taxon:** scientificName: Limoniasylvicola (Schummel, 1829); family: Limoniidae; genus: Limonia; specificEpithet: sylvicola; scientificNameAuthorship: (Schummel, 1829); **Location:** country: Russia; stateProvince: Central European Russia; county: Moskovskaya Oblast; municipality: Solnechnogorsk district; locality: Chashnikovo; verbatimElevation: 220 m; minimumElevationInMeters: 220; decimalLatitude: 56.0375; decimalLongitude: 37.1874; **Identification:** identifiedBy: V.E. Pilipenko; **Event:** samplingProtocol: Sweep net; eventDate: 1994-08-16; verbatimEventDate: 16/Aug/1994; **Record Level:** institutionCode: VPMC; basisOfRecord: PreservedSpecimen

#### Distribution

First records from Russia: RUC, RUE.

### Limonia trivittata

(Schummel, 1829)

6CBBB423-8FB7-5FB6-AF1C-57394A175058

https://ccw.naturalis.nl/detail.php?id=10697

#### Materials

- **Type status:** Other material. **Occurrence:** occurrenceRemarks: 1 female; recordedBy: D.I. Gavryushin; individualCount: 1; sex: female; preparations: Pinned; occurrenceID: EU_LIM_519; **Taxon:** scientificName: Limoniatrivittata (Schummel, 1829); family: Limoniidae; genus: Limonia; specificEpithet: trivittata; scientificNameAuthorship: (Schummel, 1829); **Location:** country: Belarus; stateProvince: Minsk; county: Barysaw; locality: Vialikaje Stachava; verbatimElevation: 156 m; minimumElevationInMeters: 156; decimalLatitude: 54.26555; decimalLongitude: 28.38332; **Identification:** identifiedBy: D.I. Gavryushin; **Event:** samplingProtocol: Sweep net; eventDate: 2013-06-07; verbatimEventDate: 7/Jul/2013; **Record Level:** institutionCode: ZMMU; basisOfRecord: PreservedSpecimen
- **Type status:** Other material. **Occurrence:** occurrenceRemarks: 1 female; recordedBy: D.I. Gavryushin; individualCount: 1; sex: female; occurrenceID: EU_LIM_520; **Taxon:** scientificName: Limoniatrivittata (Schummel, 1829); family: Limoniidae; genus: Limonia; specificEpithet: trivittata; scientificNameAuthorship: (Schummel, 1829); **Location:** country: Russia; stateProvince: East European Russia; county: Bashkortostan Respublika; municipality: Beloretsk district; locality: Abzakovo env., Karan River; verbatimElevation: 533 m; minimumElevationInMeters: 533; decimalLatitude: 53.83717; decimalLongitude: 58.57878; **Identification:** identifiedBy: D.I. Gavryushin; **Event:** samplingProtocol: Sweep net; eventDate: 2015-06-17; verbatimEventDate: 17/Jul/2015; **Record Level:** institutionCode: ZMMU; basisOfRecord: PreservedSpecimen
- **Type status:** Other material. **Occurrence:** occurrenceRemarks: 1 female; recordedBy: D.I. Gavryushin; individualCount: 1; sex: female; occurrenceID: EU_LIM_521; **Taxon:** scientificName: Limoniatrivittata (Schummel, 1829); family: Limoniidae; genus: Limonia; specificEpithet: trivittata; scientificNameAuthorship: (Schummel, 1829); **Location:** country: Russia; stateProvince: East European Russia; county: Bashkortostan Respublika; municipality: Beloretsk district; locality: Abzakovo env., Kulsugady River; verbatimElevation: 531 m; minimumElevationInMeters: 531; decimalLatitude: 53.83795; decimalLongitude: 58.5823; **Identification:** identifiedBy: D.I. Gavryushin; **Event:** samplingProtocol: Sweep net; eventDate: 2015-06-17; verbatimEventDate: 17/Jul/2015; **Record Level:** institutionCode: ZMMU; basisOfRecord: PreservedSpecimen
- **Type status:** Other material. **Occurrence:** occurrenceRemarks: 1 female; recordedBy: D.I. Gavryushin; individualCount: 1; sex: female; occurrenceID: EU_LIM_522; **Taxon:** scientificName: Limoniatrivittata (Schummel, 1829); family: Limoniidae; genus: Limonia; specificEpithet: trivittata; scientificNameAuthorship: (Schummel, 1829); **Location:** country: Russia; stateProvince: East European Russia; county: Bashkortostan Respublika; municipality: Beloretsk district; locality: Nura River (ca. 4km W of Otnurok village), at the foot of Zolotyie Shishki (Golden Cones) Mts.; verbatimElevation: 607 m; minimumElevationInMeters: 607; decimalLatitude: 54.05155; decimalLongitude: 58.26887; **Identification:** identifiedBy: D.I. Gavryushin; **Event:** samplingProtocol: Sweep net; eventDate: 2012-08-09; verbatimEventDate: 09/Aug/2012; **Record Level:** institutionCode: ZMMU; basisOfRecord: PreservedSpecimen
- **Type status:** Other material. **Occurrence:** occurrenceRemarks: 1 male; recordedBy: N.M. Paramonov; individualCount: 1; sex: male; occurrenceID: EU_LIM_523; **Taxon:** scientificName: Limoniatrivittata (Schummel, 1829); family: Limoniidae; genus: Limonia; specificEpithet: trivittata; scientificNameAuthorship: (Schummel, 1829); **Location:** country: Russia; stateProvince: East European Russia; county: Tatarstan Respublika; municipality: Zelenodol’sk district; locality: Zaymishche env., Geomagnetic station; verbatimElevation: 87 m; minimumElevationInMeters: 87; decimalLatitude: 55.82684; decimalLongitude: 48.84395; **Identification:** identifiedBy: N.M. Paramonov; **Event:** samplingProtocol: Sweep net; eventDate: 2012-06-30; verbatimEventDate: 30/Jun/2012; **Record Level:** institutionCode: ZIN; basisOfRecord: PreservedSpecimen

#### Distribution

First records from Belarus and Russia: RUE.

### Lipsothrix ecucullata

Edwards, 1938

3390367E-9C89-5367-92F2-D77D00C47D2B

https://ccw.naturalis.nl/detail.php?id=10756

#### Materials

- **Type status:** Other material. **Occurrence:** occurrenceRemarks: 1 male, 2 females; recordedBy: D.I. Gavryushin; individualCount: 3; sex: male, female; occurrenceID: EU_LIM_524; **Taxon:** scientificName: Lipsothrixecucullata Edwards, 1938; family: Limoniidae; genus: Lipsothrix; specificEpithet: ecucullata; scientificNameAuthorship: Edwards, 1938; **Location:** country: Russia; stateProvince: East European Russia; county: Bashkortostan Respublika; municipality: Beloretsk district; locality: Nura River (ca. 4km W of Otnurok village), at the foot of Zolotyie Shishki (Golden Cones) Mts.; verbatimElevation: 607 m; minimumElevationInMeters: 607; decimalLatitude: 54.05155; decimalLongitude: 58.26887; **Identification:** identifiedBy: D.I. Gavryushin; **Event:** samplingProtocol: Sweep net; eventDate: 2015-06-10; verbatimEventDate: 10/Jul/2015; **Record Level:** institutionCode: ZMMU; basisOfRecord: PreservedSpecimen
- **Type status:** Other material. **Occurrence:** occurrenceRemarks: 1 female; recordedBy: D.I. Gavryushin; individualCount: 1; sex: female; occurrenceID: EU_LIM_525; **Taxon:** scientificName: Lipsothrixecucullata Edwards, 1938; family: Limoniidae; genus: Lipsothrix; specificEpithet: ecucullata; scientificNameAuthorship: Edwards, 1938; **Location:** country: Russia; stateProvince: East European Russia; county: Bashkortostan Respublika; municipality: Beloretsk district; locality: Nura River (ca. 4km W of Otnurok village), at the foot of Zolotyie Shishki (Golden Cones) Mts.; verbatimElevation: 607 m; minimumElevationInMeters: 607; decimalLatitude: 54.05155; decimalLongitude: 58.26887; **Identification:** identifiedBy: D.I. Gavryushin; **Event:** samplingProtocol: Sweep net; eventDate: 2015-06-13; verbatimEventDate: 13/Jul/2015; **Record Level:** institutionCode: ZMMU; basisOfRecord: PreservedSpecimen
- **Type status:** Other material. **Occurrence:** occurrenceRemarks: 1 male; recordedBy: V.E. Pilipenko; individualCount: 1; sex: male; occurrenceID: EU_LIM_526; **Taxon:** scientificName: Lipsothrixecucullata Edwards, 1938; family: Limoniidae; genus: Lipsothrix; specificEpithet: ecucullata; scientificNameAuthorship: Edwards, 1938; **Location:** country: Russia; stateProvince: Central European Russia; county: Moskovskaya Oblast; municipality: Solnechnogorsk district; locality: Chashnikovo; verbatimElevation: 220 m; minimumElevationInMeters: 220; decimalLatitude: 56.0375; decimalLongitude: 37.1874; **Identification:** identifiedBy: V.E. Pilipenko; **Event:** samplingProtocol: Sweep net; eventDate: 1996-06-15; verbatimEventDate: 15/Jun/1996; **Record Level:** institutionCode: VPMC; basisOfRecord: PreservedSpecimen
- **Type status:** Other material. **Occurrence:** occurrenceRemarks: 1 male, 1 female; recordedBy: V.E. Pilipenko; individualCount: 2; sex: male, female; occurrenceID: EU_LIM_527; **Taxon:** scientificName: Lipsothrixecucullata Edwards, 1938; family: Limoniidae; genus: Lipsothrix; specificEpithet: ecucullata; scientificNameAuthorship: Edwards, 1938; **Location:** country: Russia; stateProvince: Central European Russia; county: Moskovskaya Oblast; municipality: Solnechnogorsk district; locality: Chashnikovo; verbatimElevation: 220 m; minimumElevationInMeters: 220; decimalLatitude: 56.0375; decimalLongitude: 37.1874; **Identification:** identifiedBy: V.E. Pilipenko; **Event:** samplingProtocol: Sweep net; eventDate: 1997-06-26; verbatimEventDate: 26/Jun/1997; **Record Level:** institutionCode: VPMC; basisOfRecord: PreservedSpecimen
- **Type status:** Other material. **Occurrence:** occurrenceRemarks: 1 male; recordedBy: V.E. Pilipenko; individualCount: 1; sex: male; occurrenceID: EU_LIM_528; **Taxon:** scientificName: Lipsothrixecucullata Edwards, 1938; family: Limoniidae; genus: Lipsothrix; specificEpithet: ecucullata; scientificNameAuthorship: Edwards, 1938; **Location:** country: Russia; stateProvince: Central European Russia; county: Moskovskaya Oblast; municipality: Solnechnogorsk district; locality: Chashnikovo; verbatimElevation: 220 m; minimumElevationInMeters: 220; decimalLatitude: 56.0375; decimalLongitude: 37.1874; **Identification:** identifiedBy: V.E. Pilipenko; **Event:** samplingProtocol: Sweep net; eventDate: 1997-08-15; verbatimEventDate: 15/Aug/1997; **Record Level:** institutionCode: VPMC; basisOfRecord: PreservedSpecimen
- **Type status:** Other material. **Occurrence:** occurrenceRemarks: 1 male; recordedBy: A. Polevoi; individualCount: 1; sex: male; preparations: Pinned; occurrenceID: EU_LIM_529; **Taxon:** scientificName: Lipsothrixecucullata Edwards, 1938; family: Limoniidae; genus: Lipsothrix; specificEpithet: ecucullata; scientificNameAuthorship: Edwards, 1938; **Location:** country: Russia; stateProvince: North European Russia; county: Republic Karelia; municipality: Kondopoga district; locality: Tereki, 5 km SE; verbatimElevation: 130 m; minimumElevationInMeters: 130; decimalLatitude: 62.21332; decimalLongitude: 33.87466; **Identification:** identifiedBy: A. Polevoi; **Event:** samplingProtocol: Sweep net; eventDate: 2018-06-25; verbatimEventDate: 25/Jun/2018; **Record Level:** institutionCode: FRIP; basisOfRecord: PreservedSpecimen

#### Distribution

First records from Russia: RUC, RUE, RUN.

### Lipsothrix errans

(Walker, 1848)

E4FB9E88-5D86-5C99-9EDE-50E2E0B94DE5

https://ccw.naturalis.nl/detail.php?id=10757

#### Materials

- **Type status:** Other material. **Occurrence:** occurrenceRemarks: 1 male; recordedBy: L. Papp; individualCount: 1; sex: male; preparations: Pinned; occurrenceID: EU_LIM_530; **Taxon:** scientificName: Lipsothrixerrans (Walker, 1848); family: Limoniidae; genus: Lipsothrix; specificEpithet: errans; scientificNameAuthorship: (Walker, 1848); **Location:** country: Hungary; stateProvince: Baranya; municipality: Pécs; locality: Melegmány Protected Area, Melegmány Valley; decimalLatitude: 46.11808; decimalLongitude: 18.21515; **Identification:** identifiedBy: L.-P. Kolcsár; **Event:** eventDate: 2002-05-29; verbatimEventDate: 29/May/2002; **Record Level:** institutionCode: HNHM; basisOfRecord: PreservedSpecimen
- **Type status:** Other material. **Occurrence:** occurrenceRemarks: 1 male; recordedBy: L. Papp; individualCount: 1; sex: male; preparations: Pinned; occurrenceID: EU_LIM_531; **Taxon:** scientificName: Lipsothrixerrans (Walker, 1848); family: Limoniidae; genus: Lipsothrix; specificEpithet: errans; scientificNameAuthorship: (Walker, 1848); **Location:** country: Hungary; stateProvince: Vas; municipality: Kőszeg; locality: Kőszegi Landscape Park, Hármas Stream; decimalLatitude: 47.39411; decimalLongitude: 16.46251; **Identification:** identifiedBy: L.-P. Kolcsár; **Event:** eventDate: 2002-06-10; verbatimEventDate: 10/Jul/2002; **Record Level:** institutionCode: HNHM; basisOfRecord: PreservedSpecimen
- **Type status:** Other material. **Occurrence:** occurrenceRemarks: 1 male; recordedBy: V.E. Pilipenko; individualCount: 1; sex: male; occurrenceID: EU_LIM_532; **Taxon:** scientificName: Lipsothrixerrans (Walker, 1848); family: Limoniidae; genus: Lipsothrix; specificEpithet: errans; scientificNameAuthorship: (Walker, 1848); **Location:** country: Russia; stateProvince: Central European Russia; county: Moskovskaya Oblast; municipality: Solnechnogorsk district; locality: Chashnikovo; verbatimElevation: 220 m; minimumElevationInMeters: 220; decimalLatitude: 56.0375; decimalLongitude: 37.1874; **Identification:** identifiedBy: V.E. Pilipenko; **Event:** samplingProtocol: Sweep net; eventDate: 1992-06-03; verbatimEventDate: 03/Jun/1992; **Record Level:** institutionCode: VPMC; basisOfRecord: PreservedSpecimen
- **Type status:** Other material. **Occurrence:** occurrenceRemarks: 1 male; recordedBy: V.E. Pilipenko; individualCount: 1; sex: male; occurrenceID: EU_LIM_533; **Taxon:** scientificName: Lipsothrixerrans (Walker, 1848); family: Limoniidae; genus: Lipsothrix; specificEpithet: errans; scientificNameAuthorship: (Walker, 1848); **Location:** country: Russia; stateProvince: Central European Russia; county: Moskovskaya Oblast; municipality: Solnechnogorsk district; locality: Chashnikovo; verbatimElevation: 220 m; minimumElevationInMeters: 220; decimalLatitude: 56.0375; decimalLongitude: 37.1874; **Identification:** identifiedBy: V.E. Pilipenko; **Event:** samplingProtocol: Sweep net; eventDate: 1994-06-25; verbatimEventDate: 25/Jun/1994; **Record Level:** institutionCode: VPMC; basisOfRecord: PreservedSpecimen
- **Type status:** Other material. **Occurrence:** occurrenceRemarks: 1 male; recordedBy: V.E. Pilipenko; individualCount: 1; sex: male; occurrenceID: EU_LIM_534; **Taxon:** scientificName: Lipsothrixerrans (Walker, 1848); family: Limoniidae; genus: Lipsothrix; specificEpithet: errans; scientificNameAuthorship: (Walker, 1848); **Location:** country: Russia; stateProvince: Central European Russia; county: Moskovskaya Oblast; municipality: Solnechnogorsk district; locality: Chashnikovo; verbatimElevation: 220 m; minimumElevationInMeters: 220; decimalLatitude: 56.0375; decimalLongitude: 37.1874; **Identification:** identifiedBy: V.E. Pilipenko; **Event:** samplingProtocol: Sweep net; eventDate: 1996-06-01; verbatimEventDate: 01/Jun/1996; **Record Level:** institutionCode: VPMC; basisOfRecord: PreservedSpecimen

#### Distribution

First records from Hungary and Russia: RUC.

### Lipsothrix nervosa

Edwards, 1938

D3B0C0D2-1B41-5259-861A-58BDB10C3E8A

https://ccw.naturalis.nl/detail.php?id=10772

#### Materials

- **Type status:** Other material. **Occurrence:** occurrenceRemarks: 1 male; recordedBy: K. Peeters; individualCount: 1; sex: male; occurrenceID: EU_LIM_535; **Taxon:** scientificName: Lipsothrixnervosa Edwards, 1938; family: Limoniidae; genus: Lipsothrix; specificEpithet: nervosa; scientificNameAuthorship: Edwards, 1938; **Location:** country: Belgium; stateProvince: Antwerpen; municipality: Burcht; decimalLatitude: 51.206; decimalLongitude: 4.35; **Identification:** identifiedBy: P. Oosterbroek; **Event:** eventDate: 2015-06-06/2015-06-07; verbatimEventDate: 06-07/Jun/2015; **Record Level:** basisOfRecord: HumanObservation
- **Type status:** Other material. **Occurrence:** occurrenceRemarks: 1 male; recordedBy: L. Papp; individualCount: 1; sex: male; preparations: Pinned; occurrenceID: EU_LIM_536; **Taxon:** scientificName: Lipsothrixnervosa Edwards, 1938; family: Limoniidae; genus: Lipsothrix; specificEpithet: nervosa; scientificNameAuthorship: Edwards, 1938; **Location:** country: Hungary; stateProvince: Baranya; municipality: Óbánya; locality: Keleti-Mecsek Landscape Park, Óbánya Valley; decimalLatitude: 46.21596; decimalLongitude: 18.39398; **Identification:** identifiedBy: L.-P. Kolcsár; **Event:** eventDate: 1999-05-28; verbatimEventDate: 28/May/1999; **Record Level:** institutionCode: HNHM; basisOfRecord: PreservedSpecimen
- **Type status:** Other material. **Occurrence:** occurrenceRemarks: 1 males; recordedBy: J. Roháček; individualCount: 1; sex: male; preparations: Pinned; occurrenceID: EU_LIM_537; **Taxon:** scientificName: Lipsothrixnervosa Edwards, 1938; family: Limoniidae; genus: Lipsothrix; specificEpithet: nervosa; scientificNameAuthorship: Edwards, 1938; **Location:** country: Italy; stateProvince: Calabria; municipality: Mongiana; locality: 2.5 km NNE, Serre Calabresi Mts.; verbatimElevation: 1035 m; minimumElevationInMeters: 1035; decimalLatitude: 38.53583; decimalLongitude: 16.32583; **Identification:** identifiedBy: J. Starý; **Event:** eventDate: 2018-05-22; verbatimEventDate: 22/May/2018; habitat: brook; **Record Level:** institutionCode: PCJS; basisOfRecord: PreservedSpecimen

#### Distribution

First records from Hungary and Italy (from mainland). The species is reported from Belgium in CCW by K. Peeters (in litt. 2015, 2016); we here publish the collection data for those records.

### Lipsothrix nobilis

Loew, 1873

E58F8E0D-9A17-5379-83E8-37882C102413

https://ccw.naturalis.nl/detail.php?id=10775

#### Materials

- **Type status:** Other material. **Occurrence:** occurrenceRemarks: 1; recordedBy: D.I. Gavryushin; individualCount: male; preparations: Pinned; occurrenceID: EU_LIM_538; **Taxon:** scientificName: Lipsothrixnobilis Loew, 1873; family: Limoniidae; genus: Lipsothrix; specificEpithet: nobilis; scientificNameAuthorship: Loew, 1873; **Location:** country: Serbia; stateProvince: Zaječar; municipality: Knjaževac; locality: Crni Vrh; verbatimElevation: 800 m; minimumElevationInMeters: 800; decimalLatitude: 43.407; decimalLongitude: 22.587; **Identification:** identifiedBy: D.I. Gavryushin; **Event:** samplingProtocol: Sweep net; eventDate: 2015-06-01/2015-07-07; verbatimEventDate: 01-07/Jul/2015; **Record Level:** institutionCode: ZMMU; basisOfRecord: PreservedSpecimen

#### Distribution

First record from Serbia.

### Lipsothrix remota

(Walker, 1848)

A2EC3225-266D-523A-9148-8F195FDE49E4

https://ccw.naturalis.nl/detail.php?id=10779

#### Materials

- **Type status:** Other material. **Occurrence:** occurrenceRemarks: 2 females; recordedBy: M. Pollet; individualCount: 2; sex: female; preparations: Ethanol; occurrenceID: EU_LIM_539; **Taxon:** scientificName: Lipsothrixremota (Walker, 1848); family: Limoniidae; genus: Lipsothrix; specificEpithet: remota; scientificNameAuthorship: (Walker, 1848); **Location:** island: Corsica; country: France; county: Corse-du-Sud; municipality: Serra di Scopamène et Sorbollano; locality: Campu di Bonza; verbatimElevation: 920 m; minimumElevationInMeters: 920; decimalLatitude: 41.46215; decimalLongitude: 9.07158; **Identification:** identifiedBy: P. Boardman; **Event:** samplingProtocol: Pan trap; eventDate: 2019-06-27; verbatimEventDate: 27/Jun/2019; **Record Level:** institutionCode: MNHN; basisOfRecord: PreservedSpecimen

#### Distribution

Species previously reported from mainland France; here, we publish the first record from Corsica.

### Metalimnobia (Metalimnobia) bifasciata

(Schrank, 1781)

C44E451F-AF50-5BF9-8DC3-C67C022C1DDC

https://ccw.naturalis.nl/detail.php?id=10799

#### Materials

- **Type status:** Other material. **Occurrence:** occurrenceRemarks: 3 male + 2 female; recordedBy: R. Buesink | JNM; individualCount: 5; sex: male, female; preparations: Ethanol; occurrenceID: EU_LIM_856; **Taxon:** scientificName: Metalimnobia (Metalimnobia) bifasciata (Schrank, 1781); family: Limoniidae; genus: Metalimnobia; subgenus: Metalimnobia; specificEpithet: bifasciata; scientificNameAuthorship: (Schrank, 1781); **Location:** country: Albania; stateProvince: Kukës; municipality: Dragobi; locality: Valley just southwest of Dragobi; verbatimElevation: 690 m; minimumElevationInMeters: 690; decimalLatitude: 42.4222; decimalLongitude: 19.9639; **Identification:** identifiedBy: M.C. d'Oliveira; **Event:** samplingProtocol: Flight Interception Trap; eventDate: 2019-8-7/2019-11-7; verbatimEventDate: 08/July-11/July/2019; habitat: Beech forest, next to stream; **Record Level:** institutionCode: PCMCO; basisOfRecord: PreservedSpecimen
- **Type status:** Other material. **Occurrence:** occurrenceRemarks: 2 females; recordedBy: D.I. Gavryushin; individualCount: 2; sex: female; preparations: Pinned; occurrenceID: EU_LIM_540; **Taxon:** scientificName: Metalimnobia (Metalimnobia) bifasciata (Schrank, 1781); family: Limoniidae; genus: Metalimnobia; subgenus: Metalimnobia; specificEpithet: bifasciata; scientificNameAuthorship: (Schrank, 1781); **Location:** country: Belarus; stateProvince: Minsk; county: Barysaw; locality: Barysaw; verbatimElevation: 155 m; minimumElevationInMeters: 155; decimalLatitude: 54.25542; decimalLongitude: 28.48092; **Identification:** identifiedBy: D.I. Gavryushin; **Event:** samplingProtocol: Sweep net; eventDate: 2013-06-05; verbatimEventDate: 5/Jul/2013; **Record Level:** institutionCode: ZMMU; basisOfRecord: PreservedSpecimen
- **Type status:** Other material. **Occurrence:** occurrenceRemarks: 1 male; recordedBy: L.-P. Kolcsár; individualCount: 1; sex: male; preparations: Ethanol; occurrenceID: EU_LIM_541; **Taxon:** scientificName: Metalimnobia (Metalimnobia) bifasciata (Schrank, 1781); family: Limoniidae; genus: Metalimnobia; subgenus: Metalimnobia; specificEpithet: bifasciata; scientificNameAuthorship: (Schrank, 1781); **Location:** country: Latvia; municipality: Sigulda; locality: Gauja River; verbatimElevation: 13 m; minimumElevationInMeters: 13; decimalLatitude: 57.1505; decimalLongitude: 24.8168; **Identification:** identifiedBy: L.-P. Kolcsár; **Event:** samplingProtocol: Sweep net; eventDate: 2018-06-24; verbatimEventDate: 24/Jul/2018; **Record Level:** institutionCode: CKLP; basisOfRecord: PreservedSpecimen
- **Type status:** Other material. **Occurrence:** occurrenceRemarks: 1 male; recordedBy: E. Eiroa; individualCount: 1; sex: male; preparations: Pinned; occurrenceID: EU_LIM_542; **Taxon:** scientificName: Metalimnobia (Metalimnobia) bifasciata (Schrank, 1781); family: Limoniidae; genus: Metalimnobia; subgenus: Metalimnobia; specificEpithet: bifasciata; scientificNameAuthorship: (Schrank, 1781); **Location:** country: Spain; stateProvince: Galicia, Lugo; municipality: Cervantes; locality: San Martín, sierra de Os Ancares; verbatimElevation: 386 m; minimumElevationInMeters: 386; decimalLatitude: 42.90284; decimalLongitude: -7.0647; **Identification:** identifiedBy: E. Eiroa; **Event:** samplingProtocol: Sweep net; eventDate: 1984-09-27; verbatimEventDate: 27/Sept/1984; **Record Level:** institutionCode: USC; basisOfRecord: PreservedSpecimen

#### Distribution

First records from Albania, Belarus and Spain (from mainland). We confirm the presence of the species in Latvia.

### Metalimnobia (Metalimnobia) quadrimaculata

(Linnaeus, 1760)

56625D72-BDEC-5DEE-8B0D-CE125D78F485

https://ccw.naturalis.nl/detail.php?id=10821

#### Materials

- **Type status:** Other material. **Occurrence:** occurrenceRemarks: 7 male + 8 female; recordedBy: R. Buesink | JNM; individualCount: 15; sex: male, female; preparations: Ethanol; occurrenceID: EU_LIM_857; **Taxon:** scientificName: Metalimnobia (Metalimnobia) quadrimaculata (Linnaeus, 1760); family: Limoniidae; genus: Metalimnobia; subgenus: Metalimnobia; specificEpithet: quadrimaculata; scientificNameAuthorship: (Linnaeus, 1760); **Location:** country: Albania; stateProvince: Kukës; municipality: Dragobi; locality: Valley just southwest of Dragobi; verbatimElevation: 690 m; minimumElevationInMeters: 690; decimalLatitude: 42.4222; decimalLongitude: 19.9639; **Identification:** identifiedBy: M.C. d'Oliveira; **Event:** samplingProtocol: Flight Interception Trap; eventDate: 2019-8-7/2019-11-7; verbatimEventDate: 08/July-11/July/2019; habitat: Beech forest, next to stream; **Record Level:** institutionCode: Private; basisOfRecord: PreservedSpecimen
- **Type status:** Other material. **Occurrence:** occurrenceRemarks: 1 male; recordedBy: D.I. Gavryushin; individualCount: 1; sex: male; occurrenceID: EU_LIM_543; **Taxon:** scientificName: Metalimnobia (Metalimnobia) quadrimaculata (Linnaeus, 1760); family: Limoniidae; genus: Metalimnobia; subgenus: Metalimnobia; specificEpithet: quadrimaculata; scientificNameAuthorship: (Linnaeus, 1760); **Location:** country: Russia; stateProvince: East European Russia; county: Bashkortostan Respublika; municipality: Beloretsk district; locality: Abzakovo env., Karan River; verbatimElevation: 533 m; minimumElevationInMeters: 533; decimalLatitude: 53.83717; decimalLongitude: 58.57878; **Identification:** identifiedBy: D.I. Gavryushin; **Event:** samplingProtocol: Sweep net; eventDate: 06/19/2015; verbatimEventDate: Jul-19-2015; **Record Level:** institutionCode: ZMMU; basisOfRecord: PreservedSpecimen
- **Type status:** Other material. **Occurrence:** occurrenceRemarks: 1 male; recordedBy: N.G. Petrov; individualCount: 1; sex: male; occurrenceID: EU_LIM_544; **Taxon:** scientificName: Metalimnobia (Metalimnobia) quadrimaculata (Linnaeus, 1760); family: Limoniidae; genus: Metalimnobia; subgenus: Metalimnobia; specificEpithet: quadrimaculata; scientificNameAuthorship: (Linnaeus, 1760); **Location:** country: Russia; stateProvince: East European Russia; county: Tatarstan Respublika; municipality: Kazan; locality: district Derbyshki; verbatimElevation: 100 m; minimumElevationInMeters: 100; decimalLatitude: 55.85616; decimalLongitude: 49.22023; **Identification:** identifiedBy: N.M. Paramonov; **Event:** samplingProtocol: Light trap; eventDate: 2010-04-26; verbatimEventDate: 26/Apr/2010; habitat: funnel karst origin; **Record Level:** institutionCode: ZIN; basisOfRecord: PreservedSpecimen
- **Type status:** Other material. **Occurrence:** occurrenceRemarks: 1 female; recordedBy: N.M. Paramonov; individualCount: 1; sex: female; occurrenceID: EU_LIM_545; **Taxon:** scientificName: Metalimnobia (Metalimnobia) quadrimaculata (Linnaeus, 1760); family: Limoniidae; genus: Metalimnobia; subgenus: Metalimnobia; specificEpithet: quadrimaculata; scientificNameAuthorship: (Linnaeus, 1760); **Location:** country: Russia; stateProvince: East European Russia; county: Tatarstan Respublika; municipality: Kazan; locality: district Derbyshki; verbatimElevation: 100 m; minimumElevationInMeters: 100; decimalLatitude: 55.85616; decimalLongitude: 49.22023; **Identification:** identifiedBy: N.M. Paramonov; **Event:** samplingProtocol: Sweep net; eventDate: 2010-06-15; verbatimEventDate: 15/Jun/2010; habitat: funnel karst origin; **Record Level:** institutionCode: ZIN; basisOfRecord: PreservedSpecimen
- **Type status:** Other material. **Occurrence:** occurrenceRemarks: 1 male; recordedBy: N.M. Paramonov; individualCount: 1; sex: male; occurrenceID: EU_LIM_546; **Taxon:** scientificName: Metalimnobia (Metalimnobia) quadrimaculata (Linnaeus, 1760); family: Limoniidae; genus: Metalimnobia; subgenus: Metalimnobia; specificEpithet: quadrimaculata; scientificNameAuthorship: (Linnaeus, 1760); **Location:** country: Russia; stateProvince: East European Russia; county: Tatarstan Respublika; municipality: Zelenodol’sk district; locality: Volga-Kama State Nature Biosphere Reserve, «Raifa», biostation Raifa; verbatimElevation: 100 m; minimumElevationInMeters: 100; decimalLatitude: 55.88868; decimalLongitude: 48.71434; **Identification:** identifiedBy: N.M. Paramonov; **Event:** samplingProtocol: Sweep net; eventDate: 2009-06-12; verbatimEventDate: 12/Jun/2009; **Record Level:** institutionCode: ZIN; basisOfRecord: PreservedSpecimen
- **Type status:** Other material. **Occurrence:** occurrenceRemarks: 1 male; recordedBy: N.M. Paramonov; individualCount: 1; sex: male; occurrenceID: EU_LIM_547; **Taxon:** scientificName: Metalimnobia (Metalimnobia) quadrimaculata (Linnaeus, 1760); family: Limoniidae; genus: Metalimnobia; subgenus: Metalimnobia; specificEpithet: quadrimaculata; scientificNameAuthorship: (Linnaeus, 1760); **Location:** country: Russia; stateProvince: East European Russia; county: Tatarstan Respublika; municipality: Zelenodol’sk district; locality: Volga-Kama State Nature Biosphere Reserve, «Raifa», biostation Raifa; verbatimElevation: 100 m; minimumElevationInMeters: 100; decimalLatitude: 55.88868; decimalLongitude: 48.71434; **Identification:** identifiedBy: N.M. Paramonov; **Event:** samplingProtocol: Sweep net; eventDate: 2009-06-13; verbatimEventDate: 13/Jun/2009; **Record Level:** institutionCode: ZIN; basisOfRecord: PreservedSpecimen
- **Type status:** Other material. **Occurrence:** occurrenceRemarks: 1 female; recordedBy: N.M. Paramonov; individualCount: 1; sex: female; occurrenceID: EU_LIM_548; **Taxon:** scientificName: Metalimnobia (Metalimnobia) quadrimaculata (Linnaeus, 1760); family: Limoniidae; genus: Metalimnobia; subgenus: Metalimnobia; specificEpithet: quadrimaculata; scientificNameAuthorship: (Linnaeus, 1760); **Location:** country: Russia; stateProvince: East European Russia; county: Tatarstan Respublika; municipality: Zelenodol’sk district; locality: Volga-Kama State Nature Biosphere Reserve, «Raifa», biostation Raifa; verbatimElevation: 100 m; minimumElevationInMeters: 100; decimalLatitude: 55.88868; decimalLongitude: 48.71434; **Identification:** identifiedBy: N.M. Paramonov; **Event:** samplingProtocol: Sweep net; eventDate: 2012-06-29; verbatimEventDate: 29/Jun/2012; **Record Level:** institutionCode: ZIN; basisOfRecord: PreservedSpecimen
- **Type status:** Other material. **Occurrence:** occurrenceRemarks: 1 male; recordedBy: N.M. Paramonov; individualCount: 1; sex: male; occurrenceID: EU_LIM_549; **Taxon:** scientificName: Metalimnobia (Metalimnobia) quadrimaculata (Linnaeus, 1760); family: Limoniidae; genus: Metalimnobia; subgenus: Metalimnobia; specificEpithet: quadrimaculata; scientificNameAuthorship: (Linnaeus, 1760); **Location:** country: Russia; stateProvince: East European Russia; county: Tatarstan Respublika; municipality: Zelenodol’sk district; locality: Sumka River, Lake Raifskoe; verbatimElevation: 75 m; minimumElevationInMeters: 75; decimalLatitude: 55.91237; decimalLongitude: 48.73159; **Identification:** identifiedBy: N.M. Paramonov; **Event:** samplingProtocol: Sweep net; eventDate: 2009-06-11; verbatimEventDate: 11/Jun/2009; **Record Level:** institutionCode: ZIN; basisOfRecord: PreservedSpecimen

#### Distribution

First records from Albania and Russia: RUE.

### Metalimnobia (Metalimnobia) tenua

Savchenko, 1976

911E4313-3312-503F-A142-FB4DB3B6C97C

https://ccw.naturalis.nl/detail.php?id=10826

#### Materials

- **Type status:** Other material. **Occurrence:** occurrenceRemarks: 1 male; recordedBy: L.-P. Kolcsár; individualCount: 1; sex: male; preparations: Ethanol; occurrenceID: EU_LIM_550; **Taxon:** scientificName: Metalimnobia (Metalimnobia) tenua Savchenko, 1976; family: Limoniidae; genus: Metalimnobia; subgenus: Metalimnobia; specificEpithet: tenua; scientificNameAuthorship: Savchenko, 1976; **Location:** country: Romania; stateProvince: Harghita; municipality: Suseni; locality: Harghita Mts., Senetea brook; verbatimElevation: 1050 m; minimumElevationInMeters: 1050; decimalLatitude: 46.57953; decimalLongitude: 25.57836; **Identification:** identifiedBy: L.-P. Kolcsár; **Event:** samplingProtocol: Sweep net; eventDate: 2012-08-04; verbatimEventDate: 4/Aug/2012; **Record Level:** institutionCode: CKLP; basisOfRecord: PreservedSpecimen
- **Type status:** Other material. **Occurrence:** occurrenceRemarks: 1 male; recordedBy: L.-P. Kolcsár; individualCount: 1; sex: male; preparations: Ethanol; occurrenceID: EU_LIM_551; **Taxon:** scientificName: Metalimnobia (Metalimnobia) tenua Savchenko, 1976; family: Limoniidae; genus: Metalimnobia; subgenus: Metalimnobia; specificEpithet: tenua; scientificNameAuthorship: Savchenko, 1976; **Location:** country: Romania; stateProvince: Harghita; municipality: Bălan; locality: Hăşmaş Mts., Gálkút brook; verbatimElevation: 884 m; minimumElevationInMeters: 884; decimalLatitude: 46.64734; decimalLongitude: 25.82497; **Identification:** identifiedBy: L.-P. Kolcsár; **Event:** samplingProtocol: Sweep net; eventDate: 2012-08-03; verbatimEventDate: 3/Aug/2012; **Record Level:** institutionCode: CKLP; basisOfRecord: PreservedSpecimen
- **Type status:** Other material. **Occurrence:** occurrenceRemarks: 1 male; recordedBy: L.-P. Kolcsár; individualCount: 1; sex: male; preparations: Ethanol; occurrenceID: EU_LIM_552; **Taxon:** scientificName: Metalimnobia (Metalimnobia) tenua Savchenko, 1976; family: Limoniidae; genus: Metalimnobia; subgenus: Metalimnobia; specificEpithet: tenua; scientificNameAuthorship: Savchenko, 1976; **Location:** country: Romania; stateProvince: Harghita; municipality: Bălan; locality: Giurgeu Mts., Olt River; verbatimElevation: 1078 m; minimumElevationInMeters: 1078; decimalLatitude: 46.71944; decimalLongitude: 25.73632; **Identification:** identifiedBy: L.-P. Kolcsár; **Event:** samplingProtocol: Sweep net; eventDate: 2012-08-03; verbatimEventDate: 3/Aug/2012; **Record Level:** institutionCode: CKLP; basisOfRecord: PreservedSpecimen
- **Type status:** Other material. **Occurrence:** occurrenceRemarks: 1 male; recordedBy: L.-P. Kolcsár; individualCount: 1; sex: male; preparations: Ethanol; occurrenceID: EU_LIM_553; **Taxon:** scientificName: Metalimnobia (Metalimnobia) tenua Savchenko, 1976; family: Limoniidae; genus: Metalimnobia; subgenus: Metalimnobia; specificEpithet: tenua; scientificNameAuthorship: Savchenko, 1976; **Location:** country: Romania; stateProvince: Harghita; municipality: Gheorgheni; locality: Giurgeu Mts., Magas-Bükk brook; verbatimElevation: 995 m; minimumElevationInMeters: 995; decimalLatitude: 46.72219; decimalLongitude: 25.66844; **Identification:** identifiedBy: L.-P. Kolcsár; **Event:** samplingProtocol: Sweep net; eventDate: 2012-08-03; verbatimEventDate: 3/Aug/2012; **Record Level:** institutionCode: CKLP; basisOfRecord: PreservedSpecimen
- **Type status:** Other material. **Occurrence:** occurrenceRemarks: 2 males; recordedBy: L.-P. Kolcsár; individualCount: 2; sex: male; preparations: Ethanol; occurrenceID: EU_LIM_554; **Taxon:** scientificName: Metalimnobia (Metalimnobia) tenua Savchenko, 1976; family: Limoniidae; genus: Metalimnobia; subgenus: Metalimnobia; specificEpithet: tenua; scientificNameAuthorship: Savchenko, 1976; **Location:** country: Romania; stateProvince: Harghita; municipality: Lacu Roșu; locality: Hăşmaş Mts., Suhardul Mic; verbatimElevation: 1105 m; minimumElevationInMeters: 1105; decimalLatitude: 46.79678; decimalLongitude: 25.79443; **Identification:** identifiedBy: L.-P. Kolcsár; **Event:** samplingProtocol: Sweep net; eventDate: 2010-09-05; verbatimEventDate: 5/Sep/2010; **Record Level:** institutionCode: CKLP; basisOfRecord: PreservedSpecimen
- **Type status:** Other material. **Occurrence:** occurrenceRemarks: 4 males; recordedBy: L.-P. Kolcsár; individualCount: 4; sex: male; preparations: Ethanol; occurrenceID: EU_LIM_555; **Taxon:** scientificName: Metalimnobia (Metalimnobia) tenua Savchenko, 1976; family: Limoniidae; genus: Metalimnobia; subgenus: Metalimnobia; specificEpithet: tenua; scientificNameAuthorship: Savchenko, 1976; **Location:** country: Romania; stateProvince: Harghita; municipality: Hagota; locality: Giurgeu Mts., Tisașul Valley; verbatimElevation: 860 m; minimumElevationInMeters: 860; decimalLatitude: 46.86179; decimalLongitude: 25.67723; **Identification:** identifiedBy: L.-P. Kolcsár; **Event:** samplingProtocol: Sweep net; eventDate: 2010-08-18; verbatimEventDate: 18/Aug/2010; **Record Level:** institutionCode: CKLP; basisOfRecord: PreservedSpecimen
- **Type status:** Other material. **Occurrence:** occurrenceRemarks: 1 male; recordedBy: A. Polevoi; individualCount: 1; sex: male; preparations: Pinned; occurrenceID: EU_LIM_556; **Taxon:** scientificName: Metalimnobia (Metalimnobia) tenua Savchenko, 1976; family: Limoniidae; genus: Metalimnobia; subgenus: Metalimnobia; specificEpithet: tenua; scientificNameAuthorship: Savchenko, 1976; **Location:** country: Russia; stateProvince: North European Russia; county: Arkhangelsk region; municipality: Primorsky district; locality: Chesmenskiy; verbatimElevation: 20 m; minimumElevationInMeters: 20; decimalLatitude: 64.718; decimalLongitude: 36.54676; **Identification:** identifiedBy: A. Polevoi; **Event:** samplingProtocol: Sweep net; eventDate: 2020-06-30; verbatimEventDate: 31/Jul/2020; **Record Level:** institutionCode: FRIP; basisOfRecord: PreservedSpecimen
- **Type status:** Other material. **Occurrence:** occurrenceRemarks: 1 male; recordedBy: A. Polevoi; individualCount: 1; sex: male; preparations: Pinned; occurrenceID: EU_LIM_557; **Taxon:** scientificName: Metalimnobia (Metalimnobia) tenua Savchenko, 1976; family: Limoniidae; genus: Metalimnobia; subgenus: Metalimnobia; specificEpithet: tenua; scientificNameAuthorship: Savchenko, 1976; **Location:** country: Russia; stateProvince: North European Russia; county: Republic Karelia; municipality: Prionezhskiy district; locality: Sheltozero, 3 km NW; verbatimElevation: 50 m; minimumElevationInMeters: 50; decimalLatitude: 61.39267; decimalLongitude: 35.30831; **Identification:** identifiedBy: A. Polevoi; **Event:** samplingProtocol: Sweep net; eventDate: 2004-06-13; verbatimEventDate: 13/Jul/2004; **Record Level:** institutionCode: FRIP; basisOfRecord: PreservedSpecimen
- **Type status:** Other material. **Occurrence:** occurrenceRemarks: 1 male; recordedBy: A. Polevoi; individualCount: 1; sex: male; preparations: Pinned; occurrenceID: EU_LIM_558; **Taxon:** scientificName: Metalimnobia (Metalimnobia) tenua Savchenko, 1976; family: Limoniidae; genus: Metalimnobia; subgenus: Metalimnobia; specificEpithet: tenua; scientificNameAuthorship: Savchenko, 1976; **Location:** island: Karel'skiy; country: Russia; stateProvince: North European Russia; county: Republic Karelia; municipality: Medvezhegorsk district; locality: Karel'skiy Island; verbatimElevation: 25 m; minimumElevationInMeters: 25; decimalLatitude: 62.01373; decimalLongitude: 35.20952; **Identification:** identifiedBy: A. Polevoi; **Event:** samplingProtocol: Sweep net; eventDate: 2017-06-03; verbatimEventDate: 03/Jul/2017; **Record Level:** institutionCode: FRIP; basisOfRecord: PreservedSpecimen
- **Type status:** Other material. **Occurrence:** occurrenceRemarks: 1 male; recordedBy: A. Polevoi; individualCount: 1; sex: male; preparations: Pinned; occurrenceID: EU_LIM_559; **Taxon:** scientificName: Metalimnobia (Metalimnobia) tenua Savchenko, 1976; family: Limoniidae; genus: Metalimnobia; subgenus: Metalimnobia; specificEpithet: tenua; scientificNameAuthorship: Savchenko, 1976; **Location:** country: Russia; stateProvince: North European Russia; county: Republic Karelia; municipality: Kondopoga district; locality: Tereki, 5 km SE; verbatimElevation: 130 m; minimumElevationInMeters: 130; decimalLatitude: 62.21332; decimalLongitude: 33.87466; **Identification:** identifiedBy: A. Polevoi; **Event:** samplingProtocol: Sweep net; eventDate: 2017-08-16; verbatimEventDate: 16/Aug/2017; **Record Level:** institutionCode: FRIP; basisOfRecord: PreservedSpecimen
- **Type status:** Other material. **Occurrence:** occurrenceRemarks: 1 male; recordedBy: A. Polevoi; individualCount: 1; sex: male; preparations: Pinned; occurrenceID: EU_LIM_560; **Taxon:** scientificName: Metalimnobia (Metalimnobia) tenua Savchenko, 1976; family: Limoniidae; genus: Metalimnobia; subgenus: Metalimnobia; specificEpithet: tenua; scientificNameAuthorship: Savchenko, 1976; **Location:** country: Russia; stateProvince: North European Russia; county: Republic Karelia; municipality: Kondopoga district; locality: Vendery, 1.5 km N; verbatimElevation: 170 m; minimumElevationInMeters: 170; decimalLatitude: 62.24094; decimalLongitude: 33.2971; **Identification:** identifiedBy: A. Polevoi; **Event:** samplingProtocol: Malaise trap; eventDate: 2017-08-29/2017-08-31; verbatimEventDate: 29-31/Aug/2017; **Record Level:** institutionCode: FRIP; basisOfRecord: PreservedSpecimen
- **Type status:** Other material. **Occurrence:** occurrenceRemarks: 1 male; recordedBy: A. Polevoi; individualCount: 1; sex: male; preparations: Pinned; occurrenceID: EU_LIM_561; **Taxon:** scientificName: Metalimnobia (Metalimnobia) tenua Savchenko, 1976; family: Limoniidae; genus: Metalimnobia; subgenus: Metalimnobia; specificEpithet: tenua; scientificNameAuthorship: Savchenko, 1976; **Location:** country: Russia; stateProvince: North European Russia; county: Republic Karelia; municipality: Kondopoga district; locality: Rapsudosero Lake; verbatimElevation: 170 m; minimumElevationInMeters: 170; decimalLatitude: 62.24926; decimalLongitude: 33.29573; **Identification:** identifiedBy: A. Polevoi; **Event:** samplingProtocol: Light trap; eventDate: 2017-08-30; verbatimEventDate: 30/Aug/2017; **Record Level:** institutionCode: FRIP; basisOfRecord: PreservedSpecimen

#### Distribution

First records from Romania and Russia: RUN.

### Metalimnobia (Metalimnobia) zetterstedti

(Tjeder, 1968)

736BE582-C781-51DC-B315-B8F5237C6E3A

https://ccw.naturalis.nl/detail.php?id=10836

#### Materials

- **Type status:** Other material. **Occurrence:** occurrenceRemarks: 1 female; recordedBy: D.I. Gavryushin; individualCount: 1; sex: female; preparations: Pinned; occurrenceID: EU_LIM_562; **Taxon:** scientificName: Metalimnobia (Metalimnobia) zetterstedti (Tjeder, 1968); family: Limoniidae; genus: Metalimnobia; subgenus: Metalimnobia; specificEpithet: zetterstedti; scientificNameAuthorship: (Tjeder, 1968); **Location:** country: Belarus; stateProvince: Minsk; county: Barysaw; locality: Glivin; verbatimElevation: 161 m; minimumElevationInMeters: 161; decimalLatitude: 54.14902; decimalLongitude: 28.63648; **Identification:** identifiedBy: D.I. Gavryushin; **Event:** samplingProtocol: Sweep net; eventDate: 2013-06-06; verbatimEventDate: 6/Jul/2013; **Record Level:** institutionCode: ZMMU; basisOfRecord: PreservedSpecimen

#### Distribution

First record from Belarus.

### Molophilus (Molophilus) anthracinus

Lackschewitz, 1940

9A648CD4-C569-5F93-A793-9518A421CC25

https://ccw.naturalis.nl/detail.php?id=2532

#### Materials

- **Type status:** Other material. **Occurrence:** occurrenceRemarks: 4 males; recordedBy: L.-P. Kolcsár; individualCount: 4; sex: male; preparations: Ethanol; occurrenceID: EU_LIM_563; **Taxon:** scientificName: Molophilus (Molophilus) anthracinus Lackschewitz, 1940; family: Limoniidae; genus: Molophilus; subgenus: Molophilus; specificEpithet: anthracinus; scientificNameAuthorship: Lackschewitz, 1940; **Location:** country: Romania; stateProvince: Argeș; municipality: Slatina; locality: Făgăraș Mts., Rea Valley; verbatimElevation: 1550 m; minimumElevationInMeters: 1550; decimalLatitude: 45.5918; decimalLongitude: 24.7638; **Identification:** identifiedBy: L.-P. Kolcsár; **Event:** samplingProtocol: Sweep net; eventDate: 2017-08-19; verbatimEventDate: 19/Aug/2017; **Record Level:** institutionCode: CKLP; basisOfRecord: PreservedSpecimen

#### Distribution

First record from Romania.

### Molophilus (Molophilus) appendiculatus

(Staeger, 1840)

73B8A16F-5E28-57BB-BE18-5F821629A45C

https://ccw.naturalis.nl/detail.php?id=2538&

#### Materials

- **Type status:** Other material. **Occurrence:** occurrenceRemarks: 1 male; recordedBy: D.I. Gavryushin; individualCount: 1; sex: male; occurrenceID: EU_LIM_564; **Taxon:** scientificName: Molophilus (Molophilus) appendiculatus (Staeger, 1840); family: Limoniidae; genus: Molophilus; subgenus: Molophilus; specificEpithet: appendiculatus; scientificNameAuthorship: (Staeger, 1840); **Location:** country: Russia; stateProvince: East European Russia; county: Bashkortostan Respublika; municipality: Uchaly district; locality: Ural-Tau St. env.; verbatimElevation: 777 m; minimumElevationInMeters: 777; decimalLatitude: 53.96805; decimalLongitude: 58.57613; **Identification:** identifiedBy: D.I. Gavryushin; **Event:** samplingProtocol: Sweep net; eventDate: 2015-06-09; verbatimEventDate: 09/Jul/2015; **Record Level:** institutionCode: ZMMU; basisOfRecord: PreservedSpecimen
- **Type status:** Other material. **Occurrence:** occurrenceRemarks: 2 males; recordedBy: D.I. Gavryushin; individualCount: 2; sex: male; occurrenceID: EU_LIM_565; **Taxon:** scientificName: Molophilus (Molophilus) appendiculatus (Staeger, 1840); family: Limoniidae; genus: Molophilus; subgenus: Molophilus; specificEpithet: appendiculatus; scientificNameAuthorship: (Staeger, 1840); **Location:** country: Russia; stateProvince: East European Russia; county: Bashkortostan Respublika; municipality: Beloretsk district; locality: Nura River (ca. 4km W of Otnurok village), at the foot of Zolotyie Shishki (Golden Cones) Mts.; verbatimElevation: 607 m; minimumElevationInMeters: 607; decimalLatitude: 54.05155; decimalLongitude: 58.26887; **Identification:** identifiedBy: D.I. Gavryushin; **Event:** samplingProtocol: Sweep net; eventDate: 2015-06-10; verbatimEventDate: 10/Jul/2015; **Record Level:** institutionCode: ZMMU; basisOfRecord: PreservedSpecimen
- **Type status:** Other material. **Occurrence:** occurrenceRemarks: 8 males; recordedBy: D.I. Gavryushin; individualCount: 8; sex: male; occurrenceID: EU_LIM_566; **Taxon:** scientificName: Molophilus (Molophilus) appendiculatus (Staeger, 1840); family: Limoniidae; genus: Molophilus; subgenus: Molophilus; specificEpithet: appendiculatus; scientificNameAuthorship: (Staeger, 1840); **Location:** country: Russia; stateProvince: East European Russia; county: Bashkortostan Respublika; municipality: Beloretsk district; locality: Nura River (ca. 4km W of Otnurok village), at the foot of Zolotyie Shishki (Golden Cones) Mts.; verbatimElevation: 607 m; minimumElevationInMeters: 607; decimalLatitude: 54.05155; decimalLongitude: 58.26887; **Identification:** identifiedBy: D.I. Gavryushin; **Event:** samplingProtocol: Sweep net; eventDate: 2015-06-13; verbatimEventDate: 13/Jul/2015; **Record Level:** institutionCode: ZMMU; basisOfRecord: PreservedSpecimen
- **Type status:** Other material. **Occurrence:** occurrenceRemarks: 1 male; recordedBy: D.I. Gavryushin; individualCount: 1; sex: male; occurrenceID: EU_LIM_567; **Taxon:** scientificName: Molophilus (Molophilus) appendiculatus (Staeger, 1840); family: Limoniidae; genus: Molophilus; subgenus: Molophilus; specificEpithet: appendiculatus; scientificNameAuthorship: (Staeger, 1840); **Location:** country: Russia; stateProvince: East European Russia; county: Bashkortostan Respublika; municipality: Beloretsk district; locality: Nura River (ca. 4km W of Otnurok village), at the foot of Zolotyie Shishki (Golden Cones) Mts.; verbatimElevation: 607 m; minimumElevationInMeters: 607; decimalLatitude: 54.05155; decimalLongitude: 58.26887; **Identification:** identifiedBy: D.I. Gavryushin; **Event:** samplingProtocol: Sweep net; eventDate: 2015-06-16; verbatimEventDate: 16/Jul/2015; **Record Level:** institutionCode: ZMMU; basisOfRecord: PreservedSpecimen
- **Type status:** Other material. **Occurrence:** occurrenceRemarks: 2 males; recordedBy: D.I. Gavryushin; individualCount: 2; sex: male; occurrenceID: EU_LIM_568; **Taxon:** scientificName: Molophilus (Molophilus) appendiculatus (Staeger, 1840); family: Limoniidae; genus: Molophilus; subgenus: Molophilus; specificEpithet: appendiculatus; scientificNameAuthorship: (Staeger, 1840); **Location:** country: Russia; stateProvince: East European Russia; county: Bashkortostan Respublika; municipality: Beloretsk district; locality: Nura River (ca. 4km W of Otnurok village), at the foot of Zolotyie Shishki (Golden Cones) Mts.; verbatimElevation: 607 m; minimumElevationInMeters: 607; decimalLatitude: 54.05155; decimalLongitude: 58.26887; **Identification:** identifiedBy: D.I. Gavryushin; **Event:** samplingProtocol: Sweep net; eventDate: 2012-08-08; verbatimEventDate: 08/Aug/2012; **Record Level:** institutionCode: ZMMU; basisOfRecord: PreservedSpecimen
- **Type status:** Other material. **Occurrence:** occurrenceRemarks: 1 male; recordedBy: D.I. Gavryushin; individualCount: 1; sex: male; occurrenceID: EU_LIM_569; **Taxon:** scientificName: Molophilus (Molophilus) appendiculatus (Staeger, 1840); family: Limoniidae; genus: Molophilus; subgenus: Molophilus; specificEpithet: appendiculatus; scientificNameAuthorship: (Staeger, 1840); **Location:** country: Russia; stateProvince: East European Russia; county: Bashkortostan Respublika; municipality: Beloretsk district; locality: Nura River (ca. 4km W of Otnurok village), at the foot of Zolotyie Shishki (Golden Cones) Mts.; verbatimElevation: 607 m; minimumElevationInMeters: 607; decimalLatitude: 54.05155; decimalLongitude: 58.26887; **Identification:** identifiedBy: D.I. Gavryushin; **Event:** samplingProtocol: Sweep net; eventDate: 2012-08-11; verbatimEventDate: 11/Aug/2012; **Record Level:** institutionCode: ZMMU; basisOfRecord: PreservedSpecimen
- **Type status:** Other material. **Occurrence:** occurrenceRemarks: 6 males, 3 females; recordedBy: N.M. Paramonov; individualCount: 9; sex: male, female; occurrenceID: EU_LIM_570; **Taxon:** scientificName: Molophilus (Molophilus) appendiculatus (Staeger, 1840); family: Limoniidae; genus: Molophilus; subgenus: Molophilus; specificEpithet: appendiculatus; scientificNameAuthorship: (Staeger, 1840); **Location:** country: Russia; stateProvince: East European Russia; county: Tatarstan Respublika; municipality: Verhneuslonsk district; locality: base “Zoostation”, 3,5 km NW Pustye Morkvashi env.; verbatimElevation: 80 m; minimumElevationInMeters: 80; decimalLatitude: 55.47005; decimalLongitude: 48.44092; **Identification:** identifiedBy: N.M. Paramonov; **Event:** samplingProtocol: Sweep net; eventDate: 2013-08-22/2013-08-26; verbatimEventDate: 22-26/Aug/2013; habitat: ravine, wetland; **Record Level:** institutionCode: ZIN; basisOfRecord: PreservedSpecimen
- **Type status:** Other material. **Occurrence:** occurrenceRemarks: 1 male; recordedBy: N.M. Paramonov; individualCount: 1; sex: male; occurrenceID: EU_LIM_571; **Taxon:** scientificName: Molophilus (Molophilus) appendiculatus (Staeger, 1840); family: Limoniidae; genus: Molophilus; subgenus: Molophilus; specificEpithet: appendiculatus; scientificNameAuthorship: (Staeger, 1840); **Location:** country: Russia; stateProvince: East European Russia; county: Tatarstan Respublika; municipality: Zelenodol’sk district; locality: Volga-Kama State Nature Biosphere Reserve, «Raifa», Lake Lenevo; verbatimElevation: 80 m; minimumElevationInMeters: 80; decimalLatitude: 55.90433; decimalLongitude: 48.79115; **Identification:** identifiedBy: N.M. Paramonov; **Event:** samplingProtocol: Sweep net; eventDate: 2012-06-26; verbatimEventDate: 26/Jun/2012; **Record Level:** institutionCode: ZIN; basisOfRecord: PreservedSpecimen

#### Distribution

First records from Russia: RUE.

### Molophilus (Molophilus) ater

(Meigen, 1804)

9366F87A-2511-52CE-AC7B-4892EF742D16

https://ccw.naturalis.nl/detail.php?id=2559

#### Materials

- **Type status:** Other material. **Occurrence:** occurrenceRemarks: 4 males, 1 female; recordedBy: D.I. Gavryushin; individualCount: 5; sex: male, female; preparations: Ethanol; occurrenceID: EU_LIM_572; **Taxon:** scientificName: Molophilus (Molophilus) ater (Meigen, 1804); family: Limoniidae; genus: Molophilus; subgenus: Molophilus; specificEpithet: ater; scientificNameAuthorship: (Meigen, 1804); **Location:** country: Belarus; stateProvince: Vitebsk; county: Haradok; locality: Ezerische; decimalLatitude: 55.83; decimalLongitude: 30; **Identification:** identifiedBy: D.I. Gavryushin; **Event:** samplingProtocol: Sweep net; eventDate: 2019-05-16/2019-05-17; verbatimEventDate: 16-17/May/2019; **Record Level:** institutionCode: ZMMU; basisOfRecord: PreservedSpecimen
- **Type status:** Other material. **Occurrence:** occurrenceRemarks: 1 male; recordedBy: D.I. Gavryushin; individualCount: 1; sex: male; preparations: Pinned; occurrenceID: EU_LIM_573; **Taxon:** scientificName: Molophilus (Molophilus) ater (Meigen, 1804); family: Limoniidae; genus: Molophilus; subgenus: Molophilus; specificEpithet: ater; scientificNameAuthorship: (Meigen, 1804); **Location:** country: Belarus; stateProvince: Vitebsk; county: Haradok; locality: Ezerische; decimalLatitude: 55.83; decimalLongitude: 30; **Identification:** identifiedBy: D.I. Gavryushin; **Event:** samplingProtocol: Sweep net; eventDate: 2019-05-16/2019-05-17; verbatimEventDate: 16-17/May/2019; **Record Level:** institutionCode: ZMMU; basisOfRecord: PreservedSpecimen

#### Distribution

First records from Belarus.

### Molophilus (Molophilus) bifidus

Goetghebuer, 1920

B6CF9E64-7858-536F-813D-D5CA6B9C5D99

https://ccw.naturalis.nl/detail.php?id=2594

#### Materials

- **Type status:** Other material. **Occurrence:** occurrenceRemarks: 1 male; recordedBy: D.I. Gavryushin; individualCount: 1; sex: male; occurrenceID: EU_LIM_574; **Taxon:** scientificName: Molophilus (Molophilus) bifidus Goetghebuer, 1920; family: Limoniidae; genus: Molophilus; subgenus: Molophilus; specificEpithet: bifidus; scientificNameAuthorship: Goetghebuer, 1920; **Location:** country: Russia; stateProvince: East European Russia; county: Bashkortostan Respublika; municipality: Beloretsk district; locality: Nura River (ca. 4km W of Otnurok village), at the foot of Zolotyie Shishki (Golden Cones) Mts.; verbatimElevation: 607 m; minimumElevationInMeters: 607; decimalLatitude: 54.05155; decimalLongitude: 58.26887; **Identification:** identifiedBy: D.I. Gavryushin; **Event:** samplingProtocol: Sweep net; eventDate: 2012-08-08; verbatimEventDate: 08/Aug/2012; **Record Level:** institutionCode: ZMMU; basisOfRecord: PreservedSpecimen
- **Type status:** Other material. **Occurrence:** occurrenceRemarks: 1 male; recordedBy: D.I. Gavryushin; individualCount: 1; sex: male; occurrenceID: EU_LIM_575; **Taxon:** scientificName: Molophilus (Molophilus) bifidus Goetghebuer, 1920; family: Limoniidae; genus: Molophilus; subgenus: Molophilus; specificEpithet: bifidus; scientificNameAuthorship: Goetghebuer, 1920; **Location:** country: Russia; stateProvince: East European Russia; county: Bashkortostan Respublika; municipality: Beloretsk district; locality: Nura River (ca. 4km W of Otnurok village), at the foot of Zolotyie Shishki (Golden Cones) Mts.; verbatimElevation: 607 m; minimumElevationInMeters: 607; decimalLatitude: 54.05155; decimalLongitude: 58.26887; **Identification:** identifiedBy: D.I. Gavryushin; **Event:** samplingProtocol: Sweep net; eventDate: 2012-08-11; verbatimEventDate: 11/Aug/2012; **Record Level:** institutionCode: ZMMU; basisOfRecord: PreservedSpecimen
- **Type status:** Other material. **Occurrence:** occurrenceRemarks: 1 male; recordedBy: V.E. Pilipenko; individualCount: 1; sex: male; occurrenceID: EU_LIM_576; **Taxon:** scientificName: Molophilus (Molophilus) bifidus Goetghebuer, 1920; family: Limoniidae; genus: Molophilus; subgenus: Molophilus; specificEpithet: bifidus; scientificNameAuthorship: Goetghebuer, 1920; **Location:** country: Russia; stateProvince: Central European Russia; county: Moskovskaya Oblast; municipality: Solnechnogorsk district; locality: Chashnikovo; verbatimElevation: 220 m; minimumElevationInMeters: 220; decimalLatitude: 56.0375; decimalLongitude: 37.1874; **Identification:** identifiedBy: V.E. Pilipenko; **Event:** samplingProtocol: Sweep net; eventDate: 1993-08-16; verbatimEventDate: 16/Aug/1993; **Record Level:** institutionCode: VPMC; basisOfRecord: PreservedSpecimen

#### Distribution

First records from Russia: RUC, RUE.

### Molophilus (Molophilus) bihamatus

de Meijere, 1918

03DF8B85-4DAC-5C8B-B5E0-B8382D6C713C

https://ccw.naturalis.nl/detail.php?id=2597

#### Materials

- **Type status:** Other material. **Occurrence:** occurrenceRemarks: 1 male; recordedBy: D.I. Gavryushin; individualCount: 1; sex: male; preparations: Pinned; occurrenceID: EU_LIM_577; **Taxon:** scientificName: Molophilus (Molophilus) bihamatus de Meijere, 1918; family: Limoniidae; genus: Molophilus; subgenus: Molophilus; specificEpithet: bihamatus; scientificNameAuthorship: de Meijere, 1918; **Location:** country: Belarus; stateProvince: Minsk; county: Barysaw; locality: Barysaw; verbatimElevation: 155 m; minimumElevationInMeters: 155; decimalLatitude: 54.25542; decimalLongitude: 28.48092; **Identification:** identifiedBy: D.I. Gavryushin; **Event:** samplingProtocol: Sweep net; eventDate: 2013-06-05; verbatimEventDate: 5/Jul/2013; **Record Level:** institutionCode: ZMMU; basisOfRecord: PreservedSpecimen
- **Type status:** Other material. **Occurrence:** occurrenceRemarks: 1 female; recordedBy: D.I. Gavryushin; individualCount: 1; sex: female; preparations: Pinned; occurrenceID: EU_LIM_578; **Taxon:** scientificName: Molophilus (Molophilus) bihamatus de Meijere, 1918; family: Limoniidae; genus: Molophilus; subgenus: Molophilus; specificEpithet: bihamatus; scientificNameAuthorship: de Meijere, 1918; **Location:** country: Belarus; stateProvince: Minsk; county: Barysaw; locality: Vialikaje Stachava; verbatimElevation: 156 m; minimumElevationInMeters: 156; decimalLatitude: 54.26555; decimalLongitude: 28.38332; **Identification:** identifiedBy: D.I. Gavryushin; **Event:** samplingProtocol: Sweep net; eventDate: 2013-06-07; verbatimEventDate: 7/Jul/2013; **Record Level:** institutionCode: ZMMU; basisOfRecord: PreservedSpecimen
- **Type status:** Other material. **Occurrence:** occurrenceRemarks: 1 male; recordedBy: L. Papp; individualCount: 1; sex: male; preparations: Pinned; occurrenceID: EU_LIM_579; **Taxon:** scientificName: Molophilus (Molophilus) bihamatus de Meijere, 1918; family: Limoniidae; genus: Molophilus; subgenus: Molophilus; specificEpithet: bihamatus; scientificNameAuthorship: de Meijere, 1918; **Location:** country: Hungary; stateProvince: Borsod-Abaúj-Zemplén; municipality: Füzér; locality: Zempléni Landscape Park: László-tanya; decimalLatitude: 48.5708; decimalLongitude: 21.43545; **Identification:** identifiedBy: L.-P. Kolcsár; **Event:** eventDate: 2007-06-26; verbatimEventDate: 26/Jun/2007; habitat: spring and alderbog; **Record Level:** institutionCode: HNHM; basisOfRecord: PreservedSpecimen
- **Type status:** Other material. **Occurrence:** occurrenceRemarks: 2 males, 2 females; recordedBy: L.-P. Kolcsár | E. Török; individualCount: 2; sex: male, female; preparations: Ethanol; occurrenceID: EU_LIM_580; **Taxon:** scientificName: Molophilus (Molophilus) bihamatus de Meijere, 1918; family: Limoniidae; genus: Molophilus; subgenus: Molophilus; specificEpithet: bihamatus; scientificNameAuthorship: de Meijere, 1918; **Location:** country: Romania; stateProvince: Harghita; municipality: Voșlăbeni; locality: Senetea; verbatimElevation: 760 m; minimumElevationInMeters: 760; decimalLatitude: 46.62588; decimalLongitude: 25.59745; **Identification:** identifiedBy: L.-P. Kolcsár; **Event:** samplingProtocol: Sweep net; eventDate: 2017-06-29; verbatimEventDate: 29/Jun/2017; habitat: marshy area; **Record Level:** institutionCode: CKLP; basisOfRecord: PreservedSpecimen

#### Description

Fig. 8

#### Distribution

First records from Belarus, Hungary and Romania.

### Molophilus (Molophilus) bischofi

Lackschewitz, 1940

DE3CB334-802F-5D3E-BBE1-D5356EC7EB44

https://ccw.naturalis.nl/detail.php?id=2604

#### Materials

- **Type status:** Other material. **Occurrence:** occurrenceRemarks: 1 male; recordedBy: C. Quindroit; individualCount: 1; sex: male; preparations: Ethanol; occurrenceID: EU_LIM_581; **Taxon:** scientificName: Molophilus (Molophilus) bischofi Lackschewitz, 1940; family: Limoniidae; genus: Molophilus; subgenus: Molophilus; specificEpithet: bischofi; scientificNameAuthorship: Lackschewitz, 1940; **Location:** country: France; municipality: Villeneuve-de-Rivière; locality: Moulin saint Jean; verbatimElevation: 350m; minimumElevationInMeters: 350; decimalLatitude: 43.10793; decimalLongitude: 0.66069; **Identification:** identifiedBy: C. Quindroit; **Event:** samplingProtocol: Sweep net; eventDate: 2016-04-28; verbatimEventDate: 28/Apr/2016; **Record Level:** institutionCode: PCCQ; basisOfRecord: PreservedSpecimen

#### Distribution

First record from France (from mainland).

### Molophilus (Molophilus) cinereifrons

de Meijere, 1920

1486CFF0-79C1-5E80-9001-085E6AB667E6

https://ccw.naturalis.nl/detail.php?id=2644

#### Materials

- **Type status:** Other material. **Occurrence:** occurrenceRemarks: 1 male; recordedBy: D.I. Gavryushin; individualCount: 1; sex: male; occurrenceID: EU_LIM_582; **Taxon:** scientificName: Molophilus (Molophilus) cinereifrons de Meijere, 1920; family: Limoniidae; genus: Molophilus; subgenus: Molophilus; specificEpithet: cinereifrons; scientificNameAuthorship: de Meijere, 1920; **Location:** country: Russia; stateProvince: East European Russia; county: Bashkortostan Respublika; municipality: Beloretsk district; locality: Abzakovo env., Malyi Kizil River; verbatimElevation: 510 m; minimumElevationInMeters: 510; decimalLatitude: 53.81428; decimalLongitude: 58.5942; **Identification:** identifiedBy: D.I. Gavryushin; **Event:** samplingProtocol: Sweep net; eventDate: 2015-06-12; verbatimEventDate: 12/Jul/2015; **Record Level:** institutionCode: ZMMU; basisOfRecord: PreservedSpecimen
- **Type status:** Other material. **Occurrence:** occurrenceRemarks: 1 male; recordedBy: D.I. Gavryushin; individualCount: 1; sex: male; occurrenceID: EU_LIM_583; **Taxon:** scientificName: Molophilus (Molophilus) cinereifrons de Meijere, 1920; family: Limoniidae; genus: Molophilus; subgenus: Molophilus; specificEpithet: cinereifrons; scientificNameAuthorship: de Meijere, 1920; **Location:** country: Russia; stateProvince: East European Russia; county: Bashkortostan Respublika; municipality: Beloretsk district; locality: Nura River (ca. 4km W of Otnurok village), at the foot of Zolotyie Shishki (Golden Cones) Mts.; verbatimElevation: 607 m; minimumElevationInMeters: 607; decimalLatitude: 54.05155; decimalLongitude: 58.26887; **Identification:** identifiedBy: D.I. Gavryushin; **Event:** samplingProtocol: Sweep net; eventDate: 2015-06-16; verbatimEventDate: 16/Jul/2015; **Record Level:** institutionCode: ZMMU; basisOfRecord: PreservedSpecimen

#### Distribution

First records from Russia: RUE.

### Molophilus (Molophilus) corniger

de Meijere, 1920

40129D28-2092-5647-944F-61FCE641CC73

https://ccw.naturalis.nl/detail.php?id=2660

#### Materials

- **Type status:** Other material. **Occurrence:** occurrenceRemarks: 1 male; recordedBy: D.I. Gavryushin; individualCount: 1; sex: male; occurrenceID: EU_LIM_584; **Taxon:** scientificName: Molophilus (Molophilus) corniger de Meijere, 1920; family: Limoniidae; genus: Molophilus; subgenus: Molophilus; specificEpithet: corniger; scientificNameAuthorship: de Meijere, 1920; **Location:** country: Russia; stateProvince: East European Russia; county: Bashkortostan Respublika; municipality: Beloretsk district; locality: Nura River (ca. 4km W of Otnurok village), at the foot of Zolotyie Shishki (Golden Cones) Mts.; verbatimElevation: 607 m; minimumElevationInMeters: 607; decimalLatitude: 54.05155; decimalLongitude: 58.26887; **Identification:** identifiedBy: D.I. Gavryushin; **Event:** samplingProtocol: Sweep net; eventDate: 2012-08-11; verbatimEventDate: 11/Aug/2012; **Record Level:** institutionCode: ZMMU; basisOfRecord: PreservedSpecimen

#### Distribution

First records from Russia: RUE.

### Molophilus (Molophilus) crassipygus

de Meijere, 1918

18C7CFD6-A3D7-5482-BF14-5C748313EFFD

https://ccw.naturalis.nl/detail.php?id=2667

#### Materials

- **Type status:** Other material. **Occurrence:** occurrenceRemarks: 1 male; recordedBy: L.-P. Kolcsár; individualCount: 1; sex: male; preparations: Ethanol; occurrenceID: EU_LIM_585; **Taxon:** scientificName: Molophilus (Molophilus) crassipygus de Meijere, 1918; family: Limoniidae; genus: Molophilus; subgenus: Molophilus; specificEpithet: crassipygus; scientificNameAuthorship: de Meijere, 1918; **Location:** country: Latvia; municipality: Sigulda; locality: Gauja River; verbatimElevation: 13 m; minimumElevationInMeters: 13; decimalLatitude: 57.1505; decimalLongitude: 24.8168; **Identification:** identifiedBy: L.-P. Kolcsár; **Event:** samplingProtocol: Sweep net; eventDate: 2018-06-24; verbatimEventDate: 24/Jul/2018; **Record Level:** institutionCode: CKLP; basisOfRecord: PreservedSpecimen
- **Type status:** Other material. **Occurrence:** occurrenceRemarks: 1 male; recordedBy: L.-P. Kolcsár | E. Török; individualCount: 1; sex: male; preparations: Ethanol; occurrenceID: EU_LIM_586; **Taxon:** scientificName: Molophilus (Molophilus) crassipygus de Meijere, 1918; family: Limoniidae; genus: Molophilus; subgenus: Molophilus; specificEpithet: crassipygus; scientificNameAuthorship: de Meijere, 1918; **Location:** country: North Macedonia; municipality: Izvor; locality: Treska River; verbatimElevation: 755 m; minimumElevationInMeters: 755; decimalLatitude: 41.48021; decimalLongitude: 20.83466; **Identification:** identifiedBy: L.-P. Kolcsár; **Event:** samplingProtocol: Sweep net; eventDate: 2017-06-29; verbatimEventDate: 29/Jun/2017; **Record Level:** institutionCode: CKLP; basisOfRecord: PreservedSpecimen
- **Type status:** Other material. **Occurrence:** occurrenceRemarks: 1 male; recordedBy: D.I. Gavryushin; individualCount: 1; sex: male; occurrenceID: EU_LIM_587; **Taxon:** scientificName: Molophilus (Molophilus) crassipygus de Meijere, 1918; family: Limoniidae; genus: Molophilus; subgenus: Molophilus; specificEpithet: crassipygus; scientificNameAuthorship: de Meijere, 1918; **Location:** country: Russia; stateProvince: East European Russia; county: Bashkortostan Respublika; municipality: Beloretsk district; locality: Abzakovo env., Karan River; verbatimElevation: 533 m; minimumElevationInMeters: 533; decimalLatitude: 53.83717; decimalLongitude: 58.57878; **Identification:** identifiedBy: D.I. Gavryushin; **Event:** samplingProtocol: Sweep net; eventDate: 2015-06-19; verbatimEventDate: 19/Jul/2015; **Record Level:** institutionCode: ZMMU; basisOfRecord: PreservedSpecimen
- **Type status:** Other material. **Occurrence:** occurrenceRemarks: 5 males; recordedBy: D.I. Gavryushin; individualCount: 5; sex: male; occurrenceID: EU_LIM_588; **Taxon:** scientificName: Molophilus (Molophilus) crassipygus de Meijere, 1918; family: Limoniidae; genus: Molophilus; subgenus: Molophilus; specificEpithet: crassipygus; scientificNameAuthorship: de Meijere, 1918; **Location:** country: Russia; stateProvince: East European Russia; county: Bashkortostan Respublika; municipality: Beloretsk district; locality: Abzakovo env., Kulsugady River; verbatimElevation: 531 m; minimumElevationInMeters: 531; decimalLatitude: 53.83795; decimalLongitude: 58.5823; **Identification:** identifiedBy: D.I. Gavryushin; **Event:** samplingProtocol: Sweep net; eventDate: 2015-06-17; verbatimEventDate: 17/Jul/2015; **Record Level:** institutionCode: ZMMU; basisOfRecord: PreservedSpecimen
- **Type status:** Other material. **Occurrence:** occurrenceRemarks: 1 male, 1 female; recordedBy: D.I. Gavryushin; individualCount: 2; sex: male, female; occurrenceID: EU_LIM_589; **Taxon:** scientificName: Molophilus (Molophilus) crassipygus de Meijere, 1918; family: Limoniidae; genus: Molophilus; subgenus: Molophilus; specificEpithet: crassipygus; scientificNameAuthorship: de Meijere, 1918; **Location:** country: Russia; stateProvince: East European Russia; county: Bashkortostan Respublika; municipality: Beloretsk district; locality: Mata River; verbatimElevation: 552 m; minimumElevationInMeters: 552; decimalLatitude: 54.00438; decimalLongitude: 58.46398; **Identification:** identifiedBy: D.I. Gavryushin; **Event:** samplingProtocol: Sweep net; eventDate: 2012-08-07; verbatimEventDate: 07/Aug/2012; **Record Level:** institutionCode: ZMMU; basisOfRecord: PreservedSpecimen
- **Type status:** Other material. **Occurrence:** occurrenceRemarks: 1 male; recordedBy: D.I. Gavryushin; individualCount: 1; sex: male; occurrenceID: EU_LIM_590; **Taxon:** scientificName: Molophilus (Molophilus) crassipygus de Meijere, 1918; family: Limoniidae; genus: Molophilus; subgenus: Molophilus; specificEpithet: crassipygus; scientificNameAuthorship: de Meijere, 1918; **Location:** country: Russia; stateProvince: East European Russia; county: Bashkortostan Respublika; municipality: Beloretsk district; locality: Nura River (ca. 4km W of Otnurok village), at the foot of Zolotyie Shishki (Golden Cones) Mts.; verbatimElevation: 607 m; minimumElevationInMeters: 607; decimalLatitude: 54.05155; decimalLongitude: 58.26887; **Identification:** identifiedBy: D.I. Gavryushin; **Event:** samplingProtocol: Sweep net; eventDate: 2012-08-08; verbatimEventDate: 08/Aug/2012; **Record Level:** institutionCode: ZMMU; basisOfRecord: PreservedSpecimen
- **Type status:** Other material. **Occurrence:** occurrenceRemarks: 1 male; recordedBy: D.I. Gavryushin; individualCount: 1; sex: male; occurrenceID: EU_LIM_591; **Taxon:** scientificName: Molophilus (Molophilus) crassipygus de Meijere, 1918; family: Limoniidae; genus: Molophilus; subgenus: Molophilus; specificEpithet: crassipygus; scientificNameAuthorship: de Meijere, 1918; **Location:** country: Russia; stateProvince: East European Russia; county: Bashkortostan Respublika; municipality: Beloretsk district; locality: Nura River (ca. 4km W of Otnurok village), at the foot of Zolotyie Shishki (Golden Cones) Mts.; verbatimElevation: 607 m; minimumElevationInMeters: 607; decimalLatitude: 54.05155; decimalLongitude: 58.26887; **Identification:** identifiedBy: D.I. Gavryushin; **Event:** samplingProtocol: Sweep net; eventDate: 2012-08-09; verbatimEventDate: 09/Aug/2012; **Record Level:** institutionCode: ZMMU; basisOfRecord: PreservedSpecimen
- **Type status:** Other material. **Occurrence:** occurrenceRemarks: 1 male; recordedBy: D.I. Gavryushin; individualCount: 1; sex: male; occurrenceID: EU_LIM_592; **Taxon:** scientificName: Molophilus (Molophilus) crassipygus de Meijere, 1918; family: Limoniidae; genus: Molophilus; subgenus: Molophilus; specificEpithet: crassipygus; scientificNameAuthorship: de Meijere, 1918; **Location:** country: Russia; stateProvince: East European Russia; county: Bashkortostan Respublika; municipality: Beloretsk district; locality: Nura River (ca. 4km W of Otnurok village), at the foot of Zolotyie Shishki (Golden Cones) Mts.; verbatimElevation: 607 m; minimumElevationInMeters: 607; decimalLatitude: 54.05155; decimalLongitude: 58.26887; **Identification:** identifiedBy: D.I. Gavryushin; **Event:** samplingProtocol: Sweep net; eventDate: 2012-08-11; verbatimEventDate: 11/Aug/2012; **Record Level:** institutionCode: ZMMU; basisOfRecord: PreservedSpecimen
- **Type status:** Other material. **Occurrence:** occurrenceRemarks: 4 males; recordedBy: D.I. Gavryushin; individualCount: 4; sex: male; occurrenceID: EU_LIM_593; **Taxon:** scientificName: Molophilus (Molophilus) crassipygus de Meijere, 1918; family: Limoniidae; genus: Molophilus; subgenus: Molophilus; specificEpithet: crassipygus; scientificNameAuthorship: de Meijere, 1918; **Location:** country: Russia; stateProvince: East European Russia; county: Bashkortostan Respublika; municipality: Beloretsk district; locality: Nura River (ca. 4km W of Otnurok village), at the foot of Zolotyie Shishki (Golden Cones) Mts.; verbatimElevation: 607 m; minimumElevationInMeters: 607; decimalLatitude: 54.05155; decimalLongitude: 58.26887; **Identification:** identifiedBy: D.I. Gavryushin; **Event:** samplingProtocol: Sweep net; eventDate: 2015-06-13; verbatimEventDate: 13/Jul/2015; **Record Level:** institutionCode: ZMMU; basisOfRecord: PreservedSpecimen
- **Type status:** Other material. **Occurrence:** occurrenceRemarks: 1 male; recordedBy: D.I. Gavryushin; individualCount: 1; sex: male; occurrenceID: EU_LIM_594; **Taxon:** scientificName: Molophilus (Molophilus) crassipygus de Meijere, 1918; family: Limoniidae; genus: Molophilus; subgenus: Molophilus; specificEpithet: crassipygus; scientificNameAuthorship: de Meijere, 1918; **Location:** country: Russia; stateProvince: East European Russia; county: Bashkortostan Respublika; municipality: Beloretsk district; locality: Makhmutovo env., Belaya River; verbatimElevation: 550 m; minimumElevationInMeters: 550; decimalLatitude: 54.33012; decimalLongitude: 58.80735; **Identification:** identifiedBy: D.I. Gavryushin; **Event:** samplingProtocol: Sweep net; eventDate: 2015-06-15; verbatimEventDate: 15/Jul/2015; **Record Level:** institutionCode: ZMMU; basisOfRecord: PreservedSpecimen
- **Type status:** Other material. **Occurrence:** occurrenceRemarks: 1 male; recordedBy: A. Polevoi; individualCount: 1; sex: male; preparations: Pinned; occurrenceID: EU_LIM_595; **Taxon:** scientificName: Molophilus (Molophilus) crassipygus de Meijere, 1918; family: Limoniidae; genus: Molophilus; subgenus: Molophilus; specificEpithet: crassipygus; scientificNameAuthorship: de Meijere, 1918; **Location:** country: Russia; stateProvince: North European Russia; county: Republic Karelia; municipality: Segezha district; locality: Ladozero Lake; verbatimElevation: 170 m; minimumElevationInMeters: 170; decimalLatitude: 63.58763; decimalLongitude: 35.84425; **Identification:** identifiedBy: A. Polevoi; **Event:** samplingProtocol: Malaise trap; eventDate: 2010-06-27/2010-08-13; verbatimEventDate: 27/Jun-13/Aug/2010; **Record Level:** institutionCode: FRIP; basisOfRecord: PreservedSpecimen
- **Type status:** Other material. **Occurrence:** occurrenceRemarks: 1 male; recordedBy: Polevoi A.; individualCount: 1; sex: male; preparations: Pinned; occurrenceID: EU_LIM_596; **Taxon:** scientificName: Molophilus (Molophilus) crassipygus de Meijere, 1918; family: Limoniidae; genus: Molophilus; subgenus: Molophilus; specificEpithet: crassipygus; scientificNameAuthorship: de Meijere, 1918; **Location:** country: Russia; stateProvince: North European Russia; county: Republic Karelia; municipality: Prionezhskiy district; locality: Sheltozero, 3 km NW; verbatimElevation: 50 m; minimumElevationInMeters: 50; decimalLatitude: 61.39267; decimalLongitude: 35.30831; **Identification:** identifiedBy: A. Polevoi; **Event:** samplingProtocol: Sweep net; eventDate: 2004-06-13; verbatimEventDate: 13/Jul/2004; **Record Level:** institutionCode: FRIP; basisOfRecord: PreservedSpecimen
- **Type status:** Other material. **Occurrence:** occurrenceRemarks: 1 male; recordedBy: L.-P. Kolcsár | E. Török; individualCount: 1; sex: male; preparations: Ethanol; occurrenceID: EU_LIM_597; **Taxon:** scientificName: Molophilus (Molophilus) crassipygus de Meijere, 1918; family: Limoniidae; genus: Molophilus; subgenus: Molophilus; specificEpithet: crassipygus; scientificNameAuthorship: de Meijere, 1918; **Location:** country: Serbia; municipality: Sikirje; locality: Kukavica Mts.; verbatimElevation: 648 m; minimumElevationInMeters: 648; decimalLatitude: 42.65259; decimalLongitude: 21.88019; **Identification:** identifiedBy: L.-P. Kolcsár; **Event:** samplingProtocol: Sweep net; eventDate: 2017-06-02; verbatimEventDate: 2/Jul/2017; **Record Level:** institutionCode: CKLP; basisOfRecord: PreservedSpecimen

#### Description

Fig. 9

#### Distribution

First records from Latvia, North Macedonia, Russia: RUE, RUN and Serbia.

### Molophilus (Molophilus) flavus

Goetghebuer, 1920

DFE7817B-EAF6-5337-8B3D-700E5E228970

https://ccw.naturalis.nl/detail.php?id=2777

#### Materials

- **Type status:** Other material. **Occurrence:** occurrenceRemarks: 1 male; recordedBy: L.-P. Kolcsár | E. Török; individualCount: 1; sex: male; preparations: Ethanol; occurrenceID: EU_LIM_598; **Taxon:** scientificName: Molophilus (Molophilus) flavus Goetghebuer, 1920; family: Limoniidae; genus: Molophilus; subgenus: Molophilus; specificEpithet: flavus; scientificNameAuthorship: Goetghebuer, 1920; **Location:** country: Hungary; stateProvince: Heves; municipality: Szilvásvárad; locality: Bükk Mts., Szalajka Valley; verbatimElevation: 461 m; minimumElevationInMeters: 461; decimalLatitude: 48.08318; decimalLongitude: 20.40522; **Identification:** identifiedBy: L.-P. Kolcsár; **Event:** samplingProtocol: Sweep net; eventDate: 2013-08-16; verbatimEventDate: 16/Aug/2013; **Record Level:** institutionCode: CKLP; basisOfRecord: PreservedSpecimen
- **Type status:** Other material. **Occurrence:** occurrenceRemarks: 1 male; recordedBy: D.I. Gavryushin; individualCount: 1; sex: male; preparations: Pinned; occurrenceID: EU_LIM_599; **Taxon:** scientificName: Molophilus (Molophilus) flavus Goetghebuer, 1920; family: Limoniidae; genus: Molophilus; subgenus: Molophilus; specificEpithet: flavus; scientificNameAuthorship: Goetghebuer, 1920; **Location:** country: Serbia; locality: Stara Planina Mts., Babin Zub Mountain; verbatimElevation: 1550 m; minimumElevationInMeters: 1550; decimalLatitude: 43.375; decimalLongitude: 22.625; **Identification:** identifiedBy: D.I. Gavryushin; **Event:** samplingProtocol: Sweep net; eventDate: 2015-06-01/2015-07-07; verbatimEventDate: 01-07/Jul/2015; **Record Level:** institutionCode: ZMMU; basisOfRecord: PreservedSpecimen

#### Distribution

First records from Hungary and Serbia.

### Molophilus (Molophilus) griseus

(Meigen, 1804)

5EF794D0-0DBD-5EDE-AC5A-1C55DC2B9E13

https://ccw.naturalis.nl/detail.php?id=2813

#### Materials

- **Type status:** Other material. **Occurrence:** occurrenceRemarks: 1 male; recordedBy: D.I. Gavryushin; individualCount: 1; sex: male; occurrenceID: EU_LIM_600; **Taxon:** scientificName: Molophilus (Molophilus) griseus (Meigen, 1804); family: Limoniidae; genus: Molophilus; subgenus: Molophilus; specificEpithet: griseus; scientificNameAuthorship: (Meigen, 1804); **Location:** country: Russia; stateProvince: East European Russia; county: Bashkortostan Respublika; municipality: Beloretsk district; locality: Abzakovo env., Malyi Kizil River; verbatimElevation: 510 m; minimumElevationInMeters: 510; decimalLatitude: 53.81428; decimalLongitude: 58.5942; **Identification:** identifiedBy: D.I. Gavryushin; **Event:** samplingProtocol: Sweep net; eventDate: 2015-06-12; verbatimEventDate: 12/Jul/2015; **Record Level:** institutionCode: ZMMU; basisOfRecord: PreservedSpecimen
- **Type status:** Other material. **Occurrence:** occurrenceRemarks: 1 male; recordedBy: N.M. Paramonov; individualCount: 1; sex: male; occurrenceID: EU_LIM_601; **Taxon:** scientificName: Molophilus (Molophilus) griseus (Meigen, 1804); family: Limoniidae; genus: Molophilus; subgenus: Molophilus; specificEpithet: griseus; scientificNameAuthorship: (Meigen, 1804); **Location:** country: Russia; stateProvince: East European Russia; county: Tatarstan Respublika; municipality: Laishevo district; locality: Volga-Kama State Nature Biosphere Reserve, «Saraly»; verbatimElevation: 71 m; minimumElevationInMeters: 71; decimalLatitude: 55.29303; decimalLongitude: 49.29976; **Identification:** identifiedBy: N.M. Paramonov; **Event:** samplingProtocol: Sweep net; eventDate: 2009-06-18; verbatimEventDate: 18/Jun/2009; habitat: wetland; **Record Level:** institutionCode: ZIN; basisOfRecord: PreservedSpecimen
- **Type status:** Other material. **Occurrence:** occurrenceRemarks: 2 males; recordedBy: N.M. Paramonov; individualCount: 2; sex: male; occurrenceID: EU_LIM_602; **Taxon:** scientificName: Molophilus (Molophilus) griseus (Meigen, 1804); family: Limoniidae; genus: Molophilus; subgenus: Molophilus; specificEpithet: griseus; scientificNameAuthorship: (Meigen, 1804); **Location:** country: Russia; stateProvince: East European Russia; county: Tatarstan Respublika; municipality: Zelenodol’sk district; locality: Volga-Kama State Nature Biosphere Reserve, «Raifa», Lake Lenevo; verbatimElevation: 80 m; minimumElevationInMeters: 80; decimalLatitude: 55.90433; decimalLongitude: 48.79115; **Identification:** identifiedBy: N.M. Paramonov; **Event:** samplingProtocol: Sweep net; eventDate: 2012-06-26; verbatimEventDate: 26/Jun/2012; **Record Level:** institutionCode: ZIN; basisOfRecord: PreservedSpecimen
- **Type status:** Other material. **Occurrence:** occurrenceRemarks: 2 males; recordedBy: L.-P. Kolcsár | E. Török; individualCount: 2; sex: male; preparations: Ethanol; occurrenceID: EU_LIM_603; **Taxon:** scientificName: Molophilus (Molophilus) griseus (Meigen, 1804); family: Limoniidae; genus: Molophilus; subgenus: Molophilus; specificEpithet: griseus; scientificNameAuthorship: (Meigen, 1804); **Location:** country: Serbia; municipality: Bela Crkva; verbatimElevation: 78 m; minimumElevationInMeters: 78; decimalLatitude: 44.89149; decimalLongitude: 21.3861; **Identification:** identifiedBy: L.-P. Kolcsár; **Event:** samplingProtocol: Sweep net; eventDate: 2017-04-29; verbatimEventDate: 29/Apr/2017; habitat: lakes; **Record Level:** institutionCode: CKLP; basisOfRecord: PreservedSpecimen

#### Distribution

First records from Russia: RUE and Serbia.

### Molophilus (Molophilus) ibericus

Starý, 2011

7B3E60BD-015C-5FFA-9B03-FD35C133E1C6

https://ccw.naturalis.nl/detail.php?id=17590

#### Materials

- **Type status:** Other material. **Occurrence:** occurrenceRemarks: 3 males; recordedBy: J. Roháček; individualCount: 3; sex: male; preparations: Pinned; occurrenceID: EU_LIM_604; **Taxon:** scientificName: Molophilus (Molophilus) ibericus Starý, 2011; family: Limoniidae; genus: Molophilus; subgenus: Molophilus; specificEpithet: ibericus; scientificNameAuthorship: Starý, 2011; **Location:** country: Italy; stateProvince: Calabria; municipality: Mongiana; locality: 2.5 km NNE, Serre Calabresi Mts.; verbatimElevation: 1035 m; minimumElevationInMeters: 1035; decimalLatitude: 38.53583; decimalLongitude: 16.32583; **Identification:** identifiedBy: J. Starý; **Event:** eventDate: 2018-05-22; verbatimEventDate: 22/May/2018; habitat: brook; **Record Level:** institutionCode: PCJS; basisOfRecord: PreservedSpecimen
- **Type status:** Other material. **Occurrence:** occurrenceRemarks: 5 males; recordedBy: J. Starý; individualCount: 5; sex: male; preparations: Pinned; occurrenceID: EU_LIM_605; **Taxon:** scientificName: Molophilus (Molophilus) ibericus Starý, 2011; family: Limoniidae; genus: Molophilus; subgenus: Molophilus; specificEpithet: ibericus; scientificNameAuthorship: Starý, 2011; **Location:** country: Italy; stateProvince: Calabria; municipality: Mongiana; locality: 2.5 km NNE, Serre Calabresi Mts.; verbatimElevation: 1035 m; minimumElevationInMeters: 1035; decimalLatitude: 38.53583; decimalLongitude: 16.32583; **Identification:** identifiedBy: J. Starý; **Event:** eventDate: 2018-05-22; verbatimEventDate: 22/May/2018; habitat: brook; **Record Level:** institutionCode: PCJS; basisOfRecord: PreservedSpecimen
- **Type status:** Other material. **Occurrence:** occurrenceRemarks: 11 males, 1 female; recordedBy: J. Starý; individualCount: 12; sex: male, female; preparations: Pinned; occurrenceID: EU_LIM_606; **Taxon:** scientificName: Molophilus (Molophilus) ibericus Starý, 2011; family: Limoniidae; genus: Molophilus; subgenus: Molophilus; specificEpithet: ibericus; scientificNameAuthorship: Starý, 2011; **Location:** country: Italy; stateProvince: Calabria; municipality: Mongiana; locality: 2.5 km NNE, Serre Calabresi Mts.; verbatimElevation: 1035 m; minimumElevationInMeters: 1035; decimalLatitude: 38.53583; decimalLongitude: 16.32583; **Identification:** identifiedBy: J. Starý; **Event:** eventDate: 2018-05-25; verbatimEventDate: 25/May/2018; habitat: brook; **Record Level:** institutionCode: PCJS; basisOfRecord: PreservedSpecimen
- **Type status:** Other material. **Occurrence:** occurrenceRemarks: 1 male; recordedBy: J. Starý; individualCount: 1; sex: male; preparations: Pinned; occurrenceID: EU_LIM_607; **Taxon:** scientificName: Molophilus (Molophilus) ibericus Starý, 2011; family: Limoniidae; genus: Molophilus; subgenus: Molophilus; specificEpithet: ibericus; scientificNameAuthorship: Starý, 2011; **Location:** country: Italy; stateProvince: Calabria; municipality: Comerconi; locality: 0.5 km E; verbatimElevation: 350 m; minimumElevationInMeters: 350; decimalLatitude: 38.58194; decimalLongitude: 15.94833; **Identification:** identifiedBy: J. Starý; **Event:** eventDate: 2018-05-24; verbatimEventDate: 24/May/2018; habitat: brook; **Record Level:** institutionCode: PCJS; basisOfRecord: PreservedSpecimen
- **Type status:** Other material. **Occurrence:** occurrenceRemarks: 2 males; recordedBy: J. Starý; individualCount: 2; sex: male; preparations: Pinned; occurrenceID: EU_LIM_608; **Taxon:** scientificName: Molophilus (Molophilus) ibericus Starý, 2011; family: Limoniidae; genus: Molophilus; subgenus: Molophilus; specificEpithet: ibericus; scientificNameAuthorship: Starý, 2011; **Location:** country: Italy; stateProvince: Calabria; municipality: Spilinga; locality: 4 km E; verbatimElevation: 550 m; minimumElevationInMeters: 550; decimalLatitude: 38.61556; decimalLongitude: 15.94833; **Identification:** identifiedBy: J. Starý; **Event:** eventDate: 2018-05-23; verbatimEventDate: 23/May/2018; habitat: brook; **Record Level:** institutionCode: PCJS; basisOfRecord: PreservedSpecimen

#### Distribution

First records from Italy (from mainland).

### Molophilus (Molophilus) klementi

Mendl, 1973

5D2AC715-4D58-591F-A98C-C2AD8F08EFAA

https://ccw.naturalis.nl/detail.php?id=2897

#### Materials

- **Type status:** Other material. **Occurrence:** occurrenceRemarks: 1 male; recordedBy: M.C. d'Oliveira; individualCount: 1; sex: male; preparations: Ethanol; occurrenceID: EU_LIM_858; **Taxon:** scientificName: Molophilus (Molophilus) klementi Mendl, 1973; family: Limoniidae; genus: Molophilus; subgenus: Molophilus; specificEpithet: klementi; scientificNameAuthorship: Mendl, 1973; **Location:** country: Slovenia; municipality: Kranjska Gora; locality: Gozd Martuljek, 10 meters from the Sava river; verbatimElevation: 745 m; minimumElevationInMeters: 745; decimalLatitude: 46.483; decimalLongitude: 13.837539; **Identification:** identifiedBy: M.C. d'Oliveira | J. Starý; **Event:** samplingProtocol: Light trap; eventDate: 2019-20-8; verbatimEventDate: 20/August/2019; habitat: Small woodland next to river; **Record Level:** institutionCode: PCMCO; basisOfRecord: PreservedSpecimen

#### Distribution

First record from Slovenia.

### Molophilus (Molophilus) lackschewitzianus

Alexander, 1953

236A7AB3-9D9E-545D-A121-20D527445E13

https://ccw.naturalis.nl/detail.php?id=2906

#### Materials

- **Type status:** Other material. **Occurrence:** catalogNumber: 647753; occurrenceRemarks: 17 males; recordedBy: S. Olberg; individualCount: 17; sex: male; preparations: Ethanol; occurrenceID: EU_LIM_609; **Taxon:** scientificName: Molophilus (Molophilus) lackschewitzianus Alexander, 1953; family: Limoniidae; genus: Molophilus; subgenus: Molophilus; specificEpithet: lackschewitzianus; scientificNameAuthorship: Alexander, 1953; **Location:** country: Norway; stateProvince: Oslo; municipality: Oslo; locality: Lysakerelva – Møllefaret; verbatimElevation: 90 m; minimumElevationInMeters: 90; decimalLatitude: 59.9379; decimalLongitude: 10.63373; **Identification:** identifiedBy: K.M. Olsen; **Event:** samplingProtocol: Malaise trap; eventDate: 2019-06-07/2019-07-08; verbatimEventDate: 07/Jun-08/Jul/2019; **Record Level:** institutionCode: NHMO; basisOfRecord: PreservedSpecimen
- **Type status:** Other material. **Occurrence:** catalogNumber: 651892; occurrenceRemarks: 12 males; recordedBy: S. Olberg; individualCount: 12; sex: male; preparations: Ethanol; occurrenceID: EU_LIM_610; **Taxon:** scientificName: Molophilus (Molophilus) lackschewitzianus Alexander, 1953; family: Limoniidae; genus: Molophilus; subgenus: Molophilus; specificEpithet: lackschewitzianus; scientificNameAuthorship: Alexander, 1953; **Location:** country: Norway; stateProvince: Oslo; municipality: Oslo; locality: Lysakerelva – Møllefaret; verbatimElevation: 95 m; minimumElevationInMeters: 95; decimalLatitude: 59.93797; decimalLongitude: 10.63396; **Identification:** identifiedBy: K.M. Olsen; **Event:** samplingProtocol: Malaise trap; eventDate: 2019-05-13/2019-06-07; verbatimEventDate: 13/May-07/Jun/2019; **Record Level:** institutionCode: NHMO; basisOfRecord: PreservedSpecimen
- **Type status:** Other material. **Occurrence:** catalogNumber: 516354, 516355, 516356, 516357, 516358, 516359, 644184; occurrenceRemarks: 39 male+female; recordedBy: A.E. Laugsand; individualCount: 39; sex: male, female; preparations: Ethanol; occurrenceID: EU_LIM_611; **Taxon:** scientificName: Molophilus (Molophilus) lackschewitzianus Alexander, 1953; family: Limoniidae; genus: Molophilus; subgenus: Molophilus; specificEpithet: lackschewitzianus; scientificNameAuthorship: Alexander, 1953; **Location:** country: Norway; stateProvince: Østfold; municipality: Moss; locality: Reierbukta Ř; verbatimElevation: 5 m; minimumElevationInMeters: 5; decimalLatitude: 59.42451; decimalLongitude: 10.62406; **Identification:** identifiedBy: K.M. Olsen; **Event:** samplingProtocol: Malaise trap; eventDate: 2010-06-04/2010-06-30; verbatimEventDate: 04-30/Jun/2010; **Record Level:** institutionCode: PCKMO; basisOfRecord: PreservedSpecimen
- **Type status:** Other material. **Occurrence:** catalogNumber: 520088; occurrenceRemarks: 6 males; recordedBy: A.E. Laugsand; individualCount: 6; sex: male; preparations: Ethanol; occurrenceID: EU_LIM_612; **Taxon:** scientificName: Molophilus (Molophilus) lackschewitzianus Alexander, 1953; family: Limoniidae; genus: Molophilus; subgenus: Molophilus; specificEpithet: lackschewitzianus; scientificNameAuthorship: Alexander, 1953; **Location:** country: Norway; stateProvince: Østfold; municipality: Moss; locality: Reierbukta Ř; verbatimElevation: 5 m; minimumElevationInMeters: 5; decimalLatitude: 59.42451; decimalLongitude: 10.62406; **Identification:** identifiedBy: K.M. Olsen; **Event:** samplingProtocol: Malaise trap; eventDate: 2010-06-30/2010-07-23; verbatimEventDate: 30/Jun-23/Jul/2010; **Record Level:** institutionCode: NHMO; basisOfRecord: PreservedSpecimen

#### Distribution

First records from Norway.

### Molophilus (Molophilus) medius

de Meijere, 1918

D402C389-423D-506E-9A6B-1FDDBF8E87A0

https://ccw.naturalis.nl/detail.php?id=2961

#### Materials

- **Type status:** Other material. **Occurrence:** occurrenceRemarks: 1 male; recordedBy: D.I. Gavryushin; individualCount: 1; sex: male; occurrenceID: EU_LIM_613; **Taxon:** scientificName: Molophilus (Molophilus) medius de Meijere, 1918; family: Limoniidae; genus: Molophilus; subgenus: Molophilus; specificEpithet: medius; scientificNameAuthorship: de Meijere, 1918; **Location:** country: Russia; stateProvince: East European Russia; county: Bashkortostan Respublika; municipality: Beloretsk district; locality: Nura River (ca. 4km W of Otnurok village), at the foot of Zolotyie Shishki (Golden Cones) Mts.; verbatimElevation: 607 m; minimumElevationInMeters: 607; decimalLatitude: 54.05155; decimalLongitude: 58.26887; **Identification:** identifiedBy: D.I. Gavryushin; **Event:** samplingProtocol: Sweep net; eventDate: 2012-08-11; verbatimEventDate: 11/Aug/2012; **Record Level:** institutionCode: ZMMU; basisOfRecord: PreservedSpecimen
- **Type status:** Other material. **Occurrence:** occurrenceRemarks: 1 male; recordedBy: Sh.A. Murtazin; individualCount: 1; sex: male; occurrenceID: EU_LIM_614; **Taxon:** scientificName: Molophilus (Molophilus) medius de Meijere, 1918; family: Limoniidae; genus: Molophilus; subgenus: Molophilus; specificEpithet: medius; scientificNameAuthorship: de Meijere, 1918; **Location:** country: Russia; stateProvince: East European Russia; county: Bashkortostan Respublika; municipality: Beloretsk district; locality: Otnurok village; verbatimElevation: 615 m; minimumElevationInMeters: 615; decimalLatitude: 54.06062; decimalLongitude: 58.25379; **Identification:** identifiedBy: N.M. Paramonov; **Event:** samplingProtocol: Sweep net; eventDate: 2017-09-09; verbatimEventDate: 09/Sep/2017; **Record Level:** institutionCode: ZIN; basisOfRecord: PreservedSpecimen

#### Distribution

First records from Russia: RUE.

### Molophilus (Molophilus) obsoletus

Lackschewitz, 1940

8AC8FA17-E588-589A-B873-C3A63A7A6F99

https://ccw.naturalis.nl/detail.php?id=3034

#### Materials

- **Type status:** Other material. **Occurrence:** occurrenceRemarks: 1 male; recordedBy: L.-P. Kolcsár | E. Török; individualCount: 1; sex: male; preparations: Ethanol; occurrenceID: EU_LIM_615; **Taxon:** scientificName: Molophilus (Molophilus) obsoletus Lackschewitz, 1940; family: Limoniidae; genus: Molophilus; subgenus: Molophilus; specificEpithet: obsoletus; scientificNameAuthorship: Lackschewitz, 1940; **Location:** country: Serbia; municipality: Kopaonik; locality: Kopaonik Mts.; verbatimElevation: 1556 m; minimumElevationInMeters: 1556; decimalLatitude: 43.30997; decimalLongitude: 20.76563; **Identification:** identifiedBy: L.-P. Kolcsár; **Event:** samplingProtocol: Sweep net; eventDate: 2017-06-24; verbatimEventDate: 24/Jun/2017; **Record Level:** institutionCode: CKLP; basisOfRecord: PreservedSpecimen

#### Description

Fig. 10

#### Distribution

First record from Serbia.

### Molophilus (Molophilus) ochraceus

(Meigen, 1818)

F3F81DFC-3F5F-5B03-9A75-2AC96D745FB5

https://ccw.naturalis.nl/detail.php?id=3038

#### Materials

- **Type status:** Other material. **Occurrence:** occurrenceRemarks: 12 males, 1 female; recordedBy: D.I. Gavryushin; individualCount: 13; sex: male, female; preparations: Pinned; occurrenceID: EU_LIM_616; **Taxon:** scientificName: Molophilus (Molophilus) ochraceus (Meigen, 1818); family: Limoniidae; genus: Molophilus; subgenus: Molophilus; specificEpithet: ochraceus; scientificNameAuthorship: (Meigen, 1818); **Location:** country: Belarus; stateProvince: Minsk; county: Barysaw; locality: Barysaw; verbatimElevation: 155 m; minimumElevationInMeters: 155; decimalLatitude: 54.25542; decimalLongitude: 28.48092; **Identification:** identifiedBy: D.I. Gavryushin; **Event:** samplingProtocol: Sweep net; eventDate: 2013-06-05; verbatimEventDate: 5/Jul/2013; **Record Level:** institutionCode: ZMMU; basisOfRecord: PreservedSpecimen
- **Type status:** Other material. **Occurrence:** occurrenceRemarks: 1 male; recordedBy: D.I. Gavryushin; individualCount: 1; sex: male; preparations: Pinned; occurrenceID: EU_LIM_617; **Taxon:** scientificName: Molophilus (Molophilus) ochraceus (Meigen, 1818); family: Limoniidae; genus: Molophilus; subgenus: Molophilus; specificEpithet: ochraceus; scientificNameAuthorship: (Meigen, 1818); **Location:** country: Belarus; stateProvince: Minsk; county: Barysaw; locality: Vialikaje Stachava; verbatimElevation: 156 m; minimumElevationInMeters: 156; decimalLatitude: 54.26555; decimalLongitude: 28.38332; **Identification:** identifiedBy: D.I. Gavryushin; **Event:** samplingProtocol: Sweep net; eventDate: 2013-06-07; verbatimEventDate: 7/Jul/2013; **Record Level:** institutionCode: ZMMU; basisOfRecord: PreservedSpecimen
- **Type status:** Other material. **Occurrence:** occurrenceRemarks: 1 male; recordedBy: L.-P. Kolcsár; individualCount: 1; sex: male; preparations: Ethanol; occurrenceID: EU_LIM_618; **Taxon:** scientificName: Molophilus (Molophilus) ochraceus (Meigen, 1818); family: Limoniidae; genus: Molophilus; subgenus: Molophilus; specificEpithet: ochraceus; scientificNameAuthorship: (Meigen, 1818); **Location:** country: Greece; stateProvince: Eastern Macedonia and Thrace; municipality: Chrysoupoli; locality: Nea Karya, Nestos River and Delta; verbatimElevation: 3 m; minimumElevationInMeters: 3; decimalLatitude: 40.87841; decimalLongitude: 24.78541; **Identification:** identifiedBy: L.-P. Kolcsár; **Event:** samplingProtocol: Sweep net; eventDate: 2011-05-26; verbatimEventDate: 26/May/2011; **Record Level:** institutionCode: CKLP; basisOfRecord: PreservedSpecimen
- **Type status:** Other material. **Occurrence:** occurrenceRemarks: 1 male; recordedBy: D.I. Gavryushin; individualCount: 1; sex: male; occurrenceID: EU_LIM_619; **Taxon:** scientificName: Molophilus (Molophilus) ochraceus (Meigen, 1818); family: Limoniidae; genus: Molophilus; subgenus: Molophilus; specificEpithet: ochraceus; scientificNameAuthorship: (Meigen, 1818); **Location:** country: Russia; stateProvince: East European Russia; county: Bashkortostan Respublika; municipality: Beloretsk district; locality: Abzakovo env., Malyi Kizil River; verbatimElevation: 510 m; minimumElevationInMeters: 510; decimalLatitude: 53.81428; decimalLongitude: 58.5942; **Identification:** identifiedBy: D.I. Gavryushin; **Event:** samplingProtocol: Sweep net; eventDate: 2015-06-12; verbatimEventDate: 12/Jul/2015; **Record Level:** institutionCode: ZMMU; basisOfRecord: PreservedSpecimen
- **Type status:** Other material. **Occurrence:** occurrenceRemarks: 1 male; recordedBy: D.I. Gavryushin; individualCount: 1; sex: male; occurrenceID: EU_LIM_620; **Taxon:** scientificName: Molophilus (Molophilus) ochraceus (Meigen, 1818); family: Limoniidae; genus: Molophilus; subgenus: Molophilus; specificEpithet: ochraceus; scientificNameAuthorship: (Meigen, 1818); **Location:** country: Russia; stateProvince: East European Russia; county: Bashkortostan Respublika; municipality: Beloretsk district; locality: Abzakovo env., Karan River; verbatimElevation: 533 m; minimumElevationInMeters: 533; decimalLatitude: 53.83717; decimalLongitude: 58.57878; **Identification:** identifiedBy: D.I. Gavryushin; **Event:** samplingProtocol: Sweep net; eventDate: 2015-06-17; verbatimEventDate: 17/Jul/2015; **Record Level:** institutionCode: ZMMU; basisOfRecord: PreservedSpecimen
- **Type status:** Other material. **Occurrence:** occurrenceRemarks: 1 male; recordedBy: N.M. Paramonov; individualCount: 1; sex: male; occurrenceID: EU_LIM_621; **Taxon:** scientificName: Molophilus (Molophilus) ochraceus (Meigen, 1818); family: Limoniidae; genus: Molophilus; subgenus: Molophilus; specificEpithet: ochraceus; scientificNameAuthorship: (Meigen, 1818); **Location:** country: Russia; stateProvince: East European Russia; county: Tatarstan Respublika; municipality: Zelenodol’sk district; locality: Volga-Kama State Nature Biosphere Reserve, «Raifa», Lake Lenevo; verbatimElevation: 80 m; minimumElevationInMeters: 80; decimalLatitude: 55.90433; decimalLongitude: 48.79115; **Identification:** identifiedBy: N.M. Paramonov; **Event:** samplingProtocol: Sweep net; eventDate: 2012-06-26; verbatimEventDate: 26/Jun/2012; **Record Level:** institutionCode: ZIN; basisOfRecord: PreservedSpecimen

#### Distribution

First records from Belarus and Russia: RUE. We confirm the presence of the species in Greece (from mainland).

### Molophilus (Molophilus) pleuralis

de Meijere, 1920

FCB3DD09-778D-5C39-8D96-B00EBF5163C2

https://ccw.naturalis.nl/detail.php?id=3136

#### Materials

- **Type status:** Other material. **Occurrence:** catalogNumber: 529449, 644186; occurrenceRemarks: 19 male+female; recordedBy: K.M. Olsen; individualCount: 19; sex: male, female; preparations: Ethanol; occurrenceID: EU_LIM_622; **Taxon:** scientificName: Molophilus (Molophilus) pleuralis de Meijere, 1920; family: Limoniidae; genus: Molophilus; subgenus: Molophilus; specificEpithet: pleuralis; scientificNameAuthorship: de Meijere, 1920; **Location:** country: Norway; stateProvince: Østfold; municipality: Hvaler; locality: Utgårdskilen; verbatimElevation: 0-1 m; maximumElevationInMeters: 1; decimalLatitude: 59.08321; decimalLongitude: 10.88332; **Identification:** identifiedBy: K.M. Olsen; **Event:** samplingProtocol: Sweep net; eventDate: 2017-08-21; verbatimEventDate: 21/Aug/2017; habitat: Strandengene rundt innerste delen av kilen; **Record Level:** institutionCode: PCKMO; basisOfRecord: PreservedSpecimen
- **Type status:** Other material. **Occurrence:** catalogNumber: 582359; occurrenceRemarks: 1 male; recordedBy: K.M. Olsen | T. Starholm; individualCount: 1; sex: male; preparations: Ethanol; occurrenceID: EU_LIM_623; **Taxon:** scientificName: Molophilus (Molophilus) pleuralis de Meijere, 1920; family: Limoniidae; genus: Molophilus; subgenus: Molophilus; specificEpithet: pleuralis; scientificNameAuthorship: de Meijere, 1920; **Location:** country: Norway; stateProvince: Østfold; municipality: Hvaler; locality: SW Skjellvik; verbatimElevation: 0.5 m; minimumElevationInMeters: 0.5; decimalLatitude: 59.05225; decimalLongitude: 10.92163; **Identification:** identifiedBy: K.M. Olsen; **Event:** samplingProtocol: Sweep net; eventDate: 2018-06-17; verbatimEventDate: 17/Jun/2018; habitat: Ved dammen; **Record Level:** institutionCode: NHMO; basisOfRecord: PreservedSpecimen
- **Type status:** Other material. **Occurrence:** catalogNumber: 599043; occurrenceRemarks: 2 males; recordedBy: K. Berggren; individualCount: 2; sex: male; occurrenceID: EU_LIM_624; **Taxon:** scientificName: Molophilus (Molophilus) pleuralis de Meijere, 1920; family: Limoniidae; genus: Molophilus; subgenus: Molophilus; specificEpithet: pleuralis; scientificNameAuthorship: de Meijere, 1920; **Location:** country: Norway; stateProvince: Vest-Agder; municipality: Kristiansand; locality: Nedre Timenes; verbatimElevation: 10 m; minimumElevationInMeters: 10; decimalLatitude: 58.16155; decimalLongitude: 8.10013; **Identification:** identifiedBy: K.M. Olsen; **Event:** samplingProtocol: Light trap; eventDate: 2017-06; verbatimEventDate: Jul/2017; **Record Level:** basisOfRecord: HumanObservation
- **Type status:** Other material. **Occurrence:** catalogNumber: 563686; occurrenceRemarks: 8 males; recordedBy: K. Berggren; individualCount: 8; sex: male; preparations: Ethanol; occurrenceID: EU_LIM_625; **Taxon:** scientificName: Molophilus (Molophilus) pleuralis de Meijere, 1920; family: Limoniidae; genus: Molophilus; subgenus: Molophilus; specificEpithet: pleuralis; scientificNameAuthorship: de Meijere, 1920; **Location:** country: Norway; stateProvince: Vest-Agder; municipality: Kristiansand; locality: Nedre Timenes; verbatimElevation: 10 m; minimumElevationInMeters: 10; decimalLatitude: 58.16155; decimalLongitude: 8.10013; **Identification:** identifiedBy: K.M. Olsen; **Event:** samplingProtocol: Light trap; eventDate: 2017-06/2017-09; verbatimEventDate: Jul-Sep/2017; **Record Level:** institutionCode: NHMO; basisOfRecord: PreservedSpecimen
- **Type status:** Other material. **Occurrence:** catalogNumber: 600602; occurrenceRemarks: 1 male; recordedBy: K. Berggren; individualCount: 1; sex: male; preparations: Ethanol; occurrenceID: EU_LIM_626; **Taxon:** scientificName: Molophilus (Molophilus) pleuralis de Meijere, 1920; family: Limoniidae; genus: Molophilus; subgenus: Molophilus; specificEpithet: pleuralis; scientificNameAuthorship: de Meijere, 1920; **Location:** country: Norway; stateProvince: Vest-Agder; municipality: Kristiansand; locality: Bråvann terrasse – I hagen til nr. 21; verbatimElevation: 75 m; minimumElevationInMeters: 75; decimalLatitude: 58.11029; decimalLongitude: 7.93429; **Identification:** identifiedBy: K.M. Olsen; **Event:** samplingProtocol: Light trap; eventDate: 2017-08; verbatimEventDate: Aug/2017; **Record Level:** institutionCode: NHMO; basisOfRecord: PreservedSpecimen
- **Type status:** Other material. **Occurrence:** catalogNumber: 531868; occurrenceRemarks: 1 male; recordedBy: K. Berggren; individualCount: 1; sex: male; preparations: Ethanol; occurrenceID: EU_LIM_627; **Taxon:** scientificName: Molophilus (Molophilus) pleuralis de Meijere, 1920; family: Limoniidae; genus: Molophilus; subgenus: Molophilus; specificEpithet: pleuralis; scientificNameAuthorship: de Meijere, 1920; **Location:** country: Norway; stateProvince: Vest-Agder; municipality: Kristiansand; locality: Nedre Timenes; verbatimElevation: 10 m; minimumElevationInMeters: 10; decimalLatitude: 58.16155; decimalLongitude: 8.10013; **Identification:** identifiedBy: K.M. Olsen; **Event:** samplingProtocol: Light trap; eventDate: 2017-06-01/2017-06-19; verbatimEventDate: 01-19/Jun/2017; **Record Level:** institutionCode: NHMO; basisOfRecord: PreservedSpecimen
- **Type status:** Other material. **Occurrence:** catalogNumber: 532531; occurrenceRemarks: 1 male; recordedBy: K. Berggren; individualCount: 1; sex: male; preparations: Ethanol; occurrenceID: EU_LIM_628; **Taxon:** scientificName: Molophilus (Molophilus) pleuralis de Meijere, 1920; family: Limoniidae; genus: Molophilus; subgenus: Molophilus; specificEpithet: pleuralis; scientificNameAuthorship: de Meijere, 1920; **Location:** country: Norway; stateProvince: Vest-Agder; municipality: Kristiansand; locality: Nedre Timenes; verbatimElevation: 10 m; minimumElevationInMeters: 10; decimalLatitude: 58.16155; decimalLongitude: 8.10013; **Identification:** identifiedBy: K.M. Olsen; **Event:** samplingProtocol: Light trap; eventDate: 2017-06-01/2017-06-19; verbatimEventDate: 01-19/Jun/2017; **Record Level:** institutionCode: NHMO; basisOfRecord: PreservedSpecimen
- **Type status:** Other material. **Occurrence:** occurrenceRemarks: 1 male; recordedBy: E. Eiroa; individualCount: 1; sex: male; preparations: Pinned; occurrenceID: EU_LIM_629; **Taxon:** scientificName: Molophilus (Molophilus) pleuralis de Meijere, 1920; family: Limoniidae; genus: Molophilus; subgenus: Molophilus; specificEpithet: pleuralis; scientificNameAuthorship: de Meijere, 1920; **Location:** country: Spain; stateProvince: Galicia, La Coruña; municipality: Riveira; locality: Corrubedo, laguna Carregal; verbatimElevation: 0 m; decimalLatitude: 42.57607; decimalLongitude: -9.03986; **Identification:** identifiedBy: E. Eiroa; **Event:** samplingProtocol: Sweep net; eventDate: 1995-04-10; verbatimEventDate: 10/April/1995; habitat: saltmarsh; **Record Level:** institutionCode: USC; basisOfRecord: PreservedSpecimen

#### Distribution

First records from Norway. Species previously reported form Balearic Islands and here, we report the first records from mainland Spain.

### Molophilus (Molophilus) propinquus

(Egger, 1863)

BCD7F1E8-FF5A-55D0-A544-19AE097E4B5B

https://ccw.naturalis.nl/detail.php?id=3157

#### Materials

- **Type status:** Other material. **Occurrence:** occurrenceRemarks: 1 male; recordedBy: D.I. Gavryushin; individualCount: 1; sex: male; preparations: Pinned; occurrenceID: EU_LIM_630; **Taxon:** scientificName: Molophilus (Molophilus) propinquus (Egger, 1863); family: Limoniidae; genus: Molophilus; subgenus: Molophilus; specificEpithet: propinquus; scientificNameAuthorship: (Egger, 1863); **Location:** country: Belarus; stateProvince: Minsk; county: Barysaw; locality: Glivin; verbatimElevation: 161 m; minimumElevationInMeters: 161; decimalLatitude: 54.14902; decimalLongitude: 28.63648; **Identification:** identifiedBy: D.I. Gavryushin; **Event:** samplingProtocol: Sweep net; eventDate: 2013-06-06; verbatimEventDate: 6/Jul/2013; **Record Level:** institutionCode: ZMMU; basisOfRecord: PreservedSpecimen
- **Type status:** Other material. **Occurrence:** occurrenceRemarks: 1 male; recordedBy: D.I. Gavryushin; individualCount: 1; sex: male; preparations: Pinned; occurrenceID: EU_LIM_631; **Taxon:** scientificName: Molophilus (Molophilus) propinquus (Egger, 1863); family: Limoniidae; genus: Molophilus; subgenus: Molophilus; specificEpithet: propinquus; scientificNameAuthorship: (Egger, 1863); **Location:** country: Belarus; stateProvince: Minsk; county: Barysaw; locality: Barysaw; verbatimElevation: 155 m; minimumElevationInMeters: 155; decimalLatitude: 54.25542; decimalLongitude: 28.48092; **Identification:** identifiedBy: D.I. Gavryushin; **Event:** samplingProtocol: Sweep net; eventDate: 2013-06-05; verbatimEventDate: 5/Jul/2013; **Record Level:** institutionCode: ZMMU; basisOfRecord: PreservedSpecimen
- **Type status:** Other material. **Occurrence:** occurrenceRemarks: 2 male, 1 female; recordedBy: V.I. Lantsov; individualCount: 3; sex: male, female; preparations: Pinned; occurrenceID: EU_LIM_632; **Taxon:** scientificName: Molophilus (Molophilus) propinquus (Egger, 1863); family: Limoniidae; genus: Molophilus; subgenus: Molophilus; specificEpithet: propinquus; scientificNameAuthorship: (Egger, 1863); **Location:** country: Russia; stateProvince: North Caucasus; county: Republic of Dagestan; municipality: Kumtorkalinsky, Makhachkala; locality: the Sary-Kum Dune, 15 km to the W from Makhachkala, Dagestan State Nature Reserve, River Valley. Shura-Ozen left bank; verbatimElevation: 66 m; minimumElevationInMeters: 66; decimalLatitude: 43.04361; decimalLongitude: 47.30722; **Identification:** identifiedBy: V.I. Lantsov; **Event:** samplingProtocol: Sweep net; eventDate: 2014-05-14; verbatimEventDate: 14/May/2014; habitat: grass near the water; **Record Level:** institutionCode: ZIN; basisOfRecord: PreservedSpecimen

#### Distribution

First records from Belarus and Russia: NC.

### Molophilus (Molophilus) pullus

Lackschewitz, 1927

8FA0CF43-CD78-57E9-9FB9-4DAD7ED3523A

https://ccw.naturalis.nl/detail.php?id=3168

#### Materials

- **Type status:** Other material. **Occurrence:** catalogNumber: 657075; occurrenceRemarks: 1 male; recordedBy: K.M. Olsen; individualCount: 1; sex: male; preparations: Ethanol; occurrenceID: EU_LIM_633; **Taxon:** scientificName: Molophilus (Molophilus) pullus Lackschewitz, 1927; family: Limoniidae; genus: Molophilus; subgenus: Molophilus; specificEpithet: pullus; scientificNameAuthorship: Lackschewitz, 1927; **Location:** country: Norway; stateProvince: Buskerud; municipality: Ringerike; locality: Veksalbekken; verbatimElevation: 95 m; minimumElevationInMeters: 95; decimalLatitude: 60.17397; decimalLongitude: 10.19885; **Identification:** identifiedBy: K.M. Olsen; **Event:** samplingProtocol: Malaise trap; eventDate: 2017-05-02/2017-06-23; verbatimEventDate: 02/May-23/Jun/2017; **Record Level:** institutionCode: PCKMO; basisOfRecord: PreservedSpecimen
- **Type status:** Other material. **Occurrence:** occurrenceRemarks: 1 male; recordedBy: V.E. Pilipenko; individualCount: 1; sex: male; occurrenceID: EU_LIM_634; **Taxon:** scientificName: Molophilus (Molophilus) pullus Lackschewitz, 1927; family: Limoniidae; genus: Molophilus; subgenus: Molophilus; specificEpithet: pullus; scientificNameAuthorship: Lackschewitz, 1927; **Location:** country: Russia; stateProvince: Central European Russia; county: Moskovskaya Oblast; municipality: Solnechnogorsk district; locality: Chashnikovo; verbatimElevation: 220 m; minimumElevationInMeters: 220; decimalLatitude: 56.0375; decimalLongitude: 37.1874; **Identification:** identifiedBy: V.E. Pilipenko; **Event:** samplingProtocol: Sweep net; eventDate: 1992-05-20; verbatimEventDate: 20/May/1992; **Record Level:** institutionCode: VPMC; basisOfRecord: PreservedSpecimen
- **Type status:** Other material. **Occurrence:** occurrenceRemarks: 2 males, 4 females; recordedBy: V.E. Pilipenko; individualCount: 6; sex: male, female; occurrenceID: EU_LIM_635; **Taxon:** scientificName: Molophilus (Molophilus) pullus Lackschewitz, 1927; family: Limoniidae; genus: Molophilus; subgenus: Molophilus; specificEpithet: pullus; scientificNameAuthorship: Lackschewitz, 1927; **Location:** country: Russia; stateProvince: Central European Russia; county: Moskovskaya Oblast; municipality: Solnechnogorsk district; locality: Chashnikovo; verbatimElevation: 220 m; minimumElevationInMeters: 220; decimalLatitude: 56.0375; decimalLongitude: 37.1874; **Identification:** identifiedBy: V.E. Pilipenko; **Event:** samplingProtocol: Sweep net; eventDate: 1995-05-07; verbatimEventDate: 07/May/1995; **Record Level:** institutionCode: VPMC; basisOfRecord: PreservedSpecimen

#### Distribution

First records from Norway and Russia: RUC.

### Molophilus (Molophilus) repentinus

Starý, 1971

D787C82D-B359-5B9B-99D4-3454635B08D3

https://ccw.naturalis.nl/detail.php?id=3190

#### Materials

- **Type status:** Other material. **Occurrence:** occurrenceRemarks: 12 males, 2 females; recordedBy: D.I. Gavryushin; individualCount: 14; sex: male, female; preparations: Pinned; occurrenceID: EU_LIM_636; **Taxon:** scientificName: Molophilus (Molophilus) repentinus Starý, 1971; family: Limoniidae; genus: Molophilus; subgenus: Molophilus; specificEpithet: repentinus; scientificNameAuthorship: Starý, 1971; **Location:** country: Serbia; stateProvince: Zaječar; municipality: Knjaževac; locality: Crni Vrh; verbatimElevation: 800 m; minimumElevationInMeters: 800; decimalLatitude: 43.407; decimalLongitude: 22.587; **Identification:** identifiedBy: D.I. Gavryushin; **Event:** samplingProtocol: Sweep net; eventDate: 2015-05-01/2015-05-08; verbatimEventDate: 01-08/May/2015; **Record Level:** institutionCode: ZMMU; basisOfRecord: PreservedSpecimen
- **Type status:** Other material. **Occurrence:** occurrenceRemarks: 2 males; recordedBy: D.I. Gavryushin; individualCount: 2; sex: male; preparations: Pinned; occurrenceID: EU_LIM_637; **Taxon:** scientificName: Molophilus (Molophilus) repentinus Starý, 1971; family: Limoniidae; genus: Molophilus; subgenus: Molophilus; specificEpithet: repentinus; scientificNameAuthorship: Starý, 1971; **Location:** country: Serbia; stateProvince: Zaječar; municipality: Knjaževac; locality: Crni Vrh; verbatimElevation: 800 m; minimumElevationInMeters: 800; decimalLatitude: 43.407; decimalLongitude: 22.587; **Identification:** identifiedBy: D.I. Gavryushin; **Event:** samplingProtocol: Sweep net; eventDate: 2015-06-01/2015-07-07; verbatimEventDate: 01-07/Jul/2015; **Record Level:** institutionCode: ZMMU; basisOfRecord: PreservedSpecimen
- **Type status:** Other material. **Occurrence:** occurrenceRemarks: 4 males, 1 female; recordedBy: D.I. Gavryushin; individualCount: 5; sex: male, female; preparations: Ethanol; occurrenceID: EU_LIM_638; **Taxon:** scientificName: Molophilus (Molophilus) repentinus Starý, 1971; family: Limoniidae; genus: Molophilus; subgenus: Molophilus; specificEpithet: repentinus; scientificNameAuthorship: Starý, 1971; **Location:** country: Serbia; locality: Stara Planina Mts.; verbatimElevation: 1030 m; minimumElevationInMeters: 1030; decimalLatitude: 43.396; decimalLongitude: 22.607; **Identification:** identifiedBy: D.I. Gavryushin; **Event:** samplingProtocol: Sweep net; eventDate: 2015-05-01/2015-05-08; verbatimEventDate: 01-08/May/2015; **Record Level:** institutionCode: ZMMU; basisOfRecord: PreservedSpecimen

#### Distribution

First records from Serbia.

### Molophilus (Molophilus) serpentiger

Edwards, 1938

3022BDDF-5098-538A-8706-01BE4D26958A

https://ccw.naturalis.nl/detail.php?id=3218

#### Materials

- **Type status:** Other material. **Occurrence:** catalogNumber: 581916; occurrenceRemarks: 3 males; recordedBy: K.M. Olsen; individualCount: 3; sex: male; preparations: Ethanol; occurrenceID: EU_LIM_639; **Taxon:** scientificName: Molophilus (Molophilus) serpentiger Edwards, 1938; family: Limoniidae; genus: Molophilus; subgenus: Molophilus; specificEpithet: serpentiger; scientificNameAuthorship: Edwards, 1938; **Location:** country: Norway; stateProvince: Akershus; municipality: Ski; locality: Kapelldammen; verbatimElevation: 125 m; minimumElevationInMeters: 125; decimalLatitude: 59.72433; decimalLongitude: 10.8386; **Identification:** identifiedBy: K.M. Olsen; **Event:** samplingProtocol: Sweep net; eventDate: 2018-06-12; verbatimEventDate: 12/Jun/2018; **Record Level:** institutionCode: NHMO; basisOfRecord: PreservedSpecimen
- **Type status:** Other material. **Occurrence:** catalogNumber: 528362; occurrenceRemarks: 1 male; recordedBy: K.M. Olsen; individualCount: 1; sex: male; preparations: Ethanol; occurrenceID: EU_LIM_640; **Taxon:** scientificName: Molophilus (Molophilus) serpentiger Edwards, 1938; family: Limoniidae; genus: Molophilus; subgenus: Molophilus; specificEpithet: serpentiger; scientificNameAuthorship: Edwards, 1938; **Location:** country: Norway; stateProvince: Akershus; municipality: Skedsmo; locality: SSE Buhaugen; verbatimElevation: 110 m; minimumElevationInMeters: 110; decimalLatitude: 59.97227; decimalLongitude: 10.98641; **Identification:** identifiedBy: K.M. Olsen; **Event:** samplingProtocol: Sweep net; eventDate: 2017-06-20; verbatimEventDate: 20/Jun/2017; **Record Level:** institutionCode: NHMO; basisOfRecord: PreservedSpecimen
- **Type status:** Other material. **Occurrence:** catalogNumber: 559663; occurrenceRemarks: 3 males; recordedBy: K.M. Olsen; individualCount: 3; sex: male; preparations: Ethanol; occurrenceID: EU_LIM_641; **Taxon:** scientificName: Molophilus (Molophilus) serpentiger Edwards, 1938; family: Limoniidae; genus: Molophilus; subgenus: Molophilus; specificEpithet: serpentiger; scientificNameAuthorship: Edwards, 1938; **Location:** country: Norway; stateProvince: Akershus; municipality: Skedsmo; locality: N Asak Mellom; verbatimElevation: 150 m; minimumElevationInMeters: 150; decimalLatitude: 59.98719; decimalLongitude: 11.10035; **Identification:** identifiedBy: K.M. Olsen; **Event:** samplingProtocol: Malaise trap; eventDate: 2017-06-20/2017-07-26; verbatimEventDate: 20/Jun-26/Jul/2017; **Record Level:** institutionCode: NHMO; basisOfRecord: PreservedSpecimen
- **Type status:** Other material. **Occurrence:** catalogNumber: 566052; occurrenceRemarks: 1 male; recordedBy: K.M. Olsen; individualCount: 1; sex: male; preparations: Ethanol; occurrenceID: EU_LIM_642; **Taxon:** scientificName: Molophilus (Molophilus) serpentiger Edwards, 1938; family: Limoniidae; genus: Molophilus; subgenus: Molophilus; specificEpithet: serpentiger; scientificNameAuthorship: Edwards, 1938; **Location:** country: Norway; stateProvince: Buskerud; municipality: Ringerike; locality: Veksalbekken; verbatimElevation: 95 m; minimumElevationInMeters: 95; decimalLatitude: 60.17397; decimalLongitude: 10.19885; **Identification:** identifiedBy: K.M. Olsen; **Event:** samplingProtocol: SLAM-trap; eventDate: 2017-05-02/2017-06-23; verbatimEventDate: 02/May-23/Jun/2017; **Record Level:** institutionCode: NHMO; basisOfRecord: PreservedSpecimen
- **Type status:** Other material. **Occurrence:** catalogNumber: 549360; occurrenceRemarks: 1 male; recordedBy: K.M. Olsen; individualCount: 1; sex: male; preparations: Ethanol; occurrenceID: EU_LIM_643; **Taxon:** scientificName: Molophilus (Molophilus) serpentiger Edwards, 1938; family: Limoniidae; genus: Molophilus; subgenus: Molophilus; specificEpithet: serpentiger; scientificNameAuthorship: Edwards, 1938; **Location:** country: Norway; stateProvince: Buskerud; municipality: Ringerike; locality: Veksalbekken; verbatimElevation: 90-110 m; minimumElevationInMeters: 90; maximumElevationInMeters: 110; decimalLatitude: 60.17544; decimalLongitude: 10.19964; **Identification:** identifiedBy: K.M. Olsen; **Event:** samplingProtocol: Sweep net; eventDate: 2017-06-23; verbatimEventDate: 23/Jun/2017; **Record Level:** institutionCode: NHMO; basisOfRecord: PreservedSpecimen
- **Type status:** Other material. **Occurrence:** catalogNumber: 568982; occurrenceRemarks: 1 male; recordedBy: K.M. Olsen; individualCount: 1; sex: male; preparations: Ethanol; occurrenceID: EU_LIM_644; **Taxon:** scientificName: Molophilus (Molophilus) serpentiger Edwards, 1938; family: Limoniidae; genus: Molophilus; subgenus: Molophilus; specificEpithet: serpentiger; scientificNameAuthorship: Edwards, 1938; **Location:** country: Norway; stateProvince: Buskerud; municipality: Ringerike; locality: Veksalbekken; verbatimElevation: 95 m; minimumElevationInMeters: 95; decimalLatitude: 60.17397; decimalLongitude: 10.19885; **Identification:** identifiedBy: K.M. Olsen; **Event:** samplingProtocol: Malaise trap; eventDate: 2017-06-23/2017-08-11; verbatimEventDate: 23/Jun-11/Aug/2017; **Record Level:** institutionCode: NHMO; basisOfRecord: PreservedSpecimen
- **Type status:** Other material. **Occurrence:** catalogNumber: 524898, 644187; occurrenceRemarks: 2 males; recordedBy: K.M. Olsen; individualCount: 2; sex: male; preparations: Ethanol; occurrenceID: EU_LIM_645; **Taxon:** scientificName: Molophilus (Molophilus) serpentiger Edwards, 1938; family: Limoniidae; genus: Molophilus; subgenus: Molophilus; specificEpithet: serpentiger; scientificNameAuthorship: Edwards, 1938; **Location:** country: Norway; stateProvince: Buskerud; municipality: Hurum; locality: Sandspollen – Kapellkilen; verbatimElevation: 0-1 m; maximumElevationInMeters: 1; decimalLatitude: 59.66445; decimalLongitude: 10.58944; **Identification:** identifiedBy: K.M. Olsen; **Event:** samplingProtocol: Sweep net; eventDate: 2017-05-31; verbatimEventDate: 31/May/2017; **Record Level:** institutionCode: PCKMO; basisOfRecord: PreservedSpecimen
- **Type status:** Other material. **Occurrence:** catalogNumber: 587663; occurrenceRemarks: 1 male; recordedBy: S. Olberg; individualCount: 1; sex: male; preparations: Ethanol; occurrenceID: EU_LIM_646; **Taxon:** scientificName: Molophilus (Molophilus) serpentiger Edwards, 1938; family: Limoniidae; genus: Molophilus; subgenus: Molophilus; specificEpithet: serpentiger; scientificNameAuthorship: Edwards, 1938; **Location:** country: Norway; stateProvince: Buskerud; municipality: Hole; locality: Fjulsrud; verbatimElevation: 180 m; minimumElevationInMeters: 180; decimalLatitude: 59.97035; decimalLongitude: 10.32397; **Identification:** identifiedBy: K.M. Olsen; **Event:** samplingProtocol: Window trap; eventDate: 2018-06-04/2018-07-02; verbatimEventDate: 04/Jun-02/Jul/2018; **Record Level:** institutionCode: NHMO; basisOfRecord: PreservedSpecimen
- **Type status:** Other material. **Occurrence:** catalogNumber: 548909; occurrenceRemarks: 2 males; recordedBy: K.M. Olsen | S. Olberg; individualCount: 2; sex: male; preparations: Ethanol; occurrenceID: EU_LIM_647; **Taxon:** scientificName: Molophilus (Molophilus) serpentiger Edwards, 1938; family: Limoniidae; genus: Molophilus; subgenus: Molophilus; specificEpithet: serpentiger; scientificNameAuthorship: Edwards, 1938; **Location:** country: Norway; stateProvince: Vestfold; municipality: Larvik; locality: Sandvikbukta PFO; verbatimElevation: 2 m; minimumElevationInMeters: 2; decimalLatitude: 59.01169; decimalLongitude: 10.14128; **Identification:** identifiedBy: K.M. Olsen; **Event:** samplingProtocol: Sweep net; eventDate: 2017-06-06; verbatimEventDate: 06/Jun/2017; **Record Level:** institutionCode: NHMO; basisOfRecord: PreservedSpecimen

#### Distribution

First records from Norway.

### Molophilus (Molophilus) undulatus

Tonnoir, 1920

C624EBD7-A6B6-56A8-8C63-335F17A9E0AD

https://ccw.naturalis.nl/detail.php?id=3346

#### Materials

- **Type status:** Other material. **Occurrence:** occurrenceRemarks: 1 male; recordedBy: F. Mihályi; individualCount: 1; sex: male; preparations: Pinned; occurrenceID: EU_LIM_648; **Taxon:** scientificName: Molophilus (Molophilus) undulatus Tonnoir, 1920; family: Limoniidae; genus: Molophilus; subgenus: Molophilus; specificEpithet: undulatus; scientificNameAuthorship: Tonnoir, 1920; **Location:** country: Hungary; stateProvince: Heves; municipality: Parád; locality: Mátrai Landscape Park, Pisztrángostó; decimalLatitude: 47.88164; decimalLongitude: 20.01358; **Identification:** identifiedBy: L.-P. Kolcsár; **Event:** eventDate: 1954-09-09; verbatimEventDate: 09/Sep/1954; **Record Level:** institutionCode: HNHM; basisOfRecord: PreservedSpecimen

#### Distribution

First record from Hungary.

### Molophilus (Molophilus) urodontus

Savchenko, 1978

8F103D2D-E60B-5854-BF09-A4DBE6BD4AFB

https://ccw.naturalis.nl/detail.php?id=3358

#### Materials

- **Type status:** Other material. **Occurrence:** occurrenceRemarks: 1 male, 1 male; recordedBy: V.I. Lantsov; individualCount: 2; sex: male, female; preparations: Pinned; occurrenceID: EU_LIM_649; **Taxon:** scientificName: Molophilus (Molophilus) urodontus Savchenko, 1978; family: Limoniidae; genus: Molophilus; subgenus: Molophilus; specificEpithet: urodontus; scientificNameAuthorship: Savchenko, 1978; **Location:** country: Russia; stateProvince: North Caucasus; county: Republic of Dagestan; municipality: Magaramkent, Samur; locality: Dagestan Nature Reserve. Samur liana forest; verbatimElevation: 12 m; minimumElevationInMeters: 12; decimalLatitude: 42.07306; decimalLongitude: 48.69417; **Identification:** identifiedBy: V.I. Lantsov; **Event:** samplingProtocol: Sweep net; eventDate: 2014-05-16; verbatimEventDate: 16/May/2014; habitat: hornbeam and spurge liana forest, with an admixture of oak, maple and high ash - elderberry, horsetail, plants along the stream; **Record Level:** institutionCode: ZIN; basisOfRecord: PreservedSpecimen
- **Type status:** Other material. **Occurrence:** catalogNumber: vial No3; occurrenceRemarks: 1 male; recordedBy: V.I. Lantsov; individualCount: 1; sex: male; preparations: Ethanol; occurrenceID: EU_LIM_650; **Taxon:** scientificName: Molophilus (Molophilus) urodontus Savchenko, 1978; family: Limoniidae; genus: Molophilus; subgenus: Molophilus; specificEpithet: urodontus; scientificNameAuthorship: Savchenko, 1978; **Location:** country: Russia; stateProvince: North Caucasus; county: Republic of Dagestan; municipality: Tlyarata, Salda; locality: Dagestan Nature Reserve. in vicinity of village Salda, spring slope right bank of the Dzhurmut River; verbatimElevation: 1794 m; minimumElevationInMeters: 1794; decimalLatitude: 42.08667; decimalLongitude: 48.60056; **Identification:** identifiedBy: V.I. Lantsov; **Event:** samplingProtocol: Sweep net; eventDate: 2016-06-06; verbatimEventDate: 06/Jul/2016; **Record Level:** institutionCode: ZIN; basisOfRecord: PreservedSpecimen
- **Type status:** Other material. **Occurrence:** occurrenceRemarks: 4 males, 1 female; recordedBy: V.I. Lantsov; individualCount: 5; sex: male, female; preparations: Pinned; occurrenceID: EU_LIM_651; **Taxon:** scientificName: Molophilus (Molophilus) urodontus Savchenko, 1978; family: Limoniidae; genus: Molophilus; subgenus: Molophilus; specificEpithet: urodontus; scientificNameAuthorship: Savchenko, 1978; **Location:** country: Russia; stateProvince: North Caucasus; county: Republic of Dagestan; municipality: Magaramkent, Samur; locality: Dagestan Nature Reserve. Samur liana forest, in vicinity of village Bil’bil’- Kasmalyar; verbatimElevation: 11 m; minimumElevationInMeters: 11; decimalLatitude: 41.85667; decimalLongitude: 48.60056; **Identification:** identifiedBy: V.I. Lantsov; **Event:** samplingProtocol: Sweep net; eventDate: 2016-05-22; verbatimEventDate: 22/May/2016; habitat: biotope 3, stream in the forest, sweeping on grass; **Record Level:** institutionCode: IEMT; basisOfRecord: PreservedSpecimen

#### Distribution

First records from Russia: NC.

### Neolimnomyia batava

(Edwards, 1938)

D712E096-4404-5AEF-8BBE-CA69504A3BBF

https://ccw.naturalis.nl/detail.php?id=6903

#### Materials

- **Type status:** Other material. **Occurrence:** occurrenceRemarks: 1 male; recordedBy: V.E. Pilipenko; individualCount: 1; sex: male; occurrenceID: EU_LIM_652; **Taxon:** scientificName: Neolimnomyiabatava (Edwards, 1938); family: Limoniidae; genus: Neolimnomyia; specificEpithet: batava; scientificNameAuthorship: (Edwards, 1938); **Location:** country: Russia; stateProvince: Central European Russia; county: Moskovskaya Oblast; municipality: Solnechnogorsk district; locality: Chashnikovo; verbatimElevation: 220 m; minimumElevationInMeters: 220; decimalLatitude: 56.0375; decimalLongitude: 37.1874; **Identification:** identifiedBy: V.E. Pilipenko; **Event:** samplingProtocol: Sweep net; eventDate: 1992-06-03; verbatimEventDate: 03/Jun/1992; **Record Level:** institutionCode: VPMC; basisOfRecord: PreservedSpecimen
- **Type status:** Other material. **Occurrence:** occurrenceRemarks: 4 males; recordedBy: V.E. Pilipenko; individualCount: 4; sex: male; occurrenceID: EU_LIM_653; **Taxon:** scientificName: Neolimnomyiabatava (Edwards, 1938); family: Limoniidae; genus: Neolimnomyia; specificEpithet: batava; scientificNameAuthorship: (Edwards, 1938); **Location:** country: Russia; stateProvince: Central European Russia; county: Moskovskaya Oblast; municipality: Solnechnogorsk district; locality: Chashnikovo; verbatimElevation: 220 m; minimumElevationInMeters: 220; decimalLatitude: 56.0375; decimalLongitude: 37.1874; **Identification:** identifiedBy: V.E. Pilipenko; **Event:** samplingProtocol: Sweep net; eventDate: 1992-06-14; verbatimEventDate: 14/Jun/1992; **Record Level:** institutionCode: VPMC; basisOfRecord: PreservedSpecimen
- **Type status:** Other material. **Occurrence:** occurrenceRemarks: 1 female; recordedBy: V.E. Pilipenko; individualCount: 1; sex: female; occurrenceID: EU_LIM_654; **Taxon:** scientificName: Neolimnomyiabatava (Edwards, 1938); family: Limoniidae; genus: Neolimnomyia; specificEpithet: batava; scientificNameAuthorship: (Edwards, 1938); **Location:** country: Russia; stateProvince: Central European Russia; county: Moskovskaya Oblast; municipality: Solnechnogorsk district; locality: Chashnikovo; verbatimElevation: 220 m; minimumElevationInMeters: 220; decimalLatitude: 56.0375; decimalLongitude: 37.1874; **Identification:** identifiedBy: V.E. Pilipenko; **Event:** samplingProtocol: Sweep net; eventDate: 1995-08-07; verbatimEventDate: 07/Aug/1995; **Record Level:** institutionCode: VPMC; basisOfRecord: PreservedSpecimen
- **Type status:** Other material. **Occurrence:** occurrenceRemarks: 4 males; recordedBy: V.E. Pilipenko; individualCount: 4; sex: male; occurrenceID: EU_LIM_655; **Taxon:** scientificName: Neolimnomyiabatava (Edwards, 1938); family: Limoniidae; genus: Neolimnomyia; specificEpithet: batava; scientificNameAuthorship: (Edwards, 1938); **Location:** country: Russia; stateProvince: Central European Russia; county: Moskovskaya Oblast; municipality: Solnechnogorsk district; locality: Chashnikovo; verbatimElevation: 220 m; minimumElevationInMeters: 220; decimalLatitude: 56.0375; decimalLongitude: 37.1874; **Identification:** identifiedBy: V.E. Pilipenko; **Event:** samplingProtocol: Sweep net; eventDate: 1997-06-25; verbatimEventDate: 25/Jun/1997; **Record Level:** institutionCode: VPMC; basisOfRecord: PreservedSpecimen
- **Type status:** Other material. **Occurrence:** occurrenceRemarks: 1 male, 1 female; recordedBy: V.E. Pilipenko; individualCount: 2; sex: male, female; occurrenceID: EU_LIM_656; **Taxon:** scientificName: Neolimnomyiabatava (Edwards, 1938); family: Limoniidae; genus: Neolimnomyia; specificEpithet: batava; scientificNameAuthorship: (Edwards, 1938); **Location:** country: Russia; stateProvince: Central European Russia; county: Moskovskaya Oblast; municipality: Solnechnogorsk district; locality: Chashnikovo; verbatimElevation: 220 m; minimumElevationInMeters: 220; decimalLatitude: 56.0375; decimalLongitude: 37.1874; **Identification:** identifiedBy: V.E. Pilipenko; **Event:** samplingProtocol: Sweep net; eventDate: 1997-06-03; verbatimEventDate: 03/Jul/1997; **Record Level:** institutionCode: VPMC; basisOfRecord: PreservedSpecimen

#### Distribution

First records from Russia: RUC.

### Neolimnomyia filata

(Walker, 1856)

8C8BC428-BBC7-5029-9E7C-C9FF39E02881

https://ccw.naturalis.nl/detail.php?id=6904

#### Materials

- **Type status:** Other material. **Occurrence:** occurrenceRemarks: 6 males; recordedBy: V.E. Pilipenko; individualCount: 6; sex: male; occurrenceID: EU_LIM_657; **Taxon:** scientificName: Neolimnomyiafilata (Walker, 1856); family: Limoniidae; genus: Neolimnomyia; specificEpithet: filata; scientificNameAuthorship: (Walker, 1856); **Location:** country: Russia; stateProvince: Central European Russia; county: Moskovskaya Oblast; municipality: Solnechnogorsk district; locality: Chashnikovo; verbatimElevation: 220 m; minimumElevationInMeters: 220; decimalLatitude: 56.0375; decimalLongitude: 37.1874; **Identification:** identifiedBy: V.E. Pilipenko; **Event:** samplingProtocol: Sweep net; eventDate: 1997-06-25; verbatimEventDate: 25/Jun/1997; **Record Level:** institutionCode: VPMC; basisOfRecord: PreservedSpecimen
- **Type status:** Other material. **Occurrence:** occurrenceRemarks: 5 males; recordedBy: V.E. Pilipenko; individualCount: 5; sex: male; occurrenceID: EU_LIM_658; **Taxon:** scientificName: Neolimnomyiafilata (Walker, 1856); family: Limoniidae; genus: Neolimnomyia; specificEpithet: filata; scientificNameAuthorship: (Walker, 1856); **Location:** country: Russia; stateProvince: Central European Russia; county: Moskovskaya Oblast; municipality: Solnechnogorsk district; locality: Chashnikovo; verbatimElevation: 220 m; minimumElevationInMeters: 220; decimalLatitude: 56.0375; decimalLongitude: 37.1874; **Identification:** identifiedBy: V.E. Pilipenko; **Event:** samplingProtocol: Sweep net; eventDate: 1997-06-02; verbatimEventDate: 02/Jul/1997; **Record Level:** institutionCode: VPMC; basisOfRecord: PreservedSpecimen
- **Type status:** Other material. **Occurrence:** occurrenceRemarks: 3 males; recordedBy: V.E. Pilipenko; individualCount: 3; sex: male; occurrenceID: EU_LIM_659; **Taxon:** scientificName: Neolimnomyiafilata (Walker, 1856); family: Limoniidae; genus: Neolimnomyia; specificEpithet: filata; scientificNameAuthorship: (Walker, 1856); **Location:** country: Russia; stateProvince: Central European Russia; county: Moskovskaya Oblast; municipality: Solnechnogorsk district; locality: Chashnikovo; verbatimElevation: 220 m; minimumElevationInMeters: 220; decimalLatitude: 56.0375; decimalLongitude: 37.1874; **Identification:** identifiedBy: V.E. Pilipenko; **Event:** samplingProtocol: Sweep net; eventDate: 1997-06-03; verbatimEventDate: 03/Jul/1997; **Record Level:** institutionCode: VPMC; basisOfRecord: PreservedSpecimen

#### Distribution

First records from Russia: RUC.

### Neolimnophila alaskana

(Alexander, 1924)

C49F15C3-AE37-5AB1-B66F-6A70E7A218F9

https://ccw.naturalis.nl/detail.php?id=3496

#### Materials

- **Type status:** Other material. **Occurrence:** occurrenceRemarks: 1 female; recordedBy: P. Oosterbroek; individualCount: 1; sex: female; occurrenceID: EU_LIM_660; **Taxon:** scientificName: Neolimnophilaalaskana (Alexander, 1924); family: Limoniidae; genus: Neolimnophila; specificEpithet: alaskana; scientificNameAuthorship: (Alexander, 1924); **Location:** country: Netherlands; stateProvince: Friesland; municipality: Bakkeveen; locality: Duurswoude; decimalLatitude: 53.06; decimalLongitude: 6.22; **Identification:** identifiedBy: P. Oosterbroek; **Event:** eventDate: 1961-08-20; verbatimEventDate: 20/Aug/1961; **Record Level:** institutionCode: NBCN; basisOfRecord: PreservedSpecimen
- **Type status:** Other material. **Occurrence:** catalogNumber: 668198; occurrenceRemarks: 1 female; recordedBy: K.M. Olsen; individualCount: 1; sex: female; preparations: Ethanol; occurrenceID: EU_LIM_661; **Taxon:** scientificName: Neolimnophilaalaskana (Alexander, 1924); family: Limoniidae; genus: Neolimnophila; specificEpithet: alaskana; scientificNameAuthorship: (Alexander, 1924); **Location:** country: Norway; stateProvince: Buskerud; municipality: Hurum; locality: Verket; verbatimElevation: 10 m; minimumElevationInMeters: 10; decimalLatitude: 59.61556; decimalLongitude: 10.41904; **Identification:** identifiedBy: K.M. Olsen; **Event:** samplingProtocol: Malaise trap; eventDate: 2020-06-11/2020-07-31; verbatimEventDate: 11/Jun-31/Jul/2020; habitat: Vestre deler av sandtaket; **Record Level:** institutionCode: PCKMO; basisOfRecord: PreservedSpecimen
- **Type status:** Other material. **Occurrence:** catalogNumber: 638245, 644188; occurrenceRemarks: 2 males; recordedBy: K.M. Olsen | S. Reiso; individualCount: 2; sex: male; preparations: Ethanol; occurrenceID: EU_LIM_662; **Taxon:** scientificName: Neolimnophilaalaskana (Alexander, 1924); family: Limoniidae; genus: Neolimnophila; specificEpithet: alaskana; scientificNameAuthorship: (Alexander, 1924); **Location:** country: Norway; stateProvince: Telemark; municipality: Notodden; locality: Trettelinatten; verbatimElevation: 410 m; minimumElevationInMeters: 410; decimalLatitude: 59.54955; decimalLongitude: 9.38135; **Identification:** identifiedBy: K.M. Olsen; **Event:** samplingProtocol: Malaise trap; eventDate: 2019-06-12/2019-07-09; verbatimEventDate: 12/Jun-09/Jul/2019; **Record Level:** institutionCode: PCKMO; basisOfRecord: PreservedSpecimen

#### Distribution

First records from Norway and The Netherlands.

### Neolimnophila carteri

(Tonnoir, 1921)

171E6E6A-599D-54ED-9D68-3AB668604C58

https://ccw.naturalis.nl/detail.php?id=3482

#### Materials

- **Type status:** Other material. **Occurrence:** occurrenceRemarks: 1 female; recordedBy: V.E. Pilipenko; individualCount: 1; sex: female; occurrenceID: EU_LIM_663; **Taxon:** scientificName: Neolimnophilacarteri (Tonnoir, 1921); family: Limoniidae; genus: Neolimnophila; specificEpithet: carteri; scientificNameAuthorship: (Tonnoir, 1921); **Location:** country: Russia; stateProvince: Central European Russia; county: Moskovskaya Oblast; municipality: Ruza district; locality: Glubokoe Lake; verbatimElevation: 200 m; minimumElevationInMeters: 200; decimalLatitude: 55.7539; decimalLongitude: 36.50491; **Identification:** identifiedBy: V.E. Pilipenko; **Event:** samplingProtocol: Sweep net; eventDate: 1997-06-07; verbatimEventDate: 07/Jun/1997; **Record Level:** institutionCode: VPMC; basisOfRecord: PreservedSpecimen
- **Type status:** Other material. **Occurrence:** occurrenceRemarks: 1 male; recordedBy: V.E. Pilipenko; individualCount: 1; sex: male; occurrenceID: EU_LIM_664; **Taxon:** scientificName: Neolimnophilacarteri (Tonnoir, 1921); family: Limoniidae; genus: Neolimnophila; specificEpithet: carteri; scientificNameAuthorship: (Tonnoir, 1921); **Location:** country: Russia; stateProvince: Central European Russia; county: Moskovskaya Oblast; municipality: Solnechnogorsk district; locality: Chashnikovo; verbatimElevation: 220 m; minimumElevationInMeters: 220; decimalLatitude: 56.0375; decimalLongitude: 37.1874; **Identification:** identifiedBy: V.E. Pilipenko; **Event:** samplingProtocol: Sweep net; eventDate: 1992-06-04; verbatimEventDate: 04/Jun/1992; **Record Level:** institutionCode: VPMC; basisOfRecord: PreservedSpecimen

#### Distribution

First records from Russia: RUC.

### Neolimnophila placida

(Meigen, 1830)

3437E28B-FF8B-5803-A0F2-FE68E89F86AF

https://ccw.naturalis.nl/detail.php?id=3493

#### Materials

- **Type status:** Other material. **Occurrence:** occurrenceRemarks: 1 male; recordedBy: D.I. Gavryushin; individualCount: 1; sex: male; occurrenceID: EU_LIM_665; **Taxon:** scientificName: Neolimnophilaplacida (Meigen, 1830); family: Limoniidae; genus: Neolimnophila; specificEpithet: placida; scientificNameAuthorship: (Meigen, 1830); **Location:** country: Russia; stateProvince: East European Russia; county: Bashkortostan Respublika; municipality: Beloretsk district; locality: Nura River (ca. 4km W of Otnurok village), at the foot of Zolotyie Shishki (Golden Cones) Mts.; verbatimElevation: 607 m; minimumElevationInMeters: 607; decimalLatitude: 54.05155; decimalLongitude: 58.26887; **Identification:** identifiedBy: D.I. Gavryushin; **Event:** samplingProtocol: Sweep net; eventDate: 2012-08-09; verbatimEventDate: 09/Aug/2012; **Record Level:** institutionCode: ZMMU; basisOfRecord: PreservedSpecimen

#### Distribution

First records from Russia: RUE.

### Neolimonia dumetorum

(Meigen, 1804)

9F1C4636-BFCE-50AD-A145-1546725601E6

https://ccw.naturalis.nl/detail.php?id=10864

#### Materials

- **Type status:** Other material. **Occurrence:** occurrenceRemarks: 1 male; recordedBy: L.-P. Kolcsár | E. Török; individualCount: 1; sex: male; preparations: Ethanol; occurrenceID: EU_LIM_666; **Taxon:** scientificName: Neolimoniadumetorum (Meigen, 1804); family: Limoniidae; genus: Neolimonia; specificEpithet: dumetorum; scientificNameAuthorship: (Meigen, 1804); **Location:** country: Albania; stateProvince: Korçë; municipality: Buqezë; locality: Ohrid Lake; verbatimElevation: 696 m; minimumElevationInMeters: 696; decimalLatitude: 41.04104; decimalLongitude: 20.63448; **Identification:** identifiedBy: L.-P. Kolcsár; **Event:** samplingProtocol: Sweep net; eventDate: 2017-06-29; verbatimEventDate: 29/Jun/2017; **Record Level:** institutionCode: CKLP; basisOfRecord: PreservedSpecimen
- **Type status:** Other material. **Occurrence:** occurrenceRemarks: 1 male; recordedBy: J.W. Dienske; individualCount: 1; sex: male; preparations: Pinned; occurrenceID: EU_LIM_667; **Taxon:** scientificName: Neolimoniadumetorum (Meigen, 1804); family: Limoniidae; genus: Neolimonia; specificEpithet: dumetorum; scientificNameAuthorship: (Meigen, 1804); **Location:** island: Samothraki; country: Greece; stateProvince: Eastern Macedonia and Thrace; municipality: Therma; verbatimElevation: 0-100 m; maximumElevationInMeters: 100; decimalLatitude: 40.496; decimalLongitude: 25.607; **Identification:** identifiedBy: P. Oosterbroek; **Event:** eventDate: 1984-06-17; verbatimEventDate: 17/Jun/1984; habitat: along small stream; **Record Level:** institutionCode: NBCN; basisOfRecord: PreservedSpecimen
- **Type status:** Other material. **Occurrence:** occurrenceRemarks: 1 male; recordedBy: L.-P. Kolcsár; individualCount: 1; sex: male; preparations: Ethanol; occurrenceID: EU_LIM_668; **Taxon:** scientificName: Neolimoniadumetorum (Meigen, 1804); family: Limoniidae; genus: Neolimonia; specificEpithet: dumetorum; scientificNameAuthorship: (Meigen, 1804); **Location:** country: Latvia; municipality: Sigulda; locality: Gauja River; verbatimElevation: 13 m; minimumElevationInMeters: 13; decimalLatitude: 57.1505; decimalLongitude: 24.8168; **Identification:** identifiedBy: L.-P. Kolcsár; **Event:** samplingProtocol: Sweep net; eventDate: 2018-06-24; verbatimEventDate: 24/Jul/2018; **Record Level:** institutionCode: CKLP; basisOfRecord: PreservedSpecimen
- **Type status:** Other material. **Occurrence:** occurrenceRemarks: 2 males, 1 female; recordedBy: L.-P. Kolcsár | E. Török; individualCount: 3; sex: male, female; preparations: Ethanol; occurrenceID: EU_LIM_669; **Taxon:** scientificName: Neolimoniadumetorum (Meigen, 1804); family: Limoniidae; genus: Neolimonia; specificEpithet: dumetorum; scientificNameAuthorship: (Meigen, 1804); **Location:** country: North Macedonia; municipality: Novo Selo; locality: Bistra Mts., Marlovo NP.; verbatimElevation: 990 m; minimumElevationInMeters: 990; decimalLatitude: 41.71944; decimalLongitude: 20.82889; **Identification:** identifiedBy: L.-P. Kolcsár; **Event:** samplingProtocol: Sweep net; eventDate: 2017-06-29; verbatimEventDate: 29/Jun/2017; **Record Level:** institutionCode: CKLP; basisOfRecord: PreservedSpecimen
- **Type status:** Other material. **Occurrence:** occurrenceRemarks: 1 male, 1 female; recordedBy: L.-P. Kolcsár | E. Török; individualCount: 2; sex: male, female; preparations: Ethanol; occurrenceID: EU_LIM_670; **Taxon:** scientificName: Neolimoniadumetorum (Meigen, 1804); family: Limoniidae; genus: Neolimonia; specificEpithet: dumetorum; scientificNameAuthorship: (Meigen, 1804); **Location:** country: North Macedonia; municipality: Lipkovo; locality: Skopska Crna Gora Mts., Kumanovska River; verbatimElevation: 696 m; minimumElevationInMeters: 696; decimalLatitude: 42.17982; decimalLongitude: 21.56363; **Identification:** identifiedBy: L.-P. Kolcsár; **Event:** samplingProtocol: Sweep net; eventDate: 2017-06-30; verbatimEventDate: 30/Jun/2017; **Record Level:** institutionCode: CKLP; basisOfRecord: PreservedSpecimen

#### Distribution

First records from Albania, Greece (from Samothraki) and North Macedonia. Presence of the species in Latvia is confirmed.

### Orimarga (Orimarga) attenuata

(Walker, 1848)

C5E596D5-C343-5DEE-A2A7-81291C8B029C

https://ccw.naturalis.nl/detail.php?id=10932

#### Materials

- **Type status:** Other material. **Occurrence:** occurrenceRemarks: 1 male; recordedBy: M.C. d'Oliveira; individualCount: 1; sex: male; preparations: Ethanol; occurrenceID: EU_LIM_860; **Taxon:** scientificName: Orimarga (Orimarga) attenuata (Walker, 1848); family: Limoniidae; genus: Orimarga; subgenus: Orimarga; specificEpithet: attenuata; scientificNameAuthorship: (Walker, 1848); **Location:** country: Slovenia; municipality: Kranjska Gora; locality: Gozd Martuljek, 10 meters from the Sava river; verbatimElevation: 745 m; minimumElevationInMeters: 745; decimalLatitude: 46.483; decimalLongitude: 13.837539; **Identification:** identifiedBy: M.C. d'Oliveira; **Event:** samplingProtocol: Light trap; eventDate: 2019-20-8; verbatimEventDate: 20/August/2019; habitat: Small woodland next to river; **Record Level:** institutionCode: PCMCO; basisOfRecord: PreservedSpecimen

#### Distribution

First record from Slovenia.

### Orimarga (Orimarga) juvenilis

(Zetterstedt, 1851)

6BBF373B-7779-5B25-9C87-F6B2668AB564

https://ccw.naturalis.nl/detail.php?id=10977

#### Materials

- **Type status:** Other material. **Occurrence:** catalogNumber: 668197; occurrenceRemarks: 1 female; recordedBy: K.M. Olsen; individualCount: 1; sex: female; preparations: Ethanol; occurrenceID: EU_LIM_671; **Taxon:** scientificName: Orimarga (Orimarga) juvenilis (Zetterstedt, 1851); family: Limoniidae; genus: Orimarga; subgenus: Orimarga; specificEpithet: juvenilis; scientificNameAuthorship: (Zetterstedt, 1851); **Location:** country: Norway; stateProvince: Buskerud; municipality: Hurum; locality: Verket; verbatimElevation: 10 m; minimumElevationInMeters: 10; decimalLatitude: 59.61556; decimalLongitude: 10.41904; **Identification:** identifiedBy: K.M. Olsen; **Event:** samplingProtocol: Malaise trap; eventDate: 2020-06-11/2020-07-31; verbatimEventDate: 11/Jun-31/Jul/2020; habitat: Vestre deler av sandtaket; **Record Level:** institutionCode: ZMUB; basisOfRecord: PreservedSpecimen
- **Type status:** Other material. **Occurrence:** catalogNumber: 647845; occurrenceRemarks: 1 female; recordedBy: Rikmyrsprosjektet; individualCount: 1; sex: female; preparations: Ethanol; occurrenceID: EU_LIM_672; **Taxon:** scientificName: Orimarga (Orimarga) juvenilis (Zetterstedt, 1851); family: Limoniidae; genus: Orimarga; subgenus: Orimarga; specificEpithet: juvenilis; scientificNameAuthorship: (Zetterstedt, 1851); **Location:** country: Norway; stateProvince: Hedmark; municipality: Tolga; locality: N Bjørvollen; verbatimElevation: 770 m; minimumElevationInMeters: 770; decimalLatitude: 62.38703; decimalLongitude: 11.11887; **Identification:** identifiedBy: K.M. Olsen; **Event:** samplingProtocol: Malaise trap; eventDate: 2016-06-11/2016-07-21; verbatimEventDate: 11-21/Jul/2016; habitat: Malaise trap 7; **Record Level:** institutionCode: ZMUB; basisOfRecord: PreservedSpecimen
- **Type status:** Other material. **Occurrence:** catalogNumber: 648361; occurrenceRemarks: 1 male; recordedBy: K.M. Olsen; individualCount: 1; sex: male; preparations: Ethanol; occurrenceID: EU_LIM_673; **Taxon:** scientificName: Orimarga (Orimarga) juvenilis (Zetterstedt, 1851); family: Limoniidae; genus: Orimarga; subgenus: Orimarga; specificEpithet: juvenilis; scientificNameAuthorship: (Zetterstedt, 1851); **Location:** country: Norway; stateProvince: Nordland; municipality: Evenes; locality: Stunesosen; verbatimElevation: 1-2 m; minimumElevationInMeters: 1; maximumElevationInMeters: 2; decimalLatitude: 68.46567; decimalLongitude: 16.66601; **Identification:** identifiedBy: K.M. Olsen; **Event:** samplingProtocol: Sweep net; eventDate: 2019-06-02; verbatimEventDate: 02/Jul/2019; habitat: Mellom Tårstadveien og Kjerkvatnet; **Record Level:** institutionCode: NHMO; basisOfRecord: PreservedSpecimen
- **Type status:** Other material. **Occurrence:** catalogNumber: 648505; occurrenceRemarks: 1 female; recordedBy: K.M. Olsen; individualCount: 1; sex: female; preparations: Ethanol; occurrenceID: EU_LIM_674; **Taxon:** scientificName: Orimarga (Orimarga) juvenilis (Zetterstedt, 1851); family: Limoniidae; genus: Orimarga; subgenus: Orimarga; specificEpithet: juvenilis; scientificNameAuthorship: (Zetterstedt, 1851); **Location:** country: Norway; stateProvince: Nordland; municipality: Tjeldsund; locality: Osan – På begge sider av Tjeldøyveien; verbatimElevation: 1 m; minimumElevationInMeters: 1; decimalLatitude: 68.45083; decimalLongitude: 16.15136; **Identification:** identifiedBy: K.M. Olsen; **Event:** samplingProtocol: Sweep net; eventDate: 2019-06-02; verbatimEventDate: 02/Jul/2019; **Record Level:** institutionCode: NHMO; basisOfRecord: PreservedSpecimen
- **Type status:** Other material. **Occurrence:** catalogNumber: 560689, 644189; occurrenceRemarks: 12 male+female; recordedBy: K.M. Olsen; individualCount: 12; sex: male, female; preparations: Ethanol; occurrenceID: EU_LIM_675; **Taxon:** scientificName: Orimarga (Orimarga) juvenilis (Zetterstedt, 1851); family: Limoniidae; genus: Orimarga; subgenus: Orimarga; specificEpithet: juvenilis; scientificNameAuthorship: (Zetterstedt, 1851); **Location:** country: Norway; stateProvince: Nordland; municipality: Sørfold; locality: Nerigardsøyra; verbatimElevation: 0.5 m; minimumElevationInMeters: 0.5; decimalLatitude: 67.43321; decimalLongitude: 15.69589; **Identification:** identifiedBy: K.M. Olsen; **Event:** samplingProtocol: Sweep net; eventDate: 2017-06-18; verbatimEventDate: 18/Jul/2017; **Record Level:** institutionCode: PCKMO; basisOfRecord: PreservedSpecimen

#### Distribution

The species was deleted from the Norwegian species list by Olsen et al. (2018), as no confirmed records were known. We here confirm the presence of the species in Norway and report the first valid records.

### Orimarga (Orimarga) virgo

(Zetterstedt, 1851)

B1E2ACF6-791D-57EF-80CB-1B7D9CBCE20A

https://ccw.naturalis.nl/detail.php?id=11048

#### Materials

- **Type status:** Other material. **Occurrence:** catalogNumber: 647844; occurrenceRemarks: 1 female; recordedBy: Rikmyrsprosjektet; individualCount: 1; sex: female; preparations: Ethanol; occurrenceID: EU_LIM_676; **Taxon:** scientificName: Orimarga (Orimarga) virgo (Zetterstedt, 1851); family: Limoniidae; genus: Orimarga; subgenus: Orimarga; specificEpithet: virgo; scientificNameAuthorship: (Zetterstedt, 1851); **Location:** country: Norway; stateProvince: Hedmark; municipality: Tolga; locality: N Bjørvollen; verbatimElevation: 770 m; minimumElevationInMeters: 770; decimalLatitude: 62.38703; decimalLongitude: 11.11887; **Identification:** identifiedBy: K.M. Olsen; **Event:** samplingProtocol: Malaise trap; eventDate: 2016-06-11/2016-07-21; verbatimEventDate: 11-21/Jul/2016; habitat: Malaise trap 7; **Record Level:** institutionCode: ZMUB; basisOfRecord: PreservedSpecimen
- **Type status:** Other material. **Occurrence:** catalogNumber: 647880; occurrenceRemarks: 1 female; recordedBy: Rikmyrsprosjektet; individualCount: 1; sex: female; preparations: Ethanol; occurrenceID: EU_LIM_677; **Taxon:** scientificName: Orimarga (Orimarga) virgo (Zetterstedt, 1851); family: Limoniidae; genus: Orimarga; subgenus: Orimarga; specificEpithet: virgo; scientificNameAuthorship: (Zetterstedt, 1851); **Location:** country: Norway; stateProvince: Hedmark; municipality: Tynset; locality: Brydalskjølen; verbatimElevation: 780 m; minimumElevationInMeters: 780; decimalLatitude: 62.25545; decimalLongitude: 10.90725; **Identification:** identifiedBy: K.M. Olsen; **Event:** samplingProtocol: Malaise trap; eventDate: 2016-06-11/2016-07-21; verbatimEventDate: 11-21/Jul/2016; habitat: Malaise trap 8; **Record Level:** institutionCode: ZMUB; basisOfRecord: PreservedSpecimen
- **Type status:** Other material. **Occurrence:** catalogNumber: 560733; occurrenceRemarks: 1 female; recordedBy: K.M. Olsen; individualCount: 1; sex: female; preparations: Ethanol; occurrenceID: EU_LIM_678; **Taxon:** scientificName: Orimarga (Orimarga) virgo (Zetterstedt, 1851); family: Limoniidae; genus: Orimarga; subgenus: Orimarga; specificEpithet: virgo; scientificNameAuthorship: (Zetterstedt, 1851); **Location:** country: Norway; stateProvince: Nordland; municipality: Beiarn; locality: W Evjeosen; verbatimElevation: 0-10 m; maximumElevationInMeters: 10; decimalLatitude: 67.07969; decimalLongitude: 14.38531; **Identification:** identifiedBy: K.M. Olsen; **Event:** samplingProtocol: Sweep net; eventDate: 2017-06-18; verbatimEventDate: 18/Jul/2017; **Record Level:** institutionCode: PCKMO; basisOfRecord: PreservedSpecimen

#### Distribution

First records from Norway.

### Ormosia (Ormosia) aciculata

Edwards, 1921

31FA3A20-29E1-5211-B1B7-F9D41A6D425F

https://ccw.naturalis.nl/detail.php?id=3527

#### Materials

- **Type status:** Other material. **Occurrence:** catalogNumber: 544027; occurrenceRemarks: 1 male; recordedBy: K.M. Olsen; individualCount: 1; sex: male; preparations: Ethanol; occurrenceID: EU_LIM_679; **Taxon:** scientificName: Ormosia (Ormosia) aciculata Edwards, 1921; family: Limoniidae; genus: Ormosia; subgenus: Ormosia; specificEpithet: aciculata; scientificNameAuthorship: Edwards, 1921; **Location:** country: Norway; stateProvince: Nordland; municipality: Narvik; locality: Sjøbuneset; verbatimElevation: 0-10 m; maximumElevationInMeters: 10; decimalLatitude: 68.41521; decimalLongitude: 17.30553; **Identification:** identifiedBy: J. Starý; **Event:** samplingProtocol: Sweep net; eventDate: 2017-06-01; verbatimEventDate: 01/Jul/2017; **Record Level:** institutionCode: PCKMO; basisOfRecord: PreservedSpecimen
- **Type status:** Other material. **Occurrence:** catalogNumber: 553063; occurrenceRemarks: 1 male; recordedBy: K.M. Olsen; individualCount: 1; sex: male; preparations: Ethanol; occurrenceID: EU_LIM_680; **Taxon:** scientificName: Ormosia (Ormosia) aciculata Edwards, 1921; family: Limoniidae; genus: Ormosia; subgenus: Ormosia; specificEpithet: aciculata; scientificNameAuthorship: Edwards, 1921; **Location:** country: Norway; stateProvince: Troms; municipality: Storfjord; locality: Sommerlund – (Geassesadji) S veien; verbatimElevation: 5 m; minimumElevationInMeters: 5; decimalLatitude: 69.25775; decimalLongitude: 19.8773; **Identification:** identifiedBy: K.M. Olsen; **Event:** samplingProtocol: Sweep net; eventDate: 2017-06-11; verbatimEventDate: 11/Jul/2017; **Record Level:** institutionCode: NHMO; basisOfRecord: PreservedSpecimen
- **Type status:** Other material. **Occurrence:** occurrenceRemarks: 1 male; recordedBy: D.I. Gavryushin; individualCount: 1; sex: male; occurrenceID: EU_LIM_681; **Taxon:** scientificName: Ormosia (Ormosia) aciculata Edwards, 1921; family: Limoniidae; genus: Ormosia; subgenus: Ormosia; specificEpithet: aciculata; scientificNameAuthorship: Edwards, 1921; **Location:** country: Russia; stateProvince: East European Russia; county: Bashkortostan Respublika; municipality: Beloretsk district; locality: Nura River (ca. 4km W of Otnurok village), at the foot of Zolotyie Shishki (Golden Cones) Mts.; verbatimElevation: 607 m; minimumElevationInMeters: 607; decimalLatitude: 54.05155; decimalLongitude: 58.26887; **Identification:** identifiedBy: D.I. Gavryushin; **Event:** samplingProtocol: Sweep net; eventDate: 2015-06-10; verbatimEventDate: 10/Jul/2015; **Record Level:** institutionCode: ZMMU; basisOfRecord: PreservedSpecimen
- **Type status:** Other material. **Occurrence:** occurrenceRemarks: 13 males; recordedBy: N.M. Paramonov; individualCount: 13; sex: male; occurrenceID: EU_LIM_682; **Taxon:** scientificName: Ormosia (Ormosia) aciculata Edwards, 1921; family: Limoniidae; genus: Ormosia; subgenus: Ormosia; specificEpithet: aciculata; scientificNameAuthorship: Edwards, 1921; **Location:** country: Russia; stateProvince: East European Russia; county: Tatarstan Respublika; municipality: Verhneuslonsk district; locality: base “Zoostation”, 3,5 km NW Pustye Morkvashi env.; verbatimElevation: 80 m; minimumElevationInMeters: 80; decimalLatitude: 55.47005; decimalLongitude: 48.44092; **Identification:** identifiedBy: N.M. Paramonov; **Event:** samplingProtocol: Sweep net; eventDate: 2013-05-08/2013-05-09; verbatimEventDate: 8-9/May/2013; habitat: ravine, wetland; **Record Level:** institutionCode: ZIN; basisOfRecord: PreservedSpecimen

#### Distribution

First records from Norway and Russia: RUE.

### Ormosia (Ormosia) bihamata

Lackschewitz, 1935

39CF0C49-C5F8-5496-9F00-3CE637B63DD1

https://ccw.naturalis.nl/detail.php?id=3553

#### Materials

- **Type status:** Other material. **Occurrence:** occurrenceRemarks: 1 male; recordedBy: R. Vuillot; individualCount: 1; sex: male; preparations: Ethanol; occurrenceID: EU_LIM_683; **Taxon:** scientificName: Ormosia (Ormosia) bihamata Lackschewitz, 1935; family: Limoniidae; genus: Ormosia; subgenus: Ormosia; specificEpithet: bihamata; scientificNameAuthorship: Lackschewitz, 1935; **Location:** country: France; stateProvince: Auvergne-Rhône-Alpes; municipality: Le Bourg-d'Oisans; locality: Lake Lauvitel; verbatimElevation: 1510 m; minimumElevationInMeters: 1510; decimalLatitude: 44.9639; decimalLongitude: 6.06527; **Identification:** identifiedBy: C. Quindroit; **Event:** samplingProtocol: Sweep net; eventDate: 2019-06-29; verbatimEventDate: 29/Jul/2019; **Record Level:** institutionCode: PCCQ; basisOfRecord: PreservedSpecimen

#### Distribution

First record from France (from mainland).

### Ormosia (Ormosia) clavata

(Tonnoir, 1920)

A618043F-5464-509C-8380-9AA0C97B5F2B

https://ccw.naturalis.nl/detail.php?id=3564

#### Materials

- **Type status:** Other material. **Occurrence:** occurrenceRemarks: 8 males; recordedBy: D.I. Gavryushin; individualCount: 8; sex: male; occurrenceID: EU_LIM_684; **Taxon:** scientificName: Ormosia (Ormosia) clavata (Tonnoir, 1920); family: Limoniidae; genus: Ormosia; subgenus: Ormosia; specificEpithet: clavata; scientificNameAuthorship: (Tonnoir, 1920); **Location:** country: Russia; stateProvince: East European Russia; county: Bashkortostan Respublika; municipality: Beloretsk district; locality: Abzakovo env., Karan River; verbatimElevation: 533 m; minimumElevationInMeters: 533; decimalLatitude: 53.83717; decimalLongitude: 58.57878; **Identification:** identifiedBy: D.I. Gavryushin; **Event:** samplingProtocol: Sweep net; eventDate: 2015-06-19; verbatimEventDate: 19/Jul/2015; **Record Level:** institutionCode: ZMMU; basisOfRecord: PreservedSpecimen
- **Type status:** Other material. **Occurrence:** occurrenceRemarks: 2 males; recordedBy: D.I. Gavryushin; individualCount: 2; sex: male; occurrenceID: EU_LIM_685; **Taxon:** scientificName: Ormosia (Ormosia) clavata (Tonnoir, 1920); family: Limoniidae; genus: Ormosia; subgenus: Ormosia; specificEpithet: clavata; scientificNameAuthorship: (Tonnoir, 1920); **Location:** country: Russia; stateProvince: East European Russia; county: Bashkortostan Respublika; municipality: Beloretsk district; locality: Abzakovo env., Karan River; verbatimElevation: 533 m; minimumElevationInMeters: 533; decimalLatitude: 53.83717; decimalLongitude: 58.57878; **Identification:** identifiedBy: D.I. Gavryushin; **Event:** samplingProtocol: Sweep net; eventDate: 2015-06-17; verbatimEventDate: 17/Jul/2015; **Record Level:** institutionCode: ZMMU; basisOfRecord: PreservedSpecimen
- **Type status:** Other material. **Occurrence:** occurrenceRemarks: 1 male; recordedBy: D.I. Gavryushin; individualCount: 1; sex: male; occurrenceID: EU_LIM_686; **Taxon:** scientificName: Ormosia (Ormosia) clavata (Tonnoir, 1920); family: Limoniidae; genus: Ormosia; subgenus: Ormosia; specificEpithet: clavata; scientificNameAuthorship: (Tonnoir, 1920); **Location:** country: Russia; stateProvince: East European Russia; county: Bashkortostan Respublika; municipality: Uchaly district; locality: Ural-Tau St. env., upper reaches of Mindyak River; verbatimElevation: 765 m; minimumElevationInMeters: 765; decimalLatitude: 53.96525; decimalLongitude: 58.57888; **Identification:** identifiedBy: D.I. Gavryushin; **Event:** samplingProtocol: Sweep net; eventDate: 2015-06-09; verbatimEventDate: 09/Jul/2015; **Record Level:** institutionCode: ZMMU; basisOfRecord: PreservedSpecimen
- **Type status:** Other material. **Occurrence:** occurrenceRemarks: 4 males, 2 fameles; recordedBy: D.I. Gavryushin; individualCount: 6; sex: male, female; occurrenceID: EU_LIM_687; **Taxon:** scientificName: Ormosia (Ormosia) clavata (Tonnoir, 1920); family: Limoniidae; genus: Ormosia; subgenus: Ormosia; specificEpithet: clavata; scientificNameAuthorship: (Tonnoir, 1920); **Location:** country: Russia; stateProvince: East European Russia; county: Bashkortostan Respublika; municipality: Beloretsk district; locality: Nura River (ca. 4km W of Otnurok village), at the foot of Zolotyie Shishki (Golden Cones) Mts.; verbatimElevation: 607 m; minimumElevationInMeters: 607; decimalLatitude: 54.05155; decimalLongitude: 58.26887; **Identification:** identifiedBy: D.I. Gavryushin; **Event:** samplingProtocol: Sweep net; eventDate: 2012-08-08; verbatimEventDate: 08/Aug/2012; **Record Level:** institutionCode: ZMMU; basisOfRecord: PreservedSpecimen
- **Type status:** Other material. **Occurrence:** occurrenceRemarks: 1 male; recordedBy: D.I. Gavryushin; individualCount: 1; sex: male; occurrenceID: EU_LIM_688; **Taxon:** scientificName: Ormosia (Ormosia) clavata (Tonnoir, 1920); family: Limoniidae; genus: Ormosia; subgenus: Ormosia; specificEpithet: clavata; scientificNameAuthorship: (Tonnoir, 1920); **Location:** country: Russia; stateProvince: East European Russia; county: Bashkortostan Respublika; municipality: Beloretsk district; locality: Nura River (ca. 4km W of Otnurok village), at the foot of Zolotyie Shishki (Golden Cones) Mts.; verbatimElevation: 607 m; minimumElevationInMeters: 607; decimalLatitude: 54.05155; decimalLongitude: 58.26887; **Identification:** identifiedBy: D.I. Gavryushin; **Event:** samplingProtocol: Sweep net; eventDate: 2012-08-09; verbatimEventDate: 09/Aug/2012; **Record Level:** institutionCode: ZMMU; basisOfRecord: PreservedSpecimen
- **Type status:** Other material. **Occurrence:** occurrenceRemarks: 2 males, 1 female; recordedBy: D.I. Gavryushin; individualCount: 3; sex: male, female; occurrenceID: EU_LIM_689; **Taxon:** scientificName: Ormosia (Ormosia) clavata (Tonnoir, 1920); family: Limoniidae; genus: Ormosia; subgenus: Ormosia; specificEpithet: clavata; scientificNameAuthorship: (Tonnoir, 1920); **Location:** country: Russia; stateProvince: East European Russia; county: Bashkortostan Respublika; municipality: Beloretsk district; locality: Nura River (ca. 4km W of Otnurok village), at the foot of Zolotyie Shishki (Golden Cones) Mts.; verbatimElevation: 607 m; minimumElevationInMeters: 607; decimalLatitude: 54.05155; decimalLongitude: 58.26887; **Identification:** identifiedBy: D.I. Gavryushin; **Event:** samplingProtocol: Sweep net; eventDate: 2012-08-11; verbatimEventDate: 11/Aug/2012; **Record Level:** institutionCode: ZMMU; basisOfRecord: PreservedSpecimen
- **Type status:** Other material. **Occurrence:** occurrenceRemarks: 1 male; recordedBy: V.E. Pilipenko; individualCount: 1; sex: male; occurrenceID: EU_LIM_690; **Taxon:** scientificName: Ormosia (Ormosia) clavata (Tonnoir, 1920); family: Limoniidae; genus: Ormosia; subgenus: Ormosia; specificEpithet: clavata; scientificNameAuthorship: (Tonnoir, 1920); **Location:** country: Russia; stateProvince: Central European Russia; county: Moskovskaya Oblast; municipality: Solnechnogorsk district; locality: Chashnikovo; verbatimElevation: 220 m; minimumElevationInMeters: 220; decimalLatitude: 56.0375; decimalLongitude: 37.1874; **Identification:** identifiedBy: V.E. Pilipenko; **Event:** samplingProtocol: Sweep net; eventDate: 1997-08-19; verbatimEventDate: 19/Aug/1997; **Record Level:** institutionCode: VPMC; basisOfRecord: PreservedSpecimen

#### Distribution

First records from Russia: RUC, RUE.

### Ormosia (Ormosia) depilata

Edwards, 1938

445C0113-774B-598E-8178-34B03DB7D460

https://ccw.naturalis.nl/detail.php?id=3583

#### Materials

- **Type status:** Other material. **Occurrence:** occurrenceRemarks: 1 male; recordedBy: N.M. Paramonov; individualCount: 1; sex: male; occurrenceID: EU_LIM_691; **Taxon:** scientificName: Ormosia (Ormosia) depilata Edwards, 1938; family: Limoniidae; genus: Ormosia; subgenus: Ormosia; specificEpithet: depilata; scientificNameAuthorship: Edwards, 1938; **Location:** country: Russia; stateProvince: East European Russia; county: Tatarstan Respublika; municipality: Verhneuslonsk district; locality: base “Zoostation”, 3,5 km NW Pustye Morkvashi env.; verbatimElevation: 80 m; minimumElevationInMeters: 80; decimalLatitude: 55.47005; decimalLongitude: 48.44092; **Identification:** identifiedBy: N.M. Paramonov; **Event:** samplingProtocol: Sweep net; eventDate: 2013-05-08/2013-05-09; verbatimEventDate: 8-9/May/2013; habitat: ravine, wetland; **Record Level:** institutionCode: ZIN; basisOfRecord: PreservedSpecimen

#### Distribution

First record from Russia: RUE.

### Ormosia (Ormosia) hederae

(Curtis, 1835)

52E4B586-DB0F-5839-972F-63B1A50531F5

https://ccw.naturalis.nl/detail.php?id=3615

#### Materials

- **Type status:** Other material. **Occurrence:** catalogNumber: JES-20120260; occurrenceRemarks: 1 male; recordedBy: J. Salmela; individualCount: 1; sex: male; preparations: Ethanol; occurrenceID: EU_LIM_692; **Taxon:** scientificName: Ormosia (Ormosia) hederae (Curtis, 1835); family: Limoniidae; genus: Ormosia; subgenus: Ormosia; specificEpithet: hederae; scientificNameAuthorship: (Curtis, 1835); **Location:** country: Finland; stateProvince: Nylandia; municipality: Helsinki; locality: Fastholma; verbatimElevation: 25 m; minimumElevationInMeters: 25; decimalLatitude: 60.206; decimalLongitude: 25.022; **Identification:** identifiedBy: J. Salmela; **Event:** samplingProtocol: Malaise trap; eventDate: 2011-09-18; verbatimEventDate: 18/Sep/2011; **Record Level:** institutionCode: LMM; basisOfRecord: PreservedSpecimen

#### Distribution

The species reported from Finland by Salmela 2012b and Salmela and Petrašiūnas 2014, but without locality data. Here, we publish the collection data of that record.

### Ormosia (Ormosia) lineata

(Meigen, 1804)

FF5932CF-6537-5E67-8186-6760CC5E56F2

https://ccw.naturalis.nl/detail.php?id=3643

#### Materials

- **Type status:** Other material. **Occurrence:** occurrenceRemarks: 2 males; recordedBy: N.M. Paramonov; individualCount: 2; sex: male; occurrenceID: EU_LIM_693; **Taxon:** scientificName: Ormosia (Ormosia) lineata (Meigen, 1804); family: Limoniidae; genus: Ormosia; subgenus: Ormosia; specificEpithet: lineata; scientificNameAuthorship: (Meigen, 1804); **Location:** country: Russia; stateProvince: East European Russia; county: Tatarstan Respublika; municipality: Verhneuslonsk district; locality: base “Zoostation”, 3,5 km NW Pustye Morkvashi env.; verbatimElevation: 80 m; minimumElevationInMeters: 80; decimalLatitude: 55.47005; decimalLongitude: 48.44092; **Identification:** identifiedBy: N.M. Paramonov; **Event:** samplingProtocol: Sweep net; eventDate: 2013-05-08/2013-05-09; verbatimEventDate: 8-9/May/2013; habitat: ravine, wetland; **Record Level:** institutionCode: ZIN; basisOfRecord: PreservedSpecimen

#### Distribution

We confirm the presence of the species in Russia: RUE.

### Ormosia (Ormosia) microstyla

Savchenko, 1973

88F2B2EC-A537-50FA-A0F8-C073219C3214

https://ccw.naturalis.nl/detail.php?id=3654

#### Materials

- **Type status:** Other material. **Occurrence:** occurrenceRemarks: 1 male; recordedBy: L.-P. Kolcsár; individualCount: 1; sex: male; preparations: Ethanol; occurrenceID: EU_LIM_694; **Taxon:** scientificName: Ormosia (Ormosia) microstyla Savchenko, 1973; family: Limoniidae; genus: Ormosia; subgenus: Ormosia; specificEpithet: microstyla; scientificNameAuthorship: Savchenko, 1973; **Location:** country: Romania; stateProvince: Brașov; municipality: Azuga; locality: Baiul Mts., Limbășel Valley; verbatimElevation: 1250 m; minimumElevationInMeters: 1250; decimalLatitude: 45.4989; decimalLongitude: 25.6079; **Identification:** identifiedBy: L.-P. Kolcsár; **Event:** samplingProtocol: Sweep net; eventDate: 2017-05-28; verbatimEventDate: 28/May/2017; **Record Level:** institutionCode: CKLP; basisOfRecord: PreservedSpecimen
- **Type status:** Other material. **Occurrence:** occurrenceRemarks: 1 male; recordedBy: L.-P. Kolcsár; individualCount: 1; sex: male; preparations: Ethanol; occurrenceID: EU_LIM_695; **Taxon:** scientificName: Ormosia (Ormosia) microstyla Savchenko, 1973; family: Limoniidae; genus: Ormosia; subgenus: Ormosia; specificEpithet: microstyla; scientificNameAuthorship: Savchenko, 1973; **Location:** country: Romania; stateProvince: Harghita; municipality: Bălan; locality: Hăşmaş Mts., Piatra Singuratică; verbatimElevation: 1590 m; minimumElevationInMeters: 1590; decimalLatitude: 46.6767; decimalLongitude: 25.8329; **Identification:** identifiedBy: L.-P. Kolcsár; **Event:** samplingProtocol: Sweep net; eventDate: 2013-06-13; verbatimEventDate: 13/Jun/2013; **Record Level:** institutionCode: CKLP; basisOfRecord: PreservedSpecimen

#### Description

Fig. 11

#### Distribution

First records from Romania.

### Ormosia (Ormosia) nodulosa

(Macquart, 1826)

DD15C42A-2A78-5E22-9A7A-265A44FB2AAD

https://ccw.naturalis.nl/detail.php?id=3670

#### Materials

- **Type status:** Other material. **Occurrence:** occurrenceRemarks: 1 male; recordedBy: E. Viitanen; individualCount: 1; sex: male; preparations: ethanol; occurrenceID: EU_LIM_862; **Taxon:** scientificName: Ormosia (Ormosia) nodulosa (Macquart, 1826); family: Limoniidae; genus: Ormosia; subgenus: Ormosia; specificEpithet: nodulosa; scientificNameAuthorship: (Macquart, 1826); **Location:** country: Finland; county: Uusimaa; municipality: Nurmijärvi; locality: Kaanaan metsä; verbatimElevation: 60 m; minimumElevationInMeters: 60; decimalLatitude: 60.447074; decimalLongitude: 24.847741; **Identification:** identifiedBy: E. Viitanen; **Event:** samplingProtocol: sweep net; eventDate: 2021-06-21; verbatimEventDate: 21/Jun/2021; **Record Level:** institutionCode: LMM; basisOfRecord: PreservedSpecimen

#### Distribution

The species was deleted from the Finnish species list by Salmela (2012a) as no confirmed records were known. We here confirm the presence of the species in Finland and report the first valid records.

### Ormosia (Ormosia) pseudosimilis

(Lundström, 1912)

2329CE37-3282-5999-B738-E0D8B4C28C90

https://ccw.naturalis.nl/detail.php?id=3690

#### Materials

- **Type status:** Other material. **Occurrence:** occurrenceRemarks: 2 males; recordedBy: D.I. Gavryushin; individualCount: 2; sex: male; preparations: Pinned; occurrenceID: EU_LIM_696; **Taxon:** scientificName: Ormosia (Ormosia) pseudosimilis (Lundstrom, 1912); family: Limoniidae; genus: Ormosia; subgenus: Ormosia; specificEpithet: pseudosimilis; scientificNameAuthorship: (Lundstrom, 1912); **Location:** country: Belarus; stateProvince: Minsk; county: Barysaw; locality: Glivin; verbatimElevation: 161 m; minimumElevationInMeters: 161; decimalLatitude: 54.14902; decimalLongitude: 28.63648; **Identification:** identifiedBy: D.I. Gavryushin; **Event:** samplingProtocol: Sweep net; eventDate: 2013-06-06; verbatimEventDate: 6/Jul/2013; **Record Level:** institutionCode: ZMMU; basisOfRecord: PreservedSpecimen
- **Type status:** Other material. **Occurrence:** occurrenceRemarks: 1 male; recordedBy: D.I. Gavryushin; individualCount: 1; sex: male; preparations: Pinned; occurrenceID: EU_LIM_697; **Taxon:** scientificName: Ormosia (Ormosia) pseudosimilis (Lundstrom, 1912); family: Limoniidae; genus: Ormosia; subgenus: Ormosia; specificEpithet: pseudosimilis; scientificNameAuthorship: (Lundstrom, 1912); **Location:** country: Belarus; stateProvince: Minsk; county: Barysaw; locality: Vialikaje Stachava; verbatimElevation: 156 m; minimumElevationInMeters: 156; decimalLatitude: 54.26555; decimalLongitude: 28.38332; **Identification:** identifiedBy: D.I. Gavryushin; **Event:** samplingProtocol: Sweep net; eventDate: 2013-06-07; verbatimEventDate: 7/Jul/2013; **Record Level:** institutionCode: ZMMU; basisOfRecord: PreservedSpecimen

#### Distribution

First records from Belarus.

### Ormosia (Ormosia) ruficauda

(Zetterstedt, 1838)

15031785-B96B-51B7-A2BD-4B9A37FC07B8

https://ccw.naturalis.nl/detail.php?id=3699

#### Materials

- **Type status:** Other material. **Occurrence:** occurrenceRemarks: 1 male; recordedBy: D.I. Gavryushin; individualCount: 1; sex: male; preparations: Pinned; occurrenceID: EU_LIM_698; **Taxon:** scientificName: Ormosia (Ormosia) ruficauda (Zetterstedt, 1838); family: Limoniidae; genus: Ormosia; subgenus: Ormosia; specificEpithet: ruficauda; scientificNameAuthorship: (Zetterstedt, 1838); **Location:** country: Belarus; stateProvince: Minsk; county: Barysaw; locality: Barysaw; verbatimElevation: 155 m; minimumElevationInMeters: 155; decimalLatitude: 54.25542; decimalLongitude: 28.48092; **Identification:** identifiedBy: D.I. Gavryushin; **Event:** samplingProtocol: Sweep net; eventDate: 2013-06-05; verbatimEventDate: 5/Jul/2013; **Record Level:** institutionCode: ZMMU; basisOfRecord: PreservedSpecimen
- **Type status:** Other material. **Occurrence:** occurrenceRemarks: 1 male; recordedBy: D.I. Gavryushin; individualCount: 1; sex: male; occurrenceID: EU_LIM_699; **Taxon:** scientificName: Ormosia (Ormosia) ruficauda (Zetterstedt, 1838); family: Limoniidae; genus: Ormosia; subgenus: Ormosia; specificEpithet: ruficauda; scientificNameAuthorship: (Zetterstedt, 1838); **Location:** country: Russia; stateProvince: East European Russia; county: Bashkortostan Respublika; municipality: Uchaly district; locality: Ural-Tau St. env., upper reaches of Mindyak River; verbatimElevation: 765 m; minimumElevationInMeters: 765; decimalLatitude: 53.96525; decimalLongitude: 58.57888; **Identification:** identifiedBy: D.I. Gavryushin; **Event:** samplingProtocol: Sweep net; eventDate: 2015-06-09; verbatimEventDate: 09/Jul/2015; **Record Level:** institutionCode: ZMMU; basisOfRecord: PreservedSpecimen
- **Type status:** Other material. **Occurrence:** occurrenceRemarks: 1 male, 1 female; recordedBy: D.I. Gavryushin; individualCount: 2; sex: male, female; occurrenceID: EU_LIM_700; **Taxon:** scientificName: Ormosia (Ormosia) ruficauda (Zetterstedt, 1838); family: Limoniidae; genus: Ormosia; subgenus: Ormosia; specificEpithet: ruficauda; scientificNameAuthorship: (Zetterstedt, 1838); **Location:** country: Russia; stateProvince: East European Russia; county: Bashkortostan Respublika; municipality: Beloretsk district; locality: Nura River (ca. 4km W of Otnurok village), at the foot of Zolotyie Shishki (Golden Cones) Mts.; verbatimElevation: 607 m; minimumElevationInMeters: 607; decimalLatitude: 54.05155; decimalLongitude: 58.26887; **Identification:** identifiedBy: D.I. Gavryushin; **Event:** samplingProtocol: Sweep net; eventDate: 2012-08-08; verbatimEventDate: 08/Aug/2012; **Record Level:** institutionCode: ZMMU; basisOfRecord: PreservedSpecimen
- **Type status:** Other material. **Occurrence:** occurrenceRemarks: 3 males; recordedBy: D.I. Gavryushin; individualCount: 3; sex: male; occurrenceID: EU_LIM_701; **Taxon:** scientificName: Ormosia (Ormosia) ruficauda (Zetterstedt, 1838); family: Limoniidae; genus: Ormosia; subgenus: Ormosia; specificEpithet: ruficauda; scientificNameAuthorship: (Zetterstedt, 1838); **Location:** country: Russia; stateProvince: East European Russia; county: Bashkortostan Respublika; municipality: Beloretsk district; locality: Nura River (ca. 4km W of Otnurok village), at the foot of Zolotyie Shishki (Golden Cones) Mts.; verbatimElevation: 607 m; minimumElevationInMeters: 607; decimalLatitude: 54.05155; decimalLongitude: 58.26887; **Identification:** identifiedBy: D.I. Gavryushin; **Event:** samplingProtocol: Sweep net; eventDate: 2012-08-09; verbatimEventDate: 09/Aug/2012; **Record Level:** institutionCode: ZMMU; basisOfRecord: PreservedSpecimen
- **Type status:** Other material. **Occurrence:** occurrenceRemarks: 1 male; recordedBy: D.I. Gavryushin; individualCount: 1; sex: male; occurrenceID: EU_LIM_702; **Taxon:** scientificName: Ormosia (Ormosia) ruficauda (Zetterstedt, 1838); family: Limoniidae; genus: Ormosia; subgenus: Ormosia; specificEpithet: ruficauda; scientificNameAuthorship: (Zetterstedt, 1838); **Location:** country: Russia; stateProvince: East European Russia; county: Bashkortostan Respublika; municipality: Beloretsk district; locality: Nura River (ca. 4km W of Otnurok village), at the foot of Zolotyie Shishki (Golden Cones) Mts.; verbatimElevation: 607 m; minimumElevationInMeters: 607; decimalLatitude: 54.05155; decimalLongitude: 58.26887; **Identification:** identifiedBy: D.I. Gavryushin; **Event:** samplingProtocol: Sweep net; eventDate: 2015-06-16; verbatimEventDate: 16/Jul/2015; **Record Level:** institutionCode: ZMMU; basisOfRecord: PreservedSpecimen
- **Type status:** Other material. **Occurrence:** occurrenceRemarks: 3 males; recordedBy: D.I. Gavryushin; individualCount: 3; sex: male; occurrenceID: EU_LIM_703; **Taxon:** scientificName: Ormosia (Ormosia) ruficauda (Zetterstedt, 1838); family: Limoniidae; genus: Ormosia; subgenus: Ormosia; specificEpithet: ruficauda; scientificNameAuthorship: (Zetterstedt, 1838); **Location:** country: Russia; stateProvince: East European Russia; county: Bashkortostan Respublika; municipality: Beloretsk district; locality: Makhmutovo env., Belaya River; verbatimElevation: 550 m; minimumElevationInMeters: 550; decimalLatitude: 54.33012; decimalLongitude: 58.80735; **Identification:** identifiedBy: D.I. Gavryushin; **Event:** samplingProtocol: Sweep net; eventDate: 2015-06-15; verbatimEventDate: 15/Jul/2015; **Record Level:** institutionCode: ZMMU; basisOfRecord: PreservedSpecimen

#### Distribution

First records from Belarus and Russia: RUE.

### Ormosia (Ormosia) staegeriana

Alexander, 1953

B2743C38-CE55-57BB-AE44-E96ECC8E2C9E

https://ccw.naturalis.nl/detail.php?id=3709

#### Materials

- **Type status:** Other material. **Occurrence:** occurrenceRemarks: 1 male; recordedBy: D.I. Gavryushin; individualCount: 1; sex: male; occurrenceID: EU_LIM_704; **Taxon:** scientificName: Ormosia (Ormosia) staegeriana Alexander, 1953; family: Limoniidae; genus: Ormosia; subgenus: Ormosia; specificEpithet: staegeriana; scientificNameAuthorship: Alexander, 1953; **Location:** country: Russia; stateProvince: East European Russia; county: Bashkortostan Respublika; municipality: Uchaly district; locality: Ural-Tau St. env., upper reaches of Mindyak River; verbatimElevation: 765 m; minimumElevationInMeters: 765; decimalLatitude: 53.96525; decimalLongitude: 58.57888; **Identification:** identifiedBy: D.I. Gavryushin; **Event:** samplingProtocol: Sweep net; eventDate: 2015-06-09; verbatimEventDate: 09/Jul/2015; **Record Level:** institutionCode: ZMMU; basisOfRecord: PreservedSpecimen
- **Type status:** Other material. **Occurrence:** occurrenceRemarks: 1 male; recordedBy: D.I. Gavryushin; individualCount: 1; sex: male; occurrenceID: EU_LIM_705; **Taxon:** scientificName: Ormosia (Ormosia) staegeriana Alexander, 1953; family: Limoniidae; genus: Ormosia; subgenus: Ormosia; specificEpithet: staegeriana; scientificNameAuthorship: Alexander, 1953; **Location:** country: Russia; stateProvince: East European Russia; county: Bashkortostan Respublika; municipality: Beloretsk district; locality: Nura River (ca. 4km W of Otnurok village), at the foot of Zolotyie Shishki (Golden Cones) Mts.; verbatimElevation: 607 m; minimumElevationInMeters: 607; decimalLatitude: 54.05155; decimalLongitude: 58.26887; **Identification:** identifiedBy: D.I. Gavryushin; **Event:** samplingProtocol: Sweep net; eventDate: 2015-06-10; verbatimEventDate: 10/Jul/2015; **Record Level:** institutionCode: ZMMU; basisOfRecord: PreservedSpecimen
- **Type status:** Other material. **Occurrence:** occurrenceRemarks: 1 male; recordedBy: D.I. Gavryushin; individualCount: 1; sex: male; occurrenceID: EU_LIM_706; **Taxon:** scientificName: Ormosia (Ormosia) staegeriana Alexander, 1953; family: Limoniidae; genus: Ormosia; subgenus: Ormosia; specificEpithet: staegeriana; scientificNameAuthorship: Alexander, 1953; **Location:** country: Russia; stateProvince: East European Russia; county: Bashkortostan Respublika; municipality: Beloretsk district; locality: Nura River (ca. 4km W of Otnurok village), at the foot of Zolotyie Shishki (Golden Cones) Mts.; verbatimElevation: 607 m; minimumElevationInMeters: 607; decimalLatitude: 54.05155; decimalLongitude: 58.26887; **Identification:** identifiedBy: D.I. Gavryushin; **Event:** samplingProtocol: Sweep net; eventDate: 2015-06-13; verbatimEventDate: 13/Jul/2015; **Record Level:** institutionCode: ZMMU; basisOfRecord: PreservedSpecimen

#### Distribution

First records from Russia: RUE.

### Paradelphomyia czizekiana

Starý, 1971

D8532C61-4D3E-5676-A7EE-0887D0AA39FD

https://ccw.naturalis.nl/detail.php?id=6945

#### Materials

- **Type status:** Other material. **Occurrence:** occurrenceRemarks: 3 males; recordedBy: J. Starý; individualCount: 3; sex: male; preparations: Pinned; occurrenceID: EU_LIM_707; **Taxon:** scientificName: Paradelphomyiaczizekiana Starý, 1971; family: Limoniidae; genus: Paradelphomyia; specificEpithet: czizekiana; scientificNameAuthorship: Starý, 1971; **Location:** country: Greece; stateProvince: Peloponnese; municipality: Arachova; locality: 3 km E, Taygetos Mts.; verbatimElevation: 1450 m; minimumElevationInMeters: 1450; decimalLatitude: 37.03889; decimalLongitude: 22.25417; **Identification:** identifiedBy: J. Starý; **Event:** eventDate: 2017-10-10; verbatimEventDate: 10/Oct/2017; habitat: spring; **Record Level:** institutionCode: PCJS; basisOfRecord: PreservedSpecimen
- **Type status:** Other material. **Occurrence:** occurrenceRemarks: 3 males; recordedBy: J. Starý; individualCount: 3; sex: male; preparations: Pinned; occurrenceID: EU_LIM_708; **Taxon:** scientificName: Paradelphomyiaczizekiana Starý, 1971; family: Limoniidae; genus: Paradelphomyia; specificEpithet: czizekiana; scientificNameAuthorship: Starý, 1971; **Location:** country: Greece; stateProvince: Peloponnese; municipality: Agios Vassilos; locality: 1.6 km NE, Taygetos Mts.; verbatimElevation: 1075 m; minimumElevationInMeters: 1075; decimalLatitude: 37.08472; decimalLongitude: 22.27889; **Identification:** identifiedBy: J. Starý; **Event:** eventDate: 2017-10-10; verbatimEventDate: 10/Oct/2017; habitat: brook; **Record Level:** institutionCode: PCJS; basisOfRecord: PreservedSpecimen
- **Type status:** Other material. **Occurrence:** occurrenceRemarks: 5 males, 1 female; recordedBy: J. Starý; individualCount: 6; sex: male, female; preparations: Pinned; occurrenceID: EU_LIM_709; **Taxon:** scientificName: Paradelphomyiaczizekiana Starý, 1971; family: Limoniidae; genus: Paradelphomyia; specificEpithet: czizekiana; scientificNameAuthorship: Starý, 1971; **Location:** country: Greece; stateProvince: Peloponnese; municipality: Artemisia; locality: 1 km E, Taygetos Mts.; verbatimElevation: 655 m; minimumElevationInMeters: 655; decimalLatitude: 37.09639; decimalLongitude: 22.24083; **Identification:** identifiedBy: J. Starý; **Event:** eventDate: 2017-10-07; verbatimEventDate: 7/Oct/2017; habitat: brook; **Record Level:** institutionCode: PCJS; basisOfRecord: PreservedSpecimen
- **Type status:** Other material. **Occurrence:** occurrenceRemarks: 2 males; recordedBy: J. Starý; individualCount: 2; sex: male; preparations: Pinned; occurrenceID: EU_LIM_710; **Taxon:** scientificName: Paradelphomyiaczizekiana Starý, 1971; family: Limoniidae; genus: Paradelphomyia; specificEpithet: czizekiana; scientificNameAuthorship: Starý, 1971; **Location:** country: Greece; stateProvince: Peloponnese; municipality: Alagonia; locality: 2.4 km NW, Taygetos Mts.; verbatimElevation: 1335 m; minimumElevationInMeters: 1335; decimalLatitude: 37.11528; decimalLongitude: 22.26861; **Identification:** identifiedBy: J. Starý; **Event:** eventDate: 2017-10-07; verbatimEventDate: 7/Oct/2017; habitat: brook, spring; **Record Level:** institutionCode: PCJS; basisOfRecord: PreservedSpecimen
- **Type status:** Other material. **Occurrence:** occurrenceRemarks: 2 males; recordedBy: J. Starý; individualCount: 2; sex: male; preparations: Pinned; occurrenceID: EU_LIM_711; **Taxon:** scientificName: Paradelphomyiaczizekiana Starý, 1971; family: Limoniidae; genus: Paradelphomyia; specificEpithet: czizekiana; scientificNameAuthorship: Starý, 1971; **Location:** country: Greece; stateProvince: Peloponnese; municipality: Alagonia; locality: 2.4 km NW, Taygetos Mts.; verbatimElevation: 1335 m; minimumElevationInMeters: 1335; decimalLatitude: 37.11528; decimalLongitude: 22.26861; **Identification:** identifiedBy: J. Starý; **Event:** eventDate: 2017-10-09; verbatimEventDate: 9/Oct/2017; habitat: brook, spring; **Record Level:** institutionCode: PCJS; basisOfRecord: PreservedSpecimen
- **Type status:** Other material. **Occurrence:** occurrenceRemarks: 1 male; recordedBy: J. Starý; individualCount: 1; sex: male; preparations: Pinned; occurrenceID: EU_LIM_712; **Taxon:** scientificName: Paradelphomyiaczizekiana Starý, 1971; family: Limoniidae; genus: Paradelphomyia; specificEpithet: czizekiana; scientificNameAuthorship: Starý, 1971; **Location:** country: Greece; stateProvince: Peloponnese; municipality: Kiparissi; locality: 0.4 km E, marsh near Parapiros River; decimalLatitude: 38; decimalLongitude: 21.766; **Identification:** identifiedBy: J. Starý; **Event:** eventDate: 2015-05-27; verbatimEventDate: 27/May/2015; **Record Level:** institutionCode: PCJS; basisOfRecord: PreservedSpecimen
- **Type status:** Other material. **Occurrence:** occurrenceRemarks: 1 male; recordedBy: M. Vála; individualCount: 1; sex: male; preparations: Pinned; occurrenceID: EU_LIM_713; **Taxon:** scientificName: Paradelphomyiaczizekiana Starý, 1971; family: Limoniidae; genus: Paradelphomyia; specificEpithet: czizekiana; scientificNameAuthorship: Starý, 1971; **Location:** country: Greece; stateProvince: Peloponnese; municipality: Alagonia; locality: 2.4 km NW, Taygetos Mts.; verbatimElevation: 1335 m; minimumElevationInMeters: 1335; decimalLatitude: 37.11528; decimalLongitude: 22.26861; **Identification:** identifiedBy: J. Starý; **Event:** eventDate: 2017-10-09; verbatimEventDate: 9/Oct/2017; habitat: brook, spring; **Record Level:** institutionCode: PCJS; basisOfRecord: PreservedSpecimen
- **Type status:** Other material. **Occurrence:** occurrenceRemarks: 10 males; recordedBy: L.-P. Kolcsár | E. Török; individualCount: 10; sex: male; preparations: Ethanol; occurrenceID: EU_LIM_714; **Taxon:** scientificName: Paradelphomyiaczizekiana Starý, 1971; family: Limoniidae; genus: Paradelphomyia; specificEpithet: czizekiana; scientificNameAuthorship: Starý, 1971; **Location:** country: Hungary; stateProvince: Heves; municipality: Szilvásvárad; locality: Bükk Mts., Szalajka Valley; verbatimElevation: 461 m; minimumElevationInMeters: 461; decimalLatitude: 48.0844; decimalLongitude: 20.40616; **Identification:** identifiedBy: J. Starý; **Event:** eventDate: 2013-08-16; verbatimEventDate: 16/Aug/2013; **Record Level:** institutionCode: PCJS; basisOfRecord: PreservedSpecimen

#### Distribution

First records from Greece (from mainland) and Hungary.

### Paradelphomyia ecalcarata

(Edwards, 1938)

9BC2377B-A8DF-58E8-8EAA-A639D721845F

https://ccw.naturalis.nl/detail.php?id=6953

#### Materials

- **Type status:** Other material. **Occurrence:** occurrenceRemarks: 1 male; recordedBy: L.-P. Kolcsár; individualCount: 1; sex: male; preparations: Ethanol; occurrenceID: EU_LIM_715; **Taxon:** scientificName: Paradelphomyiaecalcarata (Edwards, 1938); family: Limoniidae; genus: Paradelphomyia; specificEpithet: ecalcarata; scientificNameAuthorship: (Edwards, 1938); **Location:** country: Romania; stateProvince: Bihor; municipality: Pietroasa - Padiș; locality: Bihor Mts., Boga Valley; verbatimElevation: 900 m; minimumElevationInMeters: 900; decimalLatitude: 46.60897; decimalLongitude: 22.68597; **Identification:** identifiedBy: J. Starý; **Event:** samplingProtocol: Sweep net; eventDate: 2011-06-29; verbatimEventDate: 29/Jun/2011; **Record Level:** institutionCode: PCJS; basisOfRecord: PreservedSpecimen
- **Type status:** Other material. **Occurrence:** occurrenceRemarks: 1 male; recordedBy: L.-P. Kolcsár; individualCount: 1; sex: male; preparations: Ethanol; occurrenceID: EU_LIM_716; **Taxon:** scientificName: Paradelphomyiaecalcarata (Edwards, 1938); family: Limoniidae; genus: Paradelphomyia; specificEpithet: ecalcarata; scientificNameAuthorship: (Edwards, 1938); **Location:** country: Romania; stateProvince: Cluj; municipality: Feleac; locality: Feleacu Hills, Morii Valley; verbatimElevation: 620 m; minimumElevationInMeters: 620; decimalLatitude: 46.6958; decimalLongitude: 23.5912; **Identification:** identifiedBy: L.-P. Kolcsár; **Event:** samplingProtocol: Sweep net; eventDate: 2014-06-02; verbatimEventDate: 02/Jul/2014; **Record Level:** institutionCode: CKLP; basisOfRecord: PreservedSpecimen

#### Distribution

First records from Romania.

### Paradelphomyia fuscula

(Loew, 1873)

7E84B56B-3452-5CDB-B343-F62E77941FBD

https://ccw.naturalis.nl/detail.php?id=6958

#### Materials

- **Type status:** Other material. **Occurrence:** occurrenceRemarks: 1 male; recordedBy: L. Papp; individualCount: 1; sex: male; preparations: Pinned; occurrenceID: EU_LIM_717; **Taxon:** scientificName: Paradelphomyiafuscula (Loew, 1873); family: Limoniidae; genus: Paradelphomyia; specificEpithet: fuscula; scientificNameAuthorship: (Loew, 1873); **Location:** country: Hungary; stateProvince: Heves; municipality: Parád; locality: Mátrai Landscape Park, Vár-bükk; decimalLatitude: 47.89836; decimalLongitude: 20.06921; **Identification:** identifiedBy: L.-P. Kolcsár; **Event:** eventDate: 2007-06-03; verbatimEventDate: 03/Jul/2007; **Record Level:** institutionCode: HNHM; basisOfRecord: PreservedSpecimen
- **Type status:** Other material. **Occurrence:** occurrenceRemarks: 1 male; recordedBy: L. Papp; individualCount: 1; sex: male; preparations: Pinned; occurrenceID: EU_LIM_718; **Taxon:** scientificName: Paradelphomyiafuscula (Loew, 1873); family: Limoniidae; genus: Paradelphomyia; specificEpithet: fuscula; scientificNameAuthorship: (Loew, 1873); **Location:** country: Hungary; stateProvince: Heves; municipality: Szilvásvárad; locality: Szalajka Valley; decimalLatitude: 48.08025; decimalLongitude: 20.40587; **Identification:** identifiedBy: L.-P. Kolcsár; **Event:** eventDate: 2002-09-07; verbatimEventDate: 07/Sep/2002; **Record Level:** institutionCode: HNHM; basisOfRecord: PreservedSpecimen
- **Type status:** Other material. **Occurrence:** occurrenceRemarks: 2 males; recordedBy: L. Papp; individualCount: 2; sex: male; preparations: Pinned; occurrenceID: EU_LIM_719; **Taxon:** scientificName: Paradelphomyiafuscula (Loew, 1873); family: Limoniidae; genus: Paradelphomyia; specificEpithet: fuscula; scientificNameAuthorship: (Loew, 1873); **Location:** country: Hungary; stateProvince: Pest; municipality: Verőce; locality: Magyarkút, Keskenybükki Valley; decimalLatitude: 47.85405; decimalLongitude: 19.05528; **Identification:** identifiedBy: L.-P. Kolcsár; **Event:** eventDate: 1993-09-25; verbatimEventDate: 25/Sep/1993; habitat: oak forest; **Record Level:** institutionCode: HNHM; basisOfRecord: PreservedSpecimen
- **Type status:** Other material. **Occurrence:** occurrenceRemarks: 2 males; recordedBy: V.E. Pilipenko; individualCount: 2; sex: male; occurrenceID: EU_LIM_720; **Taxon:** scientificName: Paradelphomyiafuscula (Loew, 1873); family: Limoniidae; genus: Paradelphomyia; specificEpithet: fuscula; scientificNameAuthorship: (Loew, 1873); **Location:** country: Russia; stateProvince: Central European Russia; county: Moskovskaya Oblast; municipality: Solnechnogorsk district; locality: Chashnikovo; verbatimElevation: 220 m; minimumElevationInMeters: 220; decimalLatitude: 56.0375; decimalLongitude: 37.1874; **Identification:** identifiedBy: V.E. Pilipenko; **Event:** samplingProtocol: Sweep net; eventDate: 1994-08-16; verbatimEventDate: 16/Aug/1994; **Record Level:** institutionCode: VPMC; basisOfRecord: PreservedSpecimen
- **Type status:** Other material. **Occurrence:** occurrenceRemarks: 1 male; recordedBy: V.E. Pilipenko; individualCount: 1; sex: male; occurrenceID: EU_LIM_721; **Taxon:** scientificName: Paradelphomyiafuscula (Loew, 1873); family: Limoniidae; genus: Paradelphomyia; specificEpithet: fuscula; scientificNameAuthorship: (Loew, 1873); **Location:** country: Russia; stateProvince: Central European Russia; county: Moskovskaya Oblast; municipality: Solnechnogorsk district; locality: Chashnikovo; verbatimElevation: 220 m; minimumElevationInMeters: 220; decimalLatitude: 56.0375; decimalLongitude: 37.1874; **Identification:** identifiedBy: V.E. Pilipenko; **Event:** samplingProtocol: Sweep net; eventDate: 1997-08-21; verbatimEventDate: 21/Aug/1997; **Record Level:** institutionCode: VPMC; basisOfRecord: PreservedSpecimen
- **Type status:** Other material. **Occurrence:** occurrenceRemarks: 8 males, 11 females; recordedBy: N.M. Paramonov; individualCount: 19; sex: male, female; occurrenceID: EU_LIM_722; **Taxon:** scientificName: Paradelphomyiafuscula (Loew, 1873); family: Limoniidae; genus: Paradelphomyia; specificEpithet: fuscula; scientificNameAuthorship: (Loew, 1873); **Location:** country: Russia; stateProvince: East European Russia; county: Tatarstan Respublika; municipality: Verhneuslonsk district; locality: base “Zoostation”, 3,5 km NW Pustye Morkvashi env.; verbatimElevation: 80 m; minimumElevationInMeters: 80; decimalLatitude: 55.47005; decimalLongitude: 48.44092; **Identification:** identifiedBy: N.M. Paramonov; **Event:** samplingProtocol: Sweep net; eventDate: 2013-08-22/2013-08-26; verbatimEventDate: 22-26/Aug/2013; habitat: ravine, wetland; **Record Level:** institutionCode: ZIN; basisOfRecord: PreservedSpecimen

#### Distribution

First records from Hungary and Russia: RUC, RUE.

### Paradelphomyia nielseni

(Kuntze, 1919)

F4833846-ED68-5E04-B2E9-66B0CAB5A455

https://ccw.naturalis.nl/detail.php?id=6984

#### Materials

- **Type status:** Other material. **Occurrence:** occurrenceRemarks: 1 male; recordedBy: E. Eiroa; individualCount: 1; sex: male; preparations: Pinned; occurrenceID: EU_LIM_723; **Taxon:** scientificName: Paradelphomyianielseni (Kuntze, 1919); family: Limoniidae; genus: Paradelphomyia; specificEpithet: nielseni; scientificNameAuthorship: (Kuntze, 1919); **Location:** country: Spain; stateProvince: Cantabria; municipality: Hermandad de Campoo de Suso; locality: Riańo; verbatimElevation: 1185 m; minimumElevationInMeters: 1185; decimalLatitude: 43.01172; decimalLongitude: -4.29806; **Identification:** identifiedBy: E. Eiroa; **Event:** samplingProtocol: Sweep net; eventDate: 1995-10-24; verbatimEventDate: 24/Oct/1995; habitat: beech forest; **Record Level:** institutionCode: USC; basisOfRecord: PreservedSpecimen

#### Distribution

First record from Spain (from mainland).

### Paradelphomyia senilis

(Haliday, 1833)

B7A88730-868E-50F7-915E-8D28D75C07B8

https://ccw.naturalis.nl/detail.php?id=7005

#### Materials

- **Type status:** Other material. **Occurrence:** occurrenceRemarks: 1 male, 1 female; recordedBy: V.E. Pilipenko; individualCount: 2; sex: male, female; occurrenceID: EU_LIM_724; **Taxon:** scientificName: Paradelphomyiasenilis (Haliday, 1833); family: Limoniidae; genus: Paradelphomyia; specificEpithet: senilis; scientificNameAuthorship: (Haliday, 1833); **Location:** country: Russia; stateProvince: Central European Russia; county: Moskovskaya Oblast; municipality: Solnechnogorsk district; locality: Chashnikovo; verbatimElevation: 220 m; minimumElevationInMeters: 220; decimalLatitude: 56.0375; decimalLongitude: 37.1874; **Identification:** identifiedBy: V.E. Pilipenko; **Event:** samplingProtocol: Sweep net; eventDate: 1997-06-02; verbatimEventDate: 02/Jul/1997; **Record Level:** institutionCode: VPMC; basisOfRecord: PreservedSpecimen

#### Distribution

First record from Russia: RUC.

### Phylidorea (Macrolabina) alexanderi

(Starý, 1974)

552EC7E7-E775-5C9C-A591-E02870622943

https://ccw.naturalis.nl/detail.php?id=7124

#### Materials

- **Type status:** Other material. **Occurrence:** occurrenceRemarks: 1 male; recordedBy: L.-P. Kolcsár; individualCount: 1; sex: male; preparations: Ethanol; occurrenceID: EU_LIM_725; **Taxon:** scientificName: Phylidorea (Macrolabina) alexanderi (Starý, 1974); family: Limoniidae; genus: Phylidorea; subgenus: Macrolabina; specificEpithet: alexanderi; scientificNameAuthorship: (Starý, 1974); **Location:** country: Romania; stateProvince: Caraș-Severin; municipality: Brebu Nou; locality: Semenic Mts.; verbatimElevation: 873 m; minimumElevationInMeters: 873; decimalLatitude: 45.2188; decimalLongitude: 22.1073; **Identification:** identifiedBy: L.-P. Kolcsár; **Event:** samplingProtocol: Sweep net; eventDate: 2017-05-24; verbatimEventDate: 24/May/2017; habitat: spring; **Record Level:** institutionCode: CKLP; basisOfRecord: PreservedSpecimen
- **Type status:** Other material. **Occurrence:** occurrenceRemarks: 3 males; recordedBy: D.I. Gavryushin; individualCount: 3; sex: male; preparations: Pinned; occurrenceID: EU_LIM_726; **Taxon:** scientificName: Phylidorea (Macrolabina) alexanderi (Starý, 1974); family: Limoniidae; genus: Phylidorea; subgenus: Macrolabina; specificEpithet: alexanderi; scientificNameAuthorship: (Starý, 1974); **Location:** country: Serbia; locality: Stara Planina Mts.; verbatimElevation: 1500 m; minimumElevationInMeters: 1500; decimalLatitude: 43.37; decimalLongitude: 22.6; **Identification:** identifiedBy: D.I. Gavryushin; **Event:** samplingProtocol: Sweep net; eventDate: 2015-06-01/2015-07-07; verbatimEventDate: 01-07/Jul/2015; **Record Level:** institutionCode: ZMMU; basisOfRecord: PreservedSpecimen
- **Type status:** Other material. **Occurrence:** occurrenceRemarks: 4 males; recordedBy: L.-P. Kolcsár | E. Török; individualCount: 4; sex: male; preparations: Ethanol; occurrenceID: EU_LIM_727; **Taxon:** scientificName: Phylidorea (Macrolabina) alexanderi (Starý, 1974); family: Limoniidae; genus: Phylidorea; subgenus: Macrolabina; specificEpithet: alexanderi; scientificNameAuthorship: (Starý, 1974); **Location:** country: Serbia; municipality: Kopaonik; locality: Kopaonik Mts.; verbatimElevation: 1556 m; minimumElevationInMeters: 1556; decimalLatitude: 43.30997; decimalLongitude: 20.76563; **Identification:** identifiedBy: L.-P. Kolcsár; **Event:** samplingProtocol: Sweep net; eventDate: 2017-06-22; verbatimEventDate: 22/Jun/2017; **Record Level:** institutionCode: CKLP; basisOfRecord: PreservedSpecimen

#### Description

Figs 12, 13

#### Distribution

First records from Romania and Serbia.

### Phylidorea (Phylidorea) ferruginea

(Meigen, 1818)

81EDD384-9113-5B9D-8F46-AE09E12C4E33

https://ccw.naturalis.nl/detail.php?id=7145

#### Materials

- **Type status:** Other material. **Occurrence:** occurrenceRemarks: 1 male; recordedBy: D.I. Gavryushin; individualCount: 1; sex: male; occurrenceID: EU_LIM_734; **Taxon:** scientificName: Phylidorea (Phylidorea) ferruginea (Meigen, 1818); family: Limoniidae; genus: Phylidorea; subgenus: Phylidorea; specificEpithet: ferruginea; scientificNameAuthorship: (Meigen, 1818); **Location:** country: Russia; stateProvince: East European Russia; county: Bashkortostan Respublika; municipality: Beloretsk district; locality: Makhmutovo env., Belaya River; verbatimElevation: 550 m; minimumElevationInMeters: 550; decimalLatitude: 54.33012; decimalLongitude: 58.80735; **Identification:** identifiedBy: D.I. Gavryushin; **Event:** samplingProtocol: Sweep net; eventDate: 2015-06-15; verbatimEventDate: 15/Jul/2015; **Record Level:** institutionCode: ZMMU; basisOfRecord: PreservedSpecimen
- **Type status:** Other material. **Occurrence:** occurrenceRemarks: 1 male; recordedBy: D.I. Gavryushin; individualCount: 1; sex: male; preparations: Pinned; occurrenceID: EU_LIM_735; **Taxon:** scientificName: Phylidorea (Phylidorea) ferruginea (Meigen, 1818); family: Limoniidae; genus: Phylidorea; subgenus: Phylidorea; specificEpithet: ferruginea; scientificNameAuthorship: (Meigen, 1818); **Location:** country: Serbia; locality: Stara Planina Mts.; verbatimElevation: 1500 m; minimumElevationInMeters: 1500; decimalLatitude: 43.37; decimalLongitude: 22.6; **Identification:** identifiedBy: D.I. Gavryushin; **Event:** samplingProtocol: Sweep net; eventDate: 2014-09-16/2014-09-18; verbatimEventDate: 16-18/Sep/2014; **Record Level:** institutionCode: ZMMU; basisOfRecord: PreservedSpecimen

#### Distribution

First records from Russia: RUE and Serbia.

### Phylidorea (Paraphylidorea) fulvonervosa

(Schummel, 1829)

CA45CA0B-3310-560E-A3D5-E4C499ED0CD8

https://ccw.naturalis.nl/detail.php?id=7133

#### Materials

- **Type status:** Other material. **Occurrence:** occurrenceRemarks: 1 female; recordedBy: D.I. Gavryushin; individualCount: 1; sex: female; preparations: Pinned; occurrenceID: EU_LIM_728; **Taxon:** scientificName: Phylidorea (Paraphylidorea) fulvonervosa (Schummel, 1829); family: Limoniidae; genus: Phylidorea; subgenus: Paraphylidorea; specificEpithet: fulvonervosa; scientificNameAuthorship: (Schummel, 1829); **Location:** country: Belarus; stateProvince: Minsk; county: Barysaw; locality: Glivin; verbatimElevation: 161 m; minimumElevationInMeters: 161; decimalLatitude: 54.14902; decimalLongitude: 28.63648; **Identification:** identifiedBy: D.I. Gavryushin; **Event:** samplingProtocol: Sweep net; eventDate: 2013-06-06; verbatimEventDate: 6/Jul/2013; **Record Level:** institutionCode: ZMMU; basisOfRecord: PreservedSpecimen
- **Type status:** Other material. **Occurrence:** occurrenceRemarks: 1 male; recordedBy: D.I. Gavryushin; individualCount: 1; sex: male; occurrenceID: EU_LIM_729; **Taxon:** scientificName: Phylidorea (Paraphylidorea) fulvonervosa (Schummel, 1829); family: Limoniidae; genus: Phylidorea; subgenus: Paraphylidorea; specificEpithet: fulvonervosa; scientificNameAuthorship: (Schummel, 1829); **Location:** country: Russia; stateProvince: East European Russia; county: Bashkortostan Respublika; municipality: Beloretsk district; locality: Uzyan env., Malyi Kuhtur River; verbatimElevation: 499 m; minimumElevationInMeters: 499; decimalLatitude: 53.68733; decimalLongitude: 57.78358; **Identification:** identifiedBy: D.I. Gavryushin; **Event:** samplingProtocol: Sweep net; eventDate: 2015-06-11; verbatimEventDate: 11/Jul/2015; **Record Level:** institutionCode: ZMMU; basisOfRecord: PreservedSpecimen
- **Type status:** Other material. **Occurrence:** occurrenceRemarks: 1 male, 1 female; recordedBy: D.I. Gavryushin; individualCount: 2; sex: male, female; occurrenceID: EU_LIM_730; **Taxon:** scientificName: Phylidorea (Paraphylidorea) fulvonervosa (Schummel, 1829); family: Limoniidae; genus: Phylidorea; subgenus: Paraphylidorea; specificEpithet: fulvonervosa; scientificNameAuthorship: (Schummel, 1829); **Location:** country: Russia; stateProvince: East European Russia; county: Bashkortostan Respublika; municipality: Uchaly district; locality: Ural-Tau St. env., upper reaches of Mindyak River; verbatimElevation: 765 m; minimumElevationInMeters: 765; decimalLatitude: 53.96525; decimalLongitude: 58.57888; **Identification:** identifiedBy: D.I. Gavryushin; **Event:** samplingProtocol: Sweep net; eventDate: 2015-06-09; verbatimEventDate: 09/Jul/2015; **Record Level:** institutionCode: ZMMU; basisOfRecord: PreservedSpecimen
- **Type status:** Other material. **Occurrence:** occurrenceRemarks: 2 males, 1 female; recordedBy: D.I. Gavryushin; individualCount: 3; sex: male, female; occurrenceID: EU_LIM_731; **Taxon:** scientificName: Phylidorea (Paraphylidorea) fulvonervosa (Schummel, 1829); family: Limoniidae; genus: Phylidorea; subgenus: Paraphylidorea; specificEpithet: fulvonervosa; scientificNameAuthorship: (Schummel, 1829); **Location:** country: Russia; stateProvince: East European Russia; county: Bashkortostan Respublika; municipality: Beloretsk district; locality: Nura River (ca. 4km W of Otnurok village), at the foot of Zolotyie Shishki (Golden Cones) Mts.; verbatimElevation: 607 m; minimumElevationInMeters: 607; decimalLatitude: 54.05155; decimalLongitude: 58.26887; **Identification:** identifiedBy: D.I. Gavryushin; **Event:** samplingProtocol: Sweep net; eventDate: 2015-06-10; verbatimEventDate: 10/Jul/2015; **Record Level:** institutionCode: ZMMU; basisOfRecord: PreservedSpecimen
- **Type status:** Other material. **Occurrence:** occurrenceRemarks: 4 males; recordedBy: D.I. Gavryushin; individualCount: 4; sex: male; occurrenceID: EU_LIM_732; **Taxon:** scientificName: Phylidorea (Paraphylidorea) fulvonervosa (Schummel, 1829); family: Limoniidae; genus: Phylidorea; subgenus: Paraphylidorea; specificEpithet: fulvonervosa; scientificNameAuthorship: (Schummel, 1829); **Location:** country: Russia; stateProvince: East European Russia; county: Bashkortostan Respublika; municipality: Beloretsk district; locality: Nura River (ca. 4km W of Otnurok village), at the foot of Zolotyie Shishki (Golden Cones) Mts.; verbatimElevation: 607 m; minimumElevationInMeters: 607; decimalLatitude: 54.05155; decimalLongitude: 58.26887; **Identification:** identifiedBy: D.I. Gavryushin; **Event:** samplingProtocol: Sweep net; eventDate: 2015-06-13; verbatimEventDate: 13/Jul/2015; **Record Level:** institutionCode: ZMMU; basisOfRecord: PreservedSpecimen
- **Type status:** Other material. **Occurrence:** occurrenceRemarks: 1 male; recordedBy: D.I. Gavryushin; individualCount: 1; sex: male; occurrenceID: EU_LIM_733; **Taxon:** scientificName: Phylidorea (Paraphylidorea) fulvonervosa (Schummel, 1829); family: Limoniidae; genus: Phylidorea; subgenus: Paraphylidorea; specificEpithet: fulvonervosa; scientificNameAuthorship: (Schummel, 1829); **Location:** country: Russia; stateProvince: East European Russia; county: Bashkortostan Respublika; municipality: Beloretsk district; locality: Nura River (ca. 4km W of Otnurok village), at the foot of Zolotyie Shishki (Golden Cones) Mts.; verbatimElevation: 607 m; minimumElevationInMeters: 607; decimalLatitude: 54.05155; decimalLongitude: 58.26887; **Identification:** identifiedBy: D.I. Gavryushin; **Event:** samplingProtocol: Sweep net; eventDate: 2015-06-16; verbatimEventDate: 16/Jul/2015; **Record Level:** institutionCode: ZMMU; basisOfRecord: PreservedSpecimen

#### Distribution

First records from Belarus and Russia: RUE.

### Phylidorea (Phylidorea) heterogyna

(Bergroth, 1913)

491F4294-B732-51E0-9FBA-E80517CCDFDC

https://ccw.naturalis.nl/detail.php?id=7149

#### Materials

- **Type status:** Other material. **Occurrence:** occurrenceRemarks: 2 males; recordedBy: A. Polevoi; individualCount: 2; sex: male; preparations: Pinned; occurrenceID: EU_LIM_736; **Taxon:** scientificName: Phylidorea (Phylidorea) heterogyna (Bergroth, 1913); family: Limoniidae; genus: Phylidorea; subgenus: Phylidorea; specificEpithet: heterogyna; scientificNameAuthorship: (Bergroth, 1913); **Location:** country: Russia; stateProvince: North European Russia; county: Arkhangelsk region; municipality: Onega district; locality: Vyzhiga River; verbatimElevation: 200 m; minimumElevationInMeters: 200; decimalLatitude: 62.78168; decimalLongitude: 37.16135; **Identification:** identifiedBy: A. Polevoi; **Event:** samplingProtocol: Sweep net; eventDate: 2014-08-01; verbatimEventDate: 01/Aug/2014; **Record Level:** institutionCode: FRIP; basisOfRecord: PreservedSpecimen
- **Type status:** Other material. **Occurrence:** occurrenceRemarks: 1 male; recordedBy: D.I. Gavryushin; individualCount: 1; sex: male; occurrenceID: EU_LIM_737; **Taxon:** scientificName: Phylidorea (Phylidorea) heterogyna (Bergroth, 1913); family: Limoniidae; genus: Phylidorea; subgenus: Phylidorea; specificEpithet: heterogyna; scientificNameAuthorship: (Bergroth, 1913); **Location:** country: Russia; stateProvince: North European Russia; county: Arkhangelsk region; locality: Arkhangelsk; verbatimElevation: 37 m; minimumElevationInMeters: 37; decimalLatitude: 64.54662; decimalLongitude: 40.60855; **Identification:** identifiedBy: D.I. Gavryushin; **Event:** samplingProtocol: Sweep net; eventDate: 2011-08-05; verbatimEventDate: 05/Aug/2011; **Record Level:** institutionCode: ZMMU; basisOfRecord: PreservedSpecimen
- **Type status:** Other material. **Occurrence:** occurrenceRemarks: 4 males; recordedBy: D.I. Gavryushin; individualCount: 4; sex: male; occurrenceID: EU_LIM_738; **Taxon:** scientificName: Phylidorea (Phylidorea) heterogyna (Bergroth, 1913); family: Limoniidae; genus: Phylidorea; subgenus: Phylidorea; specificEpithet: heterogyna; scientificNameAuthorship: (Bergroth, 1913); **Location:** country: Russia; stateProvince: North European Russia; county: Arkhangelsk region; locality: Arkhangelsk; verbatimElevation: 20 m; minimumElevationInMeters: 20; decimalLatitude: 64.54738; decimalLongitude: 40.6001; **Identification:** identifiedBy: D.I. Gavryushin; **Event:** samplingProtocol: Sweep net; eventDate: 2011-08-05; verbatimEventDate: 05/Aug/2011; **Record Level:** institutionCode: ZMMU; basisOfRecord: PreservedSpecimen
- **Type status:** Other material. **Occurrence:** occurrenceRemarks: 12 males; recordedBy: D.I. Gavryushin; individualCount: 12; sex: male; occurrenceID: EU_LIM_739; **Taxon:** scientificName: Phylidorea (Phylidorea) heterogyna (Bergroth, 1913); family: Limoniidae; genus: Phylidorea; subgenus: Phylidorea; specificEpithet: heterogyna; scientificNameAuthorship: (Bergroth, 1913); **Location:** country: Russia; stateProvince: North European Russia; county: Arkhangelsk region; locality: Arkhangelsk; verbatimElevation: 19 m; minimumElevationInMeters: 19; decimalLatitude: 64.55212; decimalLongitude: 40.5913; **Identification:** identifiedBy: D.I. Gavryushin; **Event:** samplingProtocol: Sweep net; eventDate: 2011-08-04; verbatimEventDate: 04/Aug/2011; **Record Level:** institutionCode: ZMMU; basisOfRecord: PreservedSpecimen
- **Type status:** Other material. **Occurrence:** occurrenceRemarks: 1 male, 2 females; recordedBy: D.I. Gavryushin; individualCount: 3; sex: male, female; occurrenceID: EU_LIM_740; **Taxon:** scientificName: Phylidorea (Phylidorea) heterogyna (Bergroth, 1913); family: Limoniidae; genus: Phylidorea; subgenus: Phylidorea; specificEpithet: heterogyna; scientificNameAuthorship: (Bergroth, 1913); **Location:** country: Russia; stateProvince: North European Russia; county: Arkhangelsk region; locality: Arkhangelsk; verbatimElevation: 19 m; minimumElevationInMeters: 19; decimalLatitude: 64.55212; decimalLongitude: 40.5913; **Identification:** identifiedBy: D.I. Gavryushin; **Event:** samplingProtocol: Sweep net; eventDate: 2011-08-06; verbatimEventDate: 06/Aug/2011; **Record Level:** institutionCode: ZMMU; basisOfRecord: PreservedSpecimen
- **Type status:** Other material. **Occurrence:** occurrenceRemarks: 1 male; recordedBy: A. Polevoi; individualCount: 1; sex: male; preparations: Pinned; occurrenceID: EU_LIM_741; **Taxon:** scientificName: Phylidorea (Phylidorea) heterogyna (Bergroth, 1913); family: Limoniidae; genus: Phylidorea; subgenus: Phylidorea; specificEpithet: heterogyna; scientificNameAuthorship: (Bergroth, 1913); **Location:** country: Russia; stateProvince: North European Russia; county: Republic Karelia; municipality: Suojarvi district; locality: Ar'koila, 1.5 km NE; verbatimElevation: 163 m; minimumElevationInMeters: 163; decimalLatitude: 61.93518; decimalLongitude: 32.84214; **Identification:** identifiedBy: A. Polevoi; **Event:** samplingProtocol: Sweep net; eventDate: 2019-08-21; verbatimEventDate: 21/Aug/2019; **Record Level:** institutionCode: FRIP; basisOfRecord: PreservedSpecimen
- **Type status:** Other material. **Occurrence:** occurrenceRemarks: 1 male; recordedBy: A. Polevoi; individualCount: 1; sex: male; preparations: Pinned; occurrenceID: EU_LIM_742; **Taxon:** scientificName: Phylidorea (Phylidorea) heterogyna (Bergroth, 1913); family: Limoniidae; genus: Phylidorea; subgenus: Phylidorea; specificEpithet: heterogyna; scientificNameAuthorship: (Bergroth, 1913); **Location:** country: Russia; stateProvince: North European Russia; county: Republic Karelia; municipality: Pudozh district; locality: River Shoikapolda, Lake Lambuda; verbatimElevation: 170 m; minimumElevationInMeters: 170; decimalLatitude: 62.53005; decimalLongitude: 37.33701; **Identification:** identifiedBy: A. Polevoi; **Event:** samplingProtocol: Sweep net; eventDate: 2006-08-23; verbatimEventDate: 23/Aug/2006; **Record Level:** institutionCode: FRIP; basisOfRecord: PreservedSpecimen
- **Type status:** Other material. **Occurrence:** occurrenceRemarks: 2 males; recordedBy: A. Polevoi; individualCount: 2; sex: male; preparations: Pinned; occurrenceID: EU_LIM_743; **Taxon:** scientificName: Phylidorea (Phylidorea) heterogyna (Bergroth, 1913); family: Limoniidae; genus: Phylidorea; subgenus: Phylidorea; specificEpithet: heterogyna; scientificNameAuthorship: (Bergroth, 1913); **Location:** country: Russia; stateProvince: North European Russia; county: Republic Karelia; municipality: Kalevala district; locality: Hautalampi Lake; verbatimElevation: 140 m; minimumElevationInMeters: 140; decimalLatitude: 64.57388; decimalLongitude: 31.75337; **Identification:** identifiedBy: A. Polevoi; **Event:** samplingProtocol: Sweep net; eventDate: 2016-08-12; verbatimEventDate: 12/Aug/2016; **Record Level:** institutionCode: FRIP; basisOfRecord: PreservedSpecimen

#### Distribution

First records from Russia: RUN.

### Phylidorea (Phylidorea) longicornis longicornis

(Schummel, 1829)

3D3587CD-3ECF-54CF-9D91-2A08A72ACE9D

https://ccw.naturalis.nl/detail.php?id=7152

#### Materials

- **Type status:** Other material. **Occurrence:** occurrenceRemarks: 2 males; recordedBy: D.I. Gavryushin; individualCount: 2; sex: male; occurrenceID: EU_LIM_744; **Taxon:** scientificName: Phylidorea (Phylidorea) longicornis longicornis (Schummel, 1829); family: Limoniidae; genus: Phylidorea; subgenus: Phylidorea; specificEpithet: longicornis; infraspecificEpithet: longicornis; scientificNameAuthorship: (Schummel, 1829); **Location:** country: Russia; stateProvince: East European Russia; county: Bashkortostan Respublika; municipality: Uchaly district; locality: Uzunkul Lake, 1km S of Ozernyi; verbatimElevation: 504 m; minimumElevationInMeters: 504; decimalLatitude: 53.96357; decimalLongitude: 58.85165; **Identification:** identifiedBy: D.I. Gavryushin; **Event:** samplingProtocol: Sweep net; eventDate: 2015-06-14; verbatimEventDate: 14/Jul/2015; **Record Level:** institutionCode: ZMMU; basisOfRecord: PreservedSpecimen
- **Type status:** Other material. **Occurrence:** occurrenceRemarks: 1 male; recordedBy: D.I. Gavryushin; individualCount: 1; sex: male; occurrenceID: EU_LIM_745; **Taxon:** scientificName: Phylidorea (Phylidorea) longicornis longicornis (Schummel, 1829); family: Limoniidae; genus: Phylidorea; subgenus: Phylidorea; specificEpithet: longicornis; infraspecificEpithet: longicornis; scientificNameAuthorship: (Schummel, 1829); **Location:** country: Russia; stateProvince: East European Russia; county: Bashkortostan Respublika; municipality: Beloretsk district; locality: Nura River (ca. 4km W of Otnurok village), at the foot of Zolotyie Shishki (Golden Cones) Mts.; verbatimElevation: 607 m; minimumElevationInMeters: 607; decimalLatitude: 54.05155; decimalLongitude: 58.26887; **Identification:** identifiedBy: D.I. Gavryushin; **Event:** samplingProtocol: Sweep net; eventDate: 2015-06-13; verbatimEventDate: 13/Jul/2015; **Record Level:** institutionCode: ZMMU; basisOfRecord: PreservedSpecimen
- **Type status:** Other material. **Occurrence:** occurrenceRemarks: 2 males; recordedBy: D.I. Gavryushin; individualCount: 2; sex: male; occurrenceID: EU_LIM_746; **Taxon:** scientificName: Phylidorea (Phylidorea) longicornis longicornis (Schummel, 1829); family: Limoniidae; genus: Phylidorea; subgenus: Phylidorea; specificEpithet: longicornis; infraspecificEpithet: longicornis; scientificNameAuthorship: (Schummel, 1829); **Location:** country: Russia; stateProvince: East European Russia; county: Bashkortostan Respublika; municipality: Beloretsk district; locality: Nura River (ca. 4km W of Otnurok village), at the foot of Zolotyie Shishki (Golden Cones) Mts.; verbatimElevation: 607 m; minimumElevationInMeters: 607; decimalLatitude: 54.05155; decimalLongitude: 58.26887; **Identification:** identifiedBy: D.I. Gavryushin; **Event:** samplingProtocol: Sweep net; eventDate: 2015-06-16; verbatimEventDate: 16/Jul/2015; **Record Level:** institutionCode: ZMMU; basisOfRecord: PreservedSpecimen
- **Type status:** Other material. **Occurrence:** occurrenceRemarks: 1 female; recordedBy: D.I. Gavryushin; individualCount: 1; sex: female; occurrenceID: EU_LIM_747; **Taxon:** scientificName: Phylidorea (Phylidorea) longicornis longicornis (Schummel, 1829); family: Limoniidae; genus: Phylidorea; subgenus: Phylidorea; specificEpithet: longicornis; infraspecificEpithet: longicornis; scientificNameAuthorship: (Schummel, 1829); **Location:** country: Russia; stateProvince: East European Russia; county: Bashkortostan Respublika; municipality: Beloretsk district; locality: Makhmutovo env., Belaya River; verbatimElevation: 550 m; minimumElevationInMeters: 550; decimalLatitude: 54.33012; decimalLongitude: 58.80735; **Identification:** identifiedBy: D.I. Gavryushin; **Event:** samplingProtocol: Sweep net; eventDate: 2015-06-15; verbatimEventDate: 15/Jul/2015; **Record Level:** institutionCode: ZMMU; basisOfRecord: PreservedSpecimen

#### Distribution

First records from Russia: RUE.

### Phylidorea (Phylidorea) umbrarum

(Krogerus, 1937)

4CAE5E51-D199-50CF-8F36-2D80CABF6E0D

https://ccw.naturalis.nl/detail.php?id=7168

#### Materials

- **Type status:** Other material. **Occurrence:** occurrenceRemarks: 3 males, 2 females; recordedBy: D.I. Gavryushin; individualCount: 5; sex: male, female; occurrenceID: EU_LIM_748; **Taxon:** scientificName: Phylidorea (Phylidorea) umbrarum (Krogerus, 1937); family: Limoniidae; genus: Phylidorea; subgenus: Phylidorea; specificEpithet: umbrarum; scientificNameAuthorship: (Krogerus, 1937); **Location:** country: Russia; stateProvince: East European Russia; county: Bashkortostan Respublika; municipality: Uchaly district; locality: Uzunkul Lake, 1km S of Ozernyi; verbatimElevation: 504 m; minimumElevationInMeters: 504; decimalLatitude: 53.96357; decimalLongitude: 58.85165; **Identification:** identifiedBy: D.I. Gavryushin; **Event:** samplingProtocol: Sweep net; eventDate: 2015-06-14; verbatimEventDate: 14/Jul/2015; **Record Level:** institutionCode: ZMMU; basisOfRecord: PreservedSpecimen
- **Type status:** Other material. **Occurrence:** occurrenceRemarks: 1 male; recordedBy: D.I. Gavryushin; individualCount: 1; sex: male; occurrenceID: EU_LIM_749; **Taxon:** scientificName: Phylidorea (Phylidorea) umbrarum (Krogerus, 1937); family: Limoniidae; genus: Phylidorea; subgenus: Phylidorea; specificEpithet: umbrarum; scientificNameAuthorship: (Krogerus, 1937); **Location:** country: Russia; stateProvince: East European Russia; county: Bashkortostan Respublika; municipality: Beloretsk district; locality: Makhmutovo env., Belaya River; verbatimElevation: 550 m; minimumElevationInMeters: 550; decimalLatitude: 54.33012; decimalLongitude: 58.80735; **Identification:** identifiedBy: D.I. Gavryushin; **Event:** samplingProtocol: Sweep net; eventDate: 2015-06-15; verbatimEventDate: 15/Jul/2015; **Record Level:** institutionCode: ZMMU; basisOfRecord: PreservedSpecimen
- **Type status:** Other material. **Occurrence:** occurrenceRemarks: 1 male; recordedBy: N.M. Paramonov; individualCount: 1; sex: male; occurrenceID: EU_LIM_750; **Taxon:** scientificName: Phylidorea (Phylidorea) umbrarum (Krogerus, 1937); family: Limoniidae; genus: Phylidorea; subgenus: Phylidorea; specificEpithet: umbrarum; scientificNameAuthorship: (Krogerus, 1937); **Location:** country: Russia; stateProvince: East European Russia; county: Tatarstan Respublika; municipality: Laishevo district; locality: Volga-Kama State Nature Biosphere Reserve, «Saraly»; verbatimElevation: 71 m; minimumElevationInMeters: 71; decimalLatitude: 55.29303; decimalLongitude: 49.29976; **Identification:** identifiedBy: N.M. Paramonov; **Event:** samplingProtocol: Sweep net; eventDate: 2009-06-18; verbatimEventDate: 18/Jun/2009; habitat: wetland; **Record Level:** institutionCode: ZIN; basisOfRecord: PreservedSpecimen
- **Type status:** Other material. **Occurrence:** occurrenceRemarks: 1 male; recordedBy: N.M. Paramonov; individualCount: 1; sex: male; occurrenceID: EU_LIM_751; **Taxon:** scientificName: Phylidorea (Phylidorea) umbrarum (Krogerus, 1937); family: Limoniidae; genus: Phylidorea; subgenus: Phylidorea; specificEpithet: umbrarum; scientificNameAuthorship: (Krogerus, 1937); **Location:** country: Russia; stateProvince: East European Russia; county: Tatarstan Respublika; municipality: Zelenodol’sk district; locality: Zaymishche env., Geomagnetic station; verbatimElevation: 87 m; minimumElevationInMeters: 87; decimalLatitude: 55.82684; decimalLongitude: 48.84395; **Identification:** identifiedBy: N.M. Paramonov; **Event:** samplingProtocol: Sweep net; eventDate: 2011-08-19/2011-08-21; verbatimEventDate: 19-21/Aug/2011; **Record Level:** institutionCode: ZIN; basisOfRecord: PreservedSpecimen
- **Type status:** Other material. **Occurrence:** occurrenceRemarks: 1 male; recordedBy: N.M. Paramonov; individualCount: 1; sex: male; occurrenceID: EU_LIM_752; **Taxon:** scientificName: Phylidorea (Phylidorea) umbrarum (Krogerus, 1937); family: Limoniidae; genus: Phylidorea; subgenus: Phylidorea; specificEpithet: umbrarum; scientificNameAuthorship: (Krogerus, 1937); **Location:** country: Russia; stateProvince: East European Russia; county: Tatarstan Respublika; municipality: Zelenodol’sk district; locality: Volga-Kama State Nature Biosphere Reserve, «Raifa», Serbulak River; verbatimElevation: 100 m; minimumElevationInMeters: 100; decimalLatitude: 55.88868; decimalLongitude: 48.71434; **Identification:** identifiedBy: N.M. Paramonov; **Event:** samplingProtocol: Sweep net; eventDate: 2008-06-27; verbatimEventDate: 27/Jul/2008; **Record Level:** institutionCode: ZIN; basisOfRecord: PreservedSpecimen

#### Distribution

First records from Russia: RUE.

### Phyllolabis alexanderi

Lackschewitz, 1940

8133C3D1-198B-55C8-8178-F3FA8532E91D

https://ccw.naturalis.nl/detail.php?id=3769

#### Materials

- **Type status:** Other material. **Occurrence:** occurrenceRemarks: 3 males, 1 female; recordedBy: L.-P. Kolcsár; individualCount: 4; sex: male, female; preparations: Ethanol; occurrenceID: EU_LIM_753; **Taxon:** scientificName: Phyllolabisalexanderi Lackschewitz, 1940; family: Limoniidae; genus: Phyllolabis; specificEpithet: alexanderi; scientificNameAuthorship: Lackschewitz, 1940; **Location:** country: North Macedonia; municipality: Bratin Dol; locality: Pelister Mts.; verbatimElevation: 875 m; minimumElevationInMeters: 875; decimalLatitude: 41.06944; decimalLongitude: 21.23435; **Identification:** identifiedBy: L.-P. Kolcsár; **Event:** samplingProtocol: Sweep net; eventDate: 2012-05-03; verbatimEventDate: 3/May/2012; habitat: brook; **Record Level:** institutionCode: CKLP; basisOfRecord: PreservedSpecimen

#### Description

Figs 14, 15, 16

#### Distribution

First record from North Macedonia.

### Pilaria decolor

(Zetterstedt, 1851)

98C11579-08F6-5A0B-A211-A2FB712EE17D

https://ccw.naturalis.nl/detail.php?id=7184

#### Materials

- **Type status:** Other material. **Occurrence:** occurrenceRemarks: 4 males, 6 females; recordedBy: D.I. Gavryushin; individualCount: 10; sex: male, female; preparations: Pinned; occurrenceID: EU_LIM_754; **Taxon:** scientificName: Pilariadecolor (Zetterstedt, 1851); family: Limoniidae; genus: Pilaria; specificEpithet: decolor; scientificNameAuthorship: (Zetterstedt, 1851); **Location:** country: Belarus; stateProvince: Minsk; county: Barysaw; locality: Glivin; verbatimElevation: 161 m; minimumElevationInMeters: 161; decimalLatitude: 54.14902; decimalLongitude: 28.63648; **Identification:** identifiedBy: D.I. Gavryushin; **Event:** samplingProtocol: Sweep net; eventDate: 2013-06-06; verbatimEventDate: 6/Jul/2013; **Record Level:** institutionCode: ZMMU; basisOfRecord: PreservedSpecimen
- **Type status:** Other material. **Occurrence:** occurrenceRemarks: 6 males, 2 females; recordedBy: D.I. Gavryushin; individualCount: 8; sex: male, female; preparations: Pinned; occurrenceID: EU_LIM_755; **Taxon:** scientificName: Pilariadecolor (Zetterstedt, 1851); family: Limoniidae; genus: Pilaria; specificEpithet: decolor; scientificNameAuthorship: (Zetterstedt, 1851); **Location:** country: Belarus; stateProvince: Minsk; county: Barysaw; locality: Vialikaje Stachava; verbatimElevation: 156 m; minimumElevationInMeters: 156; decimalLatitude: 54.26555; decimalLongitude: 28.38332; **Identification:** identifiedBy: D.I. Gavryushin; **Event:** samplingProtocol: Sweep net; eventDate: 2013-06-07; verbatimEventDate: 7/Jul/2013; **Record Level:** institutionCode: ZMMU; basisOfRecord: PreservedSpecimen
- **Type status:** Other material. **Occurrence:** occurrenceRemarks: 1 male; recordedBy: L.-P. Kolcsár; individualCount: 1; sex: male; preparations: Ethanol; occurrenceID: EU_LIM_756; **Taxon:** scientificName: Pilariadecolor (Zetterstedt, 1851); family: Limoniidae; genus: Pilaria; specificEpithet: decolor; scientificNameAuthorship: (Zetterstedt, 1851); **Location:** country: Romania; stateProvince: Harghita; municipality: Voșlăbeni; locality: Senetea; verbatimElevation: 764 m; minimumElevationInMeters: 764; decimalLatitude: 46.62588; decimalLongitude: 25.59745; **Identification:** identifiedBy: L.-P. Kolcsár; **Event:** samplingProtocol: Sweep net; eventDate: 2011-06-02; verbatimEventDate: 2/Jul/2011; habitat: marshy meadow; **Record Level:** institutionCode: CKLP; basisOfRecord: PreservedSpecimen
- **Type status:** Other material. **Occurrence:** occurrenceRemarks: 1 male; recordedBy: D.I. Gavryushin; individualCount: 1; sex: male; occurrenceID: EU_LIM_757; **Taxon:** scientificName: Pilariadecolor (Zetterstedt, 1851); family: Limoniidae; genus: Pilaria; specificEpithet: decolor; scientificNameAuthorship: (Zetterstedt, 1851); **Location:** country: Russia; stateProvince: North European Russia; county: Arkhangelsk region; locality: Arkhangelsk; verbatimElevation: 20 m; minimumElevationInMeters: 20; decimalLatitude: 64.54738; decimalLongitude: 40.6001; **Identification:** identifiedBy: D.I. Gavryushin; **Event:** samplingProtocol: Sweep net; eventDate: 2011-08-05; verbatimEventDate: 05/Aug/2011; **Record Level:** institutionCode: ZMMU; basisOfRecord: PreservedSpecimen
- **Type status:** Other material. **Occurrence:** occurrenceRemarks: 2 females; recordedBy: D.I. Gavryushin; individualCount: 2; sex: female; occurrenceID: EU_LIM_758; **Taxon:** scientificName: Pilariadecolor (Zetterstedt, 1851); family: Limoniidae; genus: Pilaria; specificEpithet: decolor; scientificNameAuthorship: (Zetterstedt, 1851); **Location:** country: Russia; stateProvince: North European Russia; county: Arkhangelsk region; locality: Arkhangelsk; verbatimElevation: 19 m; minimumElevationInMeters: 19; decimalLatitude: 64.55212; decimalLongitude: 40.5913; **Identification:** identifiedBy: D.I. Gavryushin; **Event:** samplingProtocol: Sweep net; eventDate: 2011-08-04; verbatimEventDate: 04/Aug/2011; **Record Level:** institutionCode: ZMMU; basisOfRecord: PreservedSpecimen
- **Type status:** Other material. **Occurrence:** occurrenceRemarks: 3 males, 2 females; recordedBy: D.I. Gavryushin; individualCount: 5; sex: male, female; occurrenceID: EU_LIM_759; **Taxon:** scientificName: Pilariadecolor (Zetterstedt, 1851); family: Limoniidae; genus: Pilaria; specificEpithet: decolor; scientificNameAuthorship: (Zetterstedt, 1851); **Location:** country: Russia; stateProvince: North European Russia; county: Arkhangelsk region; locality: Arkhangelsk; verbatimElevation: 25 m; minimumElevationInMeters: 25; decimalLatitude: 64.55542; decimalLongitude: 40.61068; **Identification:** identifiedBy: D.I. Gavryushin; **Event:** samplingProtocol: Sweep net; eventDate: 2011-08-06; verbatimEventDate: 06/Aug/2011; **Record Level:** institutionCode: ZMMU; basisOfRecord: PreservedSpecimen
- **Type status:** Other material. **Occurrence:** occurrenceRemarks: 3 males; recordedBy: D.I. Gavryushin; individualCount: 3; sex: male; occurrenceID: EU_LIM_760; **Taxon:** scientificName: Pilariadecolor (Zetterstedt, 1851); family: Limoniidae; genus: Pilaria; specificEpithet: decolor; scientificNameAuthorship: (Zetterstedt, 1851); **Location:** country: Russia; stateProvince: North European Russia; county: Arkhangelsk region; municipality: Primorsky district; locality: Lodma River; verbatimElevation: 19 m; minimumElevationInMeters: 19; decimalLatitude: 64.67958; decimalLongitude: 40.73225; **Identification:** identifiedBy: D.I. Gavryushin; **Event:** samplingProtocol: Sweep net; eventDate: 2011-08-07; verbatimEventDate: 07/Aug/2011; **Record Level:** institutionCode: ZMMU; basisOfRecord: PreservedSpecimen
- **Type status:** Other material. **Occurrence:** occurrenceRemarks: 1 male; recordedBy: D.I. Gavryushin; individualCount: 1; sex: male; occurrenceID: EU_LIM_761; **Taxon:** scientificName: Pilariadecolor (Zetterstedt, 1851); family: Limoniidae; genus: Pilaria; specificEpithet: decolor; scientificNameAuthorship: (Zetterstedt, 1851); **Location:** country: Russia; stateProvince: East European Russia; county: Bashkortostan Respublika; municipality: Beloretsk district; locality: Abzakovo env., Malyi Kizil River; verbatimElevation: 510 m; minimumElevationInMeters: 510; decimalLatitude: 53.81428; decimalLongitude: 58.5942; **Identification:** identifiedBy: D.I. Gavryushin; **Event:** samplingProtocol: Sweep net; eventDate: 2015-06-12; verbatimEventDate: 12/Jul/2015; **Record Level:** institutionCode: ZMMU; basisOfRecord: PreservedSpecimen
- **Type status:** Other material. **Occurrence:** occurrenceRemarks: 1 female; recordedBy: D.I. Gavryushin; individualCount: 1; sex: female; occurrenceID: EU_LIM_762; **Taxon:** scientificName: Pilariadecolor (Zetterstedt, 1851); family: Limoniidae; genus: Pilaria; specificEpithet: decolor; scientificNameAuthorship: (Zetterstedt, 1851); **Location:** country: Russia; stateProvince: East European Russia; county: Bashkortostan Respublika; municipality: Uchaly district; locality: Uzunkul Lake, 1km S of Ozernyi; verbatimElevation: 504 m; minimumElevationInMeters: 504; decimalLatitude: 53.96357; decimalLongitude: 58.85165; **Identification:** identifiedBy: D.I. Gavryushin; **Event:** samplingProtocol: Sweep net; eventDate: 2015-06-14; verbatimEventDate: 14/Jul/2015; **Record Level:** institutionCode: ZMMU; basisOfRecord: PreservedSpecimen
- **Type status:** Other material. **Occurrence:** occurrenceRemarks: 1 male; recordedBy: A. Polevoi; individualCount: 1; sex: male; preparations: Pinned; occurrenceID: EU_LIM_763; **Taxon:** scientificName: Pilariadecolor (Zetterstedt, 1851); family: Limoniidae; genus: Pilaria; specificEpithet: decolor; scientificNameAuthorship: (Zetterstedt, 1851); **Location:** country: Russia; stateProvince: North European Russia; county: Republic Karelia; municipality: Medvezhegorsk district; locality: Bol'shoe Obozero oz.; verbatimElevation: 60 m; minimumElevationInMeters: 60; decimalLatitude: 61.90122; decimalLongitude: 35.21631; **Identification:** identifiedBy: A. Polevoi; **Event:** samplingProtocol: Sweep net; eventDate: 1997-06-11; verbatimEventDate: 11/Jul/1997; **Record Level:** institutionCode: FRIP; basisOfRecord: PreservedSpecimen
- **Type status:** Other material. **Occurrence:** occurrenceRemarks: 1 male; recordedBy: A. Polevoi; individualCount: 1; sex: male; preparations: Pinned; occurrenceID: EU_LIM_764; **Taxon:** scientificName: Pilariadecolor (Zetterstedt, 1851); family: Limoniidae; genus: Pilaria; specificEpithet: decolor; scientificNameAuthorship: (Zetterstedt, 1851); **Location:** island: Ernitskiy; country: Russia; stateProvince: North European Russia; county: Republic Karelia; municipality: Medvezhegorsk district; locality: Ernitskiy Island; verbatimElevation: 25 m; minimumElevationInMeters: 25; decimalLatitude: 61.98701; decimalLongitude: 35.17072; **Identification:** identifiedBy: A. Polevoi; **Event:** samplingProtocol: Sweep net; eventDate: 2018-06-29; verbatimEventDate: 29/Jul/2018; **Record Level:** institutionCode: FRIP; basisOfRecord: PreservedSpecimen
- **Type status:** Other material. **Occurrence:** occurrenceRemarks: 1 male; recordedBy: A. Polevoi; individualCount: 1; sex: male; preparations: Pinned; occurrenceID: EU_LIM_765; **Taxon:** scientificName: Pilariadecolor (Zetterstedt, 1851); family: Limoniidae; genus: Pilaria; specificEpithet: decolor; scientificNameAuthorship: (Zetterstedt, 1851); **Location:** country: Russia; stateProvince: North European Russia; county: Republic Karelia; municipality: Medvezhegorsk district; locality: Kurgenitsy, 2 km NE; verbatimElevation: 25 m; minimumElevationInMeters: 25; decimalLatitude: 62.08296; decimalLongitude: 35.32056; **Identification:** identifiedBy: A. Polevoi; **Event:** samplingProtocol: Sweep net; eventDate: 2000-06-19; verbatimEventDate: 19/Jul/2000; **Record Level:** institutionCode: FRIP; basisOfRecord: PreservedSpecimen
- **Type status:** Other material. **Occurrence:** occurrenceRemarks: 1 male; recordedBy: A. Polevoi; individualCount: 1; sex: male; preparations: Pinned; occurrenceID: EU_LIM_766; **Taxon:** scientificName: Pilariadecolor (Zetterstedt, 1851); family: Limoniidae; genus: Pilaria; specificEpithet: decolor; scientificNameAuthorship: (Zetterstedt, 1851); **Location:** country: Russia; stateProvince: North European Russia; county: Republic Karelia; municipality: Kondopoga district; locality: Kivach Nature Reserve; verbatimElevation: 70 m; minimumElevationInMeters: 70; decimalLatitude: 62.28148; decimalLongitude: 33.96745; **Identification:** identifiedBy: A. Polevoi; **Event:** samplingProtocol: Trunk emergence trap; eventDate: 2017-06-17/2017-08-18; verbatimEventDate: 17/Jul-18/Aug/2017; **Record Level:** institutionCode: FRIP; basisOfRecord: PreservedSpecimen
- **Type status:** Other material. **Occurrence:** occurrenceRemarks: 1 male; recordedBy: A. Polevoi; individualCount: 1; sex: male; preparations: Pinned; occurrenceID: EU_LIM_767; **Taxon:** scientificName: Pilariadecolor (Zetterstedt, 1851); family: Limoniidae; genus: Pilaria; specificEpithet: decolor; scientificNameAuthorship: (Zetterstedt, 1851); **Location:** country: Russia; stateProvince: North European Russia; county: Republic Karelia; municipality: Pudozh district; locality: River Shoikapolda; verbatimElevation: 170 m; minimumElevationInMeters: 170; decimalLatitude: 62.52823; decimalLongitude: 37.37746; **Identification:** identifiedBy: A. Polevoi; **Event:** samplingProtocol: Sweep net; eventDate: 2006-08-22; verbatimEventDate: 22/Aug/2006; **Record Level:** institutionCode: FRIP; basisOfRecord: PreservedSpecimen

#### Distribution

First records from Belarus, Romania and Russia: RUE, RUN.

### Pilaria discicollis

(Meigen, 1818)

88FDDE94-9235-5AE2-AFAD-3735BBE9C99F

https://ccw.naturalis.nl/detail.php?id=7185

#### Materials

- **Type status:** Other material. **Occurrence:** occurrenceRemarks: 1 male; recordedBy: D.I. Gavryushin; individualCount: 1; sex: male; preparations: Pinned; occurrenceID: EU_LIM_768; **Taxon:** scientificName: Pilariadiscicollis (Meigen, 1818); family: Limoniidae; genus: Pilaria; specificEpithet: discicollis; scientificNameAuthorship: (Meigen, 1818); **Location:** country: Belarus; stateProvince: Minsk; county: Barysaw; locality: Barysaw; verbatimElevation: 155 m; minimumElevationInMeters: 155; decimalLatitude: 54.25542; decimalLongitude: 28.48092; **Identification:** identifiedBy: D.I. Gavryushin; **Event:** samplingProtocol: Sweep net; eventDate: 2013-06-05; verbatimEventDate: 5/Jul/2013; **Record Level:** institutionCode: ZMMU; basisOfRecord: PreservedSpecimen
- **Type status:** Other material. **Occurrence:** occurrenceRemarks: 1 male; recordedBy: L.-P. Kolcsár; individualCount: 1; sex: male; preparations: Ethanol; occurrenceID: EU_LIM_769; **Taxon:** scientificName: Pilariadiscicollis (Meigen, 1818); family: Limoniidae; genus: Pilaria; specificEpithet: discicollis; scientificNameAuthorship: (Meigen, 1818); **Location:** country: Greece; stateProvince: Eastern Macedonia and Thrace; municipality: Chrysoupoli; locality: Nea Karya, Nestos River and Delta; verbatimElevation: 3 m; minimumElevationInMeters: 3; decimalLatitude: 40.87841; decimalLongitude: 24.78541; **Identification:** identifiedBy: L.-P. Kolcsár; **Event:** samplingProtocol: Sweep net; eventDate: 2011-05-26; verbatimEventDate: 26/May/2011; **Record Level:** institutionCode: CKLP; basisOfRecord: PreservedSpecimen

#### Distribution

First records from Belarus and Greece (from mainland).

### Pilaria fuscipennis

(Meigen, 1818)

4E8E8D5F-53F6-5BFC-A737-90F1EBFB4D52

https://ccw.naturalis.nl/detail.php?id=7191

#### Materials

- **Type status:** Other material. **Occurrence:** occurrenceRemarks: 1 female; recordedBy: D.I. Gavryushin; individualCount: 1; sex: female; preparations: Pinned; occurrenceID: EU_LIM_770; **Taxon:** scientificName: Pilariafuscipennis (Meigen, 1818); family: Limoniidae; genus: Pilaria; specificEpithet: fuscipennis; scientificNameAuthorship: (Meigen, 1818); **Location:** country: Belarus; stateProvince: Minsk; county: Barysaw; locality: Glivin; verbatimElevation: 161 m; minimumElevationInMeters: 161; decimalLatitude: 54.14902; decimalLongitude: 28.63648; **Identification:** identifiedBy: D.I. Gavryushin; **Event:** samplingProtocol: Sweep net; eventDate: 2013-06-06; verbatimEventDate: 6/Jul/2013; **Record Level:** institutionCode: ZMMU; basisOfRecord: PreservedSpecimen
- **Type status:** Other material. **Occurrence:** occurrenceRemarks: 1 female; recordedBy: D.I. Gavryushin; individualCount: 1; sex: female; preparations: Pinned; occurrenceID: EU_LIM_771; **Taxon:** scientificName: Pilariafuscipennis (Meigen, 1818); family: Limoniidae; genus: Pilaria; specificEpithet: fuscipennis; scientificNameAuthorship: (Meigen, 1818); **Location:** country: Belarus; stateProvince: Minsk; county: Barysaw; locality: Vialikaje Stachava; verbatimElevation: 156 m; minimumElevationInMeters: 156; decimalLatitude: 54.26555; decimalLongitude: 28.38332; **Identification:** identifiedBy: D.I. Gavryushin; **Event:** samplingProtocol: Sweep net; eventDate: 2013-06-07; verbatimEventDate: 7/Jul/2013; **Record Level:** institutionCode: ZMMU; basisOfRecord: PreservedSpecimen
- **Type status:** Other material. **Occurrence:** occurrenceRemarks: 1 male; recordedBy: J. Starý; individualCount: 1; sex: male; preparations: Pinned; occurrenceID: EU_LIM_772; **Taxon:** scientificName: Pilariafuscipennis (Meigen, 1818); family: Limoniidae; genus: Pilaria; specificEpithet: fuscipennis; scientificNameAuthorship: (Meigen, 1818); **Location:** country: Greece; stateProvince: Peloponnese; municipality: Dými; locality: 0.6-1 km W Vithoulkas; verbatimElevation: 200-250 m; minimumElevationInMeters: 200; maximumElevationInMeters: 250; decimalLatitude: 38.0644; decimalLongitude: 21.5429; **Identification:** identifiedBy: J. Starý; **Event:** eventDate: 2015-05-25; verbatimEventDate: 25/May/2015; habitat: brook; **Record Level:** institutionCode: PCJS; basisOfRecord: PreservedSpecimen

#### Distribution

First records from Belarus and Greece (from mainland).

### Pilaria meridiana

(Staeger, 1840)

E218355E-7ECF-5087-A431-EEB0EA5A41FA

https://ccw.naturalis.nl/detail.php?id=7198

#### Materials

- **Type status:** Other material. **Occurrence:** occurrenceRemarks: 1 male, 1 female; recordedBy: N.M. Paramonov; individualCount: 2; sex: male, female; occurrenceID: EU_LIM_773; **Taxon:** scientificName: Pilariameridiana (Staeger, 1840); family: Limoniidae; genus: Pilaria; specificEpithet: meridiana; scientificNameAuthorship: (Staeger, 1840); **Location:** country: Russia; stateProvince: East European Russia; county: Tatarstan Respublika; municipality: Laishevo district; locality: Volga-Kama State Nature Biosphere Reserve, «Saraly»; verbatimElevation: 71 m; minimumElevationInMeters: 71; decimalLatitude: 55.29303; decimalLongitude: 49.29976; **Identification:** identifiedBy: N.M. Paramonov; **Event:** samplingProtocol: Sweep net; eventDate: 2009-06-18; verbatimEventDate: 18/Jun/2009; habitat: wetland; **Record Level:** institutionCode: ZIN; basisOfRecord: PreservedSpecimen
- **Type status:** Other material. **Occurrence:** occurrenceRemarks: 2 males; recordedBy: N.M. Paramonov; individualCount: 2; sex: male; occurrenceID: EU_LIM_774; **Taxon:** scientificName: Pilariameridiana (Staeger, 1840); family: Limoniidae; genus: Pilaria; specificEpithet: meridiana; scientificNameAuthorship: (Staeger, 1840); **Location:** country: Russia; stateProvince: East European Russia; county: Tatarstan Respublika; municipality: Laishevo district; locality: Volga-Kama State Nature Biosphere Reserve, «Saraly»; verbatimElevation: 71 m; minimumElevationInMeters: 71; decimalLatitude: 55.29303; decimalLongitude: 49.29976; **Identification:** identifiedBy: N.M. Paramonov; **Event:** samplingProtocol: Sweep net; eventDate: 2009-06-24; verbatimEventDate: 24/Jun/2009; habitat: wetland; **Record Level:** institutionCode: ZIN; basisOfRecord: PreservedSpecimen
- **Type status:** Other material. **Occurrence:** occurrenceRemarks: 2 males; recordedBy: N.M. Paramonov; individualCount: 2; sex: male; occurrenceID: EU_LIM_775; **Taxon:** scientificName: Pilariameridiana (Staeger, 1840); family: Limoniidae; genus: Pilaria; specificEpithet: meridiana; scientificNameAuthorship: (Staeger, 1840); **Location:** country: Russia; stateProvince: East European Russia; county: Tatarstan Respublika; municipality: Laishevo district; locality: Volga-Kama State Nature Biosphere Reserve, «Saraly»; verbatimElevation: 71 m; minimumElevationInMeters: 71; decimalLatitude: 55.29303; decimalLongitude: 49.29976; **Identification:** identifiedBy: N.M. Paramonov; **Event:** samplingProtocol: Sweep net; eventDate: 2009-06-19; verbatimEventDate: 19/Jun/2009; habitat: wetland; **Record Level:** institutionCode: ZIN; basisOfRecord: PreservedSpecimen
- **Type status:** Other material. **Occurrence:** occurrenceRemarks: 1 male; recordedBy: N.M. Paramonov; individualCount: 1; sex: male; occurrenceID: EU_LIM_776; **Taxon:** scientificName: Pilariameridiana (Staeger, 1840); family: Limoniidae; genus: Pilaria; specificEpithet: meridiana; scientificNameAuthorship: (Staeger, 1840); **Location:** country: Russia; stateProvince: East European Russia; county: Tatarstan Respublika; municipality: Zelenodol’sk district; locality: Zaymishche env., Geomagnetic station; verbatimElevation: 87 m; minimumElevationInMeters: 87; decimalLatitude: 55.82684; decimalLongitude: 48.84395; **Identification:** identifiedBy: N.M. Paramonov; **Event:** samplingProtocol: Sweep net; eventDate: 2012-06-30; verbatimEventDate: 30/Jun/2012; **Record Level:** institutionCode: ZIN; basisOfRecord: PreservedSpecimen

#### Distribution

First records from Russia: RUE.

### Pilaria nigropunctata

(Agrell, 1945)

2DFDC994-F7DD-5BEF-8099-EA97885DED9B

https://ccw.naturalis.nl/detail.php?id=7200

#### Materials

- **Type status:** Other material. **Occurrence:** occurrenceRemarks: 2 males; recordedBy: C. Quindroit; individualCount: 2; sex: male; preparations: Ethanol; occurrenceID: EU_LIM_777; **Taxon:** scientificName: Pilarianigropunctata (Agrell, 1945); family: Limoniidae; genus: Pilaria; specificEpithet: nigropunctata; scientificNameAuthorship: (Agrell, 1945); **Location:** country: France; municipality: Notre-dame-d'Allençon; locality: Etang aux moines; verbatimElevation: 55 m; minimumElevationInMeters: 55; decimalLatitude: 47.31774; decimalLongitude: -0.43415; **Identification:** identifiedBy: C. Quindroit; **Event:** samplingProtocol: Sweep net; eventDate: 2020-06-10; verbatimEventDate: 10/Jun/2020; **Record Level:** institutionCode: PCCQ; basisOfRecord: PreservedSpecimen
- **Type status:** Other material. **Occurrence:** occurrenceRemarks: 1 male; recordedBy: C. Quindroit; individualCount: 1; sex: male; preparations: Ethanol; occurrenceID: EU_LIM_778; **Taxon:** scientificName: Pilarianigropunctata (Agrell, 1945); family: Limoniidae; genus: Pilaria; specificEpithet: nigropunctata; scientificNameAuthorship: (Agrell, 1945); **Location:** country: France; municipality: Mulsanne; locality: Les Faulx; verbatimElevation: 50 m; minimumElevationInMeters: 50; decimalLatitude: 47.93581; decimalLongitude: 0.24531; **Identification:** identifiedBy: C. Quindroit; **Event:** samplingProtocol: Sweep net; eventDate: 2020-06-20; verbatimEventDate: 20/Jun/2020; **Record Level:** institutionCode: PCCQ; basisOfRecord: PreservedSpecimen
- **Type status:** Other material. **Occurrence:** occurrenceRemarks: 1 male; recordedBy: L.-P. Kolcsár; individualCount: 1; sex: male; preparations: Ethanol; occurrenceID: EU_LIM_779; **Taxon:** scientificName: Pilarianigropunctata (Agrell, 1945); family: Limoniidae; genus: Pilaria; specificEpithet: nigropunctata; scientificNameAuthorship: (Agrell, 1945); **Location:** country: Latvia; municipality: Dolesmuiža; locality: Doles sala; verbatimElevation: 4 m; minimumElevationInMeters: 4; decimalLatitude: 56.866; decimalLongitude: 24.2014; **Identification:** identifiedBy: L.-P. Kolcsár; **Event:** samplingProtocol: Sweep net; eventDate: 2018-06-20; verbatimEventDate: 20/Jul/2018; **Record Level:** institutionCode: CKLP; basisOfRecord: PreservedSpecimen
- **Type status:** Other material. **Occurrence:** catalogNumber: 581910; occurrenceRemarks: 1 male; recordedBy: K.M. Olsen; individualCount: 1; sex: male; preparations: Ethanol; occurrenceID: EU_LIM_780; **Taxon:** scientificName: Pilarianigropunctata (Agrell, 1945); family: Limoniidae; genus: Pilaria; specificEpithet: nigropunctata; scientificNameAuthorship: (Agrell, 1945); **Location:** country: Norway; stateProvince: Akershus; municipality: Ski; locality: Kapelldammen; verbatimElevation: 125 m; minimumElevationInMeters: 125; decimalLatitude: 59.72433; decimalLongitude: 10.8386; **Identification:** identifiedBy: K.M. Olsen; **Event:** samplingProtocol: Sweep net; eventDate: 2018-06-12; verbatimEventDate: 12/Jun/2018; **Record Level:** institutionCode: PCKMO; basisOfRecord: PreservedSpecimen

#### Distribution

First records from France (from mainland), Latvia and Norway.

### Pilaria scutellata

(Staeger, 1840)

CC01D425-8264-573F-82C1-AE05F4FD779F

https://ccw.naturalis.nl/detail.php?id=7210

#### Materials

- **Type status:** Other material. **Occurrence:** occurrenceRemarks: 1 male; recordedBy: V.E. Pilipenko; individualCount: 1; sex: male; occurrenceID: EU_LIM_781; **Taxon:** scientificName: Pilariascutellata (Staeger, 1840); family: Limoniidae; genus: Pilaria; specificEpithet: scutellata; scientificNameAuthorship: (Staeger, 1840); **Location:** country: Russia; stateProvince: Central European Russia; county: Moskovskaya Oblast; municipality: Solnechnogorsk district; locality: city Zelenograd; verbatimElevation: 200 m; minimumElevationInMeters: 200; decimalLatitude: 55.98722; decimalLongitude: 37.20443; **Identification:** identifiedBy: V.E. Pilipenko; **Event:** samplingProtocol: Sweep net; eventDate: 2007-05-28; verbatimEventDate: 28/May/2007; **Record Level:** institutionCode: VPMC; basisOfRecord: PreservedSpecimen

#### Distribution

First record from Russia: RUC.

### Prionolabis cognata

(Lackschewitz, 1940)

8D4891CD-8B83-5C7A-87A1-46BDC99DA8D4

https://ccw.naturalis.nl/detail.php?id=7301

#### Materials

- **Type status:** Other material. **Occurrence:** occurrenceRemarks: 3 males; recordedBy: L.-P. Kolcsár; individualCount: 3; sex: male; preparations: Ethanol; occurrenceID: EU_LIM_782; **Taxon:** scientificName: Prionolabiscognata (Lackschewitz, 1940); family: Limoniidae; genus: Prionolabis; specificEpithet: cognata; scientificNameAuthorship: (Lackschewitz, 1940); **Location:** country: Montenegro; municipality: Šavnik; locality: Petnjica; verbatimElevation: 1057 m; minimumElevationInMeters: 1057; decimalLatitude: 42.9836; decimalLongitude: 19.0733; **Identification:** identifiedBy: L.-P. Kolcsár; **Event:** samplingProtocol: Sweep net; eventDate: 2014-04-30; verbatimEventDate: 30/Apr/2014; **Record Level:** institutionCode: CKLP; basisOfRecord: PreservedSpecimen
- **Type status:** Other material. **Occurrence:** occurrenceRemarks: 1 male; recordedBy: D.I. Gavryushin; individualCount: 1; sex: male; preparations: Ethanol; occurrenceID: EU_LIM_783; **Taxon:** scientificName: Prionolabiscognata (Lackschewitz, 1940); family: Limoniidae; genus: Prionolabis; specificEpithet: cognata; scientificNameAuthorship: (Lackschewitz, 1940); **Location:** country: Serbia; stateProvince: Zaječar; municipality: Knjaževac; locality: Crni Vrh; verbatimElevation: 800 m; minimumElevationInMeters: 800; decimalLatitude: 43.407; decimalLongitude: 22.587; **Identification:** identifiedBy: D.I. Gavryushin; **Event:** samplingProtocol: Sweep net; eventDate: 2015-06-01/2015-07-07; verbatimEventDate: 01-07/Jul/2015; **Record Level:** institutionCode: ZMMU; basisOfRecord: PreservedSpecimen
- **Type status:** Other material. **Occurrence:** occurrenceRemarks: 8 males; recordedBy: D.I. Gavryushin; individualCount: 8; sex: male; preparations: Pinned; occurrenceID: EU_LIM_784; **Taxon:** scientificName: Prionolabiscognata (Lackschewitz, 1940); family: Limoniidae; genus: Prionolabis; specificEpithet: cognata; scientificNameAuthorship: (Lackschewitz, 1940); **Location:** country: Serbia; locality: Stara Planina Mts.; verbatimElevation: 1500 m; minimumElevationInMeters: 1500; decimalLatitude: 43.37; decimalLongitude: 22.6; **Identification:** identifiedBy: D.I. Gavryushin; **Event:** samplingProtocol: Sweep net; eventDate: 2015-05-01/2015-05-08; verbatimEventDate: 01-08/May/2015; **Record Level:** institutionCode: ZMMU; basisOfRecord: PreservedSpecimen
- **Type status:** Other material. **Occurrence:** occurrenceRemarks: 1 male; recordedBy: D.I. Gavryushin; individualCount: 1; sex: male; preparations: Pinned; occurrenceID: EU_LIM_785; **Taxon:** scientificName: Prionolabiscognata (Lackschewitz, 1940); family: Limoniidae; genus: Prionolabis; specificEpithet: cognata; scientificNameAuthorship: (Lackschewitz, 1940); **Location:** country: Serbia; locality: Stara Planina Mts., Babin Zub Mountain; verbatimElevation: 1547 m; minimumElevationInMeters: 1547; decimalLatitude: 43.374; decimalLongitude: 22.621; **Identification:** identifiedBy: D.I. Gavryushin; **Event:** samplingProtocol: Sweep net; eventDate: 2015-06-03; verbatimEventDate: 3/Jul/2015; **Record Level:** institutionCode: ZMMU; basisOfRecord: PreservedSpecimen
- **Type status:** Other material. **Occurrence:** occurrenceRemarks: 1 male; recordedBy: D.I. Gavryushin; individualCount: 1; sex: male; preparations: Pinned; occurrenceID: EU_LIM_786; **Taxon:** scientificName: Prionolabiscognata (Lackschewitz, 1940); family: Limoniidae; genus: Prionolabis; specificEpithet: cognata; scientificNameAuthorship: (Lackschewitz, 1940); **Location:** country: Serbia; locality: Stara Planina Mts., Babin Zub Mountain; verbatimElevation: 1547 m; minimumElevationInMeters: 1547; decimalLatitude: 43.374; decimalLongitude: 22.621; **Identification:** identifiedBy: D.I. Gavryushin; **Event:** samplingProtocol: Sweep net; eventDate: 2015-06-06; verbatimEventDate: 6/Jul/2015; **Record Level:** institutionCode: ZMMU; basisOfRecord: PreservedSpecimen
- **Type status:** Other material. **Occurrence:** occurrenceRemarks: 21 male; recordedBy: D.I. Gavryushin; individualCount: 21; sex: male; preparations: Ethanol; occurrenceID: EU_LIM_787; **Taxon:** scientificName: Prionolabiscognata (Lackschewitz, 1940); family: Limoniidae; genus: Prionolabis; specificEpithet: cognata; scientificNameAuthorship: (Lackschewitz, 1940); **Location:** country: Serbia; locality: Stara Planina Mts.; verbatimElevation: 1030 m; minimumElevationInMeters: 1030; decimalLatitude: 43.396; decimalLongitude: 22.607; **Identification:** identifiedBy: D.I. Gavryushin; **Event:** samplingProtocol: Sweep net; eventDate: 2015-05-01/2015-05-08; verbatimEventDate: 01-08/May/2015; **Record Level:** institutionCode: ZMMU; basisOfRecord: PreservedSpecimen
- **Type status:** Other material. **Occurrence:** occurrenceRemarks: 19 males; recordedBy: D.I. Gavryushin; individualCount: 19; sex: male; preparations: Pinned; occurrenceID: EU_LIM_788; **Taxon:** scientificName: Prionolabiscognata (Lackschewitz, 1940); family: Limoniidae; genus: Prionolabis; specificEpithet: cognata; scientificNameAuthorship: (Lackschewitz, 1940); **Location:** country: Serbia; locality: Stara Planina Mts.; verbatimElevation: 1030 m; minimumElevationInMeters: 1030; decimalLatitude: 43.396; decimalLongitude: 22.607; **Identification:** identifiedBy: D.I. Gavryushin; **Event:** samplingProtocol: Sweep net; eventDate: 2015-05-01/2015-05-08; verbatimEventDate: 01-08/May/2015; **Record Level:** institutionCode: ZMMU; basisOfRecord: PreservedSpecimen

#### Description

Figs 17, 18

#### Distribution

First records from Montenegro and Serbia.

### Pseudolimnophila (Pseudolimnophila) lucorum

(Meigen, 1818)

64267114-6865-5ECD-8AFE-BA200FFA1EF4

https://ccw.naturalis.nl/detail.php?id=7418

#### Materials

- **Type status:** Other material. **Occurrence:** occurrenceRemarks: 1 female; recordedBy: L.-P. Kolcsár; individualCount: 1; sex: female; preparations: Ethanol; occurrenceID: EU_LIM_789; **Taxon:** scientificName: Pseudolimnophila (Pseudolimnophila) lucorum (Meigen, 1818); family: Limoniidae; genus: Pseudolimnophila; subgenus: Pseudolimnophila; specificEpithet: lucorum; scientificNameAuthorship: (Meigen, 1818); **Location:** country: Latvia; municipality: Skaistkalne; verbatimElevation: 12 m; minimumElevationInMeters: 12; decimalLatitude: 56.411; decimalLongitude: 24.637; **Identification:** identifiedBy: L.-P. Kolcsár; **Event:** samplingProtocol: Sweep net; eventDate: 2018-06-19; verbatimEventDate: 19/Jul/2018; habitat: birch-spruce forest, small stream; **Record Level:** institutionCode: CKLP; basisOfRecord: PreservedSpecimen
- **Type status:** Other material. **Occurrence:** catalogNumber: 558753, 644195; occurrenceRemarks: 3 male+female; recordedBy: K.M. Olsen; individualCount: 3; sex: male, female; preparations: Ethanol; occurrenceID: EU_LIM_790; **Taxon:** scientificName: Pseudolimnophila (Pseudolimnophila) lucorum (Meigen, 1818); family: Limoniidae; genus: Pseudolimnophila; subgenus: Pseudolimnophila; specificEpithet: lucorum; scientificNameAuthorship: (Meigen, 1818); **Location:** country: Norway; stateProvince: Aust-Agder; municipality: Arendal; locality: Hefte–Lille Gjerstadvann; verbatimElevation: 5 m; minimumElevationInMeters: 5; decimalLatitude: 58.46899; decimalLongitude: 8.85972; **Identification:** identifiedBy: K.M. Olsen; **Event:** samplingProtocol: Sweep net; eventDate: 2017-08-06; verbatimEventDate: 06/Aug/2017; **Record Level:** institutionCode: PCKMO; basisOfRecord: PreservedSpecimen
- **Type status:** Other material. **Occurrence:** catalogNumber: 590318; occurrenceRemarks: 2 male+female; recordedBy: K.M. Olsen; individualCount: 2; sex: male, female; preparations: Ethanol; occurrenceID: EU_LIM_791; **Taxon:** scientificName: Pseudolimnophila (Pseudolimnophila) lucorum (Meigen, 1818); family: Limoniidae; genus: Pseudolimnophila; subgenus: Pseudolimnophila; specificEpithet: lucorum; scientificNameAuthorship: (Meigen, 1818); **Location:** country: Norway; stateProvince: Østfold; municipality: Hvaler; locality: N Stuevika; verbatimElevation: 4 m; minimumElevationInMeters: 4; decimalLatitude: 59.01077; decimalLongitude: 11.08216; **Identification:** identifiedBy: K.M. Olsen; **Event:** samplingProtocol: Sweep net; eventDate: 2018-06-16; verbatimEventDate: 16/Jun/2018; **Record Level:** institutionCode: NHMO; basisOfRecord: PreservedSpecimen
- **Type status:** Other material. **Occurrence:** catalogNumber: 591140; occurrenceRemarks: 1 male; recordedBy: K.M. Olsen; individualCount: 1; sex: male; occurrenceID: EU_LIM_792; **Taxon:** scientificName: Pseudolimnophila (Pseudolimnophila) lucorum (Meigen, 1818); family: Limoniidae; genus: Pseudolimnophila; subgenus: Pseudolimnophila; specificEpithet: lucorum; scientificNameAuthorship: (Meigen, 1818); **Location:** country: Norway; stateProvince: Østfold; municipality: Hvaler; locality: Salta; verbatimElevation: 1 m; minimumElevationInMeters: 1; decimalLatitude: 59.01374; decimalLongitude: 11.06638; **Identification:** identifiedBy: K.M. Olsen; **Event:** samplingProtocol: Sweep net; eventDate: 2018-06-16; verbatimEventDate: 16/Jun/2018; habitat: E strandengområdet; **Record Level:** institutionCode: BioFokus; basisOfRecord: HumanObservation
- **Type status:** Other material. **Occurrence:** catalogNumber: 599047; occurrenceRemarks: 1 female; recordedBy: K. Berggren; individualCount: 1; sex: female; occurrenceID: EU_LIM_793; **Taxon:** scientificName: Pseudolimnophila (Pseudolimnophila) lucorum (Meigen, 1818); family: Limoniidae; genus: Pseudolimnophila; subgenus: Pseudolimnophila; specificEpithet: lucorum; scientificNameAuthorship: (Meigen, 1818); **Location:** country: Norway; stateProvince: Vest-Agder; municipality: Kristiansand; locality: Nedre Timenes; verbatimElevation: 10 m; minimumElevationInMeters: 10; decimalLatitude: 58.16155; decimalLongitude: 8.10013; **Identification:** identifiedBy: K.M. Olsen; **Event:** samplingProtocol: Light trap; eventDate: 2017-06; verbatimEventDate: Jul/2017; **Record Level:** institutionCode: BioFokus; basisOfRecord: HumanObservation
- **Type status:** Other material. **Occurrence:** catalogNumber: 600607; occurrenceRemarks: 2 male+female; recordedBy: K. Berggren; individualCount: 2; sex: male, female; preparations: Ethanol; occurrenceID: EU_LIM_794; **Taxon:** scientificName: Pseudolimnophila (Pseudolimnophila) lucorum (Meigen, 1818); family: Limoniidae; genus: Pseudolimnophila; subgenus: Pseudolimnophila; specificEpithet: lucorum; scientificNameAuthorship: (Meigen, 1818); **Location:** country: Norway; stateProvince: Vest-Agder; municipality: Kristiansand; locality: Bråvann terrasse – I hagen til nr. 21; verbatimElevation: 75 m; minimumElevationInMeters: 75; decimalLatitude: 58.11029; decimalLongitude: 7.93429; **Identification:** identifiedBy: K.M. Olsen; **Event:** samplingProtocol: Light trap; eventDate: 2017-08; verbatimEventDate: Aug/2017; **Record Level:** institutionCode: NHMO; basisOfRecord: PreservedSpecimen
- **Type status:** Other material. **Occurrence:** catalogNumber: 629208; occurrenceRemarks: 1 male; recordedBy: K. Berggren; individualCount: 1; sex: male; occurrenceID: EU_LIM_795; **Taxon:** scientificName: Pseudolimnophila (Pseudolimnophila) lucorum (Meigen, 1818); family: Limoniidae; genus: Pseudolimnophila; subgenus: Pseudolimnophila; specificEpithet: lucorum; scientificNameAuthorship: (Meigen, 1818); **Location:** country: Norway; stateProvince: Vest-Agder; municipality: Kristiansand; locality: Nedre Timenes; verbatimElevation: 10 m; minimumElevationInMeters: 10; decimalLatitude: 58.16155; decimalLongitude: 8.10013; **Identification:** identifiedBy: K.M. Olsen; **Event:** samplingProtocol: Light trap; eventDate: 2018-06-01/2018-07-11; verbatimEventDate: 01-11/Jul/2018; **Record Level:** institutionCode: BioFokus; basisOfRecord: HumanObservation
- **Type status:** Other material. **Occurrence:** catalogNumber: 593106; occurrenceRemarks: 1 female; recordedBy: K. Berggren; individualCount: 1; sex: female; preparations: Ethanol; occurrenceID: EU_LIM_796; **Taxon:** scientificName: Pseudolimnophila (Pseudolimnophila) lucorum (Meigen, 1818); family: Limoniidae; genus: Pseudolimnophila; subgenus: Pseudolimnophila; specificEpithet: lucorum; scientificNameAuthorship: (Meigen, 1818); **Location:** country: Norway; stateProvince: Vest-Agder; municipality: Kristiansand; locality: Nedre Timenes; verbatimElevation: 10 m; minimumElevationInMeters: 10; decimalLatitude: 58.16164; decimalLongitude: 8.09907; **Identification:** identifiedBy: K.M. Olsen; **Event:** samplingProtocol: Light trap; eventDate: 2017-06-17/2017-07-24; verbatimEventDate: 17-24/Jul/2017; **Record Level:** institutionCode: NHMO; basisOfRecord: PreservedSpecimen
- **Type status:** Other material. **Occurrence:** catalogNumber: 526255; occurrenceRemarks: 2 males; recordedBy: K.M. Olsen mfl.; individualCount: 2; sex: male; preparations: Ethanol; occurrenceID: EU_LIM_797; **Taxon:** scientificName: Pseudolimnophila (Pseudolimnophila) lucorum (Meigen, 1818); family: Limoniidae; genus: Pseudolimnophila; subgenus: Pseudolimnophila; specificEpithet: lucorum; scientificNameAuthorship: (Meigen, 1818); **Location:** country: Norway; stateProvince: Vestfold; municipality: Larvik; locality: NE Eineren; verbatimElevation: 8 m; minimumElevationInMeters: 8; decimalLatitude: 58.97812; decimalLongitude: 9.88454; **Identification:** identifiedBy: K.M. Olsen; **Event:** samplingProtocol: Sweep net; eventDate: 2017-06-08; verbatimEventDate: 08/Jun/2017; **Record Level:** institutionCode: PCKMO; basisOfRecord: PreservedSpecimen
- **Type status:** Other material. **Occurrence:** catalogNumber: 548921; occurrenceRemarks: 2 males; recordedBy: K.M. Olsen | S. Olberg; individualCount: 2; sex: male; preparations: Ethanol; occurrenceID: EU_LIM_798; **Taxon:** scientificName: Pseudolimnophila (Pseudolimnophila) lucorum (Meigen, 1818); family: Limoniidae; genus: Pseudolimnophila; subgenus: Pseudolimnophila; specificEpithet: lucorum; scientificNameAuthorship: (Meigen, 1818); **Location:** country: Norway; stateProvince: Vestfold; municipality: Larvik; locality: Sandvikbukta PFO; verbatimElevation: 2 m; minimumElevationInMeters: 2; decimalLatitude: 59.01169; decimalLongitude: 10.14128; **Identification:** identifiedBy: K.M. Olsen; **Event:** samplingProtocol: Sweep net; eventDate: 2017-06-06; verbatimEventDate: 06/Jun/2017; **Record Level:** institutionCode: NHMO; basisOfRecord: PreservedSpecimen
- **Type status:** Other material. **Occurrence:** catalogNumber: 584194; occurrenceRemarks: 1 male; recordedBy: S. Olberg; individualCount: 1; sex: male; preparations: Ethanol; occurrenceID: EU_LIM_799; **Taxon:** scientificName: Pseudolimnophila (Pseudolimnophila) lucorum (Meigen, 1818); family: Limoniidae; genus: Pseudolimnophila; subgenus: Pseudolimnophila; specificEpithet: lucorum; scientificNameAuthorship: (Meigen, 1818); **Location:** country: Norway; stateProvince: Vestfold; municipality: Tønsberg; locality: Gullkrona; verbatimElevation: 20 m; minimumElevationInMeters: 20; decimalLatitude: 59.28551; decimalLongitude: 10.38247; **Identification:** identifiedBy: K.M. Olsen; **Event:** samplingProtocol: Malaise trap; eventDate: 2018-06-15/2018-07-06; verbatimEventDate: 15/Jun-06/Jul/2018; **Record Level:** institutionCode: NHMO; basisOfRecord: PreservedSpecimen

#### Distribution

First records from Latvia and Norway.

### Pseudolimnophila (Pseudolimnophila) sepium

(Verrall, 1886)

91506B9D-4ABE-5BD3-93B5-D50DF2828D55

https://ccw.naturalis.nl/detail.php?id=7440

#### Materials

- **Type status:** Other material. **Occurrence:** occurrenceRemarks: 2 males; recordedBy: L.-P. Kolcsár; individualCount: 2; sex: male; preparations: Ethanol; occurrenceID: EU_LIM_800; **Taxon:** scientificName: Pseudolimnophila (Pseudolimnophila) sepium (Verrall, 1886); family: Limoniidae; genus: Pseudolimnophila; subgenus: Pseudolimnophila; specificEpithet: sepium; scientificNameAuthorship: (Verrall, 1886); **Location:** country: Latvia; municipality: Sigulda; locality: Gauja River; verbatimElevation: 13 m; minimumElevationInMeters: 13; decimalLatitude: 57.1505; decimalLongitude: 24.8168; **Identification:** identifiedBy: L.-P. Kolcsár; **Event:** samplingProtocol: Sweep net; eventDate: 2018-06-24; verbatimEventDate: 24/Jul/2018; **Record Level:** institutionCode: CKLP; basisOfRecord: PreservedSpecimen
- **Type status:** Other material. **Occurrence:** occurrenceRemarks: 1 male; recordedBy: E. Eiroa; individualCount: 1; sex: male; preparations: Pinned; occurrenceID: EU_LIM_801; **Taxon:** scientificName: Pseudolimnophila (Pseudolimnophila) sepium (Verrall, 1886); family: Limoniidae; genus: Pseudolimnophila; subgenus: Pseudolimnophila; specificEpithet: sepium; scientificNameAuthorship: (Verrall, 1886); **Location:** country: Spain; stateProvince: Asturias; municipality: Somiedo; locality: Pola de Somiedo, road to valle de Lago; verbatimElevation: 802 m; minimumElevationInMeters: 802; decimalLatitude: 43.08585; decimalLongitude: -6.24355; **Identification:** identifiedBy: E. Eiroa; **Event:** samplingProtocol: Sweep net; eventDate: 1993-06-16; verbatimEventDate: 16/June/1993; habitat: beech forest; **Record Level:** institutionCode: USC; basisOfRecord: PreservedSpecimen
- **Type status:** Other material. **Occurrence:** catalogNumber: MZB 2020-0840; occurrenceRemarks: 2 males; recordedBy: J. Mederos; individualCount: 2; sex: male; preparations: Ethanol; occurrenceID: EU_LIM_802; **Taxon:** scientificName: Pseudolimnophila (Pseudolimnophila) sepium (Verrall, 1886); family: Limoniidae; genus: Pseudolimnophila; subgenus: Pseudolimnophila; specificEpithet: sepium; scientificNameAuthorship: (Verrall, 1886); **Location:** country: Spain; stateProvince: Catalonia, Barcelona; municipality: Gualba; locality: Riera de Gualba, Montseny; verbatimElevation: 177 m; minimumElevationInMeters: 177; decimalLatitude: 41.73222; decimalLongitude: 2.50472; **Identification:** identifiedBy: J. Mederos; **Event:** samplingProtocol: Sweep net; eventDate: 2020-09-10; verbatimEventDate: 10/Sep/2020; **Record Level:** institutionCode: MCNB; basisOfRecord: PreservedSpecimen
- **Type status:** Other material. **Occurrence:** catalogNumber: MZB 2020-0841; occurrenceRemarks: 4 males; recordedBy: J. Mederos; individualCount: 4; sex: male; preparations: Ethanol; occurrenceID: EU_LIM_803; **Taxon:** scientificName: Pseudolimnophila (Pseudolimnophila) sepium (Verrall, 1886); family: Limoniidae; genus: Pseudolimnophila; subgenus: Pseudolimnophila; specificEpithet: sepium; scientificNameAuthorship: (Verrall, 1886); **Location:** country: Spain; stateProvince: Catalonia, Barcelona; municipality: Gualba; locality: Riera de Gualba, Montseny; verbatimElevation: 177 m; minimumElevationInMeters: 177; decimalLatitude: 41.73222; decimalLongitude: 2.50472; **Identification:** identifiedBy: J. Mederos; **Event:** samplingProtocol: Sweep net; eventDate: 2020-10-08; verbatimEventDate: 08/Oct/2020; **Record Level:** institutionCode: MCNB; basisOfRecord: PreservedSpecimen
- **Type status:** Other material. **Occurrence:** catalogNumber: MZB 2020-0842; occurrenceRemarks: 1 female; recordedBy: J. Mederos; individualCount: 1; sex: female; preparations: Pinned; occurrenceID: EU_LIM_804; **Taxon:** scientificName: Pseudolimnophila (Pseudolimnophila) sepium (Verrall, 1886); family: Limoniidae; genus: Pseudolimnophila; subgenus: Pseudolimnophila; specificEpithet: sepium; scientificNameAuthorship: (Verrall, 1886); **Location:** country: Spain; stateProvince: Catalonia, Barcelona; municipality: Gualba; locality: Riera de Gualba, Montseny; verbatimElevation: 177 m; minimumElevationInMeters: 177; decimalLatitude: 41.73222; decimalLongitude: 2.50472; **Identification:** identifiedBy: J. Mederos; **Event:** samplingProtocol: Sweep net; eventDate: 2020-10-08; verbatimEventDate: 08/Oct/2020; **Record Level:** institutionCode: MCNB; basisOfRecord: PreservedSpecimen
- **Type status:** Other material. **Occurrence:** catalogNumber: MZB 2020-0843; occurrenceRemarks: 1 male; recordedBy: J. Mederos; individualCount: 1; sex: male; preparations: Pinned; occurrenceID: EU_LIM_805; **Taxon:** scientificName: Pseudolimnophila (Pseudolimnophila) sepium (Verrall, 1886); family: Limoniidae; genus: Pseudolimnophila; subgenus: Pseudolimnophila; specificEpithet: sepium; scientificNameAuthorship: (Verrall, 1886); **Location:** country: Spain; stateProvince: Catalonia, Barcelona; municipality: Gualba; locality: Riera de Gualba, Montseny; verbatimElevation: 177 m; minimumElevationInMeters: 177; decimalLatitude: 41.73222; decimalLongitude: 2.50472; **Identification:** identifiedBy: J. Mederos; **Event:** samplingProtocol: Sweep net; eventDate: 2020-10-08; verbatimEventDate: 8/Oct/2020; **Record Level:** institutionCode: MCNB; basisOfRecord: PreservedSpecimen

#### Distribution

First records from Latvia and Spain (from mainland).

### Rhabdomastix (Rhabdomastix) edwardsi

Tjeder, 1967

E010D53D-5E4D-59E6-A5DA-1C4A3081AED5

https://ccw.naturalis.nl/detail.php?id=3861

#### Materials

- **Type status:** Other material. **Occurrence:** occurrenceRemarks: 1 male; recordedBy: L. Papp; individualCount: 1; sex: male; preparations: Pinned; occurrenceID: EU_LIM_806; **Taxon:** scientificName: Rhabdomastix (Rhabdomastix) edwardsi Tjeder, 1967; family: Limoniidae; genus: Rhabdomastix; subgenus: Rhabdomastix; specificEpithet: edwardsi; scientificNameAuthorship: Tjeder, 1967; **Location:** country: Hungary; stateProvince: Pest; municipality: Szokolya; locality: upper reach of Szén Stream; decimalLatitude: 47.9048; decimalLongitude: 18.9808; **Identification:** identifiedBy: L.-P. Kolcsár; **Event:** eventDate: 1999-05-15; verbatimEventDate: 15/May/1999; **Record Level:** institutionCode: HNHM; basisOfRecord: PreservedSpecimen
- **Type status:** Other material. **Occurrence:** occurrenceRemarks: 1 male; recordedBy: L.-P. Kolcsár; individualCount: 1; sex: male; preparations: Ethanol; occurrenceID: EU_LIM_807; **Taxon:** scientificName: Rhabdomastix (Rhabdomastix) edwardsi Tjeder, 1967; family: Limoniidae; genus: Rhabdomastix; subgenus: Rhabdomastix; specificEpithet: edwardsi; scientificNameAuthorship: Tjeder, 1967; **Location:** country: Montenegro; municipality: Zelenika; locality: Kotor Bay; verbatimElevation: 19 m; minimumElevationInMeters: 19; decimalLatitude: 42.45388; decimalLongitude: 18.56961; **Identification:** identifiedBy: J. Starý; **Event:** samplingProtocol: Sweep net; eventDate: 2010-05-14; verbatimEventDate: 14/May/2010; habitat: oak plantation; **Record Level:** institutionCode: PCJS; basisOfRecord: PreservedSpecimen
- **Type status:** Other material. **Occurrence:** occurrenceRemarks: 4 males; recordedBy: L.-P. Kolcsár; individualCount: 4; sex: male; preparations: Ethanol; occurrenceID: EU_LIM_808; **Taxon:** scientificName: Rhabdomastix (Rhabdomastix) edwardsi Tjeder, 1967; family: Limoniidae; genus: Rhabdomastix; subgenus: Rhabdomastix; specificEpithet: edwardsi; scientificNameAuthorship: Tjeder, 1967; **Location:** country: Montenegro; municipality: Morinj; verbatimElevation: 203 m; minimumElevationInMeters: 203; decimalLatitude: 42.48625; decimalLongitude: 18.61228; **Identification:** identifiedBy: J. Starý; **Event:** samplingProtocol: Sweep net; eventDate: 2010-05-13; verbatimEventDate: 13/May/2010; habitat: calcareous stream; **Record Level:** institutionCode: PCJS; basisOfRecord: PreservedSpecimen
- **Type status:** Other material. **Occurrence:** occurrenceRemarks: 7 males, 1 female; recordedBy: L.-P. Kolcsár; individualCount: 8; sex: male, female; preparations: Ethanol; occurrenceID: EU_LIM_809; **Taxon:** scientificName: Rhabdomastix (Rhabdomastix) edwardsi Tjeder, 1967; family: Limoniidae; genus: Rhabdomastix; subgenus: Rhabdomastix; specificEpithet: edwardsi; scientificNameAuthorship: Tjeder, 1967; **Location:** country: Montenegro; municipality: Morinj; locality: Morinj brook; verbatimElevation: 67 m; minimumElevationInMeters: 67; decimalLatitude: 42.48935; decimalLongitude: 18.62896; **Identification:** identifiedBy: J. Starý; **Event:** samplingProtocol: Sweep net; eventDate: 2010-05-13; verbatimEventDate: 13/May/2010; habitat: dry stream bed; **Record Level:** institutionCode: PCJS; basisOfRecord: PreservedSpecimen
- **Type status:** Other material. **Occurrence:** occurrenceRemarks: 1 male; recordedBy: L.-P. Kolcsár | E. Török; individualCount: 1; sex: male; preparations: Ethanol; occurrenceID: EU_LIM_810; **Taxon:** scientificName: Rhabdomastix (Rhabdomastix) edwardsi Tjeder, 1967; family: Limoniidae; genus: Rhabdomastix; subgenus: Rhabdomastix; specificEpithet: edwardsi; scientificNameAuthorship: Tjeder, 1967; **Location:** country: Serbia; municipality: Sikirje; locality: Kukavica Mts.; verbatimElevation: 648 m; minimumElevationInMeters: 648; decimalLatitude: 42.65259; decimalLongitude: 21.88019; **Identification:** identifiedBy: L.-P. Kolcsár; **Event:** samplingProtocol: Sweep net; eventDate: 2017-06-02; verbatimEventDate: 2/Jul/2017; **Record Level:** institutionCode: CKLP; basisOfRecord: PreservedSpecimen

#### Distribution

First records from Hungary, Montenegro and Serbia.

### Rhabdomastix (Rhabdomastix) filata

Starý, 2004

9E37AC13-07AE-577D-9A11-29F564BDD8FD

https://ccw.naturalis.nl/detail.php?id=3866

#### Materials

- **Type status:** Other material. **Occurrence:** occurrenceRemarks: 1 male; recordedBy: L.-P. Kolcsár; individualCount: 1; sex: male; preparations: Ethanol; occurrenceID: EU_LIM_811; **Taxon:** scientificName: Rhabdomastix (Rhabdomastix) filata Starý, 2004; family: Limoniidae; genus: Rhabdomastix; subgenus: Rhabdomastix; specificEpithet: filata; scientificNameAuthorship: Starý, 2004; **Location:** country: Romania; stateProvince: Harghita; municipality: Izvoare; locality: Harghita Mts., Ivó Stream; verbatimElevation: 877 m; minimumElevationInMeters: 877; decimalLatitude: 46.46548; decimalLongitude: 25.49882; **Identification:** identifiedBy: J. Starý; **Event:** eventDate: 2013-06-10; verbatimEventDate: 10/Jul/2013; **Record Level:** institutionCode: PCJS; basisOfRecord: PreservedSpecimen

#### Distribution

First record from Romania.

### Rhabdomastix (Rhabdomastix) hirticornis

(Lackschewitz, 1940)

CC18066C-2B26-518E-9742-7F6D2299BBDF

https://ccw.naturalis.nl/detail.php?id=3876

#### Materials

- **Type status:** Other material. **Occurrence:** occurrenceRemarks: 1 male; recordedBy: D.I. Gavryushin; individualCount: 1; sex: male; preparations: Ethanol; occurrenceID: EU_LIM_812; **Taxon:** scientificName: Rhabdomastix (Rhabdomastix) hirticornis (Lackschewitz, 1940); family: Limoniidae; genus: Rhabdomastix; subgenus: Rhabdomastix; specificEpithet: hirticornis; scientificNameAuthorship: (Lackschewitz, 1940); **Location:** country: Serbia; stateProvince: Zaječar; municipality: Knjaževac; locality: Crni Vrh; verbatimElevation: 800 m; minimumElevationInMeters: 800; decimalLatitude: 43.407; decimalLongitude: 22.587; **Identification:** identifiedBy: D.I. Gavryushin; **Event:** samplingProtocol: Sweep net; eventDate: 2015-06-01/2015-07-07; verbatimEventDate: 01-07/Jul/2015; **Record Level:** institutionCode: ZMMU; basisOfRecord: PreservedSpecimen

#### Distribution

First record from Serbia.

### Rhabdomastix (Rhabdomastix) japonica

Alexander, 1924

E11C1999-D679-533E-8128-B13AC017FA38

https://ccw.naturalis.nl/detail.php?id=3887

#### Materials

- **Type status:** Other material. **Occurrence:** catalogNumber: THS-20160001; occurrenceRemarks: 1 male; recordedBy: J. Salmela; individualCount: 1; sex: male; preparations: Ethanol; occurrenceID: EU_LIM_813; **Taxon:** scientificName: Rhabdomastix (Rhabdomastix) japonica Alexander, 1924; family: Limoniidae; genus: Rhabdomastix; subgenus: Rhabdomastix; specificEpithet: japonica; scientificNameAuthorship: Alexander, 1924; **Location:** country: Finland; stateProvince: Lapponia inariensis; municipality: Utsjoki; locality: Karigasniemi; verbatimElevation: 120 m; minimumElevationInMeters: 120; decimalLatitude: 69.369; decimalLongitude: 25.824; **Identification:** identifiedBy: J. Salmela; **Event:** samplingProtocol: Sweep net; eventDate: 2016-06-11; verbatimEventDate: 11/Jul/2016; **Record Level:** institutionCode: LMM; basisOfRecord: PreservedSpecimen
- **Type status:** Other material. **Occurrence:** catalogNumber: 666268; occurrenceRemarks: 4 male+female; recordedBy: K.M. Olsen; individualCount: 4; sex: male, female; preparations: Ethanol; occurrenceID: EU_LIM_814; **Taxon:** scientificName: Rhabdomastix (Rhabdomastix) japonica Alexander, 1924; family: Limoniidae; genus: Rhabdomastix; subgenus: Rhabdomastix; specificEpithet: japonica; scientificNameAuthorship: Alexander, 1924; **Location:** country: Norway; stateProvince: Finnmark; municipality: Tana; locality: Lismajoki–Nuorinjálbmi; verbatimElevation: 10 m; minimumElevationInMeters: 10; decimalLatitude: 70.14961; decimalLongitude: 28.18817; **Identification:** identifiedBy: K.M. Olsen | J. Starý; **Event:** samplingProtocol: Sweep net; eventDate: 2020-06-08; verbatimEventDate: 08/Jul/2020; **Record Level:** institutionCode: PCKMO; basisOfRecord: PreservedSpecimen

#### Distribution

First records from Finland and Norway.

### Rhabdomastix (Rhabdomastix) laeta

(Loew, 1873)

6D837872-513C-52A4-A881-DE3195B9B1DE

https://ccw.naturalis.nl/detail.php?id=3888

#### Materials

- **Type status:** Other material. **Occurrence:** catalogNumber: 559662; occurrenceRemarks: 2 males; recordedBy: K.M. Olsen; individualCount: 2; sex: male; preparations: Ethanol; occurrenceID: EU_LIM_815; **Taxon:** scientificName: Rhabdomastix (Rhabdomastix) laeta (Loew, 1873); family: Limoniidae; genus: Rhabdomastix; subgenus: Rhabdomastix; specificEpithet: laeta; scientificNameAuthorship: (Loew, 1873); **Location:** country: Norway; stateProvince: Akershus; municipality: Skedsmo; locality: N Asak Mellom; verbatimElevation: 150 m; minimumElevationInMeters: 150; decimalLatitude: 59.98719; decimalLongitude: 11.10035; **Identification:** identifiedBy: K.M. Olsen; **Event:** samplingProtocol: Malaise trap; eventDate: 2017-06-20/2017-07-26; verbatimEventDate: 20/Jun-26/Jul/2017; **Record Level:** institutionCode: PCKMO; basisOfRecord: PreservedSpecimen
- **Type status:** Other material. **Occurrence:** catalogNumber: 535566; occurrenceRemarks: 1 male; recordedBy: K.M. Olsen; individualCount: 1; sex: male; preparations: Ethanol; occurrenceID: EU_LIM_816; **Taxon:** scientificName: Rhabdomastix (Rhabdomastix) laeta (Loew, 1873); family: Limoniidae; genus: Rhabdomastix; subgenus: Rhabdomastix; specificEpithet: laeta; scientificNameAuthorship: (Loew, 1873); **Location:** country: Norway; stateProvince: Akershus; municipality: Skedsmo; locality: SSE Buhaugen; verbatimElevation: 110 m; minimumElevationInMeters: 110; decimalLatitude: 59.97165; decimalLongitude: 10.98637; **Identification:** identifiedBy: K.M. Olsen; **Event:** samplingProtocol: Malaise trap; eventDate: 2017-06-30/2017-09-07; verbatimEventDate: 31/Jul-07/Sep/2017; **Record Level:** institutionCode: PCKMO; basisOfRecord: PreservedSpecimen

#### Distribution

First records from Norway.

### Rhabdomastix (Rhabdomastix) laetoidea

Starý, 2004

0FC5147F-9DF4-5855-A364-158DD03CB440

https://ccw.naturalis.nl/detail.php?id=3889

#### Materials

- **Type status:** Other material. **Occurrence:** occurrenceRemarks: 2 males; recordedBy: L.-P. Kolcsár; individualCount: 2; sex: male; preparations: Ethanol; occurrenceID: EU_LIM_817; **Taxon:** scientificName: Rhabdomastix (Rhabdomastix) laetoidea Starý, 2004; family: Limoniidae; genus: Rhabdomastix; subgenus: Rhabdomastix; specificEpithet: laetoidea; scientificNameAuthorship: Starý, 2004; **Location:** country: Romania; stateProvince: Harghita; municipality: Voșlăbeni; locality: Senetea; verbatimElevation: 764 m; minimumElevationInMeters: 764; decimalLatitude: 46.62588; decimalLongitude: 25.59745; **Identification:** identifiedBy: J. Starý; **Event:** samplingProtocol: Sweep net; eventDate: 2013-06-09; verbatimEventDate: 9/Jul/2013; habitat: marshy meadow; **Record Level:** institutionCode: PCJS; basisOfRecord: PreservedSpecimen

#### Distribution

First records from Romania.

### Rhabdomastix (Rhabdomastix) subparva

Starý, 1971

E8814848-AC2E-5311-8636-64030938A211

https://ccw.naturalis.nl/detail.php?id=3938

#### Materials

- **Type status:** Other material. **Occurrence:** occurrenceRemarks: 1 male; recordedBy: L.-P. Kolcsár | E. Török; individualCount: 1; sex: male; preparations: Ethanol; occurrenceID: EU_LIM_818; **Taxon:** scientificName: Rhabdomastix (Rhabdomastix) subparva Starý, 1971; family: Limoniidae; genus: Rhabdomastix; subgenus: Rhabdomastix; specificEpithet: subparva; scientificNameAuthorship: Starý, 1971; **Location:** country: Serbia; municipality: Kopaonik; locality: Kopaonik Mts.; verbatimElevation: 1556 m; minimumElevationInMeters: 1556; decimalLatitude: 43.30997; decimalLongitude: 20.76563; **Identification:** identifiedBy: L.-P. Kolcsár; **Event:** samplingProtocol: Sweep net; eventDate: 2017-06-22; verbatimEventDate: 22/Jun/2017; **Record Level:** institutionCode: CKLP; basisOfRecord: PreservedSpecimen

#### Distribution

Here, we confirm the presence of the species in Serbia.

### Rhipidia (Rhipidia) ctenophora

Loew, 1871

A4AADFCD-4AB8-5F16-B859-8981E6E0906F

https://ccw.naturalis.nl/detail.php?id=11135

#### Materials

- **Type status:** Other material. **Occurrence:** occurrenceRemarks: 1 male; recordedBy: J. Martinovský; individualCount: 1; sex: male; preparations: Pinned; occurrenceID: EU_LIM_819; **Taxon:** scientificName: Rhipidia (Rhipidia) ctenophora Loew, 1871; family: Limoniidae; genus: Rhipidia; subgenus: Rhipidia; specificEpithet: ctenophora; scientificNameAuthorship: Loew, 1871; **Location:** country: Greece; stateProvince: Thessalia; municipality: Agricampos; locality: 2 km SW; decimalLatitude: 39.637; decimalLongitude: 22.878; **Identification:** identifiedBy: J. Starý; **Event:** eventDate: 1994-06-08; verbatimEventDate: 8/Jun/1994; **Record Level:** institutionCode: PCJS; basisOfRecord: PreservedSpecimen
- **Type status:** Other material. **Occurrence:** occurrenceRemarks: 1 female; recordedBy: J. Martinovský; individualCount: 1; sex: female; preparations: Pinned; occurrenceID: EU_LIM_820; **Taxon:** scientificName: Rhipidia (Rhipidia) ctenophora Loew, 1871; family: Limoniidae; genus: Rhipidia; subgenus: Rhipidia; specificEpithet: ctenophora; scientificNameAuthorship: Loew, 1871; **Location:** country: Italy; stateProvince: Calabria; municipality: Santa Maria del Cedro; locality: Lao Valley; decimalLatitude: 39.754; decimalLongitude: 15.842; **Identification:** identifiedBy: J. Starý; **Event:** eventDate: 1996-06-02; verbatimEventDate: 2/Jun/1996; **Record Level:** institutionCode: PCJS; basisOfRecord: PreservedSpecimen

#### Distribution

First records from Greece (from mainland). First record from the mainland of Italy, previously known from Sicily.

### Rhipidia (Rhipidia) maculata

Meigen, 1818

1F04F8CC-04D0-50B7-8191-338ADEE7B660

https://ccw.naturalis.nl/detail.php?id=11203

#### Materials

- **Type status:** Other material. **Occurrence:** occurrenceRemarks: 2 males; recordedBy: D.I. Gavryushin; individualCount: 2; sex: male; preparations: Pinned; occurrenceID: EU_LIM_821; **Taxon:** scientificName: Rhipidia (Rhipidia) maculata Meigen, 1818; family: Limoniidae; genus: Rhipidia; subgenus: Rhipidia; specificEpithet: maculata; scientificNameAuthorship: Meigen, 1818; **Location:** country: Belarus; stateProvince: Minsk; county: Barysaw; locality: Barysaw; verbatimElevation: 155 m; minimumElevationInMeters: 155; decimalLatitude: 54.25542; decimalLongitude: 28.48092; **Identification:** identifiedBy: D.I. Gavryushin; **Event:** samplingProtocol: Sweep net; eventDate: 2013-06-06; verbatimEventDate: 6/Jul/2013; **Record Level:** institutionCode: ZMMU; basisOfRecord: PreservedSpecimen
- **Type status:** Other material. **Occurrence:** occurrenceRemarks: 1 male, 5 females; recordedBy: D.I. Gavryushin; individualCount: 6; sex: male, female; occurrenceID: EU_LIM_822; **Taxon:** scientificName: Rhipidia (Rhipidia) maculata Meigen, 1818; family: Limoniidae; genus: Rhipidia; subgenus: Rhipidia; specificEpithet: maculata; scientificNameAuthorship: Meigen, 1818; **Location:** country: Russia; stateProvince: East European Russia; county: Bashkortostan Respublika; municipality: Beloretsk district; locality: Nura River (ca. 4km W of Otnurok village), at the foot of Zolotyie Shishki (Golden Cones) Mts.; verbatimElevation: 607 m; minimumElevationInMeters: 607; decimalLatitude: 54.05155; decimalLongitude: 58.26887; **Identification:** identifiedBy: D.I. Gavryushin; **Event:** samplingProtocol: Sweep net; eventDate: 2012-08-09; verbatimEventDate: 09/Aug/2012; **Record Level:** institutionCode: ZMMU; basisOfRecord: PreservedSpecimen
- **Type status:** Other material. **Occurrence:** occurrenceRemarks: 1 male, 1 female; recordedBy: D.I. Gavryushin; individualCount: 2; sex: male, female; occurrenceID: EU_LIM_823; **Taxon:** scientificName: Rhipidia (Rhipidia) maculata Meigen, 1818; family: Limoniidae; genus: Rhipidia; subgenus: Rhipidia; specificEpithet: maculata; scientificNameAuthorship: Meigen, 1818; **Location:** country: Russia; stateProvince: East European Russia; county: Bashkortostan Respublika; municipality: Beloretsk district; locality: Nura River (ca. 4km W of Otnurok village), at the foot of Zolotyie Shishki (Golden Cones) Mts.; verbatimElevation: 607 m; minimumElevationInMeters: 607; decimalLatitude: 54.05155; decimalLongitude: 58.26887; **Identification:** identifiedBy: D.I. Gavryushin; **Event:** samplingProtocol: Sweep net; eventDate: 2015-06-13; verbatimEventDate: 13/Jul/2015; **Record Level:** institutionCode: ZMMU; basisOfRecord: PreservedSpecimen
- **Type status:** Other material. **Occurrence:** occurrenceRemarks: 1 female; recordedBy: D.I. Gavryushin; individualCount: 1; sex: female; occurrenceID: EU_LIM_824; **Taxon:** scientificName: Rhipidia (Rhipidia) maculata Meigen, 1818; family: Limoniidae; genus: Rhipidia; subgenus: Rhipidia; specificEpithet: maculata; scientificNameAuthorship: Meigen, 1818; **Location:** country: Russia; stateProvince: East European Russia; county: Bashkortostan Respublika; municipality: Beloretsk district; locality: Makhmutovo env., Belaya River; verbatimElevation: 550 m; minimumElevationInMeters: 550; decimalLatitude: 54.33012; decimalLongitude: 58.80735; **Identification:** identifiedBy: D.I. Gavryushin; **Event:** samplingProtocol: Sweep net; eventDate: 2015-06-15; verbatimEventDate: 15/Jul/2015; **Record Level:** institutionCode: ZMMU; basisOfRecord: PreservedSpecimen

#### Distribution

First records from Belarus and Russia: RUE.

### Rhipidia (Rhipidia) punctiplena

Mik, 1887

8A389156-BE5C-5181-95C8-C32D58DC54DA

https://ccw.naturalis.nl/detail.php?id=11260

#### Materials

- **Type status:** Other material. **Occurrence:** occurrenceRemarks: 1 female; recordedBy: G.B. Delmastro; individualCount: 1; sex: female; preparations: Pinned; occurrenceID: EU_LIM_825; **Taxon:** scientificName: Rhipidia (Rhipidia) punctiplena Mik, 1887; family: Limoniidae; genus: Rhipidia; subgenus: Rhipidia; specificEpithet: punctiplena; scientificNameAuthorship: Mik, 1887; **Location:** country: Italy; stateProvince: Piedmont; municipality: Parella; locality: Peronetto; verbatimElevation: 250 m; minimumElevationInMeters: 250; decimalLatitude: 45.419; decimalLongitude: 7.798; **Identification:** identifiedBy: J. Starý; **Event:** eventDate: 2000-05-17; verbatimEventDate: 17/May/2000; **Record Level:** institutionCode: PCJS; basisOfRecord: PreservedSpecimen

#### Distribution

First record from Italy (from mainland).

### Rhipidia (Rhipidia) uniseriata uniseriata

Schiner, 1864

E3DDB496-0CAD-5DEA-8B05-94C92E9B036C

https://ccw.naturalis.nl/detail.php?id=11306

#### Materials

- **Type status:** Other material. **Occurrence:** occurrenceRemarks: 2 males; recordedBy: L.-P. Kolcsár, E. Török; individualCount: 2; sex: male; preparations: ethanol; occurrenceID: EU_LIM_810; **Taxon:** scientificName: Rhipidia (Rhipidia) uniseriata uniseriata Schiner, 1864; family: Limoniidae; genus: Rhipidia; subgenus: Rhipidia; specificEpithet: uniseriata; infraspecificEpithet: uniseriata; scientificNameAuthorship: Schiner, 1864; **Location:** country: North Macedonia; municipality: Novo Selo; locality: Bistra Mts., Marlovo NP.; verbatimElevation: 990 m; minimumElevationInMeters: 990; decimalLatitude: 41.719438; decimalLongitude: 20.82889; **Identification:** identifiedBy: L.-P. Kolcsár; **Event:** samplingProtocol: sweep net; eventDate: 2017-06-29; verbatimEventDate: 29/Jun/2017; **Record Level:** institutionCode: CKLP; basisOfRecord: PreservedSpecimen
- **Type status:** Other material. **Occurrence:** occurrenceRemarks: 1 female; recordedBy: N.M. Paramonov; individualCount: 1; sex: female; occurrenceID: EU_LIM_811; **Taxon:** scientificName: Rhipidia (Rhipidia) uniseriata uniseriata Schiner, 1864; family: Limoniidae; genus: Rhipidia; subgenus: Rhipidia; specificEpithet: uniseriata; infraspecificEpithet: uniseriata; scientificNameAuthorship: Schiner, 1864; **Location:** country: Russia; stateProvince: East European Russia; county: Tatarstan Respublika; municipality: Laishevo district; locality: Volga-Kama State Nature Biosphere Reserve, «Saraly», island Ornitologicheskiy; verbatimElevation: 50 m; minimumElevationInMeters: 50; decimalLatitude: 55.283916; decimalLongitude: 49.260808; **Identification:** identifiedBy: N.M. Paramonov; **Event:** samplingProtocol: sweep net; eventDate: 2009-06-23; verbatimEventDate: 23/Jun/2009; **Record Level:** institutionCode: ZIN; basisOfRecord: PreservedSpecimen
- **Type status:** Other material. **Occurrence:** occurrenceRemarks: 1 male; recordedBy: N.M. Paramonov; individualCount: 1; sex: male; occurrenceID: EU_LIM_812; **Taxon:** scientificName: Rhipidia (Rhipidia) uniseriata uniseriata Schiner, 1864; family: Limoniidae; genus: Rhipidia; subgenus: Rhipidia; specificEpithet: uniseriata; infraspecificEpithet: uniseriata; scientificNameAuthorship: Schiner, 1864; **Location:** country: Russia; stateProvince: East European Russia; county: Tatarstan Respublika; municipality: Zelenodol’sk district; locality: Ilinskoe village env; verbatimElevation: 90 m; minimumElevationInMeters: 90; decimalLatitude: 55.874548; decimalLongitude: 48.685785; **Identification:** identifiedBy: N.M. Paramonov; **Event:** samplingProtocol: sweep net; eventDate: 2009-06-12; verbatimEventDate: 12/Jun/2009; **Record Level:** institutionCode: ZIN; basisOfRecord: PreservedSpecimen
- **Type status:** Other material. **Occurrence:** occurrenceRemarks: 2 females; recordedBy: N.M. Paramonov; individualCount: 2; sex: female; occurrenceID: EU_LIM_813; **Taxon:** scientificName: Rhipidia (Rhipidia) uniseriata uniseriata Schiner, 1864; family: Limoniidae; genus: Rhipidia; subgenus: Rhipidia; specificEpithet: uniseriata; infraspecificEpithet: uniseriata; scientificNameAuthorship: Schiner, 1864; **Location:** country: Russia; stateProvince: East European Russia; county: Tatarstan Respublika; municipality: Zelenodol’sk district; locality: Volga-Kama State Nature Biosphere Reserve, «Raifa», biostation Raifa; verbatimElevation: 100 m; minimumElevationInMeters: 100; decimalLatitude: 55.88868; decimalLongitude: 48.71434; **Identification:** identifiedBy: N.M. Paramonov; **Event:** samplingProtocol: sweep net; eventDate: 2009-06-12; verbatimEventDate: 12/Jun/2009; **Record Level:** institutionCode: ZIN; basisOfRecord: PreservedSpecimen

#### Distribution

First records from North Macedonia and Russia: RUE.

### Rhypholophus bifurcatus

Goetghebuer, 1920

3971D164-6E62-5941-AA36-E69ED8D7DF0F

https://ccw.naturalis.nl/detail.php?id=3963

#### Materials

- **Type status:** Other material. **Occurrence:** occurrenceRemarks: 1 male, 3 females; recordedBy: D.I. Gavryushin; individualCount: 1; sex: female; preparations: Pinned; occurrenceID: EU_LIM_830; **Taxon:** scientificName: Rhypholophusbifurcatus Goetghebuer, 1920; family: Limoniidae; genus: Rhypholophus; specificEpithet: bifurcatus; scientificNameAuthorship: Goetghebuer, 1920; **Location:** country: Serbia; stateProvince: Zaječar; municipality: Knjaževac; locality: Crni Vrh; verbatimElevation: 800 m; minimumElevationInMeters: 800; decimalLatitude: 43.407; decimalLongitude: 22.587; **Identification:** identifiedBy: D.I. Gavryushin; **Event:** samplingProtocol: Sweep net; eventDate: 2014-09-16/2014-09-18; verbatimEventDate: 16-22/Sep/2014; **Record Level:** institutionCode: ZMMU; basisOfRecord: PreservedSpecimen

#### Distribution

First record from Serbia.

Records of *Rhypholophusvarius* (Meigen and Wiedemann, 1818) from Hungary reported by Ujvárosi (2004) are misidentified – material deposited in HNHM belongs to *Rhypholophusbifurcatus.* We, therefore, delete the species from the Hungarian checklist.

### Rhypholophus haemorrhoidalis

(Zetterstedt, 1838)

24CD5C89-7007-54CC-A7DC-DEC618B635CD

https://ccw.naturalis.nl/detail.php?id=3968

#### Materials

- **Type status:** Other material. **Occurrence:** occurrenceRemarks: 1 male; recordedBy: D.I. Gavryushin; individualCount: 1; sex: male; occurrenceID: EU_LIM_831; **Taxon:** scientificName: Rhypholophushaemorrhoidalis (Zetterstedt, 1838); family: Limoniidae; genus: Rhypholophus; specificEpithet: haemorrhoidalis; scientificNameAuthorship: (Zetterstedt, 1838); **Location:** country: Russia; stateProvince: East European Russia; county: Bashkortostan Respublika; municipality: Beloretsk district; locality: Nura River (ca. 4km W of Otnurok village), at the foot of Zolotyie Shishki (Golden Cones) Mts.; verbatimElevation: 607 m; minimumElevationInMeters: 607; decimalLatitude: 54.05155; decimalLongitude: 58.26887; **Identification:** identifiedBy: D.I. Gavryushin; **Event:** samplingProtocol: Sweep net; eventDate: 2012-08-08; verbatimEventDate: 08/Aug/2012; **Record Level:** institutionCode: ZMMU; basisOfRecord: PreservedSpecimen
- **Type status:** Other material. **Occurrence:** occurrenceRemarks: 1 male; recordedBy: D.I. Gavryushin; individualCount: 1; sex: male; occurrenceID: EU_LIM_832; **Taxon:** scientificName: Rhypholophushaemorrhoidalis (Zetterstedt, 1838); family: Limoniidae; genus: Rhypholophus; specificEpithet: haemorrhoidalis; scientificNameAuthorship: (Zetterstedt, 1838); **Location:** country: Russia; stateProvince: East European Russia; county: Bashkortostan Respublika; municipality: Beloretsk district; locality: Nura River (ca. 4km W of Otnurok village), at the foot of Zolotyie Shishki (Golden Cones) Mts.; verbatimElevation: 607 m; minimumElevationInMeters: 607; decimalLatitude: 54.05155; decimalLongitude: 58.26887; **Identification:** identifiedBy: D.I. Gavryushin; **Event:** samplingProtocol: Sweep net; eventDate: 2012-08-11; verbatimEventDate: 11/Aug/2012; **Record Level:** institutionCode: ZMMU; basisOfRecord: PreservedSpecimen
- **Type status:** Other material. **Occurrence:** occurrenceRemarks: 11 males, 12 females; recordedBy: N.M. Paramonov; individualCount: 23; sex: male, female; occurrenceID: EU_LIM_833; **Taxon:** scientificName: Rhypholophushaemorrhoidalis (Zetterstedt, 1838); family: Limoniidae; genus: Rhypholophus; specificEpithet: haemorrhoidalis; scientificNameAuthorship: (Zetterstedt, 1838); **Location:** country: Russia; stateProvince: East European Russia; county: Tatarstan Respublika; municipality: Verhneuslonsk district; locality: base “Zoostation”, 3,5 km NW Pustye Morkvashi env.; verbatimElevation: 80 m; minimumElevationInMeters: 80; decimalLatitude: 55.47005; decimalLongitude: 48.44092; **Identification:** identifiedBy: N.M. Paramonov; **Event:** samplingProtocol: Sweep net; eventDate: 2013-08-22/2013-08-26; verbatimEventDate: 22-26/Aug/2013; habitat: ravine, wetland; **Record Level:** institutionCode: ZIN; basisOfRecord: PreservedSpecimen

#### Distribution

First records from Russia: RUE.

### Scleroprocta pentagonalis

(Loew, 1873)

726998EC-4985-59C1-B4C0-5B3D6D81BCD8

https://ccw.naturalis.nl/detail.php?id=4002

#### Materials

- **Type status:** Other material. **Occurrence:** occurrenceRemarks: 2 males, 1 female; recordedBy: L.-P. Kolcsár | E. Török; individualCount: 3; sex: male, female; preparations: Ethanol; occurrenceID: EU_LIM_834; **Taxon:** scientificName: Scleroproctapentagonalis (Loew, 1873); family: Limoniidae; genus: Scleroprocta; specificEpithet: pentagonalis; scientificNameAuthorship: (Loew, 1873); **Location:** country: Serbia; municipality: Sikirje; locality: Kukavica Mts.; verbatimElevation: 648 m; minimumElevationInMeters: 648; decimalLatitude: 42.65259; decimalLongitude: 21.88019; **Identification:** identifiedBy: L.-P. Kolcsár; **Event:** samplingProtocol: Sweep net; eventDate: 2017-06-02; verbatimEventDate: 2/Jul/2017; **Record Level:** institutionCode: CKLP; basisOfRecord: PreservedSpecimen

#### Distribution

First record from Serbia.

### Symplecta (Symplecta) chosenensis

(Alexander, 1940)

D90DEE86-5186-5B8B-9D15-7AF50FB8A7A6

https://ccw.naturalis.nl/detail.php?id=4285

#### Materials

- **Type status:** Other material. **Occurrence:** occurrenceRemarks: 1 male; recordedBy: H. Siebke; individualCount: 1; sex: male; preparations: Pinned; occurrenceID: EU_LIM_839; **Taxon:** scientificName: Symplecta (Symplecta) chosenensis (Alexander, 1940); family: Limoniidae; genus: Symplecta; subgenus: Symplecta; specificEpithet: chosenensis; scientificNameAuthorship: (Alexander, 1940); **Location:** country: Norway; stateProvince: Oslo; municipality: Oslo; locality: Youngs Løkke; verbatimElevation: 5 m; minimumElevationInMeters: 5; decimalLatitude: 59.91; decimalLongitude: 10.75; **Identification:** identifiedBy: K.M. Olsen | J. Salmella | Ř. Gammelmo; **Event:** eventDate: 1849-06-06; verbatimEventDate: 06/Jul/1849; **Record Level:** institutionCode: NHMO; basisOfRecord: PreservedSpecimen
- **Type status:** Other material. **Occurrence:** occurrenceRemarks: 1 male; recordedBy: D.I. Gavryushin; individualCount: 1; sex: male; occurrenceID: EU_LIM_840; **Taxon:** scientificName: Symplecta (Symplecta) chosenensis (Alexander, 1940); family: Limoniidae; genus: Symplecta; subgenus: Symplecta; specificEpithet: chosenensis; scientificNameAuthorship: (Alexander, 1940); **Location:** country: Russia; stateProvince: East European Russia; county: Bashkortostan Respublika; municipality: Uchaly district; locality: Kazakkulovo village env., near a bridge across the Mindyak River; verbatimElevation: 507 m; minimumElevationInMeters: 507; decimalLatitude: 53.96306; decimalLongitude: 58.76534; **Identification:** identifiedBy: D.I. Gavryushin; **Event:** samplingProtocol: Sweep net; eventDate: 2012-08-12; verbatimEventDate: 12/Aug/2012; **Record Level:** institutionCode: ZMMU; basisOfRecord: PreservedSpecimen

#### Distribution

First records from Norway and Russia: RUE.

### Symplecta (Symplecta) hybrida

(Meigen, 1804)

76A5AFF6-4997-5455-98F2-0C6CC86457DF

https://ccw.naturalis.nl/detail.php?id=4292

#### Materials

- **Type status:** Other material. **Occurrence:** occurrenceRemarks: 1 male; recordedBy: D.I. Gavryushin; individualCount: 1; sex: male; preparations: Pinned; occurrenceID: EU_LIM_841; **Taxon:** scientificName: Symplecta (Symplecta) hybrida (Meigen, 1804); family: Limoniidae; genus: Symplecta; subgenus: Symplecta; specificEpithet: hybrida; scientificNameAuthorship: (Meigen, 1804); **Location:** country: Belarus; stateProvince: Minsk; county: Barysaw; locality: Glivin; verbatimElevation: 161 m; minimumElevationInMeters: 161; decimalLatitude: 54.14902; decimalLongitude: 28.63648; **Identification:** identifiedBy: D.I. Gavryushin; **Event:** samplingProtocol: Sweep net; eventDate: 2013-06-06; verbatimEventDate: 6/Jul/2013; **Record Level:** institutionCode: ZMMU; basisOfRecord: PreservedSpecimen
- **Type status:** Other material. **Occurrence:** occurrenceRemarks: 1 female; recordedBy: D.I. Gavryushin; individualCount: 1; sex: female; preparations: Pinned; occurrenceID: EU_LIM_842; **Taxon:** scientificName: Symplecta (Symplecta) hybrida (Meigen, 1804); family: Limoniidae; genus: Symplecta; subgenus: Symplecta; specificEpithet: hybrida; scientificNameAuthorship: (Meigen, 1804); **Location:** country: Belarus; stateProvince: Minsk; county: Barysaw; locality: Vialikaje Stachava; verbatimElevation: 156 m; minimumElevationInMeters: 156; decimalLatitude: 54.26555; decimalLongitude: 28.38332; **Identification:** identifiedBy: D.I. Gavryushin; **Event:** samplingProtocol: Sweep net; eventDate: 2013-06-07; verbatimEventDate: 7/Jul/2013; **Record Level:** institutionCode: ZMMU; basisOfRecord: PreservedSpecimen

#### Distribution

First records from Belarus.

### Symplecta (Psiloconopa) lindrothi

(Tjeder, 1955)

5D31CC6A-3E97-5A45-A424-CA8D197C7A7F

https://ccw.naturalis.nl/detail.php?id=4246

#### Materials

- **Type status:** Other material. **Occurrence:** catalogNumber: 666175; occurrenceRemarks: 5 male+female; recordedBy: K.M. Olsen; individualCount: 5; sex: male, female; preparations: Ethanol; occurrenceID: EU_LIM_835; **Taxon:** scientificName: Symplecta (Psiloconopa) lindrothi (Tjeder, 1955); family: Limoniidae; genus: Symplecta; subgenus: Psiloconopa; specificEpithet: lindrothi; scientificNameAuthorship: (Tjeder, 1955); **Location:** country: Norway; stateProvince: Finnmark; municipality: Tana; locality: Bodnesáttu – (N Seida); verbatimElevation: 5 m; minimumElevationInMeters: 5; decimalLatitude: 70.24973; decimalLongitude: 28.1806; **Identification:** identifiedBy: K.M. Olsen; **Event:** samplingProtocol: Sweep net; eventDate: 2020-06-01; verbatimEventDate: 0/Jul/2020; **Record Level:** institutionCode: PCKMO; basisOfRecord: PreservedSpecimen
- **Type status:** Other material. **Occurrence:** catalogNumber: 666262; occurrenceRemarks: 2 male+female; recordedBy: K.M. Olsen; individualCount: 2; sex: male, female; preparations: Ethanol; occurrenceID: EU_LIM_836; **Taxon:** scientificName: Symplecta (Psiloconopa) lindrothi (Tjeder, 1955); family: Limoniidae; genus: Symplecta; subgenus: Psiloconopa; specificEpithet: lindrothi; scientificNameAuthorship: (Tjeder, 1955); **Location:** country: Norway; stateProvince: Finnmark; municipality: Tana; locality: Lismajoki–Nuorinjálbmi; verbatimElevation: 10 m; minimumElevationInMeters: 10; decimalLatitude: 70.14961; decimalLongitude: 28.18817; **Identification:** identifiedBy: K.M. Olsen; **Event:** samplingProtocol: Sweep net; eventDate: 2020-06-08; verbatimEventDate: 08/Jul/2020; **Record Level:** institutionCode: ZMUB; basisOfRecord: PreservedSpecimen
- **Type status:** Other material. **Occurrence:** occurrenceRemarks: 1 male; recordedBy: V.E. Pilipenko; individualCount: 1; sex: male; occurrenceID: EU_LIM_837; **Taxon:** scientificName: Symplecta (Psiloconopa) meigeni (Zetterstedt, 1838); family: Limoniidae; genus: Symplecta; subgenus: Psiloconopa; specificEpithet: meigeni; scientificNameAuthorship: (Zetterstedt, 1838); **Location:** country: Russia; stateProvince: Central European Russia; county: Moskovskaya Oblast; municipality: Solnechnogorsk district; locality: Alabushevo; verbatimElevation: 220 m; minimumElevationInMeters: 220; decimalLatitude: 55.99676; decimalLongitude: 37.14919; **Identification:** identifiedBy: V.E. Pilipenko; **Event:** samplingProtocol: Sweep net; eventDate: 1992-05-25; verbatimEventDate: 25/May/1992; **Record Level:** institutionCode: VPMC; basisOfRecord: PreservedSpecimen

#### Distribution

First records from Norway.

### Symplecta (Psiloconopa) meigeni

(Zetterstedt, 1838)

42CFF6CD-B6C2-570E-B376-F51188F61FA4

https://ccw.naturalis.nl/detail.php?id=4252

#### Materials

- **Type status:** Other material. **Occurrence:** occurrenceRemarks: 1 male; recordedBy: V.E. Pilipenko; individualCount: 1; sex: male; occurrenceID: EU_LIM_837; **Taxon:** scientificName: Symplecta (Psiloconopa) meigeni (Zetterstedt, 1838); family: Limoniidae; genus: Symplecta; subgenus: Psiloconopa; specificEpithet: meigeni; scientificNameAuthorship: (Zetterstedt, 1838); **Location:** country: Russia; stateProvince: Central European Russia; county: Moskovskaya Oblast; municipality: Solnechnogorsk district; locality: Alabushevo; verbatimElevation: 220 m; minimumElevationInMeters: 220; decimalLatitude: 55.99676; decimalLongitude: 37.14919; **Identification:** identifiedBy: V.E. Pilipenko; **Event:** samplingProtocol: Sweep net; eventDate: 1992-05-25; verbatimEventDate: 25/May/1992; **Record Level:** institutionCode: VPMC; basisOfRecord: PreservedSpecimen

#### Distribution

First records from Russia: RUC.

### Symplecta (Psiloconopa) stictica stictica

(Meigen, 1818)

4A638025-0C87-5244-B208-4342359CF703

https://ccw.naturalis.nl/detail.php?id=4272

#### Materials

- **Type status:** Other material. **Occurrence:** occurrenceRemarks: 1 male; recordedBy: K.M. Olsen; individualCount: 1; sex: male; preparations: Ethanol; occurrenceID: EU_LIM_838; **Taxon:** scientificName: Symplecta (Psiloconopa) stictica stictica (Meigen, 1818); family: Limoniidae; genus: Symplecta; subgenus: Psiloconopa; specificEpithet: stictica; infraspecificEpithet: stictica; scientificNameAuthorship: (Meigen, 1818); **Location:** country: Iceland; stateProvince: Northeastern Region; municipality: Hörgárstveit; locality: Gaseyri – (Gásir); verbatimElevation: 5 m; minimumElevationInMeters: 5; decimalLatitude: 65.78453; decimalLongitude: -18.16435; **Identification:** identifiedBy: K.M. Olsen; **Event:** eventDate: 2018-06-12; verbatimEventDate: 12/Jul/2018; **Record Level:** institutionCode: NHMO; basisOfRecord: PreservedSpecimen

#### Distribution

First record from Iceland.

### Symplecta (Symplecta) tripilata

(Alexander, 1957)

774E2BFA-13A8-5EFE-B66B-FF0BE15E935B

https://ccw.naturalis.nl/detail.php?id=4305

#### Materials

- **Type status:** Other material. **Occurrence:** occurrenceRemarks: 1 male; recordedBy: V.I. Lantsov; individualCount: 1; sex: male; preparations: Pinned; occurrenceID: EU_LIM_843; **Taxon:** scientificName: Symplecta (Symplecta) tripilata (Alexander, 1957); family: Limoniidae; genus: Symplecta; subgenus: Symplecta; specificEpithet: tripilata; scientificNameAuthorship: (Alexander, 1957); **Location:** country: Russia; stateProvince: North Caucasus; county: Republic of Dagestan; municipality: Akhtynskiy, Gra; locality: the Samur Ridge, S slope, in vicinity of village Gra, Chaharkam River Valley, Samur River basin; verbatimElevation: 880 m; minimumElevationInMeters: 880; decimalLatitude: 41.45; decimalLongitude: 47.96667; **Identification:** identifiedBy: V.I. Lantsov; **Event:** samplingProtocol: Sweep net; eventDate: 2003-05-03; verbatimEventDate: 03/May/2003; habitat: near the rock along which the stream flows; **Record Level:** institutionCode: IEMT; basisOfRecord: PreservedSpecimen

#### Distribution

Presence of the species in Russia: NC mentioned in Lantsov (2020) without further details. Here, we publish the collection data for that record.

### Tasiocera (Dasymolophilus) exigua

Savchenko, 1973

9B803F54-A939-5D22-875E-F8D5BEE3F5CC

https://ccw.naturalis.nl/detail.php?id=4357

#### Materials

- **Type status:** Other material. **Occurrence:** occurrenceRemarks: 6 males, 2 females; recordedBy: M. Pollet; individualCount: 8; sex: male, female; preparations: Ethanol; occurrenceID: EU_LIM_844; **Taxon:** scientificName: Tasiocera (Dasymolophilus) exigua Savchenko, 1973; family: Limoniidae; genus: Tasiocera; subgenus: Dasymolophilus; specificEpithet: exigua; scientificNameAuthorship: Savchenko, 1973; **Location:** island: Corsica; country: France; county: Corse-du-Sud; municipality: Serra di Scopamène; locality: Castellu d'Ornucciu; verbatimElevation: 1568 m; minimumElevationInMeters: 1568; decimalLatitude: 41.50005; decimalLongitude: 9.09276; **Identification:** identifiedBy: P. Boardman; **Event:** samplingProtocol: Pan trap; eventDate: 2019-06-30; verbatimEventDate: 30/Jun/2019; **Record Level:** institutionCode: MNHN; basisOfRecord: PreservedSpecimen
- **Type status:** Other material. **Occurrence:** catalogNumber: 566053, 657074; occurrenceRemarks: 10 males; recordedBy: K.M. Olsen; individualCount: 10; sex: male; preparations: Ethanol; occurrenceID: EU_LIM_845; **Taxon:** scientificName: Tasiocera (Dasymolophilus) exigua Savchenko, 1973; family: Limoniidae; genus: Tasiocera; subgenus: Dasymolophilus; specificEpithet: exigua; scientificNameAuthorship: Savchenko, 1973; **Location:** country: Norway; stateProvince: Buskerud; municipality: Ringerike; locality: Veksalbekken; verbatimElevation: 95 m; minimumElevationInMeters: 95; decimalLatitude: 60.17397; decimalLongitude: 10.19885; **Identification:** identifiedBy: K.M. Olsen; **Event:** samplingProtocol: SLAM-trap/Malaise trap; eventDate: 2017-05-02/2017-06-23; verbatimEventDate: 02/May-23/Jun/2017; **Record Level:** institutionCode: NHMO; basisOfRecord: PreservedSpecimen
- **Type status:** Other material. **Occurrence:** catalogNumber: 550610; occurrenceRemarks: 3 males; recordedBy: K.M. Olsen; individualCount: 3; sex: male; preparations: Ethanol; occurrenceID: EU_LIM_846; **Taxon:** scientificName: Tasiocera (Dasymolophilus) exigua Savchenko, 1973; family: Limoniidae; genus: Tasiocera; subgenus: Dasymolophilus; specificEpithet: exigua; scientificNameAuthorship: Savchenko, 1973; **Location:** country: Norway; stateProvince: Buskerud; municipality: Ringerike; locality: Veksalbekken; verbatimElevation: 90-110 m; minimumElevationInMeters: 90; maximumElevationInMeters: 110; decimalLatitude: 60.17544; decimalLongitude: 10.19964; **Identification:** identifiedBy: K.M. Olsen; **Event:** samplingProtocol: Sweep net; eventDate: 2017-06-23; verbatimEventDate: 23/Jun/2017; **Record Level:** institutionCode: PCKMO; basisOfRecord: PreservedSpecimen
- **Type status:** Other material. **Occurrence:** catalogNumber: 568987, 571729, 644197; occurrenceRemarks: 20 male+female; recordedBy: K.M. Olsen; individualCount: 20; sex: male, female; preparations: Ethanol; occurrenceID: EU_LIM_847; **Taxon:** scientificName: Tasiocera (Dasymolophilus) exigua Savchenko, 1973; family: Limoniidae; genus: Tasiocera; subgenus: Dasymolophilus; specificEpithet: exigua; scientificNameAuthorship: Savchenko, 1973; **Location:** country: Norway; stateProvince: Buskerud; municipality: Ringerike; locality: Veksalbekken; verbatimElevation: 95 m; minimumElevationInMeters: 95; decimalLatitude: 60.17397; decimalLongitude: 10.19885; **Identification:** identifiedBy: K.M. Olsen; **Event:** samplingProtocol: Malaise trap; eventDate: 2017-06-23/2017-08-11; verbatimEventDate: 23/Jun-11/Aug/2017; **Record Level:** institutionCode: ZMUB | PCKMO; basisOfRecord: PreservedSpecimen
- **Type status:** Other material. **Occurrence:** occurrenceRemarks: 1 male; recordedBy: L.-P. Kolcsár | E. Török; individualCount: 1; sex: male; preparations: Ethanol; occurrenceID: EU_LIM_848; **Taxon:** scientificName: Tasiocera (Dasymolophilus) exigua Savchenko, 1973; family: Limoniidae; genus: Tasiocera; subgenus: Dasymolophilus; specificEpithet: exigua; scientificNameAuthorship: Savchenko, 1973; **Location:** country: Serbia; municipality: Kopaonik; locality: Kopaonik Mts.; verbatimElevation: 1600 m; minimumElevationInMeters: 1600; decimalLatitude: 43.2981; decimalLongitude: 20.78706; **Identification:** identifiedBy: L.-P. Kolcsár; **Event:** samplingProtocol: Sweep net; eventDate: 2017-06-22; verbatimEventDate: 22/Jun/2017; **Record Level:** institutionCode: CKLP; basisOfRecord: PreservedSpecimen
- **Type status:** Other material. **Occurrence:** occurrenceRemarks: 1 male; recordedBy: L.-P. Kolcsár | E. Török; individualCount: 1; sex: male; preparations: Ethanol; occurrenceID: EU_LIM_849; **Taxon:** scientificName: Tasiocera (Dasymolophilus) exigua Savchenko, 1973; family: Limoniidae; genus: Tasiocera; subgenus: Dasymolophilus; specificEpithet: exigua; scientificNameAuthorship: Savchenko, 1973; **Location:** country: Serbia; municipality: Banja Jošanica; locality: Kopaonik Mts., Paljestica River; verbatimElevation: 871 m; minimumElevationInMeters: 871; decimalLatitude: 43.36343; decimalLongitude: 20.7477; **Identification:** identifiedBy: L.-P. Kolcsár; **Event:** samplingProtocol: Sweep net; eventDate: 2017-06-22; verbatimEventDate: 22/Jun/2017; **Record Level:** institutionCode: CKLP; basisOfRecord: PreservedSpecimen
- **Type status:** Other material. **Occurrence:** occurrenceRemarks: 3 males; recordedBy: M. Lindström; individualCount: 3; sex: male; preparations: Ethanol; occurrenceID: EU_LIM_850; **Taxon:** scientificName: Tasiocera (Dasymolophilus) exigua Savchenko, 1973; family: Limoniidae; genus: Tasiocera; subgenus: Dasymolophilus; specificEpithet: exigua; scientificNameAuthorship: Savchenko, 1973; **Location:** country: Sweden; stateProvince: Halland; municipality: Laholm; locality: Hälleforsen, Hasslöv; verbatimElevation: 100 m; minimumElevationInMeters: 100; decimalLatitude: 56.39928; decimalLongitude: 13.0221; **Identification:** identifiedBy: M. Lindström; **Event:** samplingProtocol: Malaise trap; eventDate: 2013-04-27/2013-06-05; verbatimEventDate: 27/Apr-5/Jun/2013; **Record Level:** institutionCode: PCML; basisOfRecord: PreservedSpecimen
- **Type status:** Other material. **Occurrence:** occurrenceRemarks: 17 males; recordedBy: M. Lindström; individualCount: 17; sex: male; preparations: Ethanol; occurrenceID: EU_LIM_851; **Taxon:** scientificName: Tasiocera (Dasymolophilus) exigua Savchenko, 1973; family: Limoniidae; genus: Tasiocera; subgenus: Dasymolophilus; specificEpithet: exigua; scientificNameAuthorship: Savchenko, 1973; **Location:** country: Sweden; stateProvince: Halland; municipality: Laholm; locality: Skillnadsbäcken, Dömestorp; verbatimElevation: 100 m; minimumElevationInMeters: 100; decimalLatitude: 56.41583; decimalLongitude: 12.97094; **Identification:** identifiedBy: M. Lindström; **Event:** samplingProtocol: Malaise trap; eventDate: 2013-04-27/2013-06-05; verbatimEventDate: 27/Apr-5/Jun/2013; **Record Level:** institutionCode: PCML; basisOfRecord: PreservedSpecimen

#### Distribution

First records from France (from Corsica), Norway and Serbia. The species was first reported from Sweden in CCW, based on records uploaded to Artportalen (2017); here, we publish the collection data for those records.

### Tasiocera (Dasymolophilus) robusta

(Bangerter, 1947)

66387E70-5034-59DC-83B3-560984F38143

https://ccw.naturalis.nl/detail.php?id=4382

#### Materials

- **Type status:** Other material. **Occurrence:** occurrenceRemarks: 2 males; recordedBy: L.-P. Kolcsár | E. Török; individualCount: 2; sex: male; preparations: Ethanol; occurrenceID: EU_LIM_852; **Taxon:** scientificName: Tasiocera (Dasymolophilus) robusta (Bangerter, 1947); family: Limoniidae; genus: Tasiocera; subgenus: Dasymolophilus; specificEpithet: robusta; scientificNameAuthorship: (Bangerter, 1947); **Location:** country: North Macedonia; municipality: Izvor; locality: Treska River; verbatimElevation: 755 m; minimumElevationInMeters: 755; decimalLatitude: 41.48021; decimalLongitude: 20.83466; **Identification:** identifiedBy: L.-P. Kolcsár; **Event:** samplingProtocol: Sweep net; eventDate: 2017-06-30; verbatimEventDate: 30/Jun/2017; **Record Level:** institutionCode: CKLP; basisOfRecord: PreservedSpecimen
- **Type status:** Other material. **Occurrence:** occurrenceRemarks: 2 males, 2 females; recordedBy: L.-P. Kolcsár | E. Török; individualCount: 4; sex: male, female; preparations: Ethanol; occurrenceID: EU_LIM_853; **Taxon:** scientificName: Tasiocera (Dasymolophilus) robusta (Bangerter, 1947); family: Limoniidae; genus: Tasiocera; subgenus: Dasymolophilus; specificEpithet: robusta; scientificNameAuthorship: (Bangerter, 1947); **Location:** country: Romania; stateProvince: Bihor; municipality: Boga; locality: Bihor Mts., Boga Valley; verbatimElevation: 900 m; minimumElevationInMeters: 900; decimalLatitude: 46.60083; decimalLongitude: 22.66627; **Identification:** identifiedBy: L.-P. Kolcsár; **Event:** samplingProtocol: Sweep net; eventDate: 2011-06-29; verbatimEventDate: 29/Jun/2011; **Record Level:** institutionCode: CKLP; basisOfRecord: PreservedSpecimen

#### Distribution

First records from North Macedonia and Romania.

### Thaumastoptera (Thaumastoptera) calceata

Mik, 1866

3518C3E8-FCBF-5586-A0C4-5EBF0369E1BF

https://ccw.naturalis.nl/detail.php?id=11327

#### Materials

- **Type status:** Other material. **Occurrence:** occurrenceRemarks: 1 female; recordedBy: B. van Maanen; individualCount: 1; sex: female; occurrenceID: EU_LIM_854; **Taxon:** scientificName: Thaumastoptera (Thaumastoptera) calceata Mik, 1866; family: Limoniidae; genus: Thaumastoptera; subgenus: Thaumastoptera; specificEpithet: calceata; scientificNameAuthorship: Mik, 1866; **Location:** country: Netherlands; stateProvince: Limburg; decimalLatitude: 51.21; decimalLongitude: 5.97; **Identification:** identifiedBy: P. Oosterbroek; **Event:** samplingProtocol: Reared; eventDate: 2012; verbatimEventDate: 2012; **Record Level:** basisOfRecord: HumanObservation

#### Distribution

The species was first reported from The Netherlands in the CCW by van Maanen (in litt. 2012). Here, we publish the collection data for that record.

## Supplementary Material

7747AD0F-1FF9-59E6-B9F3-973753A6092F 10.3897/BDJ.9.e67085.suppl1 Supplementary material 1 Subdivisions of European part of Russia and list of the territories (respublika, oblast, kray) included in subdivisions Data type additional information Brief description Six main subdivisions of the European territory of Russia have been established, as North, Northwest, Central, East, South European Russia and North Caucasus. We list the territories (respublika, oblast, kray) included in these subdivisions, following the geopolitical boundaries of the Catalogue of the Craneflies of the World. We suggest a modification as Permskaya Oblast and Komi-Permyatskiy Avtonomnyy Okrug were merged in 2005 and it is now called Permskiy Kray. The Russian name of the subdivisions and territories are included. File: oo_513163.tsv https://binary.pensoft.net/file/513163 Levente-Péter Kolcsár, Pjotr Oosterbroek, Dmitry I. Gavryushin, Kjell Magne Olsen, Nikolai M. Paramonov, Valentin Pilipenko, Jaroslav Starý, Alexei Polevoi, Vladimir I. Lantsov, Eulalia Eiroa, Michael Andersson, Jukka Salmela, Clovis Quindroit, Micha C. d'Oliveira, E. Geoffrey Hancock, Jorge Mederos, Pete Boardman, Kozo Watanabe

## Figures and Tables

**Figure 3.**
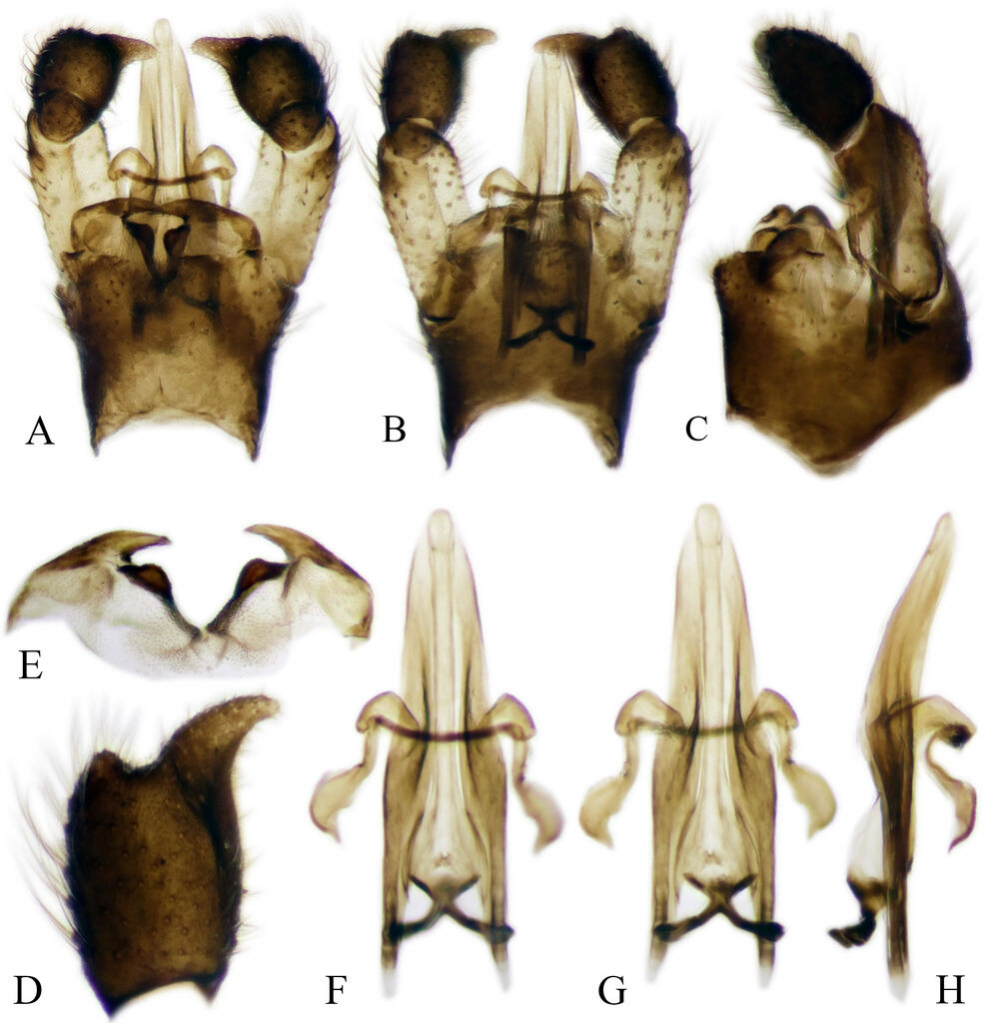
Dactylolabis (Coenolabis) posthabita (Bergroth, 1888) male terminalia. **A**. Dorsal view; **B**. Ventral view; **C**. Lateral view; **D**. Gonostylus; **E**. Proctiger; **F**. Aedeagus complex dorsal view; **G**. Aedeagus complex ventral view; **H**. Aedeagus complex lateral view. Specimen: Hungary, Potony.

**Figure 4.**
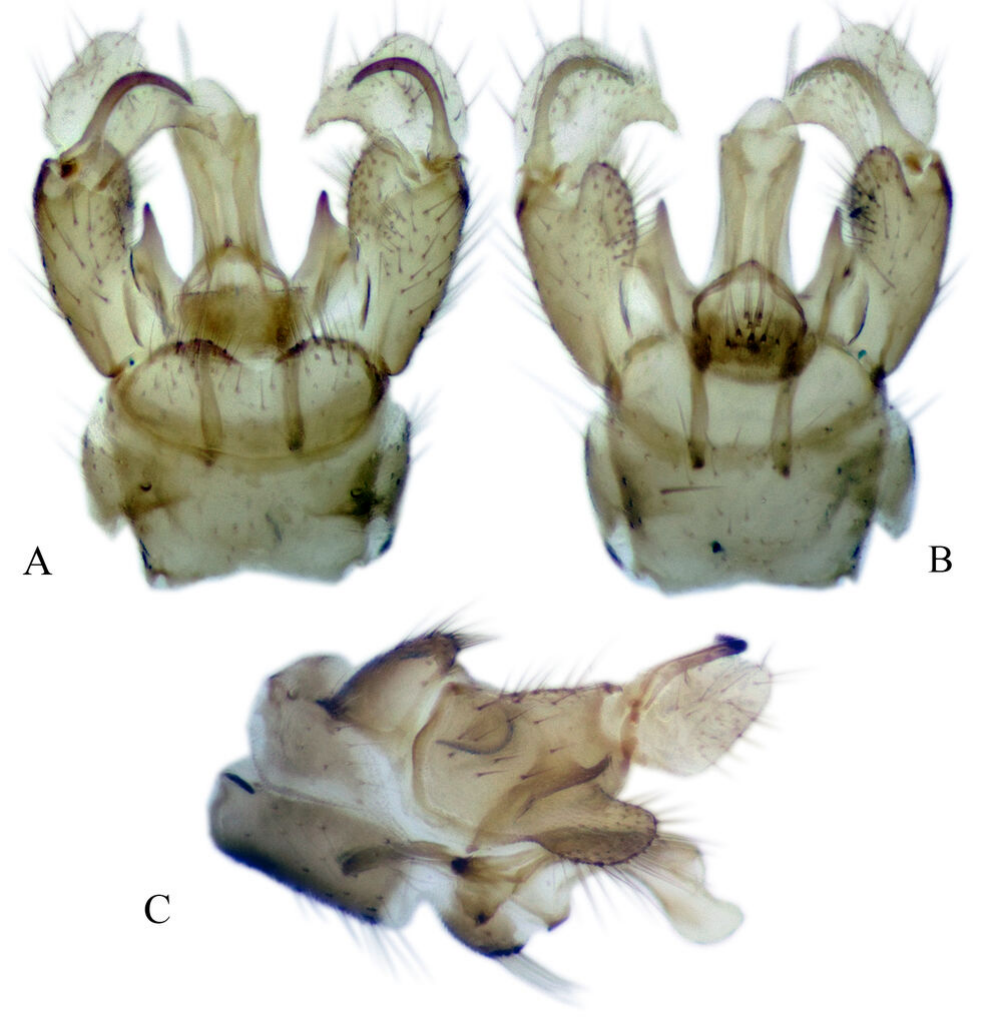
Dicranomyia (Melanolimonia) hamata Becker, 1908 male terminalia. **A**. Dorsal view; **B**. Ventral view; **C**. Lateral view. Specimen: Albania, Iljas, Gjipe Canyon.

**Figure 5.**
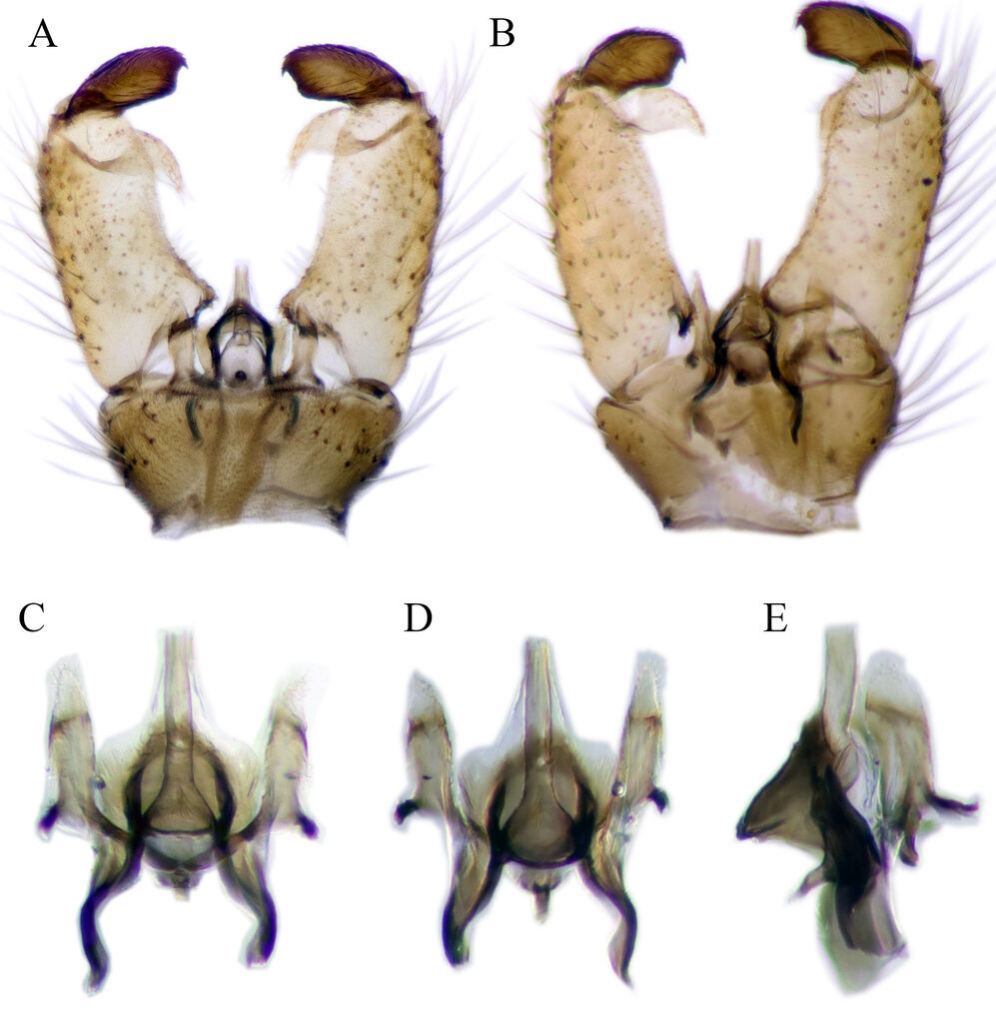
*Eloeophilasparsipunctum* Starý, 2009 male terminalia. **A**. Dorsal view; **B**. Ventral view; **C.** Aedeagus complex dorsal view; **D**. Aedeagus complex ventral view; **E**. Aedeagus complex lateral view. Specimen: Romania, Măguri-Răcătău, Gilău Mts., Someșul Rece River.

**Figure 6.**
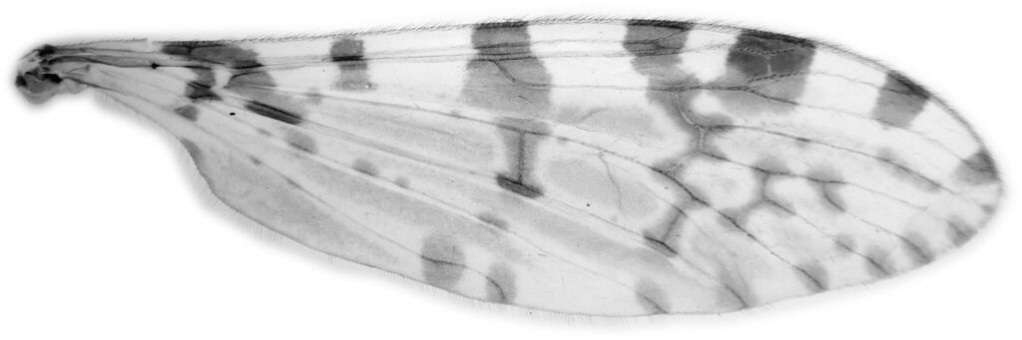
*Eloeophilasparsipunctum* Starý, 2009 wing. Specimen: Romania, Măguri-Răcătău, Gilău Mts., Someșul Rece River.

**Figure 7.**
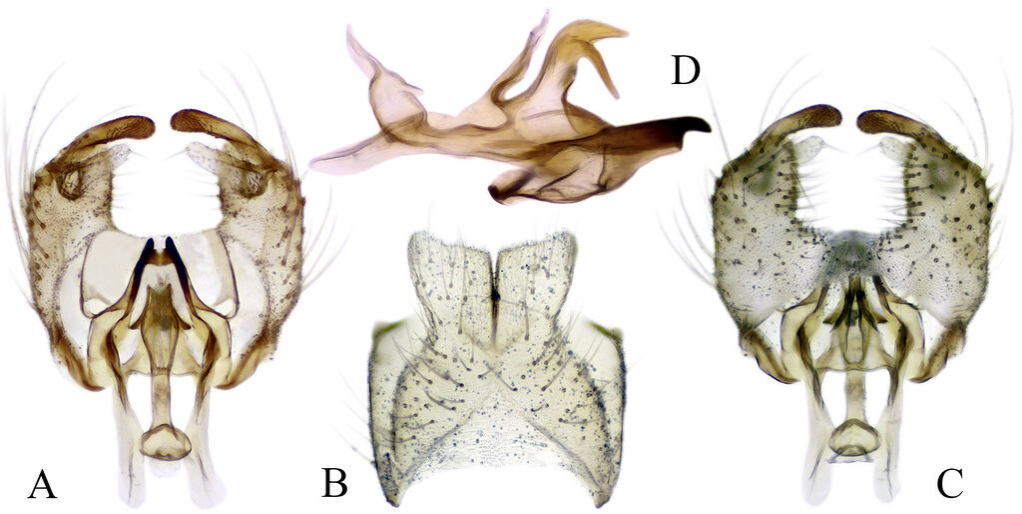
*Ilisiaoccoecata* Edwards, 1936 male terminalia. **A**. Aedeagus complex, gonocoxites and gonostyli, dorsal view; **B**. Tergite 9 ventral view; **C.** Aedeagus complex, gonocoxites and gonostyli, ventral view; **D**. Aedeagus complex lateral view. Specimen: Romania, Hagota, Giurgeu Mts., Tisașul Valley.

**Figure 8.**
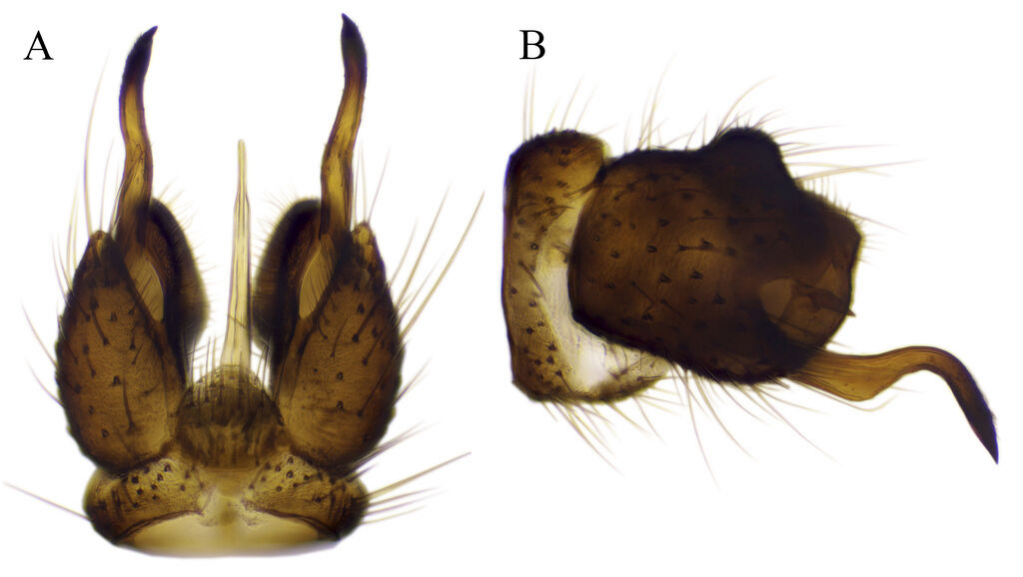
Molophilus (Molophilus) bihamatus de Meijere, 1918 male terminalia. **A**. Ventral view; **B**. Lateral view. Specimen: Romania, Voșlăbeni, Senetea.

**Figure 9.**
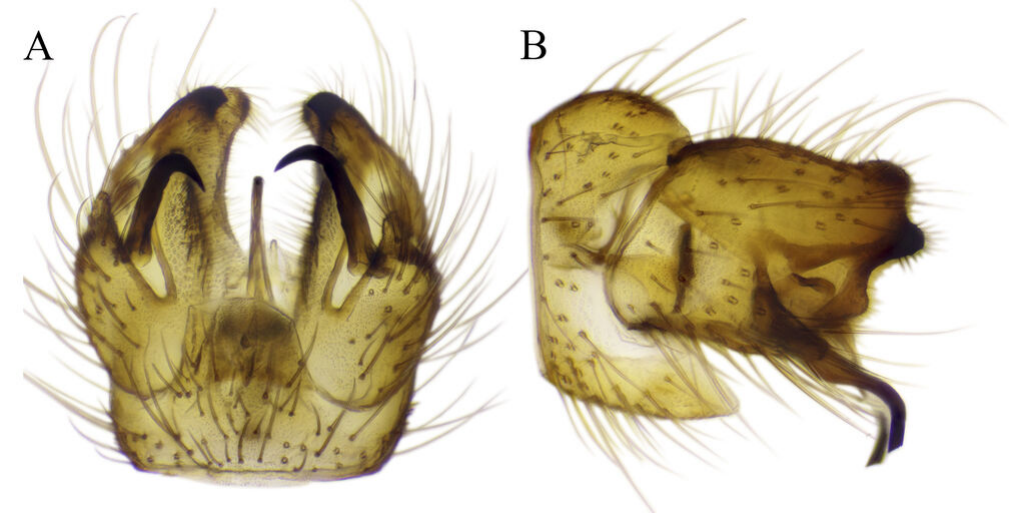
Molophilus (Molophilus) crassipygus de Meijere, 1918 male terminalia. **A**. Ventral view; **B**. Lateral view. Specimen: Serbia, Sikirje, Kukavica Mts.

**Figure 10.**
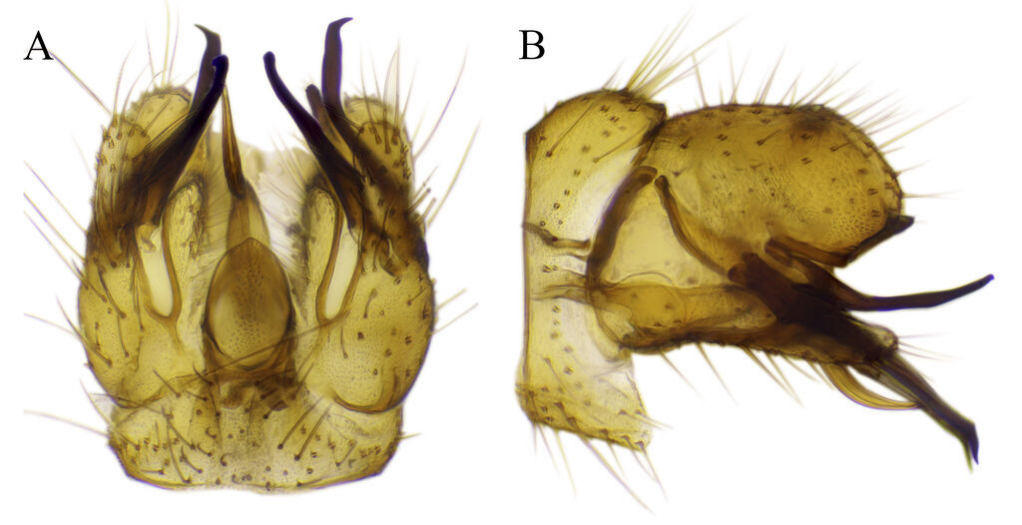
Molophilus (Molophilus) obsoletus Lackschewitz, 1940 male terminalia. **A**. Ventral view; **B**. Lateral view. Specimen: Serbia, Kopaonik, Kopaonik Mts.

**Figure 11.**
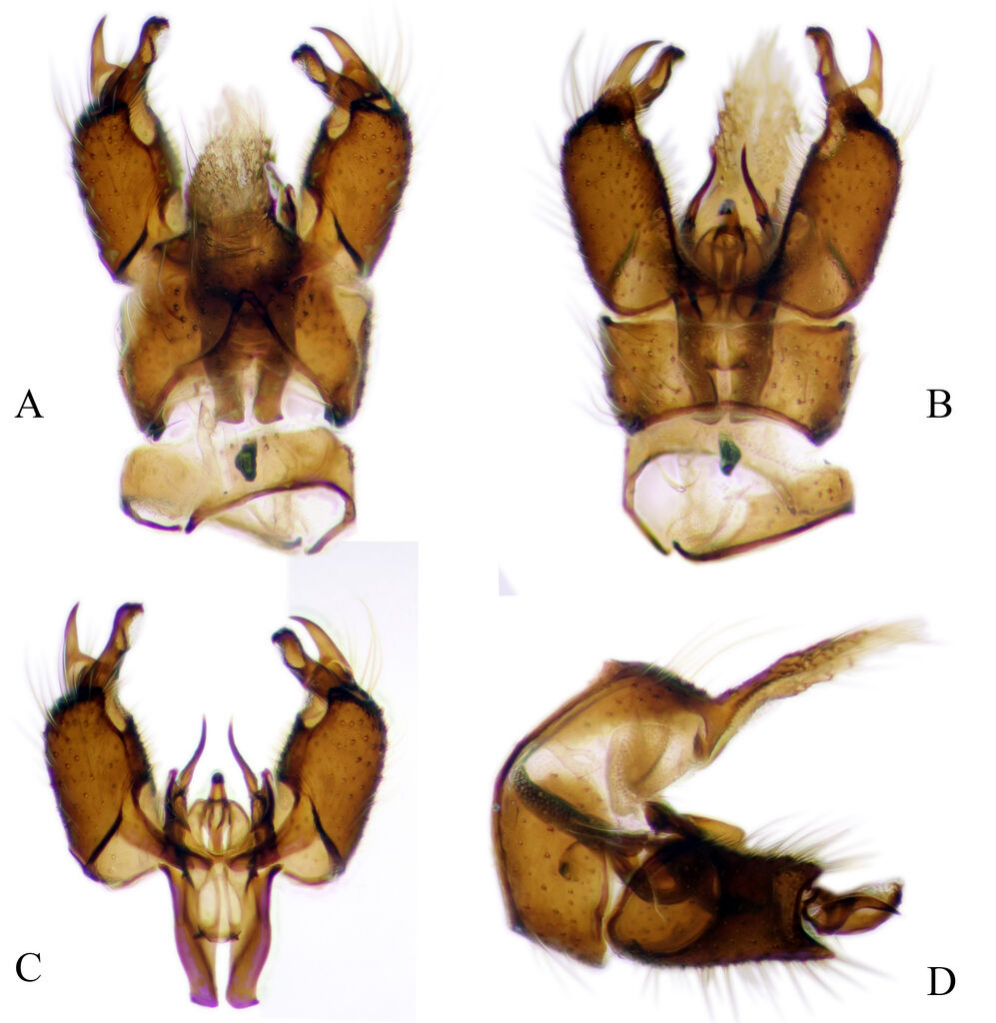
Ormosia (Ormosia) microstyla Savchenko, 1973 male terminalia. **A**. Ventral view; **B**. Dorsal view; **C**. Ventral view after tergite and sternite 9 removed; **D.** Lateral view. Specimen: Romania, Bălan, Hăşmaş Mts., Piatra Singuratică.

**Figure 12.**
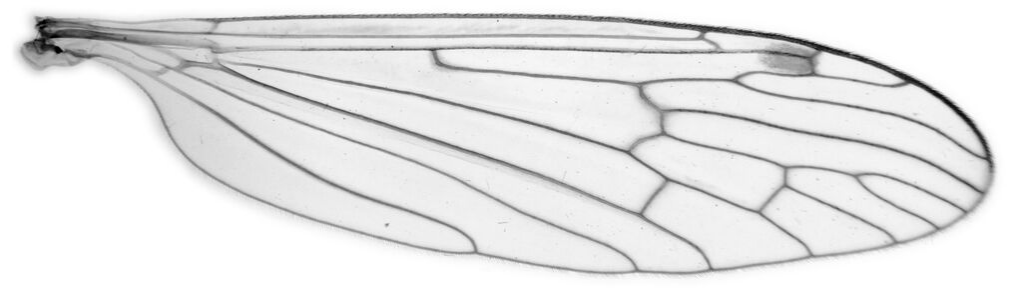
Phylidorea (Macrolabina) alexanderi (Stary, 1974) male wing. Specimen: Serbia, Kopaonik, Kopaonik Mts.

**Figure 13.**
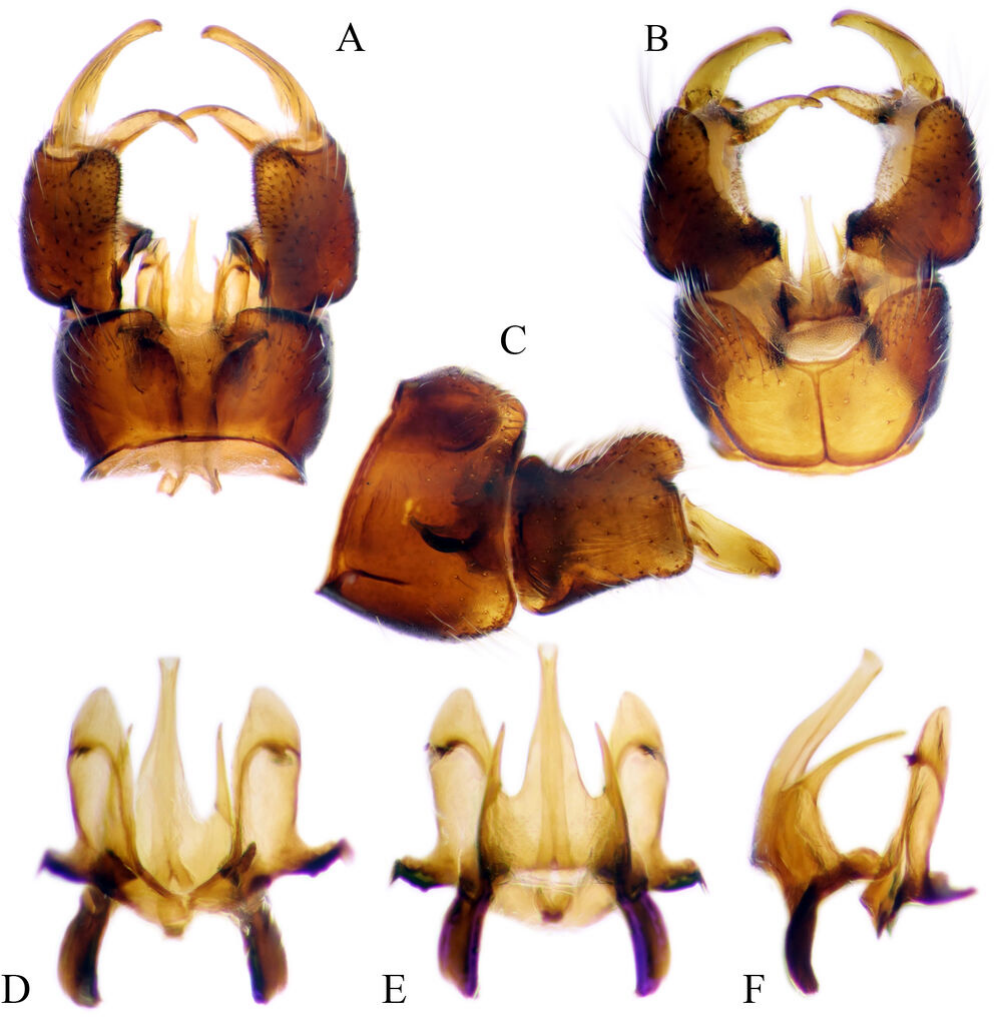
Phylidorea (Macrolabina) alexanderi (Stary, 1974) male terminalia. **A**. Dorsal view; **B**. Ventral view; **C.** Lateral view; **D**. Aedeagus complex dorsal view; **E**. Aedeagus complex ventral view; **F**. Aedeagus complex lateral view. Specimen: Serbia, Kopaonik, Kopaonik Mts.

**Figure 14.**
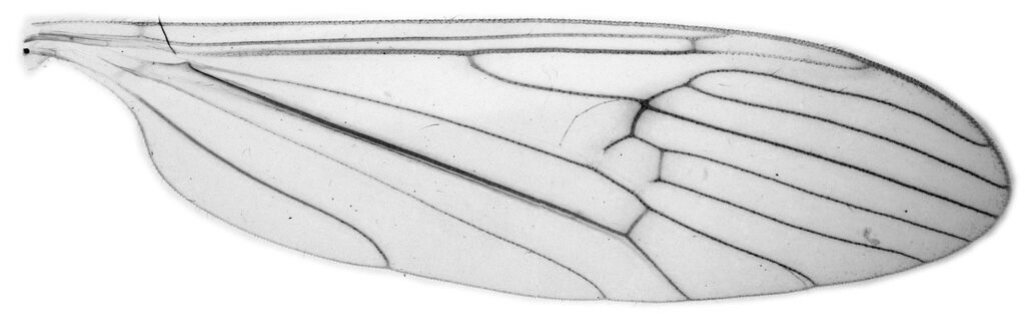
*Phyllolabisalexanderi* Lackschewitz, 1940 male wing. Specimen: North Macedonia, Bratin Dol, Pelister Mts.

**Figure 15.**
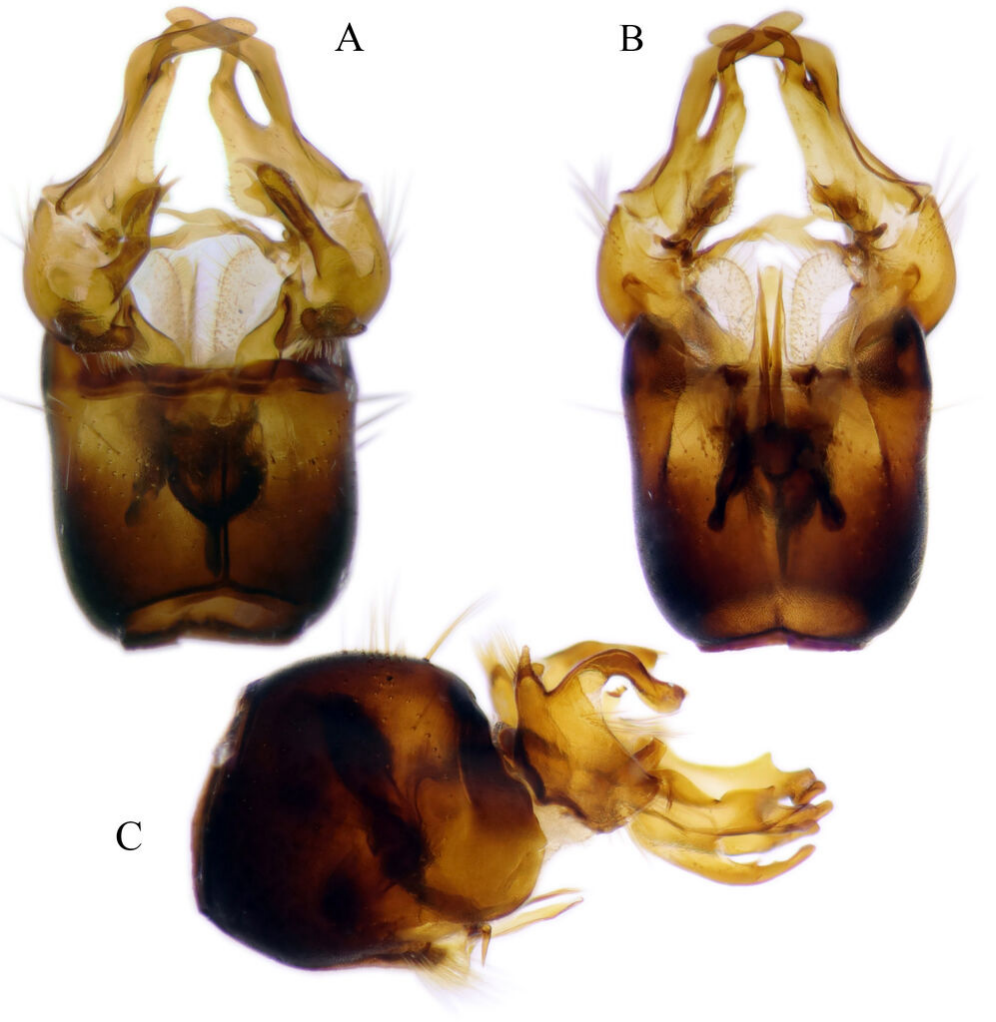
*Phyllolabisalexanderi* Lackschewitz, 1940 male terminalia. **A.** Dorsal view; **B.** Ventral view; **C.** Lateral view. Specimen: North Macedonia, Bratin Dol, Pelister Mts.

**Figure 16.**
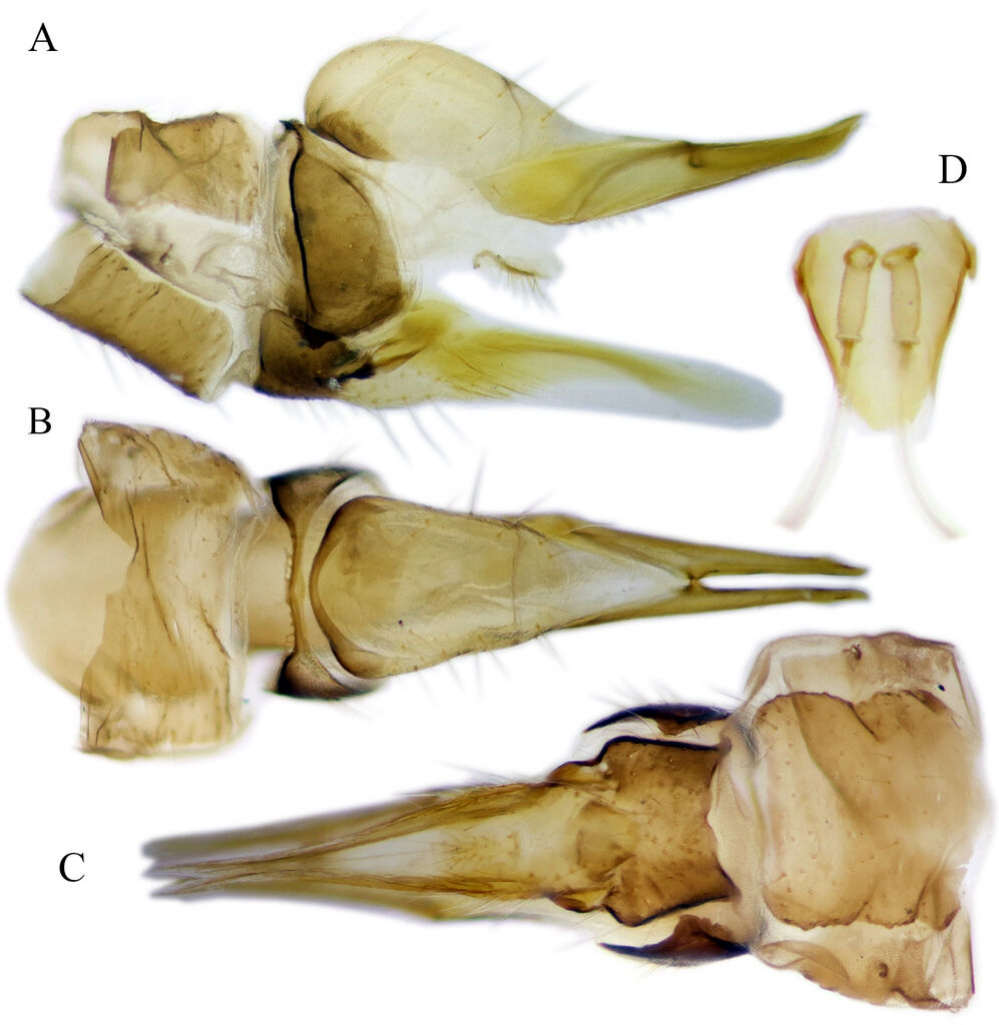
*Phyllolabisalexanderi* Lackschewitz, 1940 female terminalia. **A**. Lateral view; **B**. Dorsal view; **C**. Ventral view; **D**. Genital fork and genital opening. Specimen: North Macedonia, Bratin Dol, Pelister Mts.

**Figure 17.**
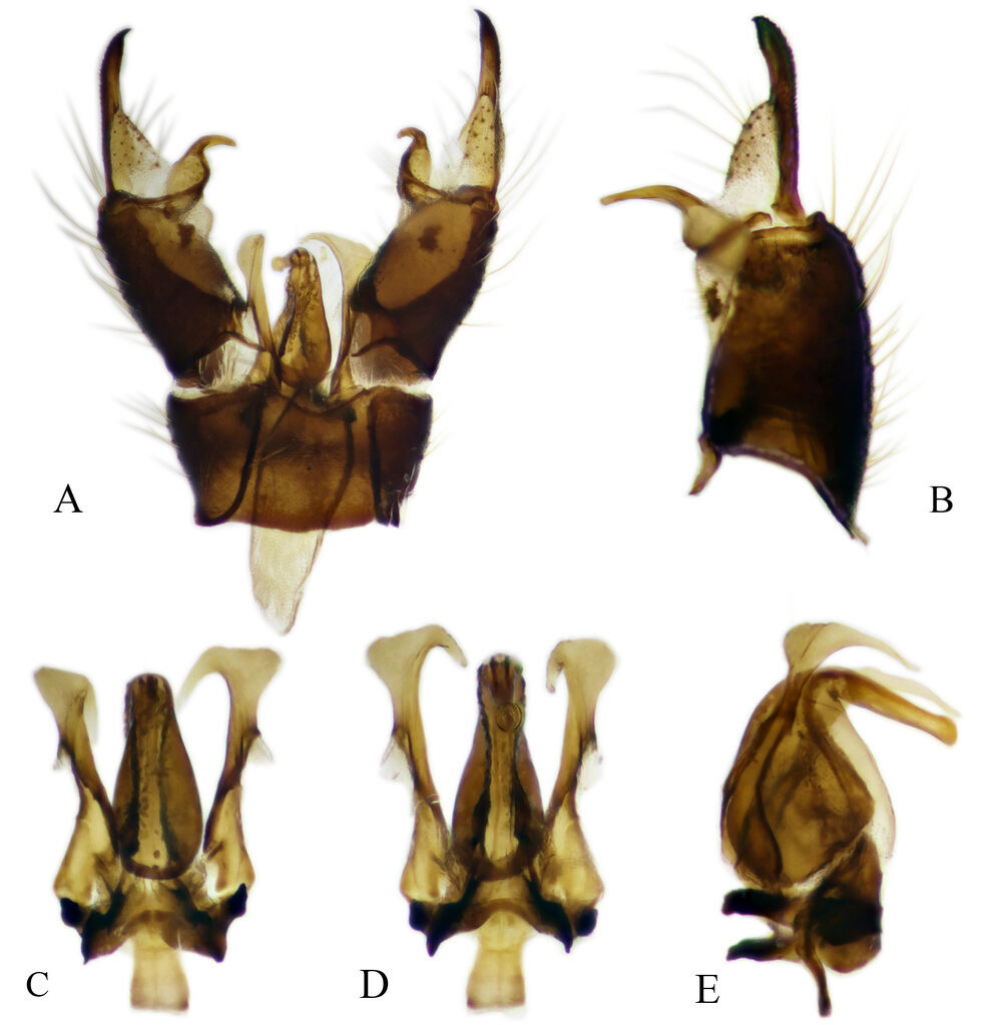
*Prionolabiscognata* (Lackschewitz, 1940) anterior parts of male. Specimen: Montenegro, Šavnik, Petnjica.

**Figure 18.**
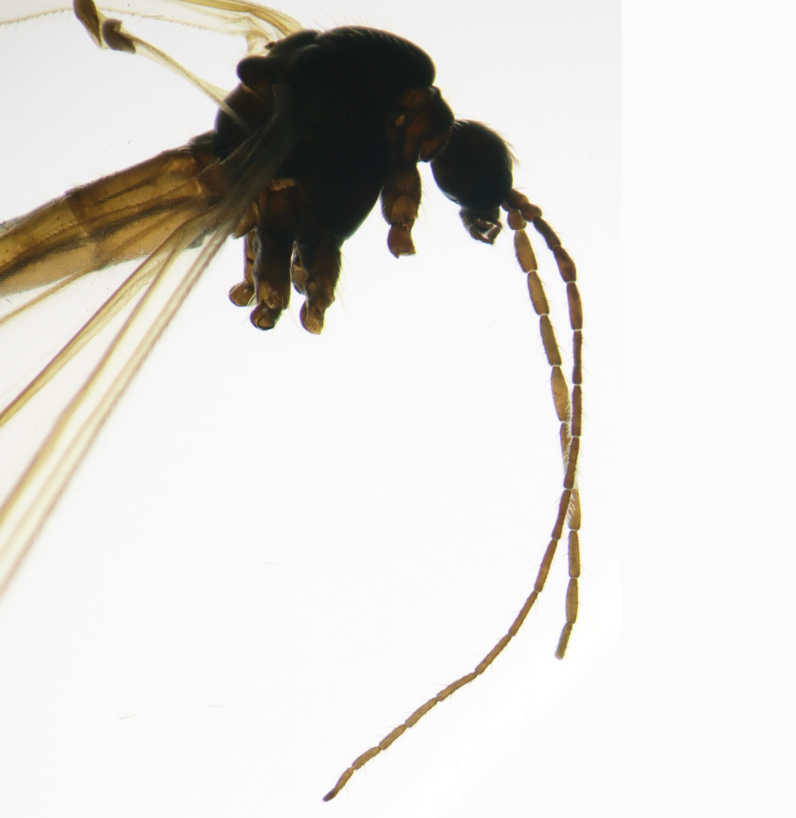
*Prionolabiscognata* (Lackschewitz, 1940) male wing. Specimen: Montenegro, Šavnik, Petnjica.

**Table 1.** List of species described from Europe between 2010 and 2020.

**Species**	**Publication**	**Distribution**
Austrolimnophila (Austrolimnophila) cretica Starý, 2014	Starý 2014b	Greece (Crete)
Austrolimnophila (Austrolimnophila) vikhrevi Gavryushin, 2015	Gavryushin 2015	Azerbajian, Georgia and Russia: North Caucasus
*Baeourarotherayi* Hancock, 2020	Hancock 2020	Spain (Jaen)
*Chioneadolomitana* Vanin, 2010	Vanin 2010	Italy (Belluno, Treviso, Udine)
*Chioneaolympiae* Vanin, 2010	Vanin 2010	Italy (Biella)
Dicranomyia (Idiopyga) boreobaltica Salmela, 2014	Salmela et al. 2014	Finland (northern Baltic area)
*Dicranophragmarelictum* Mederos, 2020	Mederos et al. 2020	Spain (Barcelona)
Ellipteroides (Ramagonomyia) mendli Hancock and Starý, 2019	Hancock and Starý 2019	Portugal (Faro) and Spain (south)
Erioptera (Erioptera) octobris Gavryushin, 2011	Gavryushin 2011	Russia: North Caucasus
*Geranomyiaeugeniana* Lantsov, 2015	Lantsov 2015a	Azerbajian, Georgia and Russia: North Caucasus
*Geranomyiafuscior* Starý, 2012	Starý 2012	Albania (Durazzo), Portugal (Faro) and Libya (Tripolis)
*Gonempedamariya* Pilipenko, 2017	Pilipenko 2017	Russia: North Caucasus
Gonomyia (Gonomyia) securiformis Starý, 2011	Starý 2011a	Bulgaria, Czech Rep., Italy (Piemonte) and Switzerland
Gonomyia (Gonomyia) undiformis Starý, 2011	Starý 2011a	Slovakia
Idiocera (Idiocera) cretopunctata Starý, 2020	Starý 2020a	Greece (Crete)
Idiocera (Idiocera) falcistylus Starý, 2020	Starý 2020a	Spain (Almeria)
*Limoniaenormis* Starý, 2017	Starý 2017	Greece (Crete)
*Limoniahartveldae* Starý, 2017	Starý 2017	Portugal (Guarda)
*Lipsothrixgaliciensis* Hancock and Hewitt, 2015	Hancock et al. 2015	Spain (Lugo)
Molophilus (Molophilus) balcanicus Kolcsár, 2015	Kolcsár et al. 2015b	Bulgaria (Stara Planina Mts)
Molophilus (Molophilus) brevifurcatus Starý, 2011	Starý 2011b	Czech Rep. and Slovakia
Molophilus (Molophilus) calabricus Starý, 2011	Starý 2011b	Italy (Calabria)
Molophilus (Molophilus) carbonis Starý, 2011	Starý 2011b	Portugal (Faro)
Molophilus (Molophilus) creticola Starý, 2011	Starý 2011b	Greece (Crete)
Molophilus (Molophilus) cypricola Starý, 2011	Starý 2011b	Cyprus
Molophilus (Molophilus) ibericus Starý, 2011	Starý 2011b	Spain and Morocco (High Atlas)
Molophilus (Molophilus) rohaceki Starý and Oboňa, 2019	Starý and Oboňa 2019	Greece (Taygetos Mts)
Molophilus (Molophilus) suboccultus Starý, 2011	Starý 2011b	Portugal (Faro)
Molophilus (Molophilus) zonzensis Boardman and Starý, 2020	Boardman and Starý 2020	France (Corsica)
*Prionolabispjotri* Hancock, 2015	Hancock et al. 2015	Portugal and Spain (Lugo)
Thaumastoptera (Thaumastoptera) spathifera Starý and Obaňa, 2018	Starý and Oboňa 2018	Italy (Sicily)

**Table 2.** Summarised list of publications reporting new country records from Europe during the period 2010–2020, including those that describe new species. The total number includes questionable and unconfirmed species.

**Country**	**Publications with new country record(s) during the period 2010-2020**	**No. of species reported as new during the period 2010-2020**	**No. of species reported as new in this article**	**Total no. of species including the new records**
Albania	Starý 2012	1	10	71
Andorra	Eiroa and Carles-Tolra 2019, Kolcsár et al. 2015b, Mederos et al. 2019, Petersen 2015	12	0	28
Austria	Kolcsár et al. 2017b, Petersen 2015, Reusch and Heiss 2012, Starý 2011b	8	1	277
Belarus	Paramonov 2019a, Paramonov and Sushko 2010	14	37	63
Belgium	Martens et al. 2014	1	5	165
Bosnia and Herzegovina	Kolcsár et al. 2017b	2	0	71
Bulgaria	Hubenov 2017, Kolcsár et al. 2015b, Starý 2011a, Starý and Stubbs 2015	7	2	222
Croatia	Kolcsár et al. 2015a	14	0	87
Cyprus	Starý 2011b	1	0	20
Czech Republic (Czechia)	Starý 2011a, Starý 2011b, Starý 2019, Starý and Stubbs 2015	6	0	300
Denmark	Byriel and Rojas 2017, Byriel et al. 2016, Fugleognatur 2012, Fugleognatur 2016, Salmela 2012c	11	0	171
Estonia		0	2	82
Finland	Salmela 2010a, Salmela 2010b, Salmela 2011b, Salmela 2012a, Salmela 2012b, Salmela 2013, Salmela et al. 2014	8	6	204
France	Boardman and Starý 2020, Kramer and Langlois 2019a, Kramer and Langlois 2019b, Quindroit and Lemoine 2020, Quindroit 2019, Quindroit 2020, Starý 2011b	18	7	279
Germany	Blick and Zaenker 2016	1	0	292
Greece	Kolcsár et al. 2015b, Kolcsár et al. 2017a, Kolcsár et al. 2017b, Petersen 2015, Starý 2011b, Starý 2014b, Starý 2017, Starý 2020a, Starý and Oboňa 2019, Starý and Stubbs 2015	14	14	116
Hungary	Kolcsár et al. 2017b, Kolcsár and Soltész 2018	30	16	177
Iceland		0	2	13
Ireland		0	0	131
Italy	Podenas 2011, Starý 2011a, Starý 2011b, Starý and Oboňa 2018, Starý and Stubbs 2015, Vanin 2010	9	6	280
Latvia		0	11	113
Liechtenstein		0	0	0
Lithuania	Markevičiūtė 2019	1	0	202
Luxembourg	Reusch and Weber 2013	4	0	35
Malta	Ebejer 2015	10	1	12
Moldova		0	0	18
Monaco		0	0	0
Montenegro	Kolcsár et al. 2017a, Kolcsár et al. 2017b	2	9	70
Netherlands	Dek and Oosterbroek 2013, Dek et al. 2014, Starý 2011a, Starý 2020b	7	2	153
North Macedonia	Kolcsár et al. 2017a	1	10	93
Norway	Olsen et al. 2018	40	42	216
Poland	Wiedeńska 2010, Wiedeńska 2014, Wiedeńska 2015, Wiedeńska 2017, Wiedeńska 2019	13	1	232
Portugal	Hancock and Starý 2019, Oosterbroek et al. 2020, Starý 2011b, Starý 2012, Starý 2014a, Starý 2017	55	5	85
Romania	Kolcsár et al. 2013, Kolcsár et al. 2015b, Kolcsár et al. 2017b	9	20	285
Russia: Central European Russia (RUC)	Krivosheina 2010, Paramonov 2016, Paramonov 2017, Paramonov 2018a, Paramonov and Klepikov 2014, Paramonov and Korobkov 2019, Paramonov and Pilipenko 2016, Pilipenko et al. 2020, Ruchin and Pilipenko 2015, Starý 2019	62	28	141
Russia: East European Russia (RUE)	Paramonov 2011, Paramonov 2012, Paramonov 2014	11	72	103
Russia: North European Russia (RUN)	Polevoi et al. 2018, Polevoi and Salmela 2014, Przhiboro 2017, Starý 2019	5	15	149
Russia: Northwest European Russia (RUW)		0	1	141
Russia: South European Russia (RUS)	Paramonov 2015, Paramonov 2018b, Paramonov 2019b	7	0	65
Russia: North Caucasus (NC)	Gavryushin 2011, Gavryushin 2014, Gavryushin 2015, Lantsov 2011, Lantsov 2014a, Lantsov 2014b, Lantsov 2015a, Lantsov 2015b, Lantsov 2017a, Lantsov 2017b, Lantsov 2019, Lantsov 2020, Pilipenko 2017	15	7	154
San Marino		0	0	0
Serbia	Kolcsár et al. 2017a, Kolcsár et al. 2017b	3	38	109
Slovakia	Starý 2011a, Starý 2011b, Starý and Stubbs 2015	5	0	320
Slovenia		0	6	113
Spain	Eiroa and Carles-Tolra 2019, Eiroa et al. 2020, Gavryushin 2012, Hancock et al. 2015, Hancock 2020, Hancock and Hewitt 2020, Hancock and Starý 2019, Kolcsár et al. 2015b, Mederos and Eiroa 2015, Mederos and Eiroa 2016, Mederos and Eiroa 2018, Mederos et al. 2019, Mederos et al. 2020, Starý 2011b, Starý 2014a, Starý 2019, Starý 2020a	43	5	181
Sweden	Andersson and Brodin 2019, Ennerfelt 2020, Fritz and Lindström 2013, Hågvar et al. 2010, Lindström and Fritz 2015, Salmela 2010b, Salmela 2011a	16	7	223
Switzerland	Starý 2011a, Starý and Stubbs 2015	2	0	298
Ukraine	Oboňa et al. 2017	2	0	243
United Kingdom	Drake and Stubbs 2014, James and Kramer 2019, Kramer 2018, Starý and Stubbs 2015	5	0	228
Turkey (European part)	Koç et al. 2016	65	0	67
